# Healthy humans can be a source of antibodies countering COVID-19

**DOI:** 10.1080/21655979.2022.2076390

**Published:** 2022-05-21

**Authors:** Nileena Velappan, Hau B. Nguyen, Sofiya Micheva-Viteva, Daniel Bedinger, Chunyan Ye, Betty Mangadu, Austin J. Watts, Robert Meagher, Steven Bradfute, Bin Hu, Geoffrey S. Waldo, Antonietta M. Lillo

**Affiliations:** a Biosciences Division, Los Alamos National Laboratory, Los Alamos, NM 87547, USA; b Experimental division, Carterra Inc, Walnut Creek, CA, 94568, USA; c Center for Global Health and Department of Internal Medicine, University of New Mexico Health Sciences Center, Albuquerque, NM, 87131, USA; d Biotechnology and Bioengineering Department, Sandia National Laboratories, Livermore, CA, 94551, USA

**Keywords:** SARS-CoV-2, COVID-19, antibody, antibody cocktail, ACE2 competitor, neutralizing antibody, detecting antibody, COVID-19 diagnostics, COVID-19 therapeutics

## Abstract

Here, we describe the isolation of 18 unique anti SARS-CoV-2 human single-chain antibodies from an antibody library derived from healthy donors. The selection used a combination of phage and yeast display technologies and included counter-selection strategies meant to direct the selection of the receptor-binding motif (RBM) of SARS-CoV-2 spike protein’s receptor binding domain (RBD2). Selected antibodies were characterized in various formats including IgG, using flow cytometry, ELISA, high throughput SPR, and fluorescence microscopy. We report antibodies’ RBD2 recognition specificity, binding affinity, and epitope diversity, as well as ability to block RBD2 binding to the human receptor angiotensin-converting enzyme 2 (ACE2) and to neutralize authentic SARS-CoV-2 virus infection in vitro. We present evidence supporting that: 1) most of our antibodies (16 out of 18) selectively recognize RBD2; 2) the best performing 8 antibodies target eight different epitopes of RBD2; 3) one of the pairs tested in sandwich assays detects RBD2 with sub-picomolar sensitivity; and 4) two antibody pairs inhibit SARS-CoV-2 infection at low nanomolar half neutralization titers. Based on these results, we conclude that our antibodies have high potential for therapeutic and diagnostic applications. Importantly, our results indicate that readily available non immune (naïve) antibody libraries obtained from healthy donors can be used to select high-quality monoclonal antibodies, bypassing the need for blood of infected patients, and offering a widely accessible and low-cost alternative to more sophisticated and expensive antibody selection approaches (e.g. single B cell analysis and natural evolution in humanized mice).

## Graphical Abstract

**Figure:**
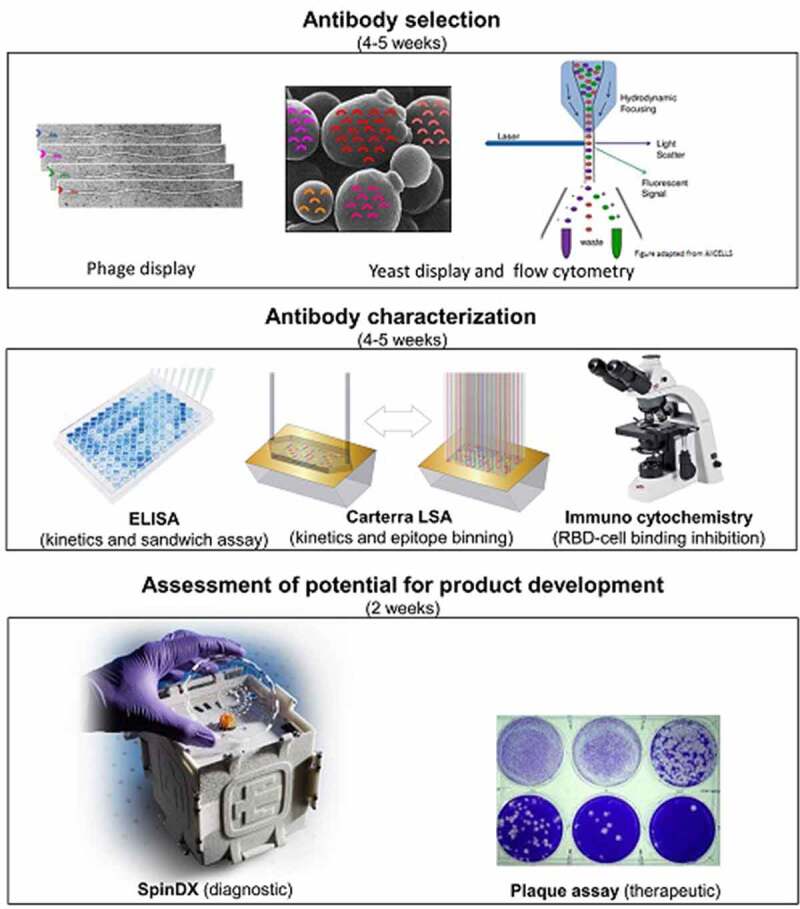

## Highlights

- Selected 16 antibodies specific for RBD2 and 2 cross reacting with RBD1
- Affinities of 8 best-performing antibodies for RBD2 range from 27 to 800 nM
- All best performers appear to target a different epitope of RBD and form 18 pairs
- E01, F07, and S01 neutralize viral infection at low nanomolar NT50 when paired up
- Pair F07+S01 can detect spike protein at LoD 160 fM

## Introduction

The world is currently experiencing a pandemic of coronavirus disease 2019 (COVID-19), caused by a novel severe acute respiratory syndrome (SARS)-like coronavirus (SARS-CoV-2). This is the seventh known coronavirus to infect humans, and the third causing widespread diseases, following SARS-CoV-1 and Middle East Respiratory Syndrome Coronavirus (MERS-CoV) [1–4]. SARS-CoV-2 is 79% identical and 86.1% homologous to SARS-CoV-1 and is classified into the genus betacoronavirus in the family Coronaviridae [2].

Surface spike protein mediates both SARS-CoV-1 and SARS-CoV-2 entry into the host cells by binding the human angiotensin-converting enzyme 2 (ACE2) through the receptor-binding domain (RBD). Important differences between the two virus’ RBDs might partly account for the higher infectivity of SARS-CoV-2. Recent cryo-EM studies revealed that the SARS-CoV-2 spike protein is an asymmetrical homotrimer with a single RBD in the ‘up’ conformation and the other two in ‘down’ conformation allowing easier interaction with ACE2 [5]. The same study also identified the ACE2 receptor-binding motif (RBM) of SARS-CoV-2 RBD (RBD2), revealing that most of the sequence differences between SARS-CoV-1 RBD (RBD1) and RBD2 reside in this region. Later, crystal X-ray diffraction structure of RBD2 in complex with ACE2 [6] identified contact residue differences between RBD1 and RBD2 and led to hypothesize that some of them might account for the higher affinity of SARS-CoV-2 for ACE2 (RBD2: 4.7 nM *vs* RBD1: 31 nM). These conclusions were further supported by biophysical characterization of the complex RBD2-ACE2 *vs* the RBD1-ACE2 [7,8]. Notably, a recent systematic bioinformatic analysis found that the only predicted conformational B cell epitope in RBD2 is located within the RBM [9]. This information suggests that the RBM might have high immunogenicity and would be a suitable target for development of SARS-CoV-2-specific antibodies with potent neutralizing function. RBD2ʹs enhanced exposure within the spike protein trimer, its immunogenicity profile, and the identification of residues within its sequence contributing to stronger interaction with ACE2, together with the ability to produce this protein separately from the whole spike protein monomer [10–12], makes RBD2, and in particular its RBM region, a good target for vaccine design, selection of therapeutic antibodies, and development of highly specific immunodiagnostic reagents for SARS-CoV-2.

Countermeasures against COVID-19 and possible future epidemic/pandemic include the development of pathogen-neutralizing therapeutics and pathogen-detecting diagnostics, capable of retaining their function in spite of the pathogen’s natural tendency to mutate. Passive immunization by administration of pathogen-specific monoclonal antibodies (mAbs) has recently been proposed as a way of fighting antibiotic resistant bacteria [13–16], whereas antibody-based diagnostics are popular for accurate, and field-deployable analysis of complex samples, owing to limited sample-processing needs, high specificity, and minimal instrumentation requirements [17–20]. Whether used for therapeutic or diagnostic applications the effectiveness of antibodies depends, among other factors, on their ability to specifically recognize the pathogen and its natural variants. Although antibodies are well known for their specificity, the use of multiple antibodies binding multiple regions (epitopes) of the target antigen instead of one allows for a more precise recognition of the antigen. Furthermore, a suite (or cocktail) of antibodies targeting multiple epitopes of an antigen indispensable to a microorganism’s pathogenicity is better suited to counter mutation-driven immunoescape than antibodies targeting one epitope. Not surprisingly, anti-SARS-CoV-2 immunotherapeutics cocktails of two antibodies targeting distinct epitopes of RBD2 (Bamlanivimab + Etesevimab, from Eli Lilly [21–23]; and Casirivimab + Imdevimab from Regeneron [24]) were among the first treatments authorized for emergency use by the FDA. Notably, the Regeneron cocktail seems to be effective (albeit less potent [25]) against SARS-CoV-2 circulating variants with the highest number RBD2 mutations (South Africa (*B.1.351) and* Brazil (P1): K417N/T, E484K, N501Y). It is also not surprising that most of the 23 SARS-CoV-2 immunoassays that have received emergency FDA approval [26,27] are sandwich assays where one antibody acts as the capturer and the other as the detector. Most of these assays target the viral nucleocapsid protein (N protein), although one targets RBD2 [26].

The Eli Lilly cocktail was derived from two separate patients who recovered from COVID-19 in North America and China, whereas the Regeneron cocktail was obtained by both natural evolution in humanized mice, and sorting of individual B cells from previously infected human patients. The resources and facilities necessary for such sophisticated antibody selections are not commonly available to the wider scientific community. ‘Naïve’ antibody libraries, on the other hand, can be rapidly accessed in a pandemic and can be selected in vitro through display technologies, allowing high-quality monoclonal antibodies discovery [28]. This approach conveniently bypasses the need for blood of infected patients, and offers a more accessible and less expensive alternative to single B cell analysis and natural evolution in humanized mice. Additionally, the specificity of the selected antibodies (which often is unsatisfactory for antibodies selected in animals [29]) can be fine-tuned and/or focused on a particular region of the antigen, by depleting the naïve library of antibodies binding to a close-relative antigen. This pre-subtraction strategy is not easily applicable to animal selections. In vitro selection of phage display libraries has been successfully used to select single-domain antibodies [30–32], or single-chain antibodies [33,34] recognizing RBD2. Most of this previously described work relies on the use of only one display platform, which usually affords a limited number of hits and does not allow to screen for features desirable for good manufacturability. Furthermore, the selection methodology was not designed to encourage recognition of different epitopes of the target molecule nor (with one exception [33]) to direct selection to the ACE2-interacting portion of RBD2.

In vitro selection of large libraries of affinity reagents (human antibodies [35] or peptides [36]), through phage [37,38] and/or yeast [39] display technology, has enabled us to select high-quality reagents for multiple targets including viral [40,41], bacterial [36,42–44], and mammalian [45–49] proteins, as well as peptides, and whole cells. In some cases, using pre-subtraction strategies, we have identified exquisitely specific reagents (e.g. peptides distinguishing metastatic from non-metastatic melanoma cells [45], and antibodies discriminating between phosphorylated from non-phosphorylated peptides [50]). In our most recent selections, toggling between phage and yeast display platforms has allowed us to select larger suites of affinity reagents, which, being well expressed on yeast, are also likely to be well folded and stable [51]. Finally, yeast display has facilitated affinity maturation of our most promising anti-*plague* antibodies [52]. Here, we describe the isolation of 18 unique anti-SARS-CoV-2 human antibodies from a naïve antibody library derived from 40 healthy donors [35]. This is an approach also recently used successfully by other groups [53,54]. The selection of this library synergized phage and yeast display technologies and used a combination of subtractive and non-subtractive selection strategies meant to obtain diverse antibodies (i.e. recognizing different regions of the target antigen) and directed selection to the RBM of RBD2. We also report on the characterization of this antibody set for RBD2 recognition specificity, binding affinity, and epitope diversity, as well as for ability to inhibit ACE2-RBD2 binding, and neutralize SARS-CoV-2 infection in vitro. We present evidence supporting that most of these antibodies selectively recognize RBD2, target at least eight different epitopes, and have high potential for therapeutic and diagnostic application.

## Results

### Phage and yeast display selection, and identification of unique monoclonal antibodies

A phage display library of human scFvs was enriched for RBD2 binders by multiple iterations of the following steps: 1) incubation with biotinylated antigen; 2) capturing the RBD2-bound phage-displayed scFvs onto streptavidin beads; 3) eluting phage; and 4) amplifying eluted phage. We employed different selection strategies to increase the chances of obtaining antibodies binding orthogonal epitopes of RBD2. Non-competitive selections were aimed to target the most antigenic RBD2 epitopes, whereas selections including competitor RBD1 were aimed to obtain antibodies to the RBM region of RBD2, where RBD1 and RBD2 differ the most. Various formats of RBD2 and RBD1 were used as selection/counter-selection antigens: 1) avitagged and biotinylated [55] RBD2 (AB-RBD2); 2) chemically biotinylated RBD2 or RBD1 (CB-RBD2/1); and 3) unlabeled RBD2 or RBD1 (RBD2/1). At the elution step of the phage selection cycle, we employed either HCl (non-selective elution) or excess non-biotinylated antigen to favor elution of more specific phage-displayed scFvs. Various combination of antigen formats and elution strategies resulted in seven different selection strategies (Supplementary Tables 1 and 2). Phage selections were monitored by calculating increments in output phage with respect to the first selection cycle. After the 3rd round of non-competitive selections 1, 2 and 3, these increments were 6-, 80- and 600-fold respectively. For competitive selections 4, 5, 6 and 7, increments were 3.4-, 45.0-, 12.7- and 113.0-fold respectively. The lower increments in competitive selections are expected considering the higher stringency of these selections due to addition of a competitor. The final phage outputs of the phage selections were subcloned in yeast.

**Figure 1.**
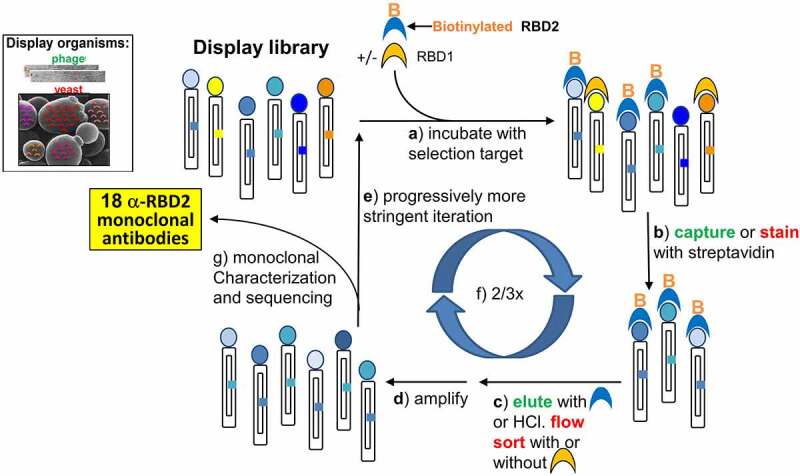
**Selection of monoclonal antibodies by in vitro evolution of display libraries.** Here we show the display technology-based selection cycle (both phage and yeast display were used in tandem) that led to the isolation of 18 anti-SARS-CoV-2 spike protein RBD (RBD2). Display organisms (either phage or yeast) are represented schematically to highlight the displayed antibody (colored circles) and the gene coding for it (colored square inside the organism’s body). Selection steps **a** through **f** are described in the figure. Actions that are color-coded in green and red pertain to phage and yeast selections, respectively. Subtractive selection strategies used non-biotinylated SARS-CoV-1 RBD (RBD1, Orange half-moon) during the incubation step (**a**). Selection of RBD2-specific antibodies was encouraged by adding excess non-biotinylated RBD2 (blue half-moon) during the phage display elution step, and non-biotinylated RBD1 during sorting (**c**) An iterative cycle representing phage and yeast display selection is shown. Each phage particle or yeast cell is represented by: an outer cylinder (the body); an inner cylinder (the plasmid) with a colored little square on one of its sides (the gene encoding the displayed antibody); and a colored dot on the smaller side of the outer cylinder (the displayed antibody). The dot and little square on any given cylinder have the same color, signifying that each phage particle or yeast cells displays only one antibody while also harboring the gene encoding that same antibody. Micrographs of filamentous phage and yeast are shown on the upper left corner of the figure and are indicated as ‘phage’, written in green, and ‘yeast’, written in red, respectively. This color coding allows to distinguish parts of the steps written by each arrow within the cycle, as pertinent to either phage display (green writing) or yeast display (red writing). SARS-CoV-2 RBD (RBD2) is indicated by blue half-moons, some of which bare an orange capital B on the convex top indicating biotinylation. SARS-CoV-1 RBD (RBD1) is indicated by orange half-moons. Sets of cylinders are at the beginning and end of each arrow. The set with the most colors (i.e. highly diverse antibody population) indicates the initial library (top left of the cycle), the sets with only shades of blue represent the isolated population of antibodies that binds to RBD2, before (1 copy of each antibody) and after (multiple copy of each antibody) amplification. The various steps written by each arrow are the following: a) incubate [the display library] with selection target (biotinylated RBD2 alone or mixed with unbiotinylated RBD1 are shown on top of the first arrow as a blue half-moon with an orange B and an orange half-moon, respectively); b) capture [written in green] with streptavidin bead. Stain [written in red] with fluorescent streptavidin; c) elute [written in green] with either [blue half-moon] or with HCl. Flow sort [written in red] ± [orange half-moon]; d) amplify; e) iterate in progressively more stringent conditions; f) sequence and characterize single clones.

Yeast display libraries were enriched for RBD2 binders by incubation with biotinylated RBD2, staining of RBD2-bound yeast with fluorescently labeled streptavidin and anti-SV5 tag (SV5 is expressed with the scFv and this staining ascertains yeast expression), isolating doubly stained yeast (i.e. yeast displaying RBD2-binding scFvs) by flow cytometry sorting, and amplifying sorted yeast. As for phage selections, we employed different strategies to increase the chances of obtaining antibodies recognizing orthogonal epitopes of RBD2 with high specificity (Figure 1). We also used the various antigen formats described above plus an additional one. The spike protein of both SARS-CoV-1 and SARS-CoV-2 is a trimer of a protein composed of two domains, S1 and S2. RBD is part of S1 domain [56]. Therefore, we introduced avitagged S1 from SARS-CoV-2 to sort antibodies that recognize RBD2 in a more native context. Before the first sort, yeast display libraries from competitive phage selection (except for selection 4, the least enriched library), bound RBD2 between 2- and 5-fold better than RBD1, they also showed comparable binding to RBD2 and S1. The details of the various sorting strategies are described in Supplementary Table S3. We monitored the sorting progress by calculating increments in percentage of doubly labeled yeast with respect to the first selection round. At the 3rd round of non-competitive selection, these increments ranged from 28- to 40-fold respectively. For competitive selections increments ranged from 8- to 21-fold.

Plasmids encoding yeast-displayed scFvs were purified from the sorted yeast and were subcloned in *E. coli*. Four hundred single plasmids were sequenced to identify selected monoclonal scFvs. Competitive selections yielded 14 unique clones (B04-H05) while non-competitive selections yielded 4 unique clones; S01, R04, R09, and R26 (Supplementary Figure 1). A comparison of our antibodies CDRs with a set of antibodies published on the antibody society website (http://opig.stats.ox.ac.uk/webapps/covabdab/) revealed the uniqueness of our antibodies. In particular, we found that: 1) only a few single CDRs or CDR pairs were shared between the two groups; 2) none of these CDRs were CDRH3 (the biggest contributor to specificity of antigen recognition [57]); 3) none of our antibodies shared the entire set of CDRs; 4) only three of our antibodies share CDRL3; and 5) antibody E01 did not share any of its CDRs (Supplementary Figure 2).

**Figure 2.**
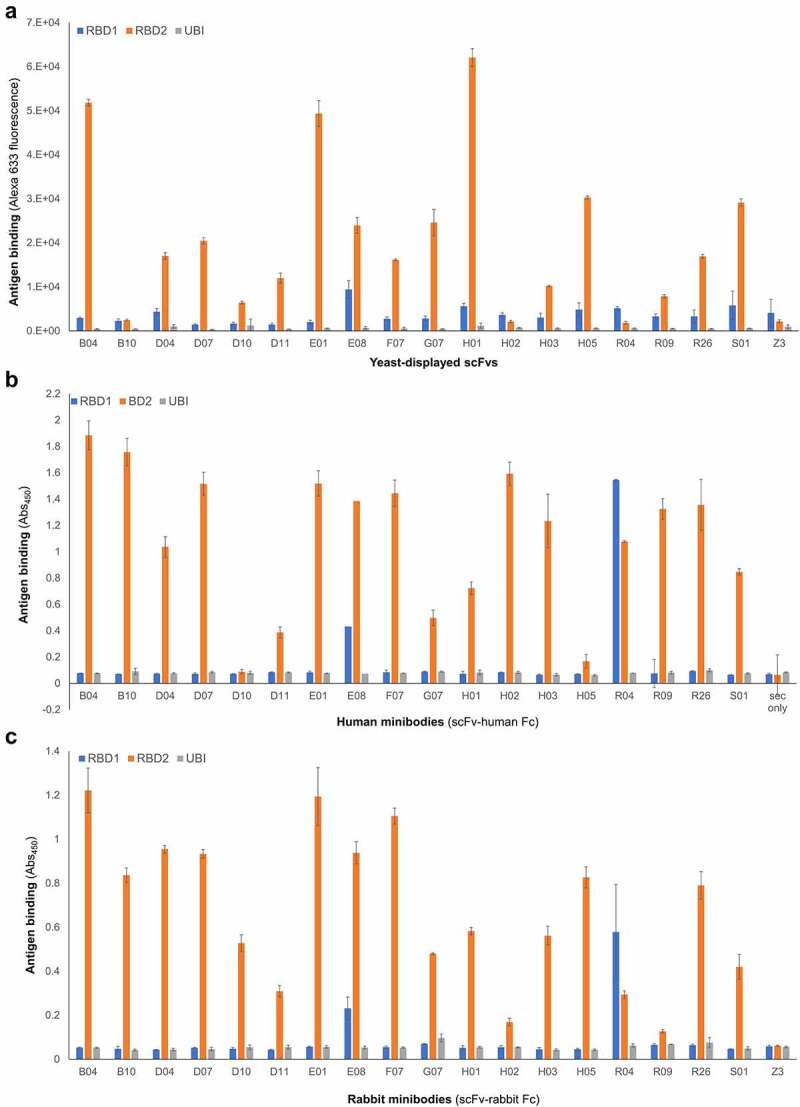
**Affinity and specificity of selected antibodies.** Relative binding affinity of (**A**) monoclonal yeast-displayed scFvs (measured at antigen concentration below saturation) and minibodies, i.e. scFv-Fc chimeras (human Fc, **B**; rabbit Fc, **C**). Data include binding to a negative control antigen (ubiquitin, UBI), and a negative control human anti-influenza M2 antibody (**A** and **C**) called Z3. The height of each bar is an average of three measurements and the error bars correspond to the standard deviations calculated for each set of three measurements Three bar graphs are shown one on top of the other (top: A; middle: B, and bottom: C). X-axes indicate the antibody corresponding to each set of blue, orange, and gray bars. The titles of these axes are: Yeast displayed scFv (graph A); Human minibodies (scFv-human Fc) (graph B); Rabbit minibodies (scFv-rabbit Fc). Y-axes represent antibody binding as a function of either yeast-bound fluorescent streptavidin (graph A), and anti-human or anti-rabbit antibody (HRP conjugated) bound to plate-immobilized minibody (graphs B and C). The titles of these axes are: Antigen binding (Alexa 633 fluorescence) (Graph A); Antigen binding (Abs450) (Graphs B and C). The bar colors represent binding to RBD1 (blue bars), RBD2 (orange bars), and ubiquitin (negative antigen, gray bars). For most of the antibodies, the blue bars’ height is comparable to the gray bars’ height, and the orange bar is taller by a factor of 10. Antibody E08 and R04 are exceptions for which there is a significant interaction with RBD1 (blue bar higher than gray bar) which in the case of antibody R04 is stronger than the interaction with RBD2 (blue bar higher than orange bar). Negative antibodies Z3 (anti influenza used for graph A and C) show only background binding to each antigen (low bar height).

Binding of yeast-displayed scFvs to RBD2 *vs* RBD1 was analyzed by flow cytometry (Figure 2A). We also produced soluble scFvs expressed in tandem with either human (Figure 2B) or rabbit (Figure 2C) fragment crystallizable Fc (scFv-Fc, called minibodies), and analyzed them by ELISA. As expected, all antibodies recognized RBD2. Also not surprisingly, competitive selections yielded a lower percentage of antibodies cross-reacting with RBD1 than non-competitive selections (7% and 25% respectively). Notable antibodies are B04 and E01, which exhibited the highest specificity for RBD2, and antibodies E08 and R04, which recognized both RBDs, with the former preferring RBD2 and the latter preferring RBD1. Of the two assays described above, the flow cytometry-based one, which used yeast displayed antibodies and sub-saturating antigen concentration, was the most reliable for determination of relative affinities. Therefore, based on data in Figure 2A, we chose the 9 and 2 highest affinity antibodies from competitive and non-competitive selections, respectively, together with the only antibody preferring RBD1 over RBD2 (R04), for further studies. Kinetic study of yeast displayed scFv’s binding to RBD2 or RBD1 included: 1) measuring antigen binding of yeast displayed antibodies at various concentrations of biotinylated antigen; 2) plotting binding *vs* antigen concentration; and 3) fitting the data to the Michaelis and Menten equation (also called one-site binding equation, Supplementary Figure 3A). The resulting affinity constant (K_D_) values ranged from ~14 to 290 nM (Supplementary Table 4), with scFvs B04, E01 and E08 having the highest affinity for RBD2. Minibody’s K_D_ values were determined by surface plasmon resonance (SPR) only (sensograms not shown) and ranged from ~4 to 900 nM, with E08, H02, and E01 being the top 3 performers (Supplementary Table 4).

### IgG production and characterization

**Figure 3.**
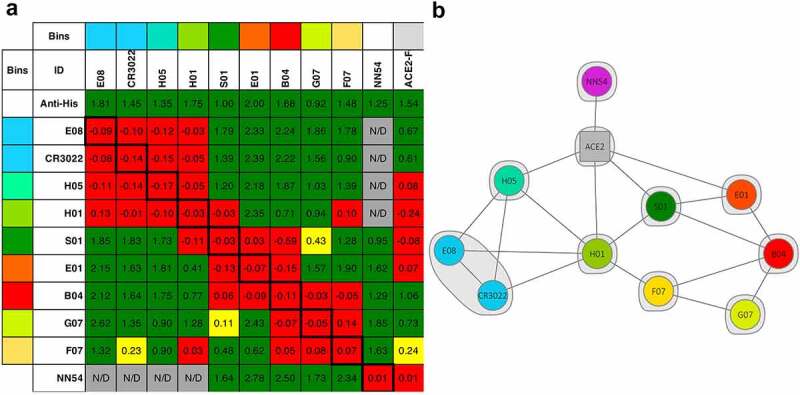
**Antibody epitope binning by surface plasmon resonance (SPR).** IgGs were tested pairwise for their ability to bind SARS-CoV-2 spike protein’s receptor binding domain (RBD2-His tag) by SPR. An anti-His tag antibody was used as a positive control non-competitive antibody. Commercial anti-RBD2 antibodies CR3022 and NN54, and recombinant human receptor angiotensin-converting enzyme 2-fragment crystallizable chimera (ACE2-Fc) were included in the analysis. SPR competition data is represented as a heat map (**A**) and displays intersections of two proteins (IgG or ACE2) with the immobilized antibody shown as rows and the injected analyte antibody shown as columns. Non-competing interactions (sandwiching) are shown as green, competing as red, and mildly competitive as yellow cells. Antibody pairs that were not analyzed (N/D) are indicated by grey cells. Notice that the global cutoffs shown for most ligands are 0.2 for the red to yellow transition and 0.25 for the yellow to green transition. However, cutoffs were adjusted individually for ligands H01, S01, B04, and G07 as their binding kinetics were quite different. Therefore, sometime different color cells bear similar values (e.g. S01/G07 = 0.11 = yellow, while F07/H01 = 0.10 = red). The network plot rendition of the SPR data (**B**) indicates competing IgG or ACE2/IgG pairs connected by a line, whereas all the non-competing IgGs are isolated. The different colors indicate antibodies targeting different epitopes (epitope bin) based on their interaction with RBD2 in the presence of either other antibodies or ACE2-Fc. An epitope bin is also represented by the gray shading covering E08 and CR3022 which share an identical competition profile *Portion A on the left* shows a colored grid with cells indicating ‘bins’ at the top and left edge of the grid. Bin colors are blue, aqua, light green, dark green, orange, red, apple green, and yellow. Only antibodies E08 and antibody CR3022 have same color bins, signifying that they target the same RBD2 epitope. All the other antibodies, and human cell receptor ACE2 (Fc-derivatized, ACE2-Fc) have different color bins indicating that they all target different RBD2 epitopes. The second row of the grid shows the immobilized antibodies used in each assay, in separate cells (E08, CR3022, H05, H01, S01, E01, B04, G07, F07, and NN54). ACE2-Fc is also there in the last cell. The second column of the grid indicated the injected antibody used in each assay, in separate cells. It starts with anti-His (antibody against histidine tag) and continues with the antibodies indicated above. ACE2-Fc is not included. The remaining cells in the grid represent the result of each assay (i.e. test of the interaction of immobilized antibody with RBD2, and of injected antibody with the ‘RBD2-immobilized antibody’ complex). Red cells indicate lack of binding of the injected antibody, signifying that immobilized and injected antibody bind the same RBD2 epitope (competitive RBD2 binding). Green cells indicate binding of the injected antibody signifying that immobilized and injected antibody bind different RBD2 epitopes (non-competitive RBD2 binding). Yellow cells indicate something in between a competitive and non-competitive interaction. Gray cells indicate assays that were not run. There are numbers inside each cells indicating the strength of the interaction. The first row of interactions includes only green. The second, third, and fourth rows start with four red cells and continue with five green cells, one gray cell, and a final green cell. The fifth row starts with five red cells and continues with three green cells, one red cell, one gray cell, and one red cell. The sixth row starts with three green cells and continues with four red cells, one yellow cell, two green cells, and one red cell. The seventh row starts with four green cells and continues with three red cells, three green cells, and one red cell. The eighth row starts with four green cells and continues with five red cells and two green cells. The ninth row starts with four green cells and continues with one yellow cell, one green cell, three red cells, and two green cells. The tenth row starts with one green cell and continues with one yellow cell, one green cell, one red cell, two green cells, three red cells, one green cell, and one yellow cell. The eleventh row starts with four gray cells, followed by five green cells, and two red cells. *Part B on the right*, shows a set of colored dots surrounded by a gray halo. Each dot includes the name of an antibody or ACE2-Fc. Each dot is connected to other dots by lines indicating competitive binding of the two connected antibodies to RBD2. Dots of the same color (only E08 and CR3022) are wrapped by the same gray halo indicating that they bind to the same epitope of RBD2. Antibody NN54 (pink dot) is only connected with ACE2-Fc (gray dot). ACE2-Fc is connected to H05, H01, S01, and E01 (aqua, light green, dark green, and orange, respectively). H05 is connected also to E08, CR3022, and H01 (blue, blue, light green, respectively). H01 is connected also with E08, CR3022, S01 (dark green), and F07 (yellow). S01 is connected also with B04 (red), and E01 (orange). E08 is connected also with CR3022. F07 is connected also with B04 and G07 (apple green). E01 is connected also with B04. G07 is connected also with B04.

Based on scFvs and minibodies kinetics and preliminary epitope binning studies (data not shown), antibodies E01, S01, F07, G07, B04, E08, H01, and H05 were converted to IgG isotype 1 (IgG1) format. Antibody R04 was also selected for conversion, due to its preference for RBD1 (unique among the antibodies described here). IgGs were produced at 100 mL scale, in yields ranging from 0.42 to 0.27 mg/mL (Table 1). First, we analyzed some of the IgGs’ specificity for RBD2 by FLISA (Supplementary Figure 4A and B, respectively). The antigens used were biotinylated RBD2, RBD2 fused with super folder green fluorescent protein (sfGFP), and RBD2 within the S1 unit of spike protein, avitagged and biotinylated. As expected, every IgG analyzed bound specifically to RBD2 in any of the formats used, except R04. Next, IgGs were analyzed by SPR to measure K_D_s of interaction with RBD2 (Table 2, and Supplementary Figure 3B). The range of K_D_ values was 50–2000 nM, with E01 having the highest affinity (K_D_: 48 nM); S01, F07, and B04 having mid-range affinities at 100–300 nM; and G07 having the lowest affinity (2000 nM). For the majority of the antibodies tested, we observed a progressive drop of affinity (higher K_D_s) going from scFv to minibody to IgG format (Supplementary Table 4 and Supplementary Figure 5). Finally, antibodies’ ability to bind RBD2 non-competitively was evaluated by sandwich ELISA (Supplementary Figure 6) and by SPR (Figure 3). From now on, a set of two antibodies binding non competitively to RBD2 will be referred to as a ‘pair’. The 8 antibodies analyzed by SPR combined in a total of 18 pairs and some of them formed pairs with previously described anti-RBD2 antibodies CDR3022 [58,59] and NN54 [60] (Figure 3). SPR was also used to determine whether our IgGs were able to compete with ACE2 in binding to RBD2, revealing that IgGs E01, H01, H05, and S01 (and possibly F07) are competitors. Antibody + antibody and antibody + ACE2 interactions with RBD2 suggest that each of the eight IgGs behave uniquely and therefore is in its own ‘bin’ (i.e. interacts with an RBD2 epitope not overlapping with any other). An extension of analyses on the COVID-19 antibody community reported by Hastie *et al.* [61] revealed that some of our antibodies belong to four different communities (2, 5, 6, and 7), details of the community assignments for the antibodies are given in Supplementary Figure 1. We have also conducted antibody clonotype analyses for all the selected antibodies finding that IGHV1-V6 are represented. The data presented in Supplementary Figure 1 provide information on the specific IGHV for each antibody, the L clonotype is indicated for some antibodies (based on available information).

**Table 1.** Yield of IgG production

Antibody	Yield (mg/mL culture)^a^	Yield quality (folds above minimum)^b^
B04	0.33	3.3
E01	0.42	4.2
E08	0.29	2.9
F07	0.41	4.1
G07	0.41	4.1
H01	0.35	3.5
H05	0.41	4.1
R04	0.27	2.7
S01	0.38	3.8

^a^from 100 mL culture.

^b^yield/minimum yield (0.1 mg/mL).

**Table 2.** Summary of data obtained for the best antibodies in various formats

Antibody name (antigen)	Affinity for RBD (k_D,_ nM)^a^		Non-competitive RBD2 binders	Competitive RBD2 binders	Recognize D614G mutant?
E01 (RBD2)	13.9 ± 1.3	scFv	E08, F07, G07, H01, H05, CR3022, and NN54	B04, S01 and ACE2	Yes
	21.5 ± 2.12	Minibody			
	90.0 ± 22.0	IgG			
S01 (RBD2)	44.1 ± 2.5	scFv	E08, F07, H05, CR3022, and NN54	B04, E01, H01, and ACE2	Yes
	22.7 E + 3	Minibody			
	170.0 ± 40.0	IgG			
F07 (RBD2)	67.5 ± 6.8	scFv	E01, E08, H05, S01, CR3022, and NN54	B04, G07, and H01	Yes
	61.5 ± 3.5	Minibody			
	300.0 ± 65.0	IgG			
G07 (RBD2)	60.3 ± 5.5	scFv	E01, E08, H01, H05, S01, CR3022, NN54, and ACE2	B04, and F07	Yes
	63.9 E + 3	Minibody			
	320.0 ± 76.0	IgG			
B04 (RBD2)	13.9 ± 1.5	scFv	E08, H01, H05, CRR3022, NN54, and ACE2	E01, F07, G07, and S01	NT
	56.8 ± 3.5	Minibody			
	210 ± 42.4	IgG			
E08 (RBD2)	13.9 ± 1.8	scFv	B04, E01, F07, G07, S01, and ACE2	H01, H05, and CR3022	NT
	3.6 ± 2.0	Minibody			
	27.0 ± 3.2	IgG			
H01 (RBD2)	66.4 ± 5.1	scFv	B04, E01, and G07	E08, F07, H05, S01, CR3022, and ACE2	NT
	281.0 ± 162.6	Minibody			
	800.0 ± 440.0	IgG			
H05 (RBD2)	16.7 ± 4.0	scFv	B04, E01, F07, G07, and S01	E08, H01, CR3022, and ACE2	NT
	51 ± 8.6	IgG			
R04 (RBD2)	293.3 ± 24.0	scFv	ND	ND	NT
	127.5 ± 7 0.0	Minibody			
	190.0 E + 3	IgG			
R04 (RBD1)	175.1 ± 20.3	scFv	NT	NT	NT

^a^Determined either by flow cytometry (scFvs) or by surface plasmon resonance, SPR (minibodies or IgGs).

^b^ + C = with competition; CB = chemically biotinylated target antigen.

^c^-C = no competition; AB = target antigen biotinylated through the avitag.

### Diagnostic application using pairs of antibodies: detection of spike and heat inactivated SARS-CoV-2 virus by ELISA and SpinDx

In the next series of assays, we tested our antibodies for recognition of full-length trimeric spike protein, both in purified form or as part of the whole virus. At the time of these experiments, the predominant virus variant carried an aspartic acid (D) to glycine (G) mutation at amino acid position 614 of spike protein (D614G variant) [62,63], therefore our antibodies were also tested for recognition for D614G spike.

Based on their performance in the previously described experiments, we selected antibodies E01, F07, G07, and S01 (in various pairwise combinations) for both sandwich ELISA (Supplementary Figure 6) and SpinDx experiments (Supplementary Figure 7). For the sandwich ELISA, we immobilized the capturing antibody on plate and chemically labeled the detecting antibody with HRP. The detection of the captured antigen was monitored by absorbance at 450 nm (Abs_450_) of the HRP substrate conversion product. SpinDx technology is a portable centrifugal microfluidic device that utilizes microsphere (beads) sedimentation for rapid detection of antigens, as previously reported by investigators at Sandia National Laboratories, Livermore, CA, USA [64–68]. The SpinDx assay is a bead-based sandwich immunoassay where antigen-specific antibodies are covalently bound to silica microspheres and used to capture antigen in a complex sample matrix. Captured antigen is then detected with a second antibody labeled with fluorescent dye. The sandwich immunoassay complex is loaded into a microfluidic disc preloaded with density media and processed in the SpinDx device to allow sedimentation of the complexes via centrifugation. As the bead complexes pass through the density media gradient, any unbound antigen or detector antibodies are excluded from sedimentation, thus allowing a homogeneous, no-wash assay for rapid and sensitive antigen detection. Limit of detection (LoD) and limit of quantification (LoQ) for spike (ELISA and SpinDX) and whole heat-inactivated virus (ELISA only) were determined using noise signals for negative control analytes (myoglobin and rhino coronavirus for spike and whole virus detection, respectively, in ELISAs) or for no analyte (SpinDx). These signals plus 3 or 8 standard deviations were inputted in the equations defining the ‘signal to analyte-concentration’ relationship for each antibody pair analyzed (Supplementary Figure 7) to obtain LoD and LoQ, respectively (Table 3).

**Table 3.** Sensitivity of two sandwich immunoassays

Assay	Antibody pair^a^	Spike		Spike D614G		Whole virus		
		LoD^b^	LoQ^c^	LoD	LoQ	LoD		LoQ
		(pM)		(pM)		(TCID50^d^/mL)		
**ELISA**^e^	S01/F07	1.22	18.2	664.2	1786.1	> 2.5 E + 4	>53.7E+4	
	S01/G07	4.1	24.1	125.4	425.6	> 2.5 E + 4	>53.7E+4	
	E01/G07	21.2	64.1	15.6	75.0	1.8E+4	12.0E+4	
	E01/F07	39.1	111.2	85.6	225.8	2.5 E + 4	53.7E+4	
**SpinDx**^f^	S01/F07	0.16	26.5	N/D^g^				
	G07/S01	1.9	43.1					
	S01/G07	10.3	26.5					

^a^(antigen-capturing IgG)/(antigen-detecting IgG).

^b^Limit of Detection.

^c^Limit of Quantification.

^d^Tissue culture infectious dose 50 (i.*e. the* dilution of *virus* required to infect 50% of the cell monolayers).

^e^Enzyme-linked immunosorbent assay.

^f^Portable Multiplexed bead-based Immunoassay platform.

^g^Not determined.

Our ELISA epitope binning experiments (Supplementary Figure 6) revealed that S01 and E01 acting as capturing antibodies form optimal pairs with G07 and F07 as detection counterparts. Therefore, we used S01/G07 or F07 and E01/G07 or F07 antibody combinations for the ELISA-based detection experiments (Supplementary Figure 7). We found that S01 was the best capturing antibody for detection of wild-type spike protein (Supplementary Figure 7A), whereas E01 was optimal for detection of the D614G spike variant (Supplementary Figure 7B). Antibody pairs S01/F07 and S01/G07 detected wild-type spike at LoDs 1.22 and 4.1 pM respectively, whereas pairs E01/G07 and E01/F07 detected D614G spike variant at LoDs 15.6 and 85.6 pM, respectively. Overall, antibody S01 seems to be more sensitive to changes in the spike amino acid sequence (31-fold minimum reduction of wt *vs* D614G spike detection sensitivity) than antibody E01 (2-fold maximum reduction of wt *vs* D614G spike detection sensitivity). This behavior might depend on these two antibodies recognizing different epitopes (Figures 3). Sandwich ELISA was also used to determine the limit of detection for heat inactivated virus (Supplementary Figure 7D) and revealed that pair E01/G07 affords the lowest LoD (1.8E+4 half tissue culture infectious dose, TCI50).

Due to limited availability of whole virus, SpinDx was only used for detection of spike protein (Supplementary Figure 7C). Prior to performing LoD experiments, a preliminary single-point antigen concentration screening allowed identification of the pair configurations affording the highest signal/noise ratio (data not shown). Pairs S01/F01 (with S01 used as capturing antibody) and S01/G7 (with S01 used as capturing or detecting antibody) were selected. As for sandwich ELISA, pair S01/F07 performed best (spike protein LoD = 0.16 pM) and allowed ~8-fold more sensitive detection of wt spike than in ELISA. Interestingly, when S01 paired up with antibody G07 it performed better (five-fold higher sensitivity) as detecting rather than capturing antibody (spike protein LoD = 1.9 *vs*. 10.3 pM).

### Identification of antibodies competing with ACE2 for RBD2 binding, and exploration of their therapeutic potential

**Figure 4.**
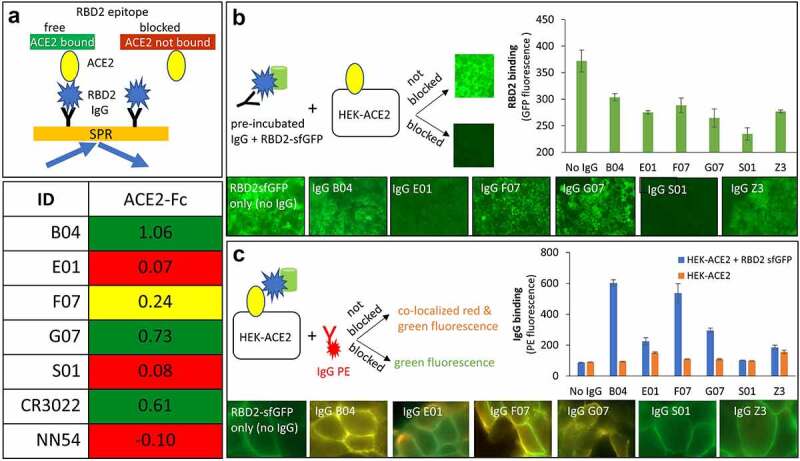
**Antibody interference with binding of SARS-CoV-2 spike protein’s receptor-binding domain (RBD2) to ACE2.** Presented are data for five anti-RBD2 antibodies (B04, E01, F07, G07 and S01) along with controls. A heat map plot of SPR data (**A**) depicts normalized ACE2-Fc binding to RBD2 captured by immobilized antibodies. The green, red and yellow cells indicate non-competing, competing and mildly competing binding relationships, respectively. Antibodies CR3022 and NN54, known to not compete and compete with ACE2, respectively, were included as controls. (**B** & **C**) Evaluation of the effect of the antibodies on interaction between HEK cells constitutively expressing ACE2 (HEK-ACE2) and RBD2-sfGFP chimera (green fluorescence). Both panels provide three pieces of information: a graphical representation of the experimental design, quantitative fluorescence data in bar graph format (error bars show standard deviation of triplicate experiment) and representative microscopy images for each of the antibodies. Data obtained without IgGs (only RBD2sfGFP) and anti-influenza M2 IgG Z3 were used as a positive control for absence of inhibition (**B**) and binding (**C**). Binding to HEK ACE2 cells in the absence of RBD2sfGFP was used as specificity control (**C**, bar graph)Upper left corner provides a graphical representation of SPR assay (light orange rectangle with blue arrows going towards and outwards) involving IgG (black Y shape), RBD2 (blue polygon), and ACE2 Fc (yellow oval). In this assay, immobilized IgGs were allowed to bind to RBD2, followed by ACE2. If the RBD2 epitope for ACE2 is free, then the yellow oval attaches to black Y – blue polygon complex. However, if the IgG occupies the ACE2 binding epitope and blocks the interaction, the yellow oval is shown unbound. Lower right corner provides the SPR colored bins similar to the description for Figure 3. The data shows B04, G07, and CR3022 as green indicating that they allow simultaneous binding of IgG and ACE2 when incubated with RBD2. E01, S01, and NN54 antibodies are shown with red shades indicating that they block ACE2 binding when complexed with RBD2. IgG F07 is shaded yellow indicating intermediate value for blocking of ACE2 binding. Figure 4B provides three sets of information; a graphical representation of the assay (right), microscopy images of cells (bottom row) and a bar graph providing quantitative values corresponding to the images. Here, RBD2-sfGFP chimera is depicted as blue polygon and green cylinder, IgG is represented as black Y shaped molecule, HEK-ACE2 cells as black square with yellow oval (ACE2). For the data shown in Figure 4B the pre-incubated IgG-RBD2 sfGFP (black Y-blue polygon-green cylinder) was added to HEK-ACE2 cells. If the IgG did not block the binding to cell surface ACE2, the RBD2-sfGFP was captured by ACE2 resulting in green fluorescent cells. However, if the IgG blocked ACE2-RBD2 interaction, the cells would not be fluorescent. A small square image of fluorescent and non-fluorescent microscopy images are given indicating non-blocking and blocking interactions. The bottom panel for Figure 3B shows microscopic images for No IgG (cells are fluorescent), IgG B04 (cells are fluorescent), IgG E01 (cells are non-fluorescent), IgG F07 (cells are fluorescent), IgG G07 (cells are fluorescent), IgG S01 (cells are non-fluorescent), IgG Z3 (cells are fluorescent). The bar graph on the right hand side of Figure 4B shows highest amount of fluorescence for no IgG control and lowest amount of fluorescence for IgG S01. The other antibodies from highest to lowest are B04, F07, G07, Z3, and E01. Figure 3C also has three sections: a graphical depiction of the assay (left), microscopic images of cells (bottom), and bar graph providing quantitative data (right). In this assay (as shown on left), HEK cells expressing ACE2 (shown as black square with yellow oval on top) are bound to RBD2-sfGFP (blue polygon-green cylinder). This complex is incubated with PE labeled IgG (red Y with attached red polygon). If the IgG-PE binds to the cell bound RBD2-sfGFP then the cells will have yellowish-orange color of co-localized signal. If the IgG-PE complex is blocked from binding the cells will remain green fluorescent. The bottom panel shows microscopic images for RBD2-sfGFP (no IgG) as green fluorescent cells, bright yellow fluorescence for cells incubated with IgG B04 F07 and G07, green fluorescence for cells incubated IgG E01, IgG S01, and Z3. The bar graph on the lower right shows PE fluorescence of bound IgG on y-axis and IgGs on the x-axis. Blue bars represent IgG binding to HEK-ACE2 + RBD2 sfGFP and orange bars indicate binding to HEK-ACE2 cells only (in the absence of RBD2-sfGFP). Both bars are at low background level for no IgG control. For IgG B04, F07, and G07, a high blue bar and low orange bar are shown. For IgG E01, IgG S01, and Z3, both blue and orange bars are low, similar to the no IgG control.

It has been established that ACE2 acts as the cellular doorway that allows SARS-CoV-2 entry into many types of cells, resulting in COVID 19 disease [69]. Therefore, in an effort to explore the therapeutic potential of our antibodies, we tested their ability to block ACE2-RBD2 interaction in three different assays. The first assay was part of the SPR assay described above and depicted in Figure 3 that involved antibody + ACE2-Fc interactions with RBD2. Figure 4A captures the relevant part of the data in Figure 3. Five out of eight tested antibodies, E01, H01, H05, S01, and possibly F07 competed with ACE2-Fc binding to RBD2. We identified E01, H01, and S01 as the strongest competitors. Notably, H01 competition was even superior to that of NN54, which in this experiment served as a positive competitor control.

The second assay included immunocytochemistry analysis (Figure 4B) of RBD2 binding to human embryonic kidney (HEK) cells with stable expression of ACE2 receptor. We applied RBD2-sfGFP chimera, and either unlabeled (Figure 4B) or phycoerythrin (PE)-labeled anti RBD2 antibodies (Figure 4C). We tested B04, E01, F07, G07, and S01 in IgG format. An anti-influenza M2 antibody, Z3, was used as a negative RBD2 competitor control. In the first set of experiments (Figure 4B), unlabeled antibodies were tested for their ability to block RBD2-sfGFP binding to ACE2 expressed on the cell surface. By measuring cell-associated green fluorescence, we found that E01 and S01 were the strongest inhibitors of RBD2-ACE2 interaction (lower fluorescence = more efficient blocking). The remaining antibodies, B04, F07, G07, including the negative control Z3 did not significantly inhibit RBD2 binding to the native ACE2 receptor. In a second set of experiments (Figure 4C) we tried to characterize the epitope targeted by the anti-RBD2 antibodies. First, we pre-incubated RBD2-sfGFP with ACE2 receptor-expressing HEK cells. This step was followed by removal of the unbound RBD2-sfGFP and second incubation with PE-labeled anti-RBD2 antibodies. Thus, results with singly labeled GFP cells will indicate that the PE-labeled anti-RBD2 antibody is specific to the epitope occupied by the ACE2 receptor, while double labeled cells will signify that the anti-RBD2 antibody recognized epitope outside of the ACE2 binding site. The results from this assay indicated that E01 and S01 specifically bind to the RBD epitope occupied by ACE2, whereas antibodies B04, F07, and G07 could bind outside of the ACE2-occupied epitope since we were able to detect both red and green fluorescences. Collectively, our immunocytochemistry experiments confirmed the results from the SPR assay demonstrating that antibodies E01 and S01 are competitive inhibitors of ACE2-RBD2 interaction (Figure 4A). Interestingly, we did not observe mild inhibition of ACE2-RBD2 interaction by F07 as revealed by the SPR experiment. This might be due to the different format of ACE2 presentation (i.e. receptor expressed on the cell surface versus soluble protein in the SPR assays). We further validated the specificity of antibody binding to RBD2 versus ACE2 receptor by comparison of fluorescence intensity generated by PE-labeled antibodies incubated with ACE2 293 HEK cells and the parental 293 HEK strain not expressing ACE2 receptors. All tested antibodies demonstrated specific labeling of the ACE2-expressing cells in the presence of RBD2 (Figure 4C, bar graphs).

**Figure 5.**
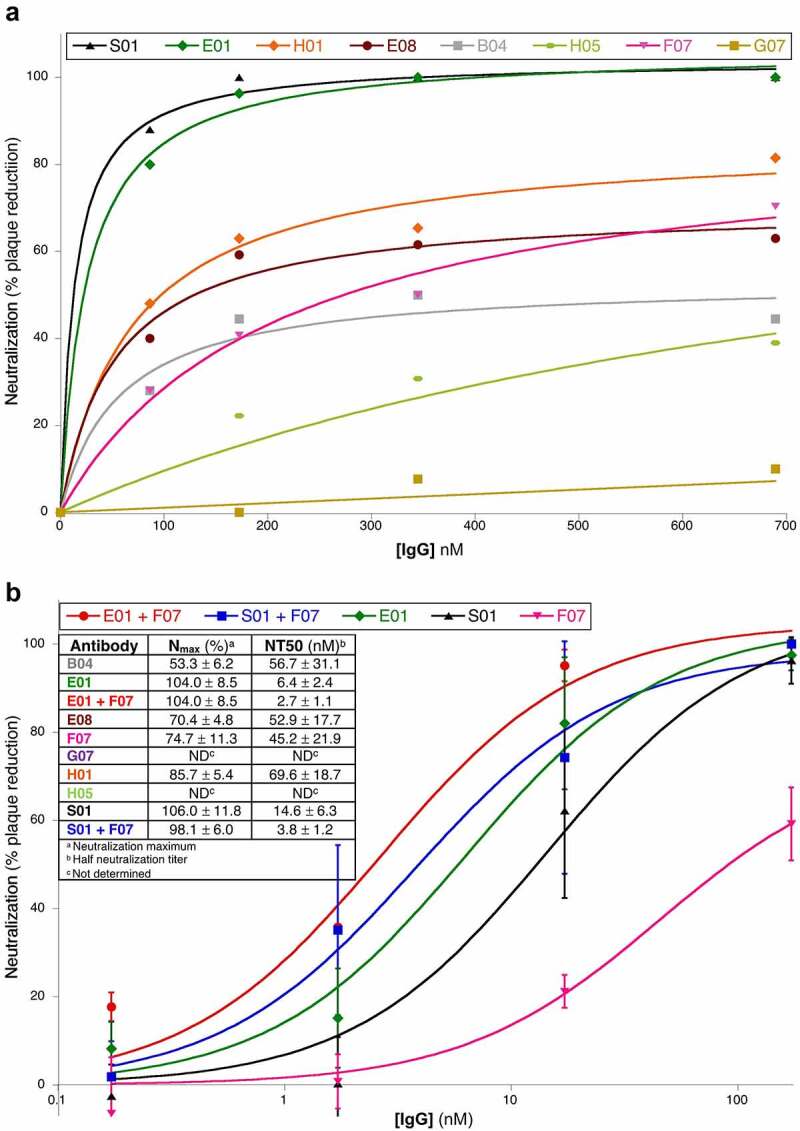
**In vitro neutralization of authentic SARS-CoV-2.** A set of 8 best performing anti-SARS-CoV-2 RBD were tested for their ability to neutralize authentic SARS-CoV-2 infection of VeroE6 cells in a preliminary screen (**A**). The three IgGs with the lowest half neutralizing titers (NT50) were retested in triplicate alone or in combination (**B**). This figure includes two scatter plots and one table. The first plot is indicated as part A. The X-axis (titled [IgG] nM) indicates the antibody concentrations used for the in vitro neutralization assay, and it goes from 0 to 700. The Y-axis (titled Neutralization (% plaque reduction)) indicates the lowering of plaque formation in the presence of any given antibody compared to the absence of antibody treatment, and it goes from 0 to 100. We show data obtained for antibodies: S02 (black line, fitting data points shown as black triangles); E01 (green line, fitting data points shown as green diamonds); H01 (orange line fitting data points shown as orange diamonds); E08 (maroon line fitting data points shown as maroon circles); B04 (gray line fitting data points shown as gray squares); F07 (pink line fitting data points shown as pink triangles); H05 (apple green line fitting data points shown as apple green ovals); G07 (mustard yellow line fitting data points shown as mustard yellow squares). S01 and E01 lines plateau at 100% neutralization. Other antibodies have lower plateaus. From the highest to the lowest plateau we have H01, E08, F07, B04, and G07. The B section of this figure (below A) includes a table of neutralization maxima (Nmax) and half neutralization titers (NT50) for each antibodies or antibody pairs. Some of these values were calculated from the curves in the A plot (corresponding to low performing antibodies) others were obtained from the curves in the B plot where each data point corresponds to experiments performed in triplicate with corresponding standard deviation. The antibodies in the B plots are E01, F07, and S01 (same color lines and bullets as in A plot). Antibodies E01 and S01 were also tested in combination with F07 (red line, fitting data point shown as red circles and blue line, fitting data points shown as blue squares, respectively). All of the curves except F07 plateaued at 100% neutralization, at or below 100 nM concentration. The 100% plateauing order was E01+ F07; E01; S01; S01+ F07; and F07. From table in B Nmax(%)/NT50(nM) for antibodies B04, E01, E01+ F07, E08, F07, G0, H01, H05, S01, and S01 + 07, were as follows: 53.3 ± 6.2/56.7 ± 31.1, 104 ± 8.5/6.4 ± 2.4,104 ± 8.5/2.7 ± 1.1, 70.4 ± 4.8/52.9 ± 17.7, 74.7 ± 11.3/45.2 ± 21.9, ND (not determined), 85.7 ± 5.4/69.6 ± 18.7, ND, 106.0 ± 11.8/14.6 ± 6.3, 98.1 ± 6.0/3.8 ± 1.2, respectively

Our third assay was based on *in vitro* neutralization of SARS-CoV-2 infection of ACE2-expressing HEK cells (Figure 5). A preliminary screen of anti-RBD2 antibodies B04, E01, E08, F07, G07, H01, H05, and S01 was performed at four dilutions, to assess the relative neutralizing potency (Figure 5A). The highest potency antibodies E01, S01, and F07 were selected for further studies, also based on orthogonality of their RBD2 binding sites (see epitope binning in Figure 3) and their performance in immunocytochemistry assays (Figure 4). We tested individual antibodies as well as pairs E01+ F07 and S01+ F07 (Figure 5B). Results suggest that E01 is the most potent antibody to block SARS-CoV-2 entry, followed by antibody S01 (NT50 ~ 6 and 15 nM respectively). When combined with antibody F07 the neutralization efficiency of these two antibodies increased by ~2 and 4-fold, respectively.

## Discussion

Human monoclonal antibodies, like the ones presented herein, offer the opportunity to develop both accurate point of care diagnostics and safe/effective therapeutics. Ideally, these antibodies recognize the pathogen with high specificity and high affinity and preserve their effectiveness against pathogen variants, targeting different epitopes of an antigen indispensable to the pathogen survival. Developing antibodies with these characteristics is possible using in vitro selection of human antibody libraries derived from healthy donors, by display technologies. Importantly, these antibody libraries can be rapidly accessed at the onset of a pandemic (infected blood is not needed), or even before the onset of a pandemic (if pathogens with the potential to cause a pandemic can be predicted). The naïve scFv library used in our selections [35] was constructed from total RNA in human peripheral blood lymphocytes, using degenerate oligonucleotides (although even using domain specific 5’ and 3’ primers, sufficient similarity to support amplification of off target V domain regions can be achieved [70]). Diversity was further enhanced by VL and VH recombination mediated by cre-recombinase. Deep sequencing result estimated the total diversity of this library to exceed 10^11^ unique clones. As noted by Moon *et al.* [71] such large naïve libraries can yield antibodies *against virtually any antigen*. We attribute our success in selecting an excellent pool of antibodies to the diversity of our library, in combination with carefully orchestrated selection protocols. Furthermore, in vitro selections offer a more accessible and less expensive alternative to natural evolution in humanized mice or human B cell isolation pipelines. In vitro selection also allows for the isolation of highly pathogen-specific antibodies, especially when the library is depleted of antibodies interacting with close relatives of the target antigen. Additionally, the use of complementary display platforms such as phage and yeast (like in this and previous work [54,61]) allows to select antibodies that are more likely to retain immunoreactivity outside the display context, tend to be better folded and more stable, and thus can be rapidly developed into commercial products. Importantly, selection by yeast display (where up to 10^5^ scFv copies can be surface exposed [72]) allows isolation of low affinity antibodies that would probably be lost if only phage display was used. This leads to higher sequence diversity of selected antibodies. Nevertheless, we recognize that there are disadvantages when antibodies are selected in vitro from display libraries (e.g., lack of affinity maturation, risk of auto-reactivity due to artificial pairings/lack of tolerance check points, developability issues [73]). However, we also think that some of these shortcomings can be mitigated by coupling phage display with yeast display, which allows for antibody affinity maturation (yeast display selection of antibody variants obtained by error-prone PCR can lead to 5–100 fold affinity increment [74]) and for selection of antibodies with glycosylation patterns similar to human cell-produced antibodies. Additionally, preincubation of display library with tissue arrays, and/or human cells, and/or human proteins might deplete the library of antibodies prone to tissue cross-reactivity, guarding against selection of potentially toxic antibodies.

The importance of switching antigen labeling format between phage display-based selection and yeast display-based sorting was a key ‘lessons-learned’ during this project. The competitive selection was likely more successful due to the fact that phage selection used CB-RBD2 and yeast sorting utilized AB-RBD2 and/or AB-S1. Similarly, non-competitive selection that used AB-RBD2 as antigen during phage panning, followed by yeast sorting strategies that used AB-S1 and/or CB-RBD1 were also successful. Conversely, the selection and sorting strategy that used only AB-RBD2 as antigen in both phage and yeast display selections only yielded antibodies whose antigen recognition is biased by the biotinylation method (i.e. they recognize AB-RBD2 but not CB-RBD2 nor unlabeled RBD2). The data presented here also suggest that chemical biotinylation is more suited than avitag-mediated biotinylation for phage panning. This is probably due to the higher level of biotinylation afforded by the former method. Furthermore, the use of avitagged antigens during subsequent yeast sorting allows to eliminate antibodies whose binding depends on chemical biotinylation and perhaps eliminates low affinity antibodies due to the lower biotinylation of the antigen.

Adoption of this in vitro selection strategy (Figure 1) has enabled us to isolate 18 single chain antibodies (Supplementary Figure 1), most of which recognize RBD2 with minimal cross-reaction with RBD1 (Figure 2). As a demonstration of the foldability/expressability of these antibodies, eight of the highest affinity scFvs were easily converted to IgGs and produced in high yield (Table 1). Based on SPR affinity constant (K_D_) measurement (Table 2), our antibodies fall in or below the 43-percentile within a set of 180 anti-RBD2 antibodies characterized by the La Jolla Institute of Immunology (https://covic.lji.org/database/). Importantly, due to the sequence variety of our antibodies, our eight highest affinity IgGs seem to recognize different RBD2 regions (epitopes) as shown by SPR (Figure 3) and sandwich ELISA (Supplementary Figure 6) epitope binning. This is an important feature for the development of effective therapeutic cocktails and accurate diagnostics. Although antibodies are well known for their specificity, the use of multiple antibodies binding multiple epitopes of the target antigen instead of one, allows for a more precise recognition of the antigen. Furthermore, when only one epitope is recognized, a mutation of the target antigen might result in immunoescape and consequent failure of the immunodiagnostics and immunotherapeutic strategy [75,76]. However, mutations spanning different regions of a functional antigen (e.g. RBD) are less likely to happen, since they might result in major structural changes and loss of function (e.g. inhibition of interaction with ACE2 receptor and host cell invasion). Therefore, a suite (or cocktail) of antibodies targeting multiple epitopes of an antigen indispensable to a microorganism’s pathogenicity is better suited to counter immune-escape than antibodies targeting one epitope. Based on in vitro studies (Figure 5), our highest affinity IgGs all protect (to a variable extent) ACE2-expressing HEK cells from infection with authentic virus. Interestingly, the most effective IgGs (lowest NT50 values, inset in Figure 5B) seem to work better in pairs. Namely, the NT50 of E01 and S01 decreases 2.4 and 3.8-fold, respectively, in the presence of F07. These NT50 values are in the 32-percentile within a set of 224 anti-RBD2 IgG subjected to a similar in vitro study at the La Jolla Institute of Immunology (https://covic.lji.org/database/). Ideally, a continuation of this work would analyze larger groups of orthogonal antibodies (e.g. adding E08 and H01 to the pairs tested here) both in vitro and in vivo, with wild type and SARS-CoV-2 variants, as a further step toward creating a powerful therapeutic cocktail. It is also exciting to note that antibodies E01 and S01 belong to COVID-19 antibody community 2 (binds to RBM) and the orthogonal binders B04, G07 & F07 belong to antibody community 5 (RBD Outer Face) based on community features detailed by Hastie et al. [61]. Antibody H01 was assigned to community 6 and antibodies H05 and E08 assigned to communities 7 (RBD Inner Face). Thus, the lead antibodies discovered from the naïve library used in our study span the same breadth of diversity (belongs to RBM, inner face, and outer face binders) obtained from alternative research approaches detailed by Hastie et al. [61]. Tan *et al.* [77] have studied the public clonotype antibodies obtained from multiple convalescent SARS-CoV-2 patients with the same IGHV gene and similar CDR H3 sequence. Antibody S01 belongs to the public clonotype for SARS-CoV-2 with IGHV3-66 NY motif in CDR H1, SGGS motif in CDR H2, and the signature Y58F somatic hypermutation present among the IGHV3-53/3-66 public clonotype antibodies[77]. This result also provides evidence toward utility of naïve antibodies libraries to discover public clonotype antibodies for emerging pathogens in the absence of convalescent serum.

Our six orthogonally binding IgGs combine in a set of 25 pairs, where each pair member binds RBD2 non-competitively with the other. Generally, antibody pairs are good candidates for highly accurate and sensitive ‘sandwich’ immunoassays, where the accuracy of detection relies on recognition of two different regions of the target molecule rather than one, and the sensitivity relies on both affinity of the capturing antibody for the target, and on the ability of capturing and detecting antibodies to bind the target non-competitively. While immunoassays are not as reliable or sensitive molecular diagnostic methods compared to polymerase chain reaction (PCR) tests [78], they have the great advantage of providing fast response diagnostic tests without sample purification and dependence on expensive/bulky instrumentation (e.g. can be pocket-size). Sandwich ELISA is one of the assays that we have used in this work to test a set of four antibody pairs for detection of both spike protein (wild type – wt and D614G variant) and whole, heat-inactivated SARS-CoV-2 virus. The lowest limit of wt spike protein detection using this assay (1.2 pM) was achieved by antibody pair S01 (capturing IgG) and F07 (detecting IgG conjugated with HRP). Whereas, the lowest limit of whole virus detection (1.8E+4 TCID50/mL, a clinically relevant concentration [79]) was achieved by antibody pair E01 (capturing IgG) and G07 (detecting IgG conjugated with HRP). While sandwich ELISA is a gold standard sensitive and quantitative method, it requires extensive laboratory equipment, trained personnel, and long incubations to obtain results. Therefore, a second sandwich immunoassay used in this work was based on SpinDx technology, a robust and sensitive point of care (POC) technology highly valuable in settings where a central testing laboratory is not readily available. In general, POC diagnostics enable rapid results, fast patient notification, and immediate decision-making by medical professionals. POCs have been used for decades for detection of disease biomarkers, pregnancy testing, glucose monitoring, and infectious disease testing, to name a few [65], with the most widely used POC being lateral flow immunoassays (LFIA) [80]. There have also been recent developments in microfluidic technology for POC, but these are not as widely used [81]. Currently, 25 SARS-CoV-2 antigen detection rapid tests have been approved for emergency use (EUA) in the United States (FDA website, as of 05/21/21). The majority of the approved tests are lateral flow assays, eight non-LFIA tests require processing in a laboratory setting with proprietary equipment for readout. The LFIAs tests are fast; however, most are qualitative, require follow-up testing due to higher chances of false-negative results compared to RT-PCR [82], and many do not differentiate between SARS-CoV-1 and SARS-CoV-2 as detailed in the FDA authorization documents (FDA.gov). SpinDx technology, on the other hand, has the capability of a lab equipment, with the advantage of portability, sensitivity comparable to ELISA due to its miniaturized ‘sandwich immunoassay on beads’ design, quantitation, and minimal sample volume requirement [64–68]. The same set of four antibody pairs tested in ELISA was also tested for detection of wt spike protein by SpinDx. In this assay, as in ELISA, the lowest limit of detection (160 fM) was achieved by antibody pair S01 (capturing IgG) and F07 (detecting IgG conjugated with Alexa Fluor 647).

## Conclusions

In vitro selection of a naïve human scFv library, using a combination of phage and yeast display and counter selection strategies, has enabled selection of a diverse set of 18 anti-RBD2 antibodies. Nine of these antibodies were easily converted to IgGs, and eight of these IgGs (B04, E01, E08, F07, G07, H01, H05, and S01) were able to recognize eight different RBD2 epitopes. Six of these eight antibodies were capable of inhibiting authentic SARS-CoV-2 virus’ infection of ACE2-expressing HEK cells, with the three most potent antibodies exhibiting even higher protection when used in pairs (E01+ F07 and S01+ F07). Four of the 25 antibody pairs formed by the 8 orthogonally binding IgGs were tested in sandwich immunoassay. Pair S01+ F07 detected spike protein at 160 fM by SpinDx assay and pair E01+ G07 detected whole virus at 1.8E+4 TCID50/mL by ELISA. As a whole, these results show the power of in vitro antibody selection based on multiple display platforms. More importantly, some of the selected antibodies seem to be good candidates for sensitive and accurate point of care diagnostics as well as therapeutic cocktails. On-going and future experiments include identification of the RBD2 epitopes targeted by our best antibodies; in vitro and in vivo studies of antibody cocktails other than the one tested here; and development of highly sensitive POC diagnostics other than SpinDx, such LFA with a Quantum Dot-based read out.

## Materials and methods

### Experimental design

We used the receptor-binding domain of SARS-CoV-2 spike protein (RBD2) as the target antigen of our phage and yeast display selection. SARS-CoV-1 RBD was used as a counter selection target to focus the selection on the RBM or RBD2 and to verify the specificity of the selected antibodies. Unique antibodies were identified by sequencing and characterized by flow cytometry and ELISA for affinity, specificity, and uniqueness of interaction with RBD2. Best performers were converted to IgGs and further characterized. SPR was used for affinity measurements and epitope binning. SPR, competitive ELISA, and fluorescent microscopy was used to study the influence of our antibodies on ACE2-RBD2 interaction. The diagnostic potential of some antibody pairs (where each pair member binds RBD2 non-competitively) was tested by measuring limit of detection of trimeric SARS-CoV-2 spike protein by sandwich ELISA and SpinDx, and of whole virus by sandwich ELISA. The therapeutic potential of the entire IgG suite was tested by in vitro studies, measuring the level of neutralization of HEK cells infection by active SARS-CoV-2. Figure 1 provides a graphical summary of the antibody selection and characterization workflow.

### Antigen preparation

Avitagged-biotinylated SARS-CoV-2 RBD (RBD2, SPD C82E9), S1 protein (S1N C82E8), unlabeled RBD2 (#SPD C52H3), SARS-CoV-1 RBD (RBD1, #SPD S52H6), trimeric SARS-CoV-2 spike protein (#SPN C52H9), and SARS-CoV-2 spike protein variant D614G (#SPNC52H3) were purchased from AcrosBiosystems. Unlabeled RBD2 and RBD1 were chemically biotinylated using the ThermoFisher EZ link NHS-LC-LC biotinylation kit (ThermoFisher, #21343, the resultant biotinylated RBD2/1 were called CB-RBD2/1) or the Novus Biologicals Lightning link kit (#370-0010), according to manufacturer’s recommendations. The CB-RBD2/1 biotinylation level was measured using Fluorescence Biotin Quantitation Kit (ThermoFisher, #46610) according to the manufacturer’s recommendations and was 2.5:1 (RBD1) and 3.1:1 (RBD2).

RBD2 and RBD1-sfGFP chimeras were produced in-house. Human codon-optimized DNA construct encoding for the RBD domain (residues 333–529) of SARS-CoV-2 spike protein (GenBank YP_009724390.1) was obtained as a gift from Dr Erik Procko. The construct was N-terminally fused to an influenza HA signal peptide and C-terminally fused to sfGFP. A 6-Histidine tag was added to the C-terminus of sfGFP for protein purification purpose. A similar human codon-optimized construct was synthesized for the RBD domain (residues 320–515) of SARS-CoV-1 spike protein (GenBank QKY_12178.1). The protein fragment length was obtained by analyzing the sequence alignment of the spike proteins from the two virus strains and optimizing sequence length for best comparison with RBD2. These constructs were cloned into pcDNA3.1 (+) (ThermoFisher, #V79020) via NheI-XhoI sites. Both RBD1-sfGFP-6His and RBD2-sfGFP-6His recombinant proteins were expressed using Expi293F expression system kit cells (ThermoFisher, #A14635) following the manufacturer’s protocol. Briefly: 1) Expi293F cell cultures were grown in Expi293 Expression Medium at 37°C, 125 rpm, 8% CO2 to 2 × 10^6^ cells/mL; 2) DNA plasmid was added at 500 ng/mL cell culture; 3) transfection enhancers were added after 20 hours of transfection; 4) and cells were cultured for an additional ~ 5 days. Cell cultures were centrifuged at 3,000 rpm for 15 minutes, and cell culture medium was collected and filtered for subsequent protein purification.

RBD1-sfGFP-6His and RBD2-sfGFP-6His proteins secreted in culture medium were incubated with Talon resin pre-equilibrated in binding buffer – 50 mM Tris-HCl pH 7.4, 300 mM NaCl, 10% glycerol using 30:1 volume ratio for 2 hours with gentle shaking at room temperature. Resin was then loaded onto the column, and the column was washed three times with 10x column volumes of binding buffer. 40 mL elution buffer (same as binding buffer plus 150 mM imidazole) was added to the column and the flow through was collected in 8 fractions. Fractions containing the protein (as revealed by fluorescence measurements) were pooled together (~40 mL) and dialyzed against 2 L 1x PBS overnight at 4°C to dilute the imidazole. Dialysis buffer was changed 2 times and proteins were collected, concentrated to 500 μg/mL and 360 μg/mL for RBD1-sfGFP-6His and RBD2-sfGFP-6His, respectively in final buffer containing 1x PBS, 10% Trehalose. Protein concentration was determined using BCA Protein Assay Kit (Pierce #23225). Protein solutions were split into 500 μL aliquots and stored at −80°C.

### ScFv antibody selections by phage display

Seven different selection strategies were adopted, where the selection antigen was always biotinylated RBD2. Selections 1, 2, and 3 were non-competitive (Supplementary Table 1) and used avitagged-biotinylated RBD2 (AB-RBD2). Selection 4, 5, 6, and 7 were competitive (Supplementary Table 2) and used chemically biotinylated (EZ link) RBD2 (CB-RBD2). For any given selection 180 μL of phage-displayed scFv library [60] (pfu 10^13^/mL) was pre-treated by blocking with 0.5–1% bovine serum albumin (BSA) in 1xPBS for 0.5–1 h with rotation. For selections 6 and 7, library pre-treatment included one more step, i.e. CB-RBD1 was incubated with the blocked library for 1 h with rotation, and RBD1-bound phage was removed by incubation with washed and blocked streptavidin beads (Dynabeads M-80, ThermoFisher, #11205D) and magnetic separation. The pretreated library was incubated with either AB-RBD2 (selections 1, 2, and 3), CB-RBD2 plus RBD1 (selections 4 and 5) or CB-RBD2 (selections 6 and 7) and incubated for 1 h at RT for 1 h. The antigen/counter antigen concentrations are indicated in Supplementary Tables 1 and 2. About 10 μL of blocked streptavidin beads were added to 200 μL of antigen-incubated library, and KingFisher magnetic particle purification system (ThermoFisher, #5400000) was used to incubate and wash the beads and to elute bead-captured phage. Three PBST (1X PBS 0.1–0.05% Tween 20) and three PBSLT (1X PBS 0.005% Tween 20) or PBS washes were performed for each selection cycle. The wash times are indicated in Supplementary Tables 1 and 2. Non-specific elution was conducted by dispersing the washed beads in 150 μL 0.1 N HCl for four minutes and neutralizing the pH with 50 μL of 1.5 M Tris pH 8.8. Specific elution was performed by incubation with excess non-biotinylated RBD2 for 30 minutes. 10 mL of Omnimax T1 grown at mid log phase (Abs_600_ or OD600 = 0.5) at 37°C were infected with eluted phage for 1 h, at 37°C static incubation, collected by centrifugation and plated on 2XYT agar plates containing carbenicillin (50 μg/mL) and glucose (3%). Standard phage amplification using M13 helper phage and PEG precipitation protocols were used to prepare the input phage subsequent rounds of selection [50]. Cells infected with the output phage from the 3^rd^ selection cycle were used for plasmid preparation (Qiagen, #27106). ScFv-encoding genes were PCR amplified using primers (GTTCTGGTGGTGGTGGTTCTGCTAGAGGCGCGC and GCAGTGGGTTTGGGATTGGTT TGCC). These primers added flanking sequences to the scFv gene that allow homologous recombination with the yeast display vector upon yeast transformation (see section below). The PCR products were purified using Qiagen PCR purification kit (Qiagen, #28104).

### ScFv antibody selections by yeast display

The yeast display vector pDNL6^50^ plasmid was digested with restriction enzymes *BssH* II, *Nhe* I, and *Nco* I and purified using Qiagen PCR purification columns (Qiagen, # 28104). Vector and scFv fragments prepared as described above were co-transformed into EBY100 yeast cells using Yeast 1Kit (Sigma, #YEAST1) to allow cloning by gap repair [50]. The transformed yeasts were grown in selective media (SD/CAA [50]) and induced using SG/RCAA media as previously described [50]. The yeast cultures were grown in SD/CAA medium at 30°C, allowed to reach an OD600 > 2.0, and mixed 1:10 with SG/R CAA induction medium. Yeast expression induction proceeded at 20°C overnight with shaking (250 rpm). Induced yeast was washed with yeast washing buffer (1X PBS, 0.5% BSA, 20 mM EDTA) and incubated for 1 h at RT with shaking in the presence of different biotinylated antigens, with or without non biotinylated RBD1 (as indicated in Supplementary Table S3). Phycoerythrin labeled anti-SV5 antibody (anti-SV5 PE) was also included at 1 μg/mL to label the SV5 expression tag appended to the displayed scFv. After more washing, Streptavidin Alexa 633 (ThermoFisher, #S21375) was added to the yeast at 5 μg/mL to label the biotinylated antigen, following incubation and washing as described before. Yeast sorting was performed on FACS Aria (Becton Dickinson). Sorted yeast was amplified and induced for subsequent selection rounds.

### Yeast plasmid preparation and scFv gene sequencing

Plasmids from enriched yeast display libraries obtained from 3^rd^ and 4^th^ round selections were isolated from 2 mL o/n yeast cultures using a modified Qiagen miniprep procedure (Qiagen, #27106) where: 1) the buffer volumes were doubled; 2) after addition of buffer PE, 100 μL of glass beads (Sigma, #G8772) were added and the mixture was vortexed (maximum rpm) at RT for 10 min; 3) the final DNA solution was added to the same column in two 800 μL aliquots. Plasmids were transformed in One Shot *E. coli* Omnimax T1 (ThermoFisher, #C854003). Notice that due to the low concentration/purity of yeast plasmid solutions, the entire transformation suspension needs to be plated to obtain a few tens of colonies. Single *E. coli* transformants were used to inoculate 2XTY-Carb-Glu (50 μg/mL carbenicillin, 3% glucose) in the wells of a 96-well sterile plate, and the plate was incubated o/n at 37°C with rotation (900 rpm). About 2 μL of the o/n culture in each well were deposited on a 2XYT-Carb-glu agar plate. After o/n incubation at 37°C, the plate was submitted for Sanger sequencing service to GeneWiz together with PNL6 Forward and Reverse primers (CACTGTACTTTT AGCTCGTAC, TAGATACCCATACGACGTTC). About ~400 single colonies were sequenced from various sort libraries and the scFv sequence was analyzed to identify unique clones using Geneious Prime software. Plasmid encoding unique scFvs were miniprepped from the bacterial colonies and transformed back to EBY100 yeast cells for specificity and affinity measurements.

### Specificity of binding and kinetic study of yeast-displayed scFvs

Yeast colonies transformed with unique scFvs were picked from SD/CAA agar plates and grown in SD/CAA liquid culture. Yeast induction and antigen staining were performed as previously described. A 96-well filter plate (Millipore, #MSGVN22) was used for high throughput washes. The analysis was performed either on FACS Aria or FACS ACURI 600 plus flow cytometers (Becton Dickinson). The specificity and affinity measurements were performed using on RBD2, RBD1, or ubiquitin (control antigen) biotinylated with the lightning link kit. The anti-influenza A M2 protein scFv Z3^41^ was used as the negative control antibody. Specificity assays were performed at non-saturating 20 nM antigen concentration. Each measurement was obtained in triplicate and averaged. Dissociation constants for the highest affinity antibodies identified by this preliminary screening were obtained by: 1) measuring antibody binding (y) at various concentrations of CB-RBD2 (x, serial 2-fold dilutions from 500 to 0.8 nM); 2) plotting the data in Kaleidagraph (Version 4.5); and 3) fitting the data to the Michaelis Menten equation adapted to antibody binding: y = AB_max_*x/(K_D_+x), where AB_max_ = maximum antibody binding and; K_D_ = antibody affinity constant.

### Conversion of scFvs to scFv-Fc (minibody) and IgGs

The scFv genes from all 18 clones were excised from PDNL6 plasmids by restriction digestion with enzymes *BssHII* (NEB, #R0199S) and *NheI* (NEB, #R3131S) and gel purified. These genes were inserted into a yeast scFv-Fc expression vector pDNL9 sacB plasmids digested with the same restriction enzymes by ligation (T4 DNA ligase, NEB, #M0202). Ligation reactions were transformed in One Shot Omnimax T1 *E. coli* cells and four clones/ligation reaction were analyzed by colony PCR for the right size insert by using pNL6 FW and REV primers and DNA sequencing. Plasmids were prepared from selected clones and transformed in YVH10 yeast cells (in-house stocks, gift from Wittrup lab at MIT) using yeast transformation kit protocol. Transformed yeasts were plated on SD/CAA plates supplemented with tryptophan (final concentration 80 μg/mL). Single YVH10 colonies were further grown in SD/CAA liquid culture medium and induced following the yeast secretion protocols described by Wentz and Shusta [83]. Culture supernatants were used in the ELISA-based binding assays described below.

Selected scFvs were also converted to IgGs by inserting the amino acid sequences corresponding to the variable heavy (VH) and variable light (VL) antibody regions into a standard IgG1 scaffold. The resulting protein sequences were submitted to ATUM Inc. (Newark, CA, USA) for codon-optimized back-translation, gene synthesis, and expression as full-length IgG1 antibodies in HEK293 cells. IgGs were received as PBS solutions from ATUM and stored in small aliquots at −80°C before use in various assays.

### Soluble antibody characterization assays

#### Common steps in ELISA/FLISA/sandwich ELISA

For all these assays, a 96-well NUNC Maxisorp plate (transparent, #442404 or black, #43711) was coated with soluble antibody (minibody or IgG) in PBS (70 μL/well) either directly or through interaction with goat anti-human antibody (Southern Biotech Inc. #Fc-UNLB). Blocking was always done using 5% BSA solution in PBS (200 μL/well). Antigens and detecting IgGs (for sandwich assays) were added in 0.5% BSA-PBS solutions (70 μL); for one-concentration assays, antigen concentration was always 100 nM. Incubation steps were at 25°C for 1 hr, unless otherwise stated. Washing included three consecutive PBST (PBS + 0.05% Tween 20) and three consecutive PBSLT (PBS + 0.005% Tween 20) addition (300 μL/well) and removal. Upon adding 100 μL/well PBS the plate’s UV/vis absorbance or fluorescence was measured. Data were obtained in triplicate (except for epitope binning in Supplementary Figure 5, which was a qualitative test), and measurements’ average/standard deviation were calculated and plotted against antigen concentration using Microsoft Excel/Kaleidagraph.

#### Minibody ELISA

Maxisorp plates (ThermoFisher, #442404,) was coated with neutravidin (ThermoFisher, #31050) at 10 μg/mL (100 μL/well) overnight at 4°C or for 1 h at 37°C. The wells were washed twice with 1X PBS and blocked with 2% milk PBS for 1 h. 0.5–1 μg of lightning link-biotinylated antigens (RBD2, RBD1, or ubiquitin) were added and incubated for 30 minutes. Unbound proteins were washed with 2X PBS. Upon addition of primary antibodies (either human or rabbit minibody crudes, 100 μL/well), the plate was incubated for 1 h. Primary antibody solutions were removed and the plate was washed. Upon addition of HRP labeled secondary antibodies (anti-human, Abcam, #ab97165 or anti-rabbit, ThermoFisher, #G21234, 1:2000 dilution, final concentration 0.5 μg/mL), incubation was carried out for 1 h, and secondary antibody solution removed. Upon washing (4X PBST and 4X PBS), HRP activity was detected by adding its substrate 3,3’, 5,5’ tetramethylbenzidine dihydrochloride (TMB, Sigma #T0440, 100 μL/well). Once blue color started to develop the reaction was quenched by adding 1 M H_2_SO_4_ (50 μL/well). Absorbance at 450 nm was measured using spectrophotometer.

*IgG FLISA* [84].A black maxisorp plate was coated with goat anti-human antibody by 1) adding a PBS solution of antibody (25 μg/mL, 70 μL/well); 2) incubating the plate at 4°C overnight; and 3) upon removal of antibody solution, blocking with 5% BSA in PBS (250 μL/well, 1 h, 25°C). Anti-RBD2 IgGs were added to the plate (10 nM solutions in 0.5% BSA PBS, 70 μL/well), incubated for 1 h at 25°C, and removed. After washing, biotinylated antigens (LLB RBD1, LLB RBD2, AB fraction S1 of spike, and negative control LLB lysozyme), or RBD1/RBD2-sfGFP chimeras (produced in house) were added (100 nM solutions in 0.5% BSA PBS, 70 μL/well), followed by incubation and washes. When biotinylated antigens were used, a 200-fold diluted streptavidin-Alexa Fluor 633 solution (ThermoFisher, #S21375) was added (1:200 dilution in 0.5% BSA PBS, 100 μL/well), followed by washing, addition of PBS (100 μL/well) and plate reading at Ex/Em 595/660 nm. When sfGFP antigen chimeras were used, after washing and addition of PBS, the plate was read at Ex/Em 480/520 nm.

*Sandwich ELISA*. Each capturing IgGs were immobilized on a 96-well Nunc Maxisorp plate (ThermoFisher #12-565-136) by depositing 70 μL of a 340 nM IgG PBS solution in each well, incubating, and blocking. The analyte (either RBD2, trimeric spike protein from BEI, #NR-53257), whole heat-inactivated SARS-CoV-2 virus (BEI #NR-52287) (2.8E+5 TCID_50_/mL, diluted to 2.52 E + 5 TCID_50_/mL*0.69 = 1.7E+5 TCID_50_/mL, total virus particle in 70 μL = 1.2E+4), or negative controls myoglobin/Human coronavirus OC43 (ATCC, #VR-1558) were added at various concentrations (for binning: [RBD2] = 10 nM and [spike] = 20 nM; for LoD/LoQ determination see concentration ranges in Supplementary Figure 7). After incubation and washing, the detecting IgG-HRP conjugates were added at 50 nM for both binning and LoD/LoQ assays, followed by incubation and washing. About 90 μL HRP substrate TMB was added until a blue color started to develop, followed by acidification (i.e. addition of 90 μL 1 M H_2_SO_4_ to interrupt the enzymatic reaction) and reading of Abs_450_. For binning, each Abs_450_ value was plotted in MS Excel (3D bar graph) as a bar at the intersection of the corresponding capturing (x-axis)/detecting (z-axis) antibody. For LoQ/LoD calculations, averages of three experiments and corresponding standard deviations (error bars) were calculated in MS Excel, plotted *vs* the antigen concentration and fitted to linear equations. Averages of three Abs_450_ measurements obtained for negative antigens myoglobin and rhino coronavirus (at max value of the antigen concentration range) plus 3 or 8 standard deviations were used as y values in the linear equations defining the signal *vs* antigens concentration obtained for each antibody combination, to find the antigen concentrations (x values) corresponding to the LoDs and LoQs, respectively.

#### Epitope binning by SPR

Epitope binning was performed with a classical sandwich assay format on a Carterra LSA SPR instrument equipped with a HC200M sensor chip (200 nm linear polycarboxylate surface) at 25°C and in a HBSTE-BSA running buffer (10 mM HEPES pH 7.4, 150 mM NaCl, 3 mM EDTA, 0.05% Tween-20, supplemented with 0.5 mg/mL BSA). Two microfluidic modules, a 96-channel print-head (96PH) and a single flow cell (SFC), were used to deliver samples onto the sensor chip. Surface preparation was performed with 25 mM MES pH 5.5 with 0.05% Tween-20 as a running buffer. The chip was activated with a freshly prepared solution of 130 mM 1-ethyl-3-(3-dimethylaminopropyl) carbodiimide (EDC) + 33 mM N-hydroxysulfosuccinimide (Sulfo-NHS) in 0.1 M MES pH 5.5 using the SFC. Antibodies were immobilized using the 96PH for 10 minutes at 15 µg/mL diluted into 10 mM sodium acetate (pH 4.25). Unreactive esters were quenched with a 7-minute injection of 1 M ethanolamine-HCl (pH 8.5) using the SFC. The binning analysis was performed over this array with the HBSTE-BSA buffer as the running buffer and sample diluent. The RBD antigen was injected in each cycle for 4 minutes at 100 nM (3.6 µg/mL) and followed immediately by a 4-minute injection of the analyte antibody at 30 µg/mL (200 nM for IgG constructs). The surface was regenerated each cycle with two 30 second pulses of Pierce IgG Elution Buffer (pH 2.8) with 1 M NaCl. Data was processed and analyzed with Epitope Tool (Carterra Inc.). Briefly, data was referenced using unprinted locations on the array and each binding cycle was normalized to the RBD capture level. The binding level of the analyte antibody just after the end of the injection was compared to that of a buffer alone injection. Signals that increased relative to the buffer controls are described as sandwiching and represent non-blocking behavior. Competition results were visualized as a heat map in which red, yellow, and green cells represent blocked, intermediate, and not blocked analyte/ligand pairs, respectively. Clones having identical patterns of competition are classified as being within the same bin cluster. The antibody NN54 was purchased from Creative Diagnostics (CABT-CS064).

#### Kinetics by SPR

Binding kinetics was also performed on the same array surface as described for epitope binning. Kinetics were analyzed at 25°C in HBSTE-BSA running buffer. The RBD antigen was injected with 5 minutes of association at six concentrations in a three-fold dilution series starting at 500 nM to 1.49 nM with a 10-minute dissociation. Data were analyzed using the Carterra Kinetics tool. Data were processed by double referencing with the subtraction of an interspot reference and buffer blank cycle, then fit to a 1:1 Langmuir model to determine the *k*_a_, *k*_d_, *K*_D_, and R_max_.

#### Antibody community assignment

As the clone CR3022 and ACE2 Fc was included in both the epitope binning assay described above and the results described by Hastie et al. [61], the following putative assignments were given. It appears the E08 clone is likely a bin 7b clone, as it binned with CR3022. Clone H05 is most likely a community 7a clone as it is completive with CR3022 and ACE2-Fc but not most of the other clones. Clone H01 was competitive with the CR3022 bin, H05, and ACE2-Fc which is a profile which would be consistent with being a member of community 6. The community of the other clones gets harder to assess without specific probes but based on the ACE-2 competition CR3022 sandwiching we can at least narrow down the possible bins for each clone, but not fully assign them. S01 is competitive with ACE2-Fc, H01, and E01 and E08, but not H05, E08, and CR3022 which is fully consistent with a clone profile for community 2a. The clone E01 is likely in another community 2 group, but not 2a as it does not compete with the putative bin 6 clone H01 but does compete with ACE2-Fc and S01. Clones B04, G07, and F07 are all not competitive with ACE2-Fc or the CR3022 which most likely puts them into one of the Bin 5 communities.

#### Antibody clonotype analyses

Three local blast databases were constructed with all the human V, D, J sequences downloaded from IMGT/GEND-DB [85] (release 202,209–1), respectively. IgBLAST [86] (version 1.18) was used to identify the top three germline gene lineage candidates using the databases for each scFv. The final gene lineage assignment was performed by combining gene names from the top three candidates.

### Spin Dx based assays

#### SpinDx Immunoassay reagent preparation

Anti-RBD2 IgGs E01, S01, F07, G07 were used to generate reagents for the SpinDx sandwich immunoassays. To generate the captured beads, 1 µm carboxylic acid-functionalized silica microspheres (Bangs Laboratories, #SC04N), were activated with N-ethyl-N0 -(3-dimethylaminopropyl) carbodiimide and n-hydroxy succinimide (0.5 mmoles of each) in 0.5 mL of 500 mM MES at pH 6.0 for 20 minutes at room temperature. Microspheres were washed twice with 100 mM phosphate buffered saline (PBS; 138 mM NaCl, 2.7 mM KCl, 10 mM Na_2_HPO_4_, pH 7.4). Each capture antibody was added to a final concentration of 1 µg of antibody per 1 mg of microspheres per reaction in 0.5 mL PBS raised to pH 8.15 with 1 M NaHCO_3_ and allowed to conjugate overnight at room temperature. The particles were then washed twice with PBS and blocked with Superblock (ThermoFisher, #37515) for 60 min at room temperature. After blocking, the particles were washed in wash buffer (0.1% (w/v) Tween-20 in PBS) and resuspended in assay buffer (1% (w/v) BSA, 0.1% (w/v) Tween-20 in PBS) to a concentration of 12% beads. To generate detection antibodies, each antibody was labeled with Alexa Fluor 647 (Life Technologies, Carlsbad, CA, USA). 10 µg of antibody was brought up to a volume of 40 µL with PBS. For each antibody, one vial of activated Alexa Fluor 647 was reconstituted in 5 µL of dimethyl sulfoxide and then added to the diluted antibody. The mixture was brought up to pH 8.15 with 5 µL of 1 M sodium bicarbonate and allowed to react at room temperature for 15 min in the dark. After the reaction was complete, unreacted dye was separated from labeled antibody using desalting spin columns with 7 kDa molecular weight cut-off (ThermoFisher, #89882). Concentrations, and dye-to-antibody ratios were determined spectrophotometrically by UV absorbance.

#### SpinDx immunoassay protocol

Serial dilutions of the antigen, SARS-CoV-2 trimeric spike protein (Acro Biosystems, #SPN-C52H8), were prepared to five times of the final concentrations of 0 ng/mL, 1 ng/mL, 10 ng/mL 100 ng/mL and 1000 ng/mL in assay buffer. To start the sandwich immunoassay, 1 µL of antigen (various concentrations) and 1 µL of detection antibody (2 nM) were added to 3 µL of a 12% (w/v) suspension of captured microspheres. Capture beads, antigen, and detector antibody were incubated at room temperature for 20 m to allow bead complexes to form. Each antigen concentration was tested with the following capturing/detecting antibody combinations: S01/G07, S01/F07, G07/S01, and G07/F07, which were selected following a preliminary screening assay using all the antibody combinations (not reported) determining which antibody combinations produced the highest dynamic range. After incubation, the suspensions were mixed to resuspend beads that had settled and the entire volume (5 µL) of each suspension was added to the channel of a SpinDx microfluidic disc preloaded with 3 µL of density medium (Percoll, 0.1% Tween 20). Discs were then placed into the SpinDx device, secured with a thumbscrew, and the analysis protocol was started via the computer-controlled graphical user interface. The device automatically spins the disc at 5000 RPM, indexes the channels, analyses each channel via laser-induced fluorescence, and reports relative fluorescence values to the connected computer. The fluorescence values are then exported to Prism (GraphPad Software, San Diego, CA, USA) for data analysis and reduction. Replicate data points were averaged, standard deviations were graphed as the error bars, and the data were fit to a four-parameter sigmoidal curve. LoD and LoQ were interpolated from the curve fit using the IUPAC definition of three and eight standard deviations above the noise, respectively [87].

### Fluorescent microscopy with HEK-ACE2 cell and RBD2-sfGFP

Human ACE2 293 cell line (Takara Bio USA, #631289) was cultured in DMEM media supplemented with 10% fetal bovine serum. To activate the expression of the ACE2 receptors from a transgene integrated into the cellular genome, we added 1 µg/mL puromycin in the cell growth media. For the microscopy experiments, cells were plated onto 8-chambered borosilicate glass slides (Nunc LabTek, #155411) coated with poly-L-Lysine (EMD Millipore/Sigma). The cells were fixed using 4% paraformaldehyde (PFA) in 1xPBS for 15 minutes, washed twice with 1xPBS and blocked using 2% BSA in 1xPBS for at least 30 minutes (up to 24 h). We performed two types of binding assays using ACE2 293 cells, in vitro RBD2 sfGFP reagent, and unlabeled or PE-labeled IgGs. In the first assay, we investigated antibody binding to the RBD2 in solution and the effect of the antibody binding on ACE2-RBD2 interaction. We incubated RBD2-sfGFP (2 μg/mL, 36 μM) with unlabeled IgG (200 μg/mL, 1.33 μM) for 1 hr prior to addition of the antibody to the ACE2 293 cells-coated chamber slides. Unbound proteins were washed, and fluorescent microscopy images were obtained with Zeiss Axio Observer Z.1.

In the second assay, antibody ability to recognize ACE2-bound RBD2 sfGFP was evaluated. Here 100 μL of 2 μg/mL (36 nM) of RBD2-sfGFP was added to each of the wells containing the HEK ACE2 cells and allowed to bind for 1 hr at RT. Excess protein was washed 3X PBST and 3X PBS. PE conjugated IgGs (1 μg/mL) was added to the wells and incubated for another hour, followed by washing steps as described for the primary antibody. HEK-ACE2 cells (in the absence of RBD2 sfGFP) were used as specificity controls. Fluorescent images were analyzed with the ZenPro software.

### Viral neutralization assay

The neutralizing activity of the eight IgGs was conducted using plaque assays with active virus as previously described [88]. Briefly, SARS-CoV-2 (isolate USA-WA1/2020, obtained from BEI Resources) was diluted to 50–100 PFU/200 µL in viral growth medium (VGM, minimal essential medium with 2.5% heat inactivated fetal calf serum) and incubated 1:1 with IgGs at concentrations ranging from 700 to 0 nM (preliminary assay) or 172 to 0.172 nM (assay using best antibodies/antibody combinations) in VGM and incubated at 37°C for 1–1.5 hours. Virus-IgG mixtures were added to 80% confluent Vero-E6 cells (ADCC, #CRL-1586) and incubated for 2 hours at 37°C. Supernatants were then removed, and cells were washed once with PBS and then overlaid with 1 mL virus overlay medium (equal volumes of 2% agarose/2× minimal essential medium with 5% fetal calf serum and 2× penicillin/streptomycin). Cells were then incubated at 37°C for 2 days and fixed at 4°C overnight with 4% formaldehyde. Fixative and viral overlay was removed, and cells were stained with 0.5% crystal violet for 1–2 minutes, washed, and dried. Percentages of plaque reduction caused by antibody-treated versus untreated virus (N = neutralization) were plotted against antibody concentrations and data points were fitted to the equation N = N_max_*[IgG]/(NT50+[IgG]) to determine the N_max_ and the NT50 (half neutralizing titer) values indicated in Figure 5B.

## Supplementary Material

Supplemental Material

## Significance statement

Countermeasures against COVID-19 or other possible future epidemic/pandemic include the development of pathogen-neutralizing/detecting antibody sets (cocktails). Antibody cocktails, more than single antibodies, are likely to retain their function in spite of the pathogen’s natural tendency to mutate. Within 40 days from antigen acquisition, we have isolated a large suite of anti COVID-19 antibodies with therapeutic and diagnostic potential, from a collection of human antibodies (antibody library). This library, obtained from healthy donors long before the COVID-19 outbreak, was readily available to us and yielded the sought-after antibodies using a low-cost antibody selection strategy. The selection strategy and the features of the selected antibodies are the topics of this publication.

## Institutions’ statement and disclaimers

Los Alamos National Laboratory is operated by Triad National Security for U.S. Department of Energy’s National Nuclear Security Administration (DOE-NNSA) under contract 89233218CNA000001.

Sandia National Laboratories are multi-mission laboratories managed and operated by National Technology & Engineering Solutions of Sandia, LLC, a wholly owned subsidiary of Honeywell International Inc. for the U.S. DOE-NNSA under contract DE-NA0003525.

## References

[1] Wu F, Zhao S, Yu B, *et al*. A new coronavirus associated with human respiratory disease in China. Nature. 2020;579:265–269. 32015508 10.1038/s41586-020-2008-3 PMC7094943

[2] Zhou P, Yang X-L, Wang X-G, *et al*. A pneumonia outbreak associated with a new coronavirus of probable bat origin. nature. 2020;579:270–273. 32015507 10.1038/s41586-020-2012-7 PMC7095418

[3] Jiang S, Shi Z, Shu Y, *et al*. A distinct name is needed for the new coronavirus. Lancet. 2020;395:949. DOI:10.1016/S0140-6736(20)30419-0 PMC7124603 32087125

[4] Lu R, Zhao X, Li J, *et al*. Genomic characterisation and epidemiology of 2019 novel coronavirus: implications for virus origins and receptor binding. Lancet. 2020;395:565–574. 32007145 10.1016/S0140-6736(20)30251-8 PMC7159086

[5] Wrapp D, Wang N, Corbett KS, *et al*. Cryo-EM structure of the 2019-nCoV spike in the prefusion conformation. Science. 2020;367:1260–1263. 32075877 10.1126/science.abb2507 PMC7164637

[6] Lan J, Ge J, Yu J, *et al*. Structure of the SARS-CoV-2 spike receptor-binding domain bound to the ACE2 receptor. Nature. 2020;581:215–220. 32225176 10.1038/s41586-020-2180-5

[7] Amin M, Sorour MK, Kasry A. Comparing the binding interactions in the receptor binding domains of SARS-CoV-2 and SARS-CoV. J Phys Chem Lett. 2020;11:4897–4900. 32478523 10.1021/acs.jpclett.0c01064

[8] Chakraborty S. Evolutionary and structural analysis elucidates mutations on SARS-CoV2 spike protein with altered human ACE2 binding affinity. Biochem Biophys Res Commun. 2021;538:97–103. 33602511 10.1016/j.bbrc.2021.01.035 PMC7883683

[9] Grifoni A, Sidney J, Zhang Y, *et al*. A sequence homology and bioinformatic approach can predict candidate targets for immune responses to SARS-CoV-2. *Cell host & microbe*. Cell Host & Microbe. 2020;27:671–680. e672. 32183941 10.1016/j.chom.2020.03.002 PMC7142693

[10] Rattanapisit K, Shanmugaraj B, Manopwisedjaroen S, *et al*. Rapid production of SARS-CoV-2 receptor binding domain (RBD) and spike specific monoclonal antibody CR3022 in Nicotiana benthamiana. Sci Rep. 2020;10:1–11. 33077899 10.1038/s41598-020-74904-1 PMC7573609

[11] Mehalko J, Drew M, Snead K, *et al*. Improved production of SARS-CoV-2 spike receptor-binding domain (RBD) for serology assays. Protein Expr Purif. 2021;179:105802. 33248226 10.1016/j.pep.2020.105802 PMC7687410

[12] Arbeitman CR, *et al*. Structural and functional comparison of SARS-CoV-2-spike receptor binding domain produced in Pichia pastoris and mammalian cells. Sci Rep. 2020;10:21779–21796. 33311634 10.1038/s41598-020-78711-6 PMC7732851

[13] Spellberg B, Guidos R, Gilbert D, *et al*. The epidemic of antibiotic-resistant infections: a call to action for the medical community from the infectious diseases society of America. Clinl Infect Dis. 2008;46:155–164. 10.1086/524891 18171244

[14] McConnell MJ. Where are we with monoclonal antibodies for multidrug-resistant infections? Drug Discovery Today. 2019;24:1132–1138. 30853568 10.1016/j.drudis.2019.03.002

[15] Berghman L, Abi-Ghanem D, Waghela S, et al. Antibodies: an alternative for antibiotics? Poult Sci. 2005;84:660–666. 15844826 10.1093/ps/84.4.660 PMC7107177

[16] Dimitrov DS, Marks JD Therapeutic Antibodies: Current State and Future Trends – Is a Paradigm Change Coming Soon? . In: Dimitrov A. (eds) Therapeutic Antibodies. Methods in Molecular Biology™ (Methods and Protocols). Vol. 525. Humana Press; 2009. p. 1–27. 10.1007/978-1-59745-554-1_. PMC3402212 19252861

[17] Mahmuda A, Bande F, Abdulhaleem N, *et al*. Monoclonal antibodies in immunodiagnostic assays: a review of recent applications. Sokoto J Vet Sci. 2017;15:1–10.

[18] Gubala V, Klein R, Templeton DM, et al. Immunodiagnostics and immunosensor design (IUPAC Technical Report). Pure Appl Chem. 2014;86:1539–1571.

[19] Gervais L, Delamarche E. Toward one-step point-of-care immunodiagnostics using capillary-driven microfluidics and PDMS substrates. Lab Chip. 2009;9:3330–3337. 19904397 10.1039/b906523g

[20] Gervais L, De Rooij N, Delamarche E. Microfluidic chips for point‐of‐care immunodiagnostics. Adv Mater. 2011;23:H151–H176. 21567479 10.1002/adma.201100464

[21] Hansen J, Baum A, Pascal KE, *et al*. Studies in humanized mice and convalescent humans yield a SARS-CoV-2 antibody cocktail. Science. 2020;369:1010–1014. 32540901 10.1126/science.abd0827 PMC7299284

[22] Shi R, Shan C, Duan X, *et al*. A human neutralizing antibody targets the receptor-binding site of SARS-CoV-2. Nature. 2020;584:120–124. 32454512 10.1038/s41586-020-2381-y

[23] Gottlieb RL, Nirula A, Chen P, *et al*. Effect of bamlanivimab as monotherapy or in combination with etesevimab on viral load in patients with mild to moderate COVID-19: a randomized clinical trial. Jama. 2021;325:632–644. 33475701 10.1001/jama.2021.0202 PMC7821080

[24] Baum A, Fulton BO, Wloga E, *et al*. Antibody cocktail to SARS-CoV-2 spike protein prevents rapid mutational escape seen with individual antibodies. Science. 2020;369:1014–1018. 32540904 10.1126/science.abd0831 PMC7299283

[25] Wang P, *et al*. Antibody resistance of SARS-CoV-2 variants B. 1.351 and B. Nature. 2021;1(1):1–6. 10.1038/s41586-021-03398-2 33684923

[26] (FDA), U. f. a. d. a. *In Vitro Diagnostics EUAs - Antigen Diagnostic Tests for SARS-CoV-2*, https://www.fda.gov/medical-devices/coronavirus-disease-2019-covid-19-emergency-use-authorizations-medical-devices/in-vitro-diagnostics-euas-antigen-diagnostic-tests-sars-cov-2 (2020).

[27] Datta M, Singh DD, Naqvi AR. Molecular Diagnostic Tools for the Detection of SARS-CoV-2. Int Rev Immunol. 2021;40:1–21. 33439059 10.1080/08830185.2020.1871477

[28] Valldorf B, Hinz SC, Russo G, *et al*. Antibody display technologies: selecting the cream of the crop. Biol Chem. 2021;403:455–477. 33759431 10.1515/hsz-2020-0377

[29] Bradbury A, Plückthun A. Reproducibility: standardize antibodies used in research. Nat News. 2015;518:27. 10.1038/518027a 25652980

[30] Chi X, Liu X, Wang C, *et al*. Humanized single domain antibodies neutralize SARS-CoV-2 by targeting the spike receptor binding domain. Nat Commun. 2020;11:1–7. 32913273 10.1038/s41467-020-18387-8 PMC7483421

[31] Wu Y, Li C, Xia S, *et al*. Identification of human single-domain antibodies against SARS-CoV-2. *Cell host & microbe*. Cell Host & Microbe. 2020;27:891–898. e895. 32413276 10.1016/j.chom.2020.04.023 PMC7224157

[32] Stefan MA, *et al*. Mabs. Milton Park, in Oxfordshire: Taylor & Francis. 2021. ID:1958663.

[33] Zeng X, Li L, Lin J, *et al*. Isolation of a human monoclonal antibody specific for the receptor binding domain of SARS-CoV-2 using a competitive phage biopanning strategy. Antibody Ther. 2020;3:95–100. DOI:10.1093/abt/tbaa008 PMC7197610 33912790

[34] Bertoglio F, Meier D, Langreder N, *et al*. SARS-CoV-2 neutralizing human recombinant antibodies selected from pre-pandemic healthy donors binding at RBD-ACE2 interface. Nat Commun. 2021;12:1–15. 33707427 10.1038/s41467-021-21609-2 PMC7952403

[35] Sblattero D, Bradbury A. Exploiting recombination in single bacteria to make large phage antibody libraries. Nat Biotechnol. 2000;18:75–80. 10625396 10.1038/71958

[36] Phipps ML, Lillo AM, Shou Y, *et al*. Beyond helper phage: using” Helper Cells” to select peptide affinity ligands. PloS one. 2016;11:e0160940. 27626637 10.1371/journal.pone.0160940 PMC5023105

[37] Smith GP, Petrenko VA. Phage display. Chem Rev. 1997;97:391–410. 11848876 10.1021/cr960065d

[38] Lillo AM, McKenzie KM, Janda KD. Cell Biology. Amsterdam, Netherlands: Elsevier; 2006. p. 491–496.

[39] Boder ET, Wittrup KD. [25] Yeast surface display for directed evolution of protein expression, affinity, and stability. Methods Enzymol. 2000;328:430–444. 11075358 10.1016/s0076-6879(00)28410-3

[40] Velappan N, Martinez JS, Valero R, *et al*. Selection and characterization of scFv antibodies against the sin nombre hantavirus nucleocapsid protein. J Immunol Methods. 2007;321:60–69. 17336997 10.1016/j.jim.2007.01.011

[41] Velappan N, *et al*. in *Mabs*. 1843754. Milton Park, in Oxfordshire: Taylor & Francis. 2020.

[42] Lillo AM, Ayriss JE, Shou Y, et al. Development of phage-based single chain Fv antibody reagents for detection of Yersinia pestis. PloS one. 2011;6:e27756. 22174746 10.1371/journal.pone.0027756 PMC3234238

[43] Lillo AM, Velappan N, Kelliher JM, *et al*. Development of anti-yersinia pestis human antibodies with features required for diagnostic and therapeutic applications. Immunotargets Ther. 2020;9:299. 33294421 10.2147/ITT.S267077 PMC7716875

[44] Close DW, Ferrara F, Dichosa AE, *et al*. Using phage display selected antibodies to dissect microbiomes for complete de novo genome sequencing of low abundance microbes. BMC Microbiol. 2013;13:1–14. 24279426 10.1186/1471-2180-13-270 PMC3907030

[45] Kim Y, Lillo AM, Steiniger SCJ, *et al*. Targeting heat shock proteins on cancer cells: selection, characterization, and cell-penetrating properties of a peptidic GRP78 ligand. Biochemistry. 2006;45:9434–9444. 16878978 10.1021/bi060264j

[46] Martinez JS, *et al*. (Google Patents), 2017.

[47] Felding-Habermann B, *et al*. Combinatorial antibody libraries from cancer patients yield ligand-mimetic arg-gly-asp-containing immunoglobulins that inhibit breast cancer metastasis. *Proceedings of the National Academy of Sciences* 101, 17210–17215 (2004). 10.1073/pnas.0407869101 PMC534417 15563590

[48] Lillo AM, Sun C, Gao C, *et al*. A human single-chain antibody specific for integrin α3β1 capable of cell internalization and delivery of antitumor agents. Chem Biol. 2004;11:897–906. 15271348 10.1016/j.chembiol.2004.04.018

[49] Kehoe JW, Velappan N, Walbolt M, *et al*. Using phage display to select antibodies recognizing post-translational modifications independently of sequence context. Mol Cell Proteomics. 2006;5:2350–2363. 16971384 10.1074/mcp.M600314-MCP200

[50] Velappan N, *et al*. in *MAbs*. 1206-1218. Taylor & Francis).

[51] Shusta EV, Kieke MC, Parke E, et al. Yeast polypeptide fusion surface display levels predict thermal stability and soluble secretion efficiency. J Mol Biol. 1999;292:949–956. 10512694 10.1006/jmbi.1999.3130

[52] Li B, *et al*. in *MAbs*. 437-445. Milton Park, in Oxfordshire: Taylor & Francis. 2013.

[53] Bell BN, Powell AE, Rodriguez C, et al. Neutralizing antibodies targeting the SARS‐CoV‐2 receptor binding domain isolated from a naïve human antibody library. Protein Sci. 2021;30:716–727. 33586288 10.1002/pro.4044 PMC7980507

[54] Ferrara F, Chillón I, Genna V, *et al*. A pandemic-enabled comparison of discovery platforms demonstrates a naïve antibody library can match the best immune-sourced antibodies. Nat Commun. 2022;13:1–12. 35075126 10.1038/s41467-021-27799-z PMC8786865

[55] Beckett D, Kovaleva E, Schatz PJ. A minimal peptide substrate in biotin holoenzyme synthetase-catalyzed biotinylation. Protein Sci. 1999;8:921–929. 10211839 10.1110/ps.8.4.921 PMC2144313

[56] Xia S, Zhu Y, Liu M, *et al*. Fusion mechanism of 2019-nCoV and fusion inhibitors targeting HR1 domain in spike protein. Cell Mol Immunol. 2020;17:765–767. 32047258 10.1038/s41423-020-0374-2 PMC7075278

[57] Xu JL, Davis MM. Diversity in the CDR3 region of VH is sufficient for most antibody specificities. Immunity. 2000;13:37–45. 10933393 10.1016/s1074-7613(00)00006-6

[58] Yuan M, Wu NC, Zhu X, *et al*. A highly conserved cryptic epitope in the receptor binding domains of SARS-CoV-2 and SARS-CoV. Science. 2020;368:630–633. 32245784 10.1126/science.abb7269 PMC7164391

[59] Ter Meulen J, *et al*. Human monoclonal antibody combination against SARS coronavirus: synergy and coverage of escape mutants. PLoS Med. 2006;3:e237. 16796401 10.1371/journal.pmed.0030237 PMC1483912

[60] Diagnostics C. Mouse anti-SARS-CoV-2 spike neutralizing monoclonal antibody, clone NN54. 2020;CABT–CS064.

[61] Hastie KM, Li H, Bedinger D, *et al*. Defining variant-resistant epitopes targeted by SARS-CoV-2 antibodies: a global consortium study. Science. 2021;374:472–478. 34554826 10.1126/science.abh2315 PMC9302186

[62] Lorenzo-Redondo R, Nam HH, Roberts SC, *et al*. A unique clade of SARS-CoV-2 viruses is associated with lower viral loads in patient upper airways. MedRxiv. 2020; DOI:10.1101/2020.05.19.20107144 PMC7655495 33186810

[63] Korber B, Fischer WM, Gnanakaran S, *et al*. Tracking changes in SARS-CoV-2 Spike: evidence that D614G increases infectivity of the COVID-19 virus. Cell. 2020;182:812–827. e819. 32697968 10.1016/j.cell.2020.06.043 PMC7332439

[64] Walsh DI III, Sommer GJ, Schaff UY, *et al*. A centrifugal fluidic immunoassay for ocular diagnostics with an enzymatically hydrolyzed fluorogenic substrate. Lab Chip. 2014;14:2673–2680. 24806296 10.1039/c4lc00279b

[65] Koh C-Y, Schaff UY, Piccini ME, *et al*. Centrifugal microfluidic platform for ultrasensitive detection of botulinum toxin. Anal Chem. 2015;87:922–928. 25521812 10.1021/ac504054u PMC4303339

[66] Litvinov J, Moen ST, Koh C-Y, et al. Centrifugal sedimentation immunoassays for multiplexed detection of enteric bacteria in ground water. Biomicrofluidics. 2016;10:014103. 26858815 10.1063/1.4939099 PMC4714988

[67] Phaneuf CR, Mangadu B, Piccini ME, et al. Rapid, portable, multiplexed detection of bacterial pathogens directly from clinical sample matrices. Biosensors (Basel). 2016;6:49. 10.3390/bios6040049 PMC5192369 27669320

[68] Phaneuf CR, Mangadu B, Tran HM, *et al*. Integrated LAMP and immunoassay platform for diarrheal disease detection. Biosens Bioelectron. 2018;120:93–101. 30172236 10.1016/j.bios.2018.08.005 PMC6145809

[69] Yang J, Petitjean SJL, Koehler M, *et al*. Molecular interaction and inhibition of SARS-CoV-2 binding to the ACE2 receptor. Nat Commun. 2020;11:1–10. 32917884 10.1038/s41467-020-18319-6 PMC7486399

[70] Erasmus MF, D’Angelo S, Ferrara F, *et al*. A single donor is sufficient to produce a highly functional in vitro antibody library. Commun Biol. 2021;4:1–16. 33742103 10.1038/s42003-021-01881-0 PMC7979914

[71] Moon SA, Ki MK, Lee S, *et al*. Antibodies against non-immunizing antigens derived from a large immune scFv library. Mol Cells. 2011;31:509–513. 21499952 10.1007/s10059-011-2268-8 PMC3887623

[72] Kieke MC, *et al*. Selection of functional T cell receptor mutants from a yeast surface-display library. *Proceedings of the National Academy of Sciences* 96, 5651–5656 (1999). 10.1073/pnas.96.10.5651 PMC21915 10318939

[73] Bailly M, Mieczkowski C, Juan V, *et al*. Predicting antibody developability profiles through early stage discovery screening. mAbs. 2020;12:1743053. 32249670 10.1080/19420862.2020.1743053 PMC7153844

[74] Velappan N, Mahajan A, Naranjo L, *et al*. Selection and characterization of FcεRI phospho-ITAM specific antibodies. MAbs. 2019;11:1206–1218. 31311408 10.1080/19420862.2019.1632113 PMC6748597

[75] Di Marzo Veronese F, Reitz MS, Gupta G, *et al*. Loss of a neutralizing epitope by a spontaneous point mutation in the V3 loop of HIV-1 isolated from an infected laboratory worker. J Biol Chem. 1993;268:25894–25901. 7503990

[76] Zhang S, Vogt M, Oliphant T, *et al*. Development of resistance to passive therapy with a potently neutralizing humanized monoclonal antibody against West Nile virus. J Infect Dis. 2009;200:202–205. 19527169 10.1086/599794 PMC2752978

[77] Tan TJC, Yuan M, Kuzelka K, *et al*. Sequence signatures of two public antibody clonotypes that bind SARS-CoV-2 receptor binding domain. Nat Commun. 2021;12:3815. 34155209 10.1038/s41467-021-24123-7 PMC8217500

[78] Kozel TR, Burnham-Marusich AR, Kraft CS. Point-of-care testing for infectious diseases: past, present, and future. J Clin Microbiol. 2017;55:2313–2320. 28539345 10.1128/JCM.00476-17 PMC5527409

[79] Pan Y, Zhang D, Yang P, et al. Viral load of SARS-CoV-2 in clinical samples. Lancet Infect Dis. 2020;20:411–412. 32105638 10.1016/S1473-3099(20)30113-4 PMC7128099

[80] Chen H, Liu K, Li Z, et al. Point of care testing for infectious diseases. Clin Chim Acta. 2019;493:138–147. 30853460 10.1016/j.cca.2019.03.008 PMC6462423

[81] Sachdeva S, Davis RW, Saha AK. Microfluidic point-of-care testing: commercial landscape and future directions. Front Bioeng Biotechnol. 2021;8:1537. 10.3389/fbioe.2020.602659 PMC7843572 33520958

[82] Hirotsu Y, Maejima M, Shibusawa M, *et al*. Comparison of automated SARS-CoV-2 antigen test for COVID-19 infection with quantitative RT-PCR using 313 nasopharyngeal swabs, including from seven serially followed patients. Inter J Infect Dis. 2020;99:397–402. 10.1016/j.ijid.2020.08.029 PMC7422837 32800855

[83] Wentz AE, Shusta EV. A novel high-throughput screen reveals yeast genes that increase secretion of heterologous proteins. Appl Environ Microbiol. 2007;73:1189–1198. 17189442 10.1128/AEM.02427-06 PMC1828678

[84] Velappan N, Clements J, Kiss C, *et al*. Fluorescence linked immunosorbant assays using microtiter plates. J Immunol Methods. 2008;336:135–141. 18514691 10.1016/j.jim.2008.04.007

[85] Lefranc M-P, Lefranc G. The immunoglobulin factsbook. Amsterdam. Netherlands: Elsevier; 2001.

[86] Ye J, Ma N, Madden TL, et al. IgBLAST: an immunoglobulin variable domain sequence analysis tool. Nucleic Acids Res. 2013;41:W34–W40. 23671333 10.1093/nar/gkt382 PMC3692102

[87] Allegrini F, Olivieri AC. IUPAC-consistent approach to the limit of detection in partial least-squares calibration. Anal Chem. 2014;86:7858–7866. 25008998 10.1021/ac501786u

[88] Bradfute SB, Hurwitz I, Yingling AV, *et al*. Severe acute respiratory syndrome coronavirus 2 neutralizing antibody titers in convalescent plasma and recipients in new mexico: an open treatment study in patients with coronavirus disease 2019. J Infect Dis. 2020;222:1620–1628. 32779705 10.1093/infdis/jiaa505 PMC7454720

