# Supplementary material for: Healthy humans can be a source of antibodies countering COVID-19
Source: Bioengineered. 2022 May 21;13(5):12598–624. doi: 10.1080/21655979.2022.2076390 (PMC9275966; doi:10.1080/21655979.2022.2076390)
Supplement: Supplemental Material [file KBIE_A_2076390_SM0860.zip › Supplementary figures.docx]

**SUPPLEMENTARY FIGURES**

**Supplementary Figure 1: Sequences of unique antibodies derived from all selections.** Single chain antibodies (scFvs) B04 through H05 were obtained by competitive selections using chemically biotinylated RBD2 as a target. scFvs R04 through S01 were obtained by non-competitive selections using avitagged-biotinylated RBD2 as a target. The three complementarity-determining regions (CDR) in each scFv’s variable light region (VL, CDRL1, 2, and 3) are indicated in red; the linker between the VL and the scFv’s variable heavy region (VH) is indicated in gray; the CDRH1, 2, and 3 are indicated in green. Antibody clonotype information and putative COVID-19 antibody community number are also indicated.

**Supplementary Figure 2. Analysis of antibody complementarity determining regions analysis.** Alignment of our antibody sequences with COVID-19 antibody sequences published on the Antibody Society website, revealed shared single CDRs or CDR pairs. None of these CDRs are CDRH3 (palest yellow bar). None of our antibodies shares the entire CDR set. Three of our antibodies share CDRL3 (palest blue bars). Antibody E01 does not share any of its CDRs.

**Supplementary Figure 3**. **Kinetic data.** **A**) Graphs from flow cytometry-based kinetic measurements of yeast-displayed scFvs. The upper left graph contains D07 kinetics at lower yeast density (1/10 of all the other experiments including the second D07 experiment). The fact that D07 K_D_ did not change significantly at the two yeast densities suggests that there is no antigen depletion in these experiments and therefore the K_D_ measurement should be accurate. The lower right graph contains kinetics of R01 binding to both RBD1 and RBD2. Negative control antibody Z3 is also included in this graph. **B**) Representative sensorgrams from surface plasmon resonance (SPR)-based kinetic measurements reported in Table 1 and Table S1.

**Supplementary Figure 4. Specificity of IgG interactions.** IgGs were immobilized on plastic and tested for recognition of either (**A**) chemically biotinylated SARS-CoV-2 RBD (RBD2, blue bars) and SARS-CoV-1 RBD (RBD1, orange bars) and avitagged-biotinylated SARS-CoV-2 spike subunit 1 (S1, grey bars), subsequently stained with streptavidin-Alexa 633, or (**B**) RBD2-sfGFP (RBD2, blue bars)) and RBD1-sfGFP (RBD1 orange bars) chimeras. Chemically biotinylated lysozyme (Lys, yellow bars) and anti-*Y. pestis* F1 antigen antibody AM2 (AM2) were used as negative controls antigen and antibody respectively. Average or three measurements plus standard deviations (error bars) are reported.

**Supplementary Figure 5**. Affinity variations depending on antibody format. Antibody affinities for SARS-CoV-2 RBD2 are reported as dissociation constants (K_D_, lower K_D_ → higher affinity) for the following formats: 1) scFv yeast-expressed (blue bars); 2) scFv-Fc (minibody, orange bars); 3) immunoglobulin G (IgG, grey bars). For affinity measurements repeated in triplicate, K_D_ averages and corresponding standard deviations (error bars) are reported.

**Supplementary Figure 6. Epitope binning by sandwich ELISA.** Identification of antibody pairs (capturing and detecting IgG) capable of binding to distinct regions (epitopes) of SARS-CoV-2 RBD2, **A**) and SARS-CoV-2 whole spike protein (**B**). Plastic bound capturing IgG (x axis) immobilize the antigen, and HRP-conjugated detecting IgG (z axis) reports the captured antigen. Anti-influenza M2 (αM2) antibody was used as a negative control IgG. Signals above αM2-mediated antigen detection (noise) reveal antibody pairs non competitively binding to the antigen.

**Supplementary Figure 7.** Detection of trimeric spike or whole SARS-CoV-2 virus by sandwich immunoassays. Detection of wild type spike (A) and D614G mutant spike (B) by sandwich ELISA. Detection of wild type spike by SpinDx (C). Detection of heat inactivated whole SARS-CoV-2 virus by sandwich ELISA (D). The antibody pairs used for detection are indicated as (antigen-capturing IgG)/(antigen-detecting IgG) (e.g. S01/G07). Only antibody pairs E01/F07 and E01/G07 could detect whole virus at the concentration that we were able to test with the limited amount of virus available to us. RFU = relative fluorescence unit; TCID50 = half tissue culture infection dose
