# Supplementary material for: Healthy humans can be a source of antibodies countering COVID-19
Source: Bioengineered. 2022 May 21;13(5):12598–624. doi: 10.1080/21655979.2022.2076390 (PMC9275966; doi:10.1080/21655979.2022.2076390)
Supplement: Supplemental Material [file KBIE_A_2076390_SM0860.zip › SUPPLEMENTARY TABLES (3).docx]

**TABLES**

**Table 1. Yield of IgG production**

| **Antibody** | **Yield**  (mg/mL culture)^a^ | **Yield quality**  (folds above minimum)^b^ |
| --- | --- | --- |
| B04 | 0.33 | 3.3 |
| E01 | 0.42 | 4.2 |
| E08 | 0.29 | 2.9 |
| F07 | 0.41 | 4.1 |
| G07 | 0.41 | 4.1 |
| H01 | 0.35 | 3.5 |
| H05 | 0.41 | 4.1 |
| R04 | 0.27 | 2.7 |
| S01 | 0.38 | 3.8 |
| ^a^ from 100 mL culture  ^b^ yield/minimum yield (0.1 mg/mL) | | |

| **Table 2. Summary of data obtained for the best antibodies in various formats** | | | | | | |
| --- | --- | --- | --- | --- | --- | --- |
| **Selection strategy** | **Antibody name**  **(antigen)** | **Affinity for RBD**  (k_D,_ nM)^a^ | | **Non-competitive RBD2 binders** | **Competitive RBD2 binders** | **Recognize**  **D614G mutant?** |
| +C, CB^b^ | E01  (RBD2) | 13.9 ± 1.3 | scFv | E08, F07, G07, H01, H05, CR3022, and NN54 | B04, S01 and ACE2 | Yes |
|  |  | 21.5 ± 2.12 | Minibody |  |  |  |
|  |  | 90.0 ± 22.0 | IgG |  |  |  |
| -C, AB^c^ | S01  (RBD2) | 44.1 ± 2.5 | scFv | E08, F07, H05, CR3022, and NN54 | B04, E01, H01, and ACE2 | Yes |
|  |  | 22.7 E+3 | Minibody |  |  |  |
|  |  | 170.0 ± 40.0 | IgG |  |  |  |
| +C, CB^b^ | F07  (RBD2) | 67.5 ± 6.8 | scFv | E01, E08, H05, S01, CR3022, and NN54 | B04, G07, and H01 | Yes |
|  |  | 61.5 ± 3.5 | Minibody |  |  |  |
|  |  | 300.0 ± 65.0 | IgG |  |  |  |
| +C, CB^b^ | G07  (RBD2) | 60.3 ± 5.5 | scFv | E01, E08, H01, H05, S01,  CR3022, NN54, and ACE2 | B04, and F07 | Yes |
|  |  | 63.9 E+3 | Minibody |  |  |  |
|  |  | 320.0 ± 76.0 | IgG |  |  |  |
| +C, CB^b^ | B04  (RBD2) | 13.9 ± 1.5 | scFv | E08, H01, H05, CRR3022, NN54, and ACE2 | E01, F07, G07, and S01 | NT |
|  |  | 56.8 ± 3.5 | Minibody |  |  |  |
|  |  | 210 ± 42.4 | IgG |  |  |  |
| +C, CB^b^ | E08  (RBD2) | 13.9 ± 1.8 | scFv | B04, E01, F07, G07, S01, and ACE2 | H01, H05, and CR3022 | NT |
|  |  | 3.6 ± 2.0 | Minibody |  |  |  |
|  |  | 27.0 ± 3.2 | IgG |  |  |  |
| +C, CB^b^ | H01  (RBD2) | 66.4 ± 5.1 | scFv | B04, E01, and G07 | E08, F07, H05, S01, CR3022, and ACE2 | NT |
|  |  | 281.0 ± 162.6 | Minibody |  |  |  |
|  |  | 800.0 ± 440.0 | IgG |  |  |  |
| +C, CB^b^ | H05  (RBD2) | 16.7 ± 4.0 | scFv | B04, E01, F07, G07, and S01 | E08, H01, CR3022, and ACE2 | NT |
|  |  | 51 ± 8.6 | IgG |  |  |  |
| -C, AB^c^ | R04  (RBD2) | 293.3 ± 24.0 | scFv | ND | ND | NT |
|  |  | 127.5 ±7 0.0 | Minibody |  |  |  |
|  |  | 190.0 E+3 | IgG |  |  |  |
|  | R04  (RBD1) | 175.1 ± 20.3 | scFv | NT | NT | NT |
| ^a^ Determined either by flow cytometry (scFvs) or by surface plasmon resonance, SPR (minibodies or IgGs)  ^b^ +C = with competition; CB = chemically biotinylated target antigen  ^c^ -C = no competition; AB = target antigen biotinylated through the avitag | | | | | | |

| **Table 3:** Sensitivity of two sandwich immunoassays | | | | | | | | |  |
| --- | --- | --- | --- | --- | --- | --- | --- | --- | --- |
| **Assay** | **Antibody pair**^a^ | **Spike** | | **Spike D614G** | | **Whole virus** | | |  |
|  |  | **LoD**^b^ | **LoQ**^c^ | **LoD** | **LoQ** | **LoD** | | **LoQ** |  |
|  |  | (pM) | | (pM) | | (TCID50^d^/mL) | | |  |
| **ELISA**^e^ | S01/F07 | 1.22 | 18.2 | 664.2 | 1786.1 | > 2.5 E+4 | >53.7E+4 | |  |
|  | S01/G07 | 4.1 | 24.1 | 125.4 | 425.6 | > 2.5 E+4 | >53.7E+4 | |  |
|  | E01/G07 | 21.2 | 64.1 | 15.6 | 75.0 | 1.8E+4 | 12.0E+4 | |  |
|  | E01/F07 | 39.1 | 111.2 | 85.6 | 225.8 | 2.5 E+4 | 53.7E+4 | |  |
| **SpinDx**^f^ | S01/F07 | 0.16 | 26.5 | N/D^g^ | | | | |  |
|  | G07/S01 | 1.9 | 43.1 |  |  |  |  |  |  |
|  | S01/G07 | 10.3 | 26.5 |  |  |  |  |  |  |
| ^a^ (antigen-capturing IgG)/(antigen-detecting IgG)  ^b^ Limit of Detection  ^c^ Limit of Quantification  ^d^ Tissue culture infectious dose 50 (i.e. the dilution of virus required to infect 50% of the cell monolayers)  ^e^ Enzyme-linked immunosorbent assay  ^f^ Portable Multiplexed bead-based Immunoassay platform  ^g^ Not determined | | | | | | | | |  |
