# Supplementary material for: Healthy humans can be a source of antibodies countering COVID-19
Source: Bioengineered. 2022 May 21;13(5):12598–624. doi: 10.1080/21655979.2022.2076390 (PMC9275966; doi:10.1080/21655979.2022.2076390)
Supplement: Supplemental Material [file KBIE_A_2076390_SM0860.zip › raw data .zip!/raw data /Examples of flow cytometry plots (ARIA) obtained for yeast selections/Batch_Analysis_RBD sorts.pdf]

FACSDiva Version 6.1.3

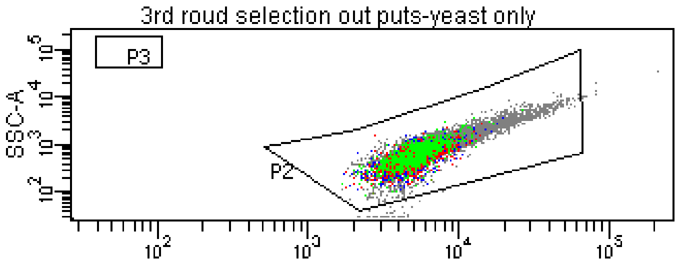

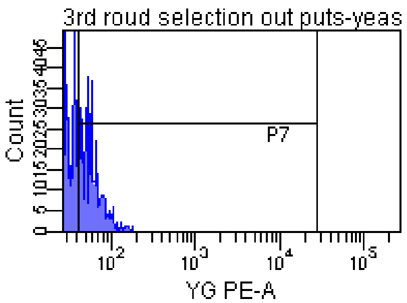

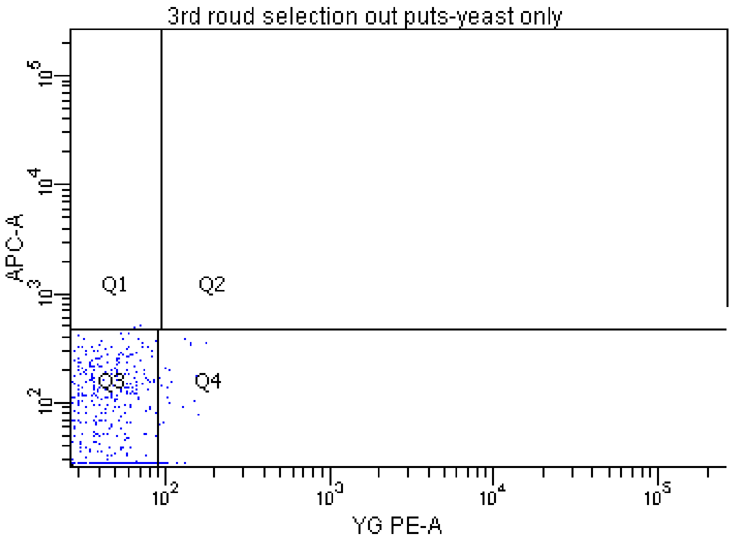

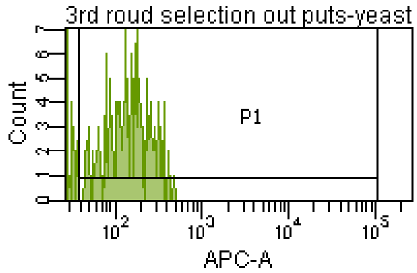

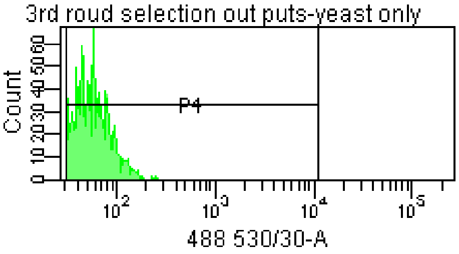

Experiment Name: Bio RBD2 selectons
Specimen Name: 3rd roud selection out puts
Tube Name: yeast only
Record Date: May 19, 2020 2:56:59 PM
$OP: Nileena
GUID: 3814fe31-802d-43e5-9c8e-7e0c493966a2

Population	Parent...	#Events	%Parent	%Gran...	%Total	488 5... Mean	488 5... Median	PE-A Mean	PE-A Median	APC-A Mean	APC-A Median
All Events	####	####	####	####	####	42	24	916	646	45	34
P2	####	####	####	####	####	42	24	908	649	45	34
P8	P2	4,703	47.1	46.8	46.8	-7	-7	508	461	15	6
Q1	P8	4	0.1	0.0	0.0	-12	3	343	312	505	496
Q2	P8	0	0.0	0.0	0.0	####	####	####	####	####	####
Q3	P8	4,668	99.3	46.7	46.4	-7	-7	508	461	14	6
Q4	P8	31	0.7	0.3	0.3	29	28	558	514	100	107
P7	P8	365	7.8	3.7	3.6	19	19	557	514	29	23
P1	P8	1,952	41.5	19.5	19.4	-11	-10	534	483	145	127
P4	P8	1,103	23.5	11.0	11.0	62	56	608	540	8	-3

FACSDiva Version 6.1.3

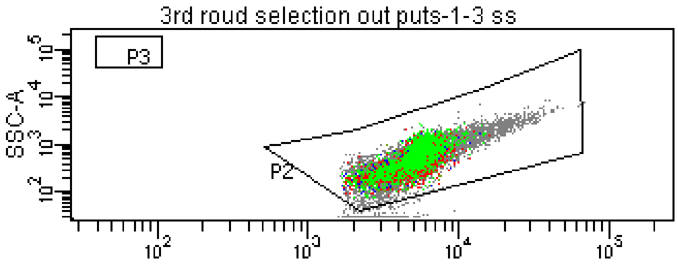

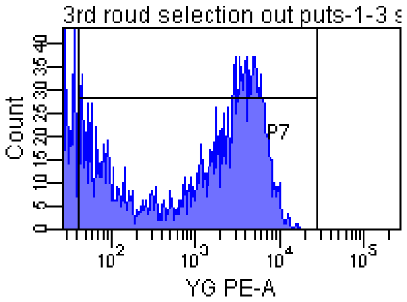

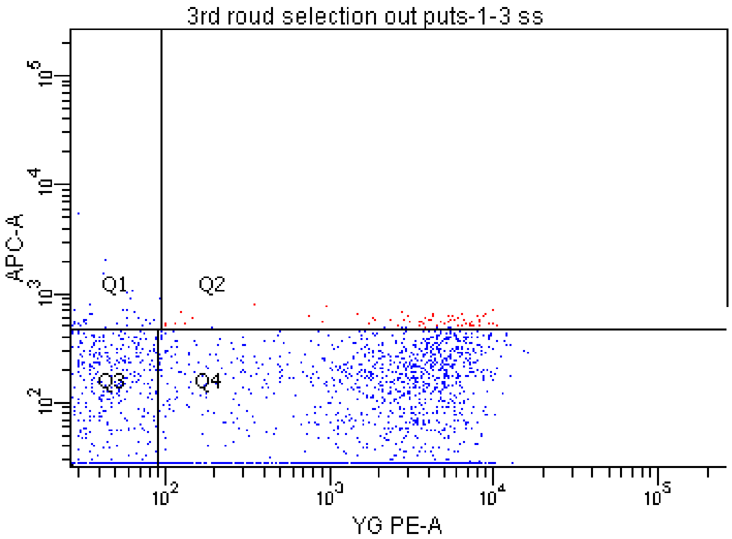

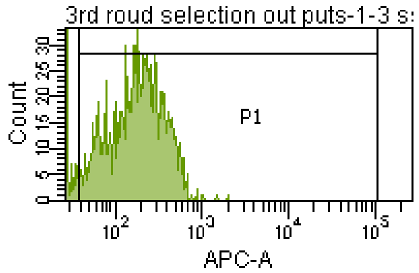

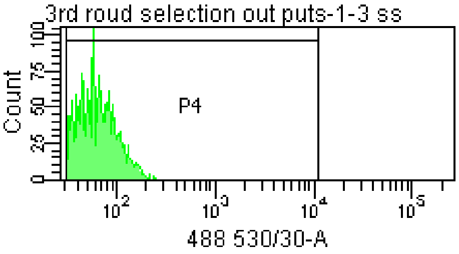

Experiment Name: Bio RBD2 selectons
Specimen Name: 3rd roud selection out puts
Tube Name: 1-3 ss
Record Date: May 19, 2020 2:58:33 PM
$OP: Nileena
GUID: 2023999a-077e-4fb7-b2dd-baad19a62def

Population	Parent...	#Events	%Parent	%Gran...	%Total	488 5... Mean	488 5... Median	PE-A Mean	PE-A Median	APC-A Mean	APC-A Median
All Events	####	####	####	####	####	56	36	1,370	817	116	83
P2	####	####	####	####	####	59	38	1,386	824	117	85
P8	P2	5,116	50.9	49.7	49.7	12	12	875	629	82	60
Q1	P8	109	2.1	1.1	1.1	5	5	609	544	905	586
Q2	P8	65	1.3	0.6	0.6	24	27	2,218	2,130	548	532
Q3	P8	3,226	63.1	32.1	31.3	6	7	505	453	52	42
Q4	P8	1,716	33.5	17.1	16.7	21	20	1,536	1,376	68	64
P7	P8	2,155	42.1	21.5	20.9	21	20	1,389	1,173	85	69
P1	P8	2,785	54.4	27.7	27.0	11	12	958	678	230	186
P4	P8	1,849	36.1	18.4	18.0	70	62	1,117	827	86	62

FACSDiva Version 6.1.3

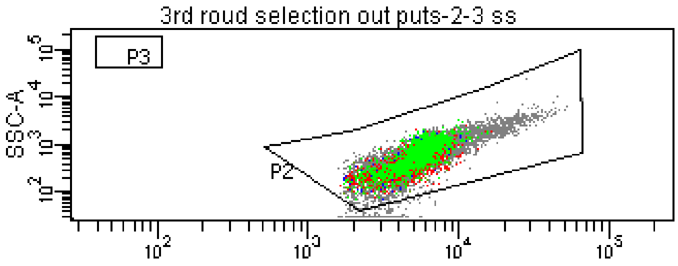

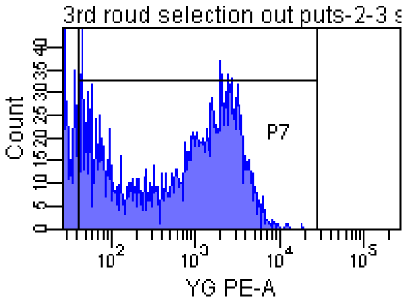

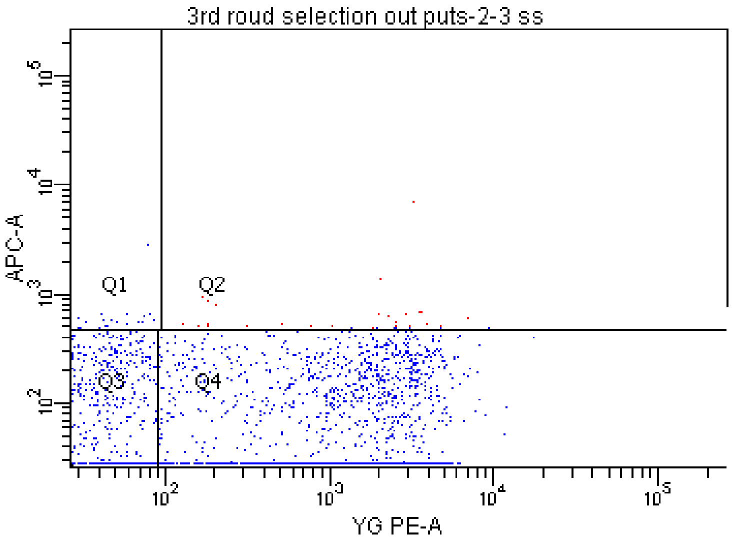

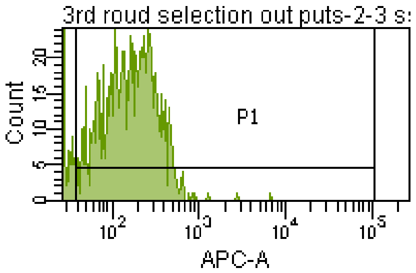

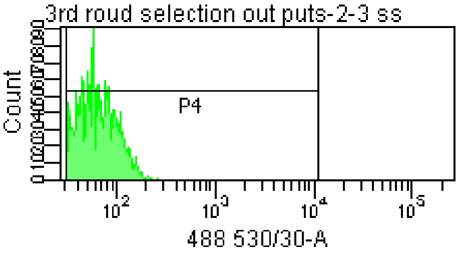

Experiment Name: Bio RBD2 selectons
Specimen Name: 3rd roud selection out puts
Tube Name: 2-3 ss
Record Date: May 19, 2020 3:00:24 PM
$OP: Nileena
GUID: eb1245cd-f6dc-4dfe-91c7-88fe310a4476

Population	Parent...	#Events	%Parent	%Gran...	%Total	488 5... Mean	488 5... Median	PE-A Mean	PE-A Median	APC-A Mean	APC-A Median
All Events	####	####	####	####	####	50	32	1,023	697	92	58
P2	####	####	####	####	####	53	34	1,038	711	94	60
P8	P2	5,061	50.4	49.2	49.2	10	9	689	569	61	39
Q1	P8	85	1.7	0.8	0.8	2	-3	579	486	807	582
Q2	P8	26	0.5	0.3	0.3	30	17	1,456	1,289	832	516
Q3	P8	3,302	65.2	32.9	32.1	8	7	510	455	44	34
Q4	P8	1,648	32.6	16.4	16.0	13	13	1,042	926	43	34
P7	P8	2,083	41.2	20.7	20.2	17	16	962	848	57	34
P1	P8	2,538	50.1	25.3	24.7	11	10	750	611	206	164
P4	P8	1,708	33.7	17.0	16.6	70	62	848	747	67	48

FACSDiva Version 6.1.3

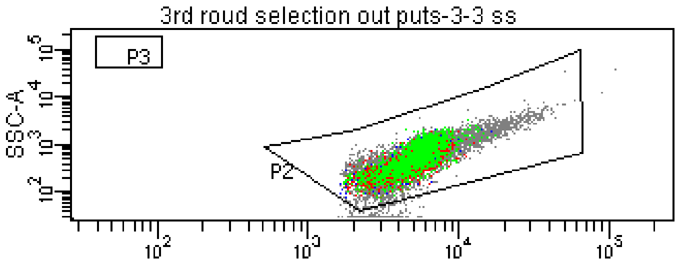

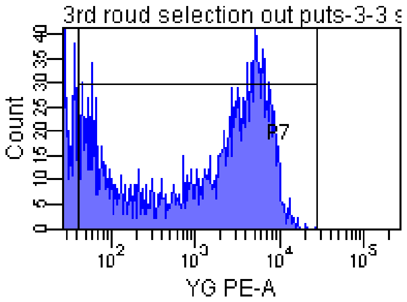

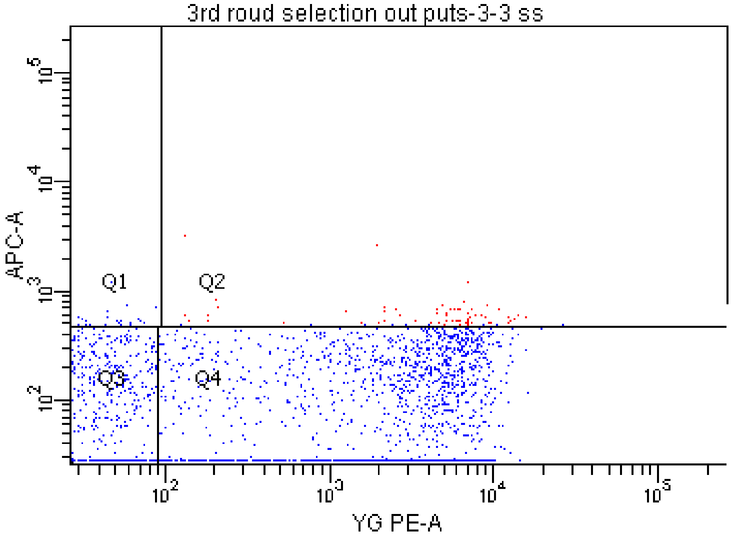

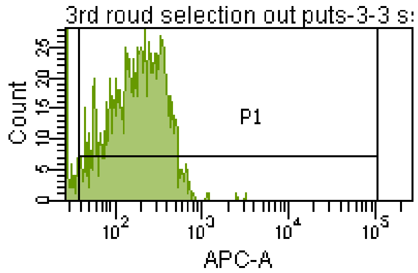

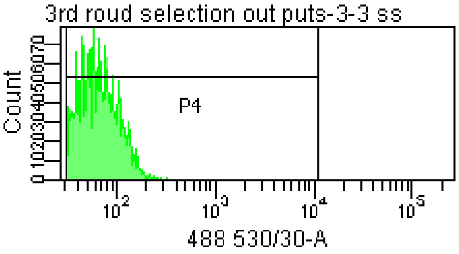

Experiment Name: Bio RBD2 selectons
Specimen Name: 3rd roud selection out puts
Tube Name: 3-3 ss
Record Date: May 19, 2020 3:01:29 PM
$OP: Nileena
GUID: 985c9fbe-39f0-44f5-abcf-135a7083b74c

Population	Parent...	#Events	%Parent	%Gran...	%Total	488 5... Mean	488 5... Median	PE-A Mean	PE-A Median	APC-A Mean	APC-A Median
All Events	####	####	####	####	####	60	39	1,482	834	135	83
P2	####	####	####	####	####	62	40	1,492	839	133	85
P8	P2	5,003	49.8	48.6	48.6	15	14	952	628	82	59
Q1	P8	114	2.3	1.1	1.1	5	-2	565	496	830	566
Q2	P8	60	1.2	0.6	0.6	37	31	2,640	2,457	648	548
Q3	P8	3,100	62.0	30.9	30.1	7	8	513	455	47	39
Q4	P8	1,729	34.6	17.2	16.8	27	26	1,707	1,478	76	70
P7	P8	2,134	42.7	21.3	20.7	27	26	1,548	1,254	90	76
P1	P8	2,708	54.1	27.0	26.3	15	14	1,072	693	233	186
P4	P8	1,871	37.4	18.6	18.2	72	63	1,298	917	82	62

FACSDiva Version 6.1.3

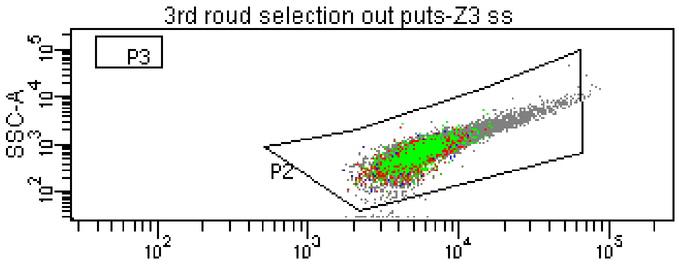

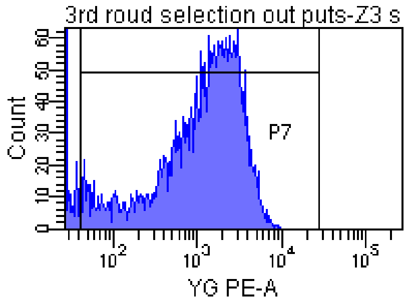

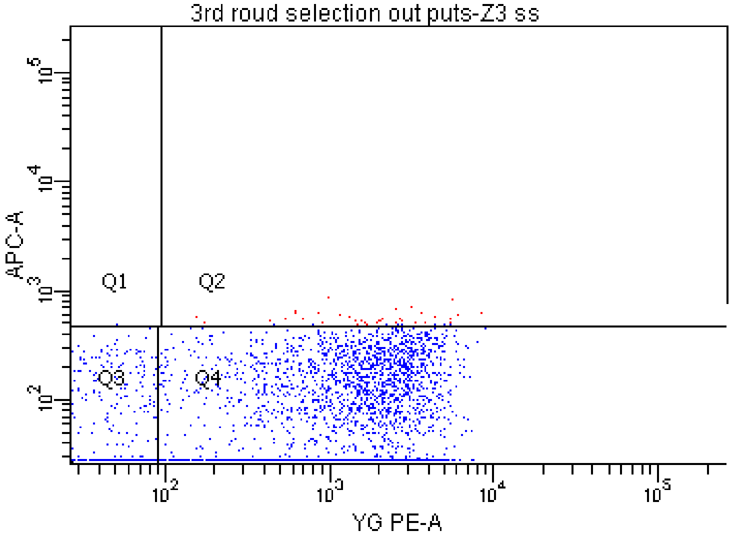

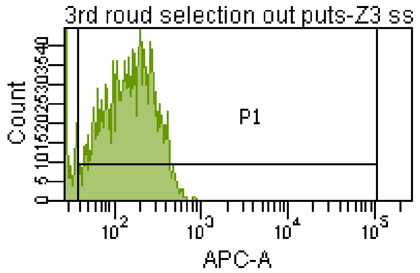

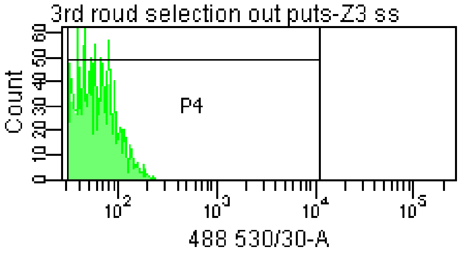

Experiment Name: Bio RBD2 selectons
Specimen Name: 3rd roud selection out puts
Tube Name: Z3 ss
Record Date: May 19, 2020 3:02:35 PM
$OP: Nileena
GUID: b7bc03fc-2817-42cf-93fc-0a7fe37437b6

Population	Parent...	#Events	%Parent	%Gran...	%Total	488 5... Mean	488 5... Median	PE-A Mean	PE-A Median	APC-A Mean	APC-A Median
All Events	####	####	####	####	####	51	29	1,569	1,109	114	59
P2	####	####	####	####	####	51	31	1,554	1,112	110	59
P8	P2	4,784	47.9	47.6	47.6	0	0	964	826	40	26
Q1	P8	2	0.0	0.0	0.0	-31	-31	128	128	8,718	8,718
Q2	P8	35	0.7	0.4	0.3	24	26	1,702	1,239	549	525
Q3	P8	1,495	31.2	15.0	14.9	-6	-7	495	457	14	7
Q4	P8	3,252	68.0	32.5	32.4	3	2	1,172	1,064	42	32
P7	P8	3,495	73.1	35.0	34.8	4	3	1,141	1,026	46	34
P1	P8	2,249	47.0	22.5	22.4	0	-1	1,076	938	182	151
P4	P8	1,349	28.2	13.5	13.4	66	58	1,216	1,057	42	23

FACSDiva Version 6.1.3

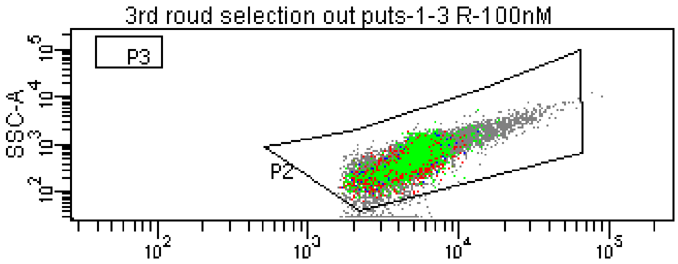

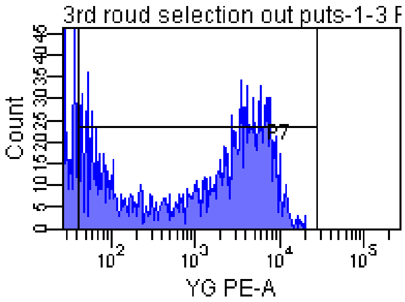

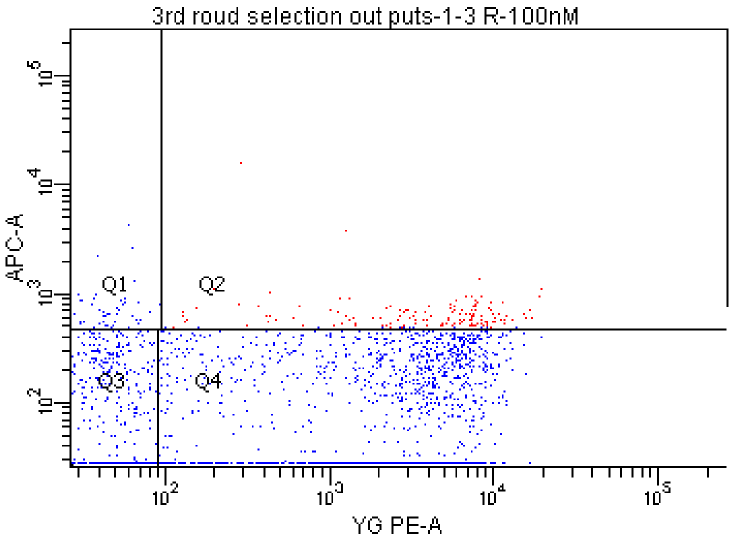

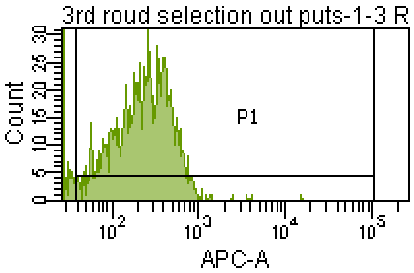

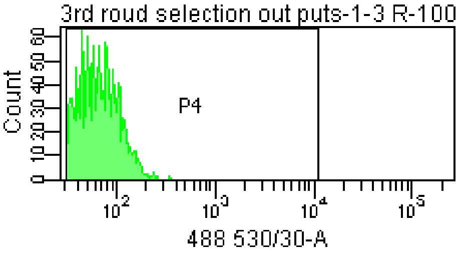

Experiment Name: Bio RBD2 selectons
Specimen Name: 3rd roud selection out puts
Tube Name: 1-3 R-100nM
Record Date: May 19, 2020 3:32:48 PM
$OP: Nileena
GUID: 47eeeba9-5c8f-47fe-88d3-d6b40cf2e5ad

Population	Parent...	#Events	%Parent	%Gran...	%Total	488 5... Mean	488 5... Median	PE-A Mean	PE-A Median	APC-A Mean	APC-A Median
All Events	####	####	####	####	####	64	40	1,529	822	183	109
P2	####	####	####	####	####	67	43	1,545	827	186	109
P8	P2	4,565	45.4	44.0	44.0	9	8	920	588	110	78
Q1	P8	206	4.5	2.0	2.0	-3	-2	533	467	996	606
Q2	P8	136	3.0	1.4	1.3	25	17	2,616	2,380	750	582
Q3	P8	2,799	61.3	27.8	27.0	3	2	491	433	32	32
Q4	P8	1,424	31.2	14.2	13.7	21	19	1,659	1,429	74	86
P7	P8	1,903	41.7	18.9	18.4	21	17	1,533	1,202	128	102
P1	P8	2,523	55.3	25.1	24.3	9	7	1,048	632	310	232
P4	P8	1,555	34.1	15.5	15.0	72	64	1,286	820	104	77

FACSDiva Version 6.1.3

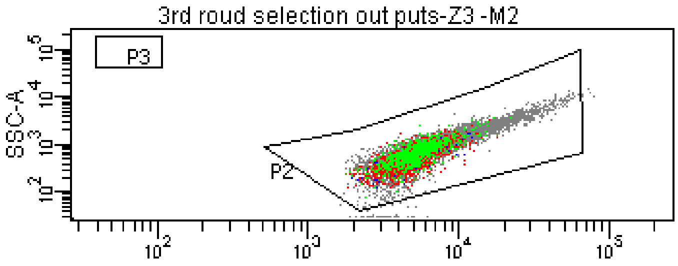

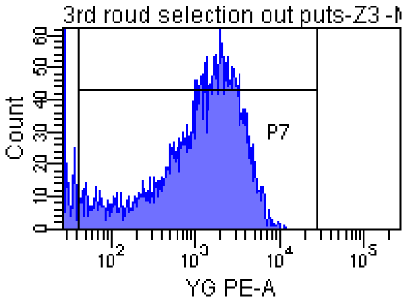

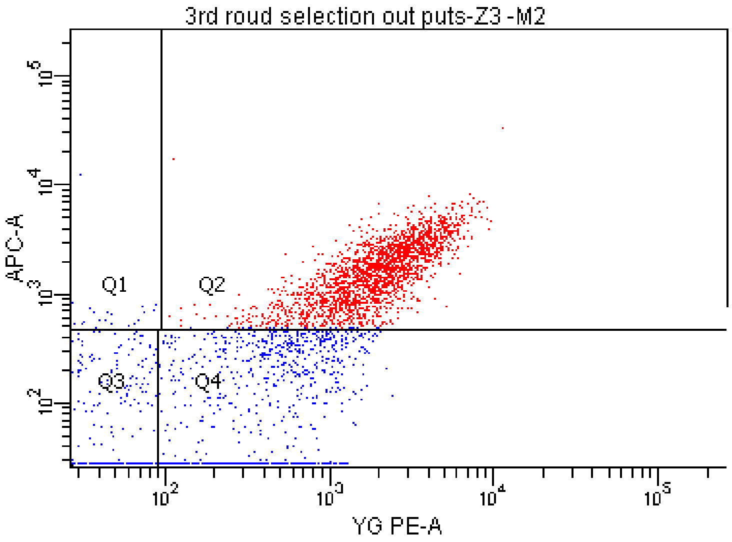

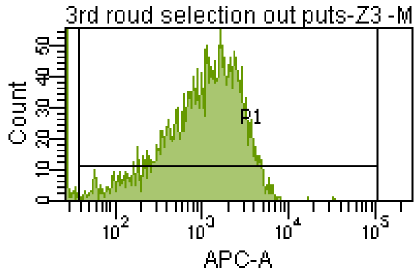

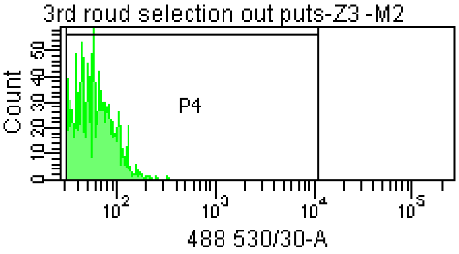

Experiment Name: Bio RBD2 selectons
Specimen Name: 3rd roud selection out puts
Tube Name: Z3 -M2
Record Date: May 19, 2020 3:34:00 PM
$OP: Nileena
GUID: 58de9918-9884-41a4-af69-42fc118b21ce

Population	Parent...	#Events	%Parent	%Gran...	%Total	488 5... Mean	488 5... Median	PE-A Mean	PE-A Median	APC-A Mean	APC-A Median
All Events	####	####	####	####	####	48	27	1,564	1,081	1,475	774
P2	####	####	####	####	####	48	28	1,563	1,085	1,468	776
P8	P2	4,349	43.5	43.2	43.2	-5	-4	957	798	983	540
Q1	P8	76	1.7	0.8	0.8	-15	-14	487	423	1,397	616
Q2	P8	2,209	50.8	22.1	22.0	2	3	1,368	1,212	1,840	1,538
Q3	P8	1,251	28.8	12.5	12.4	-13	-11	467	401	-8	-19
Q4	P8	813	18.7	8.1	8.1	-12	-13	641	572	143	164
P7	P8	3,199	73.6	32.0	31.8	-2	-1	1,136	986	1,309	978
P1	P8	3,353	77.1	33.5	33.3	-3	-2	1,101	952	1,320	919
P4	P8	1,057	24.3	10.6	10.5	65	58	1,249	1,059	1,343	938

FACSDiva Version 6.1.3

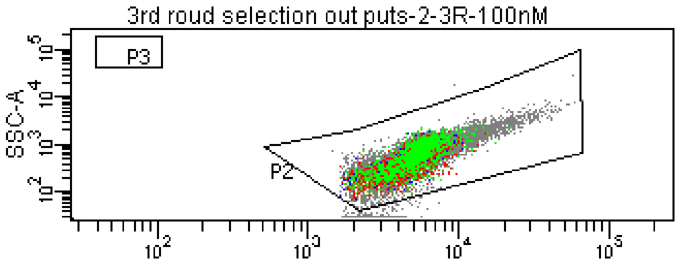

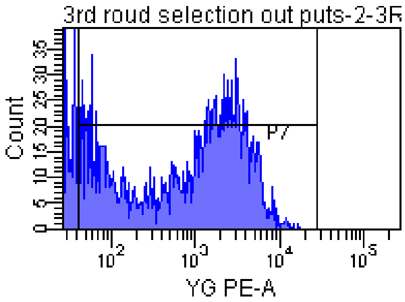

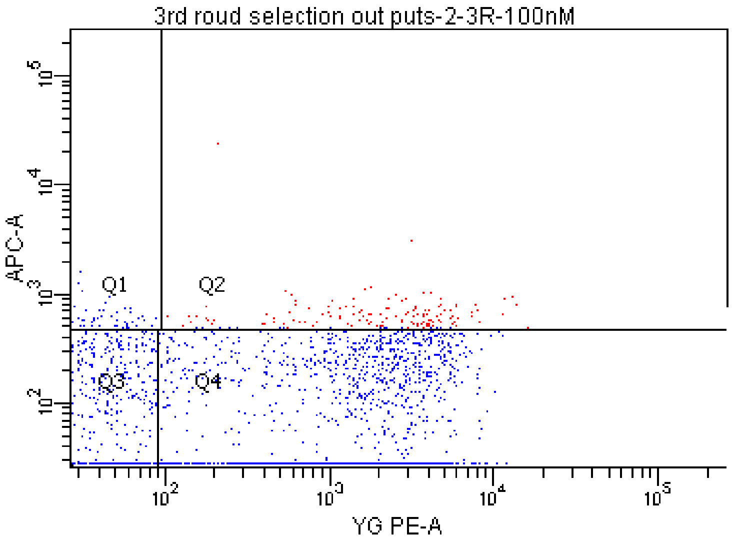

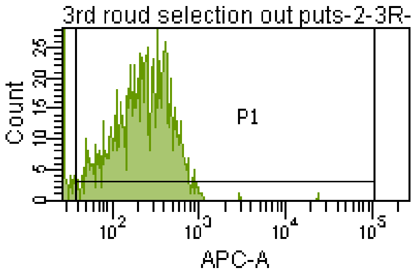

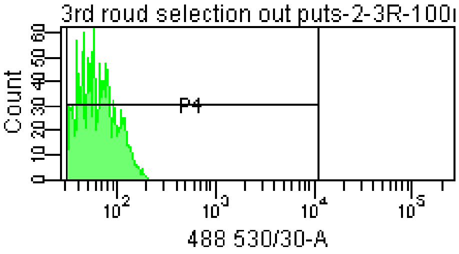

Experiment Name: Bio RBD2 selectons
Specimen Name: 3rd roud selection out puts
Tube Name: 2-3R-100nM
Record Date: May 19, 2020 3:35:20 PM
$OP: Nileena
GUID: 49c71d20-64d8-4372-8918-c322a2bf6985

Population	Parent...	#Events	%Parent	%Gran...	%Total	488 5... Mean	488 5... Median	PE-A Mean	PE-A Median	APC-A Mean	APC-A Median
All Events	####	####	####	####	####	54	34	1,170	725	240	85
P2	####	####	####	####	####	57	37	1,187	739	227	86
P8	P2	4,397	43.8	42.6	42.6	4	3	722	557	98	57
Q1	P8	223	5.1	2.2	2.2	6	0	478	449	1,034	624
Q2	P8	131	3.0	1.3	1.3	18	15	1,568	1,274	820	598
Q3	P8	2,612	59.4	26.0	25.3	-0	-1	476	423	13	20
Q4	P8	1,431	32.5	14.2	13.9	10	10	1,132	959	43	49
P7	P8	1,903	43.3	18.9	18.4	12	12	1,056	883	99	77
P1	P8	2,316	52.7	23.1	22.4	5	2	783	584	328	231
P4	P8	1,345	30.6	13.4	13.0	69	60	930	726	104	69

FACSDiva Version 6.1.3

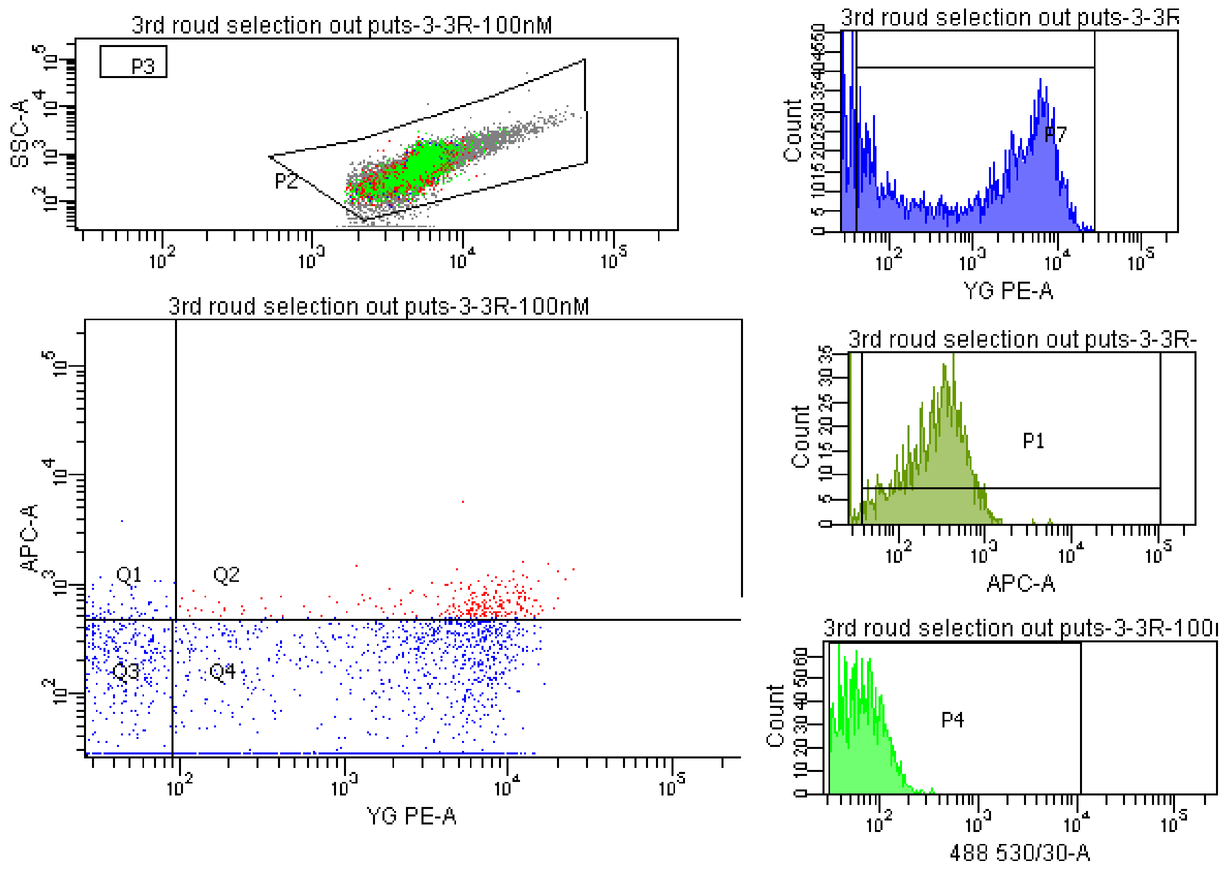

Experiment Name: Bio RBD2 selectons
Specimen Name: 3rd roud selection out puts
Tube Name: 3-3R-100nM
Record Date: May 19, 2020 3:36:36 PM
$OP: Nileena
GUID: 6ee1502f-683d-41db-a77e-04eadafc635a

Population	Parent...	#Events	%Parent	%Gran...	%Total	488 5... Mean	488 5... Median	PE-A Mean	PE-A Median	APC-A Mean	APC-A Median
All Events	####	####	####	####	####	64	40	1,709	876	264	132
P2	####	####	####	####	####	66	43	1,722	877	253	131
P8	P2	4,669	46.5	45.2	45.2	11	9	1,030	623	135	91
Q1	P8	279	6.0	2.8	2.7	-8	-5	492	440	1,038	625
Q2	P8	249	5.3	2.5	2.4	46	41	3,058	2,899	694	615
Q3	P8	2,677	57.3	26.6	25.9	2	1	498	439	22	26
Q4	P8	1,464	31.4	14.6	14.2	25	23	1,761	1,504	73	82
P7	P8	2,051	43.9	20.4	19.9	25	23	1,724	1,347	150	126
P1	P8	2,648	56.7	26.4	25.7	13	10	1,201	680	360	270
P4	P8	1,651	35.4	16.4	16.0	74	65	1,495	942	141	106

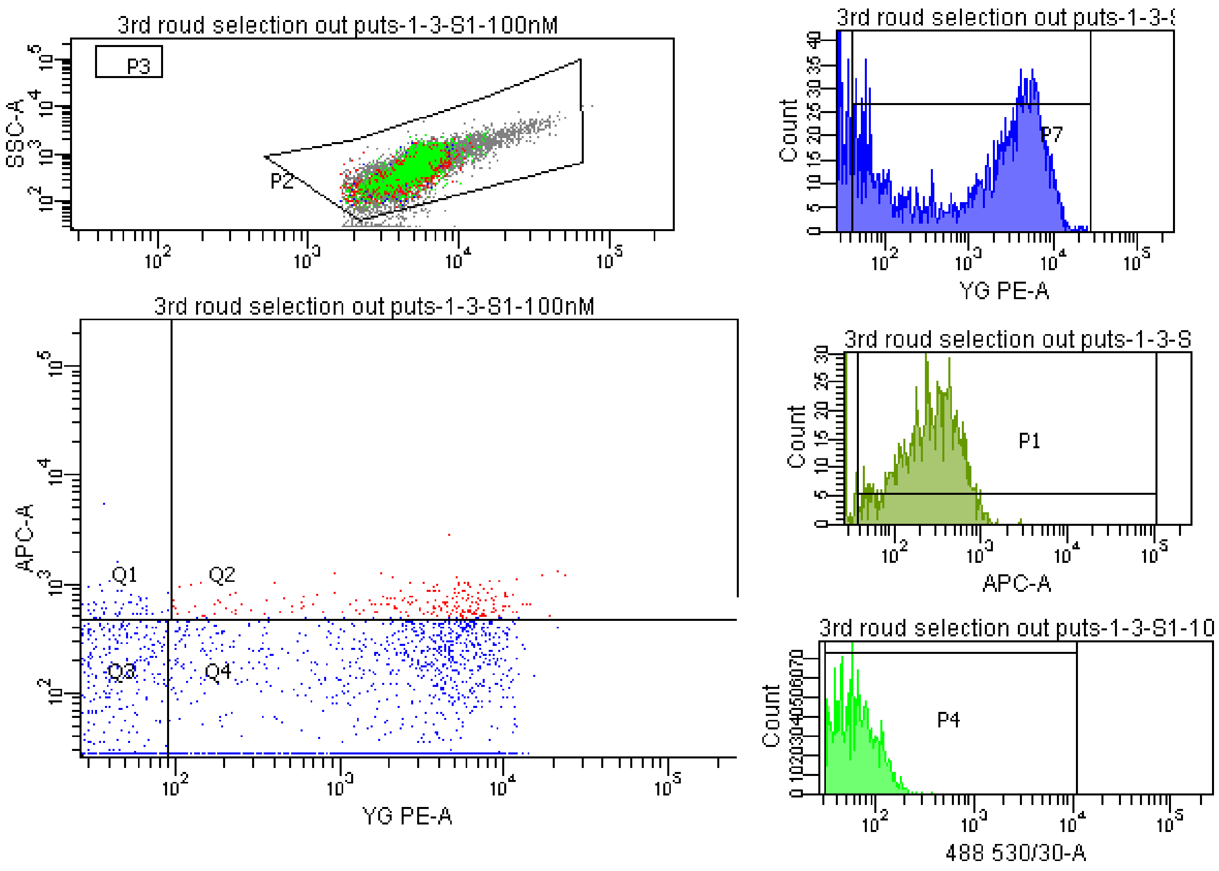

Experiment Name: Bio RBD2 selectons
Specimen Name: 3rd roud selection out puts
Tube Name: 1-3-S1-100nM
Record Date: May 19, 2020 3:38:03 PM
$OP: Nileena
GUID: ab9e75dd-94b4-453d-b872-79dab82b5dab

Population	Parent...	#Events	%Parent	%Gran...	%Total	488 5... Mean	488 5... Median	PE-A Mean	PE-A Median	APC-A Mean	APC-A Median
All Events	####	####	####	####	####	58	37	1,461	799	184	101
P2	####	####	####	####	####	60	39	1,473	802	186	102
P8	P2	4,718	47.0	45.6	45.6	10	8	911	598	107	68
Q1	P8	287	6.1	2.9	2.8	-1	-8	536	456	925	613
Q2	P8	206	4.4	2.1	2.0	33	34	2,215	1,903	676	615
Q3	P8	2,816	59.7	28.0	27.2	3	3	483	424	9	14
Q4	P8	1,409	29.9	14.0	13.6	21	20	1,651	1,408	51	73
P7	P8	1,950	41.3	19.4	18.9	22	21	1,526	1,239	118	104
P1	P8	2,541	53.9	25.3	24.6	10	8	1,027	655	340	258
P4	P8	1,627	34.5	16.2	15.7	70	61	1,247	831	100	72

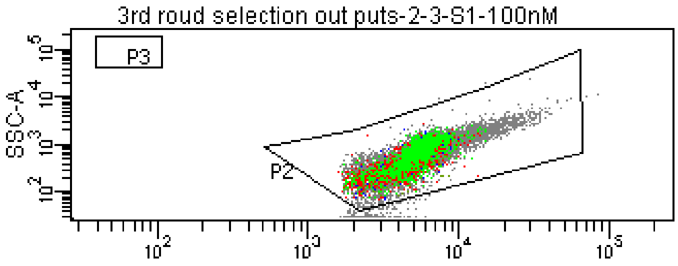

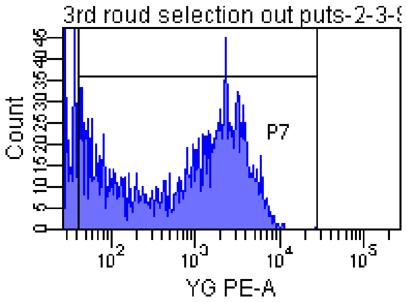

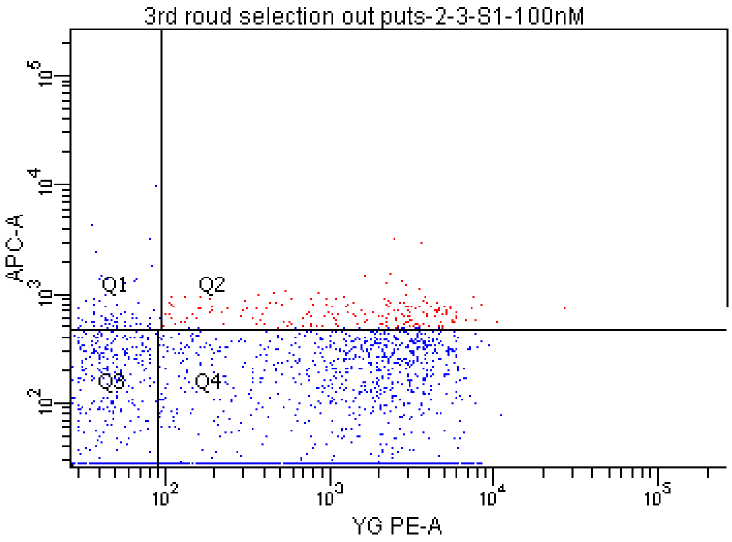

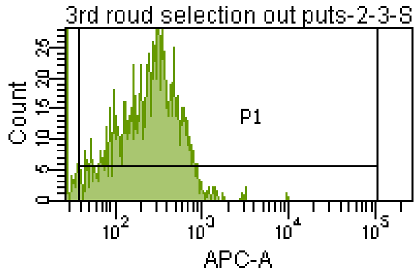

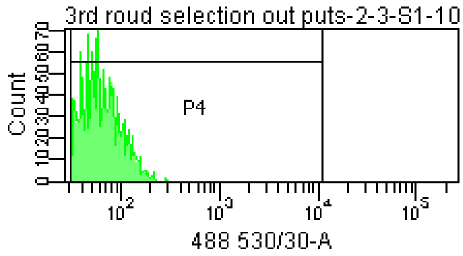

Experiment Name: Bio RBD2 selectons
Specimen Name: 3rd roud selection out puts
Tube Name: 2-3-S1-100nM
Record Date: May 19, 2020 3:39:13 PM
$OP: Nileena
GUID: c3fad22f-9abd-42d4-8077-bb43dc8d897a

Population	Parent...	#Events	%Parent	%Gran...	%Total	488 5... Mean	488 5... Median	PE-A Mean	PE-A Median	APC-A Mean	APC-A Median
All Events	####	####	####	####	####	58	33	1,111	703	281	95
P2	####	####	####	####	####	60	34	1,119	713	233	95
P8	P2	4,756	47.3	46.4	46.4	7	5	699	558	105	68
Q1	P8	368	7.7	3.7	3.6	-2	-4	498	441	874	609
Q2	P8	178	3.7	1.8	1.7	16	14	1,326	1,063	693	633
Q3	P8	2,734	57.5	27.2	26.6	3	2	472	423	3	16
Q4	P8	1,476	31.0	14.7	14.4	15	14	1,093	953	30	37
P7	P8	2,019	42.5	20.1	19.7	15	15	1,010	856	104	79
P1	P8	2,560	53.8	25.5	25.0	5	4	739	588	348	267
P4	P8	1,488	31.3	14.8	14.5	69	59	909	716	96	71

FACSDiva Version 6.1.3

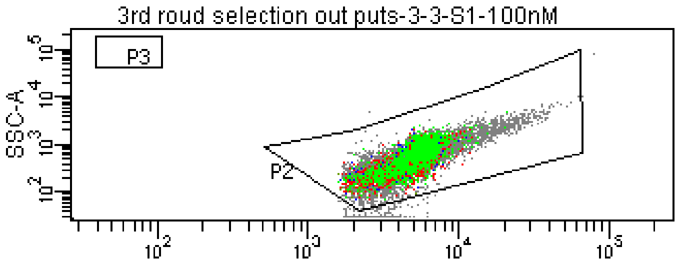

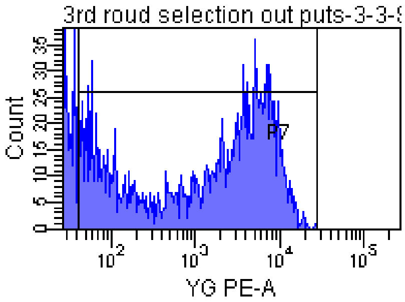

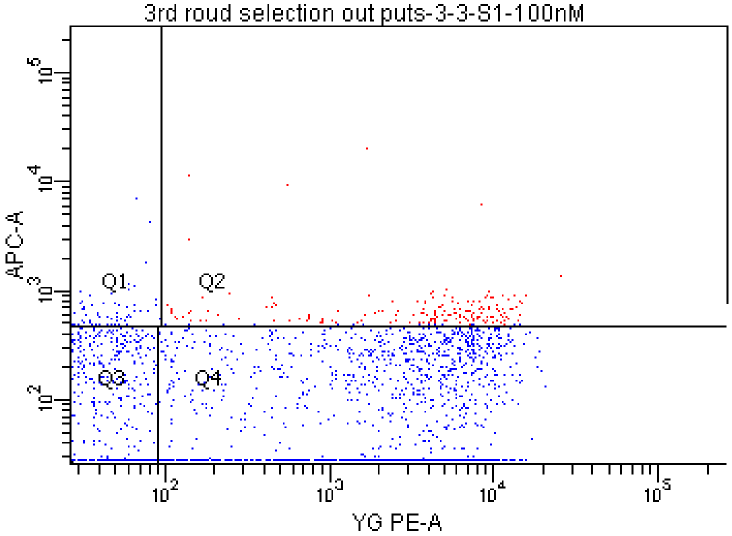

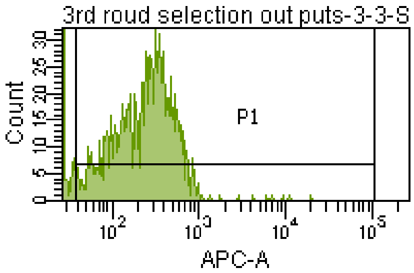

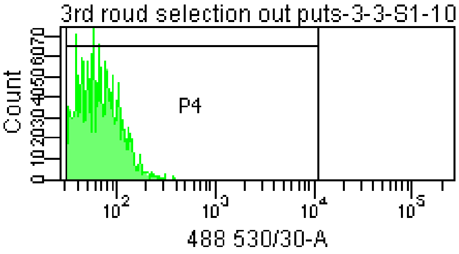

Experiment Name: Bio RBD2 selectons
Specimen Name: 3rd roud selection out puts
Tube Name: 3-3-S1-100nM
Record Date: May 19, 2020 3:40:37 PM
$OP: Nileena
GUID: 7081e5bc-2816-4101-816e-a8b0214353fc

Population	Parent...	#Events	%Parent	%Gran...	%Total	488 5... Mean	488 5... Median	PE-A Mean	PE-A Median	APC-A Mean	APC-A Median
All Events	####	####	####	####	####	62	39	1,577	818	254	103
P2	####	####	####	####	####	64	41	1,583	821	213	104
P8	P2	4,715	46.9	45.6	45.6	11	10	961	603	158	77
Q1	P8	239	5.1	2.4	2.3	-3	-2	462	412	1,695	604
Q2	P8	168	3.6	1.7	1.6	37	31	2,448	2,063	895	590
Q3	P8	2,838	60.2	28.2	27.5	5	4	495	443	33	34
Q4	P8	1,470	31.2	14.6	14.2	24	22	1,770	1,412	67	77
P7	P8	1,974	41.9	19.6	19.1	25	23	1,625	1,239	149	100
P1	P8	2,618	55.5	26.0	25.3	13	11	1,081	647	347	240
P4	P8	1,650	35.0	16.4	16.0	72	63	1,393	881	131	86

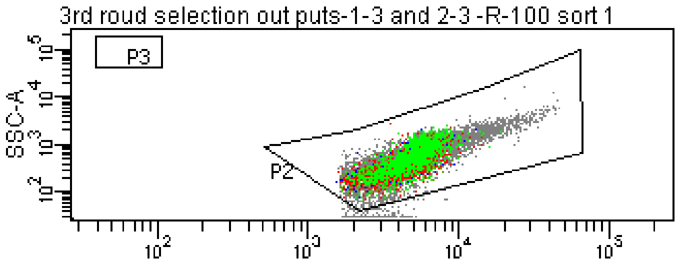

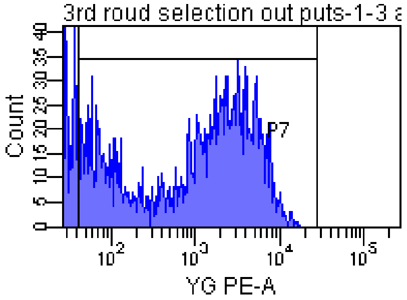

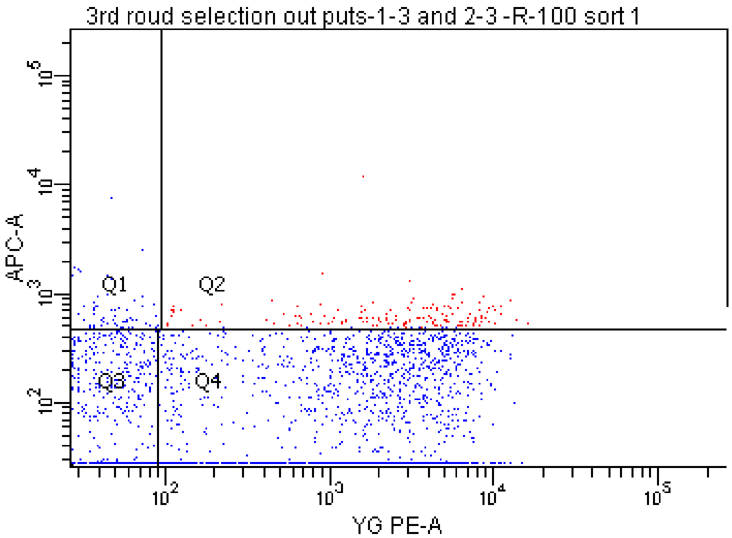

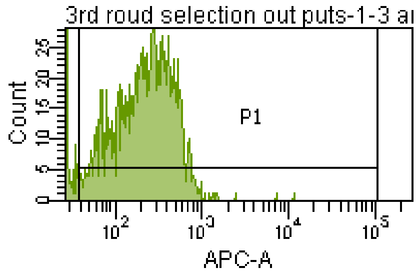

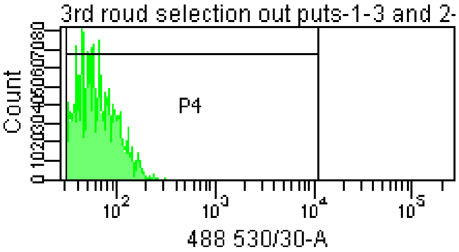

Experiment Name:	Bio RBD2 selectons
Specimen Name:	3rd roud selection out puts
Tube Name:	1-3 and 2-3 -R-100 sort 1
Record Date:	May 19, 2020 4:29:15 PM
$OP:	Nileena
GUID:	1145a0fe-7258-45c1-b2ec-c78a775d253d

Population	Parent...	#Events	%Parent	%Gran...	%Total	488 5... Mean	488 5... Median	PE-A Mean	PE-A Median	APC-A Mean	APC-A Median
All Events	####	####	####	####	####	53	33	1,088	617	197	94
P2	####	####	####	####	####	55	35	1,101	621	201	95
P8	P2	5,066	50.3	49.1	49.1	11	10	700	465	99	66
Q1	P8	243	4.8	2.4	2.4	4	4	394	339	963	600
Q2	P8	149	2.9	1.5	1.4	31	40	1,789	1,374	685	568
Q3	P8	3,012	59.5	29.9	29.2	6	5	369	318	26	24
Q4	P8	1,662	32.8	16.5	16.1	19	16	1,248	990	52	63
P7	P8	2,162	42.7	21.5	20.9	20	19	1,159	865	108	82
P1	P8	2,727	53.8	27.1	26.4	12	11	783	504	308	234
P4	P8	1,751	34.6	17.4	17.0	69	59	963	635	98	77

FACSDiva Version 6.1.3

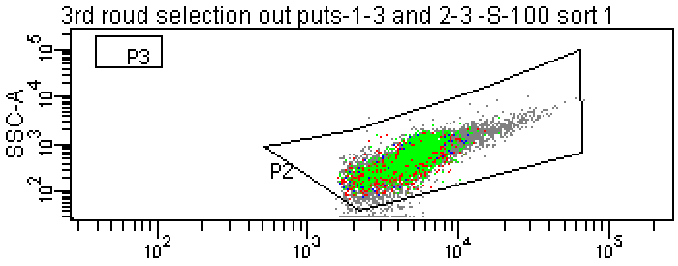

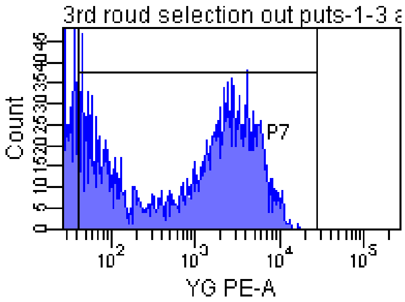

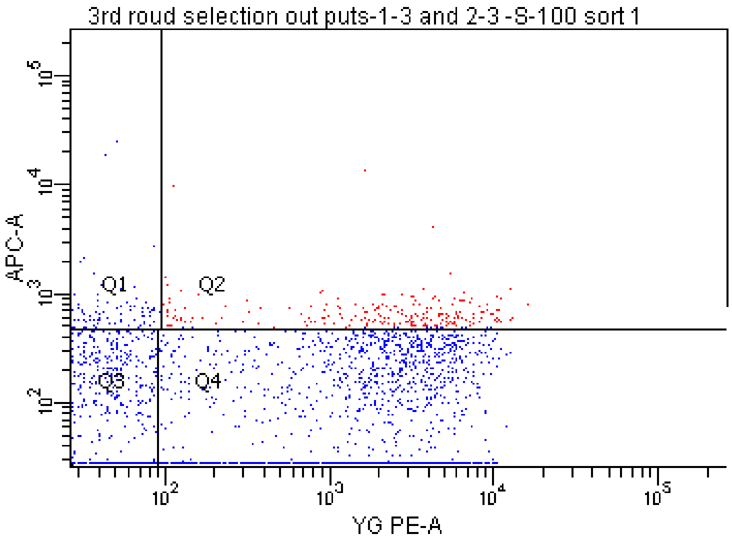

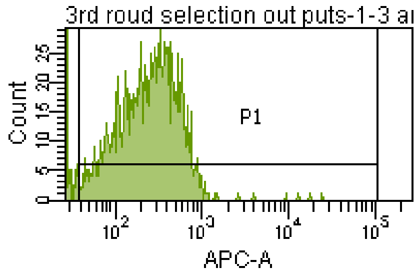

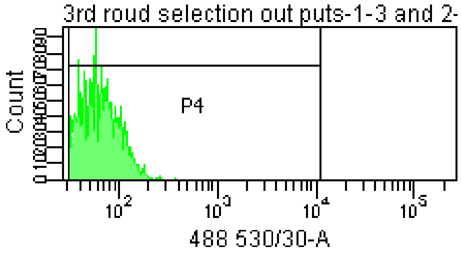

Experiment Name: Bio RBD2 selectons
Specimen Name: 3rd roud selection out puts
Tube Name: 1-3 and 2-3 -S-100 sort 1
Record Date: May 19, 2020 4:44:27 PM
$OP: Nileena
GUID: 554c6167-7ac5-4914-9cea-bc1274e78270

Population	Parent...	#Events	%Parent	%Gran...	%Total	488 5... Mean	488 5... Median	PE-A Mean	PE-A Median	APC-A Mean	APC-A Median
All Events	####	####	####	####	####	49	31	1,081	592	271	99
P2	####	####	####	####	####	51	33	1,090	594	249	99
P8	P2	5,393	53.6	52.2	52.2	10	9	684	450	119	70
Q1	P8	336	6.2	3.3	3.3	4	2	376	377	1,072	627
Q2	P8	209	3.9	2.1	2.0	28	21	1,727	1,348	759	589
Q3	P8	3,255	60.4	32.3	31.5	5	3	357	316	14	20
Q4	P8	1,593	29.5	15.8	15.4	20	20	1,280	1,061	47	59
P7	P8	2,243	41.6	22.3	21.7	21	20	1,155	910	136	89
P1	P8	2,916	54.1	29.0	28.2	11	8	756	477	356	250
P4	P8	1,836	34.0	18.2	17.8	70	62	941	622	89	70

FACSDiva Version 6.1.3

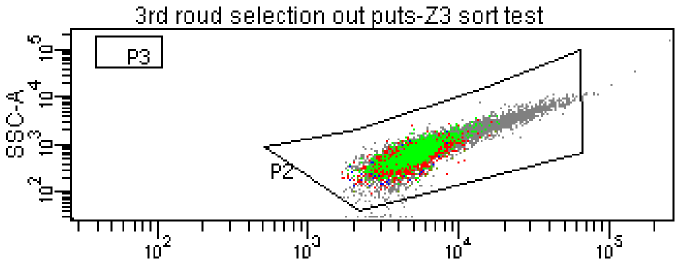

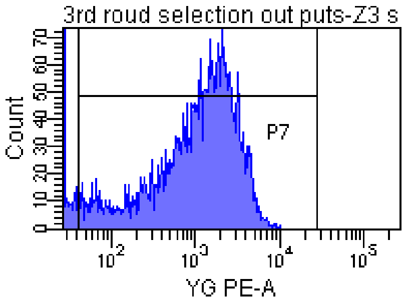

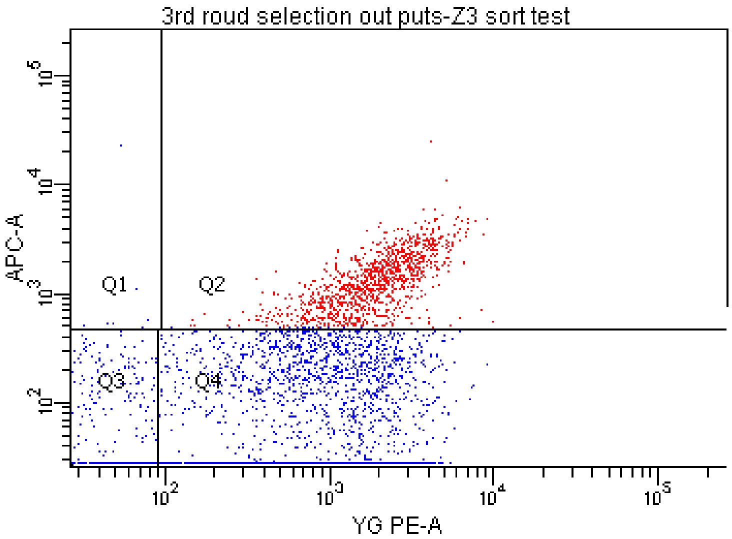

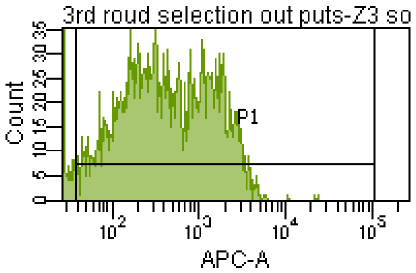

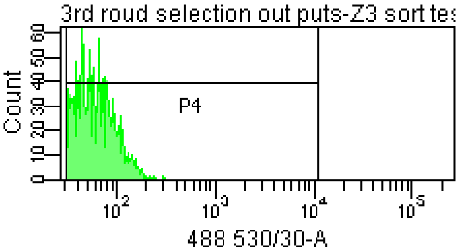

Experiment Name: Bio RBD2 selectons
Specimen Name: 3rd roud selection out puts
Tube Name: Z3 sort test
Record Date: May 19, 2020 4:57:24 PM
$OP: Nileena
GUID: a0c0381c-ec20-4434-b1ed-3edf0cb54ed6

Population	Parent...	#Events	%Parent	%Gran...	%Total	488 5... Mean	488 5... Median	PE-A Mean	PE-A Median	APC-A Mean	APC-A Median
All Events	####	####	####	####	####	48	28	1,437	919	653	178
P2	####	####	####	####	####	48	28	1,411	921	624	178
P8	P2	4,791	47.9	47.7	47.7	-1	-1	810	656	401	123
Q1	P8	23	0.5	0.2	0.2	-18	-37	444	429	1,586	595
Q2	P8	1,145	23.9	11.5	11.4	7	7	1,277	1,158	1,498	1,248
Q3	P8	1,434	29.9	14.3	14.3	-10	-10	332	286	3	-11
Q4	P8	2,189	45.7	21.9	21.8	1	2	883	752	75	72
P7	P8	3,543	74.0	35.4	35.3	3	3	982	850	536	195
P1	P8	2,998	62.6	30.0	29.8	2	1	935	785	704	311
P4	P8	1,254	26.2	12.5	12.5	66	58	1,058	887	538	176

FACSDiva Version 6.1.3

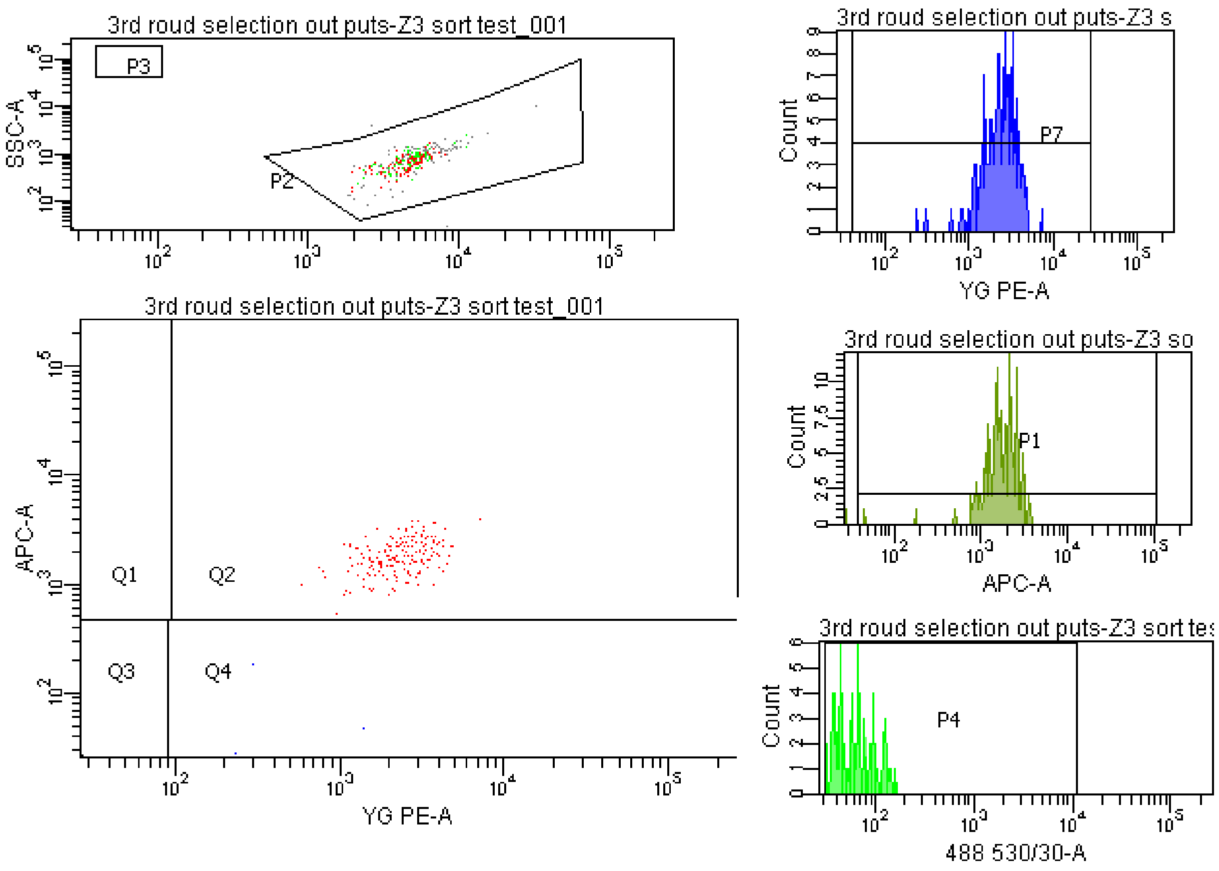

Experiment Name: Bio RBD2 selectons
Specimen Name: 3rd roud selection out puts
Tube Name: Z3 sort test_001
Record Date: May 19, 2020 5:01:19 PM
$OP: Nileena
GUID: f5ed5b32-017e-46af-ad39-3bd5abc8da31

Population	Parent...	#Events	%Parent	%Gran...	%Total	488 5... Mean	488 5... Median	PE-A Mean	PE-A Median	APC-A Mean	APC-A Median
All Events	####	####	####	####	####	37	32	1,471	1,421	2,181	1,744
P2	####	####	####	####	####	33	32	1,467	1,421	1,888	1,744
P8	P2	180	63.2	62.7	62.7	9	10	1,336	1,259	1,783	1,679
Q1	P8	0	0.0	0.0	0.0	####	####	####	####	####	####
Q2	P8	177	98.3	62.1	61.7	9	10	1,349	1,261	1,812	1,701
Q3	P8	0	0.0	0.0	0.0	####	####	####	####	####	####
Q4	P8	3	1.7	1.1	1.0	2	0	566	412	80	44
P7	P8	180	100.0	63.2	62.7	9	10	1,336	1,259	1,783	1,679
P1	P8	179	99.4	62.8	62.4	9	10	1,341	1,260	1,793	1,685
P4	P8	67	37.2	23.5	23.3	68	61	1,473	1,513	1,811	1,637

FACSDiva Version 6.1.3

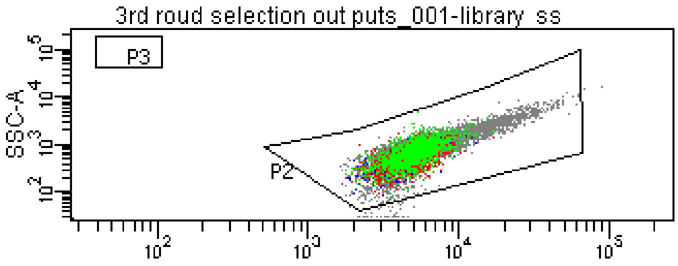

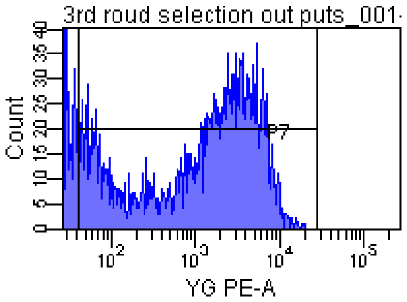

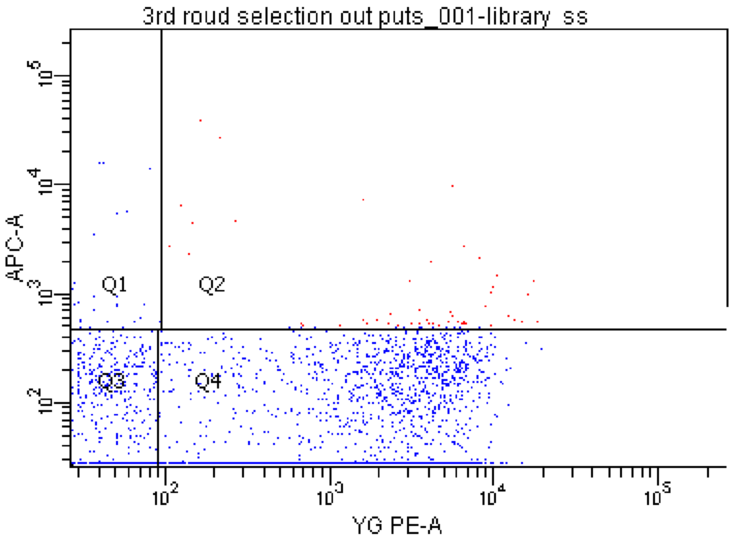

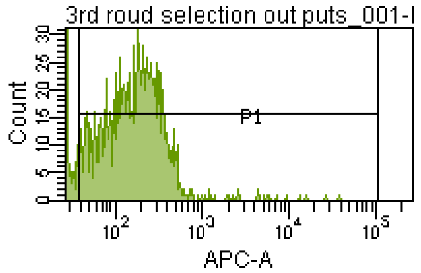

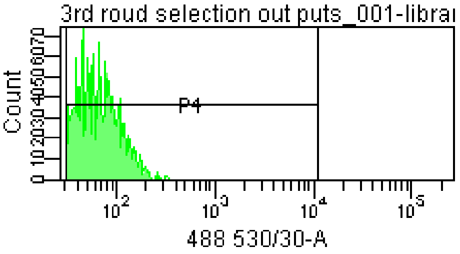

Experiment Name: Bio RBD2 selectons
Specimen Name: 3rd roud selection out puts_001
Tube Name: library ss
Record Date: May 23, 2020 11:45:21 AM
$OP: Nileena
GUID: f5e12ced-7d50-4d4f-acda-47fba6d47008

						488 5...	488 5...	PE-A	PE-A	APC-A	APC-A
Population	Parent...	#Events	%Parent	%Gran...	%Total	Mean	Median	Mean	Median	Mean	Median
All Events	####	####	####	####	####	60	39	1,499	831	299	79
P2	####	####	####	####	####	60	39	1,500	836	300	79
P8	P2	4,778	47.8	47.5	47.5	9	7	876	566	132	43
Q1	P8	102	2.1	1.0	1.0	-8	-11	479	364	2,807	857
Q2	P8	50	1.0	0.5	0.5	60	40	2,641	2,226	2,611	577
Q3	P8	2,749	57.5	27.5	27.3	5	3	421	362	42	33
Q4	P8	1,877	39.3	18.8	18.7	14	13	1,517	1,277	51	41
P7	P8	2,282	47.8	22.8	22.7	16	14	1,387	1,116	126	46
P1	P8	2,443	51.1	24.4	24.3	10	9	982	610	338	169
P4	P8	1,629	34.1	16.3	16.2	73	63	1,193	742	118	43

FACSDiva Version 6.1.3

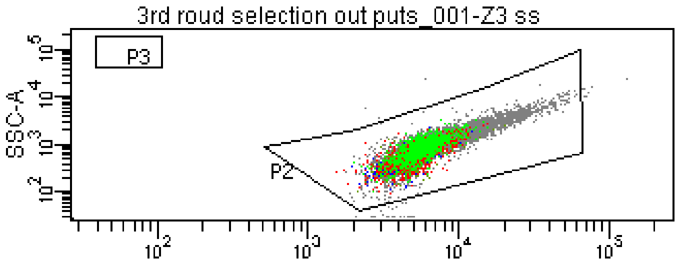

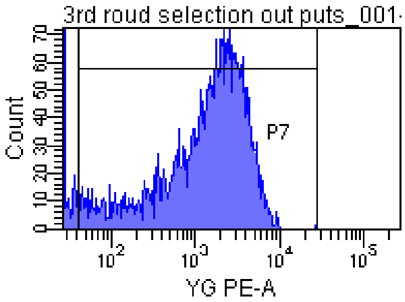

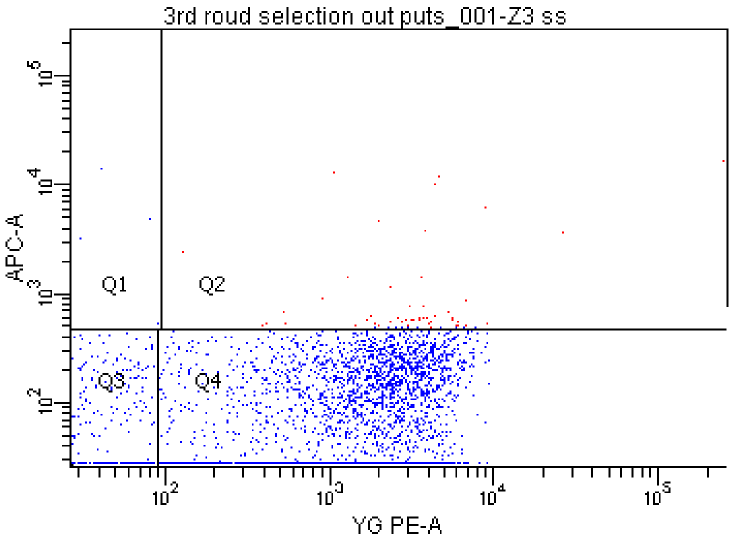

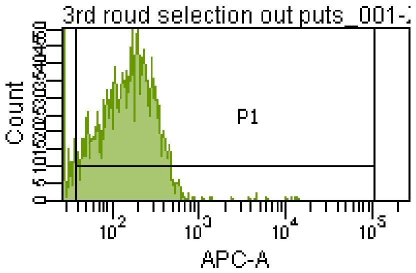

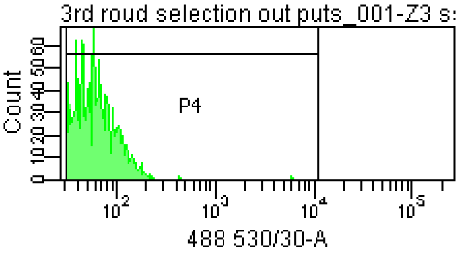

Experiment Name: Bio RBD2 selectons
Specimen Name: 3rd roud selection out puts_001
Tube Name: Z3 ss
Record Date: May 23, 2020 11:34:05 AM
$OP: Nileena
GUID: 3766f2ed-9846-4b14-ab3e-fe0828c33f35

Population	Parent...	#Events	%Parent	%Gran...	%Total	488 5... Mean	488 5... Median	PE-A Mean	PE-A Median	APC-A Mean	APC-A Median
All Events	####	####	####	####	####	56	31	1,852	1,325	305	66
P2	####	####	####	####	####	55	31	1,818	1,328	248	66
P8	P2	4,891	48.9	48.7	48.7	2	0	1,165	944	79	34
Q1	P8	15	0.3	0.2	0.1	-28	-13	588	638	7,202	4,570
Q2	P8	49	1.0	0.5	0.5	141	15	6,208	1,975	1,937	573
Q3	P8	1,346	27.5	13.5	13.4	-10	-8	500	444	12	2
Q4	P8	3,481	71.2	34.8	34.7	5	3	1,354	1,225	48	42
P7	P8	3,710	75.9	37.1	37.0	5	3	1,326	1,190	71	43
P1	P8	2,392	48.9	23.9	23.8	5	3	1,362	1,120	251	155
P4	P8	1,353	27.7	13.5	13.5	71	59	1,589	1,275	72	43

FACSDiva Version 6.1.3

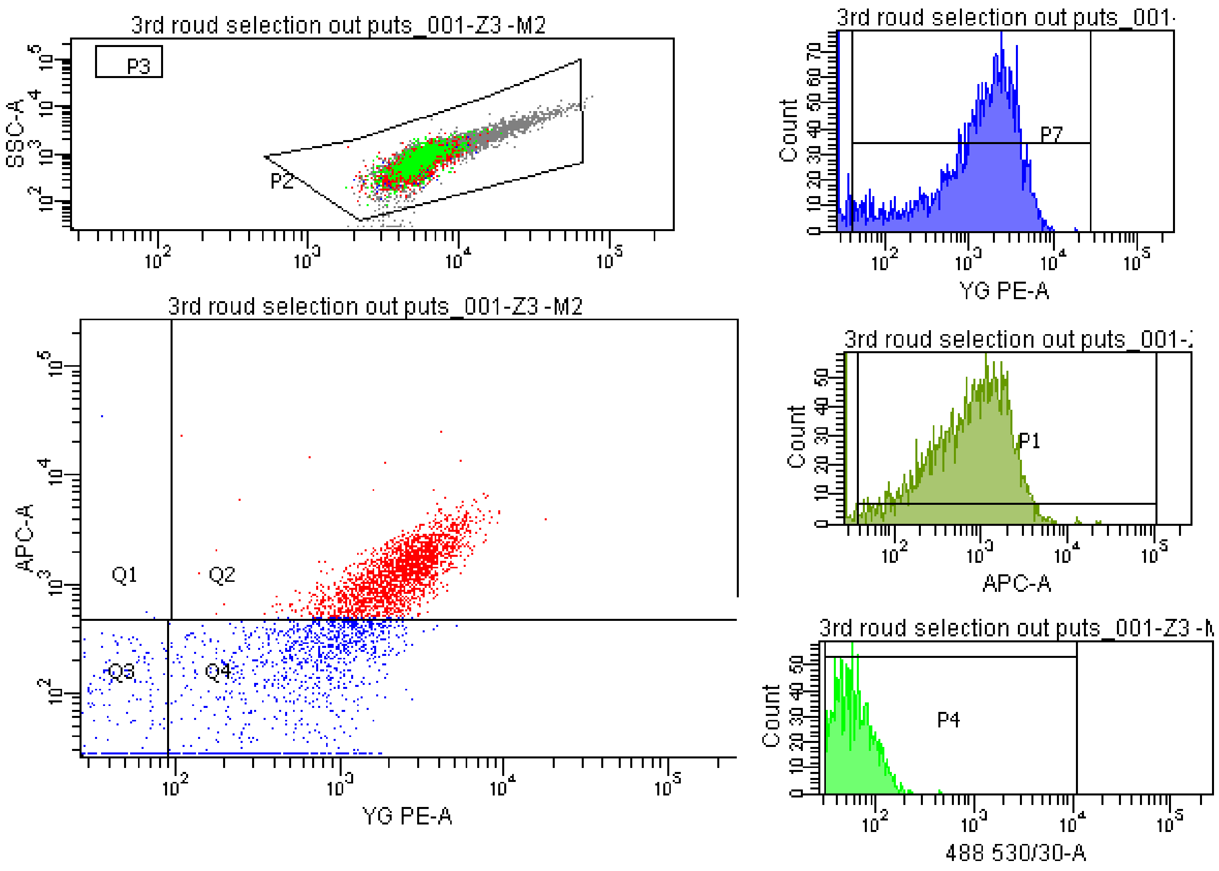

Experiment Name: Bio RBD2 selectons
Specimen Name: 3rd roud selection out puts_001
Tube Name: Z3 -M2
Record Date: May 23, 2020 11:32:00 AM
$OP: Nileena
GUID: 07bd6e0e-836e-4caa-a809-e73ccb4c8063

Population	Parent...	#Events	%Parent	%Gran...	%Total	488 5... Mean	488 5... Median	PE-A Mean	PE-A Median	APC-A Mean	APC-A Median
All Events	####	####	####	####	####	48	31	1,715	1,275	1,190	591
P2	####	####	####	####	####	48	31	1,712	1,281	1,184	593
P8	P2	4,694	47.0	46.8	46.8	-1	-2	1,072	909	746	390
Q1	P8	7	0.1	0.1	0.1	-26	-51	397	215	5,561	893
Q2	P8	2,152	45.8	21.5	21.4	7	7	1,602	1,495	1,500	1,239
Q3	P8	1,281	27.3	12.8	12.8	-12	-14	465	424	11	-3
Q4	P8	1,254	26.7	12.6	12.5	-4	-3	786	729	176	185
P7	P8	3,582	76.3	35.9	35.7	3	2	1,263	1,127	964	688
P1	P8	3,620	77.1	36.2	36.1	2	1	1,237	1,108	990	680
P4	P8	1,277	27.2	12.8	12.7	66	58	1,352	1,196	995	657

FACSDiva Version 6.1.3

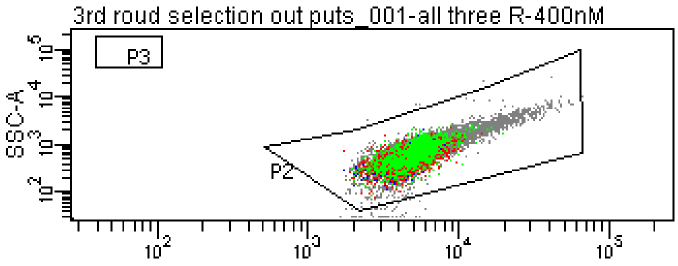

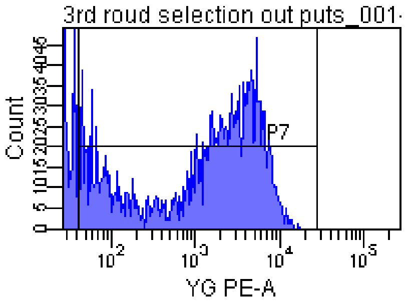

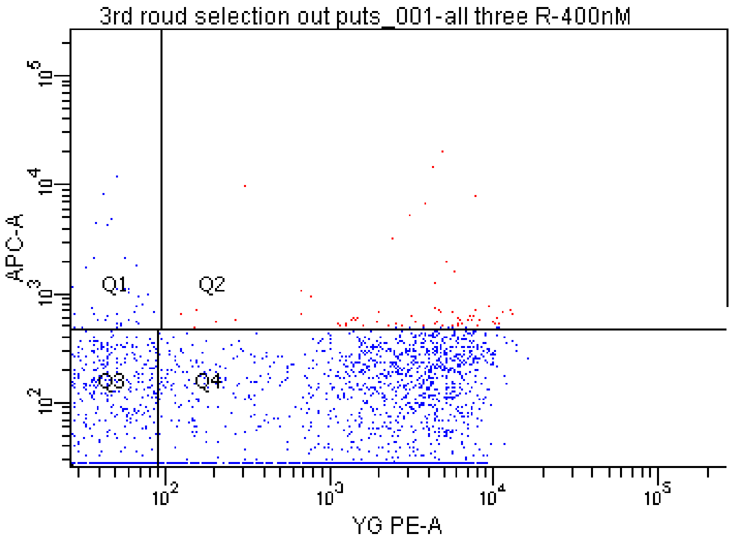

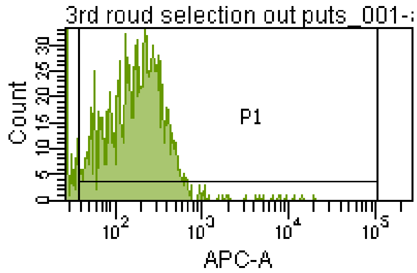

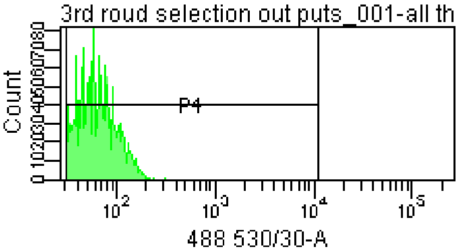

Experiment Name: Bio RBD2 selectons
Specimen Name: 3rd roud selection out puts_001
Tube Name: all three R-400nM
Record Date: May 23, 2020 11:35:22 AM
$OP: Nileena
GUID: e3452161-0753-4a0f-813b-c02563d5d80b

Population	Parent...	#Events	%Parent	%Gran...	%Total	488 5... Mean	488 5... Median	PE-A Mean	PE-A Median	APC-A Mean	APC-A Median
All Events	####	####	####	####	####	54	35	1,513	836	327	89
P2	####	####	####	####	####	55	35	1,511	838	293	89
P8	P2	4,967	49.7	49.4	49.4	6	4	893	570	138	53
Q1	P8	132	2.7	1.3	1.3	-8	-16	443	379	2,596	766
Q2	P8	65	1.3	0.7	0.6	30	25	2,276	2,073	1,568	565
Q3	P8	2,862	57.6	28.6	28.4	1	0	419	367	40	33
Q4	P8	1,908	38.4	19.1	19.0	13	13	1,589	1,349	67	59
P7	P8	2,329	46.9	23.3	23.2	14	13	1,438	1,172	125	66
P1	P8	2,648	53.3	26.5	26.3	7	5	1,016	639	337	172
P4	P8	1,561	31.4	15.6	15.5	69	61	1,211	832	124	65

FACSDiva Version 6.1.3

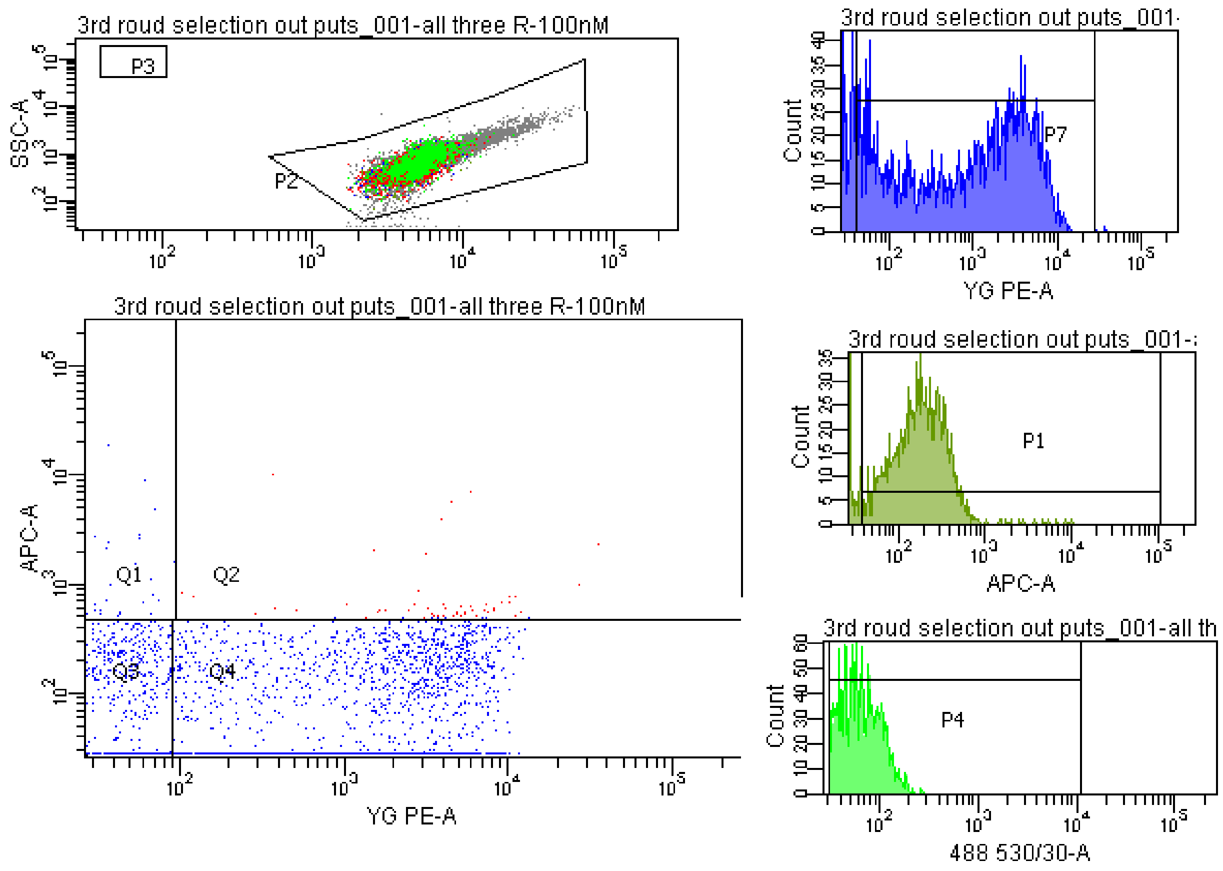

Experiment Name: Bio RBD2 selectons
Specimen Name: 3rd roud selection out puts_001
Tube Name: all three R-100nM
Record Date: May 23, 2020 11:37:00 AM
$OP: Nileena
GUID: 36b30d1b-6bf6-48a0-85d6-9526eeef1276

Population	Parent...	#Events	%Parent	%Gran...	%Total	488 5... Mean	488 5... Median	PE-A Mean	PE-A Median	APC-A Mean	APC-A Median
All Events	####	####	####	####	####	63	29	1,433	722	306	89
P2	####	####	####	####	####	64	31	1,438	723	306	89
P8	P2	5,050	50.5	50.2	50.2	3	2	790	510	118	60
Q1	P8	134	2.7	1.3	1.3	-11	-13	426	384	2,085	810
Q2	P8	51	1.0	0.5	0.5	29	15	2,738	2,166	1,125	563
Q3	P8	3,008	59.6	30.1	29.9	0	-1	421	369	49	42
Q4	P8	1,857	36.8	18.6	18.5	8	7	1,361	1,117	62	61
P7	P8	2,303	45.6	23.0	22.9	10	9	1,231	924	95	66
P1	P8	2,709	53.6	27.1	26.9	4	3	886	553	299	177
P4	P8	1,471	29.1	14.7	14.6	70	61	1,068	706	105	63

FACSDiva Version 6.1.3

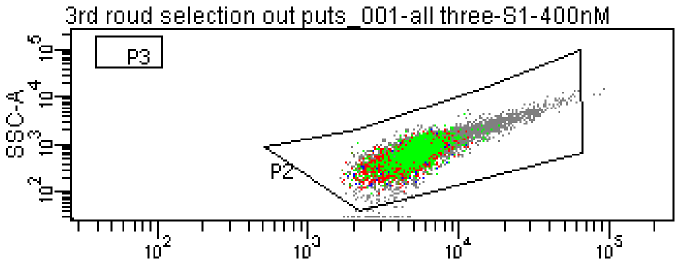

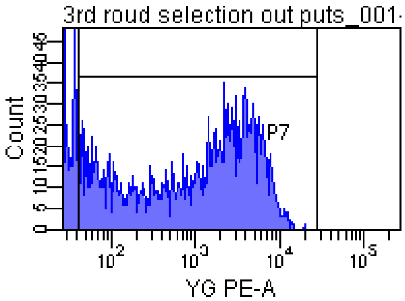

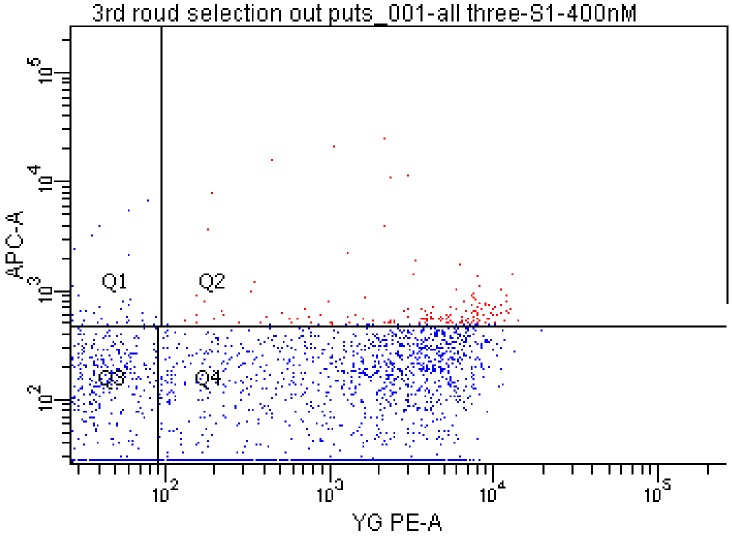

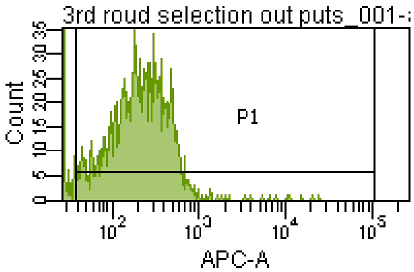

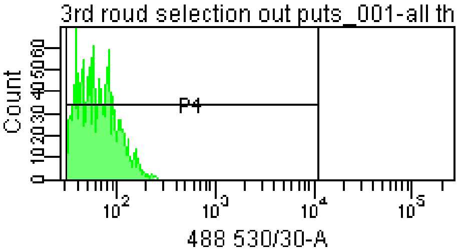

Experiment Name: Bio RBD2 selectons
Specimen Name: 3rd roud selection out puts_001
Tube Name: all three-S1-400nM
Record Date: May 23, 2020 11:48:04 AM
$OP: Nileena
GUID: 72ba149a-5546-494e-82cc-cc155c4bab0e

Population	Parent...	#Events	%Parent	%Gran...	%Total	488 5... Mean	488 5... Median	PE-A Mean	PE-A Median	APC-A Mean	APC-A Median
All Events	####	####	####	####	####	54	32	1,455	760	265	106
P2	####	####	####	####	####	54	33	1,453	763	263	106
P8	P2	4,908	49.1	48.8	48.8	4	1	814	522	142	70
Q1	P8	140	2.9	1.4	1.4	-15	-15	395	355	1,662	755
Q2	P8	144	2.9	1.4	1.4	32	27	2,472	2,327	1,278	563
Q3	P8	2,869	58.5	28.7	28.5	-1	-2	414	364	46	43
Q4	P8	1,755	35.8	17.5	17.4	10	8	1,364	1,119	85	82
P7	P8	2,214	45.1	22.1	22.0	12	9	1,302	980	166	97
P1	P8	2,757	56.2	27.6	27.4	5	3	952	579	328	196
P4	P8	1,464	29.8	14.6	14.5	70	61	1,134	741	131	83

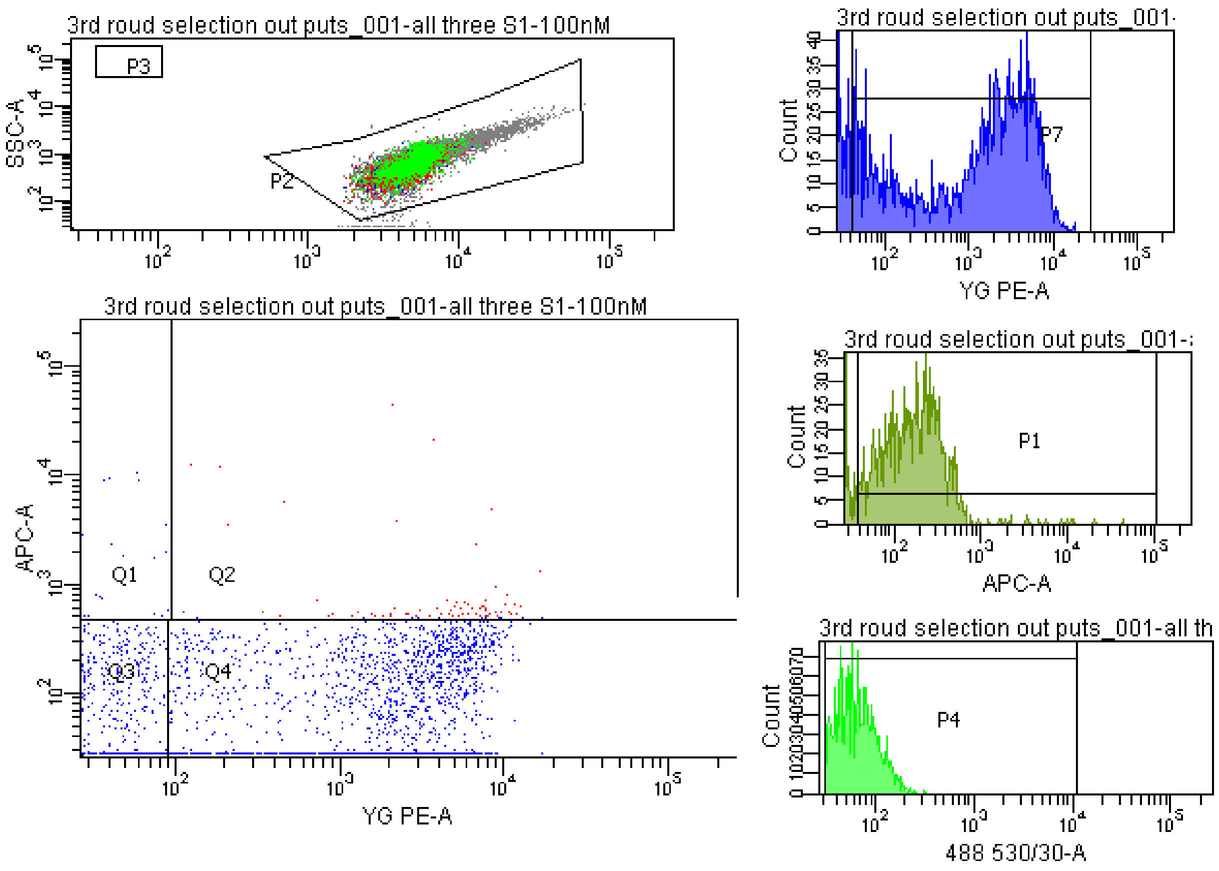

Experiment Name: Bio RBD2 selectons
Specimen Name: 3rd roud selection out puts_001
Tube Name: all three S1-100nM
Record Date: May 23, 2020 11:49:21 AM
$OP: Nileena
GUID: 15933682-a617-43df-a407-104f564edf76

Population	Parent...	#Events	%Parent	%Gran...	%Total	488 5... Mean	488 5... Median	PE-A Mean	PE-A Median	APC-A Mean	APC-A Median
All Events	####	####	####	####	####	57	37	1,440	799	253	88
P2	####	####	####	####	####	57	37	1,442	806	217	88
P8	P2	4,845	48.4	48.0	48.0	9	8	868	556	129	55
Q1	P8	115	2.4	1.1	1.1	-6	-5	431	397	1,919	809
Q2	P8	69	1.4	0.7	0.7	37	40	2,546	2,456	2,038	544
Q3	P8	2,771	57.2	27.7	27.5	4	3	410	362	44	30
Q4	P8	1,890	39.0	18.9	18.7	17	16	1,503	1,240	75	64
P7	P8	2,284	47.1	22.8	22.6	18	17	1,392	1,107	143	67
P1	P8	2,605	53.8	26.0	25.8	11	11	1,004	631	306	170
P4	P8	1,626	33.6	16.3	16.1	71	62	1,169	776	115	66

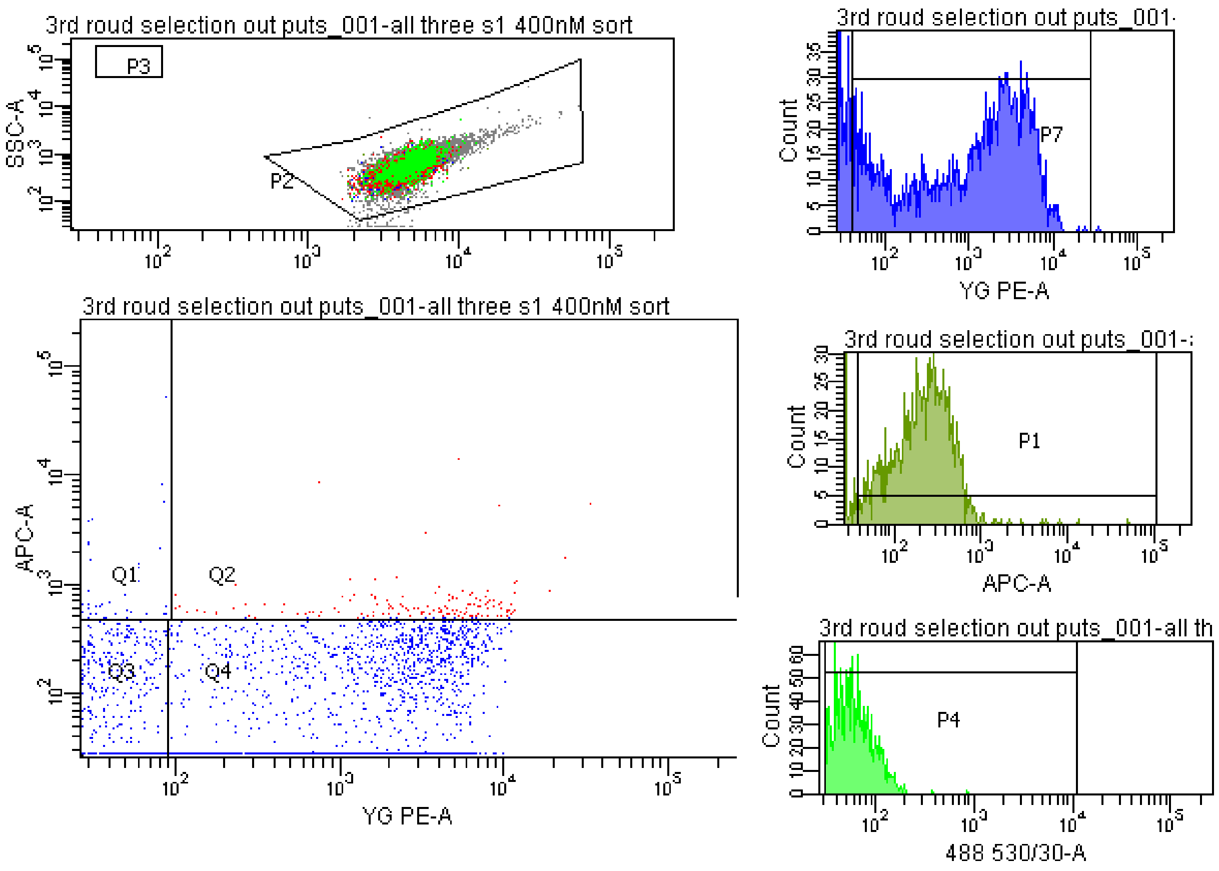

Experiment Name: Bio RBD2 selectons
Specimen Name: 3rd roud selection out puts_001
Tube Name: all three s1 400nM sort
Record Date: May 23, 2020 12:38:57 PM
$OP: Nileena
GUID: 3ef16f0f-cee1-4e6d-ada0-5b7688527d8d

Population	Parent...	#Events	%Parent	%Gran...	%Total	488 5... Mean	488 5... Median	PE-A Mean	PE-A Median	APC-A Mean	APC-A Median
All Events	####	####	####	####	####	39	22	1,020	572	350	81
P2	####	####	####	####	####	39	22	1,024	577	352	82
P8	P2	5,091	50.9	50.5	50.5	-4	-4	716	437	145	59
Q1	P8	191	3.8	1.9	1.9	-18	-20	380	326	2,239	760
Q2	P8	137	2.7	1.4	1.4	21	9	2,341	2,024	850	571
Q3	P8	3,049	59.9	30.5	30.2	-6	-5	346	305	28	25
Q4	P8	1,714	33.7	17.1	17.0	0	1	1,282	1,067	64	68
P7	P8	2,159	42.4	21.6	21.4	3	2	1,218	933	140	82
P1	P8	2,719	53.4	27.2	27.0	-2	-3	842	487	381	212
P4	P8	1,289	25.3	12.9	12.8	67	58	1,001	607	127	72

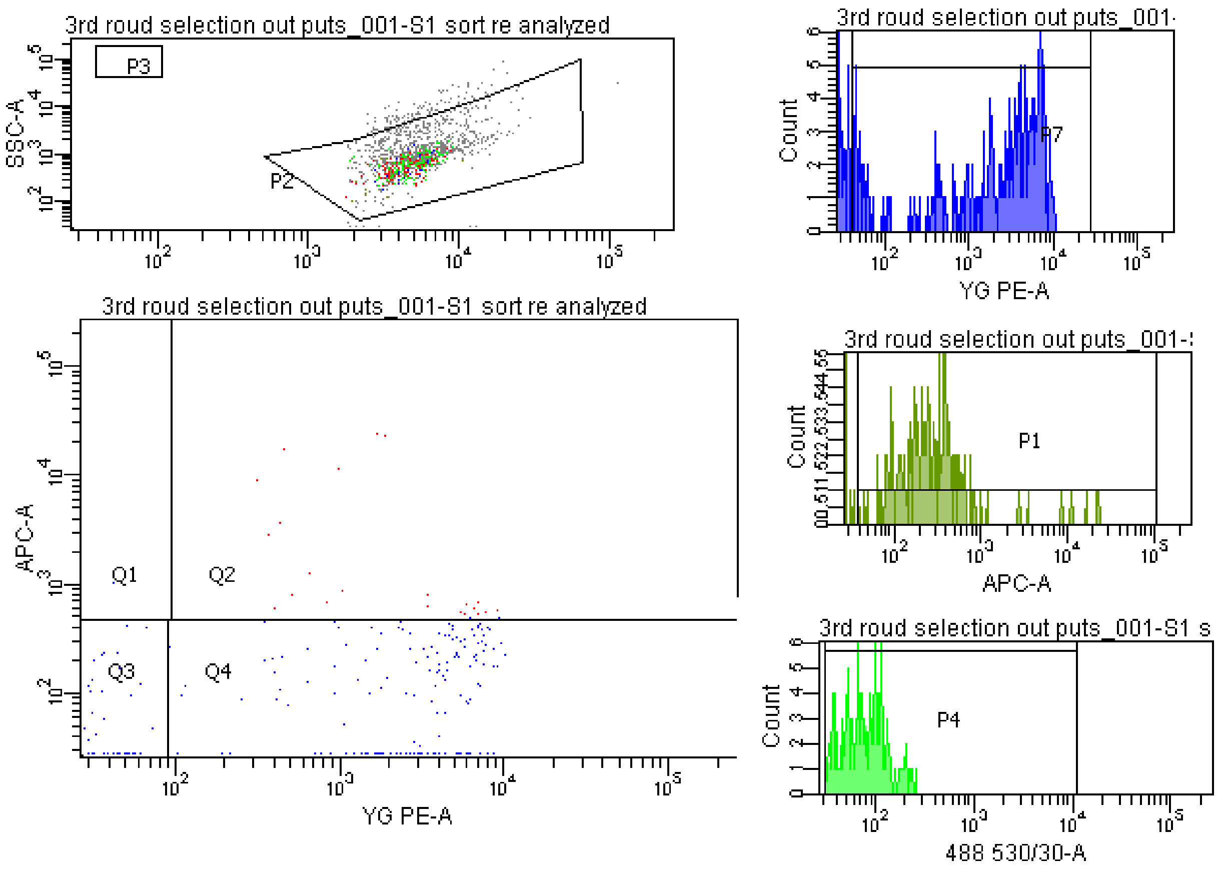

Experiment Name: Bio RBD2 selectons
Specimen Name: 3rd roud selection out puts_001
Tube Name: S1 sort re analyzed
Record Date: May 23, 2020 12:43:26 PM
$OP: Nileena
GUID: d354d0b0-62c1-4c52-ae94-3af40f17c612

Population	Parent...	#Events	%Parent	%Gran...	%Total	488 5... Mean	488 5... Median	PE-A Mean	PE-A Median	APC-A Mean	APC-A Median
All Events	####	####	####	####	####	102	55	2,844	2,021	456	196
P2	####	####	####	####	####	81	55	2,257	1,836	430	191
P8	P2	327	29.0	26.7	26.7	10	5	1,153	740	397	109
Q1	P8	5	1.5	0.4	0.4	36	87	453	407	715	597
Q2	P8	22	6.7	2.0	1.8	24	33	1,703	1,197	4,410	713
Q3	P8	151	46.2	13.4	12.3	1	-1	422	377	62	55
Q4	P8	149	45.6	13.2	12.2	17	10	1,836	1,728	133	133
P7	P8	189	57.8	16.8	15.4	16	14	1,695	1,528	629	168
P1	P8	212	64.8	18.8	17.3	14	6	1,331	804	653	211
P4	P8	116	35.5	10.3	9.5	88	76	1,379	903	476	154

FACSDiva Version 6.1.3

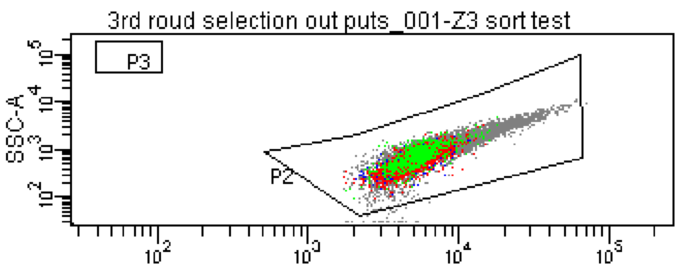

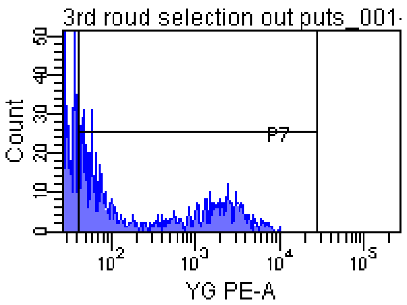

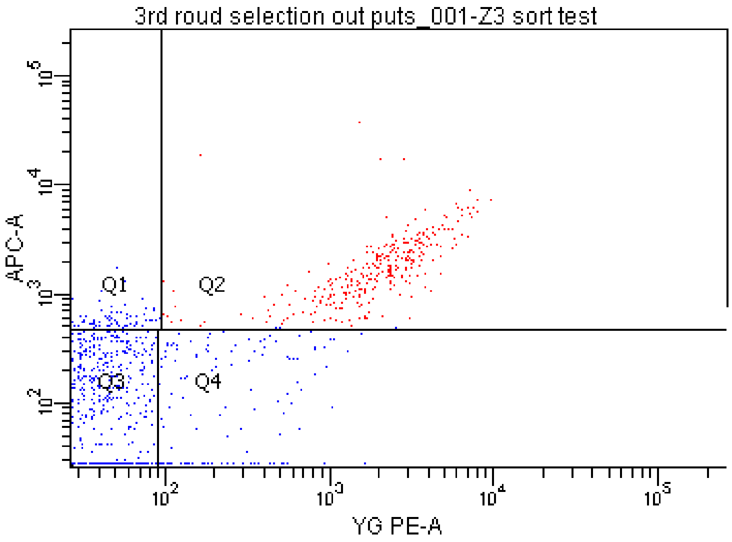

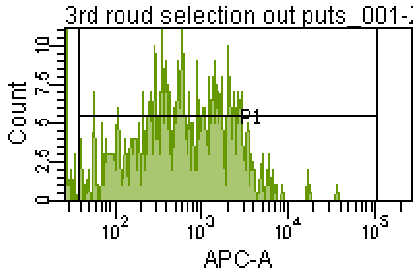

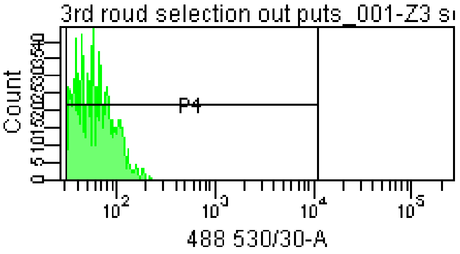

Experiment Name: Bio RBD2 selectons
Specimen Name: 3rd roud selection out puts_001
Tube Name: Z3 sort test
Record Date: May 23, 2020 11:14:44 AM
$OP: Nileena
GUID: b036c84b-ed10-4cb3-93a0-5f01c56abe42

Population	Parent...	#Events	%Parent	%Gran...	%Total	488 5... Mean	488 5... Median	PE-A Mean	PE-A Median	APC-A Mean	APC-A Median
All Events	####	####	####	####	####	43	20	992	691	464	209
P2	####	####	####	####	####	43	21	996	694	466	209
P8	P2	4,269	42.7	42.5	42.5	-12	-12	562	456	276	128
Q1	P8	366	8.6	3.7	3.6	-19	-15	606	523	639	595
Q2	P8	296	6.9	3.0	2.9	11	11	1,557	1,376	2,212	1,585
Q3	P8	3,486	81.7	34.9	34.7	-14	-14	472	421	78	78
Q4	P8	121	2.8	1.2	1.2	12	3	593	533	150	149
P7	P8	738	17.3	7.4	7.3	13	11	963	714	984	383
P1	P8	2,743	64.3	27.4	27.3	-13	-14	624	490	486	257
P4	P8	881	20.6	8.8	8.8	66	58	754	580	421	136

FACSDiva Version 6.1.3

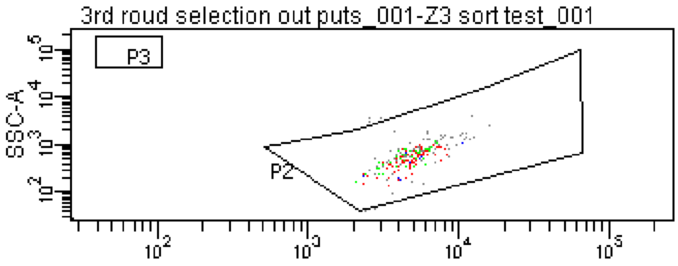

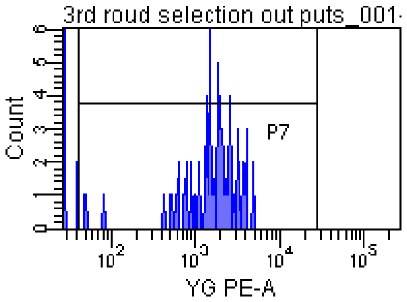

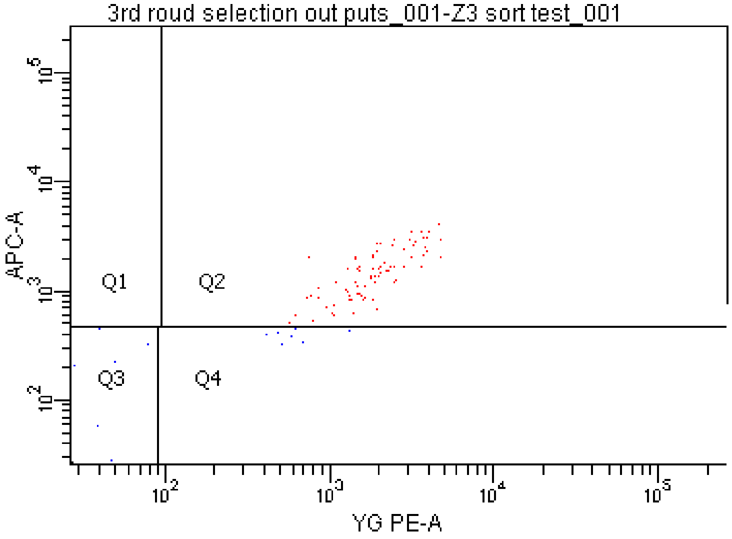

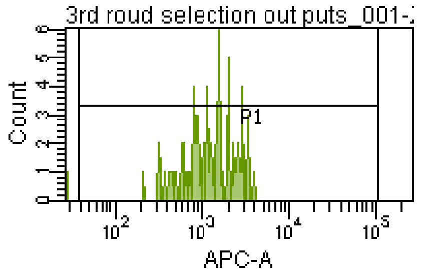

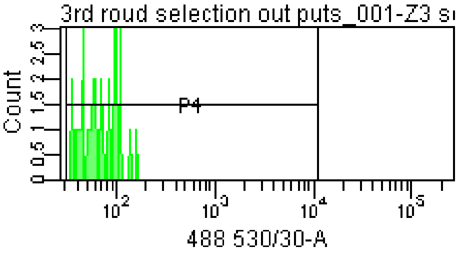

Experiment Name: Bio RBD2 selectons
Specimen Name: 3rd roud selection out puts_001
Tube Name: Z3 sort test_001
Record Date: May 23, 2020 11:17:43 AM
$OP: Nileena
GUID: bce2d329-41df-4be3-9296-52b048fa7acd

Population	Parent...	#Events	%Parent	%Gran...	%Total	488 5... Mean	488 5... Median	PE-A Mean	PE-A Median	APC-A Mean	APC-A Median
All Events	####	####	####	####	####	28	15	1,049	915	1,266	837
P2	####	####	####	####	####	29	15	1,026	915	1,083	844
P8	P2	122	60.1	58.7	58.7	4	0	913	875	1,071	854
Q1	P8	1	0.8	0.5	0.5	-12	-12	468	468	482	482
Q2	P8	78	63.9	38.4	37.5	4	1	1,182	1,099	1,585	1,494
Q3	P8	35	28.7	17.2	16.8	-1	0	408	355	100	53
Q4	P8	8	6.6	3.9	3.8	29	2	564	543	382	386
P7	P8	89	73.0	43.8	42.8	7	3	1,103	1,004	1,428	1,277
P1	P8	106	86.9	52.2	51.0	6	3	993	915	1,241	1,044
P4	P8	36	29.5	17.7	17.3	73	66	918	896	1,063	914

FACSDiva Version 6.1.3

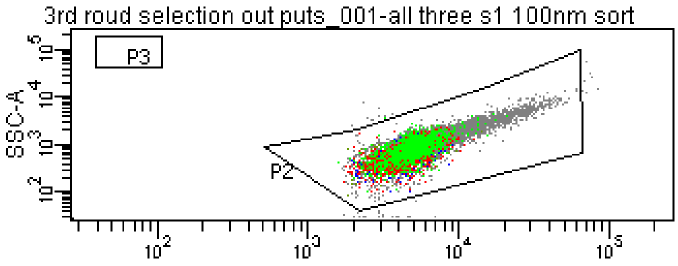

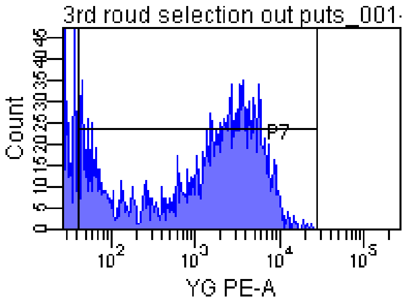

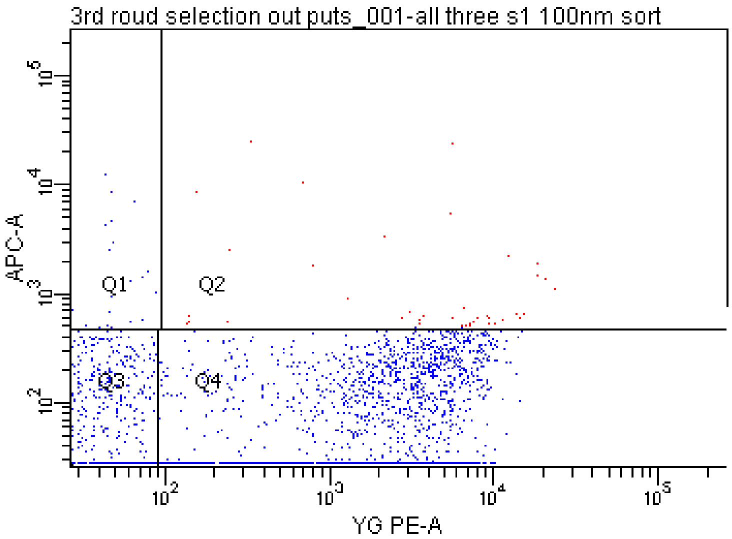

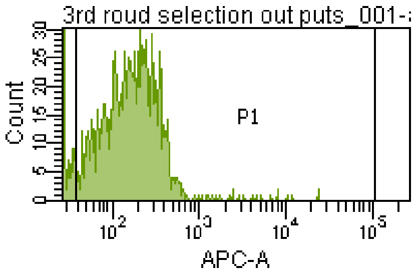

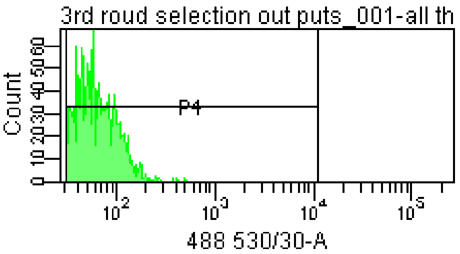

Experiment Name: Bio RBD2 selectons
Specimen Name: 3rd roud selection out puts_001
Tube Name: all three s1 100nm sort
Record Date: May 23, 2020 1:27:44 PM
$OP: Nileena
GUID: 569210b1-8ca7-40e8-a9e4-1cf4f260ed66

Population	Parent...	#Events	%Parent	%Gran...	%Total	488 5... Mean	488 5... Median	PE-A Mean	PE-A Median	APC-A Mean	APC-A Median
All Events	####	####	####	####	####	70	35	1,795	959	432	91
P2	####	####	####	####	####	60	35	1,719	959	312	90
P8	P2	4,689	46.9	46.7	46.7	6	4	929	626	135	54
Q1	P8	93	2.0	0.9	0.9	-10	-12	518	395	2,709	1,245
Q2	P8	39	0.8	0.4	0.4	86	50	3,573	3,033	2,589	602
Q3	P8	2,798	59.7	28.0	27.9	2	1	487	416	52	38
Q4	P8	1,759	37.5	17.6	17.5	11	10	1,596	1,359	76	62
P7	P8	2,118	45.2	21.2	21.1	13	11	1,480	1,212	142	65
P1	P8	2,504	53.4	25.0	25.0	8	7	1,088	706	304	157
P4	P8	1,414	30.2	14.1	14.1	69	59	1,296	868	159	72

FACSDiva Version 6.1.3

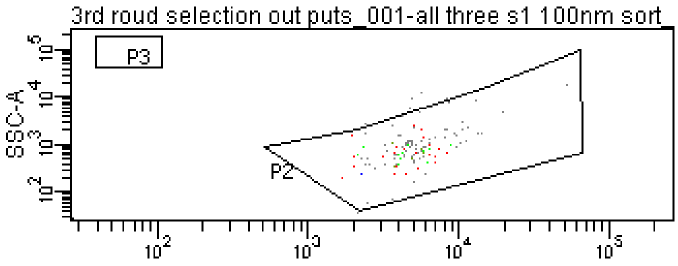

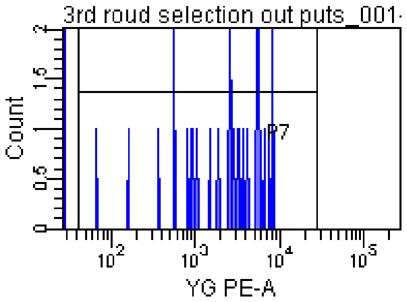

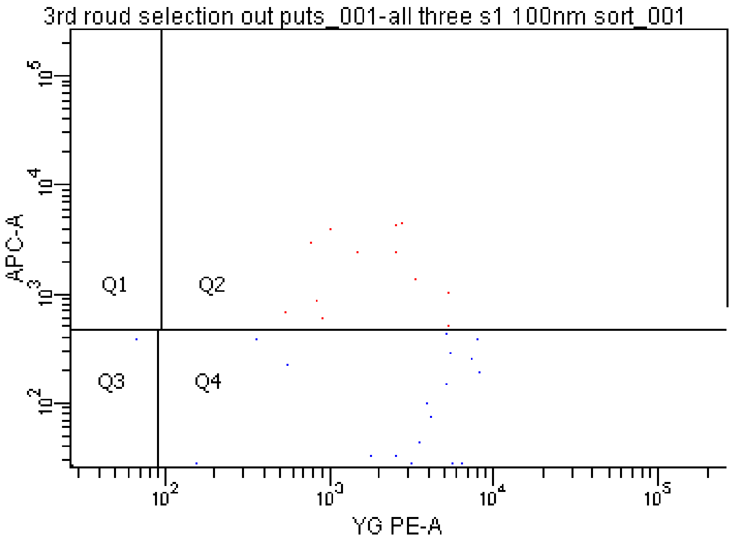

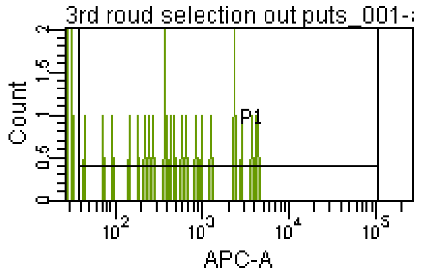

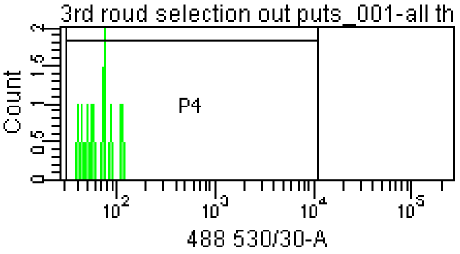

Experiment Name: Bio RBD2 selectons
Specimen Name: 3rd roud selection out puts_001
Tube Name: all three s1 100nm sort_001
Record Date: May 23, 2020 1:26:31 PM
$OP: Nileena
GUID: 559d36df-5b61-4410-b0c5-9b0f91390388

Population	Parent...	#Events	%Parent	%Gran...	%Total	488 5... Mean	488 5... Median	PE-A Mean	PE-A Median	APC-A Mean	APC-A Median
All Events	####	####	####	####	####	65	49	2,291	1,680	4,755	246
P2	####	####	####	####	####	68	54	2,189	1,658	4,030	243
P8	P2	40	34.8	33.3	33.3	10	3	1,463	1,281	733	257
Q1	P8	2	5.0	1.7	1.7	-18	-18	1,249	1,249	601	601
Q2	P8	12	30.0	10.4	10.0	15	9	1,215	949	2,045	1,801
Q3	P8	9	22.5	7.8	7.5	-8	-15	691	322	159	179
Q4	P8	17	42.5	14.8	14.2	18	3	2,072	2,393	126	92
P7	P8	30	75.0	26.1	25.0	16	5	1,673	1,581	902	367
P1	P8	32	80.0	27.8	26.7	13	5	1,490	1,281	929	375
P4	P8	11	27.5	9.6	9.2	70	71	1,886	1,851	928	244

FACSDiva Version 6.1.3

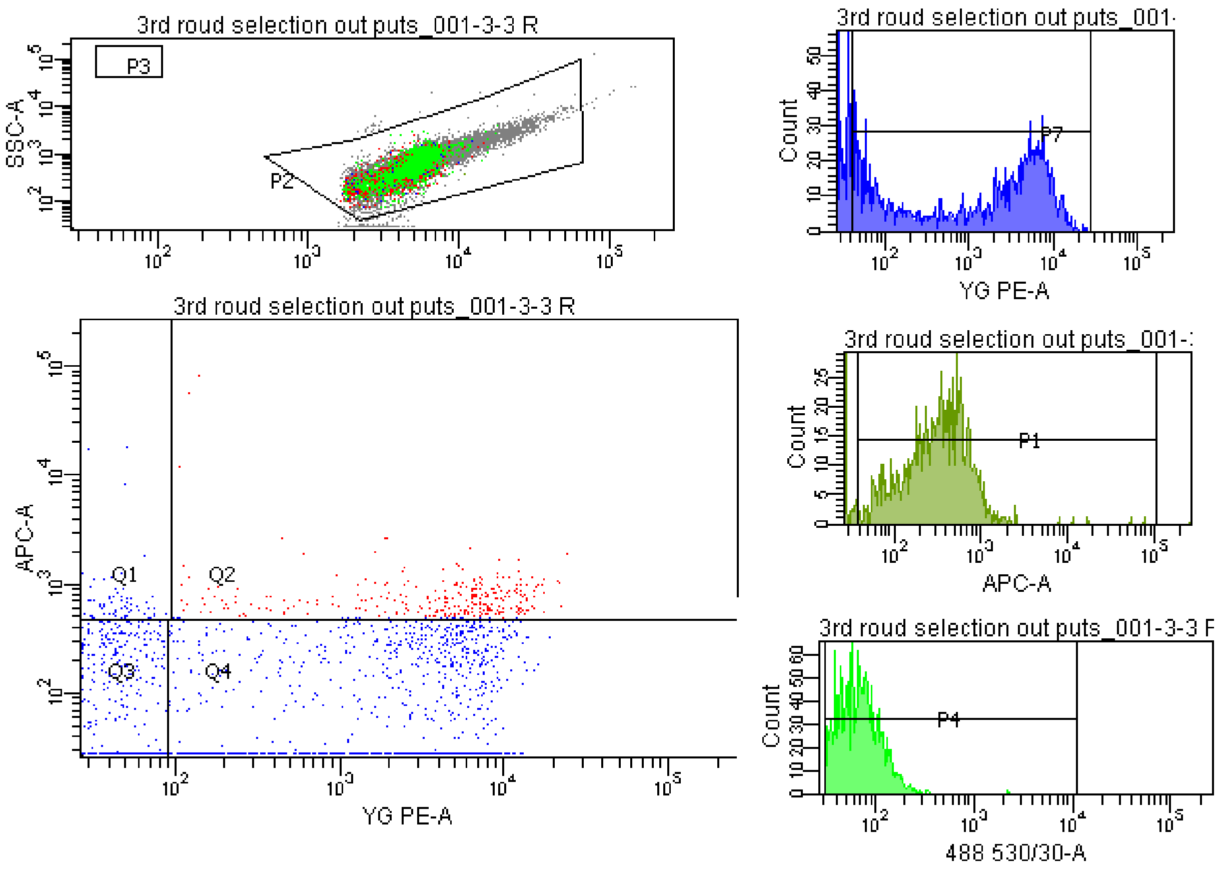

Experiment Name: Bio RBD2 selectons
Specimen Name: 3rd roud selection out puts_001
Tube Name: 3-3 R
Record Date: May 23, 2020 1:29:07 PM
$OP: Nileena
GUID: f42ab192-eda0-47a5-a9c9-c549933774f0

Population	Parent...	#Events	%Parent	%Gran...	%Total	488 5... Mean	488 5... Median	PE-A Mean	PE-A Median	APC-A Mean	APC-A Median
All Events	####	####	####	####	####	105	50	2,001	763	907	169
P2	####	####	####	####	####	82	51	1,944	763	390	167
P8	P2	4,314	42.9	42.0	42.0	13	10	881	481	252	81
Q1	P8	367	8.5	3.7	3.6	4	3	431	367	1,215	627
Q2	P8	286	6.6	2.8	2.8	48	42	2,820	2,525	2,169	683
Q3	P8	2,632	61.0	26.2	25.7	6	2	400	353	-10	2
Q4	P8	1,029	23.9	10.2	10.0	27	26	1,732	1,470	46	69
P7	P8	1,659	38.5	16.5	16.2	29	28	1,653	1,185	429	142
P1	P8	2,394	55.5	23.8	23.3	14	9	1,042	520	504	299
P4	P8	1,567	36.3	15.6	15.3	76	66	1,303	699	389	87

FACSDiva Version 6.1.3

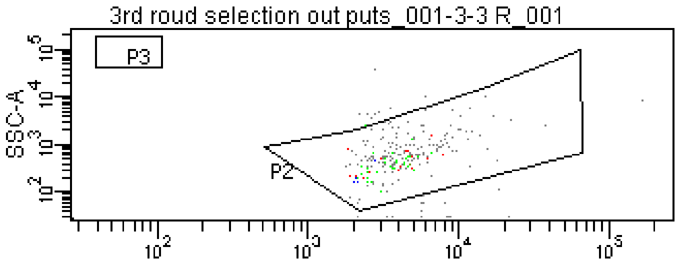

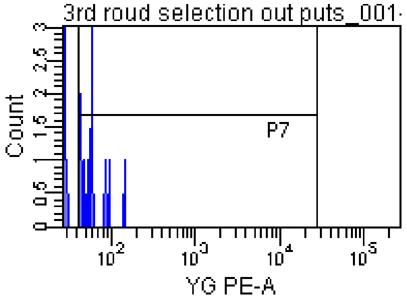

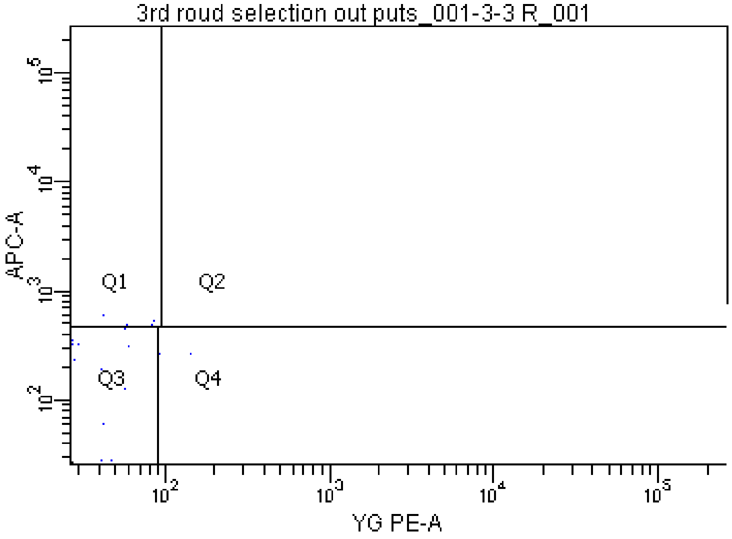

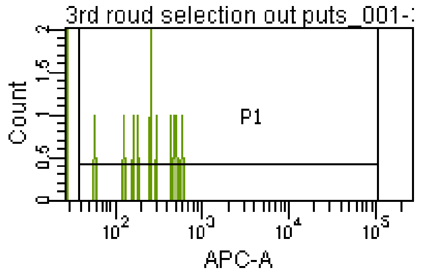

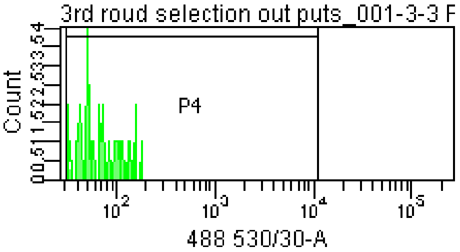

Experiment Name: Bio RBD2 selectons
Specimen Name: 3rd roud selection out puts_001
Tube Name: 3-3 R_001
Record Date: May 23, 2020 1:42:45 PM
$OP: Nileena
GUID: 2037cbb1-be5a-498f-ad4b-d54ee7b84fac

Population	Parent...	#Events	%Parent	%Gran...	%Total	488 5... Mean	488 5... Median	PE-A Mean	PE-A Median	APC-A Mean	APC-A Median
All Events	####	####	####	####	####	86	68	2,325	2,334	1,808	193
P2	####	####	####	####	####	86	72	2,586	2,688	856	208
P8	P2	53	25.1	22.6	22.6	34	40	2,527	2,461	1,495	181
Q1	P8	7	13.2	3.3	3.0	31	49	2,910	3,965	10,344	572
Q2	P8	0	0.0	0.0	0.0	####	####	####	####	####	####
Q3	P8	44	83.0	20.9	18.7	34	40	2,292	2,373	143	154
Q4	P8	2	3.8	0.9	0.9	47	47	6,345	6,345	254	254
P7	P8	14	26.4	6.6	6.0	56	57	3,610	3,559	262	254
P1	P8	43	81.1	20.4	18.3	36	41	2,771	2,635	1,863	237
P4	P8	31	58.5	14.7	13.2	76	68	3,048	3,065	1,752	222

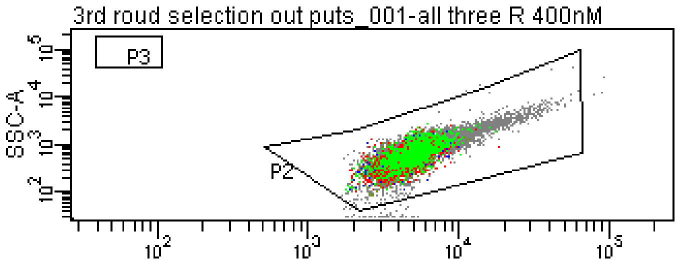

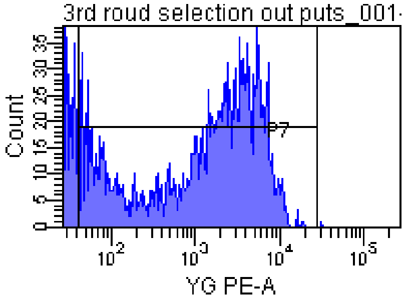

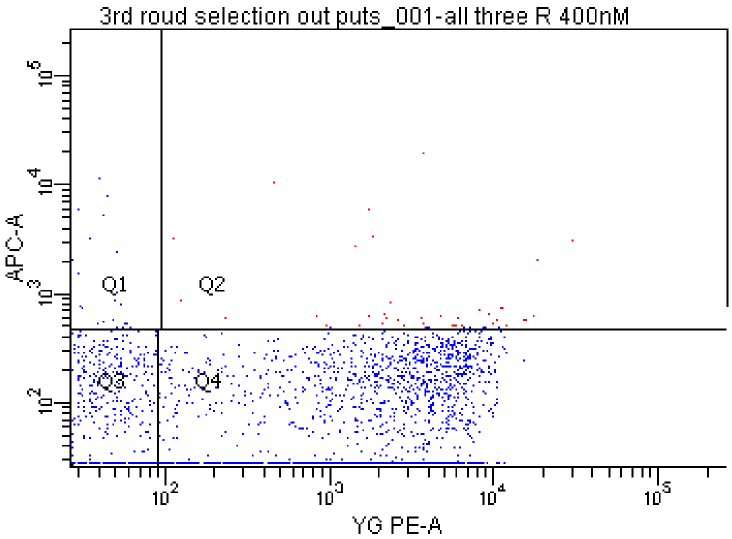

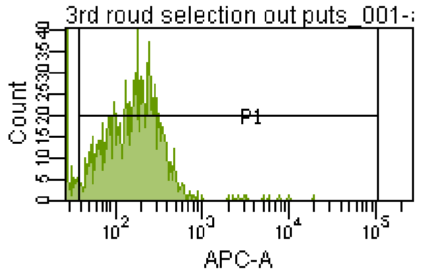

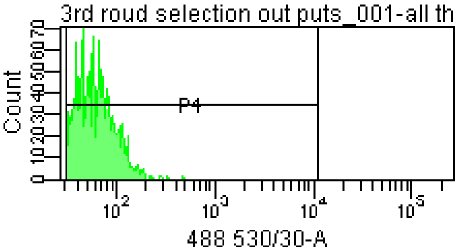

Experiment Name: Bio RBD2 selectons
Specimen Name: 3rd roud selection out puts_001
Tube Name: all three R 400nM
Record Date: May 23, 2020 2:04:21 PM
$OP: Nileena
GUID: 023f424d-b164-48d9-a56d-4ef672d3cf4e

Population	Parent...	#Events	%Parent	%Gran...	%Total	488 5... Mean	488 5... Median	PE-A Mean	PE-A Median	APC-A Mean	APC-A Median
All Events	####	####	####	####	####	52	33	1,482	835	386	85
P2	####	####	####	####	####	52	33	1,453	839	341	86
P8	P2	4,743	46.3	46.0	46.0	5	3	880	579	104	57
Q1	P8	106	2.2	1.0	1.0	-21	-26	442	438	1,629	850
Q2	P8	40	0.8	0.4	0.4	35	14	2,941	2,118	1,674	580
Q3	P8	2,823	59.5	27.5	27.4	0	-1	450	399	48	36
Q4	P8	1,774	37.4	17.3	17.2	12	12	1,544	1,321	68	65
P7	P8	2,118	44.7	20.7	20.5	12	11	1,417	1,135	103	68
P1	P8	2,538	53.5	24.8	24.6	7	7	1,019	638	263	168
P4	P8	1,445	30.5	14.1	14.0	67	59	1,240	849	104	75

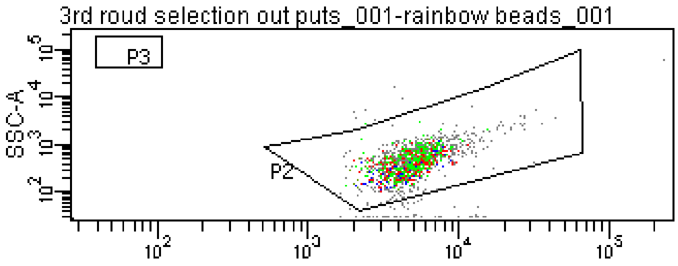

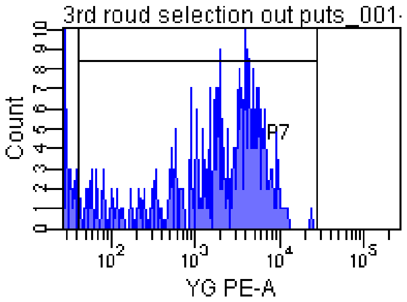

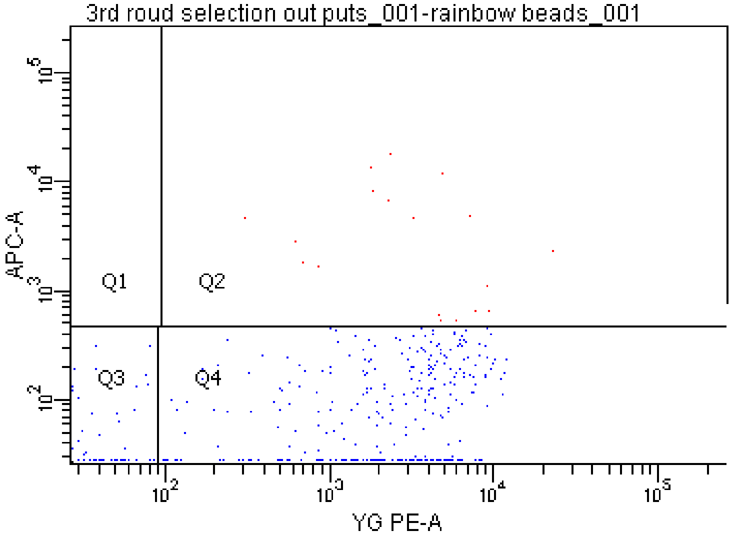

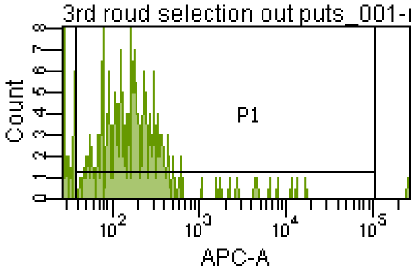

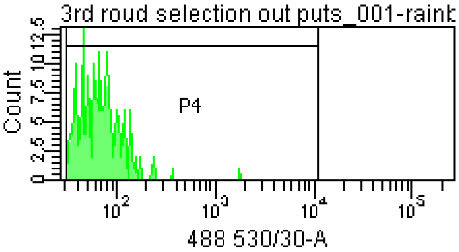

Experiment Name: Bio RBD2 selectons
Specimen Name: 3rd roud selection out puts_001
Tube Name: rainbow beads_001
Record Date: May 23, 2020 3:12:44 PM
$OP: Nileena
GUID: fe6145f0-5bfe-4ebe-a402-7c4c059bf211

Population	Parent...	#Events	%Parent	%Gran...	%Total	488 5... Mean	488 5... Median	PE-A Mean	PE-A Median	APC-A Mean	APC-A Median
All Events	####	####	####	####	####	47	27	1,137	477	1,150	77
P2	####	####	####	####	####	45	29	1,112	502	558	80
P8	P2	694	52.9	50.3	50.3	13	8	875	400	632	58
Q1	P8	6	0.9	0.5	0.4	0	8	779	429	9,167	1,376
Q2	P8	19	2.7	1.4	1.4	120	34	2,345	1,734	18,019	2,768
Q3	P8	366	52.7	27.9	26.5	-2	-1	219	208	44	33
Q4	P8	303	43.7	23.1	22.0	25	24	1,577	1,451	83	78
P7	P8	346	49.9	26.4	25.1	29	22	1,527	1,343	1,064	86
P1	P8	389	56.1	29.7	28.2	14	15	1,068	521	506	156
P4	P8	239	34.4	18.2	17.3	80	64	1,311	766	1,556	90

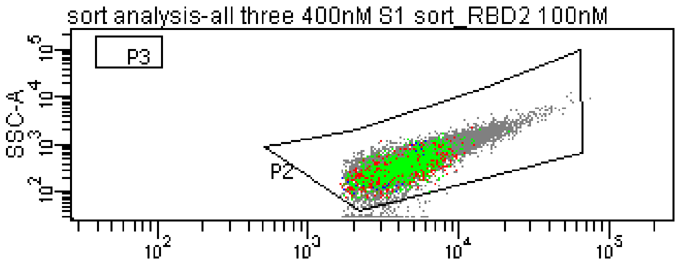

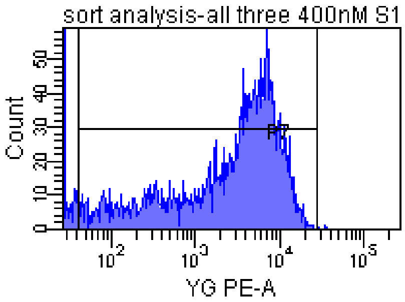

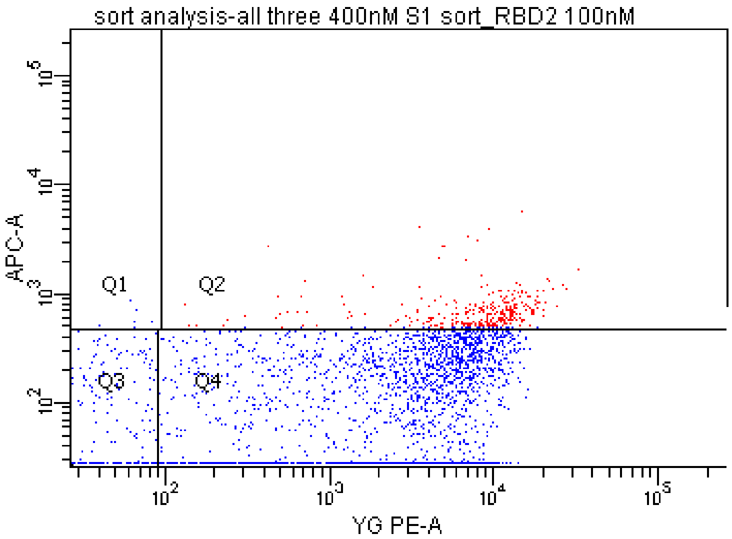

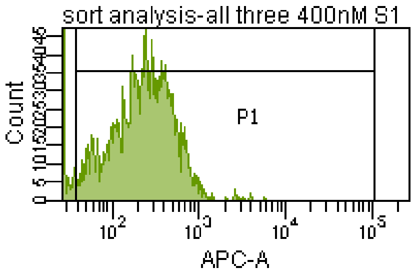

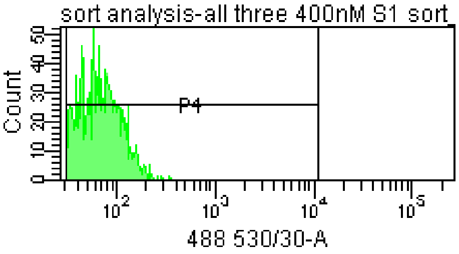

Experiment Name:	Bio RBD2 selectons
Specimen Name:	sort analysis
Tube Name:	all three 400nM S1 sort_RBD2 100nM
Record Date:	May 26, 2020 2:57:07 PM
$OP:	Nileena
GUID:	7517878d-a180-413d-9d19-e96a33fed2e1

Population	Parent...	#Events	%Parent	%Gran...	%Total	488 5... Mean	488 5... Median	PE-A Mean	PE-A Median	APC-A Mean	APC-A Median
All Events	####	####	####	####	####	77	54	2,533	1,908	274	185
P2	####	####	####	####	####	81	58	2,583	1,959	279	189
P8	P2	3,214	32.3	31.2	31.2	12	9	1,721	1,288	170	129
Q1	P8	10	0.3	0.1	0.1	22	17	558	404	828	640
Q2	P8	310	9.6	3.1	3.0	53	51	4,038	3,937	746	625
Q3	P8	699	21.7	7.0	6.8	-13	-10	309	258	33	30
Q4	P8	2,195	68.3	22.1	21.3	13	11	1,848	1,655	129	129
P7	P8	2,629	81.8	26.4	25.5	17	14	2,022	1,722	198	159
P1	P8	2,141	66.6	21.5	20.8	15	13	2,093	1,807	290	224
P4	P8	1,154	35.9	11.6	11.2	75	66	2,542	2,303	231	182

FACSDiva Version 6.1.3

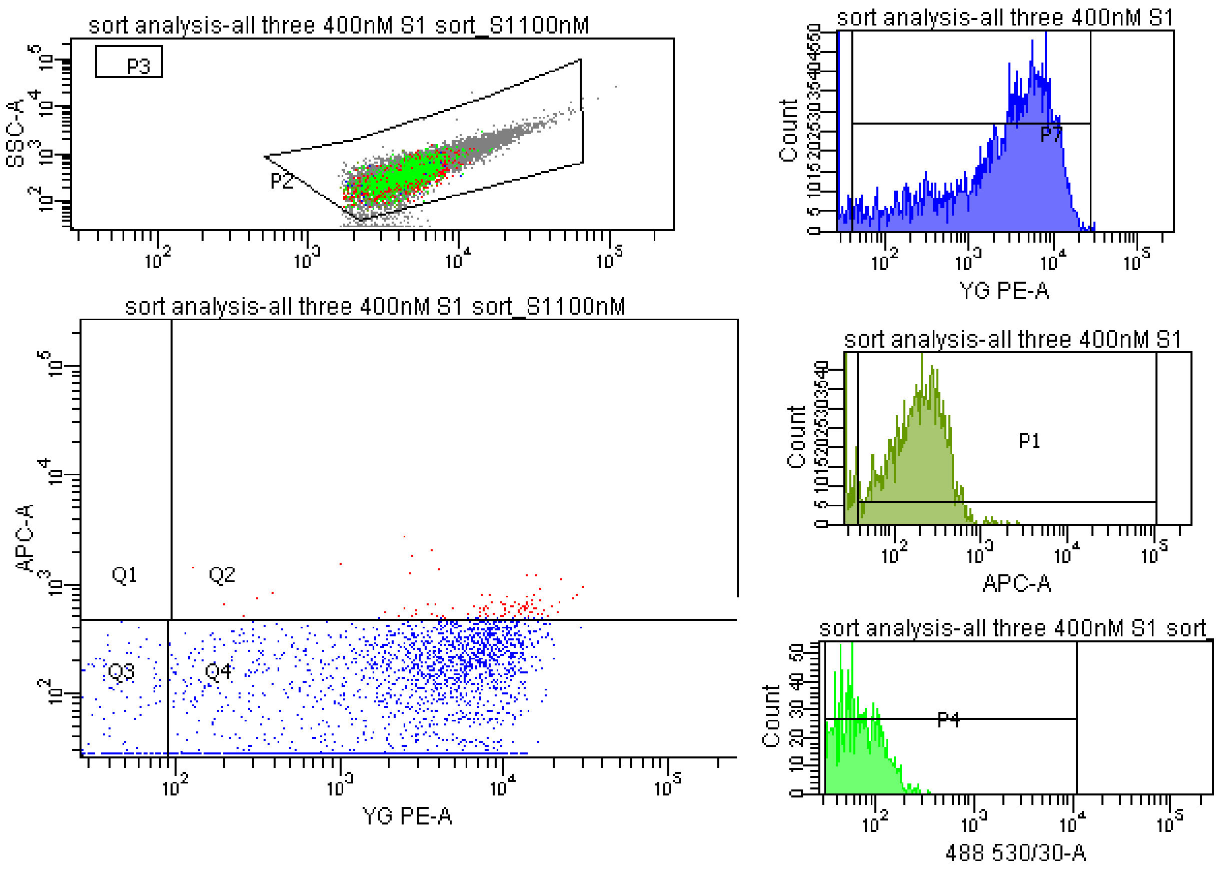

Experiment Name: Bio RBD2 selectons
Specimen Name: sort analysis
Tube Name: all three 400nM S1 sort_S1100nM
Record Date: May 26, 2020 2:59:09 PM
$OP: Nileena
GUID: 5aba3224-3d5f-4122-9a2f-9cc759658b9f

Population	Parent...	#Events	%Parent	%Gran...	%Total	488 5... Mean	488 5... Median	PE-A Mean	PE-A Median	APC-A Mean	APC-A Median
All Events	####	####	####	####	####	81	56	2,609	1,814	205	132
P2	####	####	####	####	####	84	59	2,625	1,850	200	135
P8	P2	3,129	31.4	30.4	30.4	13	11	1,764	1,296	114	86
Q1	P8	8	0.3	0.1	0.1	-25	-20	299	295	3,971	2,866
Q2	P8	98	3.1	1.0	1.0	61	58	4,381	4,172	678	579
Q3	P8	544	17.4	5.5	5.3	-10	-12	336	276	18	8
Q4	P8	2,479	79.2	24.9	24.1	17	14	1,979	1,619	100	92
P7	P8	2,676	85.5	26.8	26.0	18	14	1,994	1,588	117	97
P1	P8	1,866	59.6	18.7	18.2	19	15	2,181	1,781	239	188
P4	P8	1,126	36.0	11.3	11.0	77	65	2,587	2,275	146	121

FACSDiva Version 6.1.3

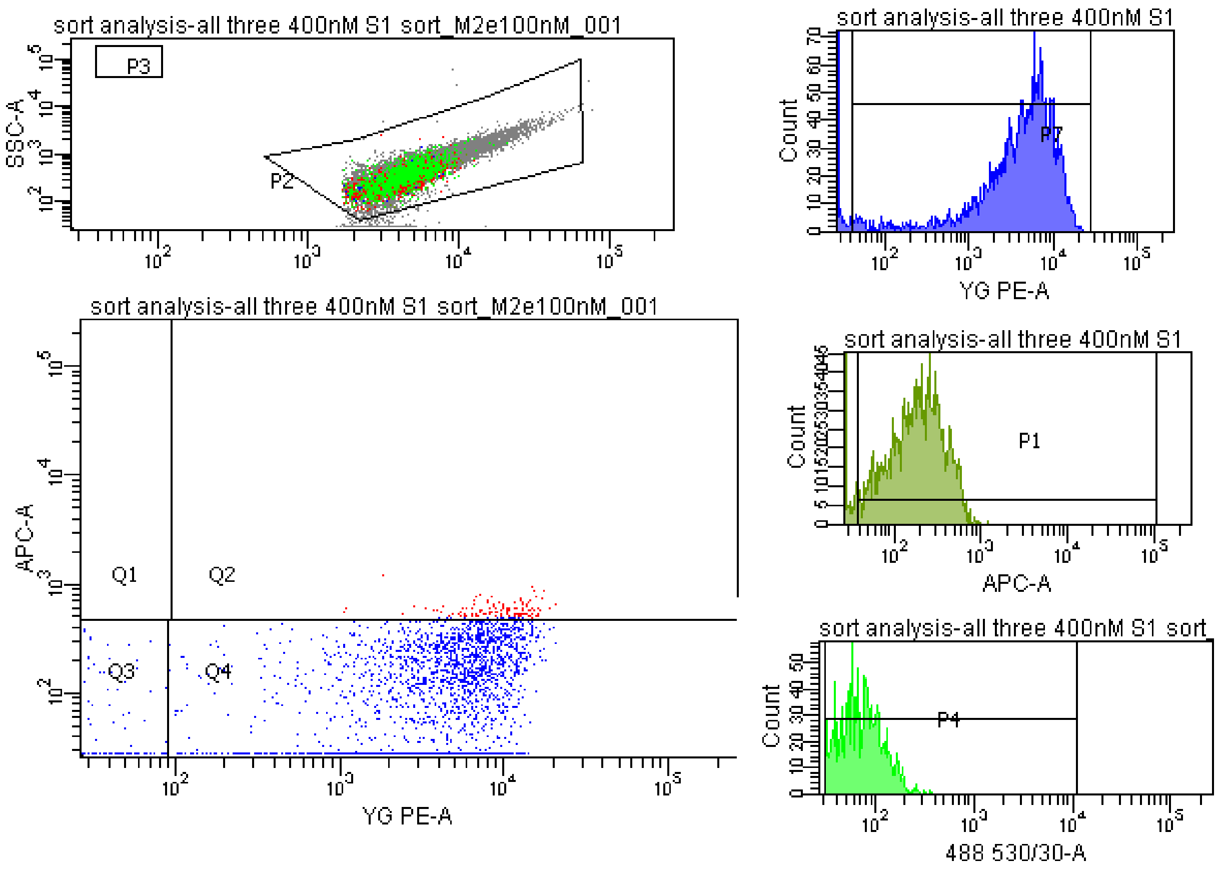

Experiment Name: Bio RBD2 selectons
Specimen Name: sort analysis
Tube Name: all three 400nM S1 sort_M2e100nM_001
Record Date: May 26, 2020 3:00:15 PM
$OP: Nileena
GUID: a73c724c-224e-42a2-b761-86f5da264e83

Population	Parent...	#Events	%Parent	%Gran...	%Total	488 5... Mean	488 5... Median	PE-A Mean	PE-A Median	APC-A Mean	APC-A Median
All Events	####	####	####	####	####	93	66	3,179	2,497	242	148
P2	####	####	####	####	####	97	71	3,218	2,552	194	150
P8	P2	2,980	29.9	29.0	29.0	19	17	2,085	1,831	106	93
Q1	P8	2	0.1	0.0	0.0	-7	-7	1,813	1,813	1,841	1,841
Q2	P8	120	4.0	1.2	1.2	59	51	4,359	4,240	567	546
Q3	P8	444	14.9	4.5	4.3	-14	-12	294	245	12	16
Q4	P8	2,414	81.0	24.3	23.5	24	22	2,302	2,085	99	98
P7	P8	2,591	86.9	26.0	25.2	25	22	2,354	2,111	118	104
P1	P8	1,838	61.7	18.5	17.9	25	21	2,466	2,293	220	186
P4	P8	1,196	40.1	12.0	11.6	79	70	2,823	2,681	139	127

FACSDiva Version 6.1.3

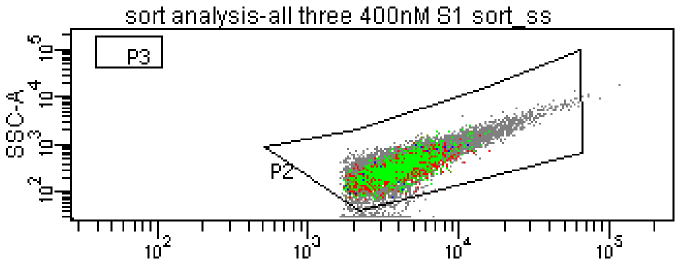

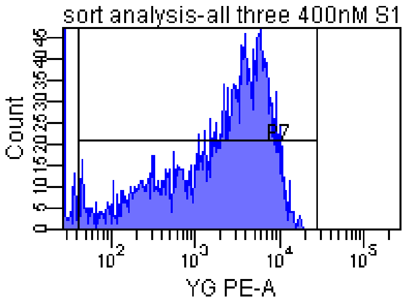

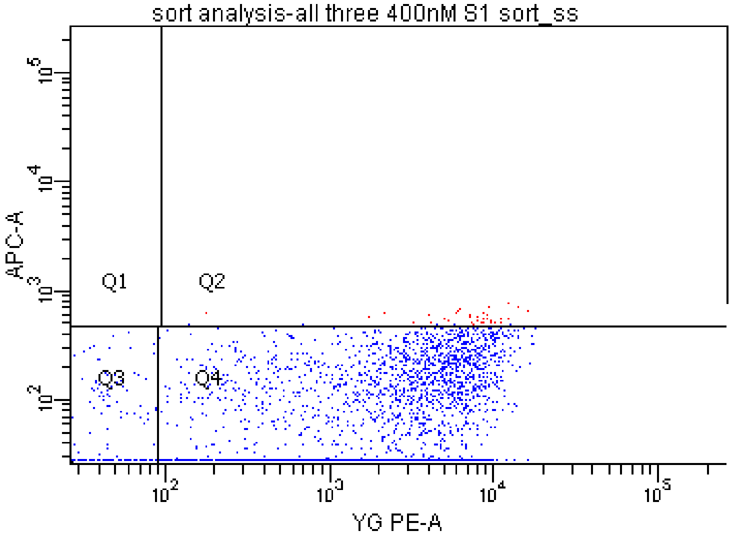

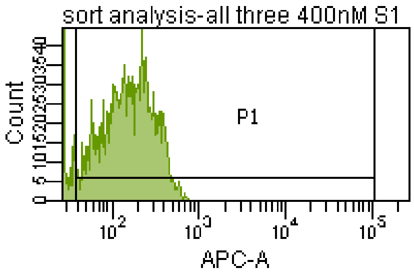

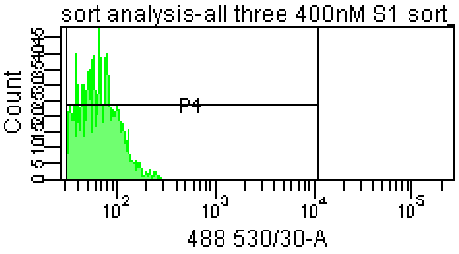

Experiment Name: Bio RBD2 selectons
Specimen Name: sort analysis
Tube Name: all three 400nM S1 sort_ss
Record Date: May 26, 2020 3:01:23 PM
$OP: Nileena
GUID: c35ba0b9-dbc8-4f95-bec5-8f7b59c934df

Population	Parent...	#Events	%Parent	%Gran...	%Total	488 5... Mean	488 5... Median	PE-A Mean	PE-A Median	APC-A Mean	APC-A Median
All Events	####	####	####	####	####	70	46	2,120	1,410	125	90
P2	####	####	####	####	####	75	50	2,157	1,448	128	93
P8	P2	3,100	31.2	29.9	29.9	7	5	1,409	1,073	66	55
Q1	P8	2	0.1	0.0	0.0	20	20	458	458	493	493
Q2	P8	34	1.1	0.3	0.3	21	20	3,410	3,454	551	531
Q3	P8	536	17.3	5.4	5.2	-13	-13	305	245	17	9
Q4	P8	2,528	81.5	25.5	24.3	11	10	1,617	1,380	69	62
P7	P8	2,657	85.7	26.8	25.6	10	9	1,595	1,347	74	63
P1	P8	1,663	53.6	16.7	16.0	11	10	1,744	1,500	183	155
P4	P8	1,029	33.2	10.4	9.9	72	64	1,968	1,751	89	83

FACSDiva Version 6.1.3

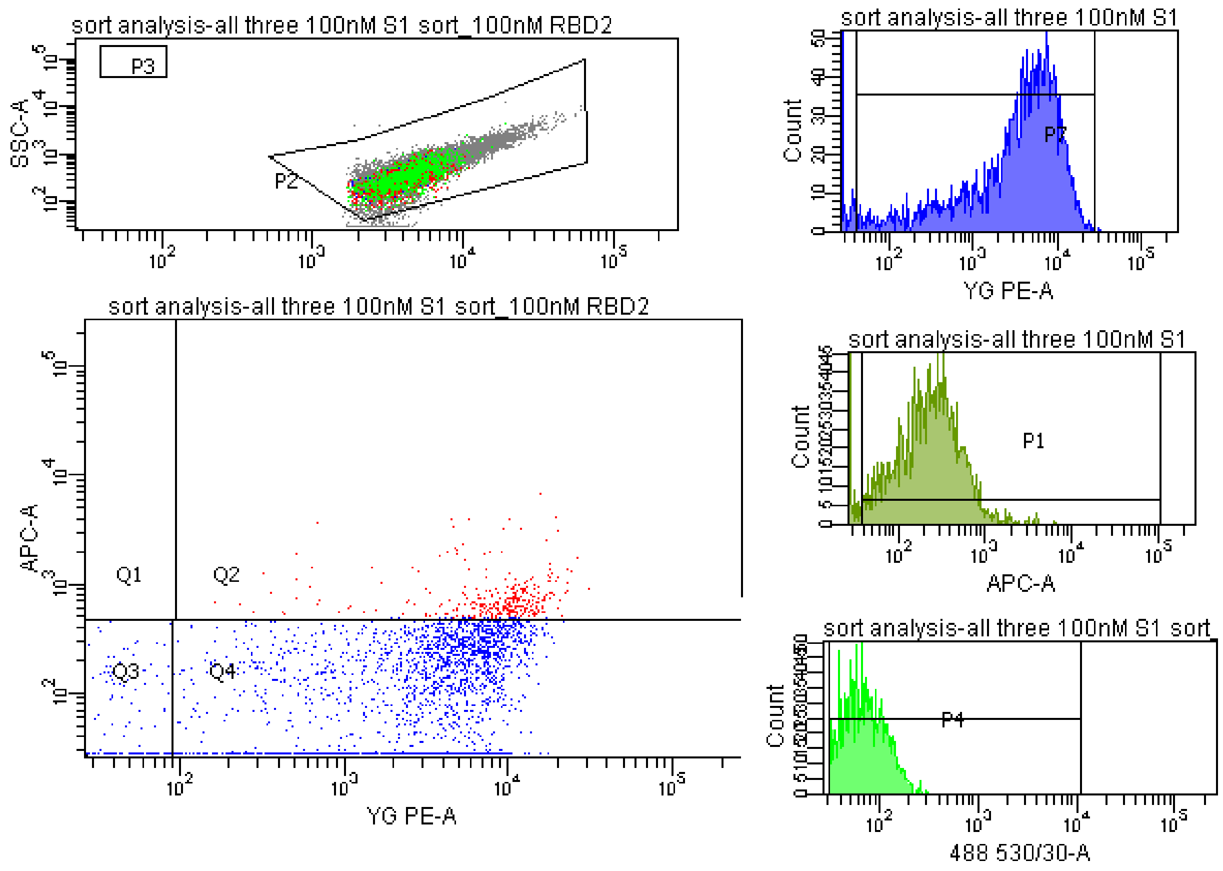

Experiment Name: Bio RBD2 selectons
Specimen Name: sort analysis
Tube Name: all three 100nM S1 sort_100nM RBD2
Record Date: May 26, 2020 3:02:34 PM
$OP: Nileena
GUID: 51da0c13-1c19-429e-bd77-711537d1e272

Population	Parent...	#Events	%Parent	%Gran...	%Total	488 5... Mean	488 5... Median	PE-A Mean	PE-A Median	APC-A Mean	APC-A Median
All Events	####	####	####	####	####	84	61	2,848	2,160	301	190
P2	####	####	####	####	####	88	64	2,896	2,202	305	194
P8	P2	3,007	30.2	29.3	29.3	16	12	1,908	1,502	182	128
Q1	P8	5	0.2	0.1	0.0	-3	7	252	229	648	491
Q2	P8	307	10.2	3.1	3.0	56	57	4,221	4,202	838	642
Q3	P8	533	17.7	5.4	5.2	-11	-11	288	238	14	6
Q4	P8	2,162	71.9	21.7	21.1	17	14	1,983	1,768	129	128
P7	P8	2,530	84.1	25.4	24.7	21	17	2,208	1,898	213	156
P1	P8	2,033	67.6	20.4	19.8	23	21	2,329	2,058	304	222
P4	P8	1,155	38.4	11.6	11.3	78	68	2,753	2,507	255	193

FACSDiva Version 6.1.3

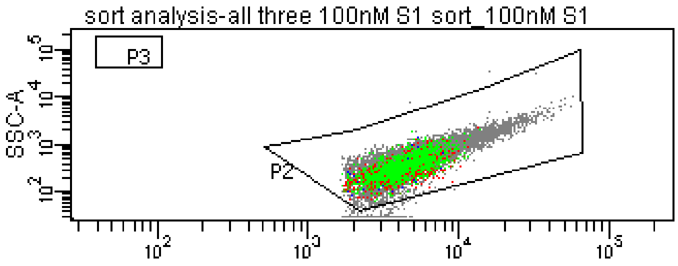

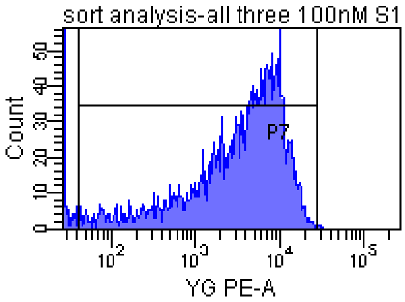

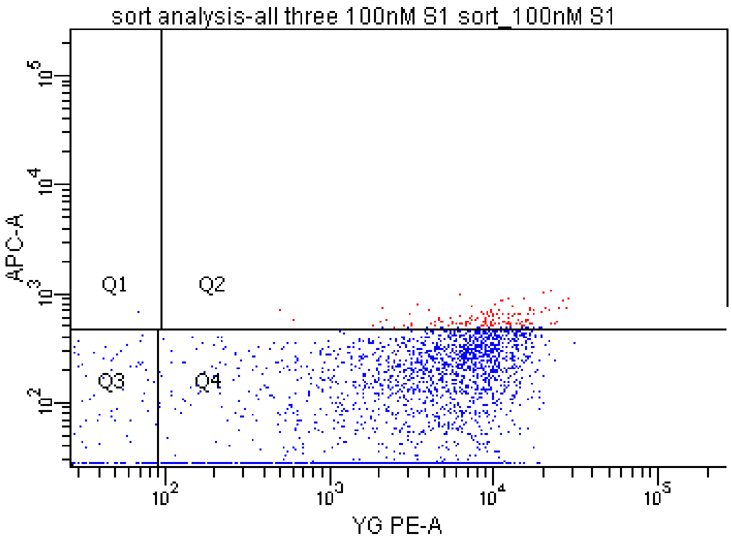

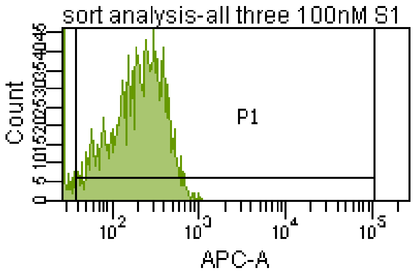

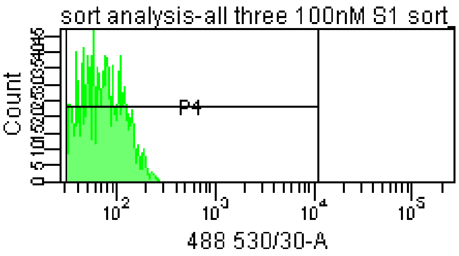

Experiment Name: Bio RBD2 selectons
Specimen Name: sort analysis
Tube Name: all three 100nM S1 sort_100nM S1
Record Date: May 26, 2020 3:03:46 PM
$OP: Nileena
GUID: f38b86f6-d279-4d97-9ea5-5346a2635fd1

Population	Parent...	#Events	%Parent	%Gran...	%Total	488 5... Mean	488 5... Median	PE-A Mean	PE-A Median	APC-A Mean	APC-A Median
All Events	####	####	####	####	####	89	63	3,048	2,234	217	157
P2	####	####	####	####	####	92	66	3,096	2,292	211	159
P8	P2	3,080	30.9	30.0	30.0	20	15	2,037	1,563	118	103
Q1	P8	3	0.1	0.0	0.0	7	7	328	125	2,809	638
Q2	P8	123	4.0	1.2	1.2	56	51	4,597	4,150	590	546
Q3	P8	510	16.6	5.1	5.0	-13	-13	293	238	19	10
Q4	P8	2,444	79.4	24.5	23.8	24	22	2,274	1,934	112	112
P7	P8	2,632	85.5	26.4	25.7	25	22	2,316	1,952	131	121
P1	P8	1,939	63.0	19.5	18.9	26	22	2,480	2,181	234	202
P4	P8	1,226	39.8	12.3	12.0	79	71	2,984	2,799	156	144

FACSDiva Version 6.1.3

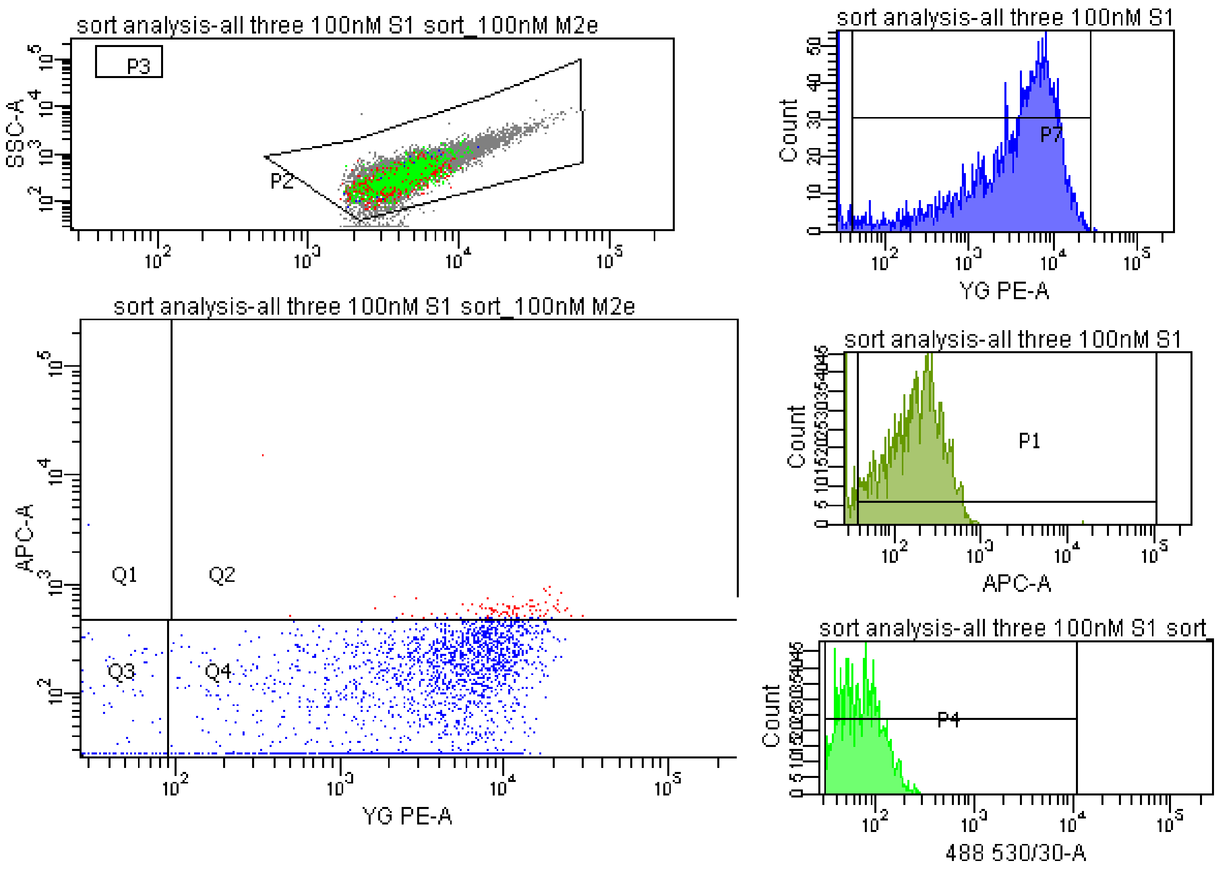

Experiment Name: Bio RBD2 selectons
Specimen Name: sort analysis
Tube Name: all three 100nM S1 sort_100nM M2e
Record Date: May 26, 2020 3:05:01 PM
$OP: Nileena
GUID: 88eab8d7-412a-4bc1-bb66-9aa91a38ec04

Population	Parent...	#Events	%Parent	%Gran...	%Total	488 5... Mean	488 5... Median	PE-A Mean	PE-A Median	APC-A Mean	APC-A Median
All Events	####	####	####	####	####	90	66	3,114	2,409	193	148
P2	####	####	####	####	####	94	71	3,167	2,466	196	150
P8	P2	3,005	30.2	29.3	29.3	19	17	2,056	1,658	114	97
Q1	P8	2	0.1	0.0	0.0	-15	-15	568	568	2,893	2,893
Q2	P8	89	3.0	0.9	0.9	71	78	5,226	4,753	735	558
Q3	P8	472	15.7	4.7	4.6	-10	-7	298	259	25	13
Q4	P8	2,442	81.3	24.5	23.8	23	22	2,282	2,016	106	102
P7	P8	2,585	86.0	26.0	25.2	24	22	2,338	2,027	126	108
P1	P8	1,893	63.0	19.0	18.4	25	23	2,470	2,189	221	181
P4	P8	1,213	40.4	12.2	11.8	78	70	2,934	2,724	141	135

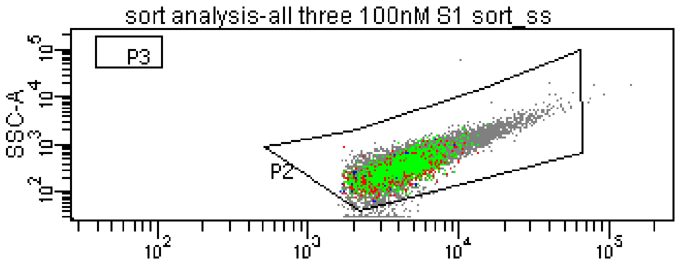

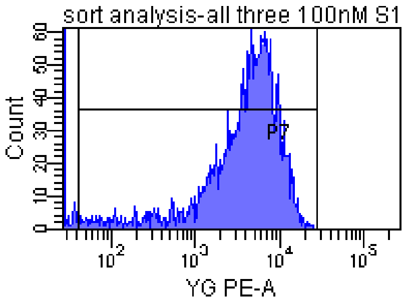

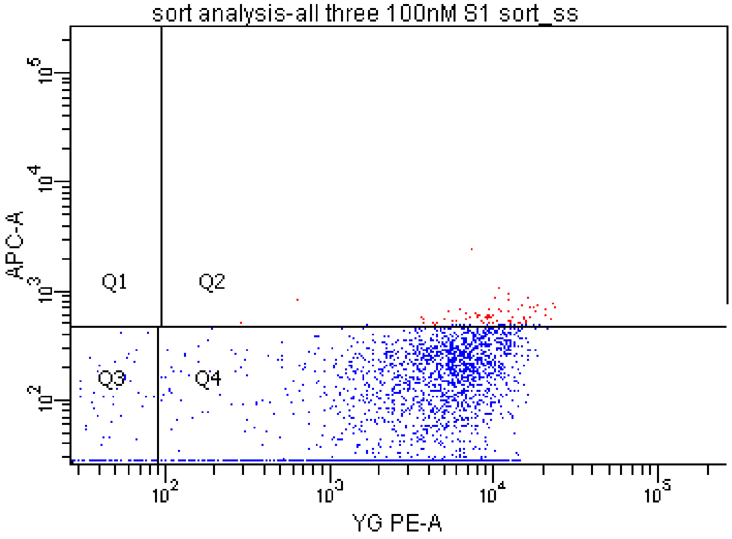

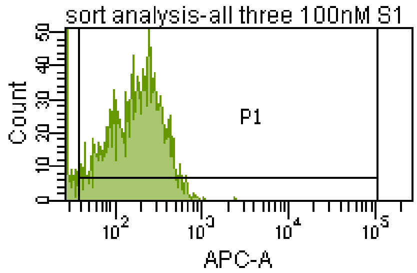

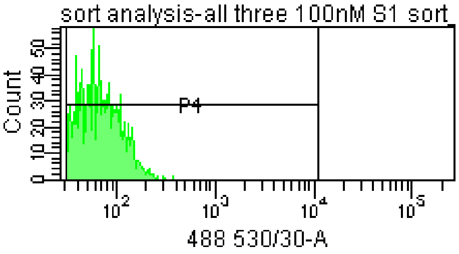

Experiment Name: Bio RBD2 selectons
Specimen Name: sort analysis
Tube Name: all three 100nM S1 sort_ss
Record Date: May 26, 2020 3:06:03 PM
$OP: Nileena
GUID: 02f27c75-4d3c-4064-b3a6-79d9e45b640e

Population	Parent...	#Events	%Parent	%Gran...	%Total	488 5... Mean	488 5... Median	PE-A Mean	PE-A Median	APC-A Mean	APC-A Median
All Events	####	####	####	####	####	95	61	2,970	2,246	218	130
P2	####	####	####	####	####	92	64	2,974	2,280	177	131
P8	P2	3,069	30.8	29.9	29.9	18	16	1,967	1,734	95	79
Q1	P8	0	0.0	0.0	0.0	####	####	####	####	####	####
Q2	P8	67	2.2	0.7	0.7	58	44	4,752	4,526	617	554
Q3	P8	459	15.0	4.6	4.5	-10	-12	281	249	20	19
Q4	P8	2,543	82.9	25.5	24.8	22	22	2,198	1,984	95	87
P7	P8	2,661	86.7	26.7	25.9	23	22	2,226	1,984	107	91
P1	P8	1,823	59.4	18.3	17.7	24	22	2,341	2,172	204	175
P4	P8	1,231	40.1	12.4	12.0	75	64	2,652	2,490	122	107

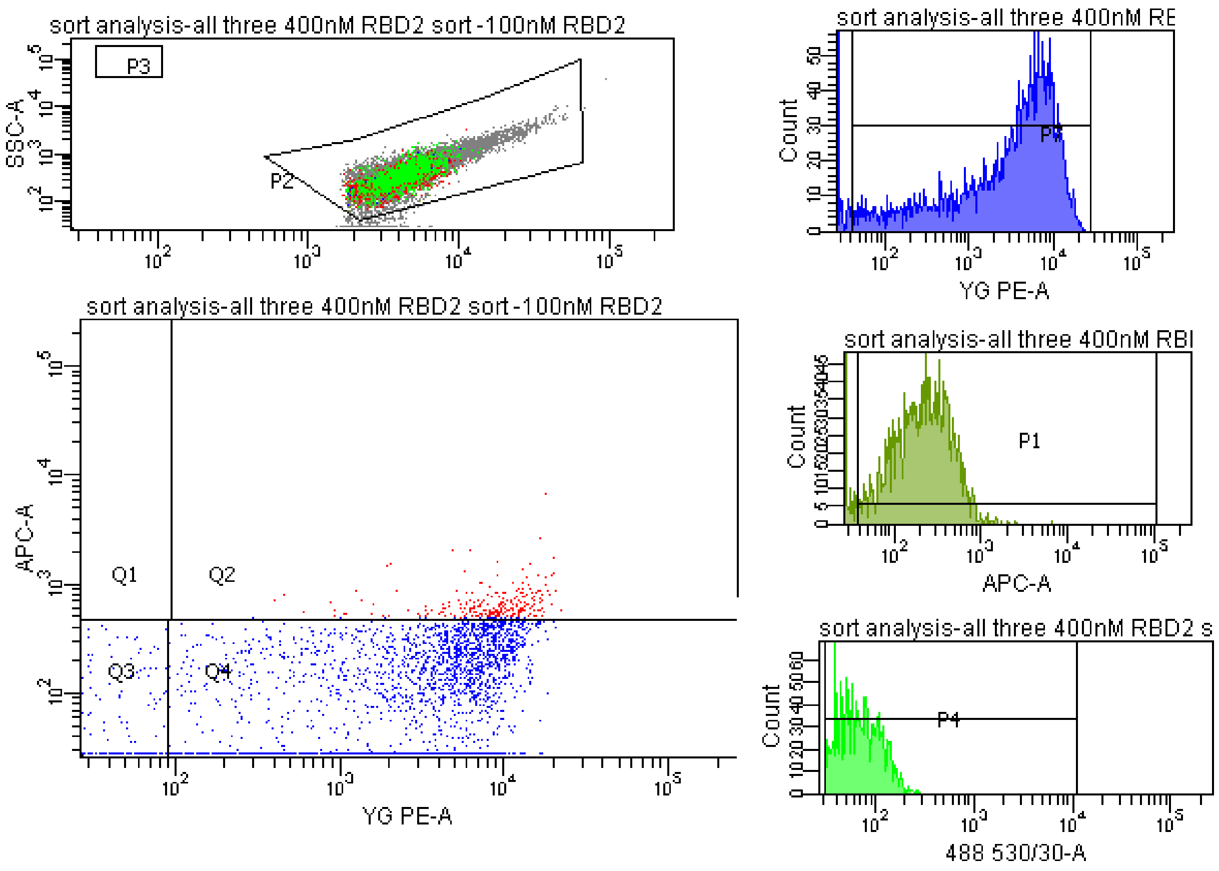

Experiment Name:	Bio RBD2 selectons
Specimen Name:	sort analysis
Tube Name:	all three 400nM RBD2 sort-100nM RBD2
Record Date:	May 26, 2020 3:08:05 PM
$OP:	Nileena
GUID:	1dbbf0e0-8056-4a08-8283-5fc4b3d20a4f

Population	Parent...	#Events	%Parent	%Gran...	%Total	488 5... Mean	488 5... Median	PE-A Mean	PE-A Median	APC-A Mean	APC-A Median
All Events	####	####	####	####	####	83	59	2,808	2,019	247	166
P2	####	####	####	####	####	86	62	2,846	2,068	240	168
P8	P2	3,297	33.1	32.1	32.1	18	16	1,829	1,357	143	112
Q1	P8	1	0.0	0.0	0.0	35	35	47	47	1,086	1,086
Q2	P8	244	7.4	2.4	2.4	57	53	4,158	4,021	689	581
Q3	P8	694	21.0	7.0	6.8	-8	-5	288	249	15	3
Q4	P8	2,358	71.5	23.7	22.9	22	22	2,042	1,813	124	120
P7	P8	2,712	82.3	27.2	26.4	24	24	2,161	1,876	170	138
P1	P8	2,128	64.5	21.3	20.7	24	24	2,295	2,092	261	208
P4	P8	1,320	40.0	13.2	12.8	77	68	2,677	2,494	199	156

FACSDiva Version 6.1.3

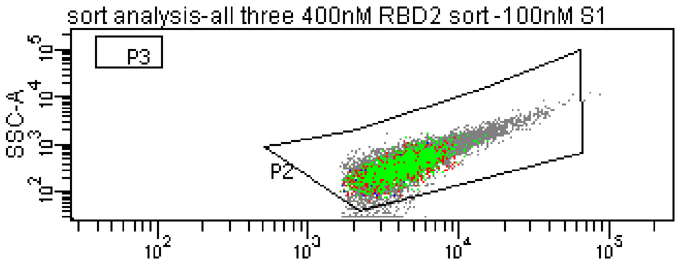

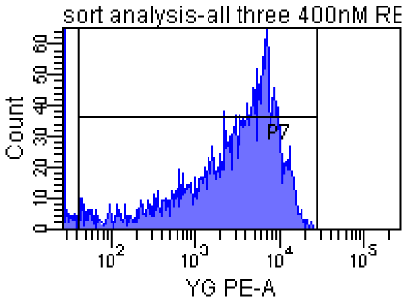

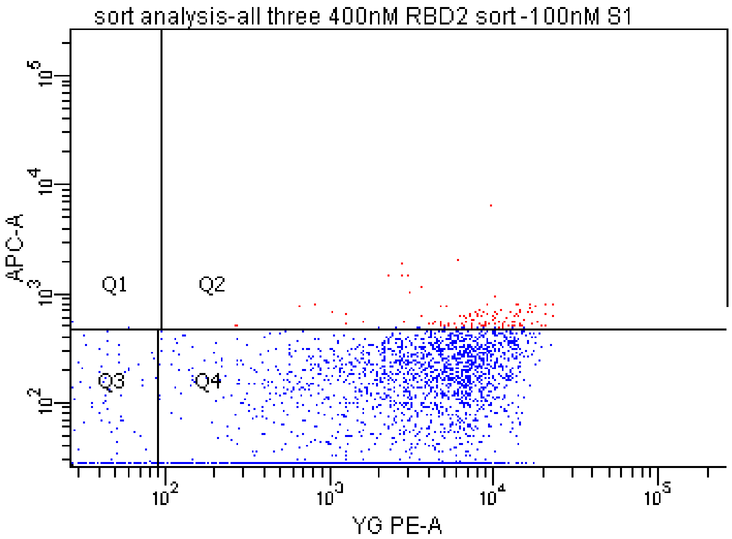

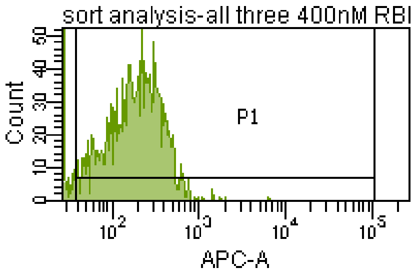

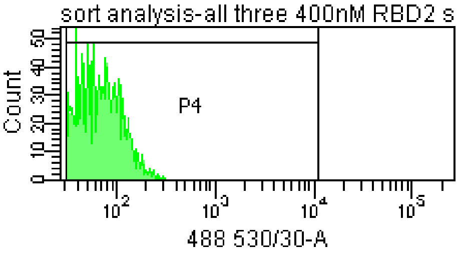

Experiment Name: Bio RBD2 selectons
Specimen Name: sort analysis
Tube Name: all three 400nM RBD2 sort-100nM S1
Record Date: May 26, 2020 3:09:14 PM
$OP: Nileena
GUID: 0c74dae1-bb21-4567-854e-2b91caf201f9

Population	Parent...	#Events	%Parent	%Gran...	%Total	488 5... Mean	488 5... Median	PE-A Mean	PE-A Median	APC-A Mean	APC-A Median
All Events	####	####	####	####	####	76	53	2,566	1,915	167	131
P2	####	####	####	####	####	79	57	2,601	1,965	169	133
P8	P2	3,392	34.1	33.1	33.1	16	14	1,775	1,347	100	83
Q1	P8	5	0.1	0.1	0.0	33	10	273	249	586	535
Q2	P8	111	3.3	1.1	1.1	54	54	4,000	3,830	680	555
Q3	P8	597	17.6	6.0	5.8	-5	-5	272	247	9	5
Q4	P8	2,679	79.0	26.9	26.1	19	17	2,020	1,710	95	92
P7	P8	2,877	84.8	28.9	28.0	20	19	2,045	1,708	115	99
P1	P8	2,004	59.1	20.1	19.5	21	17	2,184	1,844	223	187
P4	P8	1,290	38.0	13.0	12.6	76	66	2,562	2,364	134	112

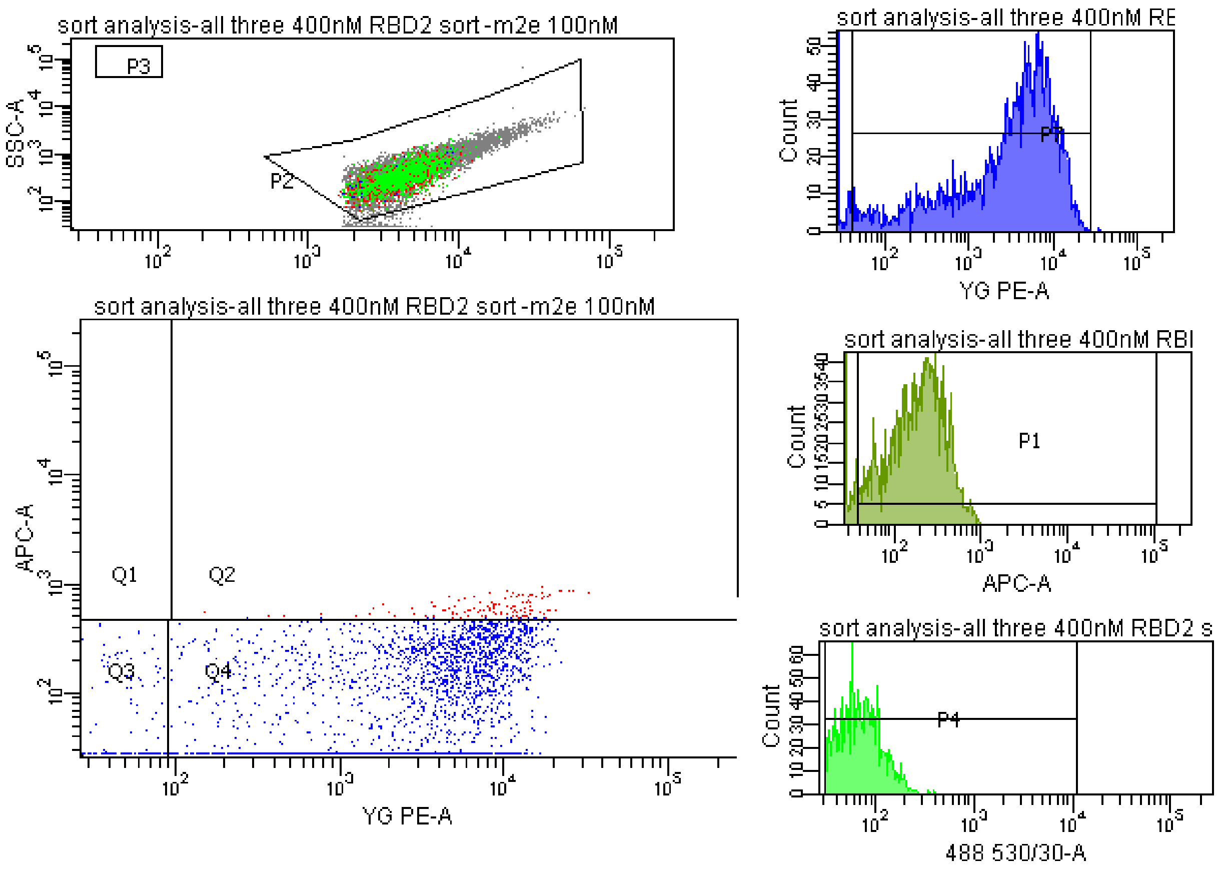

Experiment Name: Bio RBD2 selectons
Specimen Name: sort analysis
Tube Name: all three 400nM RBD2 sort-m2e 100nM
Record Date: May 26, 2020 3:10:27 PM
$OP: Nileena
GUID: 86587a58-4de9-4491-9855-64a964d67988

Population	Parent...	#Events	%Parent	%Gran...	%Total	488 5... Mean	488 5... Median	PE-A Mean	PE-A Median	APC-A Mean	APC-A Median
All Events	####	####	####	####	####	85	60	2,879	2,085	197	126
P2	####	####	####	####	####	88	63	2,924	2,148	175	128
P8	P2	3,318	33.3	32.2	32.2	18	15	1,867	1,417	102	81
Q1	P8	3	0.1	0.0	0.0	-102	-156	408	452	6,195	502
Q2	P8	114	3.4	1.1	1.1	59	53	4,376	3,852	597	559
Q3	P8	622	18.7	6.2	6.0	-7	-8	287	249	18	-1
Q4	P8	2,579	77.7	25.9	25.1	22	20	2,139	1,815	94	92
P7	P8	2,789	84.1	28.0	27.1	22	20	2,157	1,801	111	97
P1	P8	1,945	58.6	19.5	18.9	24	22	2,338	1,996	232	193
P4	P8	1,300	39.2	13.0	12.6	77	68	2,729	2,509	134	121

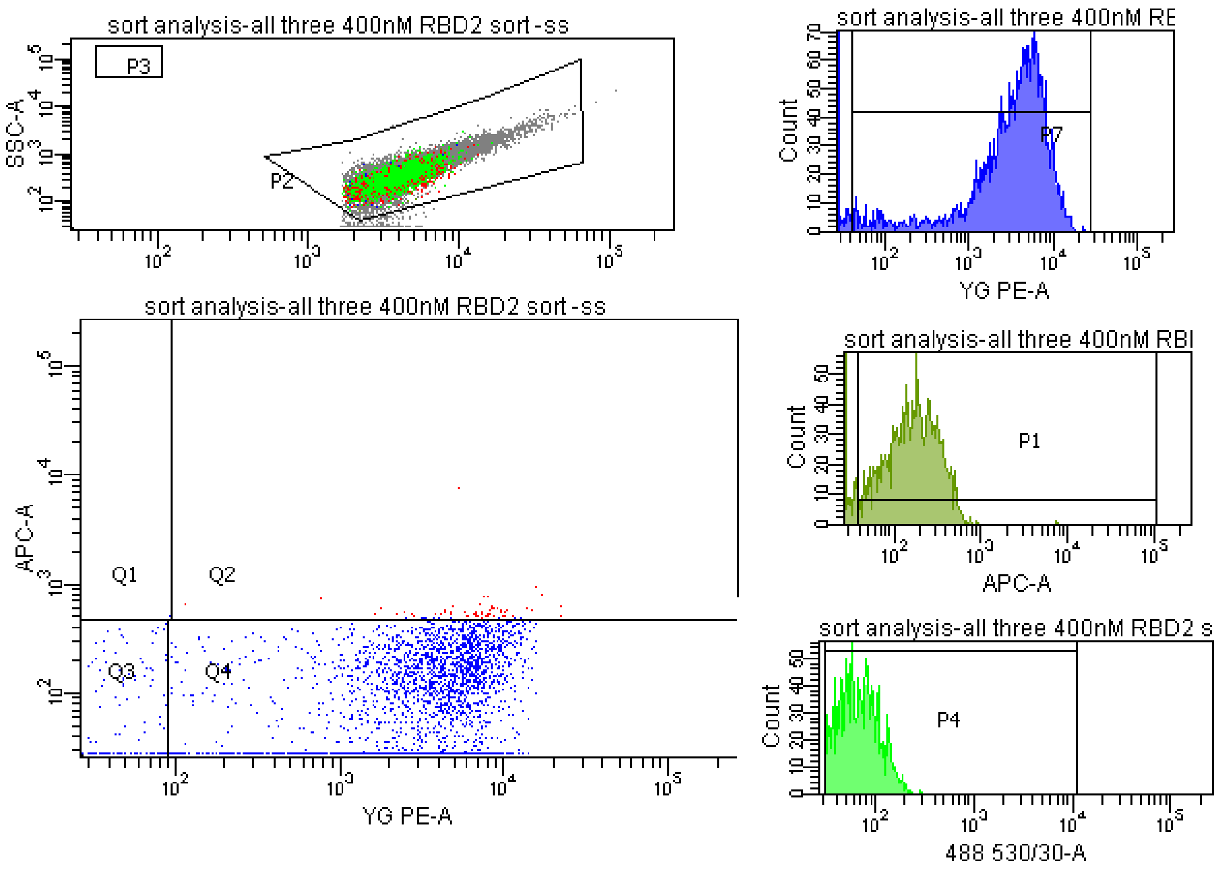

Experiment Name: Bio RBD2 selectons
Specimen Name: sort analysis
Tube Name: all three 400nM RBD2 sort-ss
Record Date: May 26, 2020 3:11:24 PM
$OP: Nileena
GUID: 60ef78a7-5b20-408b-9338-ac13c50adffd

Population	Parent...	#Events	%Parent	%Gran...	%Total	488 5... Mean	488 5... Median	PE-A Mean	PE-A Median	APC-A Mean	APC-A Median
All Events	####	####	####	####	####	85	52	2,500	1,900	174	111
P2	####	####	####	####	####	80	56	2,517	1,932	151	113
P8	P2	3,496	35.1	33.9	33.9	17	15	1,682	1,458	90	76
Q1	P8	2	0.1	0.0	0.0	7	7	955	955	3,468	3,468
Q2	P8	55	1.6	0.6	0.5	50	41	3,447	3,359	673	524
Q3	P8	598	17.1	6.0	5.8	-6	-7	256	220	27	8
Q4	P8	2,841	81.3	28.5	27.5	21	20	1,948	1,770	89	86
P7	P8	2,984	85.4	30.0	28.9	21	20	1,928	1,743	99	88
P1	P8	2,059	58.9	20.7	20.0	20	16	1,957	1,798	198	163
P4	P8	1,360	38.9	13.7	13.2	74	66	2,203	2,056	97	88

FACSDiva Version 6.1.3

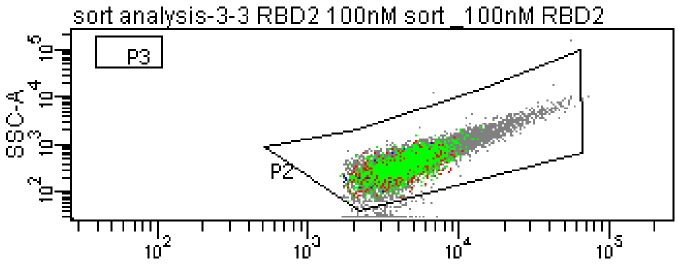

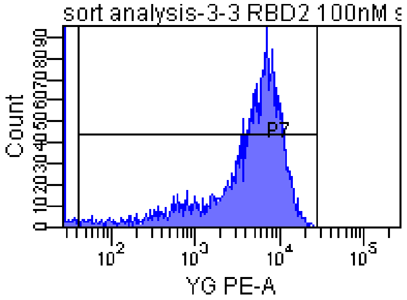

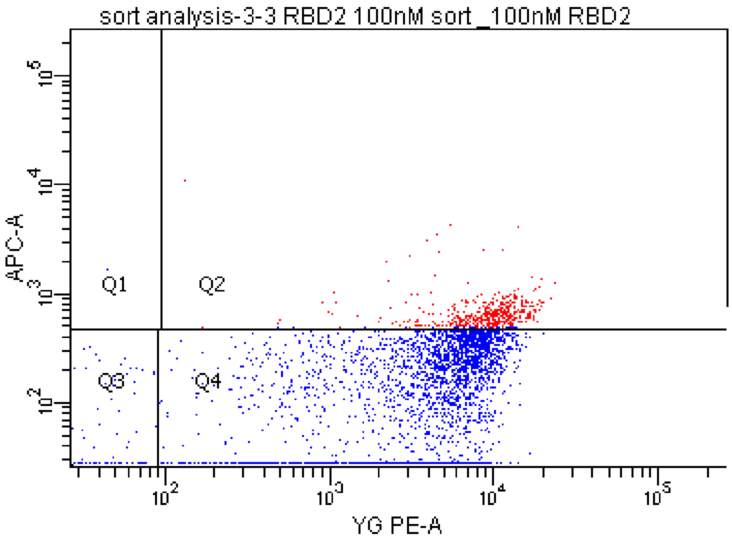

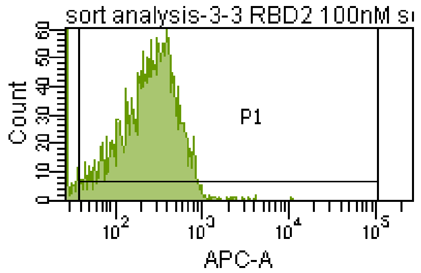

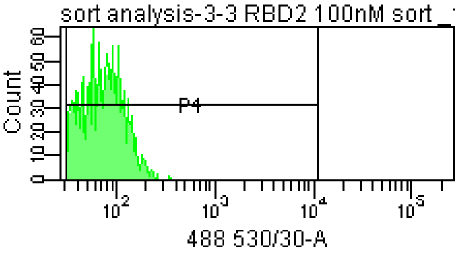

Experiment Name: Bio RBD2 selectons
Specimen Name: sort analysis
Tube Name: 3-3 RBD2 100nM sort _100nM RBD2
Record Date: May 26, 2020 3:13:33 PM
$OP: Nileena
GUID: 21f70134-2fb5-47af-822e-3f1e99694ade

Population	Parent...	#Events	%Parent	%Gran...	%Total	488 5... Mean	488 5... Median	PE-A Mean	PE-A Median	APC-A Mean	APC-A Median
All Events	####	####	####	####	####	98	72	3,436	2,772	399	243
P2	####	####	####	####	####	101	75	3,457	2,809	369	246
P8	P2	3,491	35.0	34.2	34.2	28	27	2,294	2,146	212	181
Q1	P8	4	0.1	0.0	0.0	-11	-8	505	437	1,316	1,133
Q2	P8	416	11.9	4.2	4.1	61	58	4,265	4,222	711	601
Q3	P8	378	10.8	3.8	3.7	-1	2	254	221	18	16
Q4	P8	2,693	77.1	27.0	26.4	28	26	2,278	2,201	160	164
P7	P8	3,153	90.3	31.6	30.9	31	31	2,513	2,372	231	200
P1	P8	2,604	74.6	26.1	25.5	34	33	2,645	2,595	306	254
P4	P8	1,643	47.1	16.5	16.1	80	72	2,974	2,874	256	228

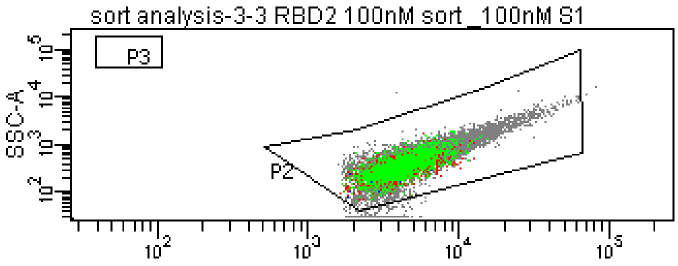

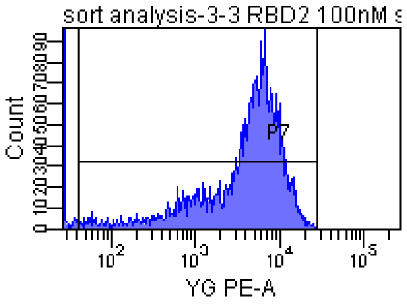

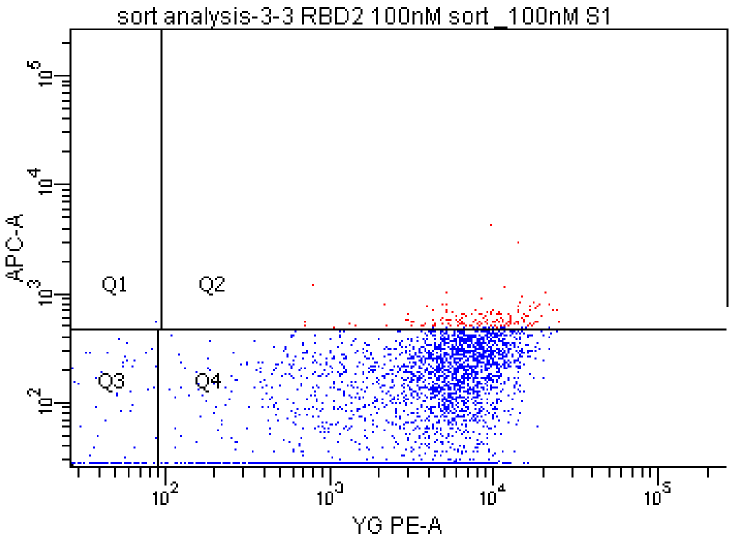

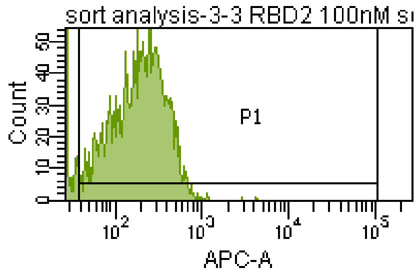

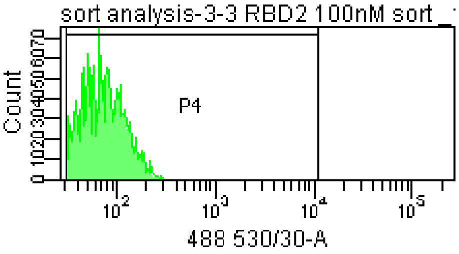

Experiment Name: Bio RBD2 selectons
Specimen Name: sort analysis
Tube Name: 3-3 RBD2 100nM sort _100nM S1
Record Date: May 26, 2020 3:14:40 PM
$OP: Nileena
GUID: 5a713f66-fba1-4234-9c4a-dfb332a08c7b

Population	Parent...	#Events	%Parent	%Gran...	%Total	488 5... Mean	488 5... Median	PE-A Mean	PE-A Median	APC-A Mean	APC-A Median
All Events	####	####	####	####	####	90	66	3,138	2,509	210	152
P2	####	####	####	####	####	93	69	3,174	2,542	211	154
P8	P2	3,695	37.1	36.1	36.1	28	25	2,182	2,006	124	105
Q1	P8	5	0.1	0.1	0.0	-22	-12	273	193	1,891	520
Q2	P8	148	4.0	1.5	1.4	60	59	4,327	4,078	624	542
Q3	P8	417	11.3	4.2	4.1	-5	-7	231	207	25	17
Q4	P8	3,125	84.6	31.4	30.5	31	28	2,344	2,183	110	107
P7	P8	3,335	90.3	33.5	32.6	31	29	2,392	2,200	131	117
P1	P8	2,389	64.7	24.0	23.3	33	31	2,572	2,381	229	194
P4	P8	1,712	46.3	17.2	16.7	80	70	2,831	2,636	152	135

FACSDiva Version 6.1.3

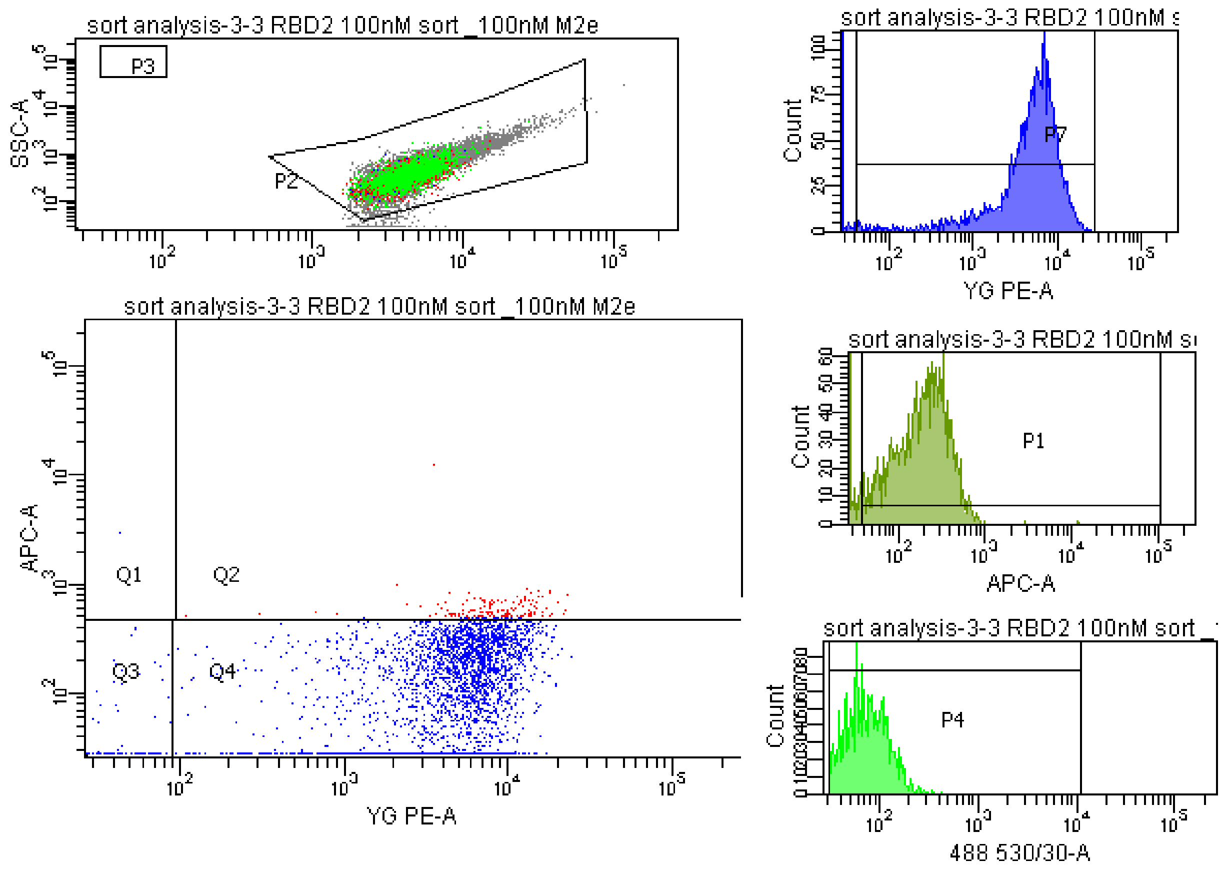

Experiment Name: Bio RBD2 selectons
Specimen Name: sort analysis
Tube Name: 3-3 RBD2 100nM sort _100nM M2e
Record Date: May 26, 2020 3:16:06 PM
$OP: Nileena
GUID: a0acb1ea-6d13-4ca8-81ce-3dbd2809fb7d

Population	Parent...	#Events	%Parent	%Gran...	%Total	488 5... Mean	488 5... Median	PE-A Mean	PE-A Median	APC-A Mean	APC-A Median
All Events	####	####	####	####	####	90	68	3,142	2,636	184	150
P2	####	####	####	####	####	92	71	3,166	2,675	185	151
P8	P2	3,778	38.0	37.0	37.0	31	29	2,304	2,170	119	104
Q1	P8	1	0.0	0.0	0.0	-8	-8	70	70	2,856	2,856
Q2	P8	134	3.5	1.3	1.3	58	50	4,218	3,882	661	539
Q3	P8	433	11.5	4.4	4.2	-3	-5	280	245	5	-9
Q4	P8	3,210	85.0	32.3	31.4	35	34	2,498	2,347	110	108
P7	P8	3,393	89.8	34.1	33.2	35	34	2,533	2,361	132	117
P1	P8	2,442	64.6	24.5	23.9	36	34	2,658	2,536	225	194
P4	P8	1,841	48.7	18.5	18.0	81	72	2,877	2,709	135	128

FACSDiva Version 6.1.3

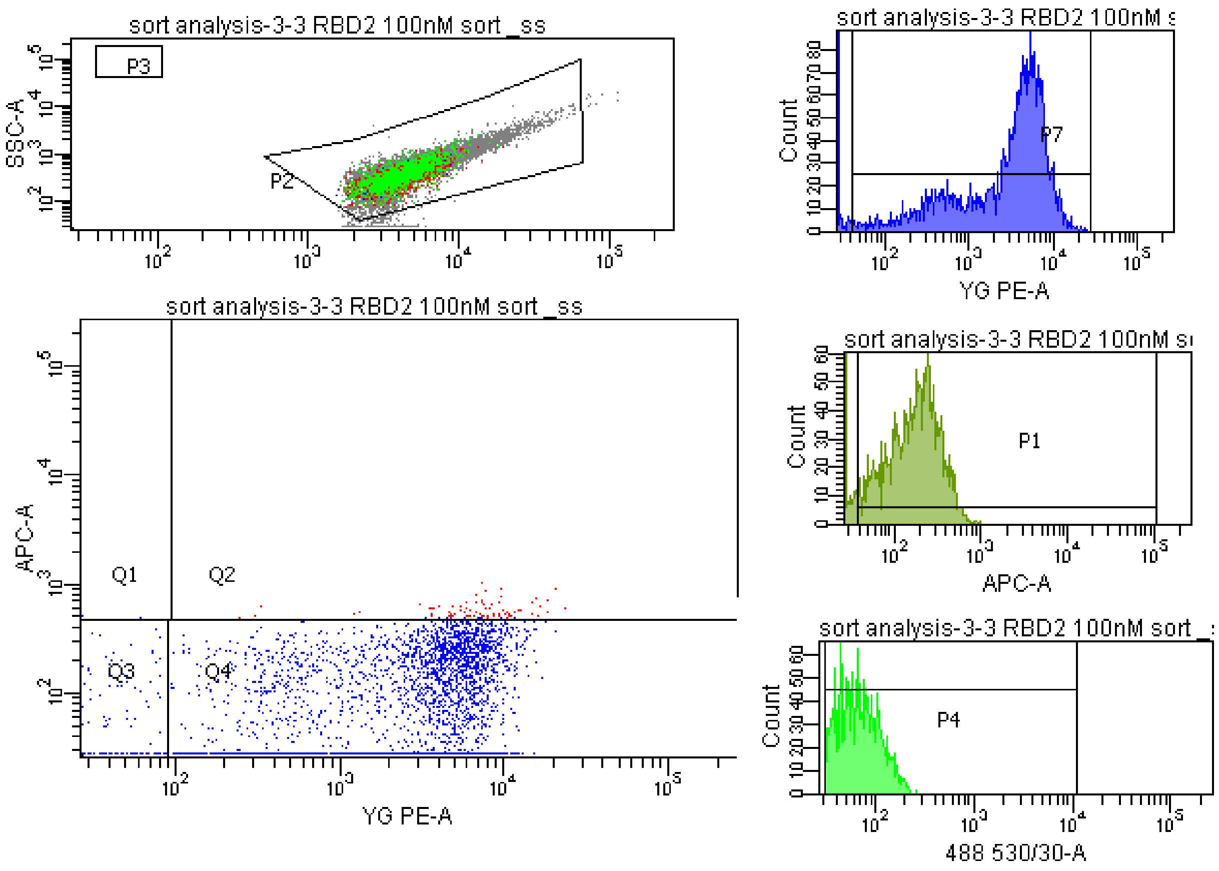

Experiment Name: Bio RBD2 selectons
Specimen Name: sort analysis
Tube Name: 3-3 RBD2 100nM sort _ss
Record Date: May 26, 2020 3:17:06 PM
$OP: Nileena
GUID: 7349c466-c0ee-4219-9ca0-10bb02f9d457

Population	Parent...	#Events	%Parent	%Gran...	%Total	488 5... Mean	488 5... Median	PE-A Mean	PE-A Median	APC-A Mean	APC-A Median
All Events	####	####	####	####	####	78	53	2,467	1,908	159	119
P2	####	####	####	####	####	80	56	2,475	1,936	159	122
P8	P2	3,688	37.1	35.9	35.9	18	17	1,693	1,562	95	81
Q1	P8	2	0.1	0.0	0.0	-9	-9	586	586	502	502
Q2	P8	79	2.1	0.8	0.8	61	73	3,698	3,375	563	528
Q3	P8	451	12.2	4.5	4.4	-8	-9	250	198	18	15
Q4	P8	3,156	85.6	31.7	30.7	21	21	1,849	1,755	94	88
P7	P8	3,303	89.6	33.2	32.2	22	21	1,861	1,753	103	93
P1	P8	2,240	60.7	22.5	21.8	21	21	1,970	1,899	197	175
P4	P8	1,499	40.6	15.1	14.6	75	65	2,238	2,148	114	101

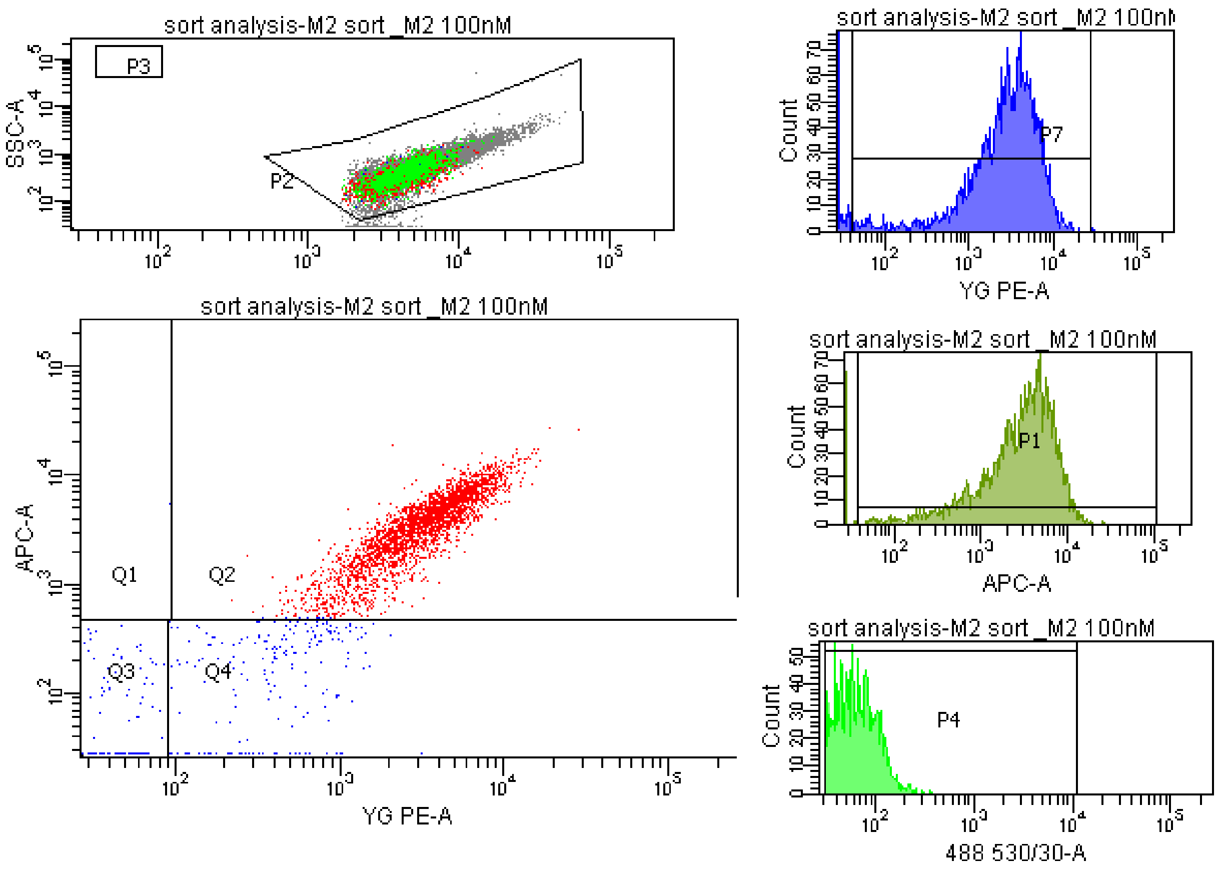

Experiment Name: Bio RBD2 selectons
Specimen Name: sort analysis
Tube Name: M2 sort _M2 100nM
Record Date: May 26, 2020 3:18:22 PM
$OP: Nileena
GUID: e5c4c4f3-192e-4e7a-be69-16ba30e00405

Population	Parent...	#Events	%Parent	%Gran...	%Total	488 5... Mean	488 5... Median	PE-A Mean	PE-A Median	APC-A Mean	APC-A Median
All Events	####	####	####	####	####	71	49	2,186	1,628	4,433	3,358
P2	####	####	####	####	####	74	51	2,214	1,654	4,474	3,407
P8	P2	3,606	36.2	35.3	35.3	14	13	1,441	1,230	3,124	2,571
Q1	P8	3	0.1	0.0	0.0	-24	-19	151	52	2,104	515
Q2	P8	2,805	77.8	28.2	27.5	20	17	1,758	1,552	3,993	3,499
Q3	P8	586	16.3	5.9	5.7	-7	-11	280	241	28	21
Q4	P8	212	5.9	2.1	2.1	-0	-1	482	453	199	220
P7	P8	3,069	85.1	30.8	30.1	18	15	1,641	1,433	3,658	3,190
P1	P8	3,244	90.0	32.6	31.8	16	15	1,570	1,370	3,483	3,009
P4	P8	1,316	36.5	13.2	12.9	71	62	1,869	1,648	4,041	3,578

FACSDiva Version 6.1.3

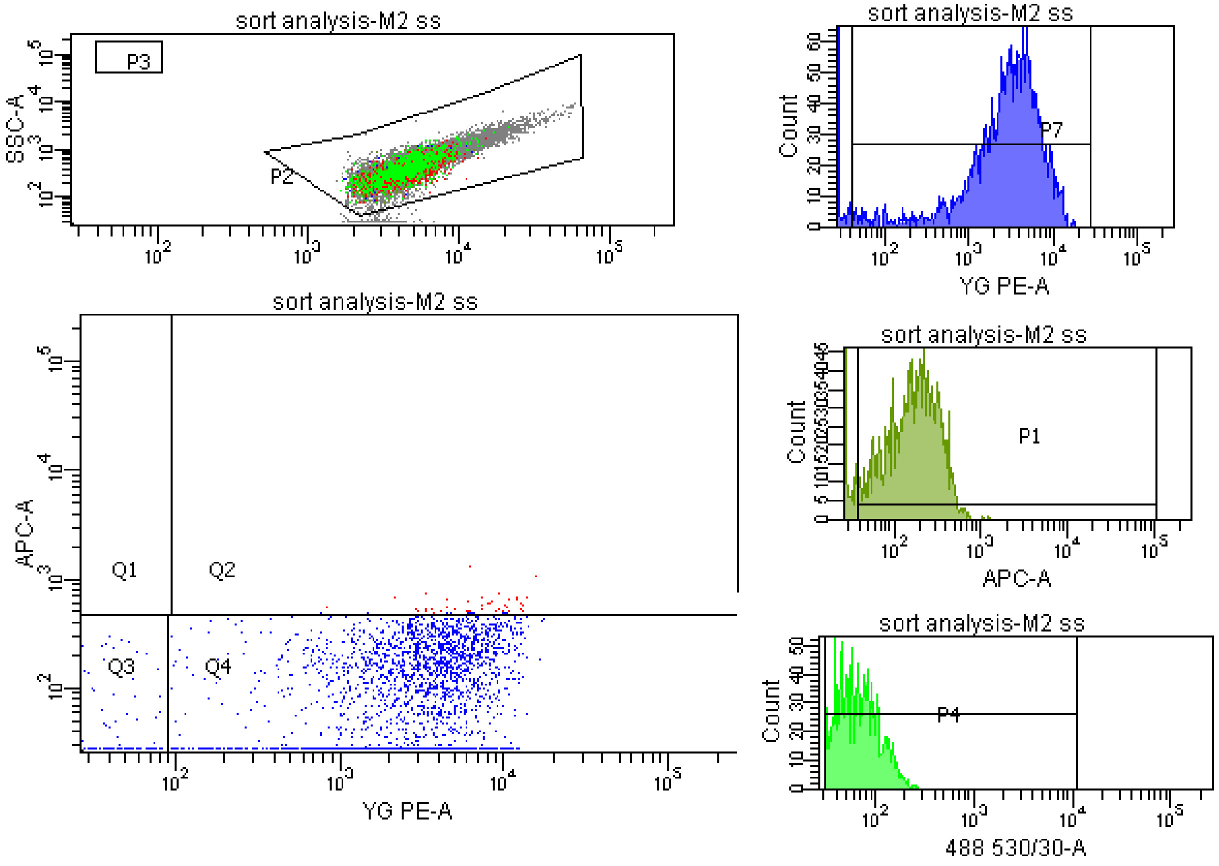

Experiment Name: Bio RBD2 selectons
Specimen Name: sort analysis
Tube Name: M2 ss
Record Date: May 26, 2020 3:19:33 PM
$OP: Nileena
GUID: 5b002252-64a5-4886-a772-3b35f8559086

Population	Parent...	#Events	%Parent	%Gran...	%Total	488 5... Mean	488 5... Median	PE-A Mean	PE-A Median	APC-A Mean	APC-A Median
All Events	####	####	####	####	####	77	54	2,385	1,730	147	117
P2	####	####	####	####	####	79	57	2,414	1,758	149	119
P8	P2	3,365	33.7	33.1	33.1	15	12	1,515	1,271	82	71
Q1	P8	1	0.0	0.0	0.0	-56	-56	258	258	479	479
Q2	P8	41	1.2	0.4	0.4	58	51	3,445	3,391	576	526
Q3	P8	581	17.3	5.8	5.7	-9	-10	276	233	21	17
Q4	P8	2,742	81.5	27.5	26.9	19	16	1,750	1,506	87	82
P7	P8	2,839	84.4	28.5	27.9	20	16	1,745	1,492	92	82
P1	P8	1,941	57.7	19.5	19.1	19	15	1,788	1,565	192	166
P4	P8	1,261	37.5	12.6	12.4	73	64	1,997	1,756	102	94

FACSDiva Version 6.1.3

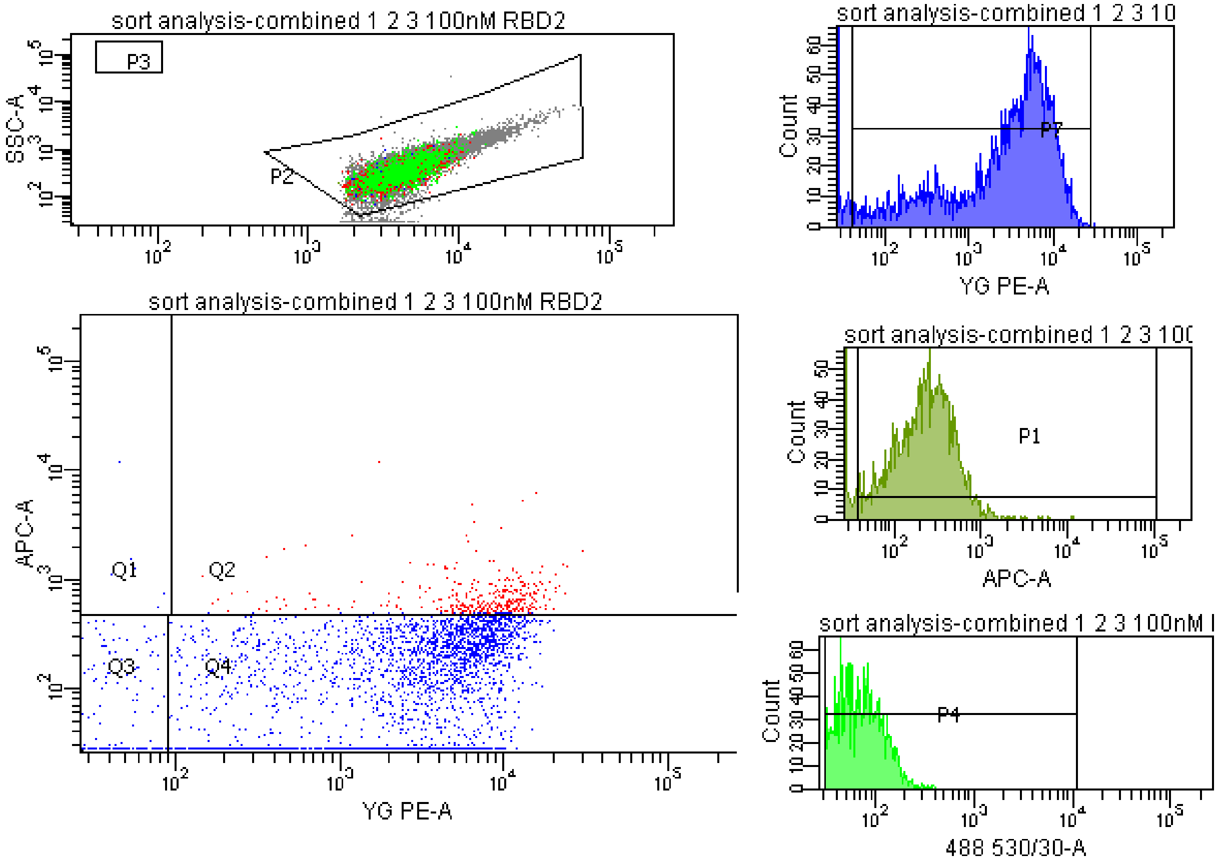

Experiment Name: Bio RBD2 selectons
Specimen Name: sort analysis
Tube Name: combined 1 2 3 100nM RBD2
Record Date: May 26, 2020 3:27:06 PM
$OP: Nileena
GUID: 5fe7876d-5ed1-4852-98f9-1fe903515d3e

Population	Parent...	#Events	%Parent	%Gran...	%Total	488 5... Mean	488 5... Median	PE-A Mean	PE-A Median	APC-A Mean	APC-A Median
All Events	####	####	####	####	####	77	54	2,566	1,916	286	174
P2	####	####	####	####	####	81	58	2,616	1,962	285	177
P8	P2	3,761	37.7	36.5	36.5	22	19	1,785	1,370	180	125
Q1	P8	18	0.5	0.2	0.2	-24	-40	261	245	3,139	662
Q2	P8	323	8.6	3.2	3.1	58	46	3,935	3,652	790	603
Q3	P8	703	18.7	7.1	6.8	-5	-2	250	214	23	12
Q4	P8	2,717	72.2	27.3	26.4	25	23	1,937	1,671	128	128
P7	P8	3,140	83.5	31.5	30.5	27	24	2,084	1,789	197	153
P1	P8	2,496	66.4	25.1	24.3	28	25	2,188	1,915	308	226
P4	P8	1,562	41.5	15.7	15.2	79	69	2,562	2,342	221	186

FACSDiva Version 6.1.3

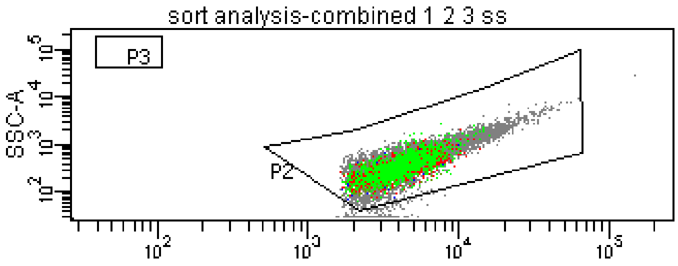

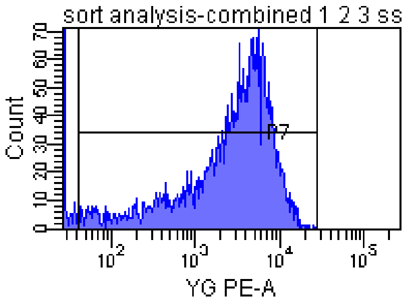

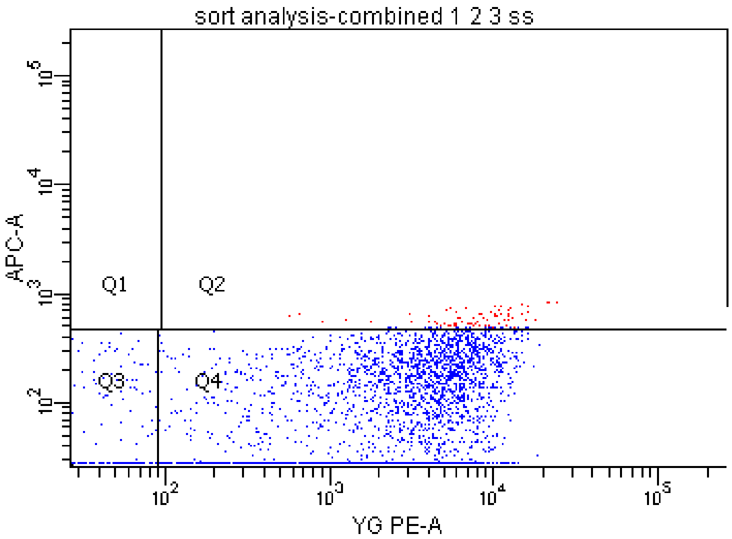

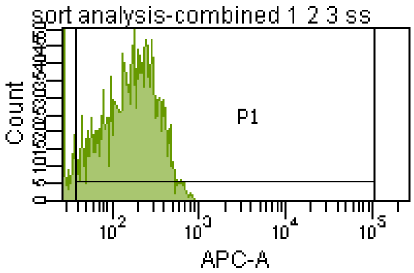

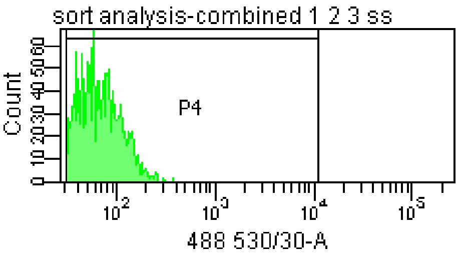

Experiment Name:	Bio RBD2 selectons
Specimen Name:	sort analysis
Tube Name:	combined 1 2 3 ss
Record Date:	May 26, 2020 3:29:59 PM
$OP:	Nileena
GUID:	6340c7e3-6308-4bcc-a3b1-2a650fae73b5

Population	Parent...	#Events	%Parent	%Gran...	%Total	488 5... Mean	488 5... Median	PE-A Mean	PE-A Median	APC-A Mean	APC-A Median
All Events	####	####	####	####	####	78	49	2,232	1,631	176	101
P2	####	####	####	####	####	76	51	2,248	1,661	155	104
P8	P2	3,790	38.1	36.7	36.7	19	16	1,591	1,306	85	68
Q1	P8	7	0.2	0.1	0.1	-22	-17	372	282	2,073	659
Q2	P8	69	1.8	0.7	0.7	60	74	4,041	3,637	583	566
Q3	P8	639	16.9	6.4	6.2	-4	-2	240	182	14	-6
Q4	P8	3,075	81.1	30.9	29.8	23	22	1,819	1,594	83	77
P7	P8	3,254	85.9	32.7	31.5	24	22	1,814	1,569	92	78
P1	P8	2,179	57.5	21.9	21.1	24	23	1,916	1,697	203	170
P4	P8	1,537	40.6	15.4	14.9	75	64	2,174	1,925	111	98

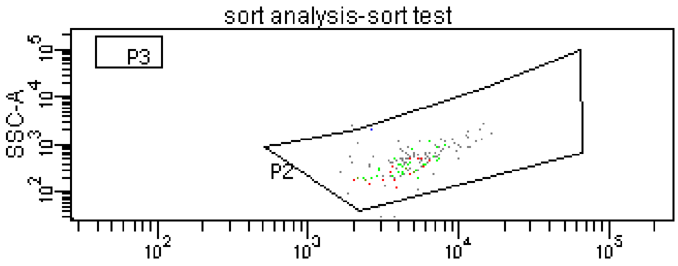

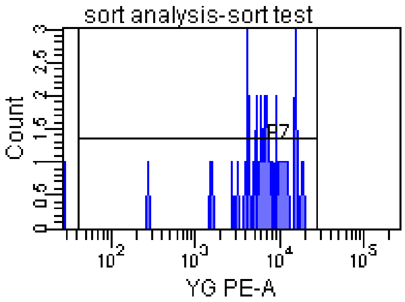

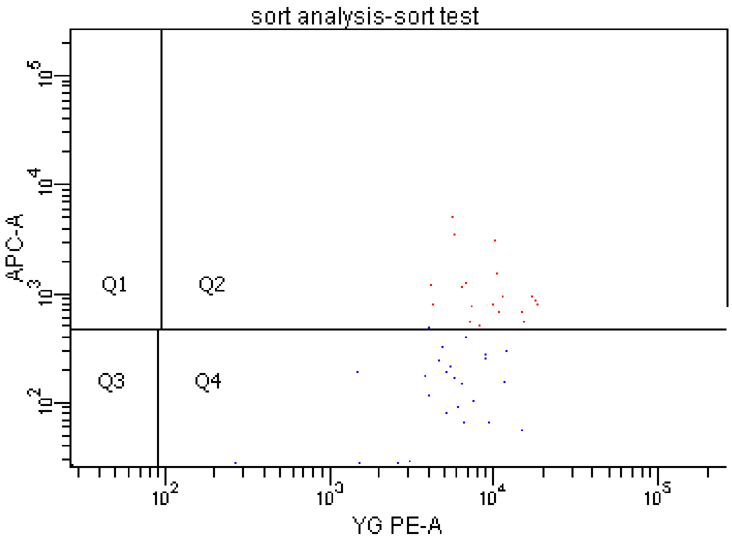

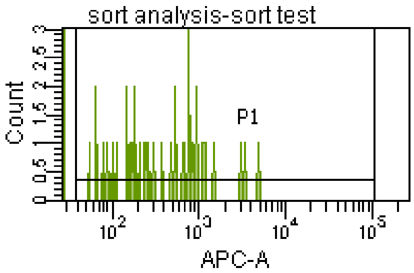

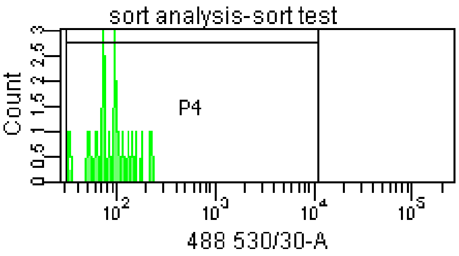

Experiment Name: Bio RBD2 selectons
Specimen Name: sort analysis
Tube Name: sort test
Record Date: May 26, 2020 4:46:00 PM
$OP: Nileena
GUID: fe8ff03d-bf93-4100-9045-e3b5710bee01

Population	Parent...	#Events	%Parent	%Gran...	%Total	488 5... Mean	488 5... Median	PE-A Mean	PE-A Median	APC-A Mean	APC-A Median
All Events	####	####	####	####	####	106	88	3,967	3,521	957	504
P2	####	####	####	####	####	111	95	4,006	3,559	649	495
P8	P2	46	34.1	32.9	32.9	47	51	3,222	2,729	627	297
Q1	P8	0	0.0	0.0	0.0	####	####	####	####	####	####
Q2	P8	20	43.5	14.8	14.3	60	56	4,247	4,125	1,256	804
Q3	P8	1	2.2	0.7	0.7	-38	-38	1,985	1,985	-114	-114
Q4	P8	25	54.3	18.5	17.9	39	50	2,451	2,450	153	148
P7	P8	45	97.8	33.3	32.1	49	52	3,249	2,754	643	304
P1	P8	41	89.1	30.4	29.3	56	61	3,483	2,971	709	471
P4	P8	26	56.5	19.3	18.6	99	92	3,682	2,979	565	423

FACSDiva Version 6.1.3

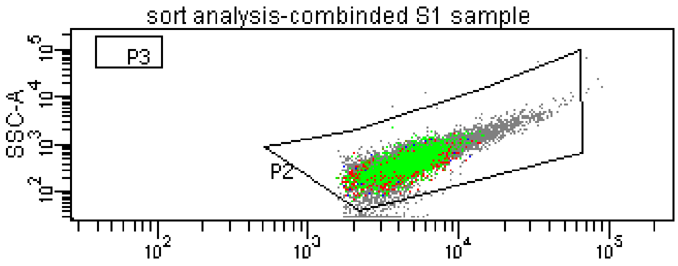

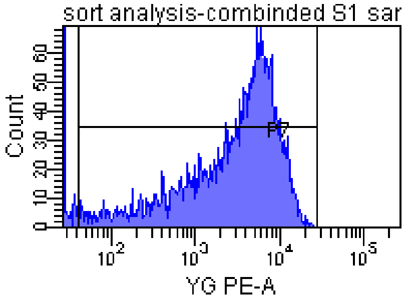

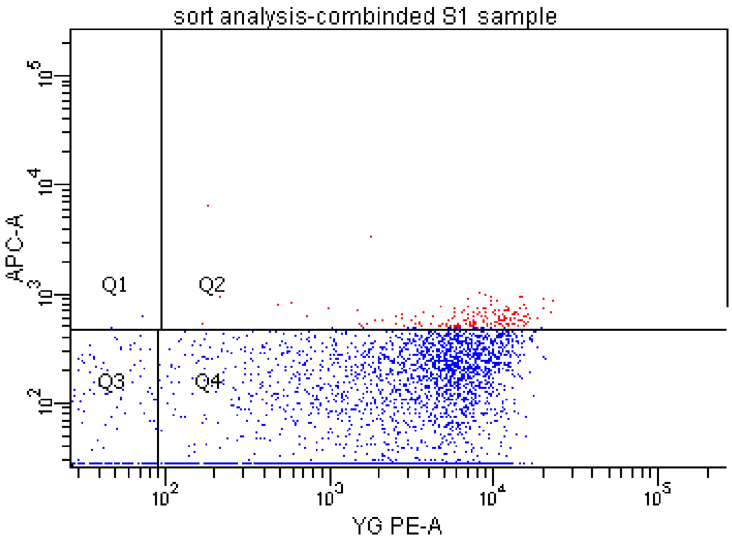

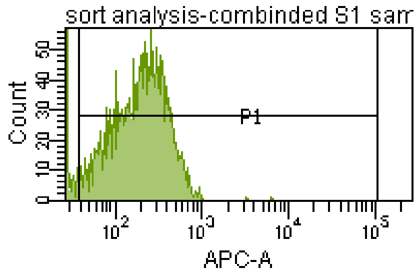

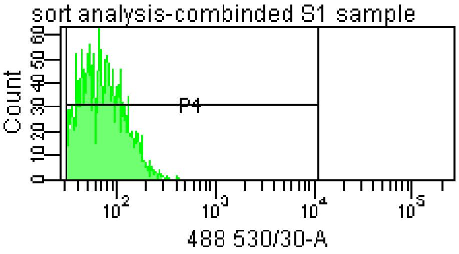

Experiment Name: Bio RBD2 selectons
Specimen Name: sort analysis
Tube Name: combinded S1 sample
Record Date: May 26, 2020 5:08:07 PM
$OP: Nileena
GUID: db9959f5-94fd-4032-b0c5-256fdad652ab

Population	Parent...	#Events	%Parent	%Gran...	%Total	488 5... Mean	488 5... Median	PE-A Mean	PE-A Median	APC-A Mean	APC-A Median
All Events	####	####	####	####	####	80	58	2,568	1,923	212	137
P2	####	####	####	####	####	82	61	2,583	1,959	190	139
P8	P2	3,871	38.9	37.8	37.8	24	21	1,807	1,410	116	95
Q1	P8	7	0.2	0.1	0.1	25	56	410	181	2,583	499
Q2	P8	165	4.3	1.7	1.6	69	71	4,027	3,793	645	566
Q3	P8	658	17.0	6.6	6.4	-7	-9	209	173	23	15
Q4	P8	3,041	78.6	30.5	29.7	28	26	2,036	1,782	102	101
P7	P8	3,296	85.1	33.1	32.2	30	27	2,087	1,799	127	110
P1	P8	2,396	61.9	24.1	23.4	30	28	2,183	1,933	238	199
P4	P8	1,689	43.6	17.0	16.5	80	69	2,536	2,338	160	131

FACSDiva Version 6.1.3

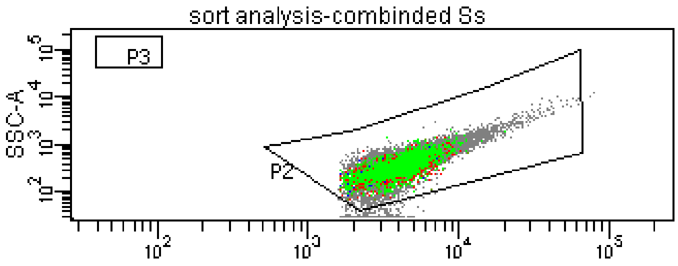

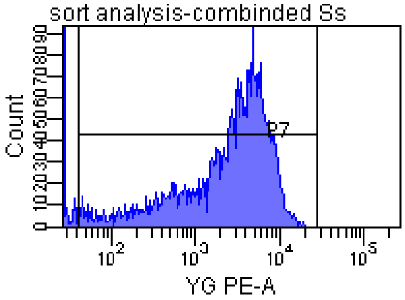

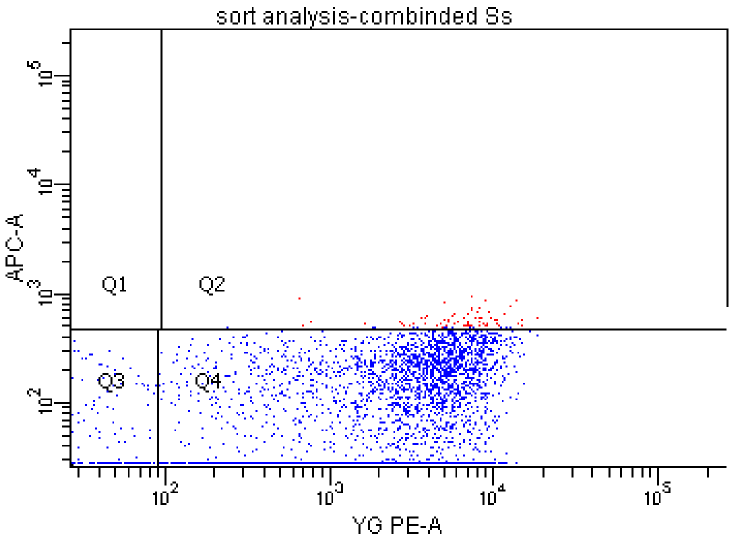

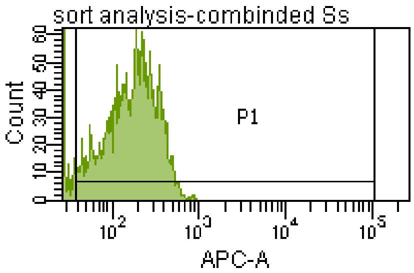

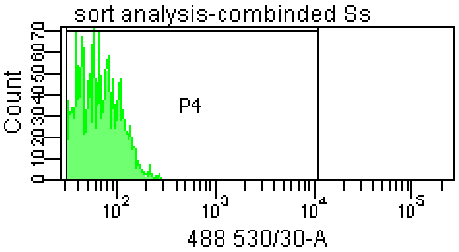

Experiment Name: Bio RBD2 selectons
Specimen Name: sort analysis
Tube Name: combinded Ss
Record Date: May 26, 2020 5:09:15 PM
$OP: Nileena
GUID: 08cbd93b-ee2a-468d-ac56-d3d28ca71780

Population	Parent...	#Events	%Parent	%Gran...	%Total	488 5... Mean	488 5... Median	PE-A Mean	PE-A Median	APC-A Mean	APC-A Median
All Events	####	####	####	####	####	59	40	1,910	1,461	130	97
P2	####	####	####	####	####	62	44	1,937	1,485	132	99
P8	P2	4,298	43.1	41.6	41.6	19	17	1,491	1,253	80	69
Q1	P8	3	0.1	0.0	0.0	-4	24	139	8	485	479
Q2	P8	68	1.6	0.7	0.7	53	46	3,313	3,346	560	524
Q3	P8	713	16.6	7.2	6.9	-4	-4	182	152	8	-1
Q4	P8	3,514	81.8	35.3	34.0	23	22	1,722	1,521	85	82
P7	P8	3,698	86.0	37.1	35.8	23	22	1,704	1,498	91	82
P1	P8	2,438	56.7	24.5	23.6	23	22	1,780	1,580	197	175
P4	P8	1,722	40.1	17.3	16.7	73	64	2,071	1,943	101	89

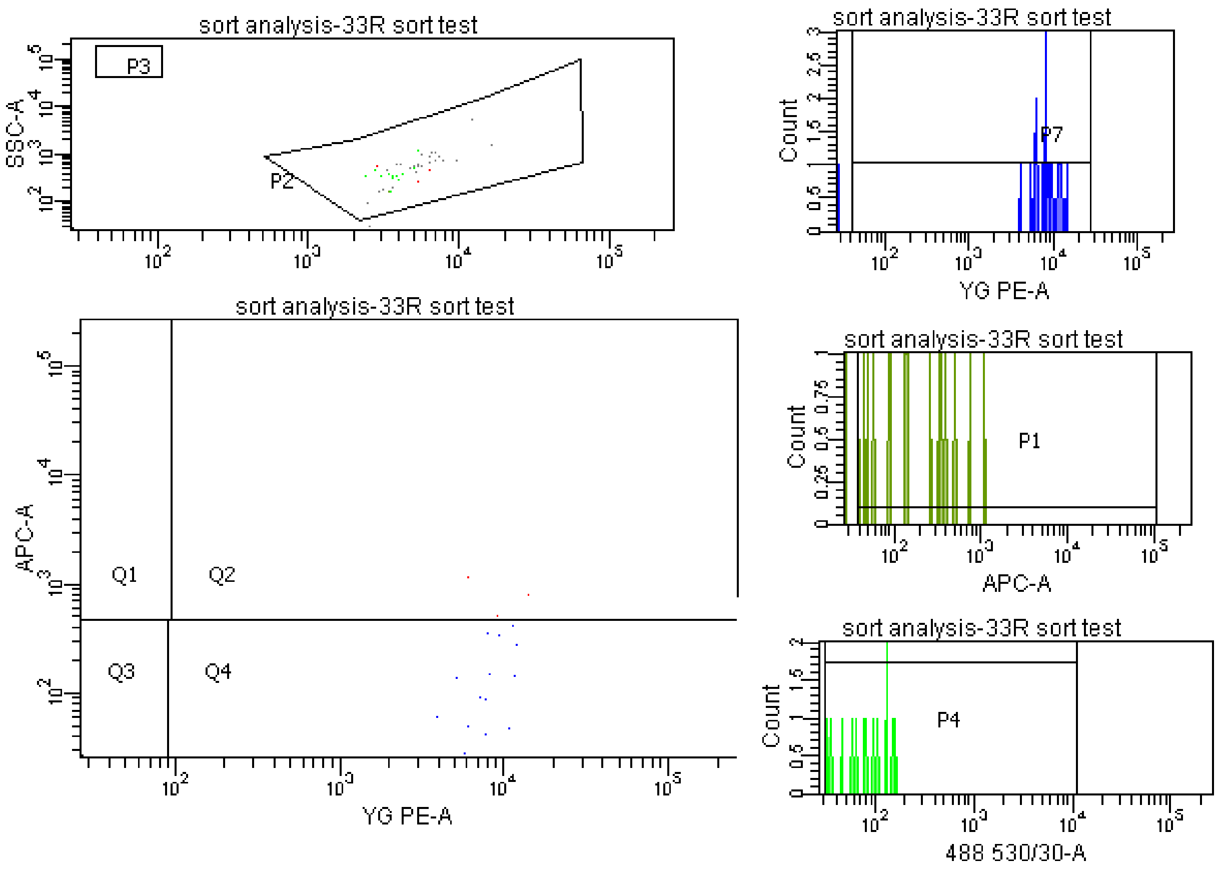

Experiment Name: Bio RBD2 selectons
Specimen Name: sort analysis
Tube Name: 33R sort test
Record Date: May 26, 2020 5:29:31 PM
$OP: Nileena
GUID: 8ae3a948-48d9-49af-bab0-8f6b026091e6

Population	Parent...	#Events	%Parent	%Gran...	%Total	488 5... Mean	488 5... Median	PE-A Mean	PE-A Median	APC-A Mean	APC-A Median
All Events	####	####	####	####	####	119	94	3,666	3,598	379	235
P2	####	####	####	####	####	120	90	3,729	3,610	390	244
P8	P2	18	42.9	41.9	41.9	55	59	3,324	3,321	237	132
Q1	P8	0	0.0	0.0	0.0	####	####	####	####	####	####
Q2	P8	3	16.7	7.1	7.0	94	81	4,214	3,535	781	749
Q3	P8	1	5.6	2.4	2.3	35	35	195	195	-58	-58
Q4	P8	14	77.8	33.3	32.6	48	59	3,356	3,321	142	109
P7	P8	17	94.4	40.5	39.5	56	61	3,508	3,366	255	135
P1	P8	16	88.9	38.1	37.2	58	68	3,584	3,399	277	138
P4	P8	13	72.2	31.0	30.2	88	81	3,312	3,275	250	88

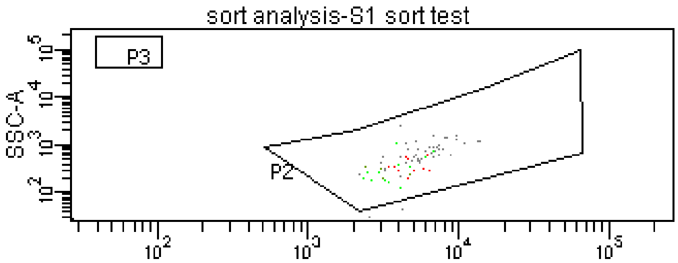

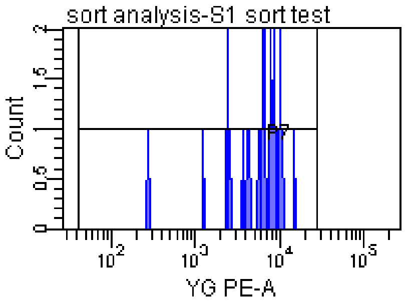

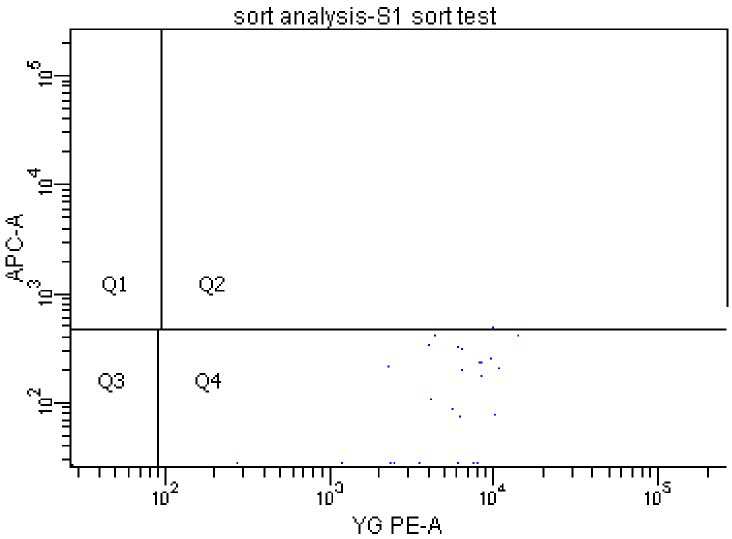

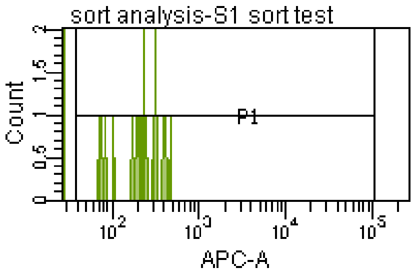

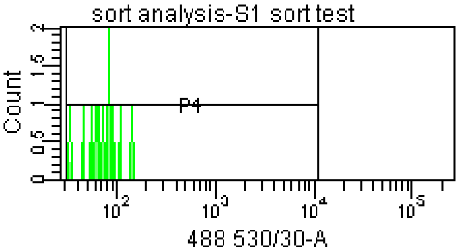

Experiment Name:	Bio RBD2 selectons
Specimen Name:	sort analysis
Tube Name:	S1 sort test
Record Date:	May 26, 2020 5:31:04 PM
$OP:	Nileena
GUID:	045a7c23-d579-4d9d-82b2-668bcf85efbc

Population	Parent...	#Events	%Parent	%Gran...	%Total	488 5... Mean	488 5... Median	PE-A Mean	PE-A Median	APC-A Mean	APC-A Median
All Events	####	####	####	####	####	80	71	3,404	3,056	228	207
P2	####	####	####	####	####	83	77	3,420	3,056	229	207
P8	P2	26	37.7	36.6	36.6	17	8	2,590	2,466	138	136
Q1	P8	0	0.0	0.0	0.0	####	####	####	####	####	####
Q2	P8	0	0.0	0.0	0.0	####	####	####	####	####	####
Q3	P8	0	0.0	0.0	0.0	####	####	####	####	####	####
Q4	P8	26	100.0	37.7	36.6	17	8	2,590	2,466	138	136
P7	P8	26	100.0	37.7	36.6	17	8	2,590	2,466	138	136
P1	P8	17	65.4	24.6	23.9	10	-1	2,948	2,996	232	224
P4	P8	12	46.2	17.4	16.9	75	68	3,003	3,053	128	78

FACSDiva Version 6.1.3

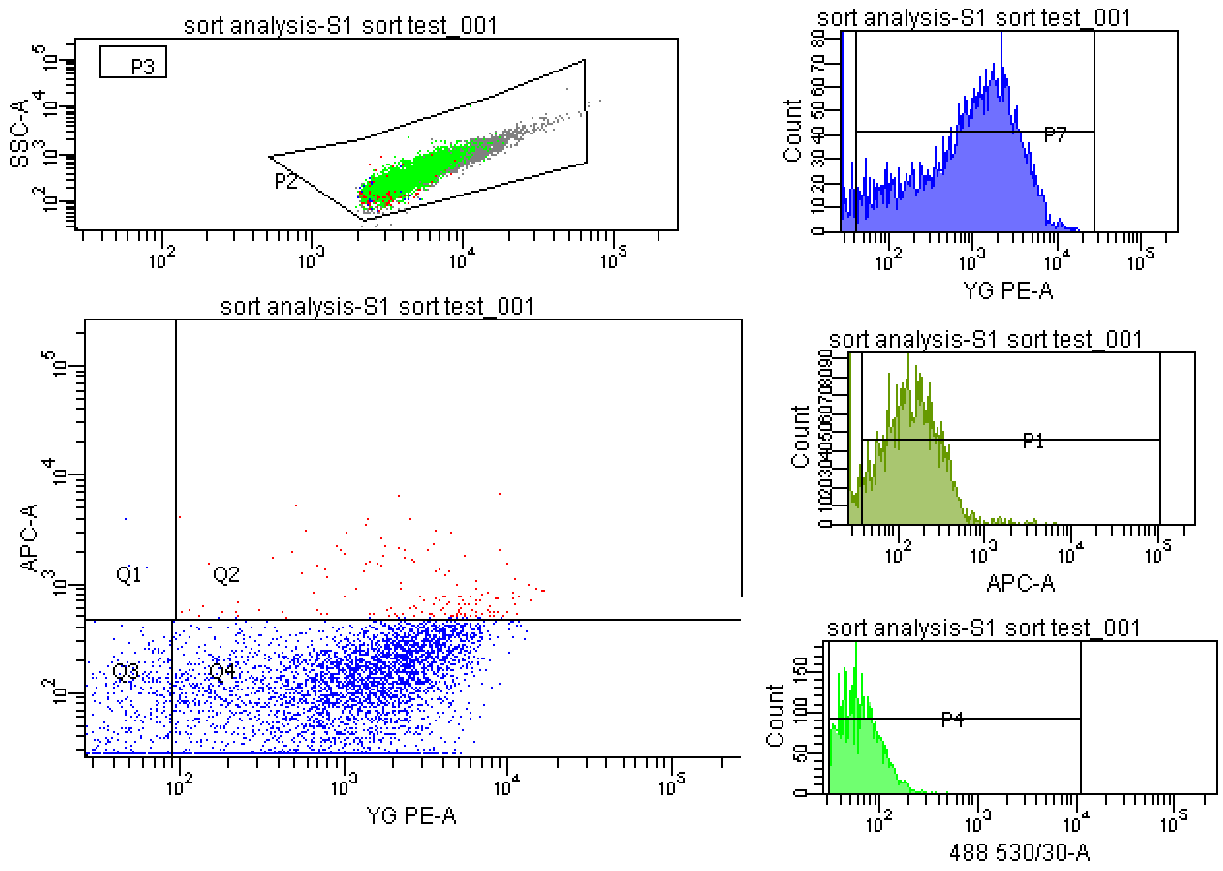

Experiment Name:	Bio RBD2 selectons
Specimen Name:	sort analysis
Tube Name:	S1 sort test_001
Record Date:	May 28, 2020 3:31:54 PM
$OP:	Nileena
GUID:	08633170-5929-4f52-89b4-288d1493efb5

Population	Parent...	#Events	%Parent	%Gran...	%Total	488 5... Mean	488 5... Median	PE-A Mean	PE-A Median	APC-A Mean	APC-A Median
All Events	####	####	####	####	####	78	59	1,792	1,334	237	121
P2	####	####	####	####	####	77	59	1,788	1,335	237	121
P8	P2	6,162	61.7	61.6	61.6	49	41	1,151	818	130	81
Q1	P8	10	0.2	0.1	0.1	18	16	87	68	3,664	2,795
Q2	P8	145	2.4	1.5	1.4	115	99	3,166	2,977	1,100	682
Q3	P8	1,732	28.1	17.3	17.3	19	17	51	38	37	26
Q4	P8	4,275	69.4	42.8	42.7	58	53	1,530	1,359	126	106
P7	P8	4,746	77.0	47.5	47.4	58	51	1,483	1,269	153	107
P1	P8	4,128	67.0	41.3	41.2	58	52	1,474	1,298	196	131
P4	P8	3,812	61.9	38.1	38.1	70	61	1,671	1,543	168	119

FACSDiva Version 6.1.3

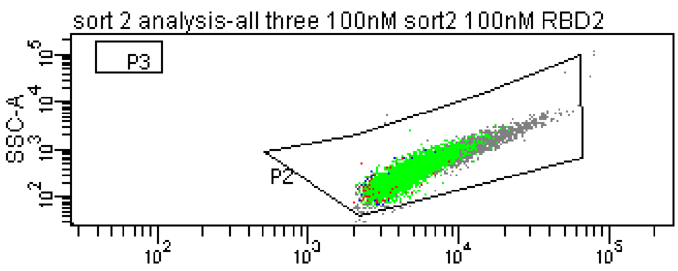

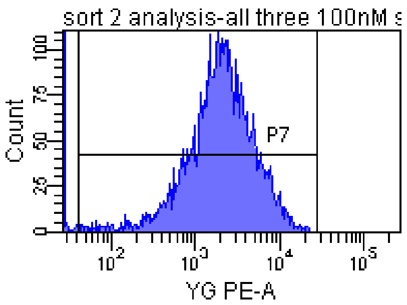

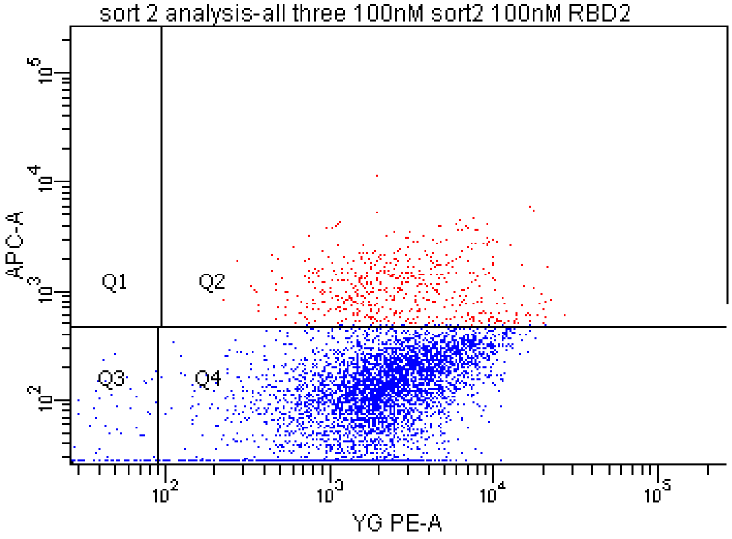

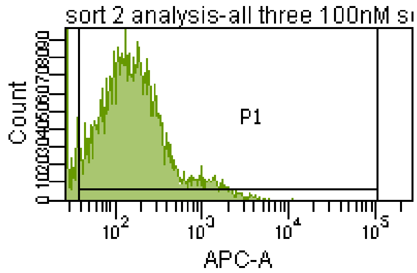

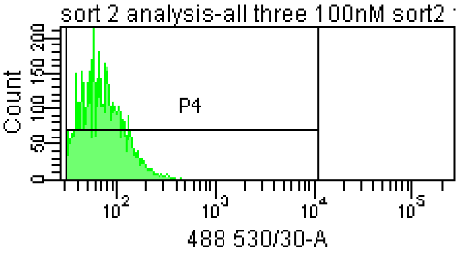

Experiment Name: Bio RBD2 selectons
Specimen Name: sort 2 analysis
Tube Name: all three 100nM sort2 100nM RBD2
Record Date: May 29, 2020 2:41:31 PM
$OP: Nileena
GUID: 2f2c5ec5-40bf-4f35-83d9-f8e419ee8f82

Population	Parent...	#Events	%Parent	%Gran...	%Total	488 5... Mean	488 5... Median	PE-A Mean	PE-A Median	APC-A Mean	APC-A Median
All Events	####	####	####	####	####	105	77	3,214	2,454	412	153
P2	####	####	####	####	####	104	77	3,203	2,459	369	153
P8	P2	6,026	60.3	60.2	60.2	65	57	2,083	1,866	217	106
Q1	P8	7	0.1	0.1	0.1	16	0	31	26	5,079	3,037
Q2	P8	505	8.4	5.1	5.0	97	80	3,208	2,481	1,284	999
Q3	P8	809	13.4	8.1	8.1	17	15	40	31	20	14
Q4	P8	4,705	78.1	47.1	47.0	70	61	2,317	2,068	130	111
P7	P8	5,263	87.3	52.7	52.5	72	62	2,378	2,081	239	123
P1	P8	4,499	74.7	45.0	44.9	75	65	2,476	2,207	292	148
P4	P8	4,574	75.9	45.8	45.7	81	69	2,564	2,269	249	132

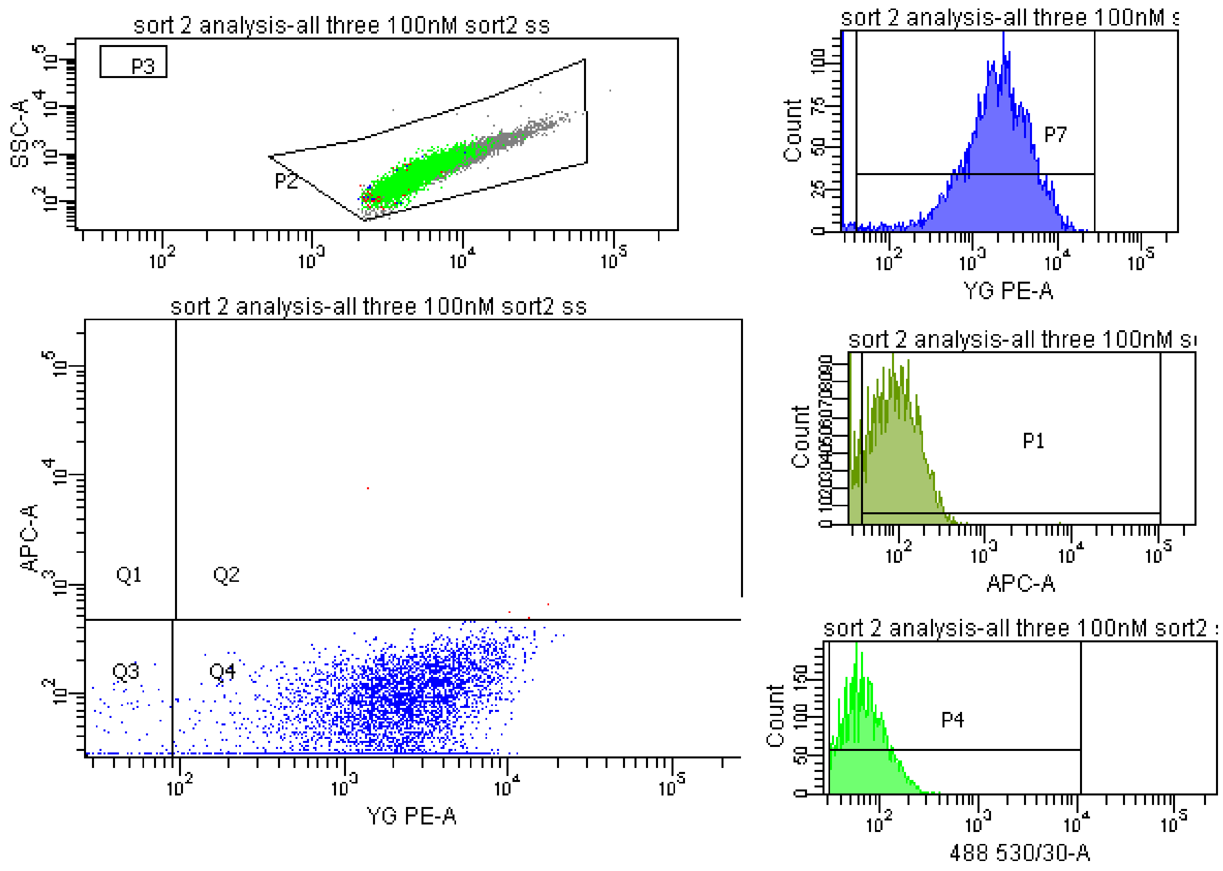

Experiment Name: Bio RBD2 selectons
Specimen Name: sort 2 analysis
Tube Name: all three 100nM sort2 ss
Record Date: May 29, 2020 2:43:40 PM
$OP: Nileena
GUID: 74dc275a-c84c-45ae-bf20-548ab4d3d643

Population	Parent...	#Events	%Parent	%Gran...	%Total	488 5... Mean	488 5... Median	PE-A Mean	PE-A Median	APC-A Mean	APC-A Median
All Events	####	####	####	####	####	101	75	2,959	2,346	187	79
P2	####	####	####	####	####	101	75	2,961	2,348	177	79
P8	P2	6,084	60.9	60.8	60.8	63	56	1,951	1,789	65	52
Q1	P8	0	0.0	0.0	0.0	####	####	####	####	####	####
Q2	P8	4	0.1	0.0	0.0	202	196	6,152	7,191	2,254	572
Q3	P8	817	13.4	8.2	8.2	17	14	40	29	22	14
Q4	P8	5,263	86.5	52.7	52.6	69	61	2,244	2,009	70	59
P7	P8	5,316	87.4	53.2	53.1	69	61	2,227	1,995	72	58
P1	P8	3,523	57.9	35.2	35.2	76	68	2,391	2,177	114	95
P4	P8	4,556	74.9	45.6	45.5	79	68	2,412	2,199	76	65

FACSDiva Version 6.1.3

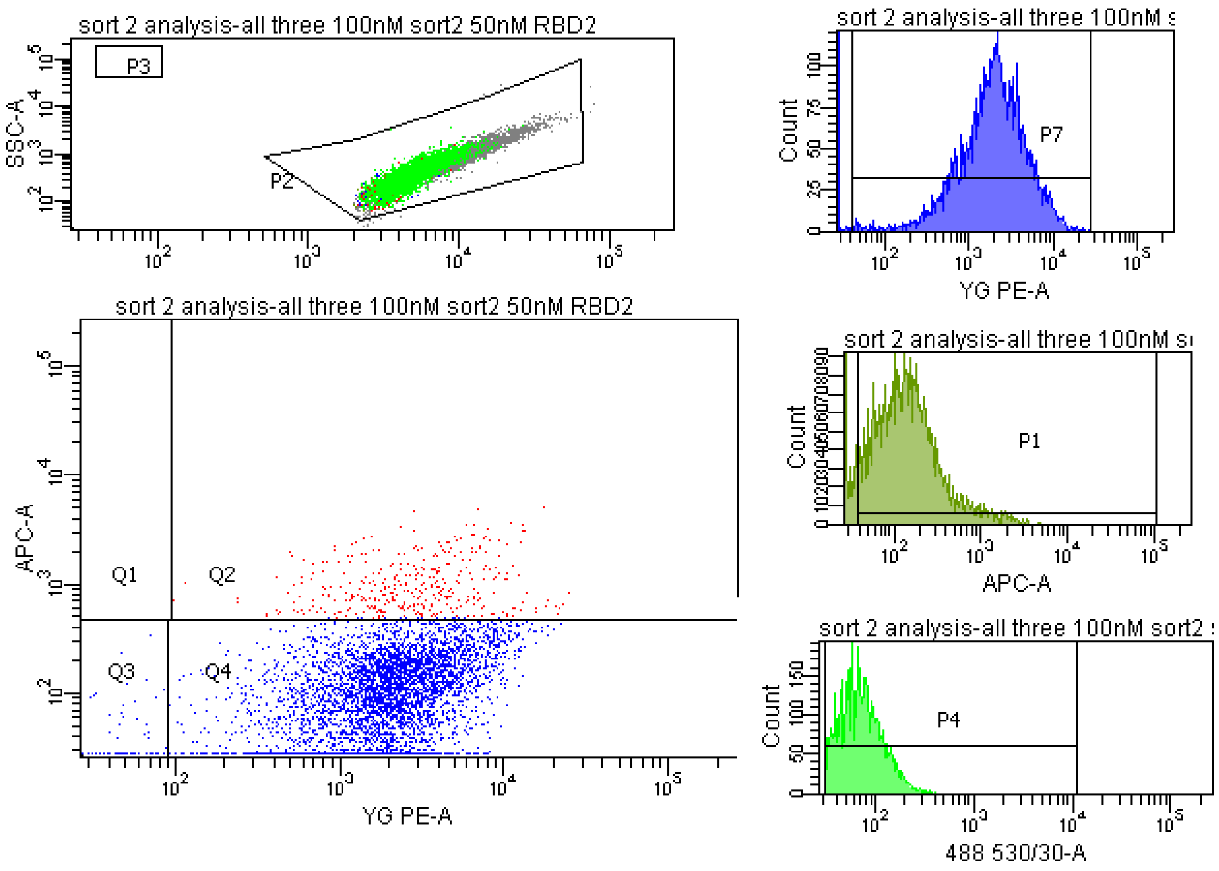

Experiment Name: Bio RBD2 selectons
Specimen Name: sort 2 analysis
Tube Name: all three 100nM sort2 50nM RBD2
Record Date: May 29, 2020 2:45:38 PM
$OP: Nileena
GUID: ce320125-8b06-451a-bb85-d123e7927f88

Population	Parent...	#Events	%Parent	%Gran...	%Total	488 5... Mean	488 5... Median	PE-A Mean	PE-A Median	APC-A Mean	APC-A Median
All Events	####	####	####	####	####	101	74	2,989	2,342	287	124
P2	####	####	####	####	####	99	74	2,985	2,344	256	124
P8	P2	6,147	61.5	61.4	61.4	63	56	1,997	1,842	155	85
Q1	P8	1	0.0	0.0	0.0	64	64	324	324	4,554	4,554
Q2	P8	359	5.8	3.6	3.6	94	78	3,084	2,568	1,099	821
Q3	P8	810	13.2	8.1	8.1	17	14	41	33	23	17
Q4	P8	4,977	81.0	49.8	49.7	68	61	2,237	2,018	108	90
P7	P8	5,384	87.6	53.9	53.8	70	61	2,275	2,035	173	98
P1	P8	4,301	70.0	43.0	42.9	73	64	2,361	2,141	225	130
P4	P8	4,615	75.1	46.2	46.1	79	69	2,465	2,230	184	105

FACSDiva Version 6.1.3

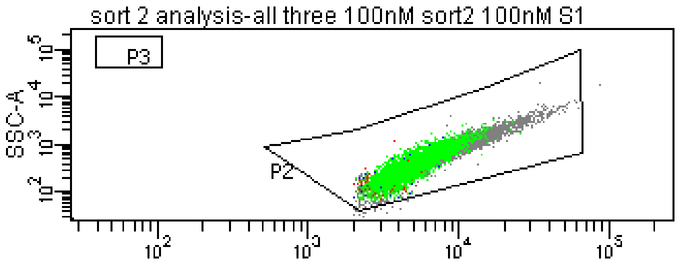

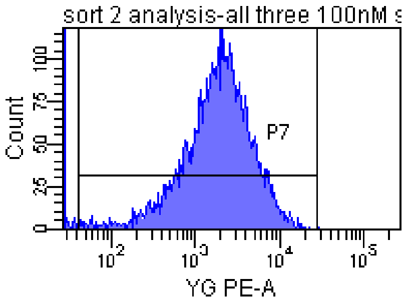

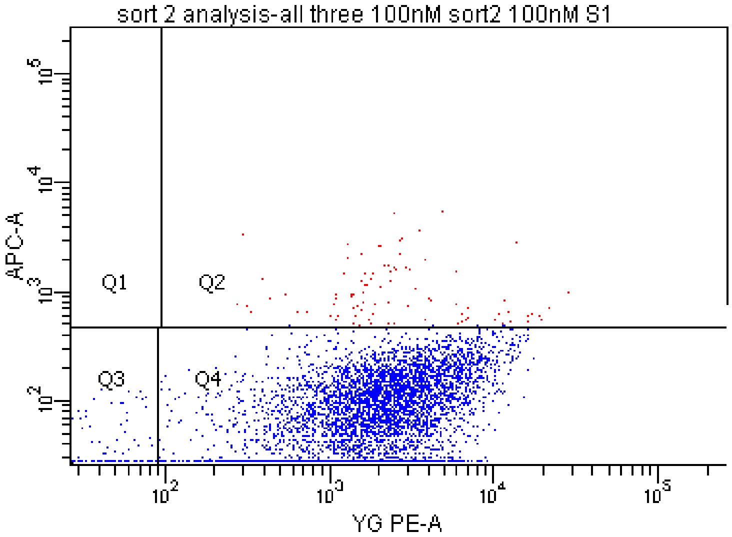

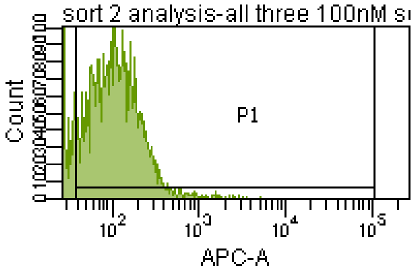

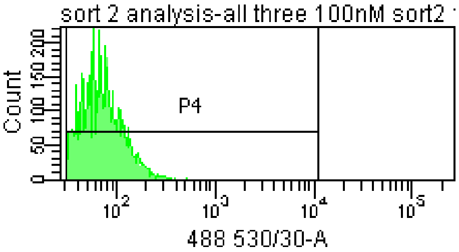

Experiment Name: Bio RBD2 selectons
Specimen Name: sort 2 analysis
Tube Name: all three 100nM sort2 100nM S1
Record Date: May 29, 2020 2:47:11 PM
$OP: Nileena
GUID: ccba5184-80d9-4b6e-ab4f-e5ab49cffc74

Population	Parent...	#Events	%Parent	%Gran...	%Total	488 5... Mean	488 5... Median	PE-A Mean	PE-A Median	APC-A Mean	APC-A Median
All Events	####	####	####	####	####	97	72	2,924	2,317	185	91
P2	####	####	####	####	####	97	72	2,930	2,324	164	91
P8	P2	6,299	63.1	62.9	62.9	62	54	1,954	1,819	91	62
Q1	P8	3	0.0	0.0	0.0	9	-3	62	89	2,603	1,360
Q2	P8	89	1.4	0.9	0.9	108	68	3,198	2,020	1,170	788
Q3	P8	824	13.1	8.3	8.2	16	14	42	31	19	13
Q4	P8	5,383	85.5	53.9	53.7	68	61	2,228	2,032	83	69
P7	P8	5,530	87.8	55.4	55.2	68	60	2,218	2,020	100	70
P1	P8	3,916	62.2	39.2	39.1	74	65	2,392	2,199	149	106
P4	P8	4,674	74.2	46.8	46.7	78	68	2,443	2,228	109	77

FACSDiva Version 6.1.3

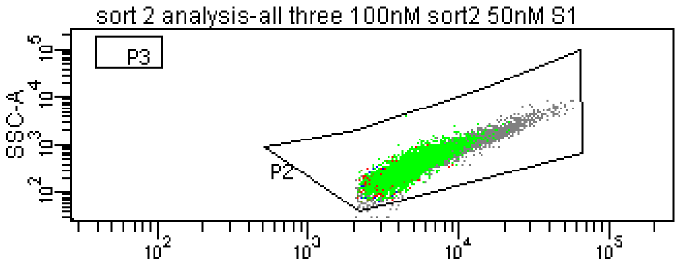

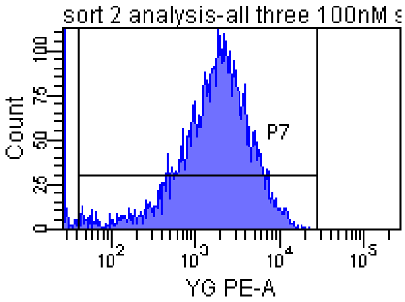

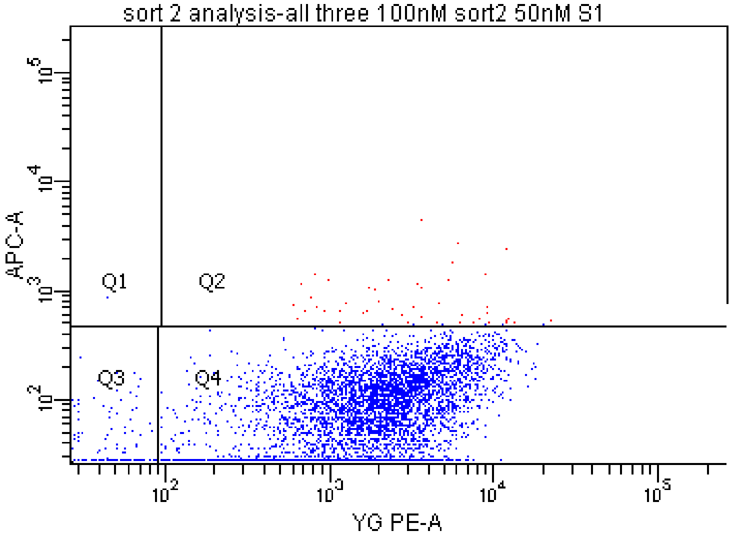

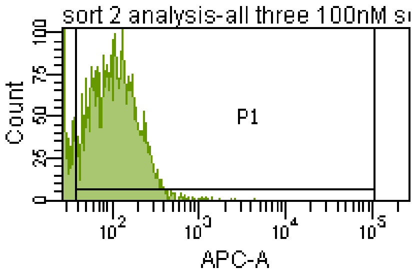

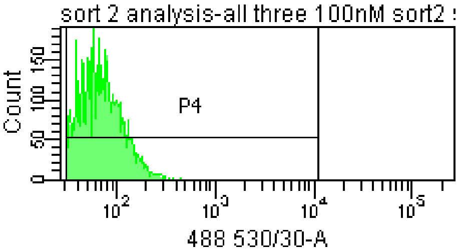

Experiment Name: Bio RBD2 selectons
Specimen Name: sort 2 analysis
Tube Name: all three 100nM sort2 50nM S1
Record Date: May 29, 2020 2:48:54 PM
$OP: Nileena
GUID: 9ef53d87-837f-47a2-8fdf-9bb0a7277c77

Population	Parent...	#Events	%Parent	%Gran...	%Total	488 5... Mean	488 5... Median	PE-A Mean	PE-A Median	APC-A Mean	APC-A Median
All Events	####	####	####	####	####	93	70	2,719	2,134	156	84
P2	####	####	####	####	####	93	70	2,723	2,137	156	84
P8	P2	6,355	63.6	63.5	63.5	59	51	1,820	1,658	77	57
Q1	P8	4	0.1	0.0	0.0	22	25	78	82	1,756	1,946
Q2	P8	45	0.7	0.5	0.4	113	86	3,562	2,604	906	658
Q3	P8	949	14.9	9.5	9.5	17	15	43	36	19	11
Q4	P8	5,357	84.3	53.6	53.5	66	59	2,121	1,884	79	65
P7	P8	5,492	86.4	55.0	54.9	66	58	2,100	1,867	85	65
P1	P8	3,790	59.6	38.0	37.9	72	63	2,267	2,063	132	104
P4	P8	4,576	72.0	45.8	45.7	77	66	2,320	2,099	92	72

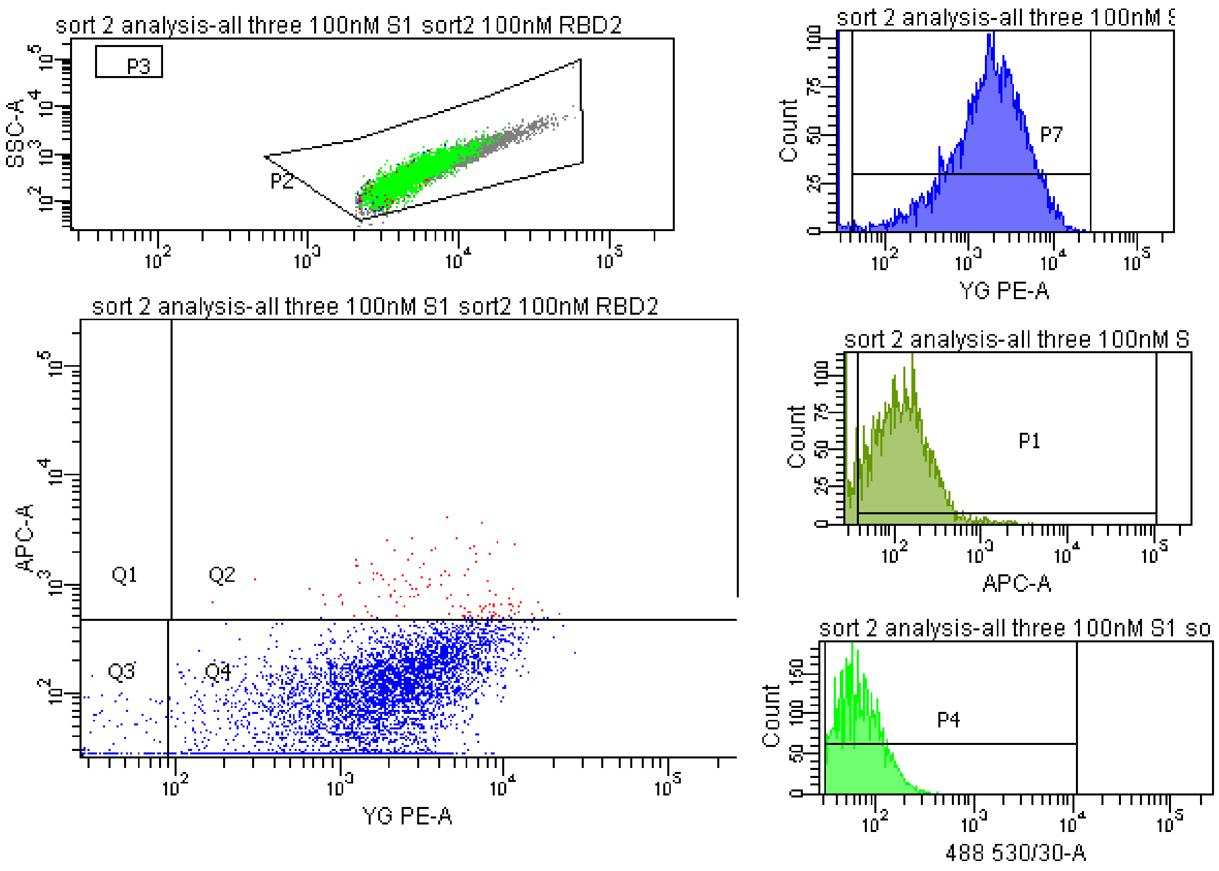

Experiment Name: Bio RBD2 selectons
Specimen Name: sort 2 analysis
Tube Name: all three 100nM S1 sort2 100nM RBD2
Record Date: May 29, 2020 2:50:41 PM
$OP: Nileena
GUID: 9d31f891-cc5b-4d0e-a93f-6e9b3a68f902

Population	Parent...	#Events	%Parent	%Gran...	%Total	488 5... Mean	488 5... Median	PE-A Mean	PE-A Median	APC-A Mean	APC-A Median
All Events	####	####	####	####	####	94	69	2,751	2,090	190	106
P2	####	####	####	####	####	94	69	2,752	2,094	190	106
P8	P2	6,405	64.1	64.0	64.0	60	51	1,832	1,616	112	76
Q1	P8	3	0.0	0.0	0.0	19	8	33	48	1,857	1,993
Q2	P8	121	1.9	1.2	1.2	126	112	3,985	3,368	975	746
Q3	P8	904	14.1	9.0	9.0	19	16	42	32	18	13
Q4	P8	5,377	84.0	53.8	53.7	65	57	2,085	1,842	107	88
P7	P8	5,555	86.7	55.6	55.5	66	57	2,104	1,848	125	89
P1	P8	4,344	67.8	43.5	43.4	72	63	2,279	2,061	164	119
P4	P8	4,560	71.2	45.7	45.5	78	66	2,353	2,125	140	101

FACSDiva Version 6.1.3

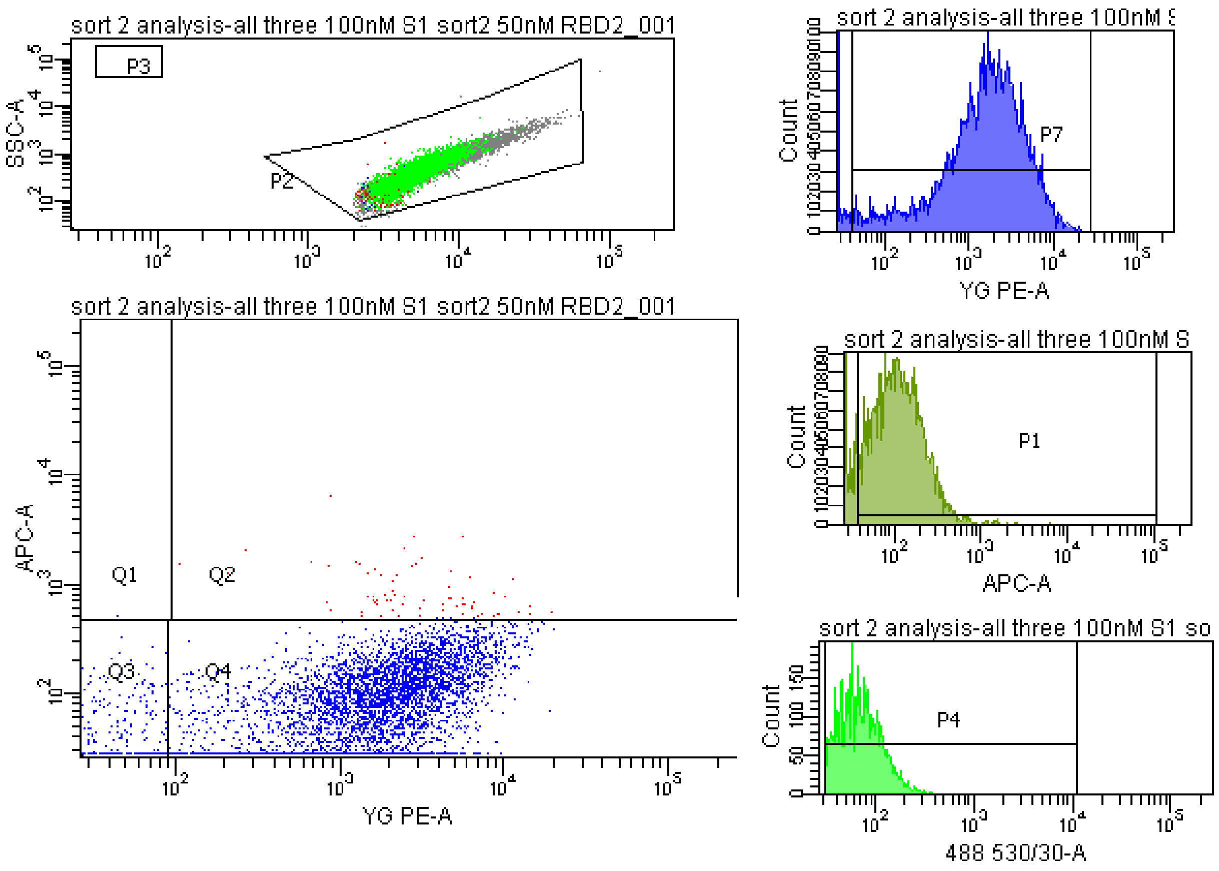

Experiment Name: Bio RBD2 selectons
Specimen Name: sort 2 analysis
Tube Name: all three 100nM S1 sort2 50nM RBD2_001
Record Date: May 29, 2020 2:52:03 PM
$OP: Nileena
GUID: 7bdf8ec7-2216-47ad-81c9-f3afdf5cfde6

Population	Parent...	#Events	%Parent	%Gran...	%Total	488 5... Mean	488 5... Median	PE-A Mean	PE-A Median	APC-A Mean	APC-A Median
All Events	####	####	####	####	####	88	66	2,472	1,918	196	90
P2	####	####	####	####	####	88	66	2,469	1,918	155	90
P8	P2	6,326	63.3	63.2	63.2	57	49	1,674	1,449	96	63
Q1	P8	4	0.1	0.0	0.0	16	19	85	63	10,912	2,800
Q2	P8	71	1.1	0.7	0.7	101	72	3,225	2,317	958	670
Q3	P8	1,130	17.9	11.3	11.3	17	14	47	40	25	17
Q4	P8	5,121	81.0	51.3	51.2	65	57	2,012	1,749	91	75
P7	P8	5,350	84.6	53.6	53.5	64	57	1,972	1,717	102	75
P1	P8	3,993	63.1	40.0	39.9	70	62	2,120	1,905	152	106
P4	P8	4,286	67.8	42.9	42.8	77	66	2,241	2,051	120	86

FACSDiva Version 6.1.3

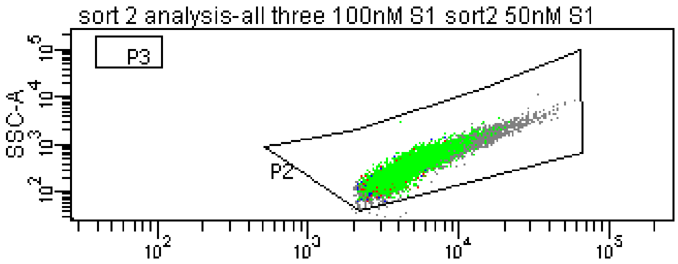

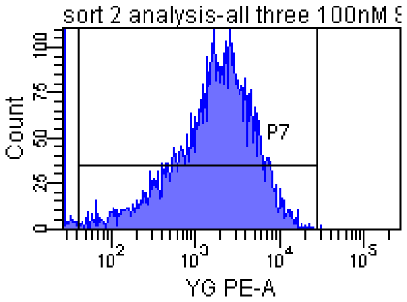

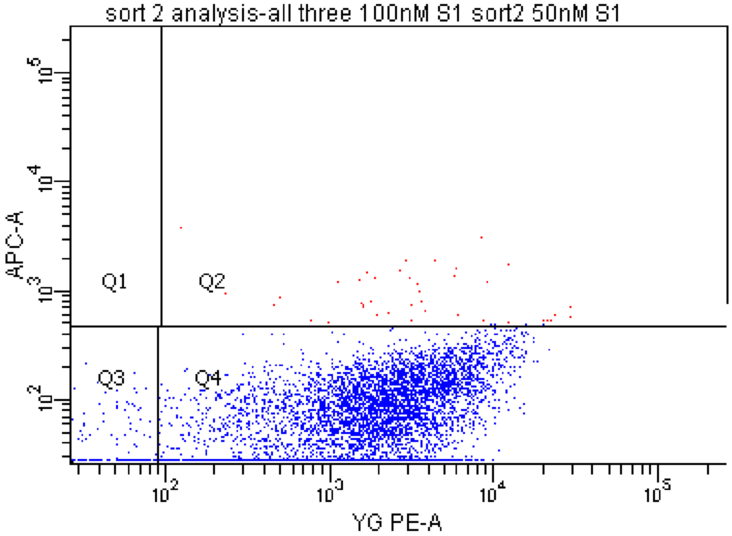

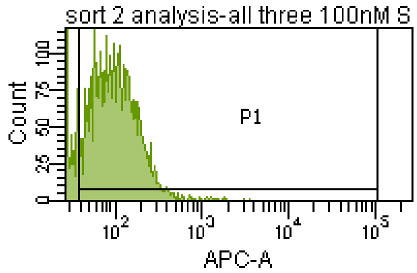

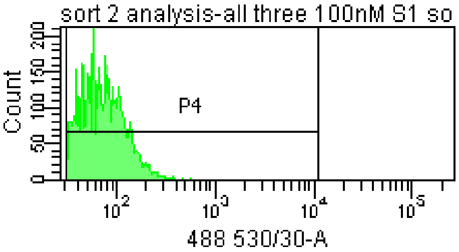

Experiment Name: Bio RBD2 selectons
Specimen Name: sort 2 analysis
Tube Name: all three 100nM S1 sort2 50nM S1
Record Date: May 29, 2020 2:53:20 PM
$OP: Nileena
GUID: 8a69e46c-02b8-4cb4-a0a8-d80fb5228d10

Population	Parent...	#Events	%Parent	%Gran...	%Total	488 5... Mean	488 5... Median	PE-A Mean	PE-A Median	APC-A Mean	APC-A Median
All Events	####	####	####	####	####	87	64	2,631	2,055	130	70
P2	####	####	####	####	####	87	64	2,634	2,059	130	70
P8	P2	7,002	70.1	69.9	69.9	61	51	1,896	1,631	70	52
Q1	P8	1	0.0	0.0	0.0	14	14	94	94	1,339	1,339
Q2	P8	40	0.6	0.4	0.4	132	98	4,038	2,266	1,018	742
Q3	P8	1,054	15.1	10.5	10.5	17	14	39	32	18	10
Q4	P8	5,907	84.4	59.1	59.0	68	59	2,213	1,962	73	61
P7	P8	6,035	86.2	60.4	60.3	67	59	2,190	1,936	78	61
P1	P8	4,089	58.4	40.9	40.8	75	65	2,424	2,194	122	95
P4	P8	4,927	70.4	49.3	49.2	80	69	2,497	2,274	88	68

FACSDiva Version 6.1.3

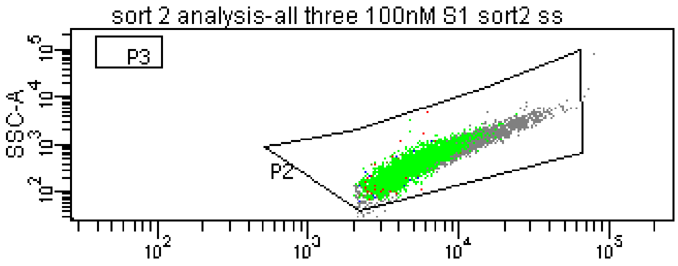

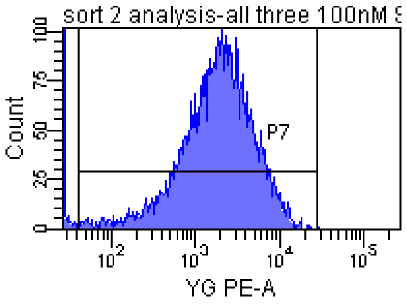

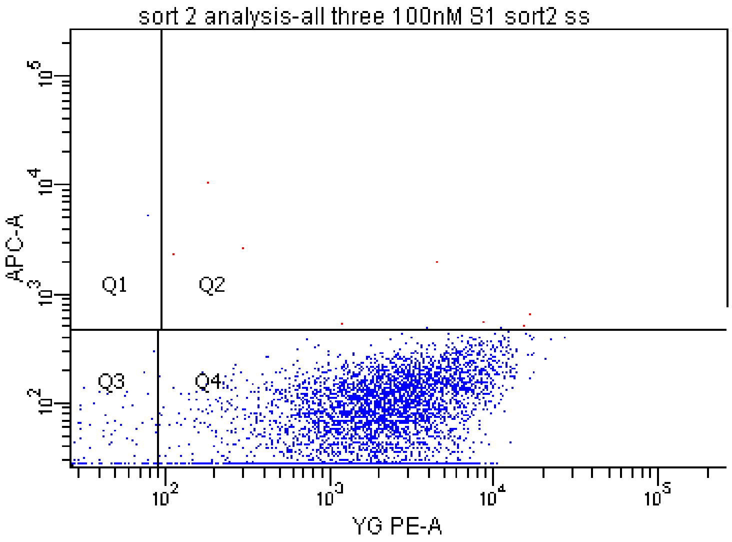

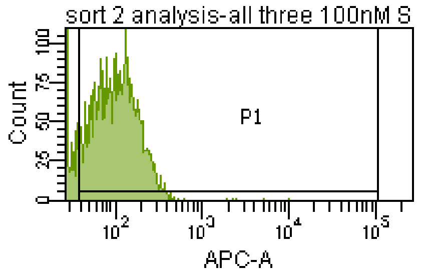

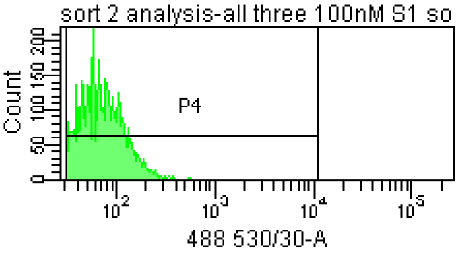

Experiment Name: Bio RBD2 selectons
Specimen Name: sort 2 analysis
Tube Name: all three 100nM S1 sort2 ss
Record Date: May 29, 2020 2:54:31 PM
$OP: Nileena
GUID: 18eb439f-2d5f-4156-8349-6104911ce4b9

Population	Parent...	#Events	%Parent	%Gran...	%Total	488 5... Mean	488 5... Median	PE-A Mean	PE-A Median	APC-A Mean	APC-A Median
All Events	####	####	####	####	####	99	73	2,933	2,287	118	79
P2	####	####	####	####	####	99	73	2,927	2,290	116	79
P8	P2	6,222	62.3	62.1	62.1	62	53	1,935	1,722	70	55
Q1	P8	6	0.1	0.1	0.1	10	8	132	87	2,675	2,465
Q2	P8	8	0.1	0.1	0.1	223	209	3,675	3,055	2,346	1,258
Q3	P8	920	14.8	9.2	9.2	19	16	42	34	20	14
Q4	P8	5,288	85.0	52.9	52.8	70	60	2,264	2,007	73	62
P7	P8	5,353	86.0	53.6	53.5	69	60	2,241	1,988	77	62
P1	P8	3,662	58.9	36.6	36.6	75	65	2,384	2,167	125	100
P4	P8	4,573	73.5	45.8	45.7	79	68	2,437	2,215	80	68

FACSDiva Version 6.1.3

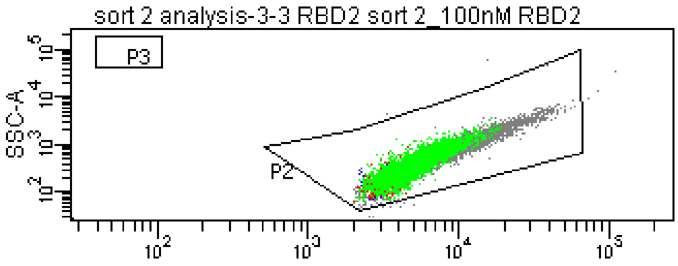

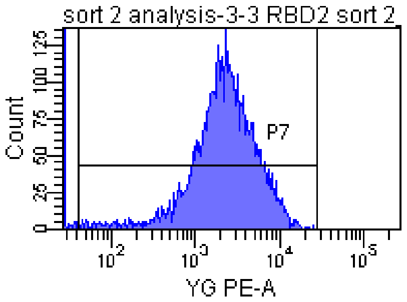

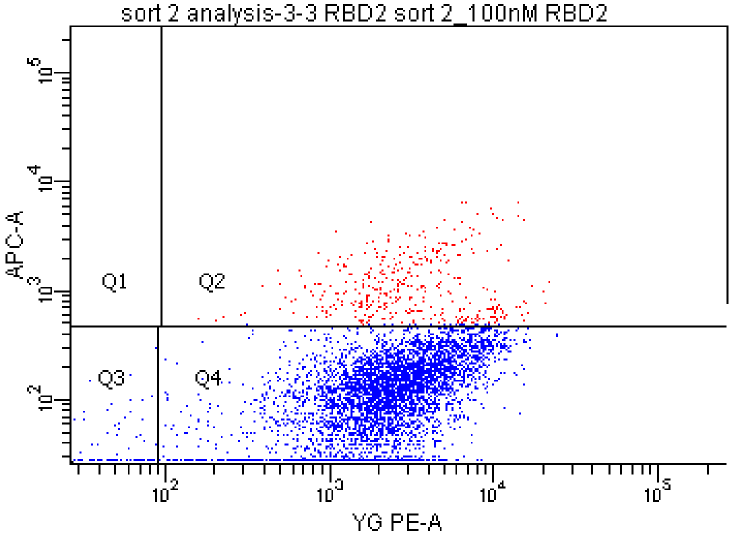

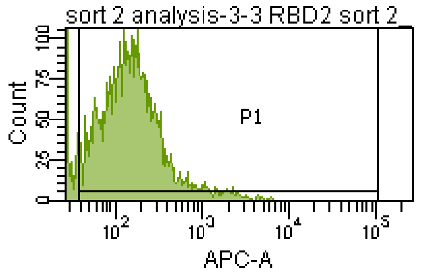

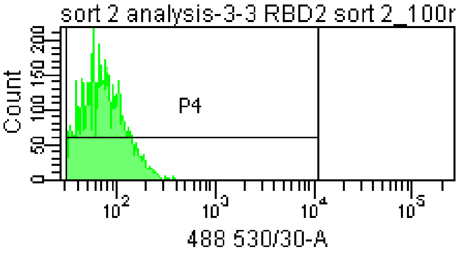

Experiment Name: Bio RBD2 selectons
Specimen Name: sort 2 analysis
Tube Name: 3-3 RBD2 sort 2_100nM RBD2
Record Date: May 29, 2020 2:56:03 PM
$OP: Nileena
GUID: 06feec8e-35f4-4df7-9d47-6593eec6c4fc

Population	Parent...	#Events	%Parent	%Gran...	%Total	488 5... Mean	488 5... Median	PE-A Mean	PE-A Median	APC-A Mean	APC-A Median
All Events	####	####	####	####	####	106	78	3,169	2,487	361	146
P2	####	####	####	####	####	105	78	3,160	2,488	319	146
P8	P2	6,302	63.0	62.9	62.9	68	60	2,141	2,012	186	103
Q1	P8	1	0.0	0.0	0.0	29	29	11	11	2,503	2,503
Q2	P8	391	6.2	3.9	3.9	95	74	3,155	2,520	1,296	948
Q3	P8	823	13.1	8.2	8.2	19	17	44	34	19	11
Q4	P8	5,087	80.7	50.9	50.8	74	66	2,403	2,173	128	112
P7	P8	5,535	87.8	55.4	55.3	75	66	2,433	2,178	209	119
P1	P8	4,708	74.7	47.1	47.0	78	70	2,527	2,304	250	142
P4	P8	4,994	79.2	50.0	49.9	82	71	2,549	2,306	217	124

FACSDiva Version 6.1.3

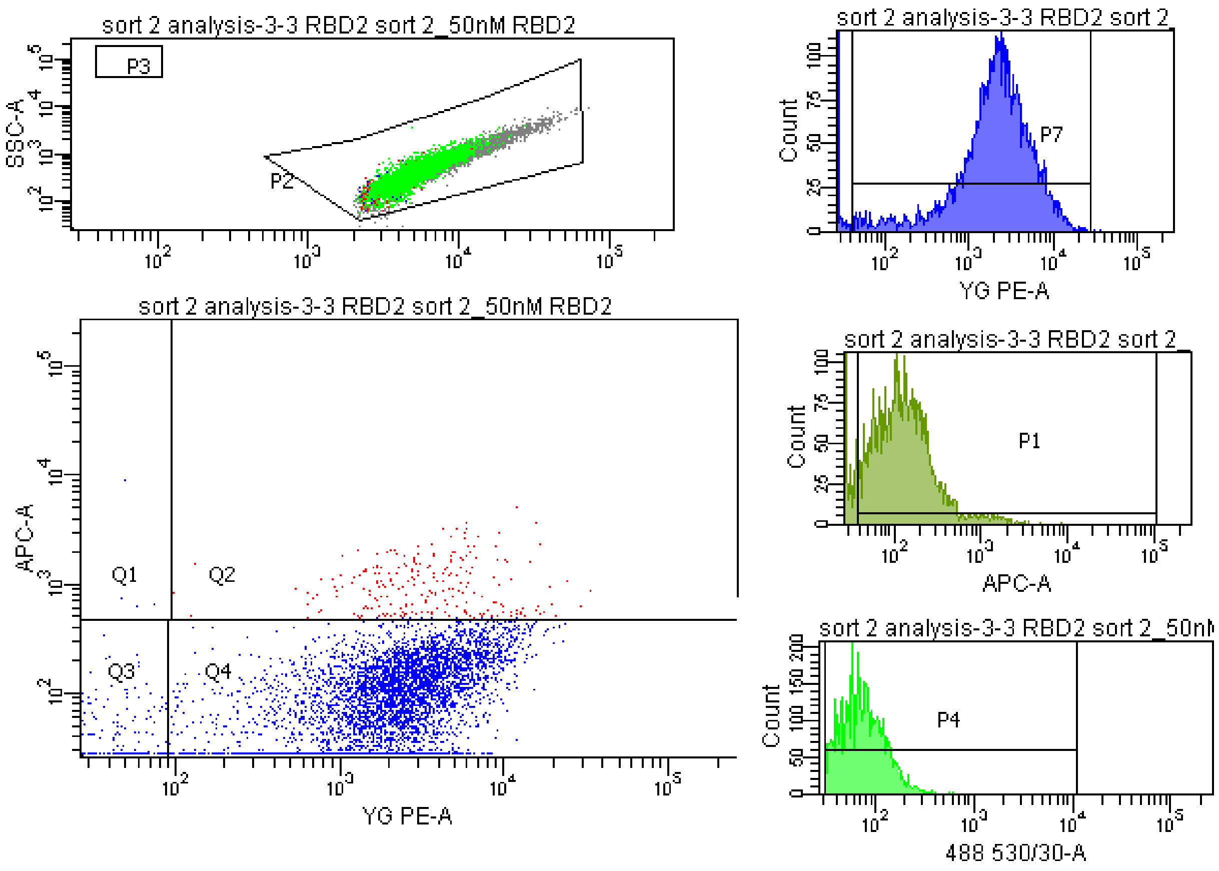

Experiment Name: Bio RBD2 selectons
Specimen Name: sort 2 analysis
Tube Name: 3-3 RBD2 sort 2_50nM RBD2
Record Date: May 29, 2020 2:57:18 PM
$OP: Nileena
GUID: 26e183f6-3ddd-48d7-833d-5d0718ed9ab0

Population	Parent...	#Events	%Parent	%Gran...	%Total	488 5... Mean	488 5... Median	PE-A Mean	PE-A Median	APC-A Mean	APC-A Median
All Events	####	####	####	####	####	105	78	3,127	2,457	218	117
P2	####	####	####	####	####	105	78	3,128	2,461	218	117
P8	P2	6,131	61.4	61.3	61.3	66	58	2,058	1,918	131	79
Q1	P8	5	0.1	0.1	0.0	33	9	169	144	2,228	621
Q2	P8	225	3.7	2.3	2.2	107	86	3,453	2,725	1,086	898
Q3	P8	912	14.9	9.1	9.1	17	14	45	36	27	17
Q4	P8	4,989	81.4	50.0	49.9	73	64	2,365	2,129	105	89
P7	P8	5,328	86.9	53.3	53.2	73	64	2,357	2,123	147	92
P1	P8	4,254	69.4	42.6	42.5	78	69	2,483	2,273	189	118
P4	P8	4,683	76.4	46.9	46.8	82	70	2,539	2,289	156	100

FACSDiva Version 6.1.3

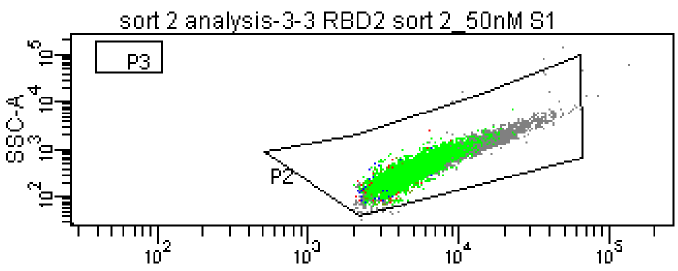

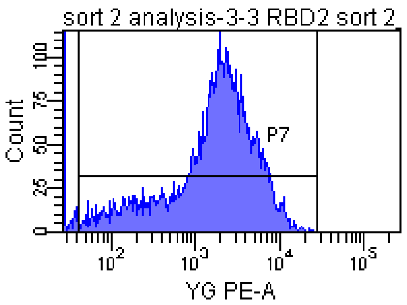

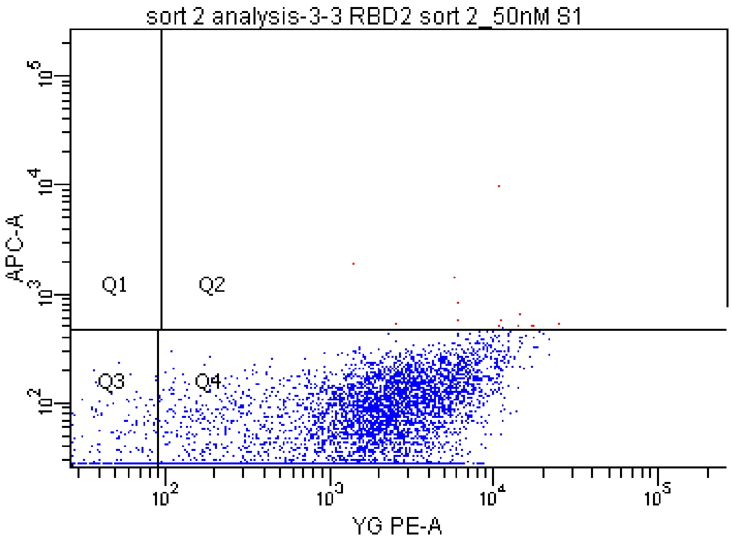

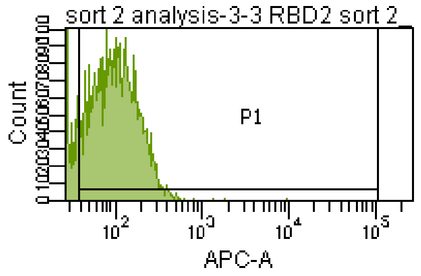

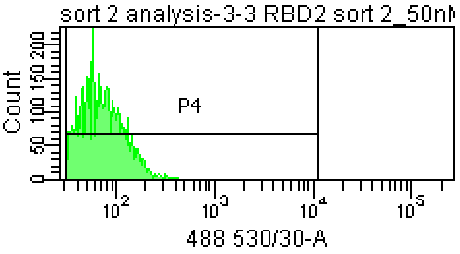

Experiment Name: Bio RBD2 selectons
Specimen Name: sort 2 analysis
Tube Name: 3-3 RBD2 sort 2_50nM S1
Record Date: May 29, 2020 2:58:30 PM
$OP: Nileena
GUID: f89412f3-8665-41b3-9cc4-0d857c8bb93a

Population	Parent...	#Events	%Parent	%Gran...	%Total	488 5... Mean	488 5... Median	PE-A Mean	PE-A Median	APC-A Mean	APC-A Median
All Events	####	####	####	####	####	100	71	2,902	2,218	236	80
P2	####	####	####	####	####	99	72	2,881	2,221	175	80
P8	P2	6,451	64.6	64.4	64.4	61	52	1,855	1,734	75	55
Q1	P8	3	0.0	0.0	0.0	5	-1	81	50	7,388	9,853
Q2	P8	14	0.2	0.1	0.1	245	259	6,462	6,406	1,328	528
Q3	P8	1,051	16.3	10.5	10.5	19	16	50	38	21	13
Q4	P8	5,383	83.4	53.9	53.7	69	60	2,197	2,006	78	64
P7	P8	5,571	86.4	55.8	55.6	68	59	2,142	1,965	79	63
P1	P8	3,859	59.8	38.6	38.5	76	66	2,355	2,175	125	97
P4	P8	4,611	71.5	46.1	46.0	80	69	2,420	2,209	89	72

FACSDiva Version 6.1.3

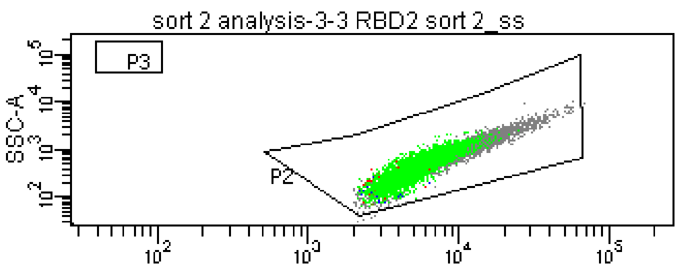

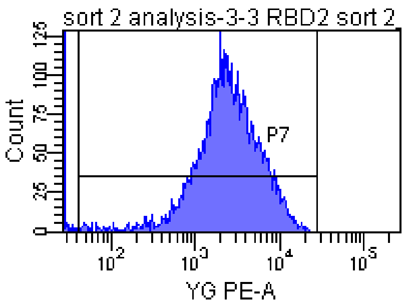

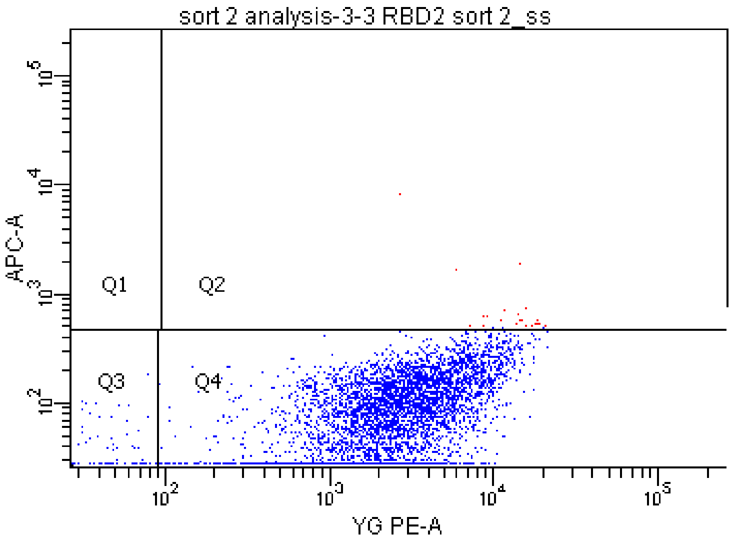

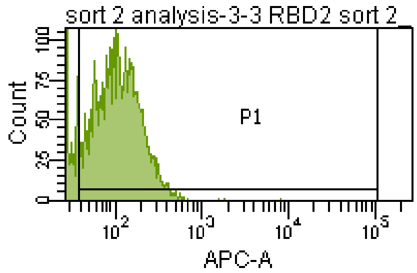

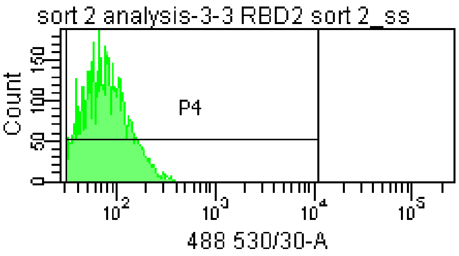

Experiment Name: Bio RBD2 selectons
Specimen Name: sort 2 analysis
Tube Name: 3-3 RBD2 sort 2_ss
Record Date: May 29, 2020 2:59:41 PM
$OP: Nileena
GUID: 4739c7b2-e42a-429b-8e3d-1f26bcc1f12d

Population	Parent...	#Events	%Parent	%Gran...	%Total	488 5... Mean	488 5... Median	PE-A Mean	PE-A Median	APC-A Mean	APC-A Median
All Events	####	####	####	####	####	117	88	3,585	2,763	145	95
P2	####	####	####	####	####	117	88	3,577	2,766	144	95
P8	P2	6,091	61.0	60.9	60.9	74	64	2,362	2,189	80	63
Q1	P8	0	0.0	0.0	0.0	####	####	####	####	####	####
Q2	P8	21	0.3	0.2	0.2	236	250	7,579	7,568	1,009	553
Q3	P8	789	13.0	7.9	7.9	19	16	44	35	18	11
Q4	P8	5,281	86.7	52.9	52.8	81	71	2,687	2,366	86	73
P7	P8	5,352	87.9	53.6	53.5	81	71	2,682	2,358	89	73
P1	P8	3,832	62.9	38.4	38.3	90	78	2,897	2,585	128	105
P4	P8	4,927	80.9	49.3	49.2	88	75	2,791	2,466	94	79

FACSDiva Version 6.1.3

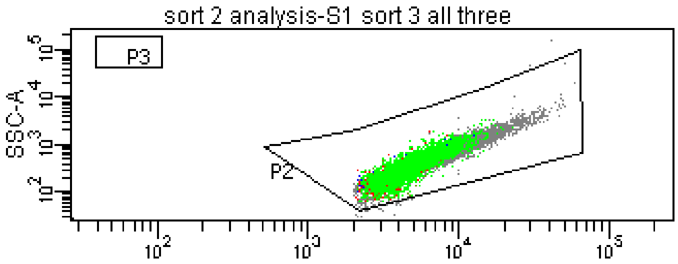

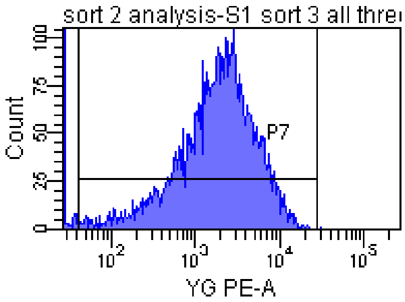

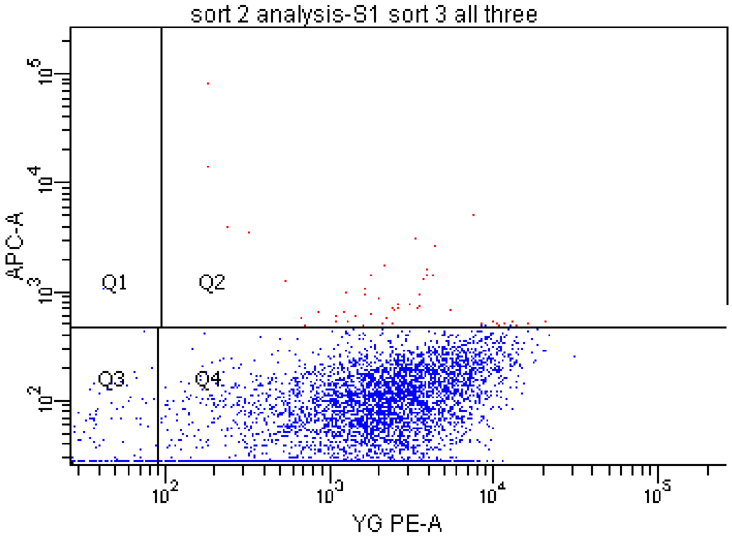

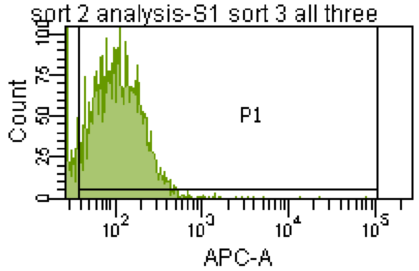

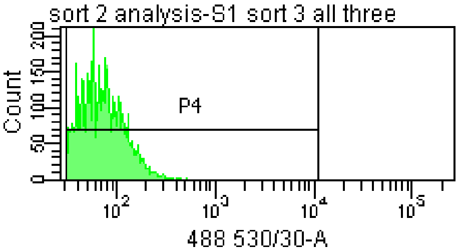

Experiment Name: Bio RBD2 selectons
Specimen Name: sort 2 analysis
Tube Name: S1 sort 3 all three
Record Date: May 29, 2020 3:45:54 PM
$OP: Nileena
GUID: 744419c7-f817-4e72-843f-d3e110639b61

Population	Parent...	#Events	%Parent	%Gran...	%Total	488 5... Mean	488 5... Median	PE-A Mean	PE-A Median	APC-A Mean	APC-A Median
All Events	####	####	####	####	####	98	72	2,831	2,179	212	83
P2	####	####	####	####	####	96	72	2,821	2,187	175	83
P8	P2	6,361	63.8	63.5	63.5	62	53	1,885	1,676	101	59
Q1	P8	7	0.1	0.1	0.1	19	26	49	46	3,781	1,808
Q2	P8	50	0.8	0.5	0.5	107	72	2,861	1,886	3,288	685
Q3	P8	875	13.8	8.8	8.7	18	15	45	32	21	12
Q4	P8	5,429	85.3	54.4	54.2	69	59	2,174	1,927	79	67
P7	P8	5,558	87.4	55.7	55.5	68	59	2,149	1,902	108	67
P1	P8	3,851	60.5	38.6	38.5	75	66	2,331	2,085	172	104
P4	P8	4,611	72.5	46.2	46.0	80	69	2,402	2,159	115	75

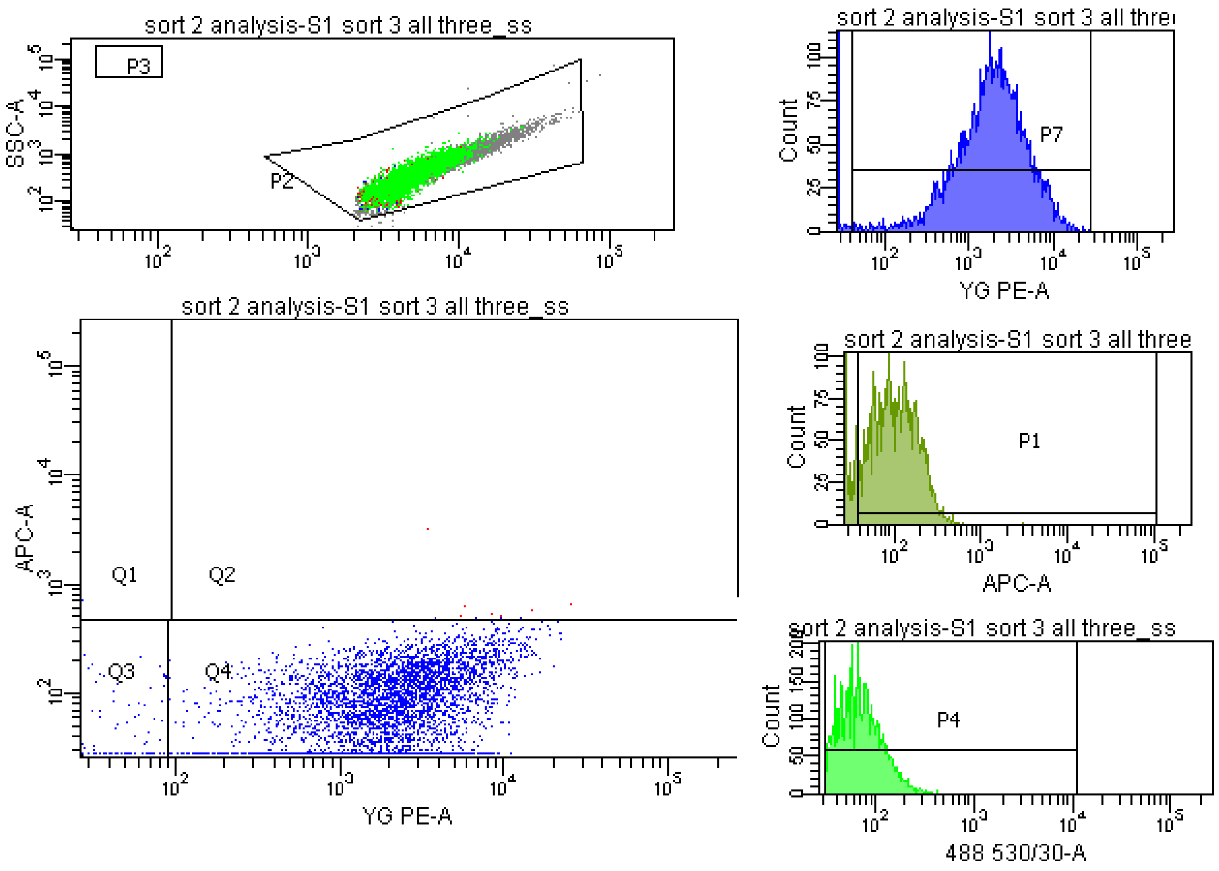

Experiment Name: Bio RBD2 selectons
Specimen Name: sort 2 analysis
Tube Name: S1 sort 3 all three_ss
Record Date: May 29, 2020 3:47:23 PM
$OP: Nileena
GUID: 255b5b73-adb2-4fd5-a86c-d468df776732

Population	Parent...	#Events	%Parent	%Gran...	%Total	488 5... Mean	488 5... Median	PE-A Mean	PE-A Median	APC-A Mean	APC-A Median
All Events	####	####	####	####	####	100	72	2,880	2,192	129	79
P2	####	####	####	####	####	99	72	2,876	2,193	119	79
P8	P2	6,292	63.0	62.8	62.8	61	53	1,874	1,691	65	54
Q1	P8	3	0.0	0.0	0.0	40	19	109	113	1,017	691
Q2	P8	7	0.1	0.1	0.1	209	216	5,666	4,365	899	545
Q3	P8	877	13.9	8.8	8.8	17	14	40	27	20	14
Q4	P8	5,405	85.9	54.1	54.0	68	60	2,167	1,936	71	60
P7	P8	5,465	86.9	54.7	54.6	68	60	2,152	1,918	71	60
P1	P8	3,618	57.5	36.2	36.1	74	64	2,286	2,073	121	102
P4	P8	4,614	73.3	46.2	46.1	78	66	2,353	2,134	78	66

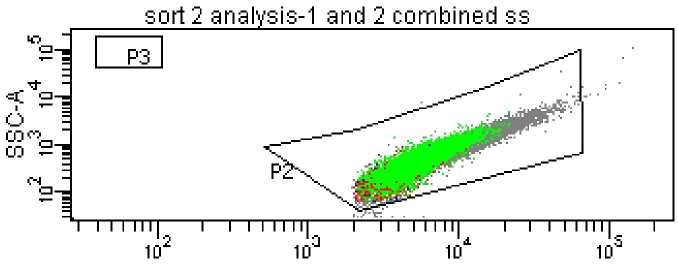

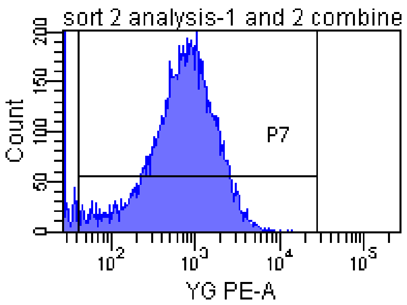

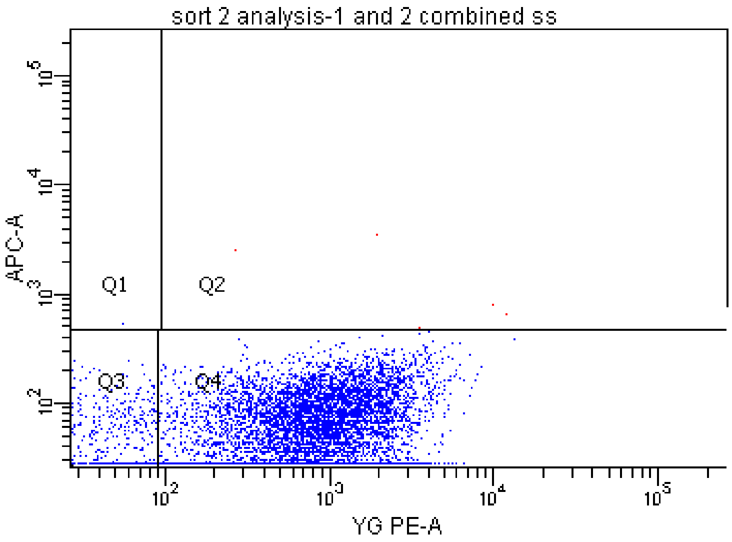

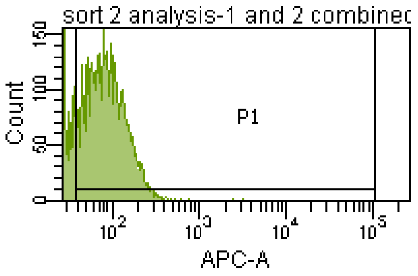

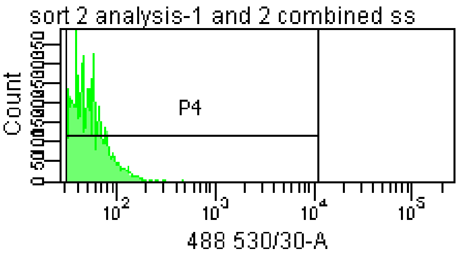

Experiment Name: Bio RBD2 selectons
Specimen Name: sort 2 analysis
Tube Name: 1 and 2 combined ss
Record Date: May 29, 2020 6:40:54 PM
$OP: Nileena
GUID: a3221f9f-ff53-453a-ad06-986b9a2ba8b1

Population	Parent...	#Events	%Parent	%Gran...	%Total	488 5... Mean	488 5... Median	PE-A Mean	PE-A Median	APC-A Mean	APC-A Median
All Events	####	####	####	####	####	59	40	1,058	789	96	42
P2	####	####	####	####	####	58	40	1,035	791	81	42
P8	P2	12,590	63.1	62.8	62.8	33	28	644	586	36	25
Q1	P8	3	0.0	0.0	0.0	20	25	70	75	7,957	2,102
Q2	P8	5	0.0	0.0	0.0	196	159	3,752	2,158	1,509	773
Q3	P8	2,843	22.6	14.2	14.2	15	14	38	24	16	10
Q4	P8	9,739	77.4	48.8	48.6	38	34	819	732	38	30
P7	P8	10,104	80.3	50.6	50.4	38	33	795	714	39	30
P1	P8	5,278	41.9	26.4	26.3	41	35	790	719	100	81
P4	P8	5,824	46.3	29.2	29.1	56	49	931	869	49	40

FACSDiva Version 6.1.3

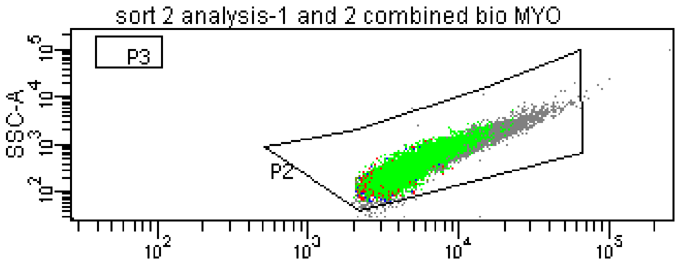

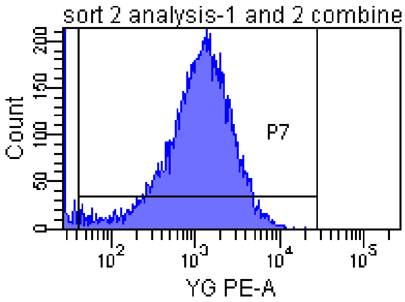

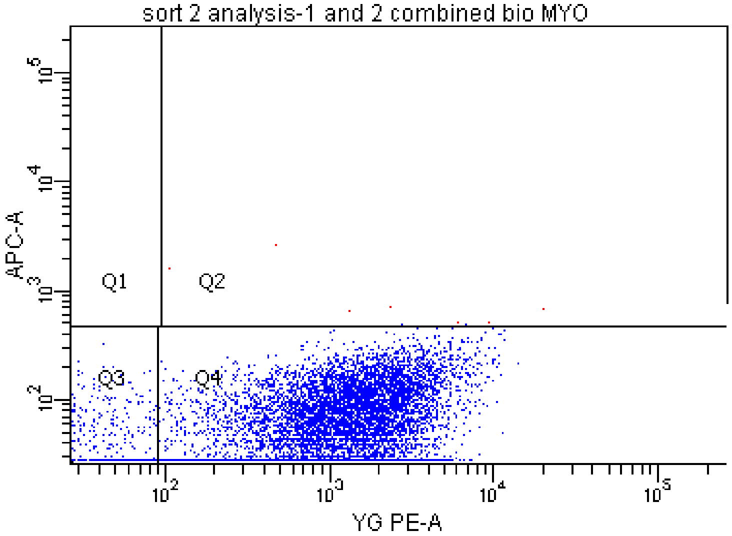

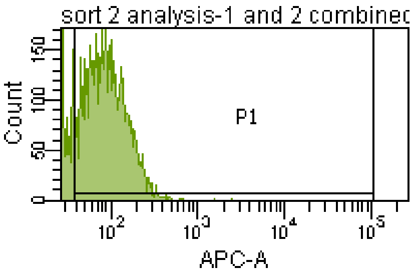

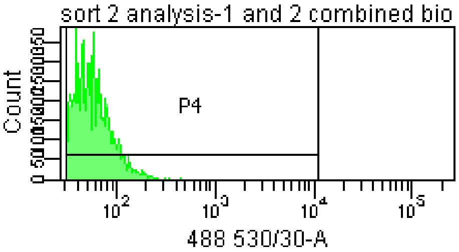

Experiment Name: Bio RBD2 selectons
Specimen Name: sort 2 analysis
Tube Name: 1 and 2 combined bio MYO
Record Date: May 29, 2020 6:41:53 PM
$OP: Nileena
GUID: 36dc573d-0d68-448c-a108-51d15ce1036a

						488 5...	488 5...	PE-A	PE-A	APC-A	APC-A
Population	Parent...	#Events	%Parent	%Gran...	%Total	Mean	Median	Mean	Median	Mean	Median
All Events	####	####	####	####	####	69	49	1,558	1,203	87	53
P2	####	####	####	####	####	68	49	1,543	1,204	79	53
P8	P2	12,955	64.9	64.6	64.6	41	36	995	911	46	35
Q1	P8	3	0.0	0.0	0.0	5	8	12	-4	4,834	3,681
Q2	P8	7	0.1	0.0	0.0	138	61	3,305	2,083	988	639
Q3	P8	2,612	20.2	13.1	13.0	14	13	31	20	17	11
Q4	P8	10,333	79.8	51.8	51.6	48	43	1,237	1,122	51	41
P7	P8	10,581	81.7	53.0	52.8	47	41	1,213	1,103	51	41
P1	P8	6,181	47.7	31.0	30.8	51	45	1,237	1,151	102	84
P4	P8	7,415	57.2	37.1	37.0	62	53	1,385	1,303	59	49

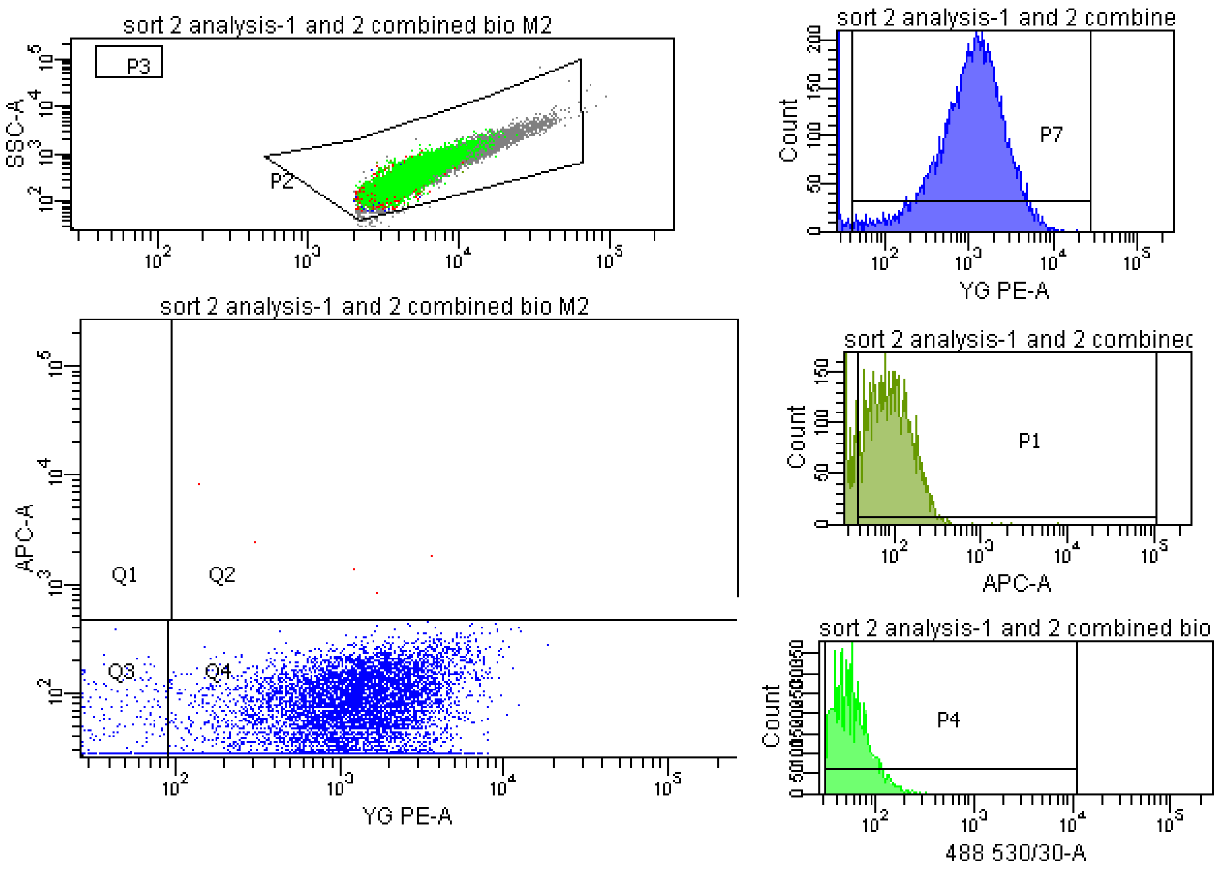

Experiment Name: Bio RBD2 selectons
Specimen Name: sort 2 analysis
Tube Name: 1 and 2 combined bio M2
Record Date: May 29, 2020 6:42:39 PM
$OP: Nileena
GUID: 72a047f2-566d-403d-ac43-bb0abc1c3d94

Population	Parent...	#Events	%Parent	%Gran...	%Total	488 5... Mean	488 5... Median	PE-A Mean	PE-A Median	APC-A Mean	APC-A Median
All Events	####	####	####	####	####	67	49	1,491	1,179	96	52
P2	####	####	####	####	####	66	49	1,482	1,182	92	52
P8	P2	13,022	65.2	65.0	65.0	41	36	999	910	44	34
Q1	P8	0	0.0	0.0	0.0	####	####	####	####	####	####
Q2	P8	5	0.0	0.0	0.0	56	47	1,041	1,388	2,784	1,719
Q3	P8	2,338	18.0	11.7	11.7	14	13	31	17	14	8
Q4	P8	10,679	82.0	53.5	53.3	47	41	1,210	1,083	49	40
P7	P8	10,878	83.5	54.5	54.3	46	41	1,191	1,068	50	39
P1	P8	6,177	47.4	30.9	30.8	50	45	1,217	1,109	104	87
P4	P8	7,453	57.2	37.3	37.2	62	53	1,372	1,281	58	48

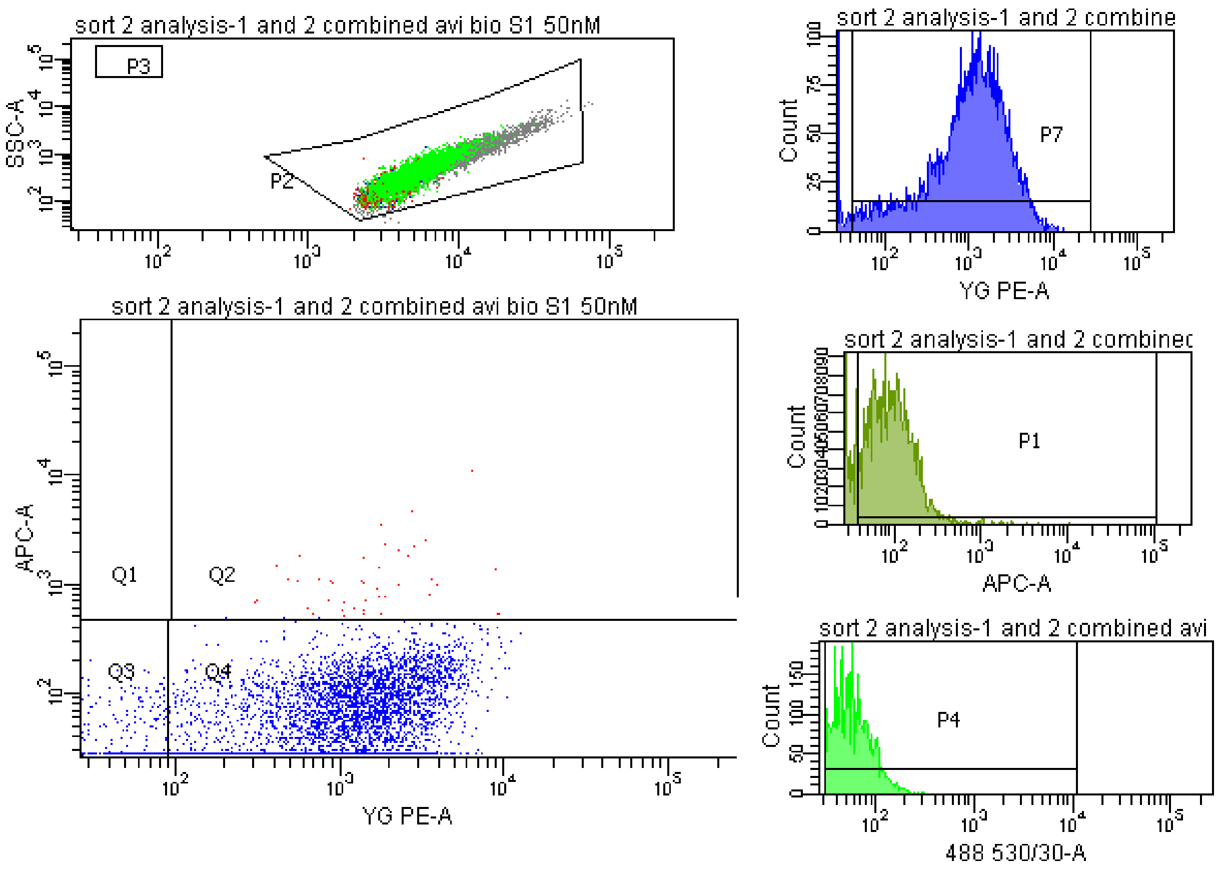

Experiment Name: Bio RBD2 selectons
Specimen Name: sort 2 analysis
Tube Name: 1 and 2 combined avi bio S1 50nM
Record Date: May 29, 2020 6:33:41 PM
$OP: Nileena
GUID: cefc29ac-740f-4c6f-abb4-ed071402f6dc

Population	Parent...	#Events	%Parent	%Gran...	%Total	488 5... Mean	488 5... Median	PE-A Mean	PE-A Median	APC-A Mean	APC-A Median
All Events	####	####	####	####	####	70	50	1,581	1,222	113	59
P2	####	####	####	####	####	69	50	1,575	1,224	102	59
P8	P2	6,399	64.1	64.0	64.0	42	36	994	905	58	37
Q1	P8	1	0.0	0.0	0.0	-7	-7	54	54	1,032	1,032
Q2	P8	42	0.7	0.4	0.4	65	48	1,518	1,143	1,381	947
Q3	P8	1,382	21.6	13.8	13.8	15	12	36	24	16	10
Q4	P8	4,974	77.7	49.8	49.7	49	44	1,256	1,141	58	47
P7	P8	5,229	81.7	52.4	52.3	48	43	1,211	1,107	67	46
P1	P8	3,160	49.4	31.7	31.6	52	46	1,276	1,182	121	89
P4	P8	3,625	56.6	36.3	36.2	64	56	1,417	1,356	78	55

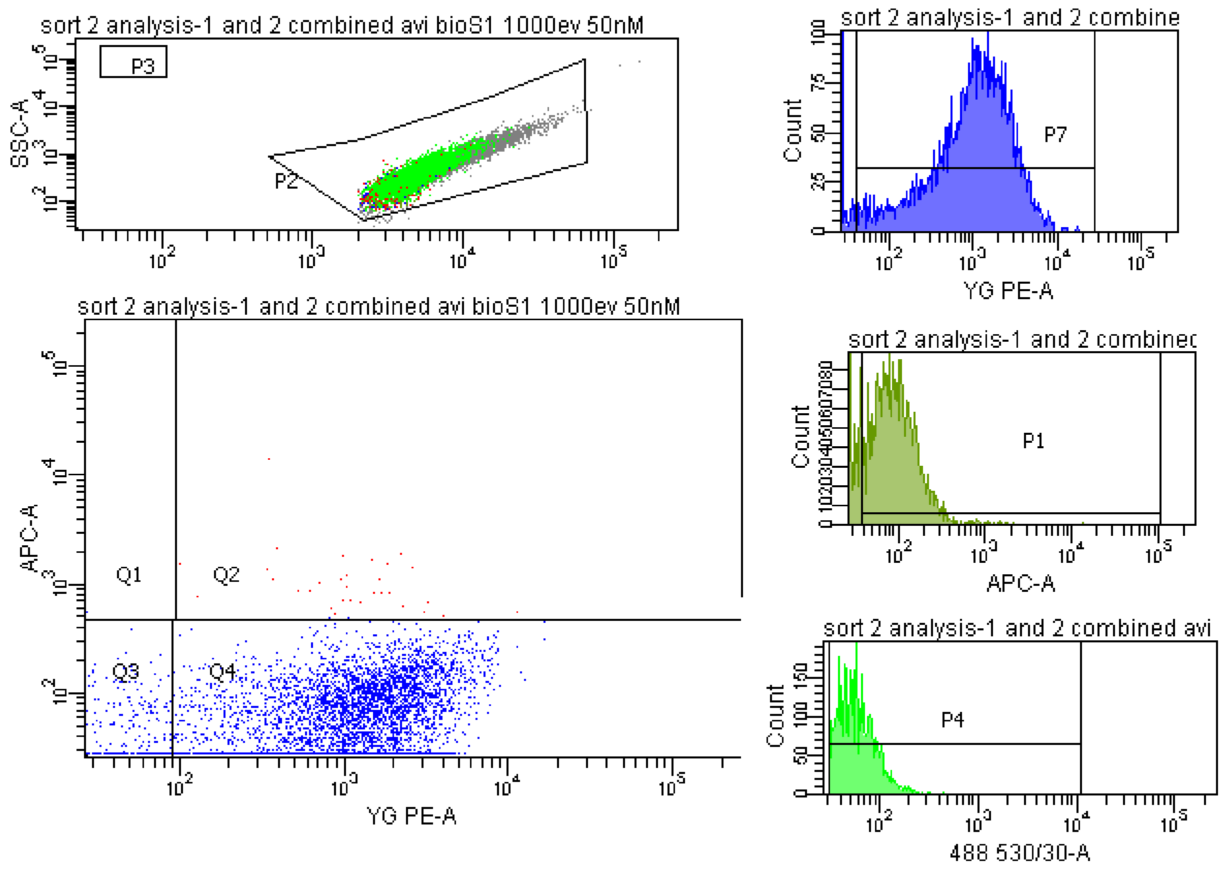

Experiment Name: Bio RBD2 selectons
Specimen Name: sort 2 analysis
Tube Name: 1 and 2 combined avi bioS1 1000ev 50nM
Record Date: May 29, 2020 6:32:34 PM
$OP: Nileena
GUID: 23fb99f3-12d2-448c-a202-56d607a5274c

Population	Parent...	#Events	%Parent	%Gran...	%Total	488 5... Mean	488 5... Median	PE-A Mean	PE-A Median	APC-A Mean	APC-A Median
All Events	####	####	####	####	####	69	48	1,594	1,177	97	55
P2	####	####	####	####	####	67	48	1,522	1,180	92	55
P8	P2	6,637	66.5	66.3	66.3	41	35	977	860	56	37
Q1	P8	4	0.1	0.0	0.0	11	8	30	29	3,793	2,150
Q2	P8	32	0.5	0.3	0.3	53	41	1,170	991	1,365	833
Q3	P8	1,454	21.9	14.6	14.5	14	13	37	25	19	12
Q4	P8	5,147	77.6	51.5	51.4	48	43	1,242	1,130	56	44
P7	P8	5,395	81.3	54.0	53.9	47	41	1,196	1,091	62	43
P1	P8	3,235	48.7	32.4	32.3	51	45	1,254	1,170	120	86
P4	P8	3,635	54.8	36.4	36.3	63	56	1,426	1,355	70	55

FACSDiva Version 6.1.3

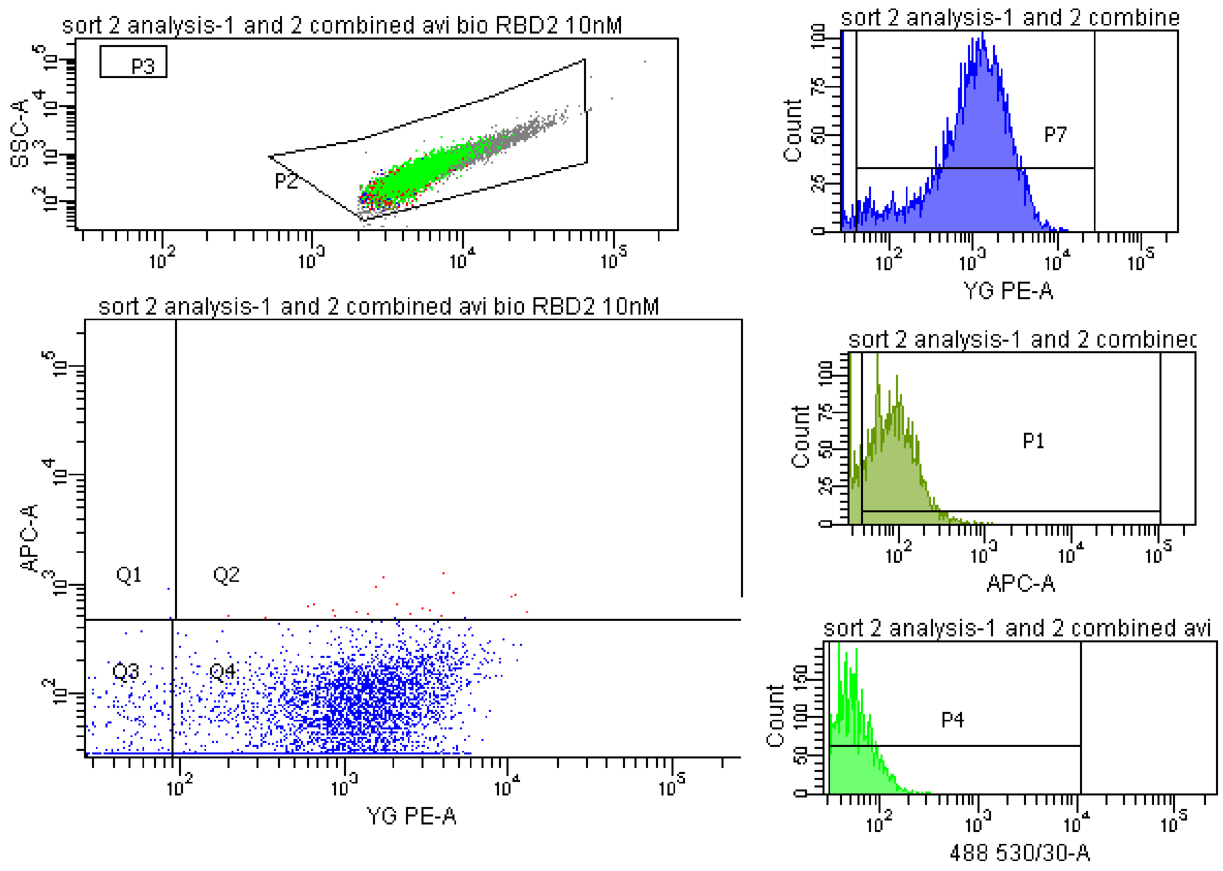

Experiment Name: Bio RBD2 selectons
Specimen Name: sort 2 analysis
Tube Name: 1 and 2 combined avi bio RBD2 10nM
Record Date: May 29, 2020 6:35:34 PM
$OP: Nileena
GUID: 40050fd4-67f7-4bbe-85ac-024f7f33d350

Population	Parent...	#Events	%Parent	%Gran...	%Total	488 5... Mean	488 5... Median	PE-A Mean	PE-A Median	APC-A Mean	APC-A Median
All Events	####	####	####	####	####	67	47	1,502	1,151	146	57
P2	####	####	####	####	####	65	47	1,460	1,154	119	57
P8	P2	6,673	66.8	66.6	66.6	40	35	947	860	51	39
Q1	P8	2	0.0	0.0	0.0	25	25	60	60	2,753	2,753
Q2	P8	20	0.3	0.2	0.2	98	66	2,110	1,640	648	564
Q3	P8	1,466	22.0	14.7	14.6	14	13	36	25	17	9
Q4	P8	5,185	77.7	51.9	51.8	47	41	1,200	1,100	58	48
P7	P8	5,446	81.6	54.6	54.4	45	40	1,155	1,058	59	47
P1	P8	3,364	50.4	33.7	33.6	49	43	1,178	1,104	108	87
P4	P8	3,629	54.4	36.4	36.2	62	53	1,366	1,307	69	56

FACSDiva Version 6.1.3

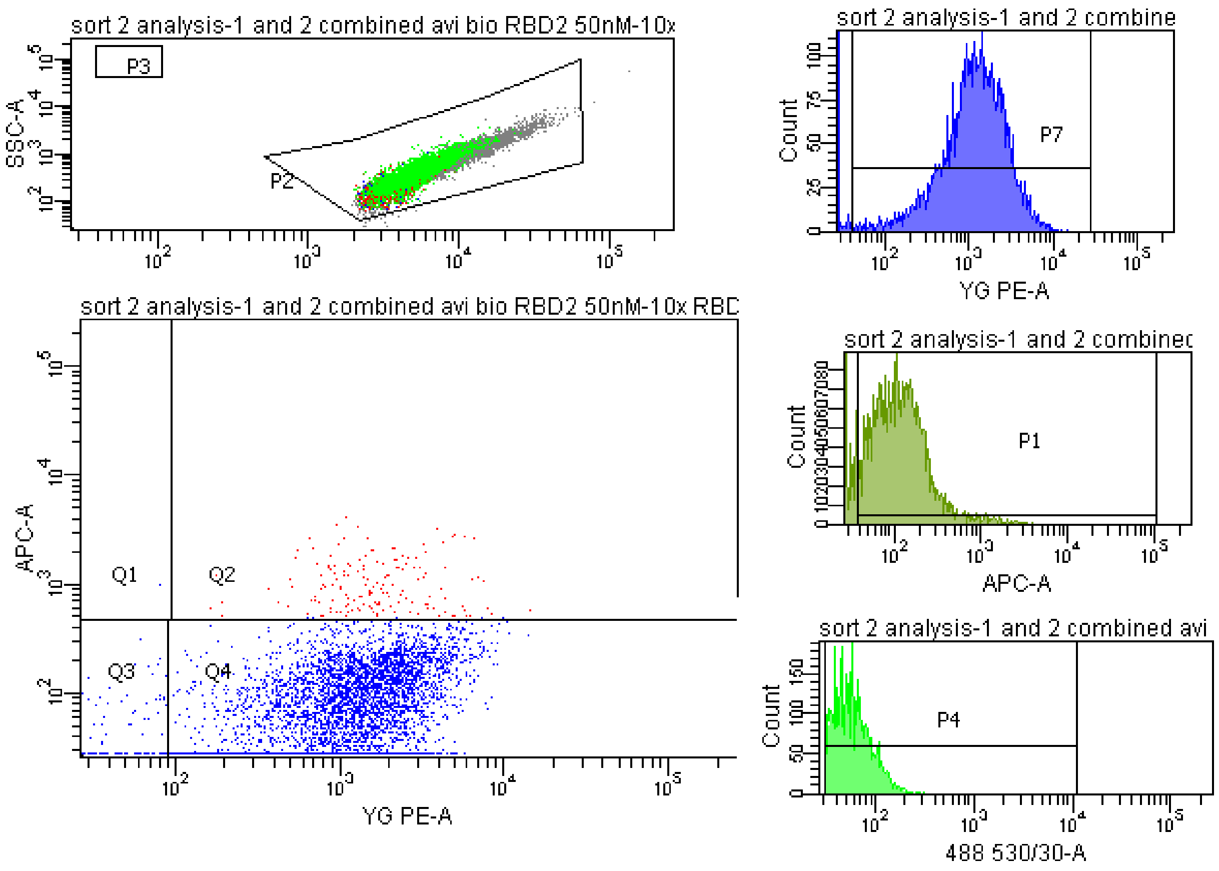

Experiment Name: Bio RBD2 selectons
Specimen Name: sort 2 analysis
Tube Name: 1 and 2 combined avi bio RBD2 50nM-10x RBD1
Record Date: May 29, 2020 6:37:02 PM
$OP: Nileena
GUID: 3113aa44-59f4-477e-bc3f-5c0805e8c251

Population	Parent...	#Events	%Parent	%Gran...	%Total	488 5... Mean	488 5... Median	PE-A Mean	PE-A Median	APC-A Mean	APC-A Median
All Events	####	####	####	####	####	73	52	1,674	1,285	174	87
P2	####	####	####	####	####	72	53	1,655	1,288	172	87
P8	P2	6,195	62.0	61.9	61.9	43	38	1,061	980	96	57
Q1	P8	2	0.0	0.0	0.0	19	19	65	65	1,739	1,739
Q2	P8	160	2.6	1.6	1.6	64	50	1,606	1,310	1,100	881
Q3	P8	975	15.7	9.8	9.7	15	13	31	16	15	6
Q4	P8	5,058	81.6	50.7	50.5	48	43	1,242	1,107	79	64
P7	P8	5,287	85.3	52.9	52.8	48	43	1,239	1,105	110	66
P1	P8	3,625	58.5	36.3	36.2	52	46	1,305	1,188	170	109
P4	P8	3,689	59.5	36.9	36.8	64	56	1,422	1,327	124	78

FACSDiva Version 6.1.3

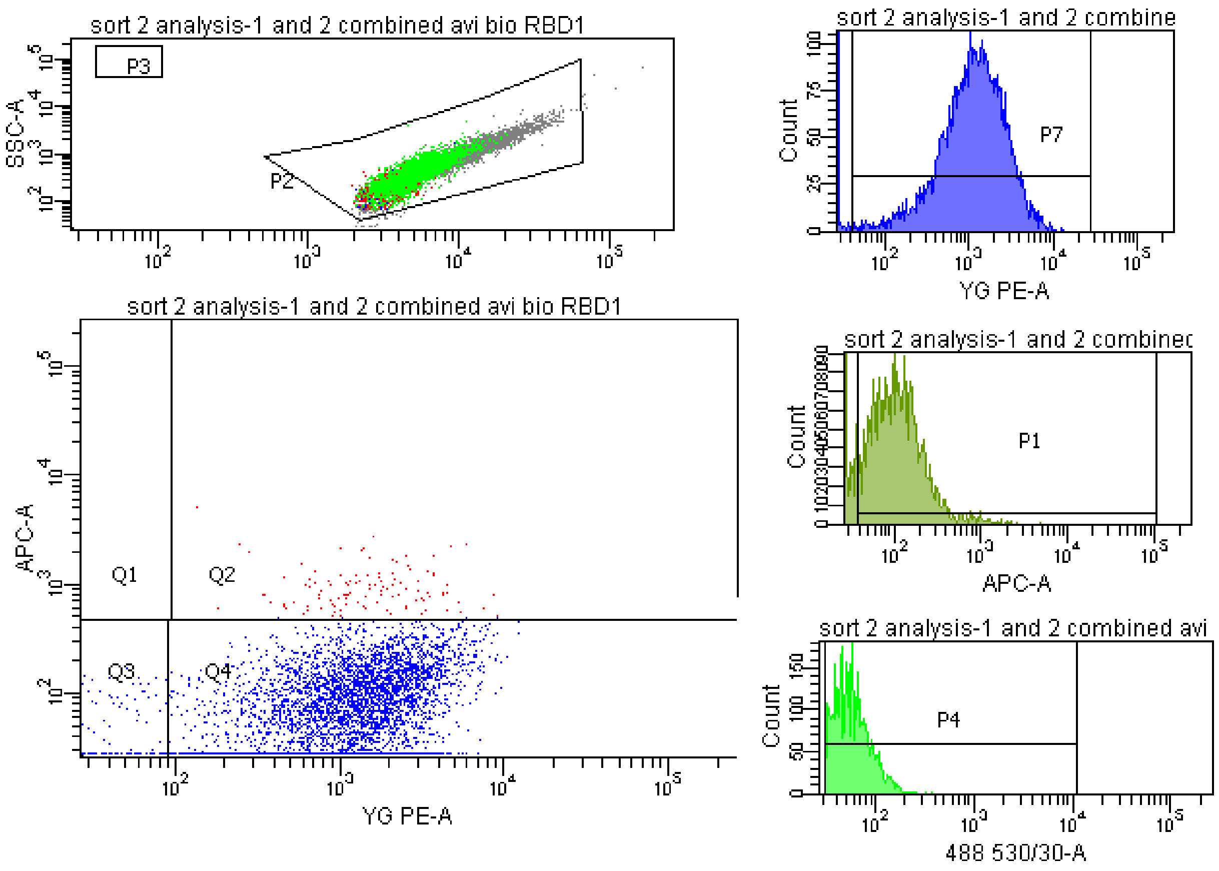

Experiment Name:	Bio RBD2 selectons
Specimen Name:	sort 2 analysis
Tube Name:	1 and 2 combined avi bio RBD1
Record Date:	May 29, 2020 6:38:09 PM
$OP:	Nileena
GUID:	5b590272-a8b2-4e3c-9bda-0e7150ad8fbe

Population	Parent...	#Events	%Parent	%Gran...	%Total	488 5... Mean	488 5... Median	PE-A Mean	PE-A Median	APC-A Mean	APC-A Median
All Events	####	####	####	####	####	74	51	1,745	1,278	159	80
P2	####	####	####	####	####	73	52	1,704	1,280	144	80
P8	P2	6,249	62.6	62.4	62.4	42	36	1,045	976	80	51
Q1	P8	3	0.0	0.0	0.0	7	8	-24	-14	2,182	2,500
Q2	P8	110	1.8	1.1	1.1	55	47	1,465	1,311	984	805
Q3	P8	995	15.9	10.0	9.9	15	12	28	14	18	12
Q4	P8	5,141	82.3	51.5	51.3	46	41	1,233	1,116	72	58
P7	P8	5,321	85.1	53.3	53.1	46	41	1,223	1,109	90	59
P1	P8	3,516	56.3	35.2	35.1	50	45	1,281	1,196	149	102
P4	P8	3,580	57.3	35.9	35.7	62	54	1,418	1,333	103	72

FACSDiva Version 6.1.3

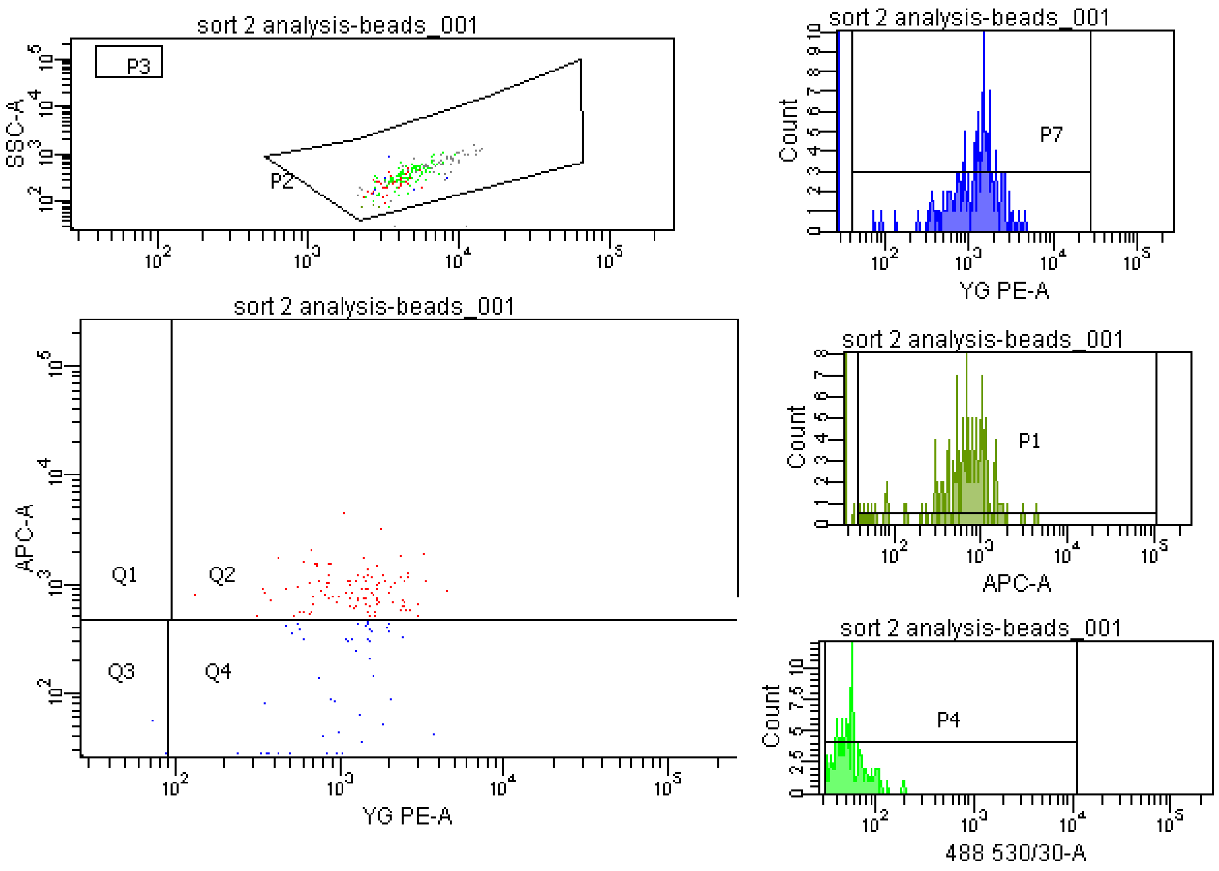

Experiment Name: Bio RBD2 selectons
Specimen Name: sort 2 analysis
Tube Name: beads_001
Record Date: May 29, 2020 8:15:03 PM
$OP: Nileena
GUID: 3fd6d6a7-6daa-446a-8a7f-38e51d6408d3

Population	Parent...	#Events	%Parent	%Gran...	%Total	488 5... Mean	488 5... Median	PE-A Mean	PE-A Median	APC-A Mean	APC-A Median
All Events	####	####	####	####	####	53	49	1,244	1,211	725	665
P2	####	####	####	####	####	53	49	1,256	1,213	732	678
P8	P2	158	73.8	73.1	73.1	45	44	1,116	1,126	663	620
Q1	P8	0	0.0	0.0	0.0	####	####	####	####	####	####
Q2	P8	101	63.9	47.2	46.8	50	47	1,239	1,230	948	831
Q3	P8	13	8.2	6.1	6.0	13	4	50	41	18	14
Q4	P8	44	27.8	20.6	20.4	45	41	1,148	1,137	199	216
P7	P8	147	93.0	68.7	68.1	47	46	1,197	1,184	711	657
P1	P8	139	88.0	65.0	64.4	49	47	1,214	1,215	753	687
P4	P8	105	66.5	49.1	48.6	60	53	1,309	1,313	721	687

FACSDiva Version 6.1.3

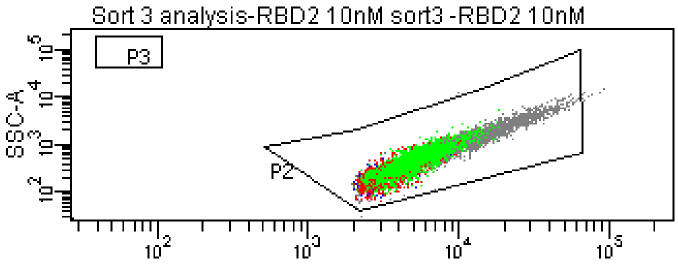

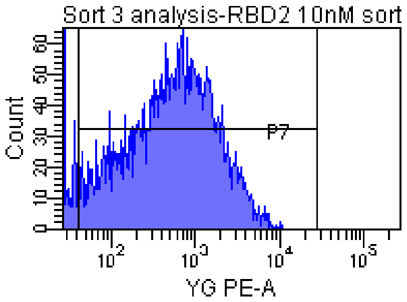

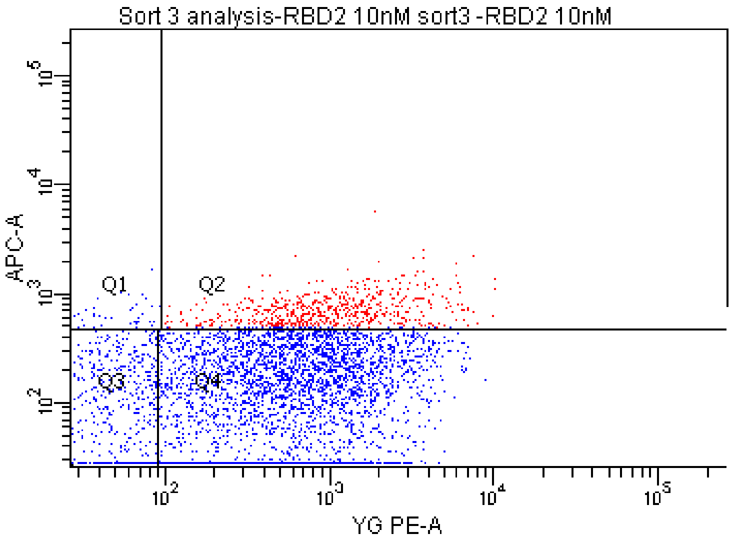

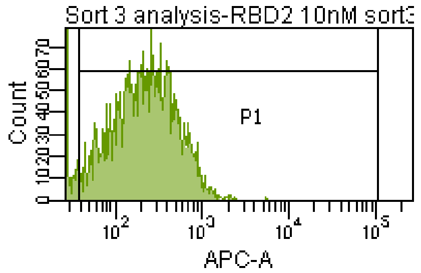

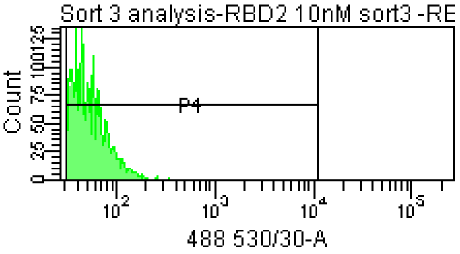

Experiment Name: Bio RBD2 selectons
Specimen Name: Sort 3 analysis
Tube Name: RBD2 10nM sort3 -RBD2 10nM
Record Date: Jun 2, 2020 2:24:20 PM
$OP: Nileena
GUID: ef062edf-b59f-4a99-8433-1c3768629cb1

Population	Parent...	#Events	%Parent	%Gran...	%Total	488 5... Mean	488 5... Median	PE-A Mean	PE-A Median	APC-A Mean	APC-A Median
All Events	####	####	####	####	####	57	35	1,205	663	322	166
P2	####	####	####	####	####	56	35	1,189	661	314	166
P8	P2	6,035	60.4	60.3	60.3	28	23	622	382	188	119
Q1	P8	60	1.0	0.6	0.6	12	8	80	55	1,034	591
Q2	P8	582	9.6	5.8	5.8	50	43	1,232	1,022	729	654
Q3	P8	2,102	34.8	21.0	21.0	12	11	59	28	60	40
Q4	P8	3,291	54.5	32.9	32.9	35	32	884	693	159	141
P7	P8	4,240	70.3	42.4	42.4	35	31	869	671	236	170
P1	P8	4,287	71.0	42.9	42.8	32	26	748	524	274	193
P4	P8	2,317	38.4	23.2	23.2	57	49	1,113	968	258	183

FACSDiva Version 6.1.3

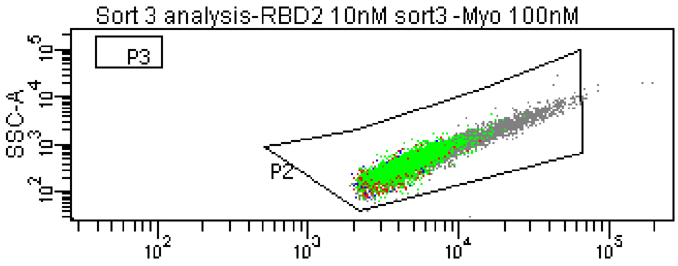

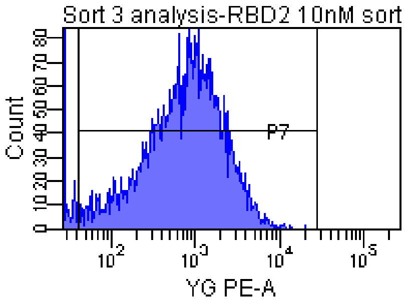

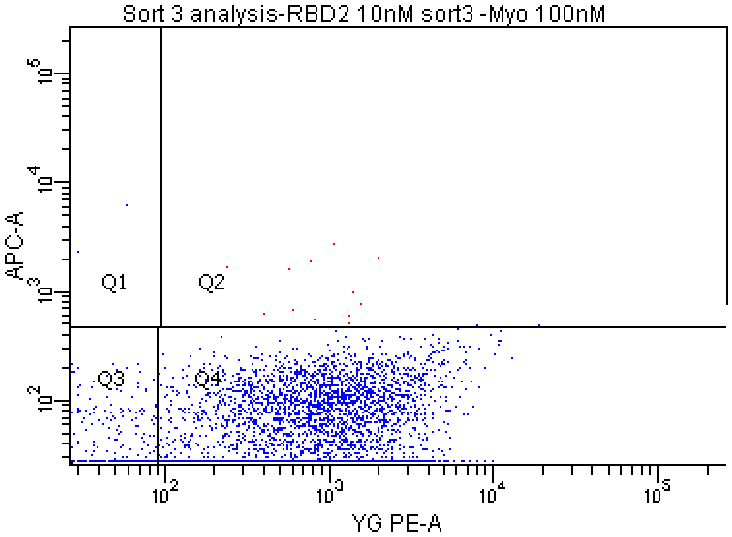

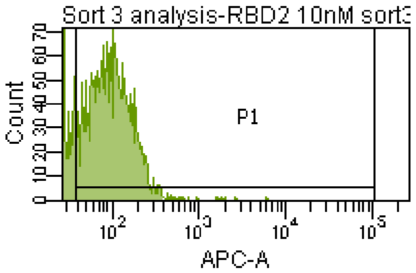

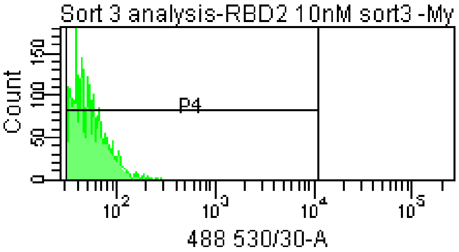

Experiment Name: Bio RBD2 selectons
Specimen Name: Sort 3 analysis
Tube Name: RBD2 10nM sort3 -Myo 100nM
Record Date: Jun 2, 2020 2:26:32 PM
$OP: Nileena
GUID: bc6d1ff4-df13-48ad-b41e-c30aced7fb78

Population	Parent...	#Events	%Parent	%Gran...	%Total	488 5... Mean	488 5... Median	PE-A Mean	PE-A Median	APC-A Mean	APC-A Median
All Events	####	####	####	####	####	65	38	1,483	879	182	50
P2	####	####	####	####	####	62	38	1,455	877	153	50
P8	P2	6,065	60.7	60.6	60.6	31	27	789	618	47	29
Q1	P8	7	0.1	0.1	0.1	18	17	147	74	7,702	5,970
Q2	P8	12	0.2	0.1	0.1	37	40	1,104	1,141	1,169	835
Q3	P8	1,576	26.0	15.8	15.8	13	11	52	20	14	10
Q4	P8	4,470	73.7	44.8	44.7	38	33	1,049	867	44	36
P7	P8	4,672	77.0	46.8	46.7	37	33	1,015	835	47	34
P1	P8	2,712	44.7	27.2	27.1	38	33	975	783	131	91
P4	P8	2,655	43.8	26.6	26.5	57	49	1,260	1,138	60	46

FACSDiva Version 6.1.3

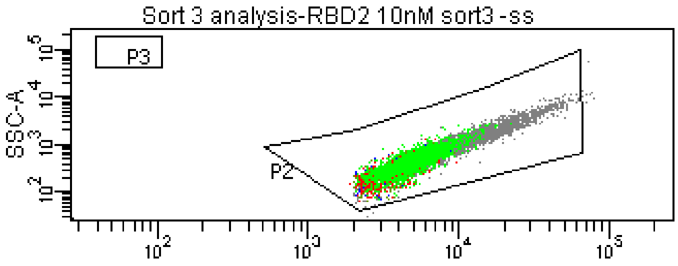

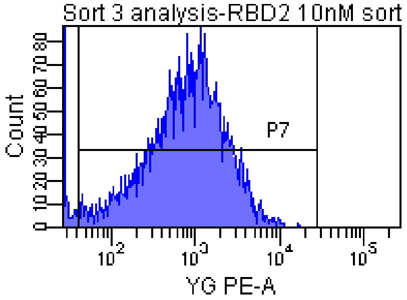

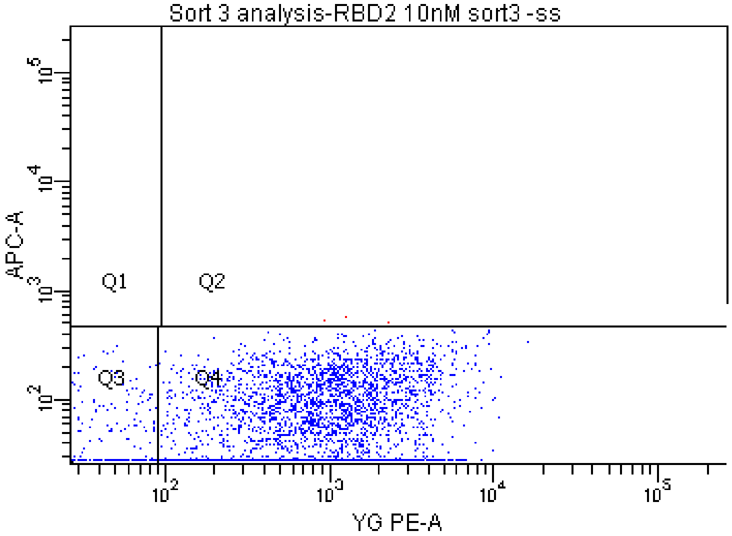

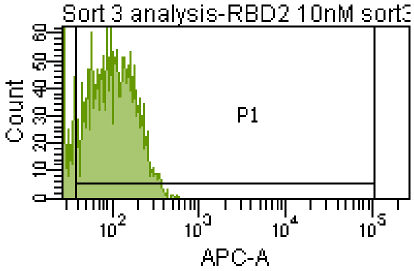

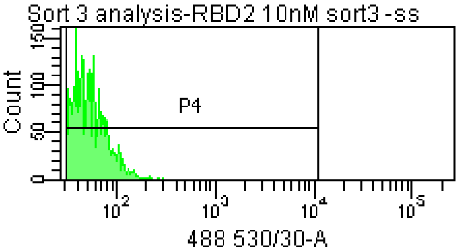

Experiment Name: Bio RBD2 selectons
Specimen Name: Sort 3 analysis
Tube Name: RBD2 10nM sort3 -ss
Record Date: Jun 2, 2020 2:27:36 PM
$OP: Nileena
GUID: 0fc7b5fe-b9e0-447e-8497-1b0ef1524521

Population	Parent...	#Events	%Parent	%Gran...	%Total	488 5... Mean	488 5... Median	PE-A Mean	PE-A Median	APC-A Mean	APC-A Median
All Events	####	####	####	####	####	63	40	1,508	944	135	50
P2	####	####	####	####	####	62	40	1,493	943	126	50
P8	P2	5,792	58.0	57.9	57.9	32	27	824	637	35	27
Q1	P8	1	0.0	0.0	0.0	36	36	76	76	1,434	1,434
Q2	P8	3	0.1	0.0	0.0	58	65	1,247	958	512	498
Q3	P8	1,407	24.3	14.1	14.1	13	11	47	14	14	10
Q4	P8	4,381	75.6	43.9	43.8	38	34	1,073	895	42	33
P7	P8	4,542	78.4	45.5	45.4	37	33	1,041	863	41	32
P1	P8	2,614	45.1	26.2	26.1	38	33	994	812	119	101
P4	P8	2,572	44.4	25.8	25.7	58	50	1,306	1,164	54	47

FACSDiva Version 6.1.3

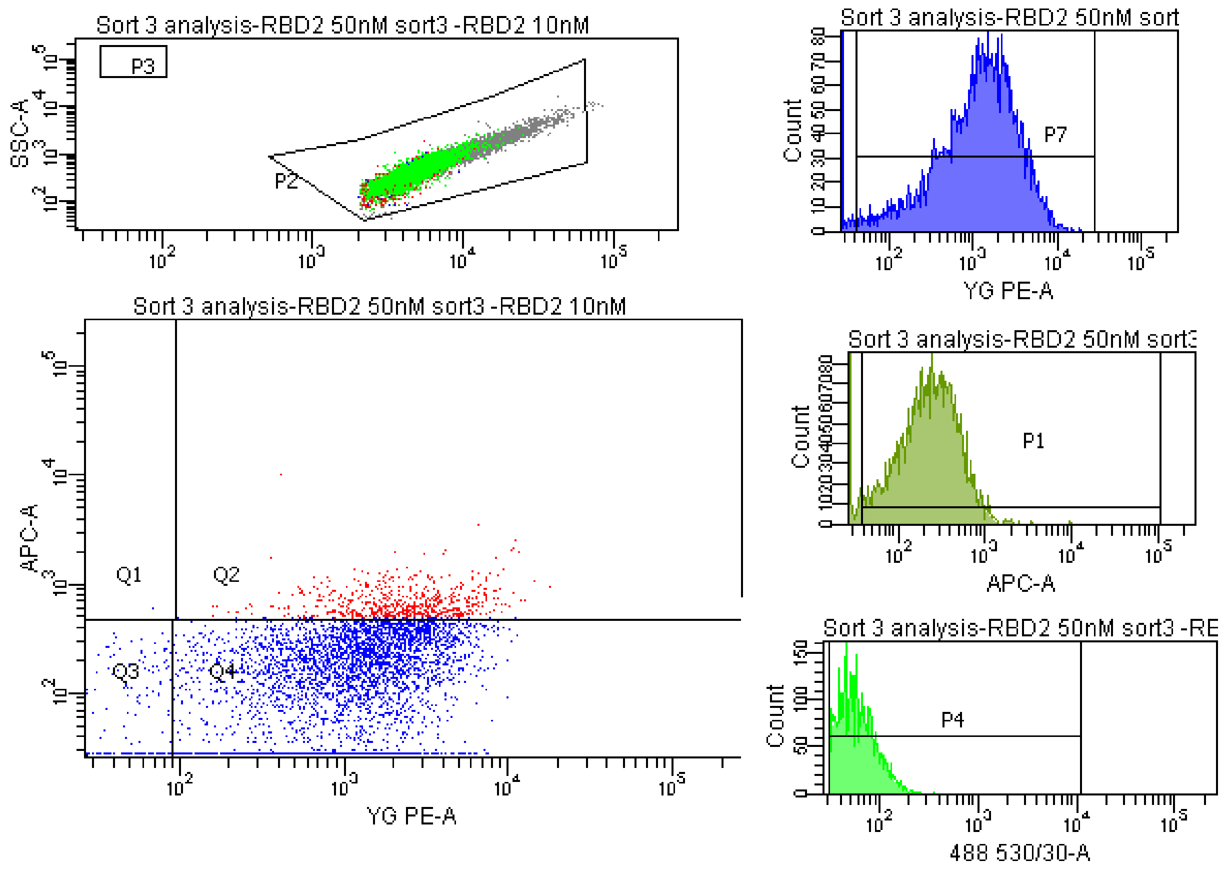

Experiment Name: Bio RBD2 selectons
Specimen Name: Sort 3 analysis
Tube Name: RBD2 50nM sort3 -RBD2 10nM
Record Date: Jun 2, 2020 2:30:09 PM
$OP: Nileena
GUID: 8f545606-3404-4927-9341-e0f94c53fa60

Population	Parent...	#Events	%Parent	%Gran...	%Total	488 5... Mean	488 5... Median	PE-A Mean	PE-A Median	APC-A Mean	APC-A Median
All Events	####	####	####	####	####	75	51	2,023	1,444	396	215
P2	####	####	####	####	####	74	51	2,001	1,441	389	215
P8	P2	5,726	57.3	57.2	57.2	41	35	1,169	993	201	148
Q1	P8	6	0.1	0.1	0.1	14	12	138	107	10,745	5,231
Q2	P8	604	10.5	6.0	6.0	73	63	2,256	2,026	706	604
Q3	P8	1,177	20.6	11.8	11.8	14	13	48	10	14	3
Q4	P8	3,939	68.8	39.4	39.4	44	40	1,339	1,185	163	159
P7	P8	4,657	81.3	46.6	46.6	47	41	1,429	1,251	231	187
P1	P8	4,168	72.8	41.7	41.7	47	41	1,400	1,254	296	219
P4	P8	3,156	55.1	31.6	31.6	64	56	1,691	1,579	255	213

FACSDiva Version 6.1.3

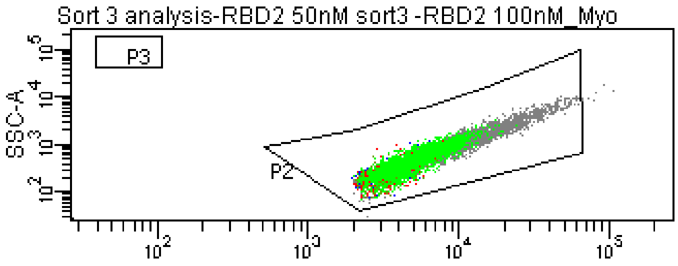

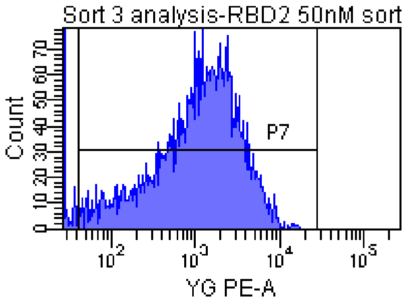

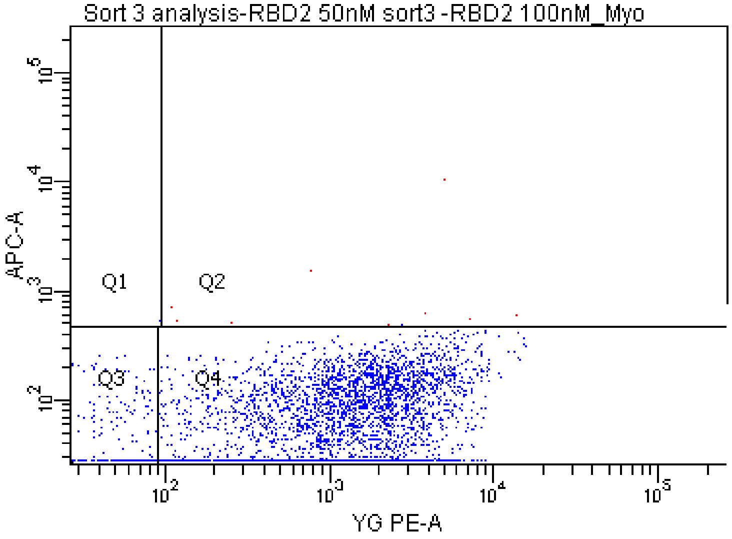

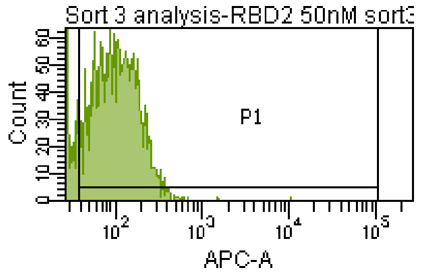

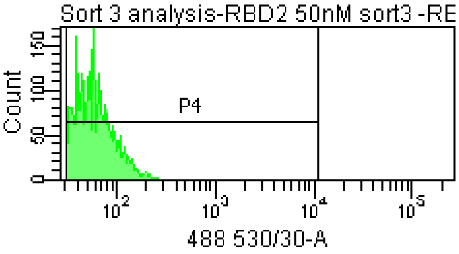

Experiment Name: Bio RBD2 selectons
Specimen Name: Sort 3 analysis
Tube Name: RBD2 50nM sort3 -RBD2 100nM_Myo
Record Date: Jun 2, 2020 2:31:18 PM
$OP: Nileena
GUID: 47e9e5e0-ac6d-401a-92e2-24286a83e5c0

Population	Parent...	#Events	%Parent	%Gran...	%Total	488 5... Mean	488 5... Median	PE-A Mean	PE-A Median	APC-A Mean	APC-A Median
All Events	####	####	####	####	####	77	52	1,967	1,388	110	65
P2	####	####	####	####	####	76	52	1,947	1,385	108	65
P8	P2	5,495	55.0	54.9	54.9	41	35	1,116	882	52	39
Q1	P8	4	0.1	0.0	0.0	28	22	26	4	2,209	2,086
Q2	P8	9	0.2	0.1	0.1	94	46	2,486	1,519	1,740	575
Q3	P8	1,300	23.7	13.0	13.0	14	12	51	17	20	13
Q4	P8	4,182	76.1	41.8	41.8	49	44	1,445	1,255	56	46
P7	P8	4,376	79.6	43.8	43.7	48	43	1,393	1,203	57	45
P1	P8	2,765	50.3	27.7	27.6	49	43	1,364	1,180	125	101
P4	P8	3,042	55.4	30.4	30.4	65	57	1,656	1,532	67	56

FACSDiva Version 6.1.3

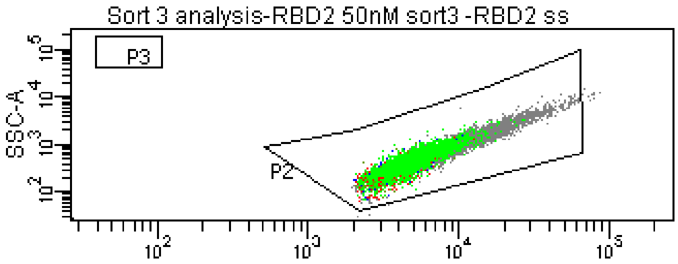

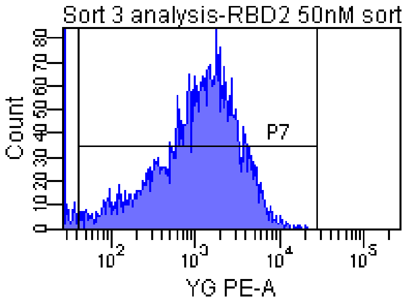

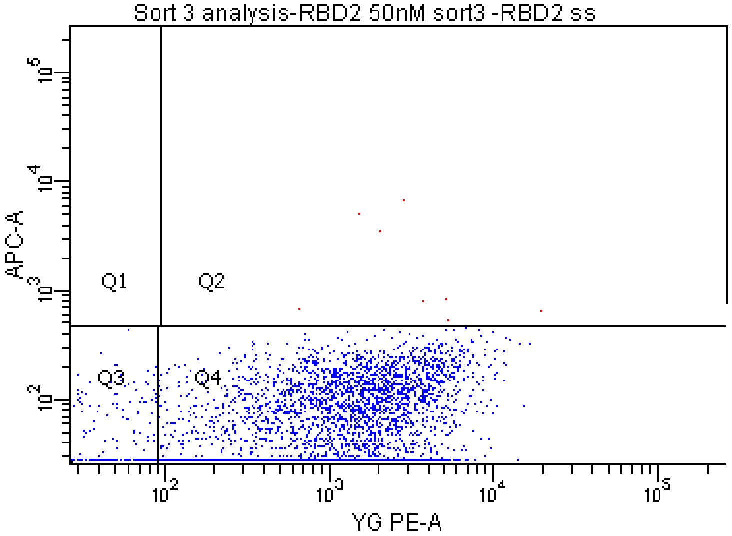

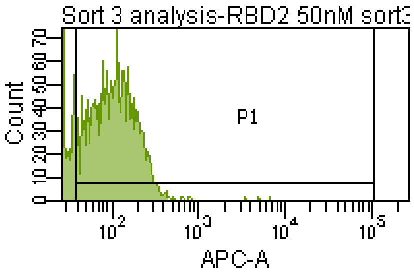

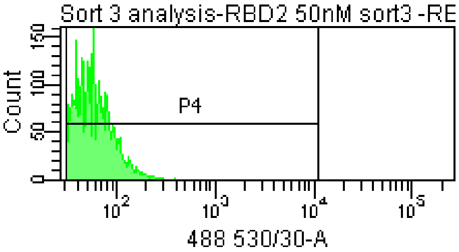

Experiment Name: Bio RBD2 selectons
Specimen Name: Sort 3 analysis
Tube Name: RBD2 50nM sort3 -RBD2 ss
Record Date: Jun 2, 2020 2:32:16 PM
$OP: Nileena
GUID: f2cad92d-5c9e-4b9c-b9cc-673d0c1410f4

Population	Parent...	#Events	%Parent	%Gran...	%Total	488 5... Mean	488 5... Median	PE-A Mean	PE-A Median	APC-A Mean	APC-A Median
All Events	####	####	####	####	####	76	52	1,955	1,360	123	62
P2	####	####	####	####	####	75	52	1,935	1,359	119	62
P8	P2	5,304	53.1	53.0	53.0	41	36	1,131	942	50	36
Q1	P8	1	0.0	0.0	0.0	1	1	24	24	16,357	16,357
Q2	P8	8	0.2	0.1	0.1	134	105	3,140	1,596	2,251	782
Q3	P8	1,148	21.6	11.5	11.5	15	13	48	10	19	13
Q4	P8	4,147	78.2	41.5	41.4	49	43	1,426	1,228	51	41
P7	P8	4,290	80.9	42.9	42.9	48	41	1,389	1,197	54	41
P1	P8	2,576	48.6	25.8	25.7	50	43	1,385	1,191	131	101
P4	P8	2,964	55.9	29.7	29.6	65	56	1,642	1,485	66	52

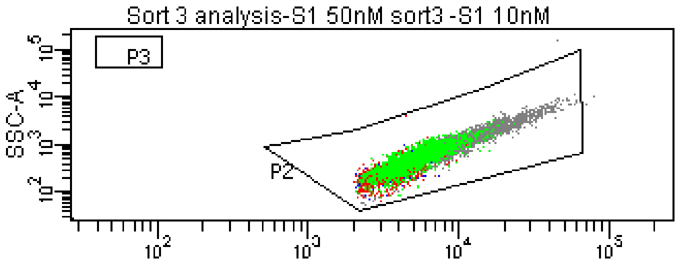

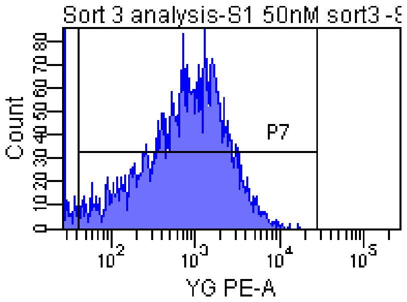

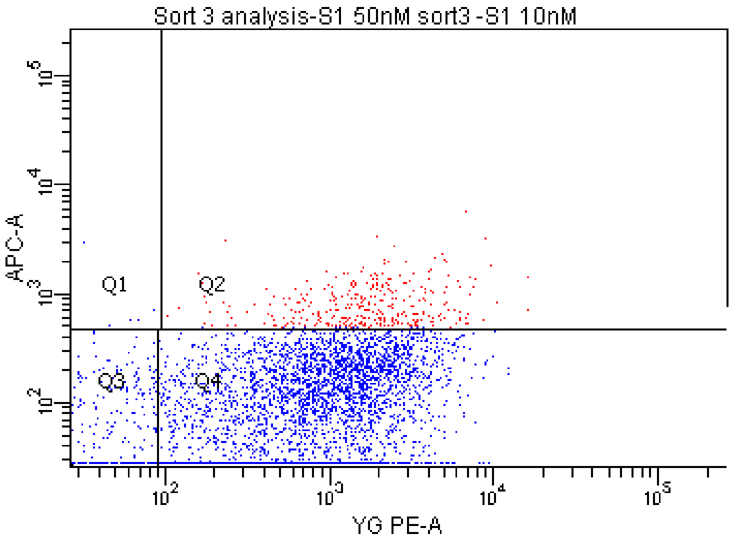

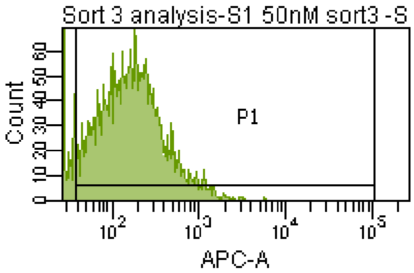

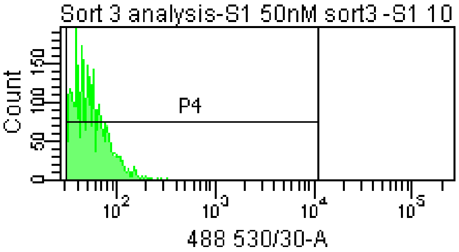

Experiment Name: Bio RBD2 selectons
Specimen Name: Sort 3 analysis
Tube Name: S1 50nM sort3 -S1 10nM
Record Date: Jun 2, 2020 2:33:36 PM
$OP: Nileena
GUID: 4bcb54d0-1d9f-45e7-85fc-758a95dda8af

Population	Parent...	#Events	%Parent	%Gran...	%Total	488 5... Mean	488 5... Median	PE-A Mean	PE-A Median	APC-A Mean	APC-A Median
All Events	####	####	####	####	####	60	41	1,274	820	235	102
P2	####	####	####	####	####	60	41	1,266	819	204	102
P8	P2	6,231	62.3	62.3	62.3	35	31	749	512	120	68
Q1	P8	8	0.1	0.1	0.1	4	11	109	68	3,748	1,272
Q2	P8	325	5.2	3.3	3.2	62	52	1,763	1,481	848	687
Q3	P8	1,966	31.6	19.7	19.7	21	19	58	24	25	19
Q4	P8	3,932	63.1	39.3	39.3	40	35	1,011	821	101	85
P7	P8	4,473	71.8	44.7	44.7	40	35	1,024	823	153	95
P1	P8	3,742	60.1	37.4	37.4	40	35	940	747	225	144
P4	P8	3,067	49.2	30.7	30.7	58	50	1,114	970	162	100

FACSDiva Version 6.1.3

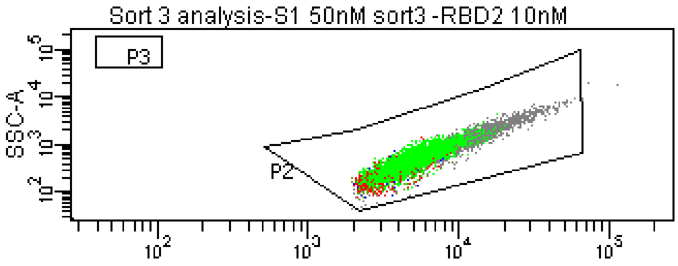

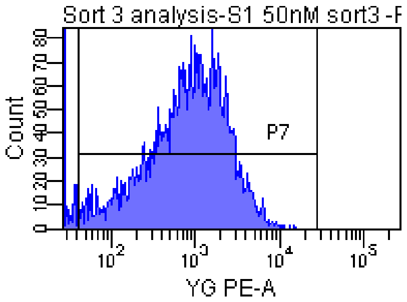

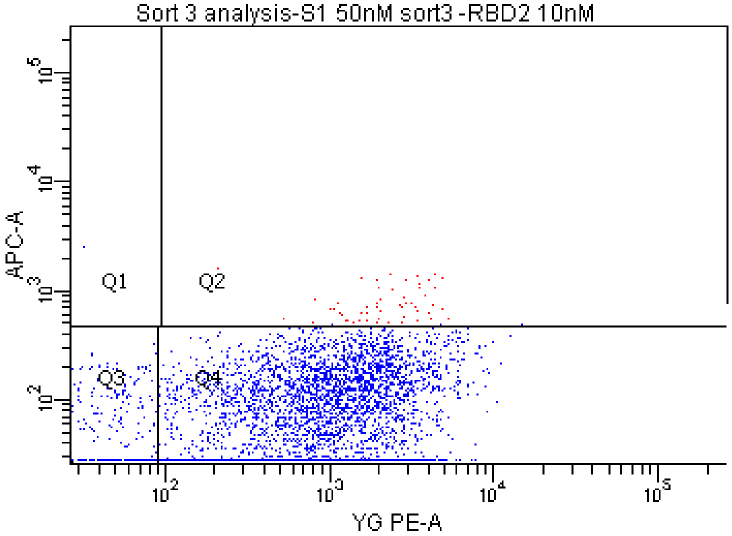

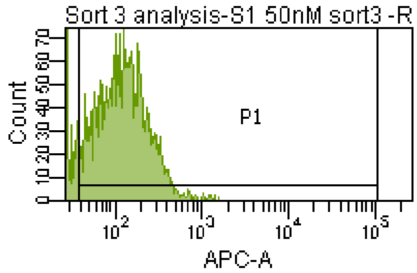

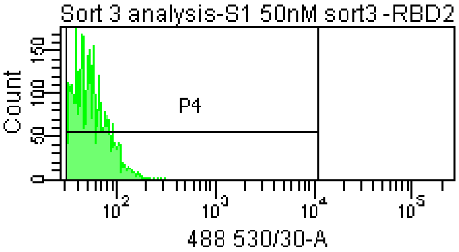

Experiment Name: Bio RBD2 selectons
Specimen Name: Sort 3 analysis
Tube Name: S1 50nM sort3 -RBD2 10nM
Record Date: Jun 2, 2020 2:34:45 PM
$OP: Nileena
GUID: 6ff47b7d-d471-4f4a-8914-66ecf00757c6

Population	Parent...	#Events	%Parent	%Gran...	%Total	488 5... Mean	488 5... Median	PE-A Mean	PE-A Median	APC-A Mean	APC-A Median
All Events	####	####	####	####	####	59	41	1,280	874	144	68
P2	####	####	####	####	####	58	41	1,274	874	136	68
P8	P2	6,363	63.7	63.6	63.6	36	32	788	580	64	46
Q1	P8	8	0.1	0.1	0.1	27	22	68	58	2,227	1,725
Q2	P8	55	0.9	0.6	0.5	68	63	1,911	1,753	762	667
Q3	P8	1,911	30.0	19.1	19.1	22	20	51	26	22	14
Q4	P8	4,389	69.0	43.9	43.9	42	37	1,095	907	69	58
P7	P8	4,632	72.8	46.3	46.3	41	36	1,067	882	75	58
P1	P8	3,359	52.8	33.6	33.6	41	36	973	788	151	117
P4	P8	3,184	50.0	31.9	31.8	59	51	1,167	1,044	85	65

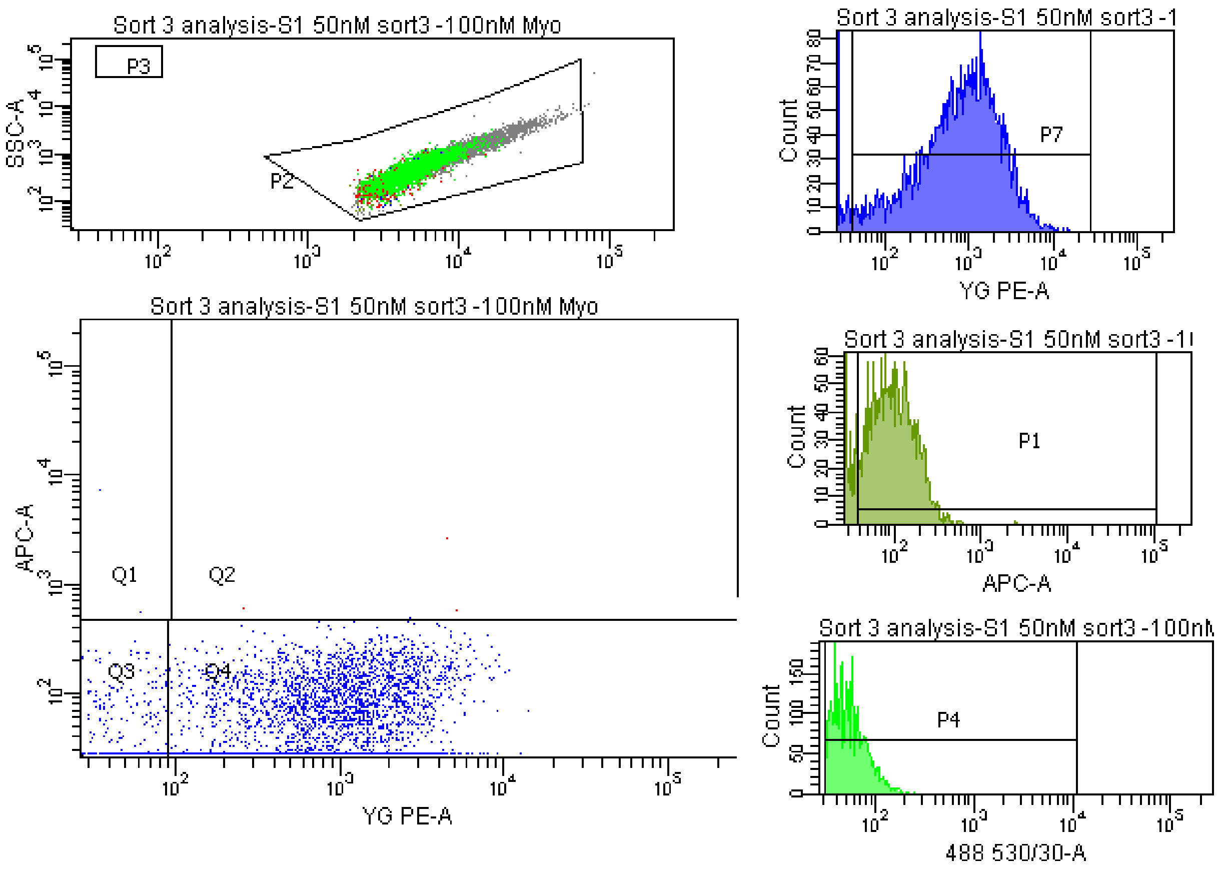

Experiment Name: Bio RBD2 selectons
Specimen Name: Sort 3 analysis
Tube Name: S1 50nM sort3 -100nM Myo
Record Date: Jun 2, 2020 2:36:11 PM
$OP: Nileena
GUID: aa31a9a4-c257-4737-abf3-937bcc11a00c

Population	Parent...	#Events	%Parent	%Gran...	%Total	488 5... Mean	488 5... Median	PE-A Mean	PE-A Median	APC-A Mean	APC-A Median
All Events	####	####	####	####	####	60	41	1,253	819	95	46
P2	####	####	####	####	####	60	41	1,248	818	94	46
P8	P2	6,316	63.2	63.1	63.1	35	31	733	520	45	26
Q1	P8	5	0.1	0.1	0.0	32	20	147	89	14,389	7,046
Q2	P8	3	0.0	0.0	0.0	67	45	2,201	2,636	1,230	576
Q3	P8	2,075	32.9	20.8	20.7	23	20	46	19	22	14
Q4	P8	4,233	67.0	42.3	42.3	41	37	1,070	901	39	31
P7	P8	4,442	70.3	44.4	44.4	41	36	1,028	850	39	30
P1	P8	2,816	44.6	28.2	28.2	40	35	870	657	137	95
P4	P8	3,103	49.1	31.0	31.0	58	51	1,091	966	47	37

FACSDiva Version 6.1.3

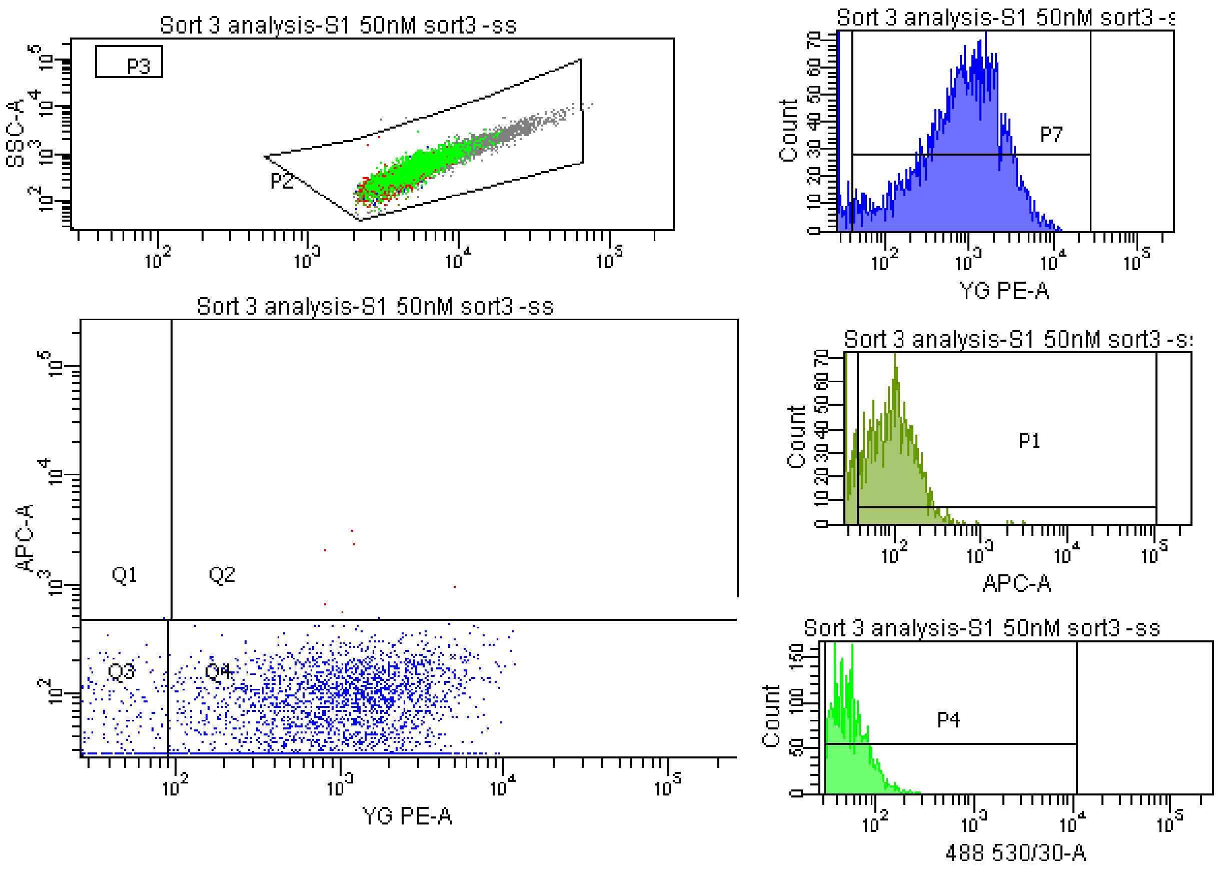

Experiment Name: Bio RBD2 selectons
Specimen Name: Sort 3 analysis
Tube Name: S1 50nM sort3 -ss
Record Date: Jun 2, 2020 2:37:17 PM
$OP: Nileena
GUID: 3f0c059e-614c-4a82-b139-c88f23ddbee5

Population	Parent...	#Events	%Parent	%Gran...	%Total	488 5... Mean	488 5... Median	PE-A Mean	PE-A Median	APC-A Mean	APC-A Median
All Events	####	####	####	####	####	63	45	1,351	888	122	47
P2	####	####	####	####	####	63	45	1,345	887	110	46
P8	P2	5,937	59.4	59.3	59.3	36	31	780	546	47	28
Q1	P8	5	0.1	0.1	0.0	18	5	148	175	13,414	11,475
Q2	P8	6	0.1	0.1	0.1	70	29	1,503	883	1,547	1,427
Q3	P8	1,850	31.2	18.5	18.5	22	20	50	26	18	16
Q4	P8	4,076	68.7	40.8	40.7	42	37	1,111	907	41	33
P7	P8	4,272	72.0	42.7	42.7	42	36	1,069	870	42	32
P1	P8	2,646	44.6	26.5	26.4	43	36	950	688	137	94
P4	P8	2,933	49.4	29.3	29.3	60	52	1,166	1,006	52	42

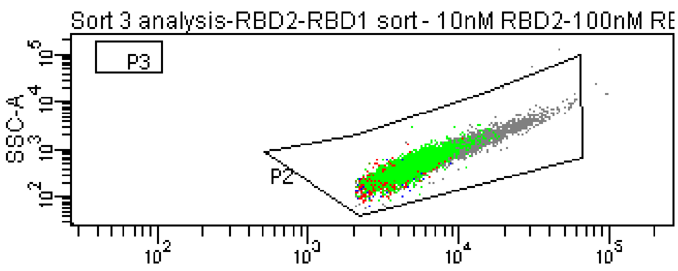

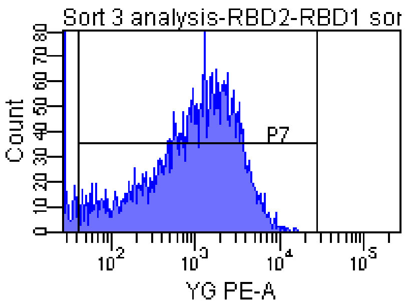

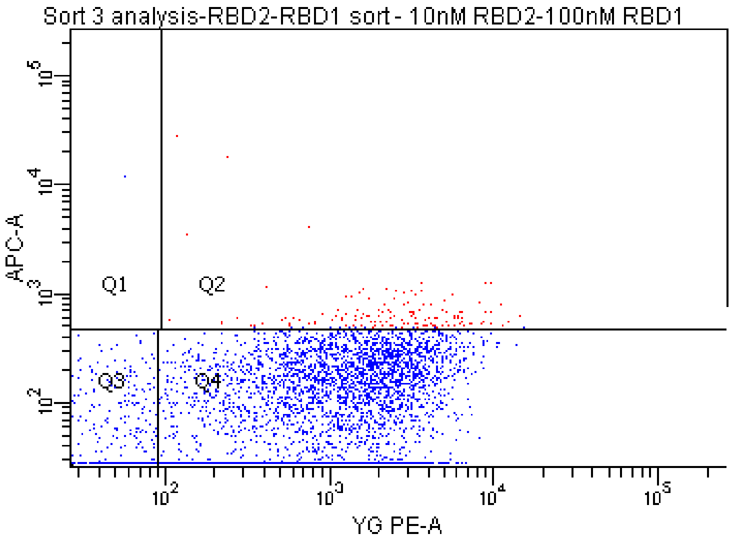

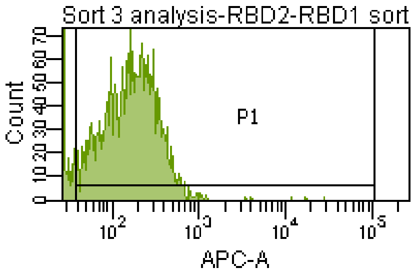

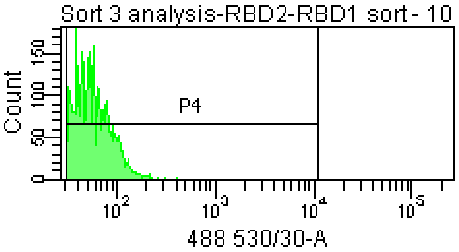

Experiment Name: Bio RBD2 selectons
Specimen Name: Sort 3 analysis
Tube Name: RBD2-RBD1 sort - 10nM RBD2-100nM RBD1
Record Date: Jun 2, 2020 2:39:20 PM
$OP: Nileena
GUID: 06c2d491-a9b4-4ae5-abb2-e741b57ea1c2

Population	Parent...	#Events	%Parent	%Gran...	%Total	488 5... Mean	488 5... Median	PE-A Mean	PE-A Median	APC-A Mean	APC-A Median
All Events	####	####	####	####	####	65	47	1,496	973	234	108
P2	####	####	####	####	####	64	47	1,483	972	203	108
P8	P2	6,332	63.3	63.3	63.3	38	33	846	458	105	68
Q1	P8	6	0.1	0.1	0.1	9	1	7	15	10,321	10,225
Q2	P8	135	2.1	1.4	1.3	87	78	2,370	2,095	995	575
Q3	P8	2,491	39.3	24.9	24.9	22	20	48	23	19	16
Q4	P8	3,700	58.4	37.0	37.0	47	43	1,329	1,135	114	105
P7	P8	4,063	64.2	40.6	40.6	47	43	1,297	1,087	141	105
P1	P8	3,725	58.8	37.2	37.2	45	39	1,107	845	213	151
P4	P8	3,310	52.3	33.1	33.1	62	53	1,317	1,183	127	100

FACSDiva Version 6.1.3

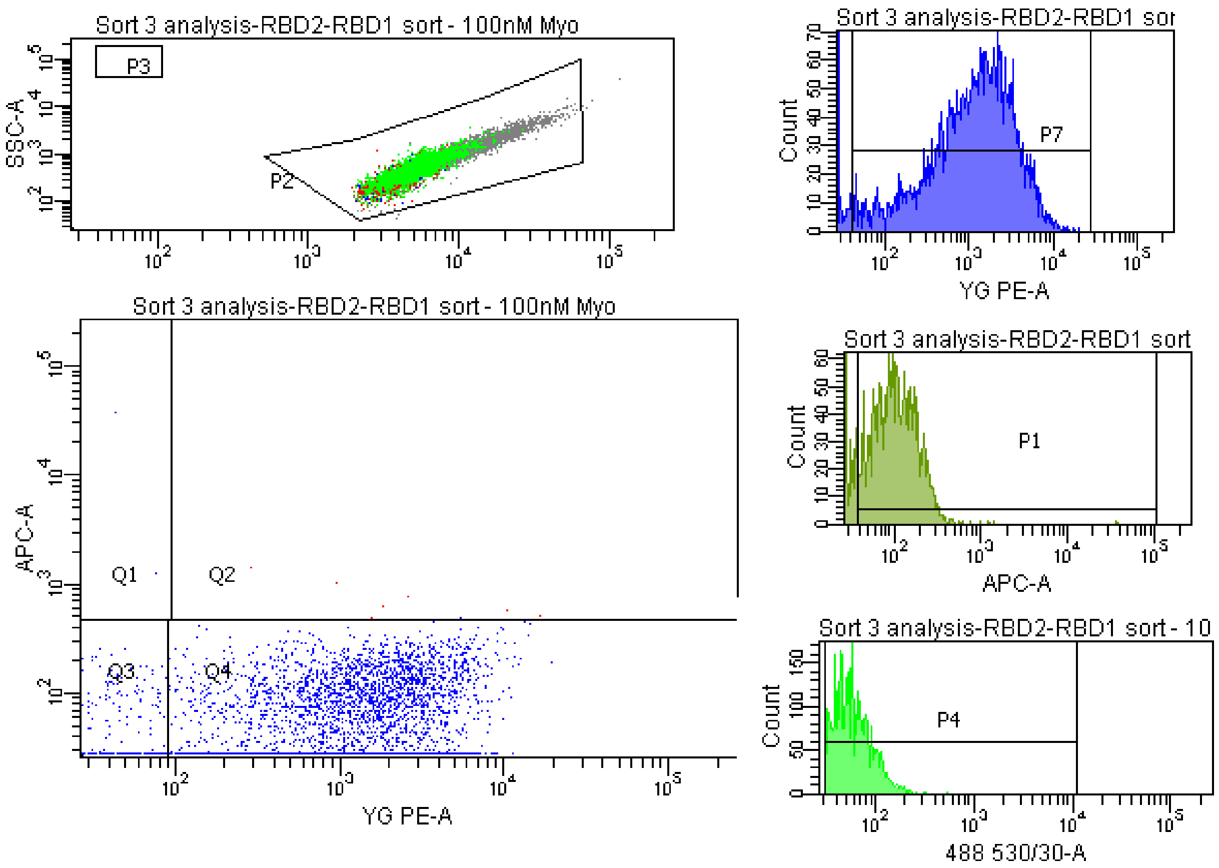

Experiment Name: Bio RBD2 selectons
Specimen Name: Sort 3 analysis
Tube Name: RBD2-RBD1 sort - 100nM Myo
Record Date: Jun 2, 2020 2:40:25 PM
$OP: Nileena
GUID: 66587714-1cef-4385-855f-d3fddb288039

Population	Parent...	#Events	%Parent	%Gran...	%Total	488 5... Mean	488 5... Median	PE-A Mean	PE-A Median	APC-A Mean	APC-A Median
All Events	####	####	####	####	####	70	49	1,669	1,105	162	51
P2	####	####	####	####	####	70	49	1,661	1,105	147	51
P8	P2	6,282	62.8	62.8	62.8	40	34	936	562	50	30
Q1	P8	9	0.1	0.1	0.1	86	14	172	59	7,775	3,408
Q2	P8	7	0.1	0.1	0.1	125	71	3,238	1,936	737	595
Q3	P8	2,362	37.6	23.6	23.6	22	20	44	15	20	13
Q4	P8	3,904	62.1	39.0	39.0	51	46	1,472	1,233	49	41
P7	P8	4,071	64.8	40.7	40.7	50	45	1,424	1,184	58	39
P1	P8	2,903	46.2	29.0	29.0	48	41	1,171	834	139	98
P4	P8	3,393	54.0	33.9	33.9	64	54	1,430	1,291	70	43

FACSDiva Version 6.1.3

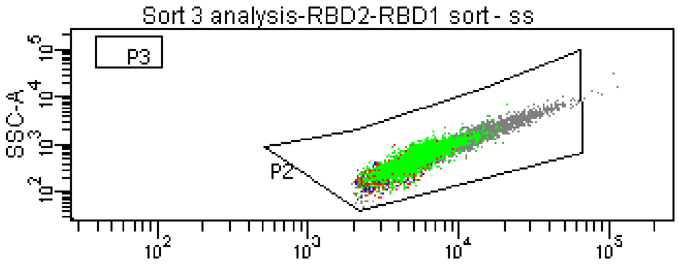

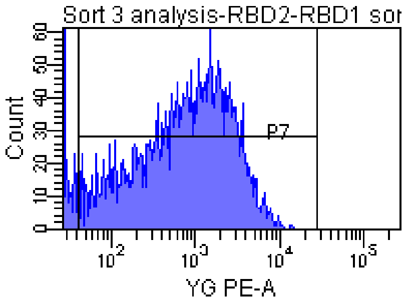

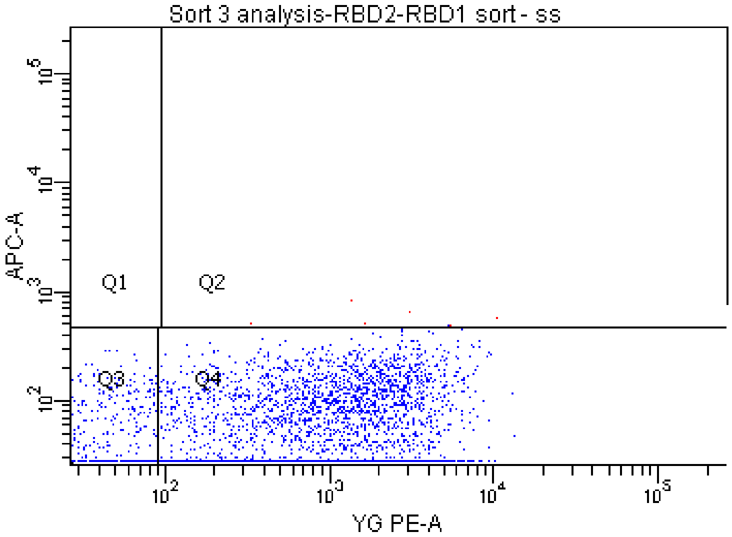

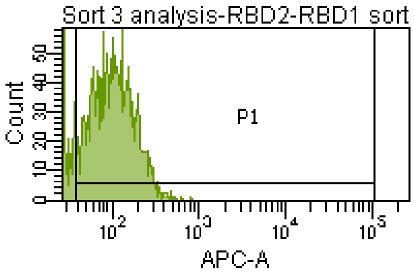

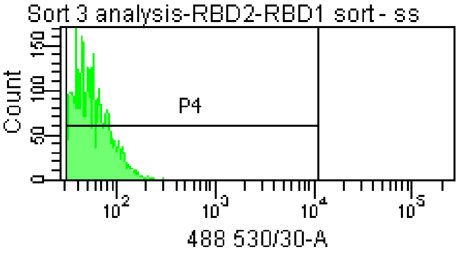

Experiment Name: Bio RBD2 selectons
Specimen Name: Sort 3 analysis
Tube Name: RBD2-RBD1 sort - ss
Record Date: Jun 2, 2020 2:41:25 PM
$OP: Nileena
GUID: 46a1fd95-8baf-4943-8cc0-fe8854d5b0fc

Population	Parent...	#Events	%Parent	%Gran...	%Total	488 5... Mean	488 5... Median	PE-A Mean	PE-A Median	APC-A Mean	APC-A Median
All Events	####	####	####	####	####	64	45	1,220	668	103	45
P2	####	####	####	####	####	63	45	1,212	667	93	45
P8	P2	6,032	60.3	60.3	60.3	37	32	686	290	36	26
Q1	P8	4	0.1	0.0	0.0	-1	-10	-4	-26	3,654	1,447
Q2	P8	6	0.1	0.1	0.1	84	49	2,436	1,930	567	511
Q3	P8	2,587	42.9	25.9	25.9	24	22	45	19	21	16
Q4	P8	3,435	56.9	34.4	34.3	47	41	1,167	936	42	34
P7	P8	3,698	61.3	37.0	37.0	46	40	1,097	856	42	34
P1	P8	2,688	44.6	26.9	26.9	42	36	841	440	119	99
P4	P8	3,076	51.0	30.8	30.7	60	52	1,070	836	48	37

FACSDiva Version 6.1.3

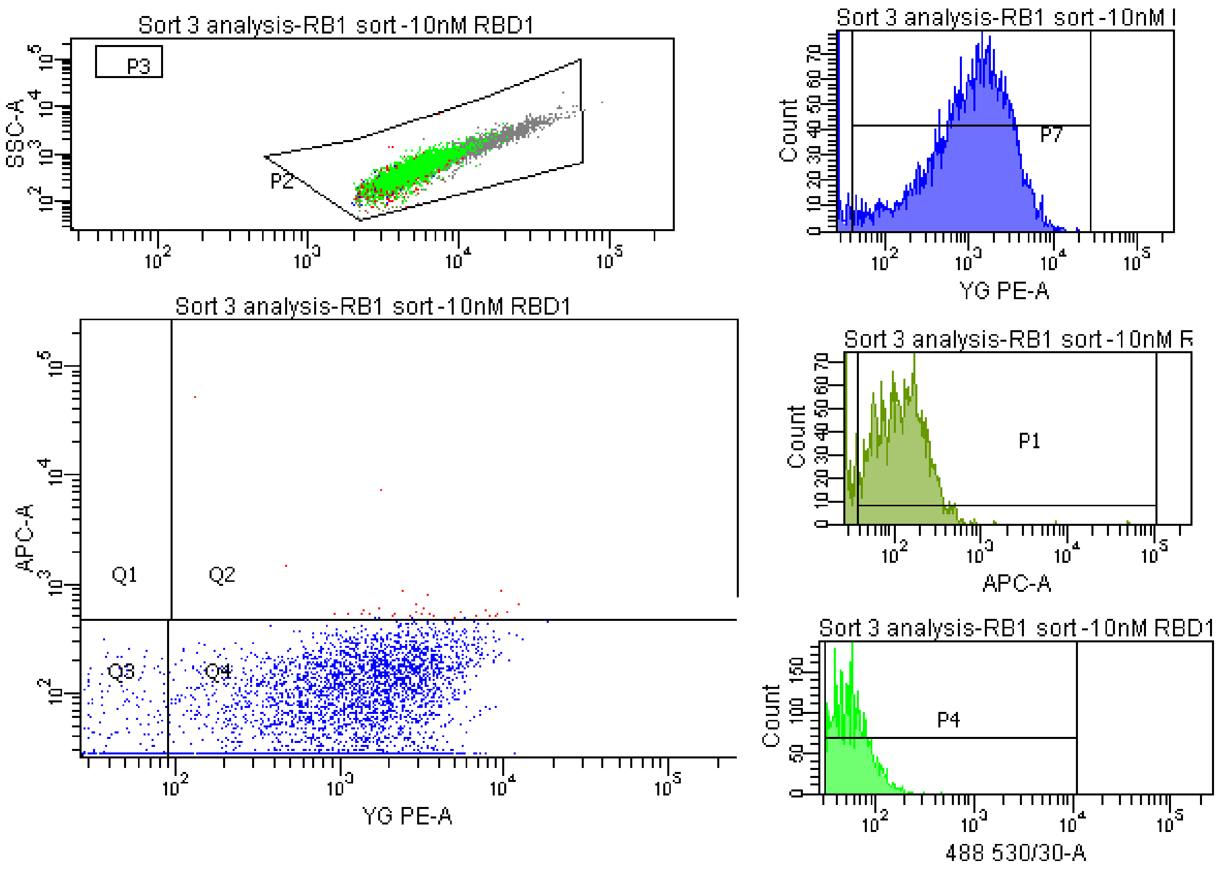

Experiment Name: Bio RBD2 selectons
Specimen Name: Sort 3 analysis
Tube Name: RB1 sort-10nM RBD1
Record Date: Jun 2, 2020 2:42:33 PM
$OP: Nileena
GUID: 7e1f79cf-cc7d-445f-b373-56debe5c88ab

Population	Parent...	#Events	%Parent	%Gran...	%Total	488 5... Mean	488 5... Median	PE-A Mean	PE-A Median	APC-A Mean	APC-A Median
All Events	####	####	####	####	####	65	47	1,541	1,081	128	66
P2	####	####	####	####	####	65	47	1,537	1,080	127	66
P8	P2	6,341	63.4	63.4	63.4	39	34	937	673	68	46
Q1	P8	5	0.1	0.1	0.0	5	13	-4	-4	2,418	912
Q2	P8	33	0.5	0.3	0.3	91	65	2,854	2,445	2,296	515
Q3	P8	1,982	31.3	19.8	19.8	21	20	43	17	20	15
Q4	P8	4,321	68.1	43.2	43.2	47	41	1,334	1,138	71	59
P7	P8	4,508	71.1	45.1	45.1	46	40	1,305	1,100	86	59
P1	P8	3,343	52.7	33.4	33.4	46	40	1,195	995	157	112
P4	P8	3,389	53.4	33.9	33.9	62	54	1,399	1,279	83	67

FACSDiva Version 6.1.3

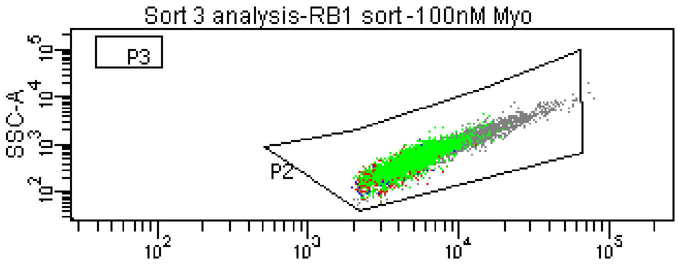

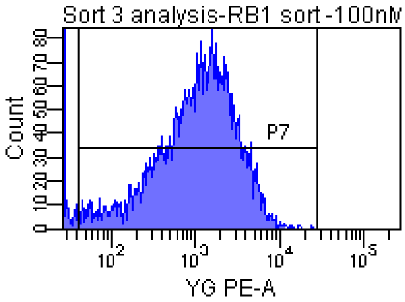

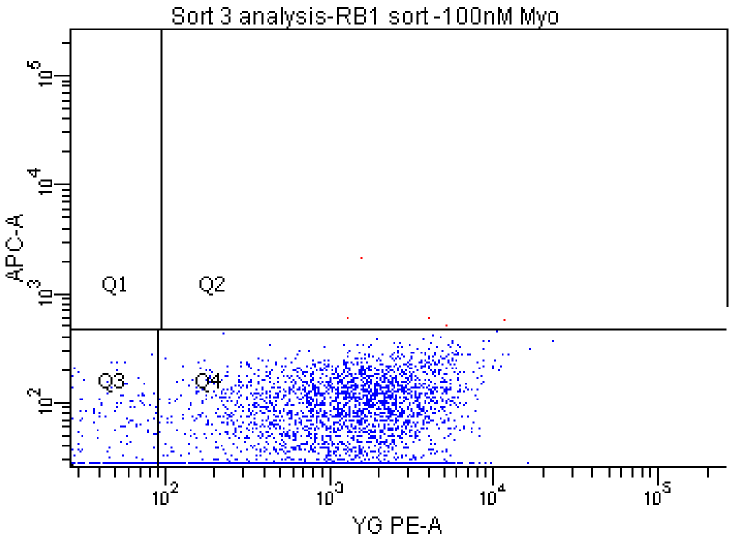

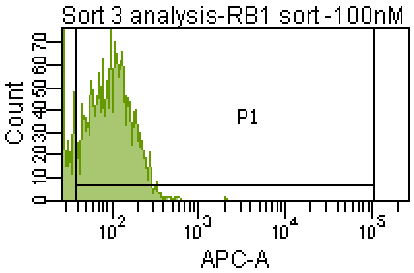

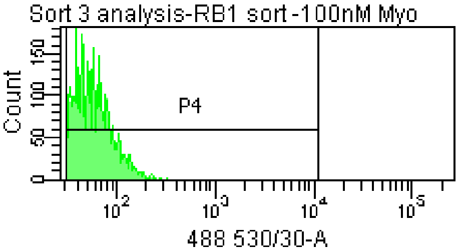

Experiment Name: Bio RBD2 selectons
Specimen Name: Sort 3 analysis
Tube Name: RB1 sort-100nM Myo
Record Date: Jun 2, 2020 2:43:38 PM
$OP: Nileena
GUID: 1415b232-9666-45eb-9ab5-b798680ef4d2

Population	Parent...	#Events	%Parent	%Gran...	%Total	488 5... Mean	488 5... Median	PE-A Mean	PE-A Median	APC-A Mean	APC-A Median
All Events	####	####	####	####	####	64	46	1,518	1,056	77	47
P2	####	####	####	####	####	64	46	1,511	1,056	76	47
P8	P2	6,429	64.3	64.2	64.2	40	34	948	683	40	29
Q1	P8	4	0.1	0.0	0.0	17	16	93	69	2,997	1,253
Q2	P8	5	0.1	0.1	0.0	116	87	3,600	3,577	847	564
Q3	P8	2,026	31.5	20.3	20.2	20	19	43	19	21	11
Q4	P8	4,394	68.3	44.0	43.9	48	43	1,364	1,148	45	37
P7	P8	4,548	70.7	45.5	45.4	48	41	1,326	1,113	45	37
P1	P8	2,949	45.9	29.5	29.5	47	39	1,183	930	117	99
P4	P8	3,465	53.9	34.7	34.6	63	54	1,425	1,308	52	43

FACSDiva Version 6.1.3

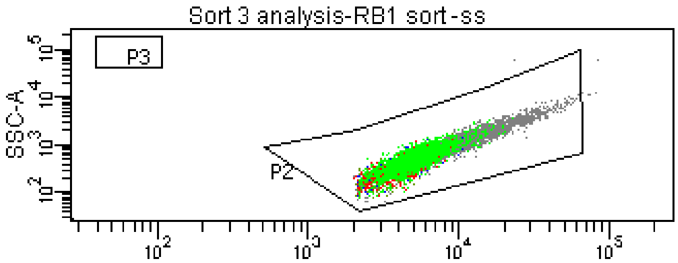

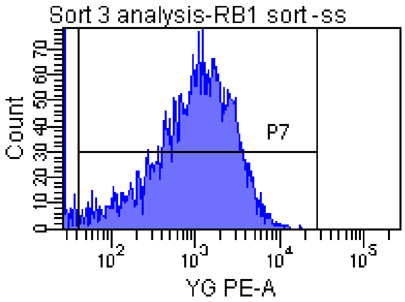

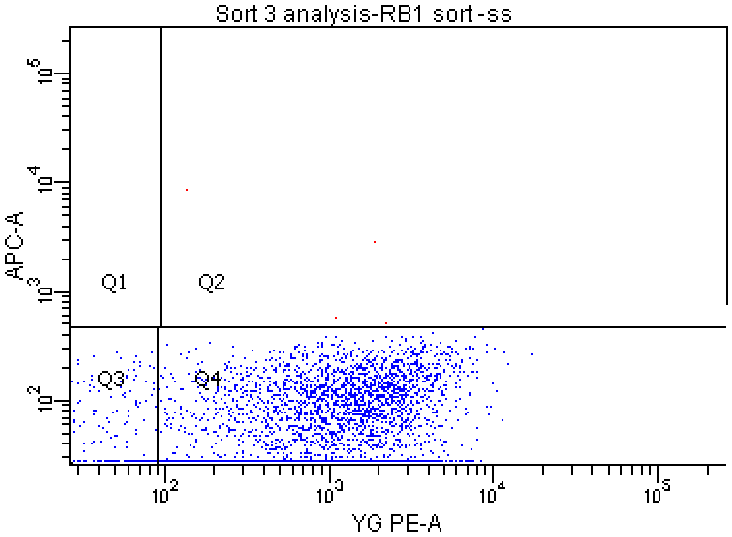

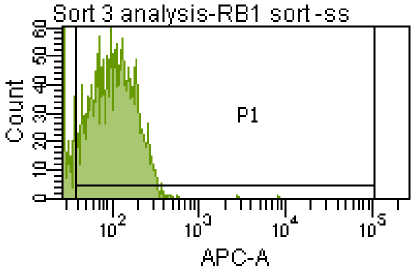

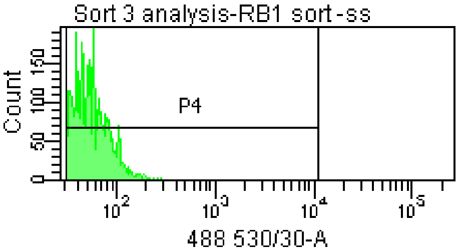

Experiment Name: Bio RBD2 selectons
Specimen Name: Sort 3 analysis
Tube Name: RB1 sort-ss
Record Date: Jun 2, 2020 2:44:44 PM
$OP: Nileena
GUID: 61c5ea73-8c45-412e-b693-e50bf751f2ea

Population	Parent...	#Events	%Parent	%Gran...	%Total	488 5... Mean	488 5... Median	PE-A Mean	PE-A Median	APC-A Mean	APC-A Median
All Events	####	####	####	####	####	66	47	1,474	997	94	50
P2	####	####	####	####	####	65	47	1,458	996	90	50
P8	P2	6,074	60.8	60.7	60.7	38	34	856	597	42	29
Q1	P8	4	0.1	0.0	0.0	4	4	37	2	6,400	1,319
Q2	P8	4	0.1	0.0	0.0	63	50	1,406	1,578	3,006	1,657
Q3	P8	2,003	33.0	20.0	20.0	21	20	48	14	22	17
Q4	P8	4,063	66.9	40.7	40.6	46	41	1,254	1,047	42	37
P7	P8	4,202	69.2	42.0	42.0	45	40	1,220	1,021	44	36
P1	P8	2,828	46.6	28.3	28.3	44	38	1,020	756	131	102
P4	P8	3,301	54.3	33.0	33.0	59	51	1,264	1,148	50	42

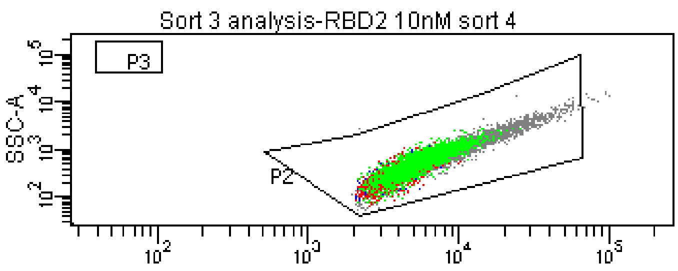

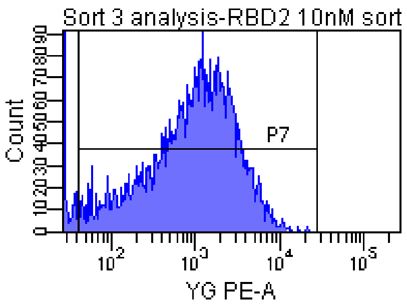

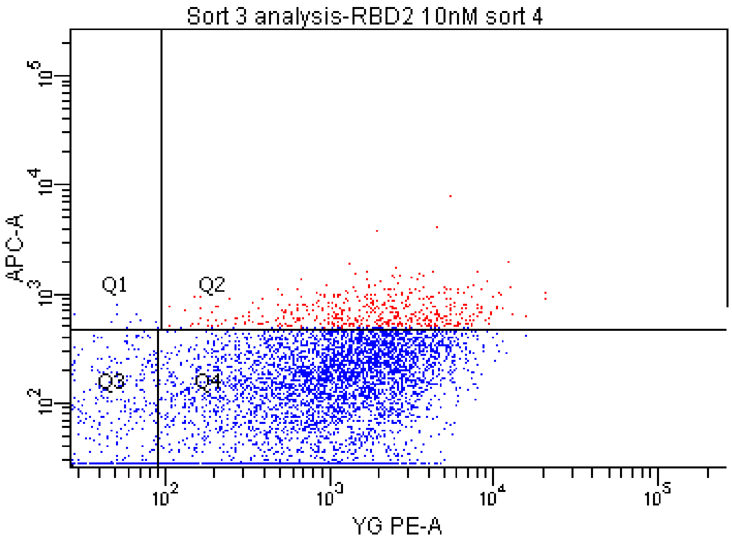

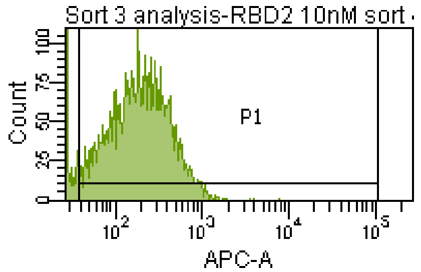

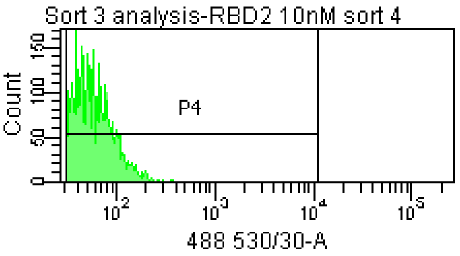

Experiment Name: Bio RBD2 selectons
Specimen Name: Sort 3 analysis
Tube Name: RBD2 10nM sort 4
Record Date: Jun 2, 2020 4:57:43 PM
$OP: Nileena
GUID: fda1df34-b064-45ea-8983-c49c2729781a

Population	Parent...	#Events	%Parent	%Gran...	%Total	488 5... Mean	488 5... Median	PE-A Mean	PE-A Median	APC-A Mean	APC-A Median
All Events	####	####	####	####	####	68	44	1,540	978	327	155
P2	####	####	####	####	####	64	44	1,516	976	293	155
P8	P2	6,589	65.9	65.8	65.8	38	32	957	683	174	112
Q1	P8	12	0.2	0.1	0.1	17	9	94	87	2,785	614
Q2	P8	470	7.1	4.7	4.7	72	62	1,971	1,626	729	628
Q3	P8	1,663	25.2	16.6	16.6	14	12	51	40	36	21
Q4	P8	4,444	67.4	44.4	44.4	44	38	1,191	973	160	139
P7	P8	5,179	78.6	51.8	51.7	45	38	1,206	957	209	153
P1	P8	4,799	72.8	48.0	47.9	45	38	1,168	920	242	173
P4	P8	3,326	50.5	33.3	33.2	64	54	1,529	1,381	237	175

FACSDiva Version 6.1.3

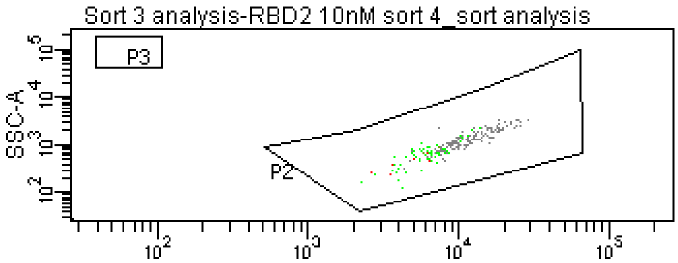

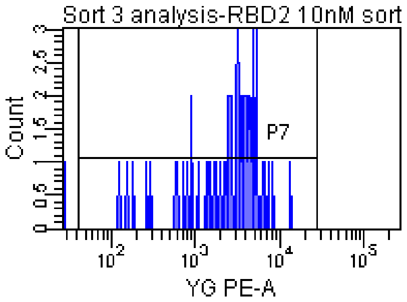

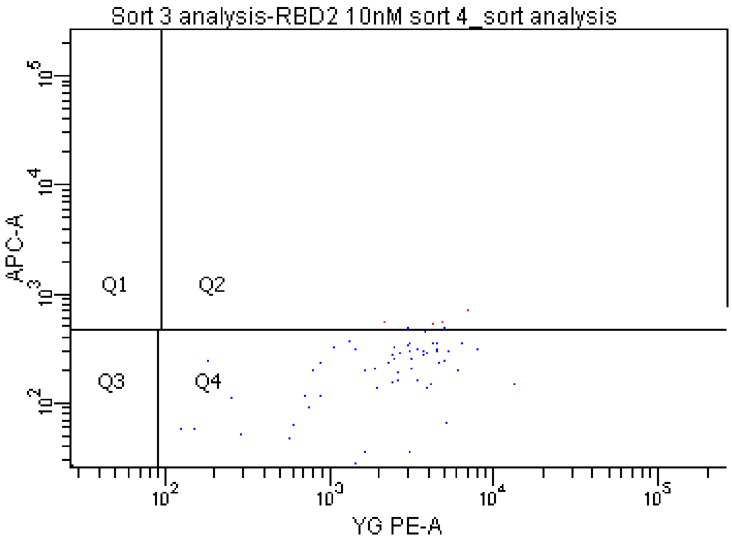

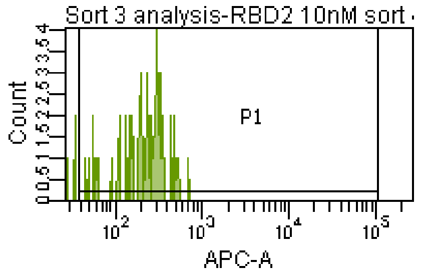

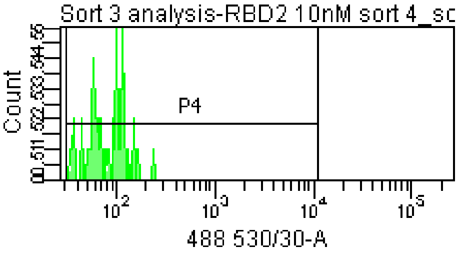

Experiment Name: Bio RBD2 selectons
Specimen Name: Sort 3 analysis
Tube Name: RBD2 10nM sort 4_sort analysis
Record Date: Jun 2, 2020 6:09:07 PM
$OP: Nileena
GUID: c39074bc-873f-430a-a632-7207938e5876

Population	Parent...	#Events	%Parent	%Gran...	%Total	488 5... Mean	488 5... Median	PE-A Mean	PE-A Median	APC-A Mean	APC-A Median
All Events	####	####	####	####	####	109	102	2,654	2,553	383	324
P2	####	####	####	####	####	109	102	2,654	2,553	383	324
P8	P2	62	27.0	27.0	27.0	77	68	2,069	2,041	237	235
Q1	P8	0	0.0	0.0	0.0	####	####	####	####	####	####
Q2	P8	4	6.5	1.7	1.7	134	137	2,877	3,205	558	527
Q3	P8	1	1.6	0.4	0.4	34	34	25	25	15	15
Q4	P8	57	91.9	24.8	24.8	74	65	2,049	2,018	218	227
P7	P8	61	98.4	26.5	26.5	78	69	2,103	2,064	240	235
P1	P8	58	93.5	25.2	25.2	80	72	2,141	2,135	252	238
P4	P8	54	87.1	23.5	23.5	86	81	2,235	2,206	252	238

FACSDiva Version 6.1.3

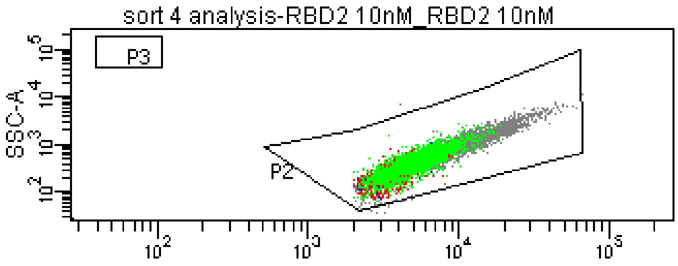

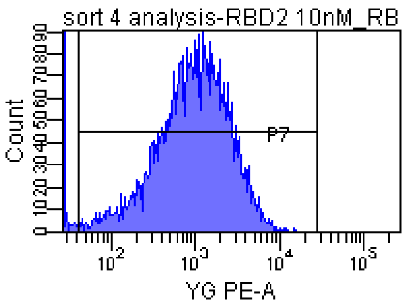

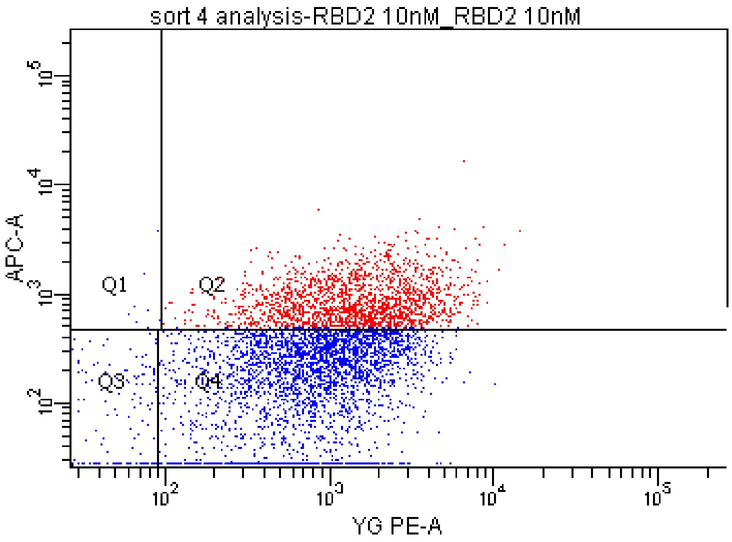

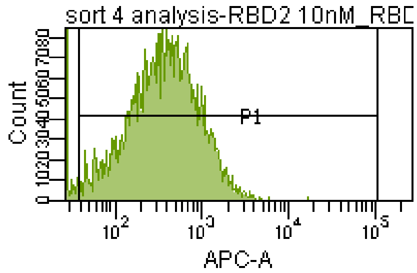

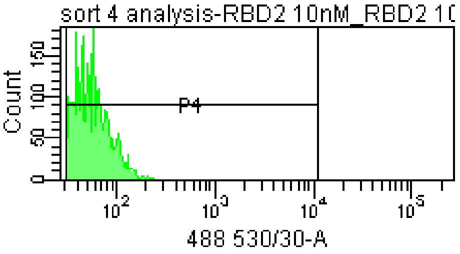

Experiment Name: Bio RBD2 selectons
Specimen Name: sort 4 analysis
Tube Name: RBD2 10nM_RBD2 10nM
Record Date: Jun 5, 2020 2:22:10 PM
$OP: Nileena
GUID: e78a35e2-3b09-4362-b6f7-c2a6b2de59ea

Population	Parent...	#Events	%Parent	%Gran...	%Total	488 5... Mean	488 5... Median	PE-A Mean	PE-A Median	APC-A Mean	APC-A Median
All Events	####	####	####	####	####	65	50	1,658	1,303	655	406
P2	####	####	####	####	####	65	50	1,657	1,305	650	406
P8	P2	5,882	58.9	58.8	58.8	40	35	1,051	941	411	271
Q1	P8	7	0.1	0.1	0.1	16	12	193	192	1,170	678
Q2	P8	1,827	31.1	18.3	18.3	55	49	1,561	1,382	947	772
Q3	P8	921	15.7	9.2	9.2	17	14	46	12	35	21
Q4	P8	3,127	53.2	31.3	31.3	38	35	1,050	964	207	204
P7	P8	5,061	86.0	50.7	50.6	44	39	1,216	1,076	473	336
P1	P8	4,964	84.4	49.7	49.6	43	39	1,179	1,062	492	343
P4	P8	3,319	56.4	33.3	33.2	60	53	1,425	1,325	522	380

FACSDiva Version 6.1.3

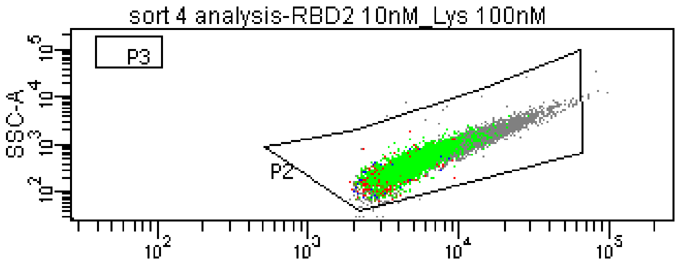

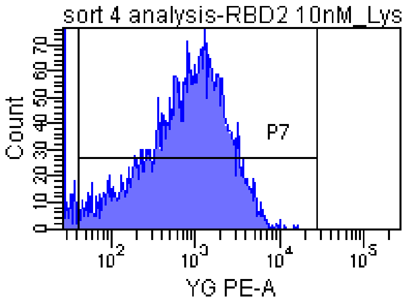

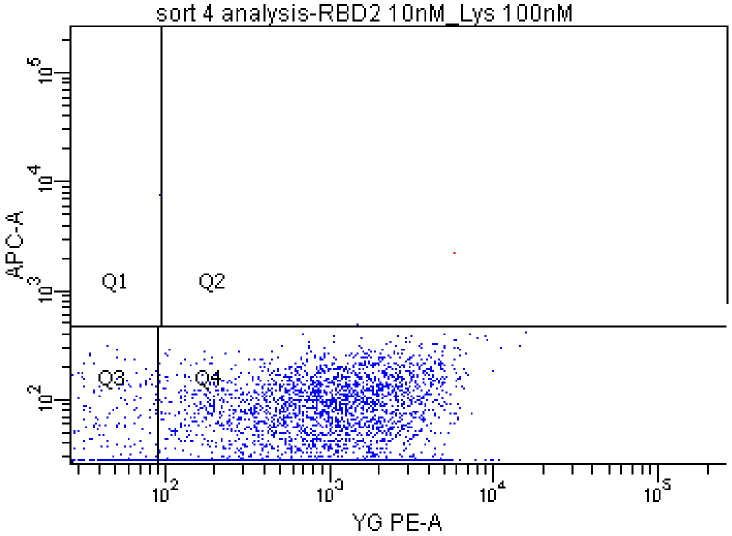

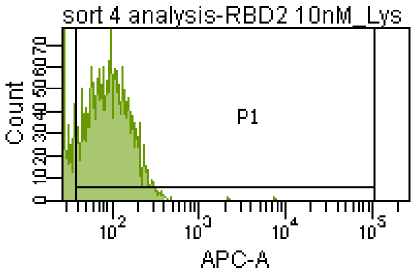

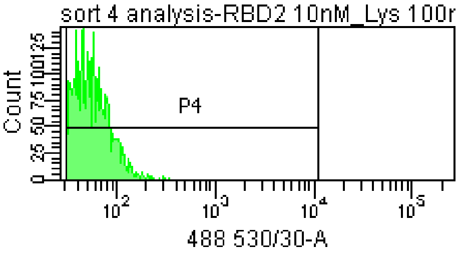

Experiment Name: Bio RBD2 selectons
Specimen Name: sort 4 analysis
Tube Name: RBD2 10nM_Lys 100nM
Record Date: Jun 5, 2020 2:23:37 PM
$OP: Nileena
GUID: ce6a3e5e-8751-4535-8446-ce88ba31f820

Population	Parent...	#Events	%Parent	%Gran...	%Total	488 5... Mean	488 5... Median	PE-A Mean	PE-A Median	APC-A Mean	APC-A Median
All Events	####	####	####	####	####	66	47	1,456	1,022	94	53
P2	####	####	####	####	####	66	47	1,441	1,024	93	53
P8	P2	5,716	57.2	57.1	57.1	37	32	872	672	41	30
Q1	P8	2	0.0	0.0	0.0	-3	-3	100	100	4,733	4,733
Q2	P8	1	0.0	0.0	0.0	291	291	4,707	4,707	2,128	2,128
Q3	P8	1,514	26.5	15.2	15.1	18	15	51	11	19	10
Q4	P8	4,199	73.5	42.0	41.9	44	39	1,168	1,008	46	38
P7	P8	4,400	77.0	44.0	43.9	43	37	1,123	965	47	37
P1	P8	2,627	46.0	26.3	26.2	43	37	1,070	903	111	91
P4	P8	2,908	50.9	29.1	29.0	60	52	1,316	1,245	52	43

FACSDiva Version 6.1.3

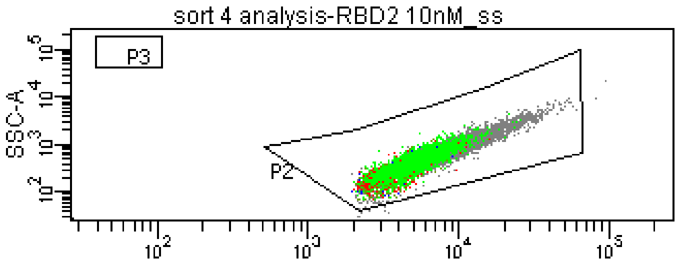

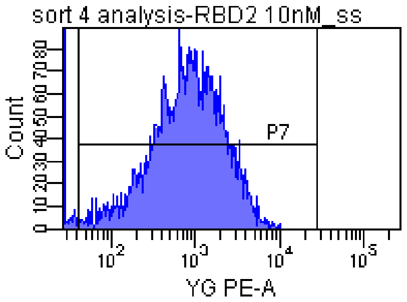

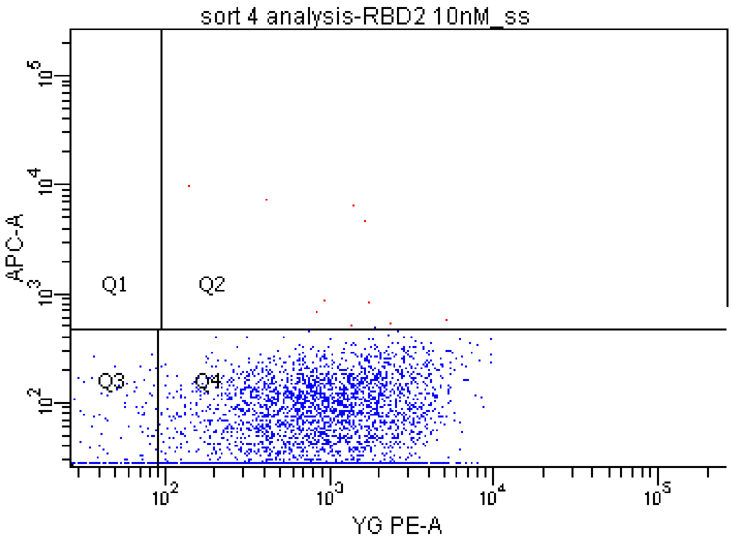

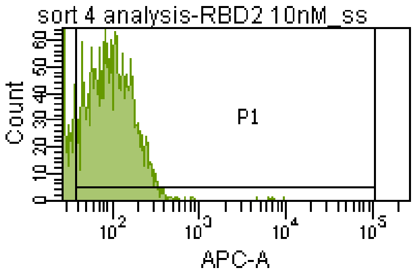

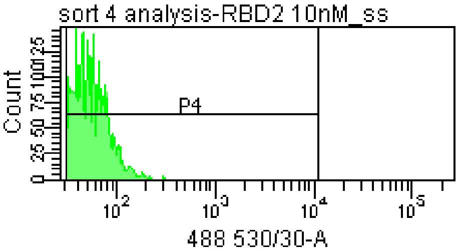

Experiment Name: Bio RBD2 selectons
Specimen Name: sort 4 analysis
Tube Name: RBD2 10nM_ss
Record Date: Jun 5, 2020 2:26:01 PM
$OP: Nileena
GUID: e3e92d01-f693-4574-afa5-63c5c719cb8f

Population	Parent...	#Events	%Parent	%Gran...	%Total	488 5... Mean	488 5... Median	PE-A Mean	PE-A Median	APC-A Mean	APC-A Median
All Events	####	####	####	####	####	64	49	1,472	1,127	88	50
P2	####	####	####	####	####	64	49	1,467	1,128	87	50
P8	P2	5,536	55.4	55.3	55.3	38	33	904	779	43	30
Q1	P8	0	0.0	0.0	0.0	####	####	####	####	####	####
Q2	P8	10	0.2	0.1	0.1	51	35	937	899	3,092	814
Q3	P8	952	17.2	9.5	9.5	17	15	55	12	19	12
Q4	P8	4,574	82.6	45.8	45.7	42	37	1,081	922	41	34
P7	P8	4,706	85.0	47.1	47.0	41	36	1,057	907	48	34
P1	P8	2,554	46.1	25.6	25.5	43	37	1,048	890	124	96
P4	P8	2,908	52.5	29.1	29.1	59	52	1,256	1,128	56	41

FACSDiva Version 6.1.3

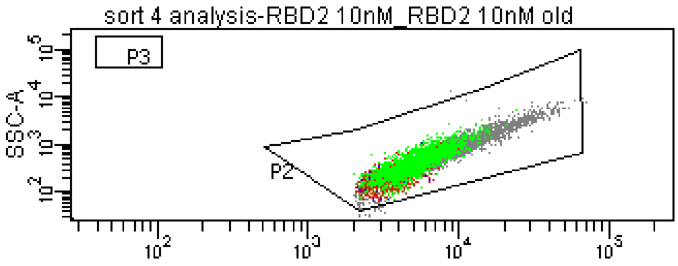

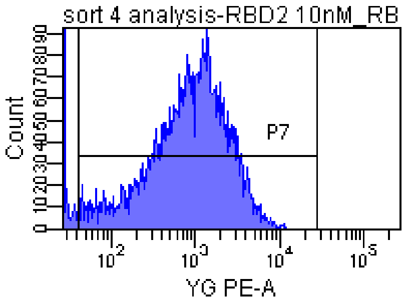

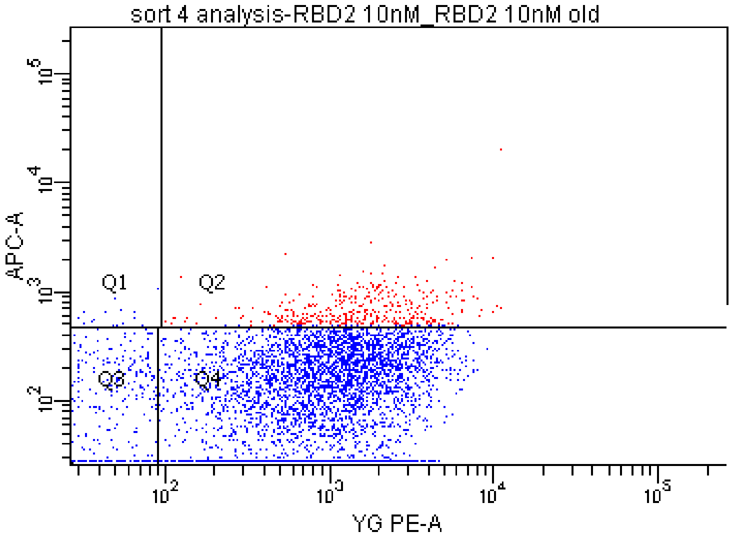

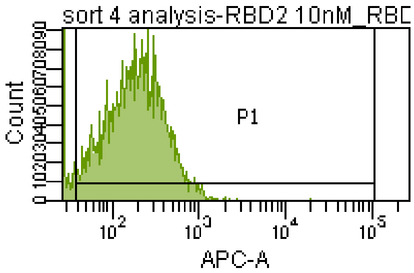

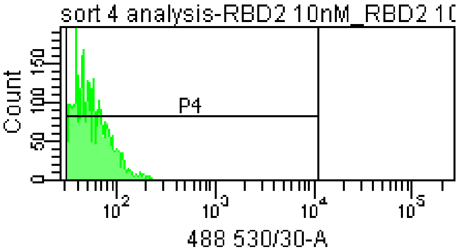

Experiment Name: Bio RBD2 selectons
Specimen Name: sort 4 analysis
Tube Name: RBD2 10nM_RBD2 10nM old
Record Date: Jun 5, 2020 2:29:44 PM
$OP: Nileena
GUID: eff06132-25e7-4b4e-816a-259ed932e41b

Population	Parent...	#Events	%Parent	%Gran...	%Total	488 5... Mean	488 5... Median	PE-A Mean	PE-A Median	APC-A Mean	APC-A Median
All Events	####	####	####	####	####	62	47	1,485	1,167	270	172
P2	####	####	####	####	####	62	47	1,486	1,169	263	172
P8	P2	5,922	59.3	59.1	59.1	37	33	939	816	161	117
Q1	P8	21	0.4	0.2	0.2	16	13	96	60	601	569
Q2	P8	366	6.2	3.7	3.7	62	50	1,650	1,370	757	610
Q3	P8	1,271	21.5	12.7	12.7	16	13	57	22	51	34
Q4	P8	4,264	72.0	42.7	42.6	41	37	1,145	1,025	140	126
P7	P8	4,839	81.7	48.5	48.3	42	37	1,140	1,012	186	140
P1	P8	4,215	71.2	42.2	42.1	41	36	1,088	979	237	178
P4	P8	3,123	52.7	31.3	31.2	59	51	1,342	1,249	203	154

FACSDiva Version 6.1.3

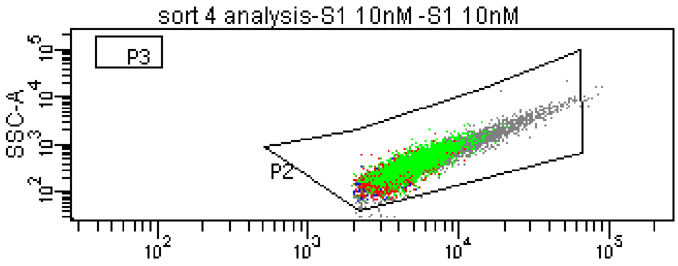

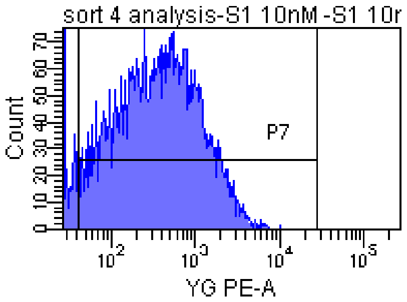

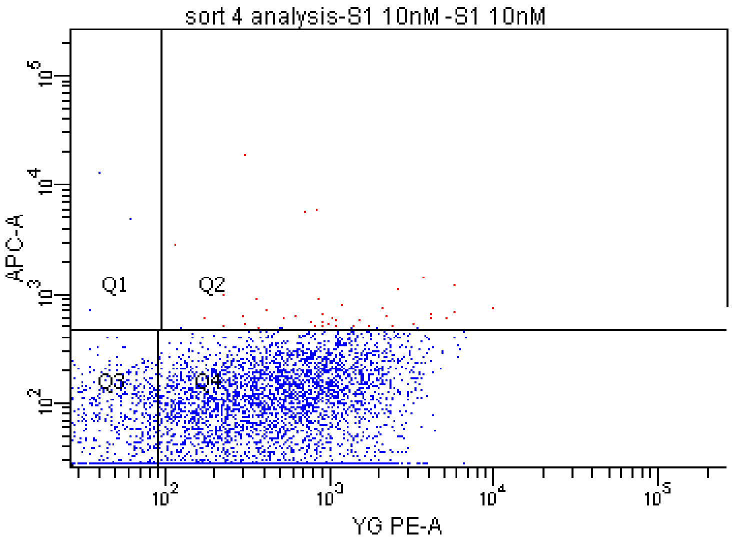

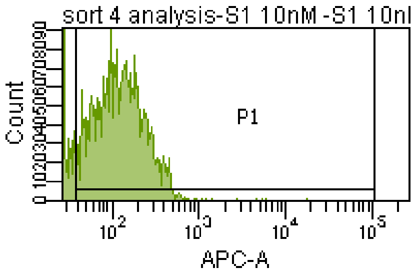

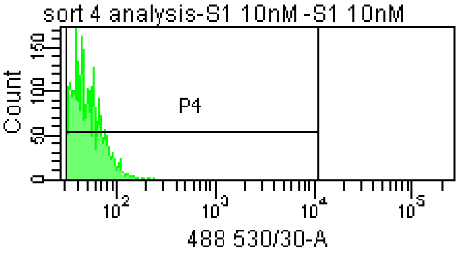

Experiment Name: Bio RBD2 selectons
Specimen Name: sort 4 analysis
Tube Name: S1 10nM -S1 10nM
Record Date: Jun 5, 2020 2:31:19 PM
$OP: Nileena
GUID: 1175b74a-d578-4cc8-bc74-47de2cd87fb7

Population	Parent...	#Events	%Parent	%Gran...	%Total	488 5... Mean	488 5... Median	PE-A Mean	PE-A Median	APC-A Mean	APC-A Median
All Events	####	####	####	####	####	44	31	656	374	108	66
P2	####	####	####	####	####	44	31	649	375	106	66
P8	P2	6,929	69.4	69.2	69.2	27	24	435	277	74	48
Q1	P8	6	0.1	0.1	0.1	13	6	106	53	3,661	1,650
Q2	P8	41	0.6	0.4	0.4	56	46	1,387	942	1,357	578
Q3	P8	2,233	32.2	22.4	22.3	14	13	60	35	25	17
Q4	P8	4,649	67.1	46.6	46.4	33	29	607	460	81	67
P7	P8	5,328	76.9	53.4	53.2	32	28	555	404	86	61
P1	P8	3,773	54.5	37.8	37.7	32	27	550	392	155	114
P4	P8	2,586	37.3	25.9	25.8	53	47	719	564	99	80

FACSDiva Version 6.1.3

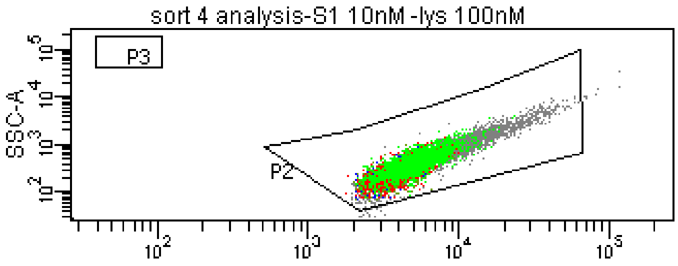

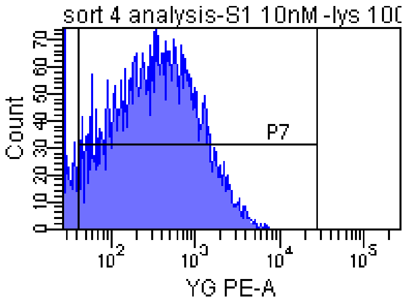

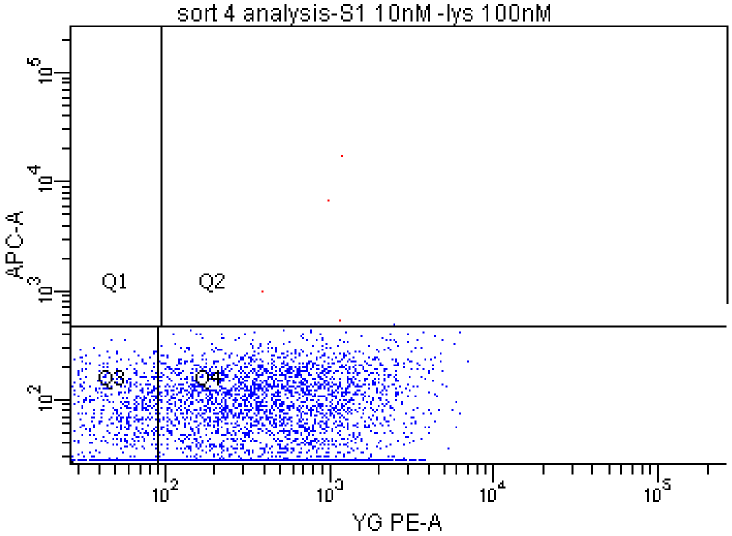

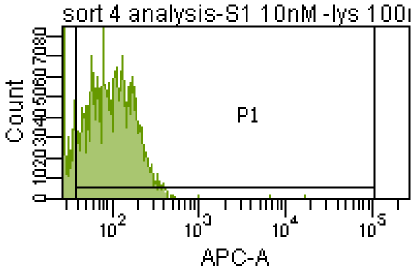

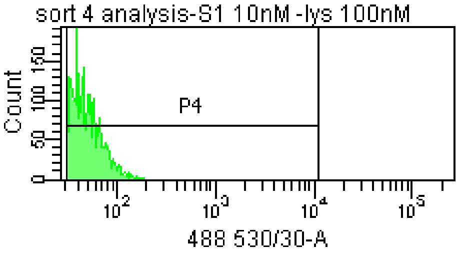

Experiment Name: Bio RBD2 selectons
Specimen Name: sort 4 analysis
Tube Name: S1 10nM -lys 100nM
Record Date: Jun 5, 2020 2:32:42 PM
$OP: Nileena
GUID: 704eecab-9a3e-40cc-801e-360cb79cfe06

Population	Parent...	#Events	%Parent	%Gran...	%Total	488 5... Mean	488 5... Median	PE-A Mean	PE-A Median	APC-A Mean	APC-A Median
All Events	####	####	####	####	####	44	31	626	353	63	42
P2	####	####	####	####	####	43	31	620	353	62	42
P8	P2	6,840	68.5	68.3	68.3	27	24	406	258	40	30
Q1	P8	0	0.0	0.0	0.0	####	####	####	####	####	####
Q2	P8	4	0.1	0.0	0.0	48	55	898	906	6,138	3,764
Q3	P8	2,287	33.4	22.9	22.8	15	14	53	29	25	21
Q4	P8	4,549	66.5	45.6	45.4	32	29	583	433	42	34
P7	P8	5,224	76.4	52.3	52.2	31	28	523	372	45	34
P1	P8	3,183	46.5	31.9	31.8	30	26	468	292	124	101
P4	P8	2,563	37.5	25.7	25.6	52	46	663	498	59	43

FACSDiva Version 6.1.3

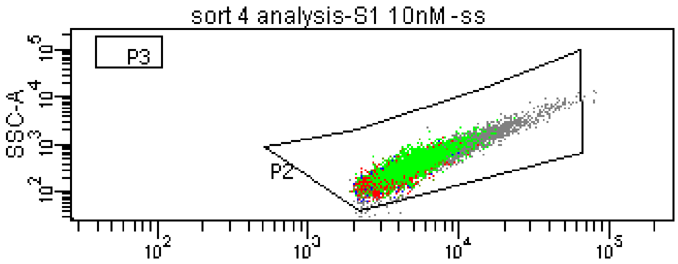

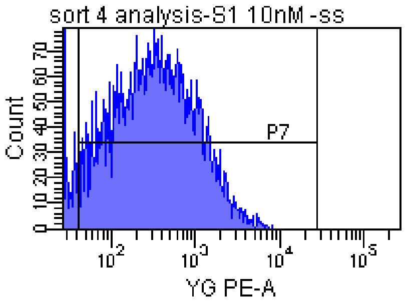

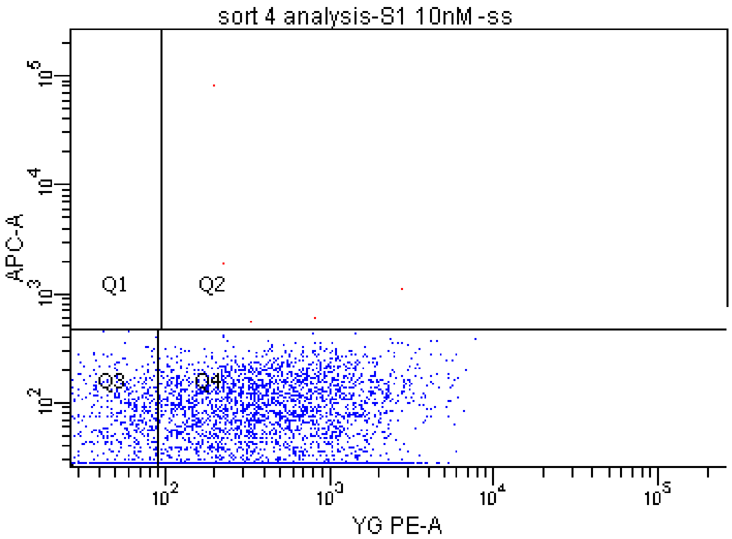

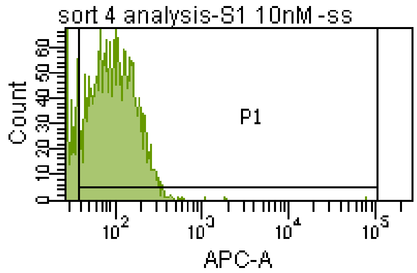

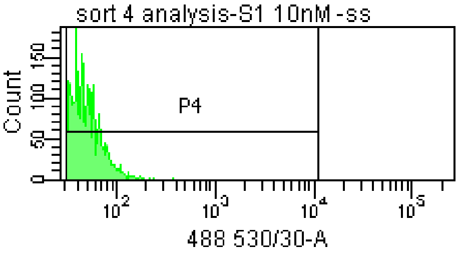

Experiment Name: Bio RBD2 selectons
Specimen Name: sort 4 analysis
Tube Name: S1 10nM -ss
Record Date: Jun 5, 2020 2:35:31 PM
$OP: Nileena
GUID: d1a45c23-50c9-491c-9ad2-8011afbfc35a

Population	Parent...	#Events	%Parent	%Gran...	%Total	488 5... Mean	488 5... Median	PE-A Mean	PE-A Median	APC-A Mean	APC-A Median
All Events	####	####	####	####	####	42	29	610	334	64	35
P2	####	####	####	####	####	42	29	604	334	64	36
P8	P2	6,984	70.0	69.7	69.7	26	23	405	253	44	24
Q1	P8	2	0.0	0.0	0.0	-2	-2	166	166	3,059	3,059
Q2	P8	5	0.1	0.1	0.0	38	33	656	393	16,419	1,056
Q3	P8	2,293	32.8	23.0	22.9	14	13	55	35	23	18
Q4	P8	4,684	67.1	46.9	46.8	32	28	576	419	35	27
P7	P8	5,388	77.1	54.0	53.8	30	27	516	356	49	26
P1	P8	3,029	43.4	30.3	30.2	30	26	468	288	141	97
P4	P8	2,510	35.9	25.1	25.1	52	46	671	503	78	40

FACSDiva Version 6.1.3

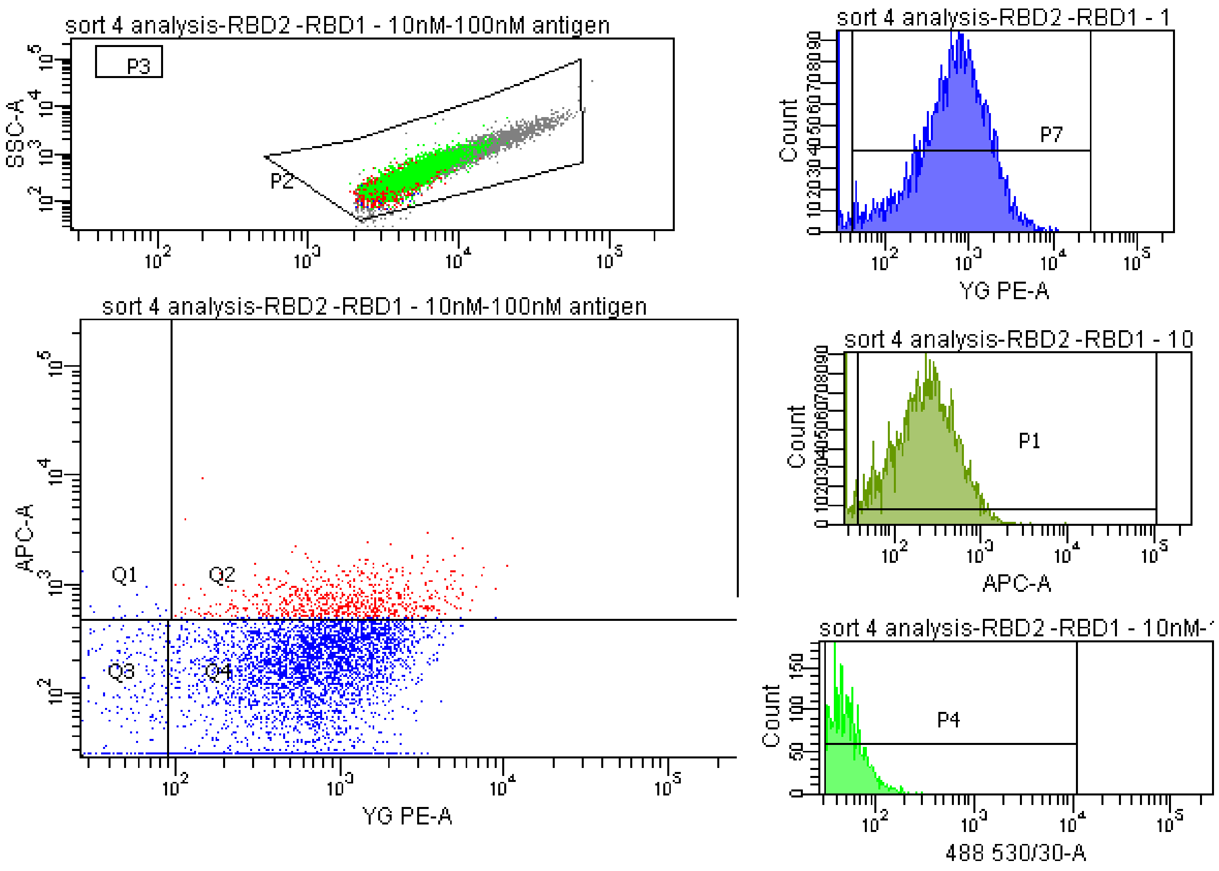

Experiment Name: Bio RBD2 selectons
Specimen Name: sort 4 analysis
Tube Name: RBD2 -RBD1 - 10nM-100nM antigen
Record Date: Jun 5, 2020 2:36:59 PM
$OP: Nileena
GUID: 23ba4e41-f2f5-4164-880c-17f36143e430

Population	Parent...	#Events	%Parent	%Gran...	%Total	488 5... Mean	488 5... Median	PE-A Mean	PE-A Median	APC-A Mean	APC-A Median
All Events	####	####	####	####	####	67	41	1,133	829	414	229
P2	####	####	####	####	####	60	41	1,126	832	391	230
P8	P2	5,920	59.3	59.1	59.1	32	28	663	595	221	157
Q1	P8	31	0.5	0.3	0.3	19	23	58	9	1,574	648
Q2	P8	713	12.0	7.1	7.1	51	44	1,116	935	760	649
Q3	P8	1,210	20.4	12.1	12.1	14	12	42	-2	42	20
Q4	P8	3,966	67.0	39.7	39.6	35	32	776	701	168	160
P7	P8	4,862	82.1	48.7	48.5	36	33	802	712	254	190
P1	P8	4,463	75.4	44.7	44.5	36	32	766	690	304	223
P4	P8	2,712	45.8	27.2	27.1	56	49	938	864	283	217

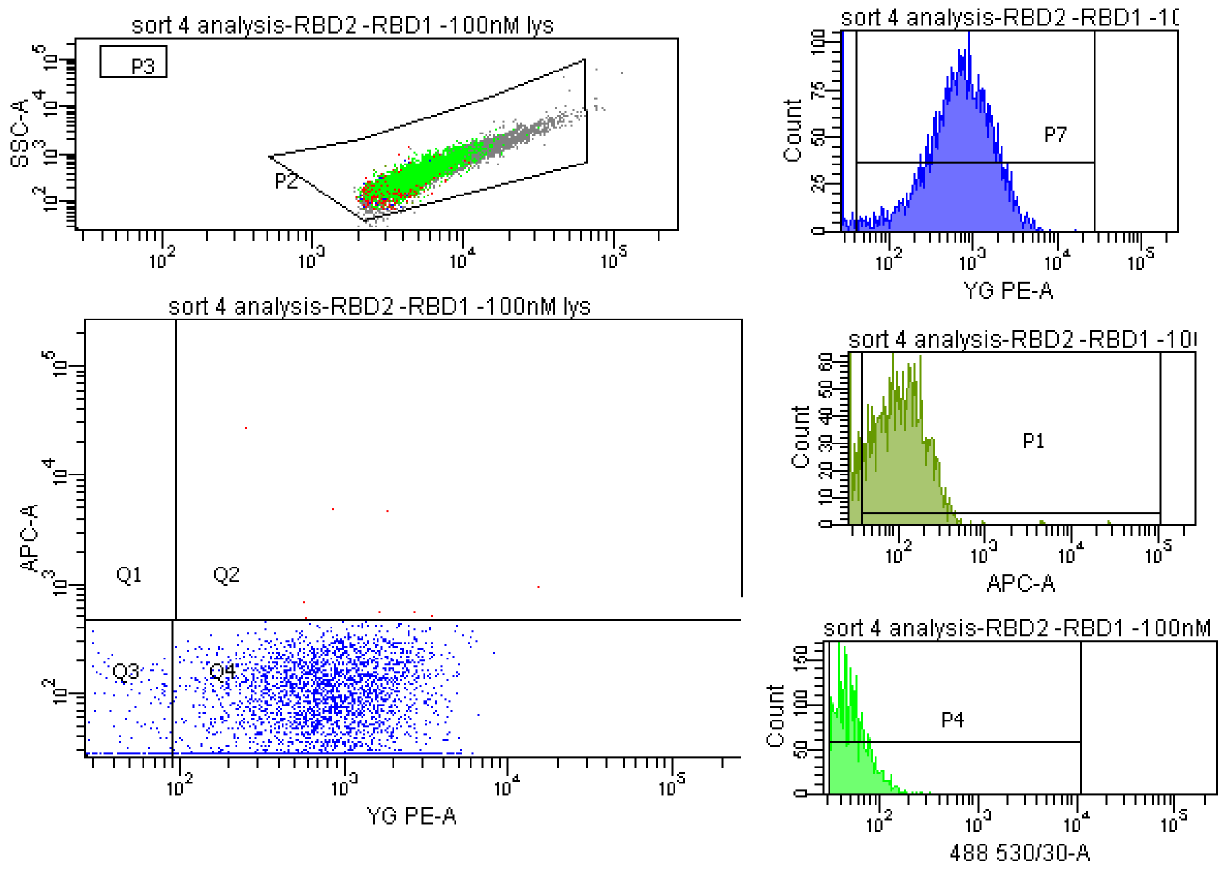

Experiment Name: Bio RBD2 selectons
Specimen Name: sort 4 analysis
Tube Name: RBD2 -RBD1 -100nM lys
Record Date: Jun 5, 2020 2:39:04 PM
$OP: Nileena
GUID: 16c23de8-5845-46e4-8283-9642655841eb

Population	Parent...	#Events	%Parent	%Gran...	%Total	488 5... Mean	488 5... Median	PE-A Mean	PE-A Median	APC-A Mean	APC-A Median
All Events	####	####	####	####	####	59	41	1,132	835	223	48
P2	####	####	####	####	####	58	41	1,105	838	196	48
P8	P2	5,946	59.6	59.3	59.3	33	28	683	620	44	28
Q1	P8	3	0.1	0.0	0.0	10	13	144	69	3,288	3,181
Q2	P8	9	0.2	0.1	0.1	77	44	1,766	1,272	4,310	653
Q3	P8	1,218	20.5	12.2	12.1	14	12	35	-3	22	15
Q4	P8	4,716	79.3	47.3	47.0	38	34	849	742	40	30
P7	P8	4,848	81.5	48.6	48.3	37	33	833	729	47	30
P1	P8	2,726	45.8	27.3	27.2	36	33	755	678	145	109
P4	P8	2,759	46.4	27.6	27.5	56	49	960	876	52	41

FACSDiva Version 6.1.3

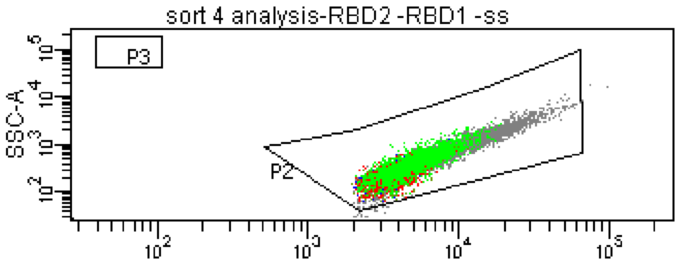

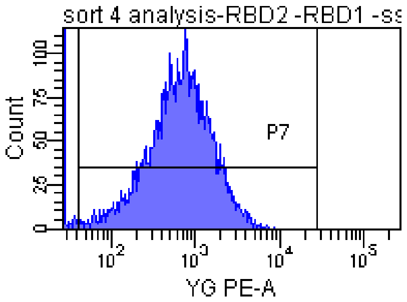

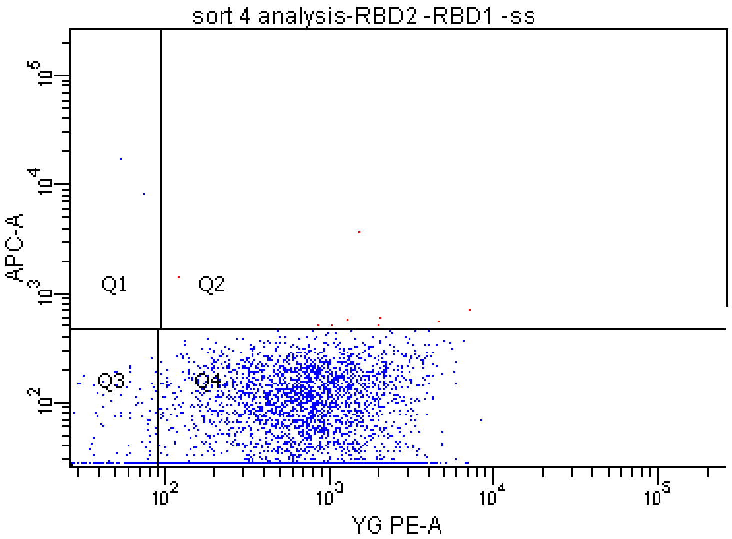

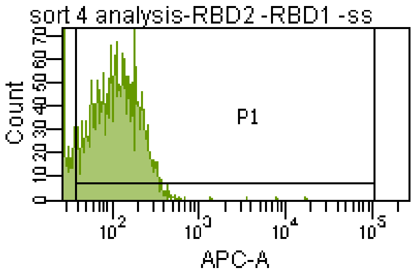

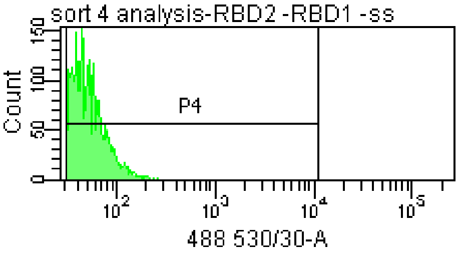

Experiment Name: Bio RBD2 selectons
Specimen Name: sort 4 analysis
Tube Name: RBD2 -RBD1 -ss
Record Date: Jun 5, 2020 2:41:41 PM
$OP: Nileena
GUID: 462f02e4-b4f7-4d07-a173-aa9d7daafd8c

Population	Parent...	#Events	%Parent	%Gran...	%Total	488 5... Mean	488 5... Median	PE-A Mean	PE-A Median	APC-A Mean	APC-A Median
All Events	####	####	####	####	####	55	39	1,061	806	78	41
P2	####	####	####	####	####	55	39	1,059	809	78	41
P8	P2	5,986	59.9	59.7	59.7	32	28	669	611	34	21
Q1	P8	2	0.0	0.0	0.0	22	22	359	359	12,331	12,331
Q2	P8	9	0.2	0.1	0.1	90	61	1,450	1,292	951	544
Q3	P8	1,036	17.3	10.4	10.3	13	12	42	5	14	10
Q4	P8	4,939	82.5	49.5	49.3	35	32	800	708	32	25
P7	P8	5,061	84.5	50.7	50.5	35	32	787	695	38	25
P1	P8	2,647	44.2	26.5	26.4	35	31	726	655	141	112
P4	P8	2,709	45.3	27.1	27.0	55	48	924	852	46	32

FACSDiva Version 6.1.3

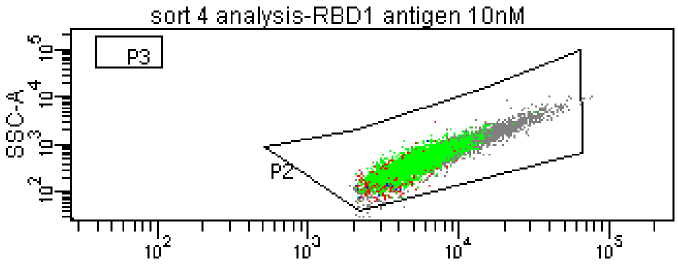

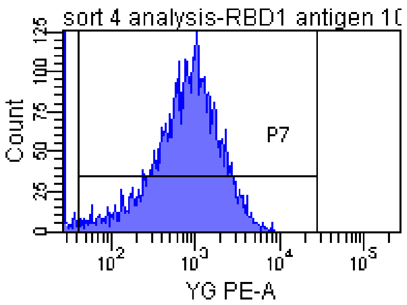

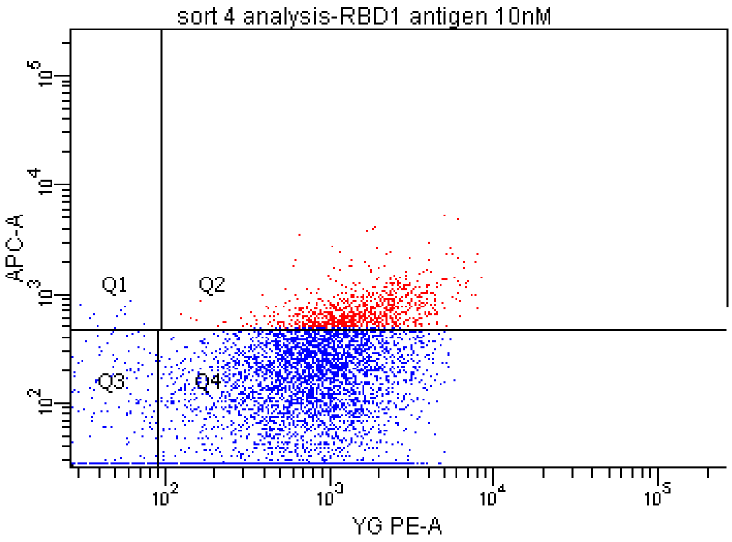

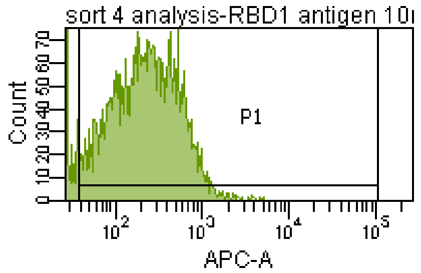

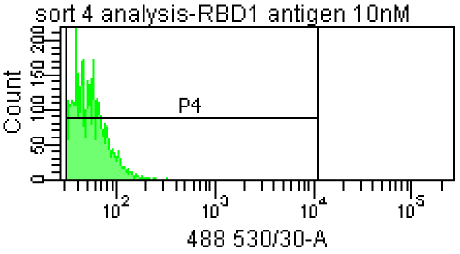

Experiment Name: Bio RBD2 selectons
Specimen Name: sort 4 analysis
Tube Name: RBD1 antigen 10nM
Record Date: Jun 5, 2020 2:43:36 PM
$OP: Nileena
GUID: d43cfc80-5447-49d5-baf6-347879a412c1

Population	Parent...	#Events	%Parent	%Gran...	%Total	488 5... Mean	488 5... Median	PE-A Mean	PE-A Median	APC-A Mean	APC-A Median
All Events	####	####	####	####	####	59	44	1,247	942	320	177
P2	####	####	####	####	####	59	44	1,244	942	320	177
P8	P2	6,423	64.3	64.2	64.2	36	33	810	741	204	124
Q1	P8	16	0.2	0.2	0.2	23	20	82	63	818	629
Q2	P8	861	13.4	8.6	8.6	60	53	1,503	1,339	768	638
Q3	P8	937	14.6	9.4	9.4	14	13	52	13	42	26
Q4	P8	4,609	71.8	46.1	46.0	36	34	838	766	129	108
P7	P8	5,626	87.6	56.3	56.2	39	36	920	821	226	148
P1	P8	4,518	70.3	45.2	45.1	40	36	924	843	306	214
P4	P8	3,360	52.3	33.6	33.6	57	50	1,087	995	274	180

FACSDiva Version 6.1.3

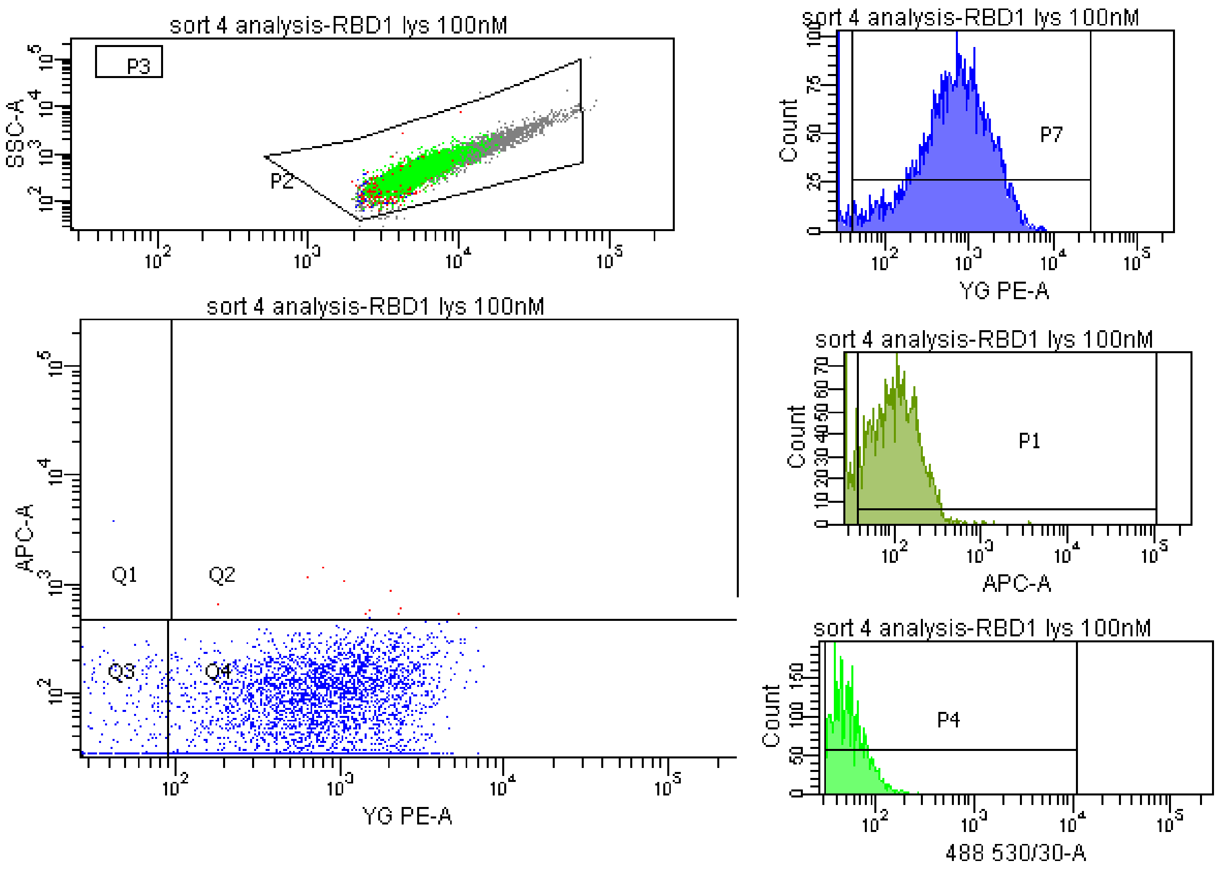

Experiment Name: Bio RBD2 selectons
Specimen Name: sort 4 analysis
Tube Name: RBD1 lys 100nM
Record Date: Jun 5, 2020 2:50:22 PM
$OP: Nileena
GUID: 20e2c42f-d5b3-4cb7-990f-0ff715064a05

Population	Parent...	#Events	%Parent	%Gran...	%Total	488 5... Mean	488 5... Median	PE-A Mean	PE-A Median	APC-A Mean	APC-A Median
All Events	####	####	####	####	####	61	43	1,213	886	142	53
P2	####	####	####	####	####	61	43	1,203	887	105	53
P8	P2	6,263	62.7	62.5	62.5	35	32	736	651	55	34
Q1	P8	5	0.1	0.1	0.0	-4	-5	212	137	14,514	3,601
Q2	P8	10	0.2	0.1	0.1	66	61	1,561	1,556	758	601
Q3	P8	1,402	22.4	14.0	14.0	16	15	52	11	22	15
Q4	P8	4,846	77.4	48.5	48.4	41	37	932	826	48	40
P7	P8	5,058	80.8	50.7	50.5	40	37	903	799	50	40
P1	P8	3,013	48.1	30.2	30.1	40	36	839	752	146	105
P4	P8	3,173	50.7	31.8	31.7	57	50	1,031	965	57	47

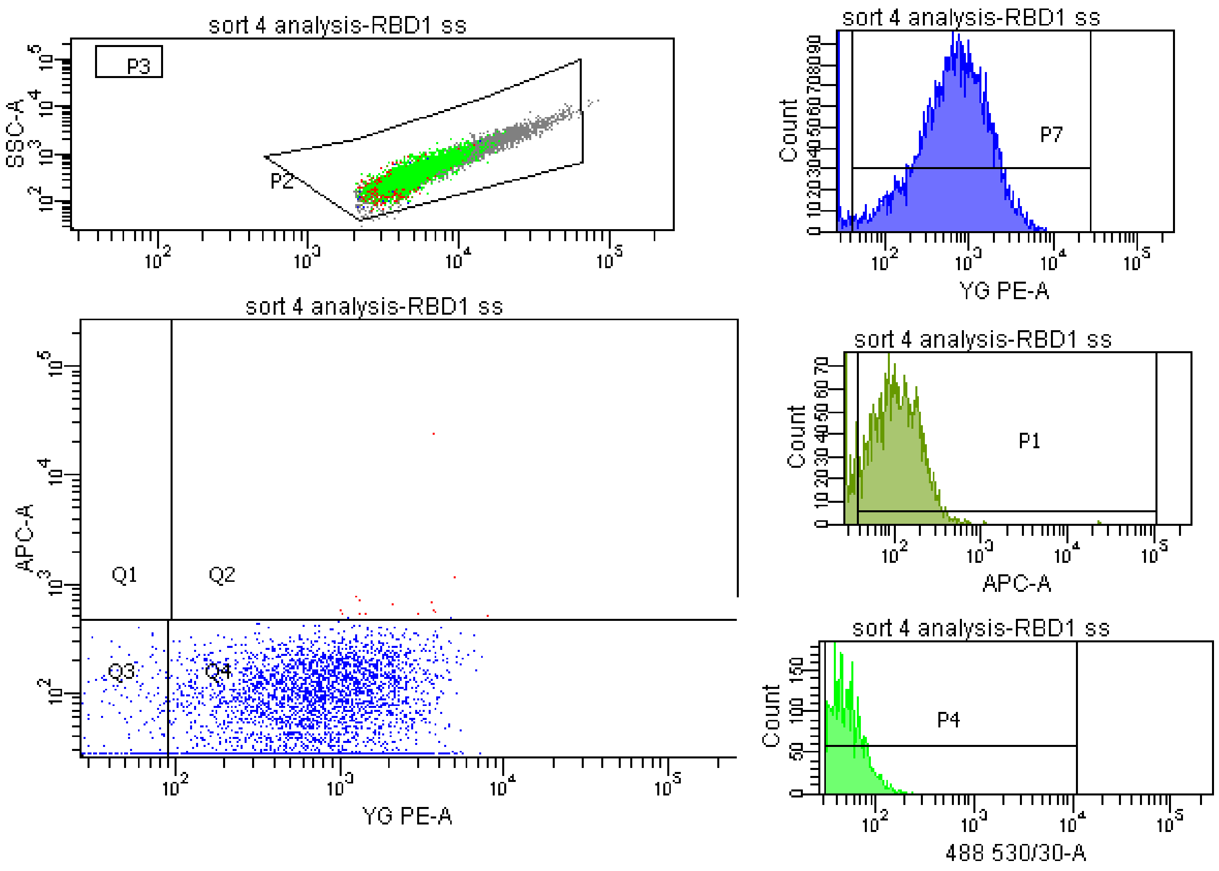

Experiment Name: Bio RBD2 selectons
Specimen Name: sort 4 analysis
Tube Name: RBD1 ss
Record Date: Jun 5, 2020 2:51:26 PM
$OP: Nileena
GUID: dbde48be-0f9e-4a85-950d-b2f92c22529e

Population	Parent...	#Events	%Parent	%Gran...	%Total	488 5... Mean	488 5... Median	PE-A Mean	PE-A Median	APC-A Mean	APC-A Median
All Events	####	####	####	####	####	60	43	1,161	871	96	55
P2	####	####	####	####	####	59	43	1,153	871	94	55
P8	P2	6,169	61.8	61.6	61.6	35	32	740	671	49	34
Q1	P8	5	0.1	0.1	0.0	7	5	33	-2	3,113	662
Q2	P8	14	0.2	0.1	0.1	83	67	1,740	1,327	2,219	552
Q3	P8	1,093	17.7	10.9	10.9	17	15	53	14	23	13
Q4	P8	5,057	82.0	50.6	50.5	39	35	886	787	46	38
P7	P8	5,238	84.9	52.4	52.3	38	35	866	767	51	38
P1	P8	2,960	48.0	29.6	29.6	38	34	828	749	139	107
P4	P8	3,129	50.7	31.3	31.3	56	49	997	931	62	45

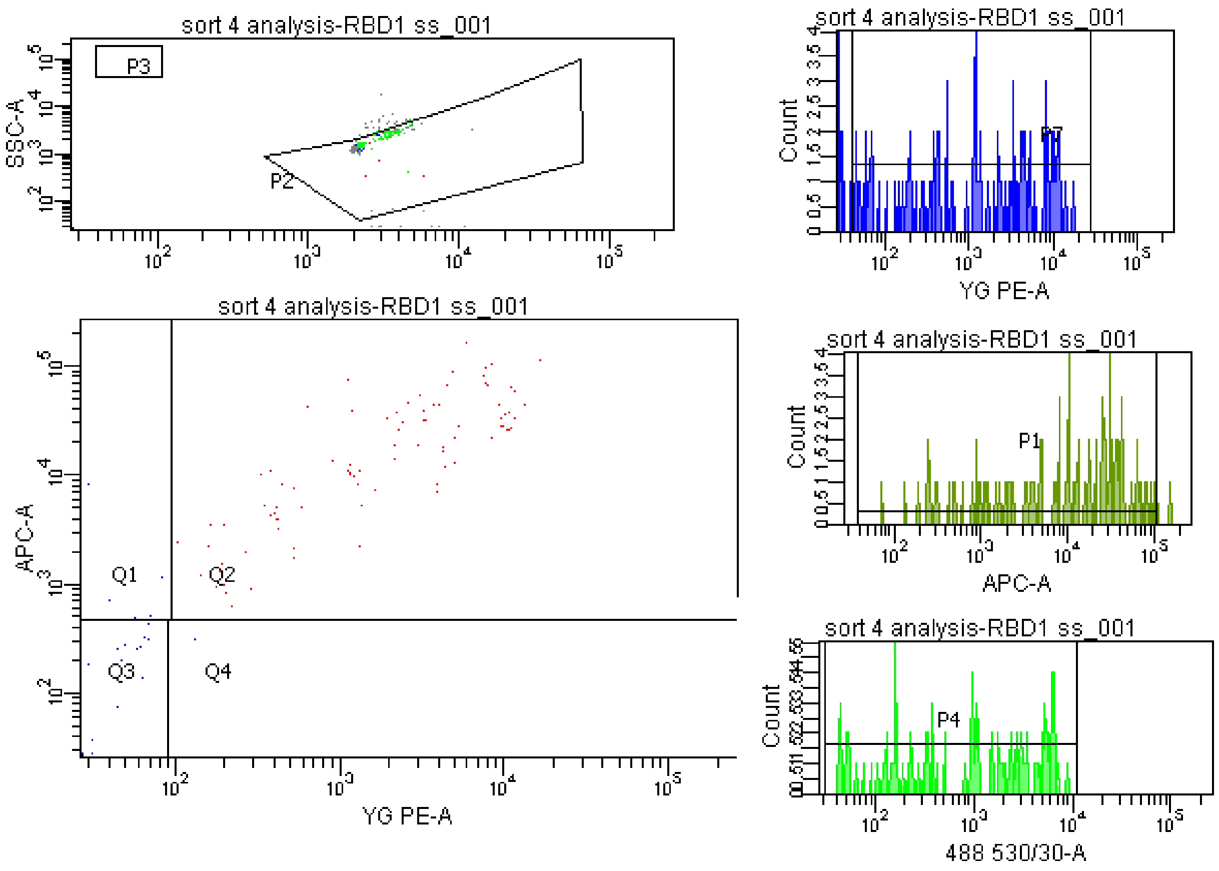

Experiment Name: Bio RBD2 selectons
Specimen Name: sort 4 analysis
Tube Name: RBD1 ss_001
Record Date: Jun 9, 2020 2:41:02 PM
$OP: Nileena
GUID: 229bdbf9-ece6-4730-8b44-7ce7f9961cd6

Population	Parent...	#Events	%Parent	%Gran...	%Total	488 5... Mean	488 5... Median	PE-A Mean	PE-A Median	APC-A Mean	APC-A Median
All Events	####	####	####	####	####	1,577	328	1,894	657	18,631	4,154
P2	####	####	####	####	####	1,530	324	1,826	620	18,782	3,844
P8	P2	135	52.9	47.5	47.5	1,529	340	1,872	754	16,901	4,925
Q1	P8	7	5.2	2.7	2.5	86	74	387	392	3,779	1,121
Q2	P8	88	65.2	34.5	31.0	2,330	1,503	2,722	1,854	25,587	13,290
Q3	P8	39	28.9	15.3	13.7	16	13	257	222	85	35
Q4	P8	1	0.7	0.4	0.4	117	117	533	533	302	302
P7	P8	102	75.6	40.0	35.9	2,017	1,023	2,398	1,296	22,122	10,392
P1	P8	113	83.7	44.3	39.8	1,706	794	2,058	1,100	17,837	7,986
P4	P8	104	77.0	40.8	36.6	1,983	1,005	2,359	1,248	21,920	10,329

FACSDiva Version 6.1.3

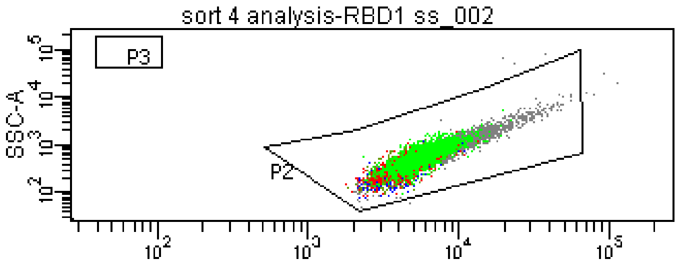

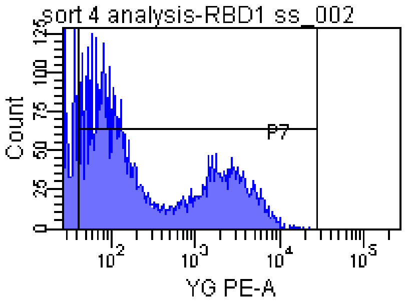

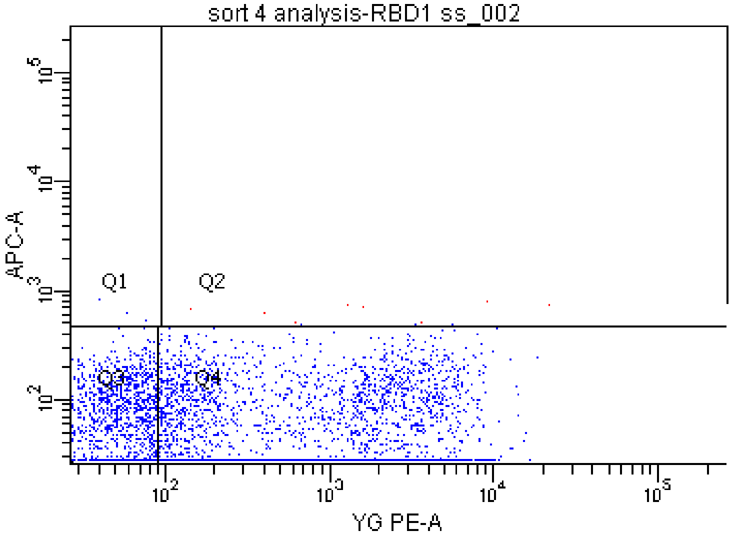

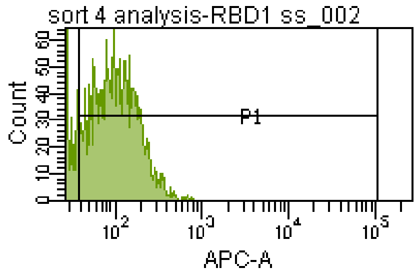

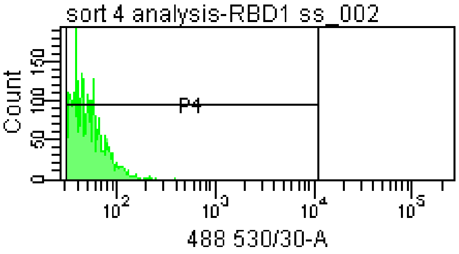

Experiment Name: Bio RBD2 selectons
Specimen Name: sort 4 analysis
Tube Name: RBD1 ss_002
Record Date: Jun 8, 2020 6:18:07 PM
$OP: Nileena
GUID: 87f4411f-e718-4b13-a42c-abb8dfec075c

Population	Parent...	#Events	%Parent	%Gran...	%Total	488 5... Mean	488 5... Median	PE-A Mean	PE-A Median	APC-A Mean	APC-A Median
All Events	####	####	####	####	####	39	25	567	111	44	10
P2	####	####	####	####	####	36	25	554	111	34	10
P8	P2	7,734	77.4	77.3	77.3	24	20	322	70	10	0
Q1	P8	5	0.1	0.1	0.0	15	13	74	66	937	782
Q2	P8	8	0.1	0.1	0.1	96	47	1,937	596	625	652
Q3	P8	4,056	52.4	40.6	40.5	15	13	45	17	-2	-7
Q4	P8	3,665	47.4	36.7	36.6	35	29	627	334	21	10
P7	P8	5,417	70.0	54.2	54.1	29	24	446	132	17	6
P1	P8	2,674	34.6	26.7	26.7	28	23	412	98	118	97
P4	P8	2,485	32.1	24.9	24.8	54	47	711	314	25	13

FACSDiva Version 6.1.3

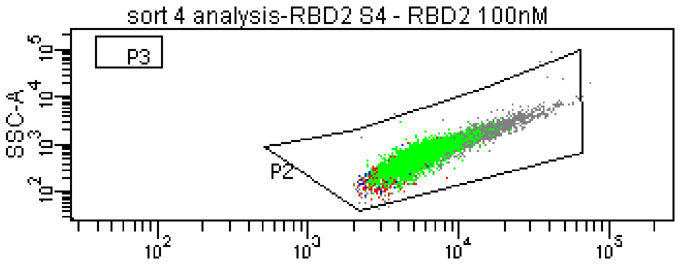

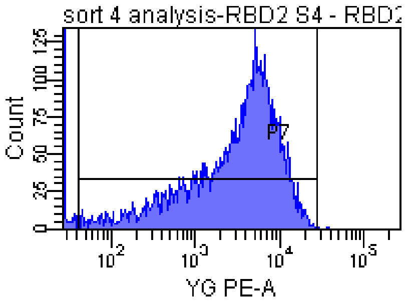

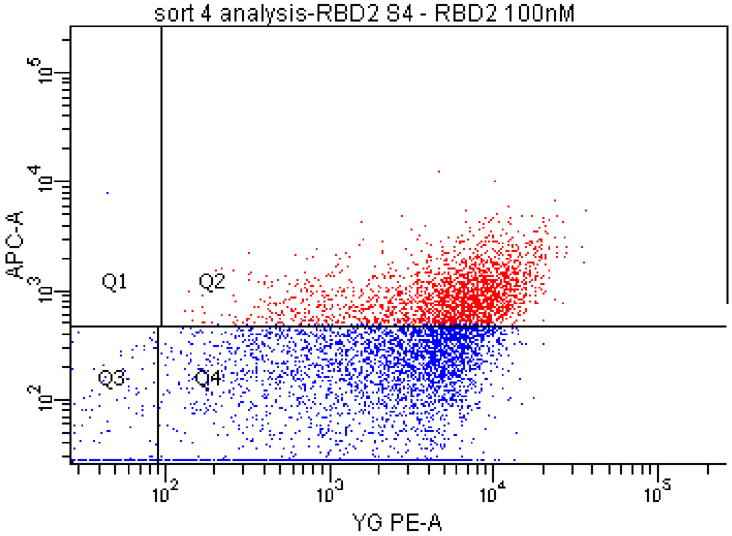

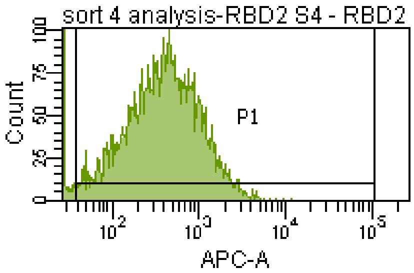

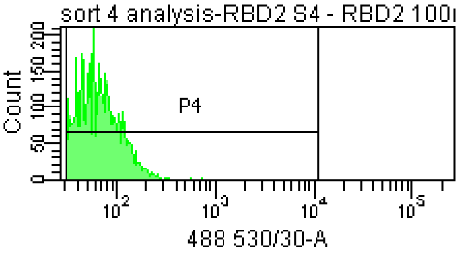

Experiment Name: Bio RBD2 selectons
Specimen Name: sort 4 analysis
Tube Name: RBD2 S4 - RBD2 100nM
Record Date: Jun 9, 2020 2:42:06 PM
$OP: Nileena
GUID: b20e5604-85a3-4092-9692-15bdb9883cc5

Population	Parent...	#Events	%Parent	%Gran...	%Total	488 5... Mean	488 5... Median	PE-A Mean	PE-A Median	APC-A Mean	APC-A Median
All Events	####	####	####	####	####	86	58	2,494	1,745	1,024	409
P2	####	####	####	####	####	84	58	2,469	1,745	966	409
P8	P2	7,126	71.3	71.2	71.2	51	43	1,535	1,184	434	261
Q1	P8	1	0.0	0.0	0.0	39	39	173	173	7,769	7,769
Q2	P8	2,249	31.6	22.5	22.5	82	75	2,712	2,510	1,064	839
Q3	P8	1,094	15.4	10.9	10.9	17	15	54	36	-7	-14
Q4	P8	3,782	53.1	37.8	37.8	42	39	1,264	1,082	184	181
P7	P8	6,151	86.3	61.5	61.4	56	48	1,755	1,485	500	328
P1	P8	5,614	78.8	56.1	56.1	58	49	1,821	1,564	564	376
P4	P8	4,479	62.9	44.8	44.7	74	63	2,176	1,955	584	406

FACSDiva Version 6.1.3

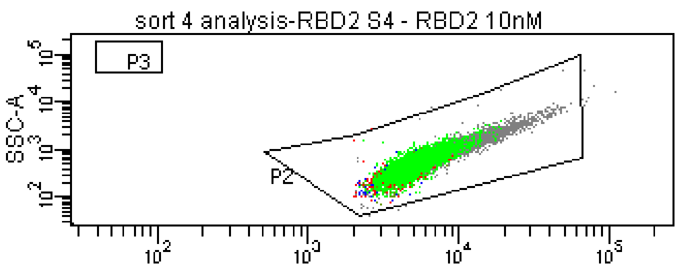

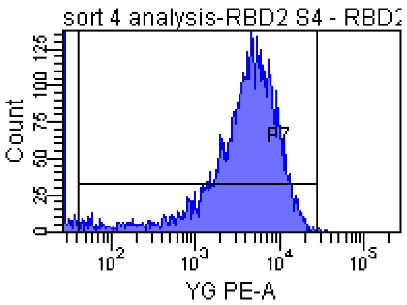

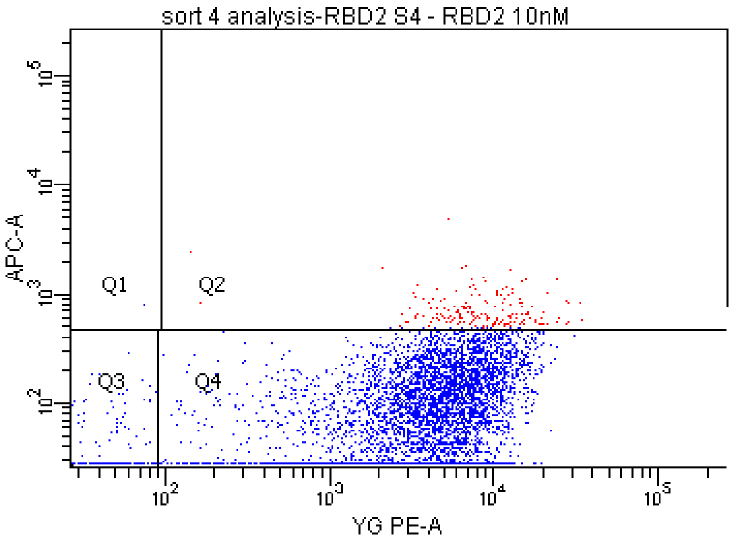

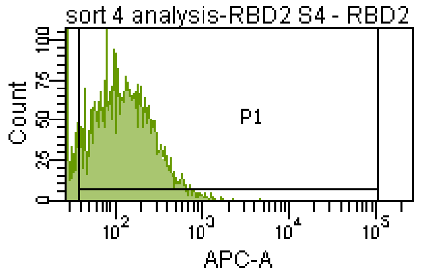

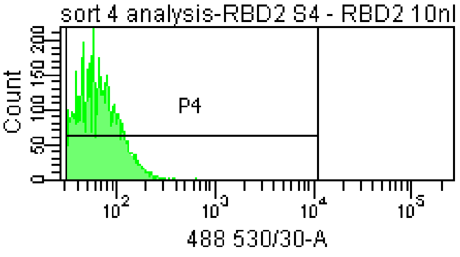

Experiment Name: Bio RBD2 selectons
Specimen Name: sort 4 analysis
Tube Name: RBD2 S4 - RBD2 10nM
Record Date: Jun 9, 2020 2:43:48 PM
$OP: Nileena
GUID: d35e9db7-574f-49b4-bc58-834108f1d3e4

Population	Parent...	#Events	%Parent	%Gran...	%Total	488 5... Mean	488 5... Median	PE-A Mean	PE-A Median	APC-A Mean	APC-A Median
All Events	####	####	####	####	####	89	62	2,551	1,858	278	95
P2	####	####	####	####	####	86	62	2,500	1,858	263	95
P8	P2	6,886	68.9	68.8	68.8	54	47	1,665	1,434	91	57
Q1	P8	2	0.0	0.0	0.0	49	49	184	184	678	678
Q2	P8	172	2.5	1.7	1.7	121	104	3,739	3,181	742	617
Q3	P8	1,016	14.8	10.2	10.1	17	15	53	37	3	-3
Q4	P8	5,696	82.7	57.0	56.9	58	51	1,891	1,650	86	66
P7	P8	5,959	86.5	59.6	59.5	59	52	1,905	1,655	104	68
P1	P8	3,957	57.5	39.6	39.5	66	59	2,118	1,858	171	121
P4	P8	4,668	67.8	46.7	46.6	73	63	2,179	1,954	119	78

FACSDiva Version 6.1.3

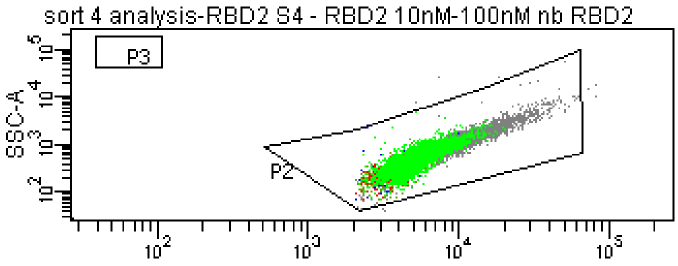

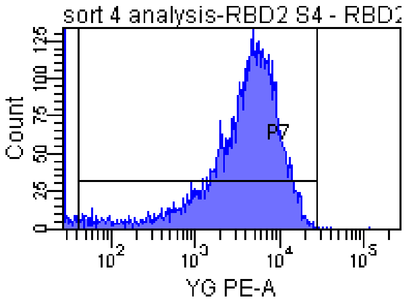

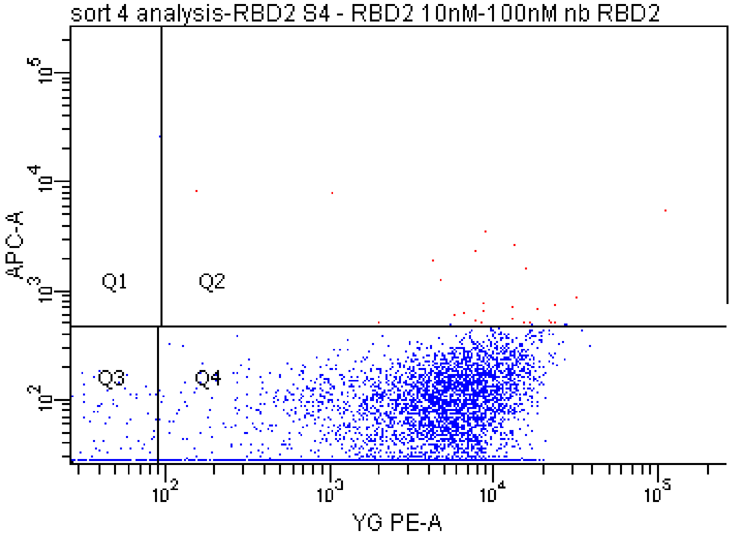

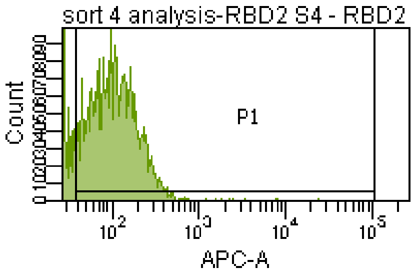

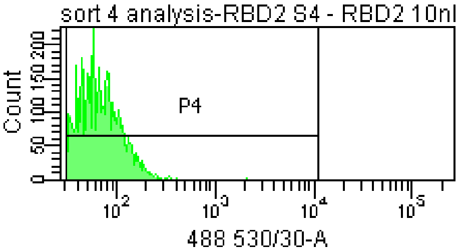

Experiment Name: Bio RBD2 selectons
Specimen Name: sort 4 analysis
Tube Name: RBD2 S4 - RBD2 10nM-100nM nb RBD2
Record Date: Jun 9, 2020 2:44:53 PM
$OP: Nileena
GUID: 279f759f-f441-4c11-a5ef-f04a64c22554

Population	Parent...	#Events	%Parent	%Gran...	%Total	488 5... Mean	488 5... Median	PE-A Mean	PE-A Median	APC-A Mean	APC-A Median
All Events	####	####	####	####	####	90	62	2,582	1,886	246	58
P2	####	####	####	####	####	89	62	2,566	1,886	182	58
P8	P2	7,079	70.8	70.7	70.7	54	47	1,689	1,428	56	32
Q1	P8	4	0.1	0.0	0.0	25	22	84	66	10,405	7,915
Q2	P8	26	0.4	0.3	0.3	229	148	6,167	4,274	1,667	660
Q3	P8	1,064	15.0	10.6	10.6	16	14	45	32	-1	-6
Q4	P8	5,985	84.5	59.8	59.8	60	53	1,963	1,709	52	41
P7	P8	6,117	86.4	61.2	61.1	60	52	1,928	1,680	61	41
P1	P8	3,377	47.7	33.8	33.7	68	60	2,191	1,930	144	102
P4	P8	4,722	66.7	47.2	47.2	75	64	2,264	2,039	72	49

FACSDiva Version 6.1.3

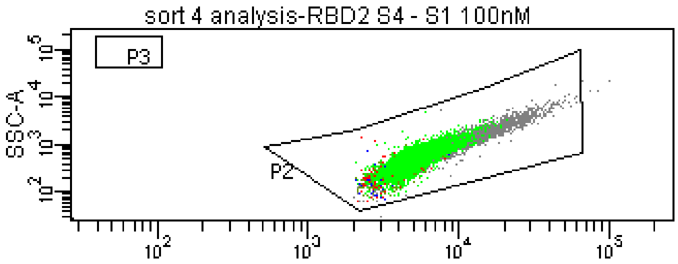

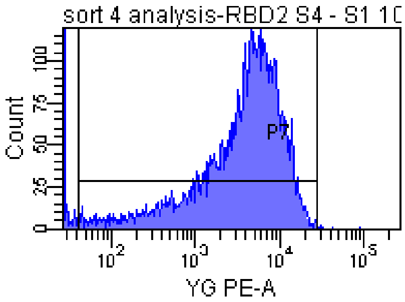

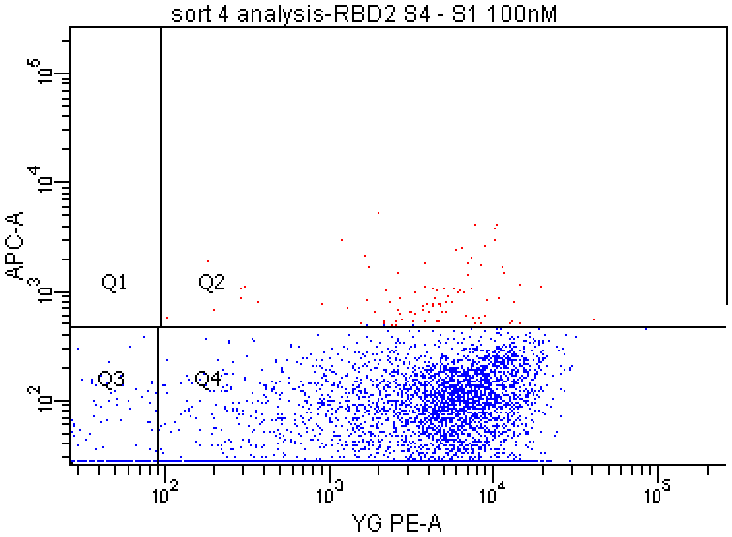

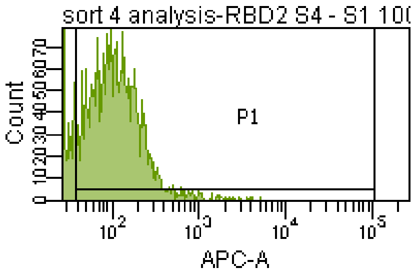

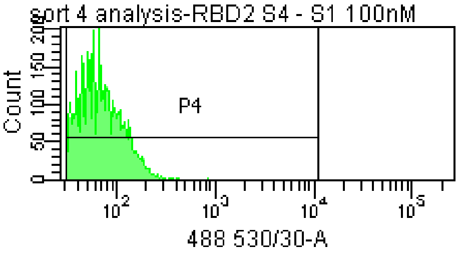

Experiment Name: Bio RBD2 selectons
Specimen Name: sort 4 analysis
Tube Name: RBD2 S4 - S1 100nM
Record Date: Jun 9, 2020 2:46:35 PM
$OP: Nileena
GUID: e55e2cf4-3ece-4ec3-8163-aa0efed7de0e

Population	Parent...	#Events	%Parent	%Gran...	%Total	488 5... Mean	488 5... Median	PE-A Mean	PE-A Median	APC-A Mean	APC-A Median
All Events	####	####	####	####	####	93	63	2,800	1,951	167	50
P2	####	####	####	####	####	91	63	2,766	1,951	141	50
P8	P2	7,011	70.1	70.1	70.1	55	47	1,726	1,426	50	27
Q1	P8	1	0.0	0.0	0.0	-15	-15	15	15	481	481
Q2	P8	89	1.3	0.9	0.9	75	57	2,141	1,635	1,092	757
Q3	P8	1,048	14.9	10.5	10.5	15	13	47	34	6	-3
Q4	P8	5,873	83.8	58.7	58.7	62	53	2,020	1,724	42	31
P7	P8	6,059	86.4	60.6	60.5	61	52	1,968	1,691	56	32
P1	P8	3,165	45.1	31.6	31.6	67	57	2,157	1,853	148	102
P4	P8	4,644	66.2	46.4	46.4	77	65	2,363	2,103	64	39

FACSDiva Version 6.1.3

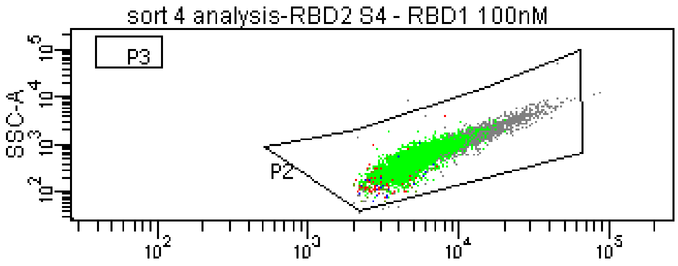

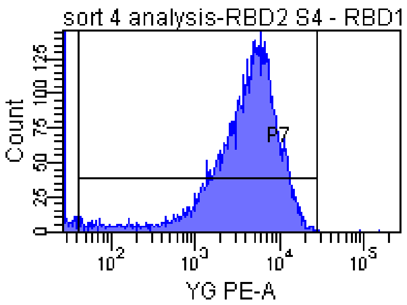

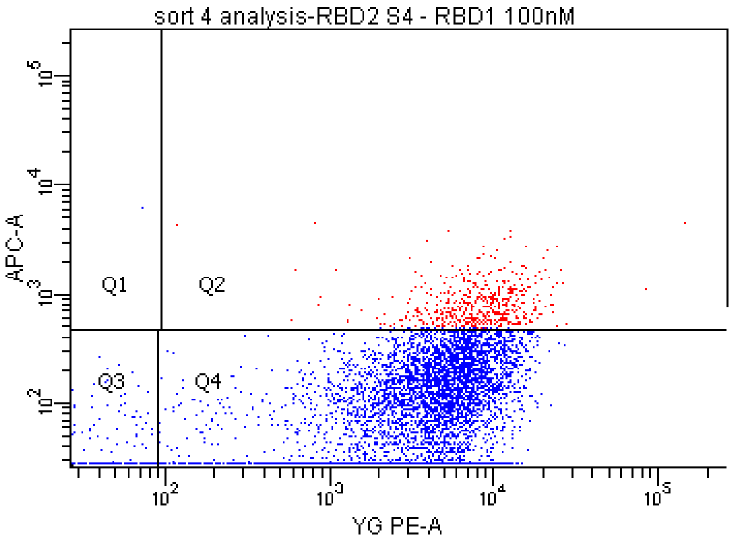

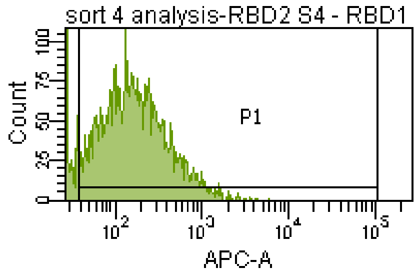

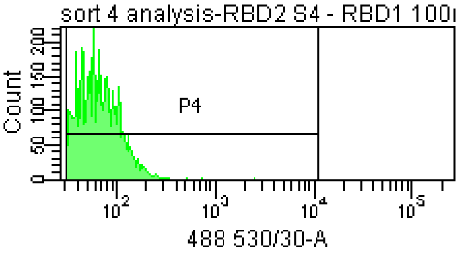

Experiment Name:	Bio RBD2 selectons
Specimen Name:	sort 4 analysis
Tube Name:	RBD2 S4 - RBD1 100nM
Record Date:	Jun 9, 2020 2:47:55 PM
$OP:	Nileena
GUID:	4f8ec606-3bd4-4766-ab64-0f3f1cb996dc

Population	Parent...	#Events	%Parent	%Gran...	%Total	488 5... Mean	488 5... Median	PE-A Mean	PE-A Median	APC-A Mean	APC-A Median
All Events	####	####	####	####	####	81	59	2,414	1,850	352	118
P2	####	####	####	####	####	81	59	2,413	1,850	350	118
P8	P2	7,341	73.4	73.3	73.3	53	46	1,665	1,432	144	73
Q1	P8	3	0.0	0.0	0.0	7	3	204	88	3,706	4,434
Q2	P8	512	7.0	5.1	5.1	111	96	3,649	3,160	878	710
Q3	P8	1,110	15.1	11.1	11.1	17	15	51	35	-5	-12
Q4	P8	5,716	77.9	57.2	57.1	55	49	1,802	1,605	105	83
P7	P8	6,329	86.2	63.3	63.2	59	52	1,900	1,671	166	94
P1	P8	4,480	61.0	44.8	44.7	66	58	2,127	1,893	249	153
P4	P8	4,913	66.9	49.1	49.1	73	63	2,198	1,993	194	110

FACSDiva Version 6.1.3

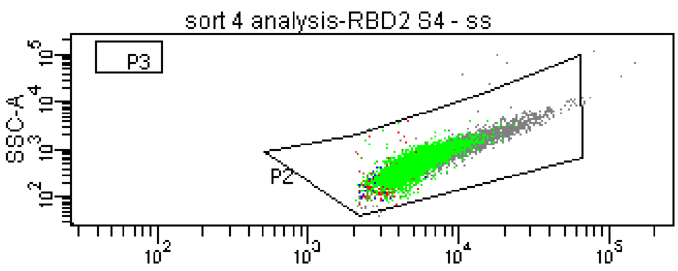

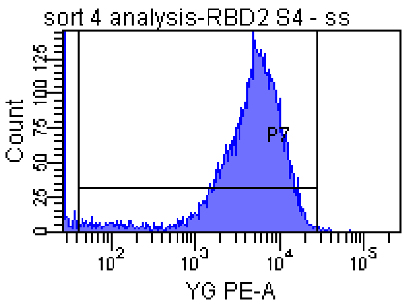

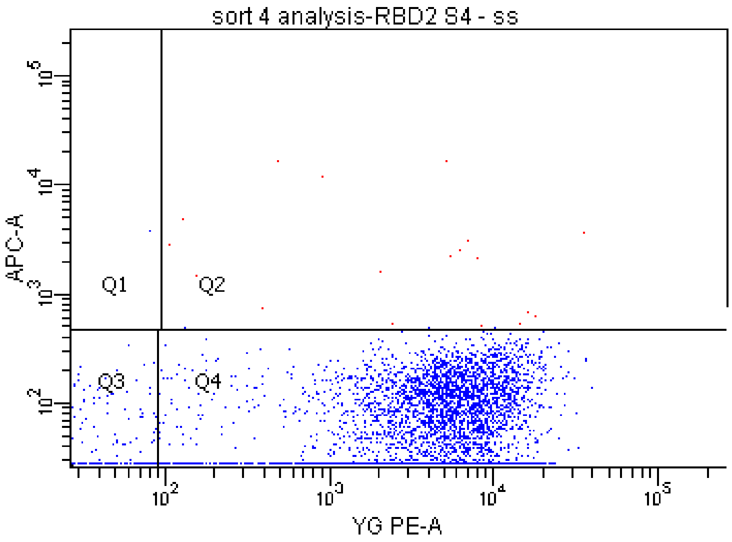

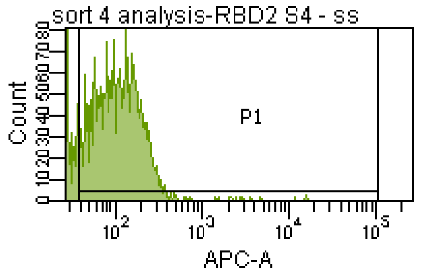

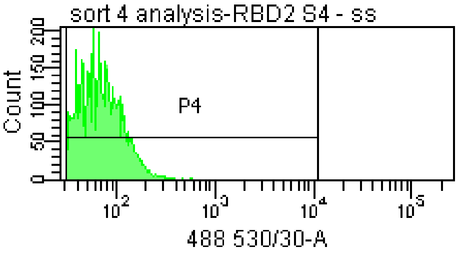

Experiment Name: Bio RBD2 selectons
Specimen Name: sort 4 analysis
Tube Name: RBD2 S4 - ss
Record Date: Jun 9, 2020 2:49:01 PM
$OP: Nileena
GUID: 53b248ce-6b92-44fa-84e0-63fb436de9b0

Population	Parent...	#Events	%Parent	%Gran...	%Total	488 5... Mean	488 5... Median	PE-A Mean	PE-A Median	APC-A Mean	APC-A Median
All Events	####	####	####	####	####	124	66	2,962	2,164	175	35
P2	####	####	####	####	####	98	66	2,882	2,162	99	35
P8	P2	6,895	69.0	68.8	68.8	58	50	1,905	1,676	35	14
Q1	P8	5	0.1	0.0	0.0	38	20	227	119	1,770	927
Q2	P8	18	0.3	0.2	0.2	132	77	2,845	2,258	3,897	2,066
Q3	P8	945	13.7	9.4	9.4	15	14	47	37	-5	-12
Q4	P8	5,927	86.0	59.3	59.2	65	57	2,200	1,916	28	19
P7	P8	6,040	87.6	60.4	60.3	64	56	2,141	1,887	38	19
P1	P8	2,856	41.4	28.6	28.5	67	58	2,229	1,925	149	106
P4	P8	4,878	70.7	48.8	48.7	77	66	2,442	2,191	42	24

FACSDiva Version 6.1.3

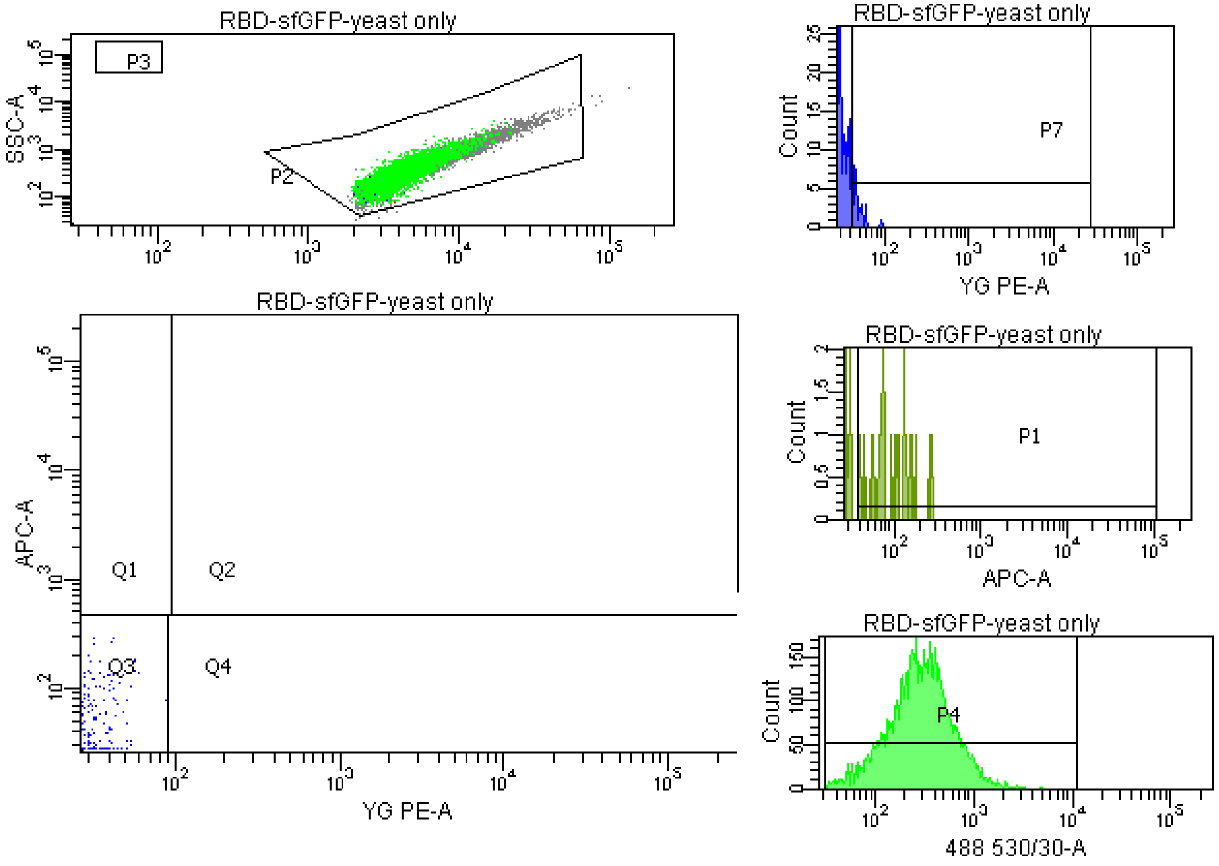

Experiment Name: Bio RBD2 selectons
Specimen Name: RBD-sfGFP
Tube Name: yeast only
Record Date: Jun 17, 2020 5:07:56 PM
$OP: Nileena
GUID: 7175fe12-902d-4541-a07d-1b8f1bb274f7

Population	Parent...	#Events	%Parent	%Gran...	%Total	488 5... Mean	488 5... Median	PE-A Mean	PE-A Median	APC-A Mean	APC-A Median
All Events	####	####	####	####	####	592	351	92	31	31	20
P2	####	####	####	####	####	584	351	88	31	31	20
P8	P2	7,203	72.0	71.9	71.9	356	278	40	19	20	14
Q1	P8	0	0.0	0.0	0.0	####	####	####	####	####	####
Q2	P8	0	0.0	0.0	0.0	####	####	####	####	####	####
Q3	P8	7,203	100.0	72.0	71.9	356	278	40	19	20	14
Q4	P8	0	0.0	0.0	0.0	####	####	####	####	####	####
P7	P8	35	0.5	0.4	0.3	809	431	150	92	80	68
P1	P8	2,171	30.1	21.7	21.7	441	335	54	27	78	68
P4	P8	7,028	97.6	70.3	70.1	365	284	41	19	21	14

FACSDiva Version 6.1.3

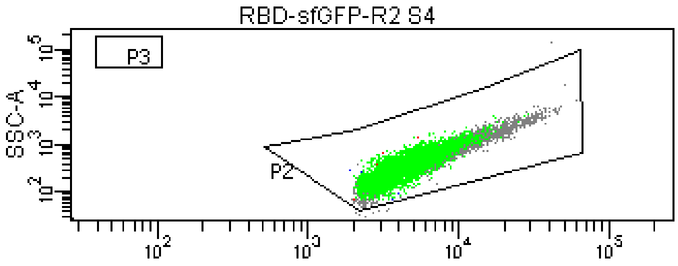

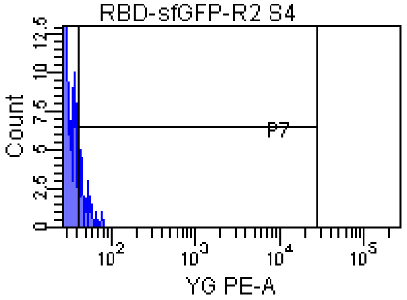

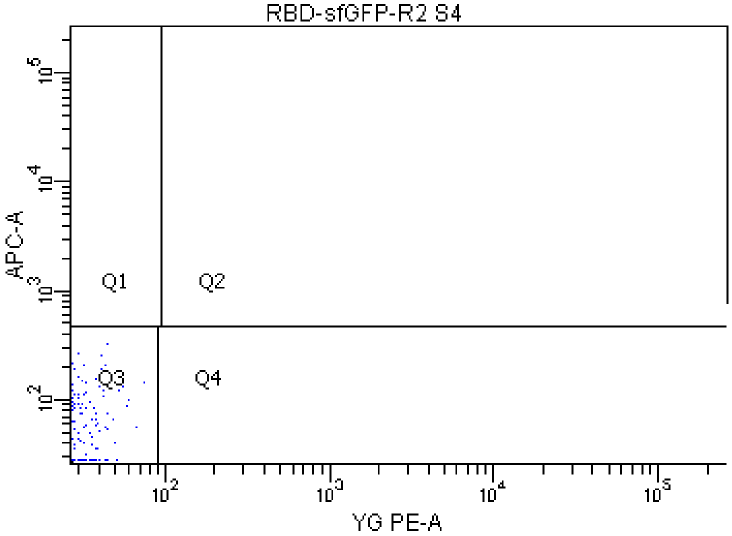

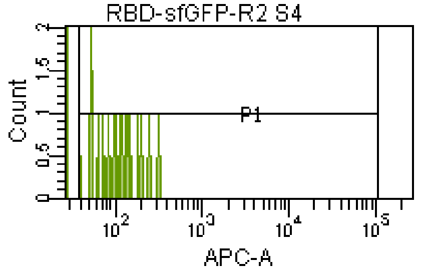

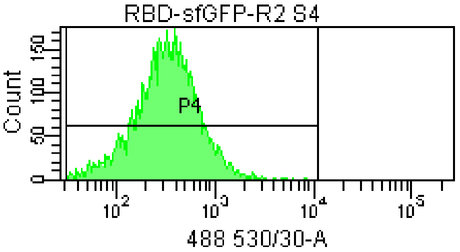

Experiment Name: Bio RBD2 selectons
Specimen Name: RBD-sfGFP
Tube Name: R2 S4
Record Date: Jun 17, 2020 5:08:58 PM
$OP: Nileena
GUID: cc9d72a5-d9ee-4e03-90a3-06976473a890

Population	Parent...	#Events	%Parent	%Gran...	%Total	488 5... Mean	488 5... Median	PE-A Mean	PE-A Median	APC-A Mean	APC-A Median
All Events	####	####	####	####	####	620	393	82	43	29	17
P2	####	####	####	####	####	617	393	74	43	29	17
P8	P2	7,355	73.6	73.4	73.4	415	319	40	32	20	12
Q1	P8	0	0.0	0.0	0.0	####	####	####	####	####	####
Q2	P8	0	0.0	0.0	0.0	####	####	####	####	####	####
Q3	P8	7,355	100.0	73.6	73.4	415	319	40	32	20	12
Q4	P8	0	0.0	0.0	0.0	####	####	####	####	####	####
P7	P8	22	0.3	0.2	0.2	863	579	151	90	101	88
P1	P8	2,189	29.8	21.9	21.9	533	383	53	41	78	67
P4	P8	7,229	98.3	72.3	72.2	413	324	41	33	20	12

FACSDiva Version 6.1.3

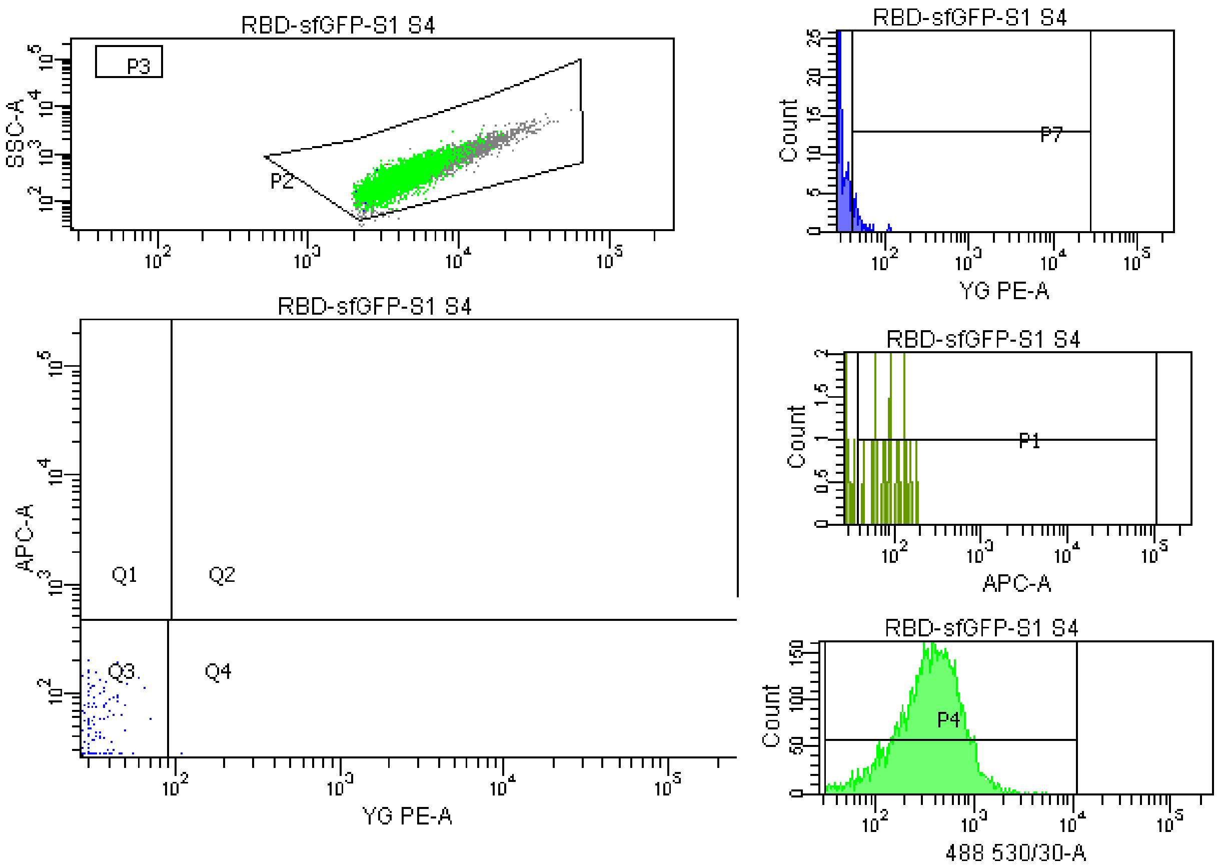

Experiment Name: Bio RBD2 selectons
Specimen Name: RBD-sfGFP
Tube Name: S1 S4
Record Date: Jun 17, 2020 5:10:50 PM
$OP: Nileena
GUID: 7d572c49-c05f-4dad-9ded-bf7d229e8350

Population	Parent...	#Events	%Parent	%Gran...	%Total	488 5... Mean	488 5... Median	PE-A Mean	PE-A Median	APC-A Mean	APC-A Median
All Events	####	####	####	####	####	609	420	63	32	23	13
P2	####	####	####	####	####	610	421	63	32	23	13
P8	P2	7,536	75.4	75.2	75.2	423	353	36	23	16	9
Q1	P8	1	0.0	0.0	0.0	861	861	17	17	551	551
Q2	P8	0	0.0	0.0	0.0	####	####	####	####	####	####
Q3	P8	7,534	100.0	75.3	75.2	422	353	36	23	16	9
Q4	P8	1	0.0	0.0	0.0	1,395	1,395	433	433	7	7
P7	P8	22	0.3	0.2	0.2	712	529	128	113	71	67
P1	P8	2,018	26.8	20.2	20.1	518	422	48	29	75	66
P4	P8	7,270	96.5	72.7	72.5	437	364	37	24	16	10

FACSDiva Version 6.1.3

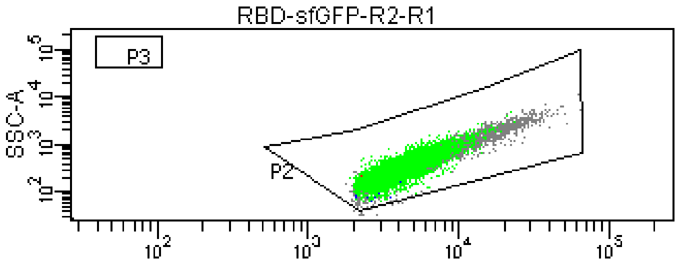

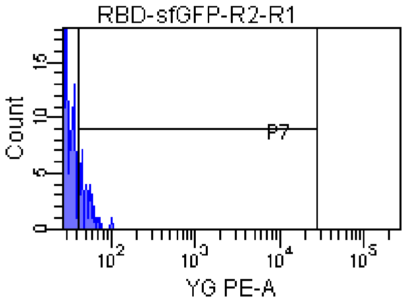

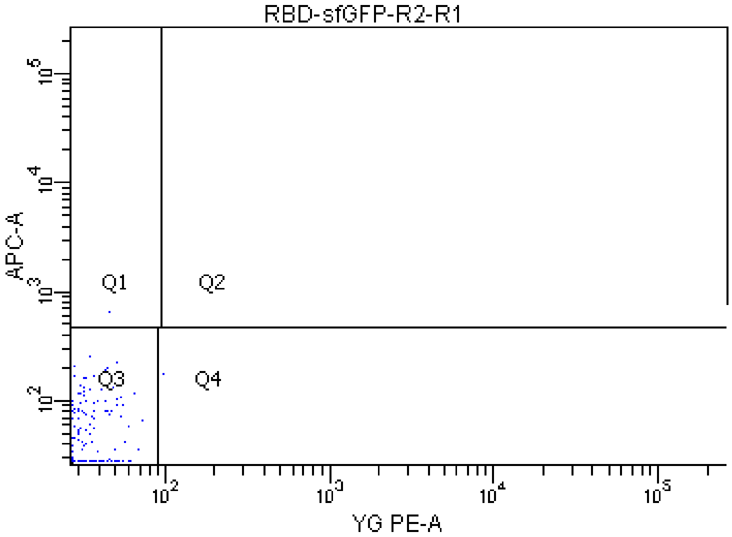

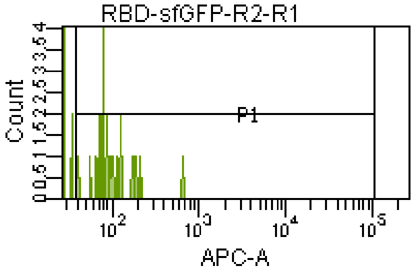

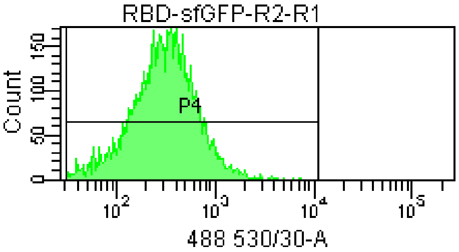

Experiment Name: Bio RBD2 selectons
Specimen Name: RBD-sfGFP
Tube Name: R2-R1
Record Date: Jun 17, 2020 5:12:15 PM
$OP: Nileena
GUID: 92fcc99c-84b6-47d7-8b4d-d837f85048d6

Population	Parent...	#Events	%Parent	%Gran...	%Total	488 5... Mean	488 5... Median	PE-A Mean	PE-A Median	APC-A Mean	APC-A Median
All Events	####	####	####	####	####	588	346	66	27	28	16
P2	####	####	####	####	####	588	347	65	27	27	16
P8	P2	7,784	77.8	77.7	77.7	374	291	33	19	18	11
Q1	P8	1	0.0	0.0	0.0	631	631	62	62	631	631
Q2	P8	0	0.0	0.0	0.0	####	####	####	####	####	####
Q3	P8	7,782	100.0	77.8	77.6	374	291	33	19	18	11
Q4	P8	1	0.0	0.0	0.0	765	765	117	117	168	168
P7	P8	39	0.5	0.4	0.4	601	541	119	92	79	63
P1	P8	2,209	28.4	22.1	22.0	467	349	44	26	76	66
P4	P8	7,472	96.0	74.7	74.6	388	303	34	20	18	11

FACSDiva Version 6.1.3

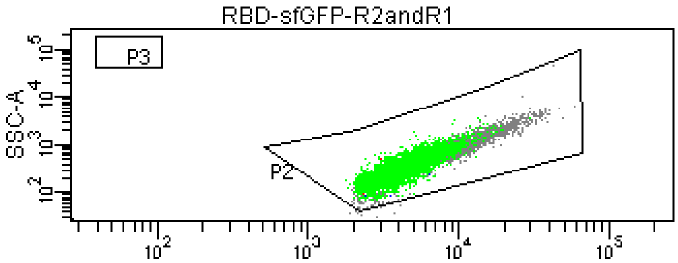

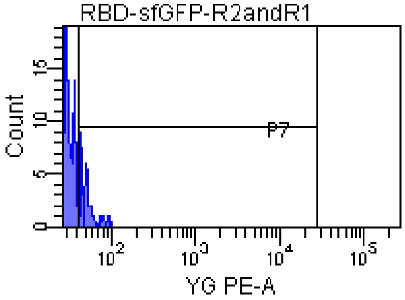

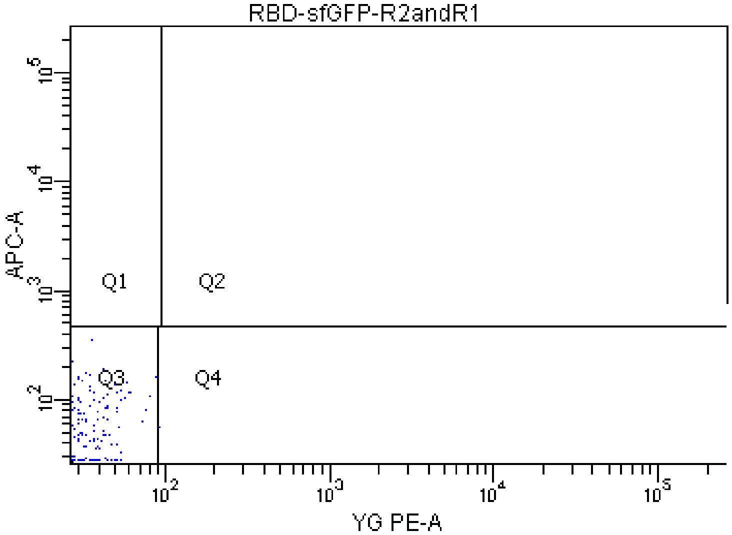

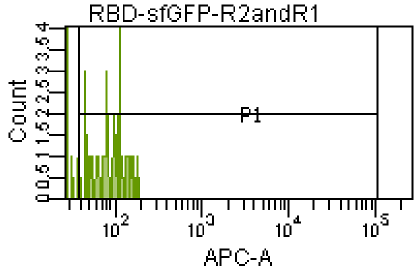

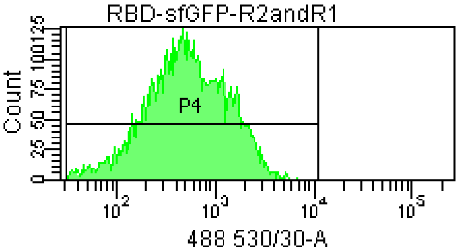

Experiment Name: Bio RBD2 selectons
Specimen Name: RBD-sfGFP
Tube Name: R2andR1
Record Date: Jun 17, 2020 5:13:24 PM
$OP: Nileena
GUID: 80df26b3-0bac-4b5e-803f-9c136148d228

Population	Parent...	#Events	%Parent	%Gran...	%Total	488 5... Mean	488 5... Median	PE-A Mean	PE-A Median	APC-A Mean	APC-A Median
All Events	####	####	####	####	####	1,069	625	76	32	29	17
P2	####	####	####	####	####	1,070	626	76	32	29	17
P8	P2	7,196	72.0	71.8	71.8	725	486	38	21	19	12
Q1	P8	1	0.0	0.0	0.0	1,479	1,479	166	166	558	558
Q2	P8	0	0.0	0.0	0.0	####	####	####	####	####	####
Q3	P8	7,194	100.0	71.9	71.8	725	486	38	21	19	12
Q4	P8	1	0.0	0.0	0.0	2,024	2,024	112	112	52	52
P7	P8	42	0.6	0.4	0.4	1,128	931	110	86	75	73
P1	P8	2,108	29.3	21.1	21.0	841	572	51	31	77	66
P4	P8	6,972	96.9	69.7	69.6	747	507	39	22	19	12

FACSDiva Version 6.1.3

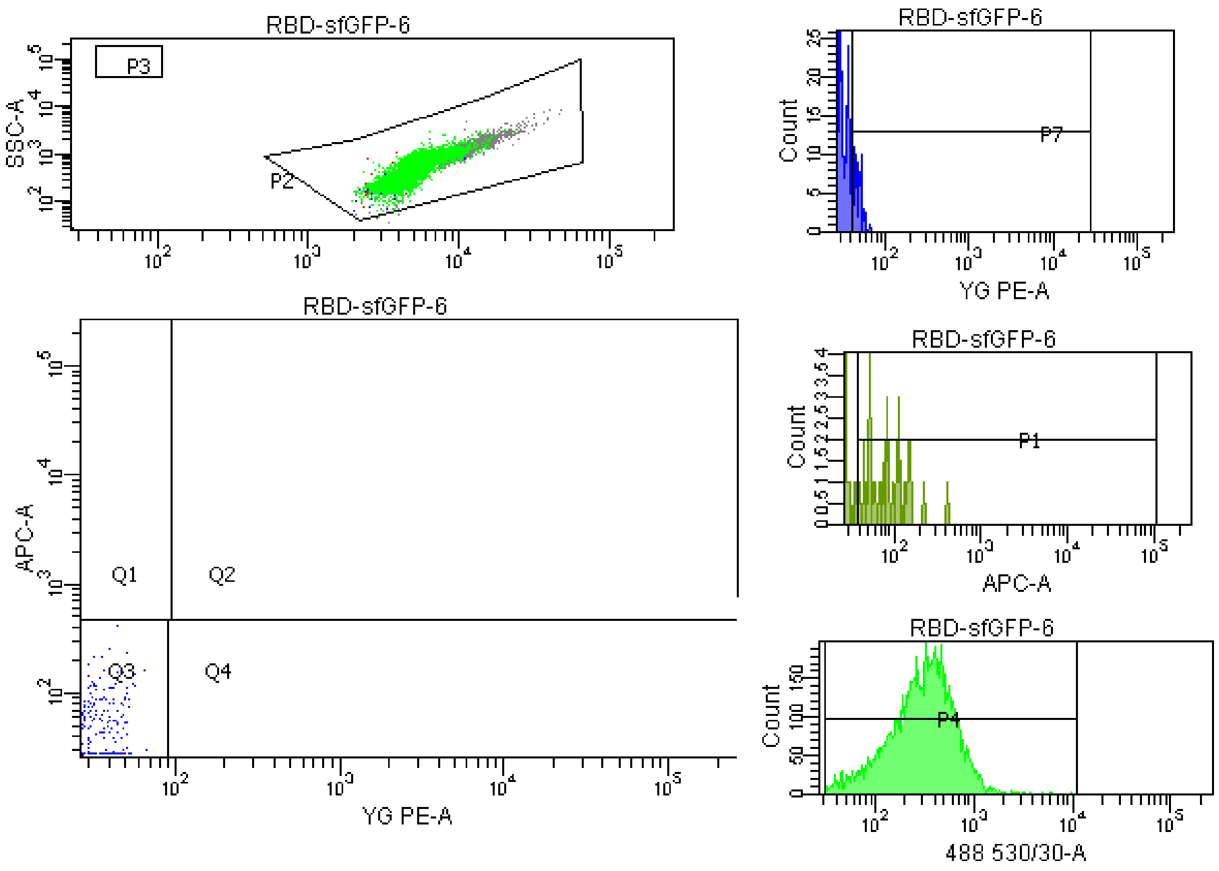

Experiment Name: Bio RBD2 selectons
Specimen Name: RBD-sfGFP
Tube Name: 6
Record Date: Jun 17, 2020 5:14:33 PM
$OP: Nileena
GUID: ebc0c8f2-ccec-424e-856d-763626527e29

Population	Parent...	#Events	%Parent	%Gran...	%Total	488 5... Mean	488 5... Median	PE-A Mean	PE-A Median	APC-A Mean	APC-A Median
All Events	####	####	####	####	####	470	322	67	41	21	12
P2	####	####	####	####	####	470	322	67	41	21	12
P8	P2	8,785	87.8	87.8	87.8	357	283	48	35	16	10
Q1	P8	0	0.0	0.0	0.0	####	####	####	####	####	####
Q2	P8	0	0.0	0.0	0.0	####	####	####	####	####	####
Q3	P8	8,785	100.0	87.8	87.8	357	283	48	35	16	10
Q4	P8	0	0.0	0.0	0.0	####	####	####	####	####	####
P7	P8	58	0.7	0.6	0.6	547	414	116	99	69	52
P1	P8	2,443	27.8	24.4	24.4	464	327	57	43	76	65
P4	P8	8,045	91.6	80.4	80.4	368	311	50	37	17	10

FACSDiva Version 6.1.3

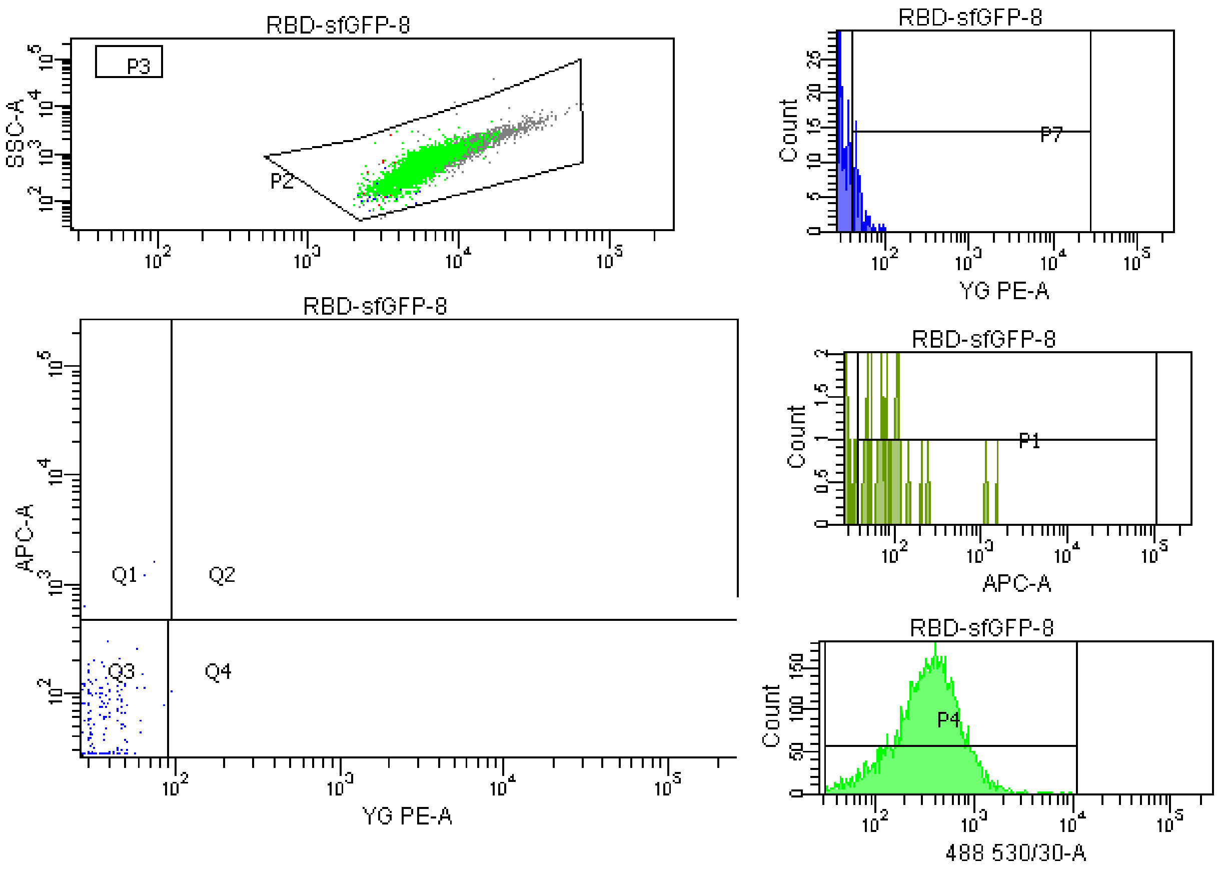

Experiment Name: Bio RBD2 selectons
Specimen Name: RBD-sfGFP
Tube Name: 8
Record Date: Jun 17, 2020 5:15:44 PM
$OP: Nileena
GUID: f446d5b6-9173-44b8-af8e-222a3b9f6264

Population	Parent...	#Events	%Parent	%Gran...	%Total	488 5... Mean	488 5... Median	PE-A Mean	PE-A Median	APC-A Mean	APC-A Median
All Events	####	####	####	####	####	660	402	101	60	42	19
P2	####	####	####	####	####	657	402	99	60	30	19
P8	P2	7,959	79.6	79.5	79.5	437	330	58	45	20	13
Q1	P8	3	0.0	0.0	0.0	3,602	3,009	468	516	1,101	1,163
Q2	P8	0	0.0	0.0	0.0	####	####	####	####	####	####
Q3	P8	7,955	99.9	79.6	79.5	436	330	58	45	20	13
Q4	P8	1	0.0	0.0	0.0	424	424	244	244	97	97
P7	P8	50	0.6	0.5	0.5	743	491	154	117	115	58
P1	P8	2,458	30.9	24.6	24.6	581	392	73	57	79	68
P4	P8	7,517	94.4	75.2	75.1	435	348	60	47	21	14

FACSDiva Version 6.1.3

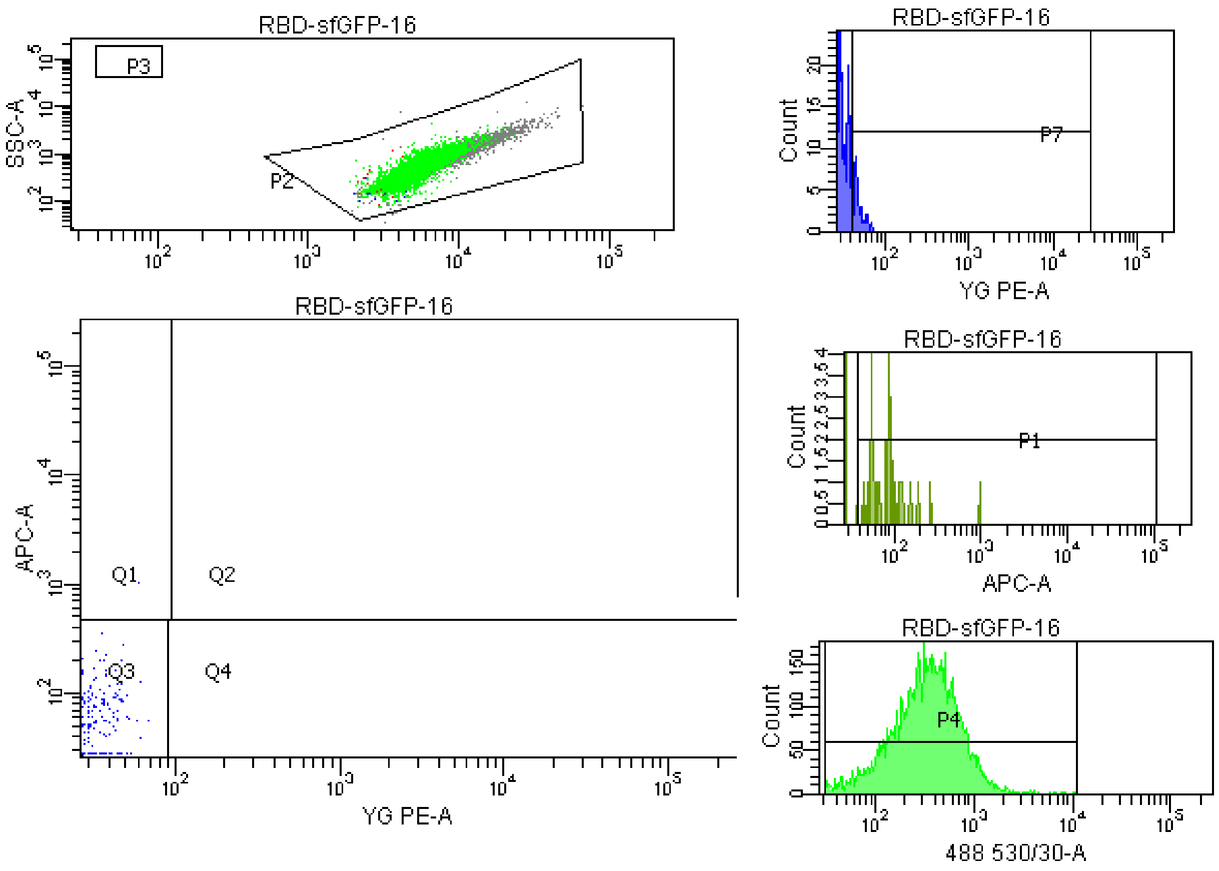

Experiment Name: Bio RBD2 selectons
Specimen Name: RBD-sfGFP
Tube Name: 16
Record Date: Jun 17, 2020 5:16:58 PM
$OP: Nileena
GUID: 6fdf7e74-a6d4-4bcf-b958-730ac32e6916

Population	Parent...	#Events	%Parent	%Gran...	%Total	488 5... Mean	488 5... Median	PE-A Mean	PE-A Median	APC-A Mean	APC-A Median
All Events	####	####	####	####	####	655	385	89	58	26	16
P2	####	####	####	####	####	654	385	89	58	26	16
P8	P2	7,977	79.8	79.7	79.7	444	314	56	46	18	11
Q1	P8	1	0.0	0.0	0.0	3,135	3,135	505	505	966	966
Q2	P8	0	0.0	0.0	0.0	####	####	####	####	####	####
Q3	P8	7,976	100.0	79.8	79.7	444	314	56	46	18	11
Q4	P8	0	0.0	0.0	0.0	####	####	####	####	####	####
P7	P8	40	0.5	0.4	0.4	639	439	159	132	88	59
P1	P8	2,366	29.7	23.7	23.6	608	371	71	56	77	66
P4	P8	7,516	94.2	75.2	75.1	427	332	58	47	19	12

FACSDiva Version 6.1.3

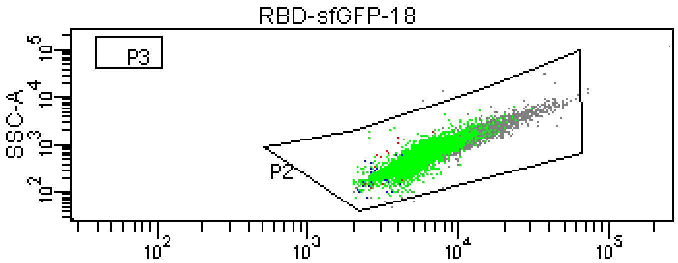

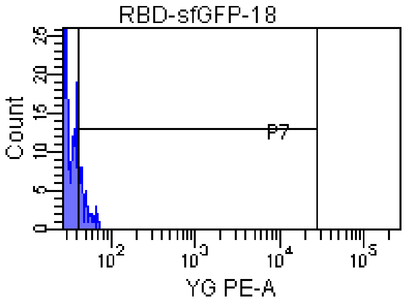

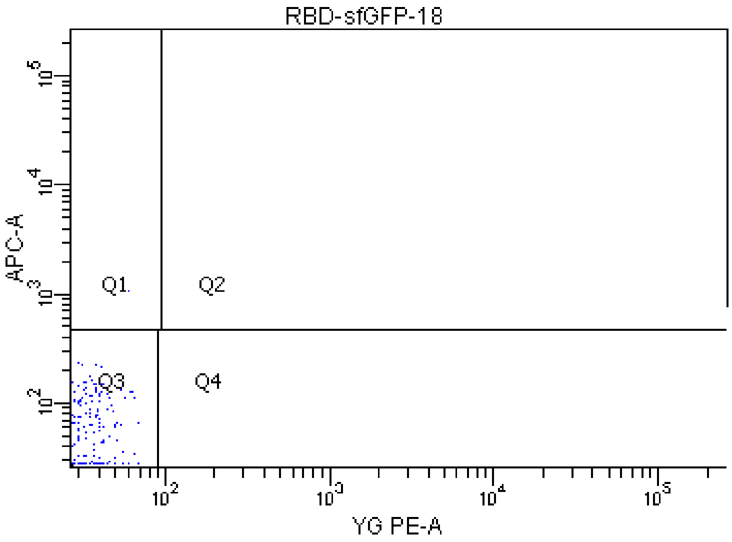

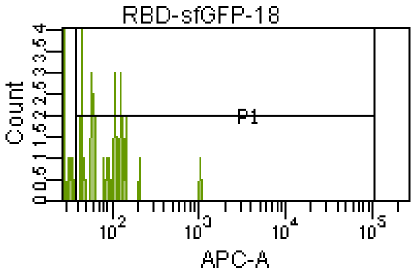

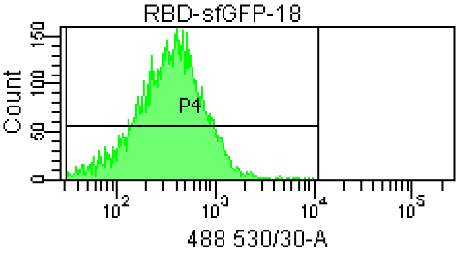

Experiment Name: Bio RBD2 selectons
Specimen Name: RBD-sfGFP
Tube Name: 18
Record Date: Jun 17, 2020 5:18:07 PM
$OP: Nileena
GUID: 3dd467e6-054c-426d-94e8-659f7389e110

Population	Parent...	#Events	%Parent	%Gran...	%Total	488 5... Mean	488 5... Median	PE-A Mean	PE-A Median	APC-A Mean	APC-A Median
All Events	####	####	####	####	####	862	446	140	66	43	23
P2	####	####	####	####	####	832	446	128	65	39	23
P8	P2	7,379	73.8	73.7	73.7	521	336	60	46	21	14
Q1	P8	3	0.0	0.0	0.0	42,674	3,641	289	209	695	525
Q2	P8	0	0.0	0.0	0.0	####	####	####	####	####	####
Q3	P8	7,376	100.0	73.8	73.7	504	336	60	46	21	14
Q4	P8	0	0.0	0.0	0.0	####	####	####	####	####	####
P7	P8	43	0.6	0.4	0.4	642	435	141	126	86	58
P1	P8	2,311	31.3	23.1	23.1	793	411	78	60	80	68
P4	P8	6,927	93.9	69.3	69.2	456	356	62	48	22	14

FACSDiva Version 6.1.3

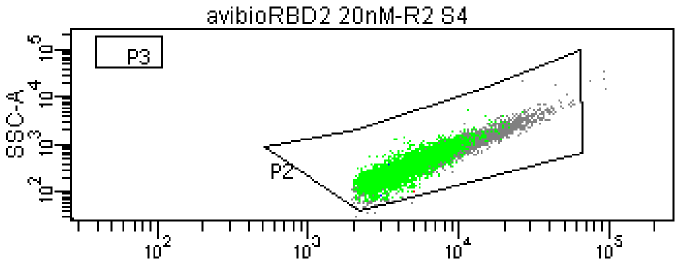

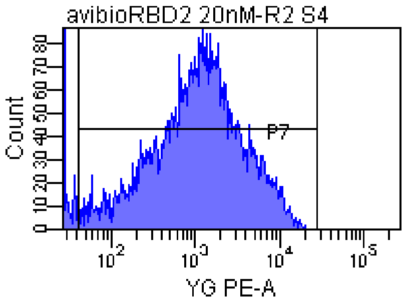

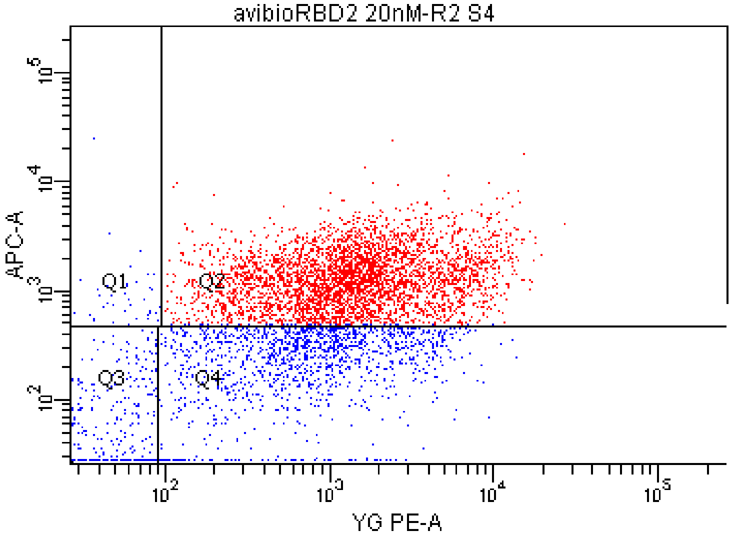

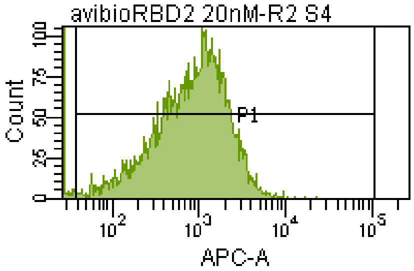

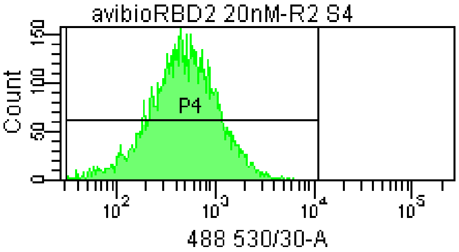

Experiment Name: Bio RBD2 selectons
Specimen Name: avibioRBD2 20nM
Tube Name: R2 S4
Record Date: Jun 17, 2020 5:55:46 PM
$OP: Nileena
GUID: 0c91a06d-47b9-48ee-94f9-8eb97cafb665

Population	Parent...	#Events	%Parent	%Gran...	%Total	488 5... Mean	488 5... Median	PE-A Mean	PE-A Median	APC-A Mean	APC-A Median
All Events	####	####	####	####	####	1,041	617	975	423	1,417	845
P2	####	####	####	####	####	1,026	617	956	423	1,395	845
P8	P2	7,057	70.6	70.4	70.4	614	467	568	288	886	585
Q1	P8	44	0.6	0.4	0.4	389	284	49	31	1,680	811
Q2	P8	3,842	54.4	38.4	38.3	789	625	856	517	1,496	1,214
Q3	P8	1,744	24.7	17.4	17.4	305	253	33	22	29	17
Q4	P8	1,427	20.2	14.3	14.2	531	455	462	289	264	276
P7	P8	5,465	77.4	54.6	54.5	705	559	723	424	1,128	874
P1	P8	5,843	82.8	58.4	58.3	683	537	677	387	1,071	800
P4	P8	6,982	98.9	69.8	69.7	621	472	574	294	893	596

FACSDiva Version 6.1.3

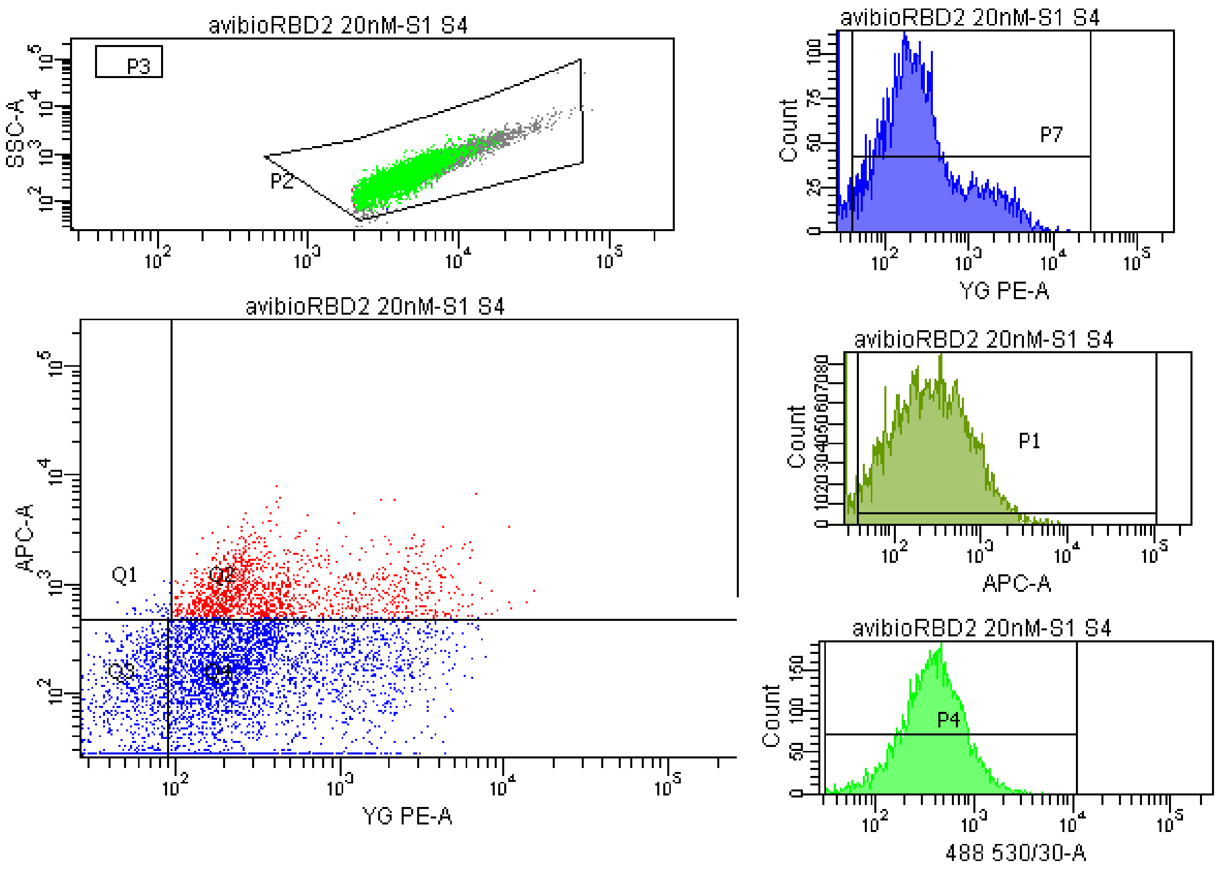

Experiment Name: Bio RBD2 selectons
Specimen Name: avibioRBD2 20nM
Tube Name: S1 S4
Record Date: Jun 17, 2020 5:56:38 PM
$OP: Nileena
GUID: da1778c9-e07a-435a-a262-30dcd349d50c

Population	Parent...	#Events	%Parent	%Gran...	%Total	488 5... Mean	488 5... Median	PE-A Mean	PE-A Median	APC-A Mean	APC-A Median
All Events	####	####	####	####	####	736	444	319	113	453	182
P2	####	####	####	####	####	693	445	279	113	419	183
P8	P2	7,607	76.1	75.9	75.9	465	377	190	88	299	145
Q1	P8	30	0.4	0.3	0.3	374	350	61	41	647	567
Q2	P8	1,504	19.8	15.0	15.0	669	534	355	165	979	782
Q3	P8	2,438	32.0	24.4	24.3	324	268	36	25	52	30
Q4	P8	3,635	47.8	36.4	36.3	475	405	226	122	180	158
P7	P8	5,895	77.5	59.0	58.8	508	419	237	118	378	220
P1	P8	5,772	75.9	57.7	57.6	512	420	229	112	395	228
P4	P8	7,503	98.6	75.0	74.9	471	382	192	90	302	147

FACSDiva Version 6.1.3

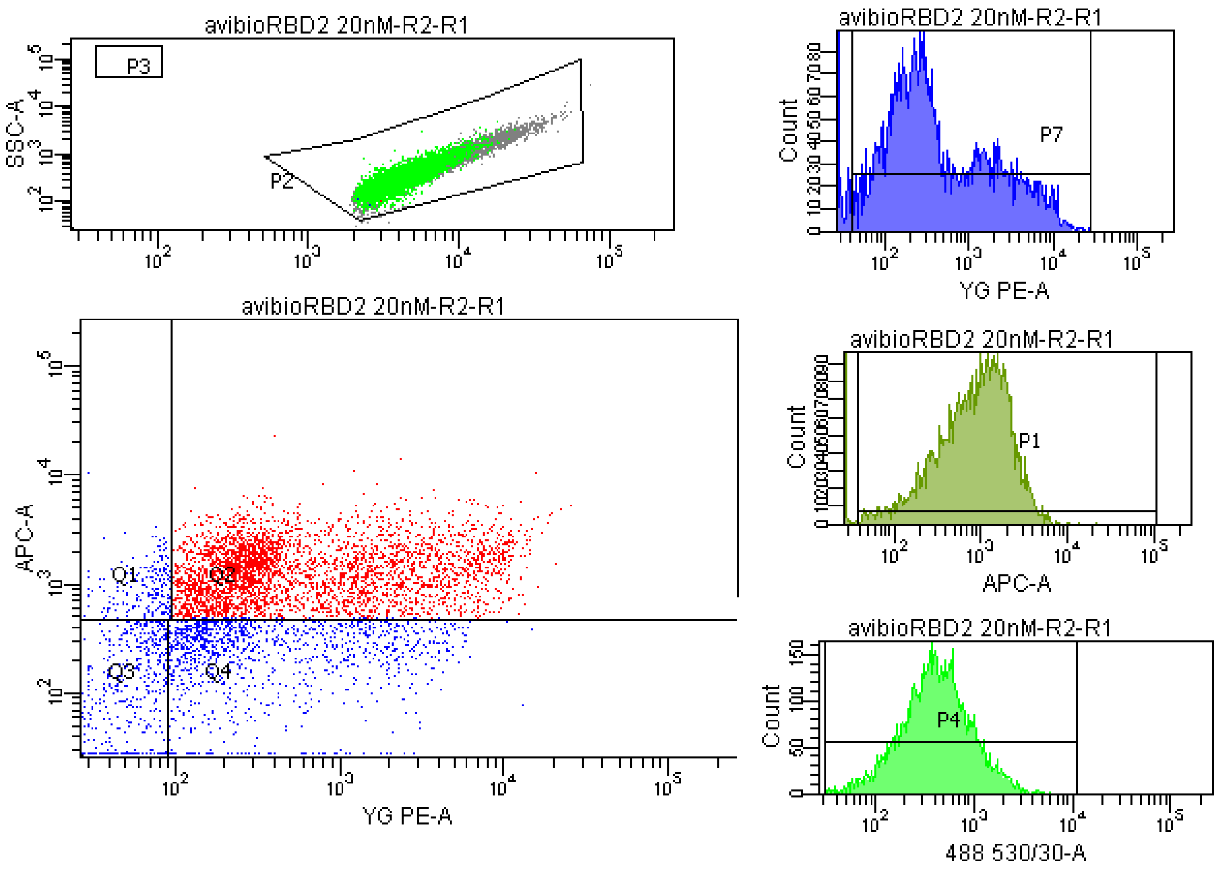

Experiment Name: Bio RBD2 selectons
Specimen Name: avibioRBD2 20nM
Tube Name: R2-R1
Record Date: Jun 17, 2020 5:57:43 PM
$OP: Nileena
GUID: 6740a90a-f27b-4a5e-985c-7631a86fb9ec

Population	Parent...	#Events	%Parent	%Gran...	%Total	488 5... Mean	488 5... Median	PE-A Mean	PE-A Median	APC-A Mean	APC-A Median
All Events	####	####	####	####	####	884	506	612	155	1,307	773
P2	####	####	####	####	####	876	506	605	155	1,291	774
P8	P2	7,350	73.5	73.4	73.4	546	403	383	108	859	562
Q1	P8	224	3.0	2.2	2.2	363	277	55	38	1,063	834
Q2	P8	3,760	51.2	37.6	37.5	712	540	616	206	1,500	1,253
Q3	P8	2,136	29.1	21.4	21.3	319	251	34	22	53	24
Q4	P8	1,230	16.7	12.3	12.3	466	396	334	138	263	272
P7	P8	5,517	75.1	55.2	55.1	626	473	500	164	1,127	883
P1	P8	6,041	82.2	60.4	60.3	604	453	458	142	1,047	784
P4	P8	7,274	99.0	72.7	72.6	552	408	387	110	867	569

FACSDiva Version 6.1.3

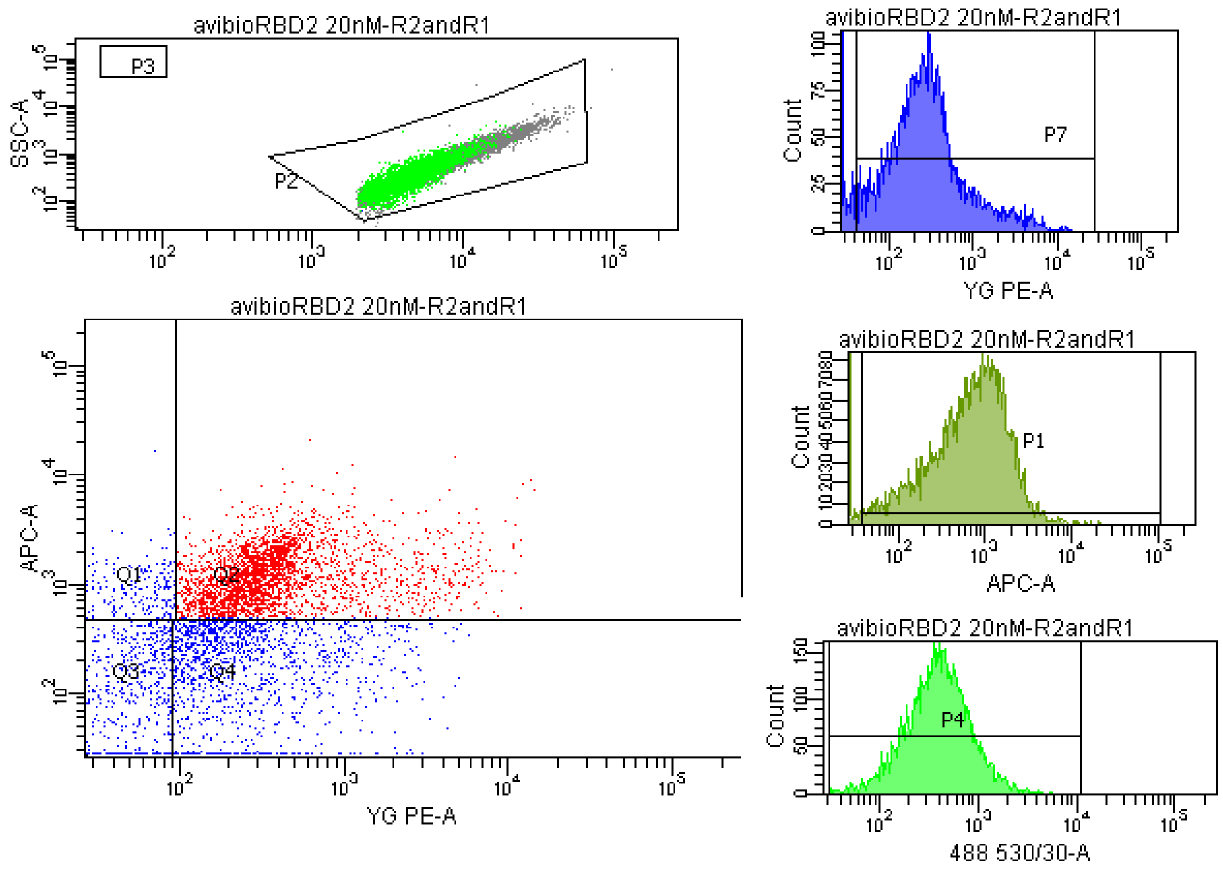

Experiment Name:	Bio RBD2 selectons
Specimen Name:	avibioRBD2 20nM
Tube Name:	R2andR1
Record Date:	Jun 17, 2020 5:58:39 PM
$OP:	Nileena
GUID:	b4540a12-7e03-46fc-8b51-d528e1dda040

Population	Parent...	#Events	%Parent	%Gran...	%Total	488 5... Mean	488 5... Median	PE-A Mean	PE-A Median	APC-A Mean	APC-A Median
All Events	####	####	####	####	####	901	510	347	137	1,277	649
P2	####	####	####	####	####	892	510	341	137	1,263	649
P8	P2	6,833	68.3	68.2	68.2	502	387	185	92	702	411
Q1	P8	299	4.4	3.0	3.0	381	327	58	35	1,201	877
Q2	P8	2,919	42.7	29.2	29.1	655	505	305	158	1,362	1,103
Q3	P8	2,172	31.8	21.7	21.7	350	280	37	24	64	32
Q4	P8	1,443	21.1	14.4	14.4	443	371	193	111	224	221
P7	P8	4,889	71.5	48.9	48.8	566	439	246	132	923	690
P1	P8	5,526	80.9	55.3	55.2	547	424	218	112	869	615
P4	P8	6,751	98.8	67.5	67.4	498	390	187	94	705	419

FACSDiva Version 6.1.3

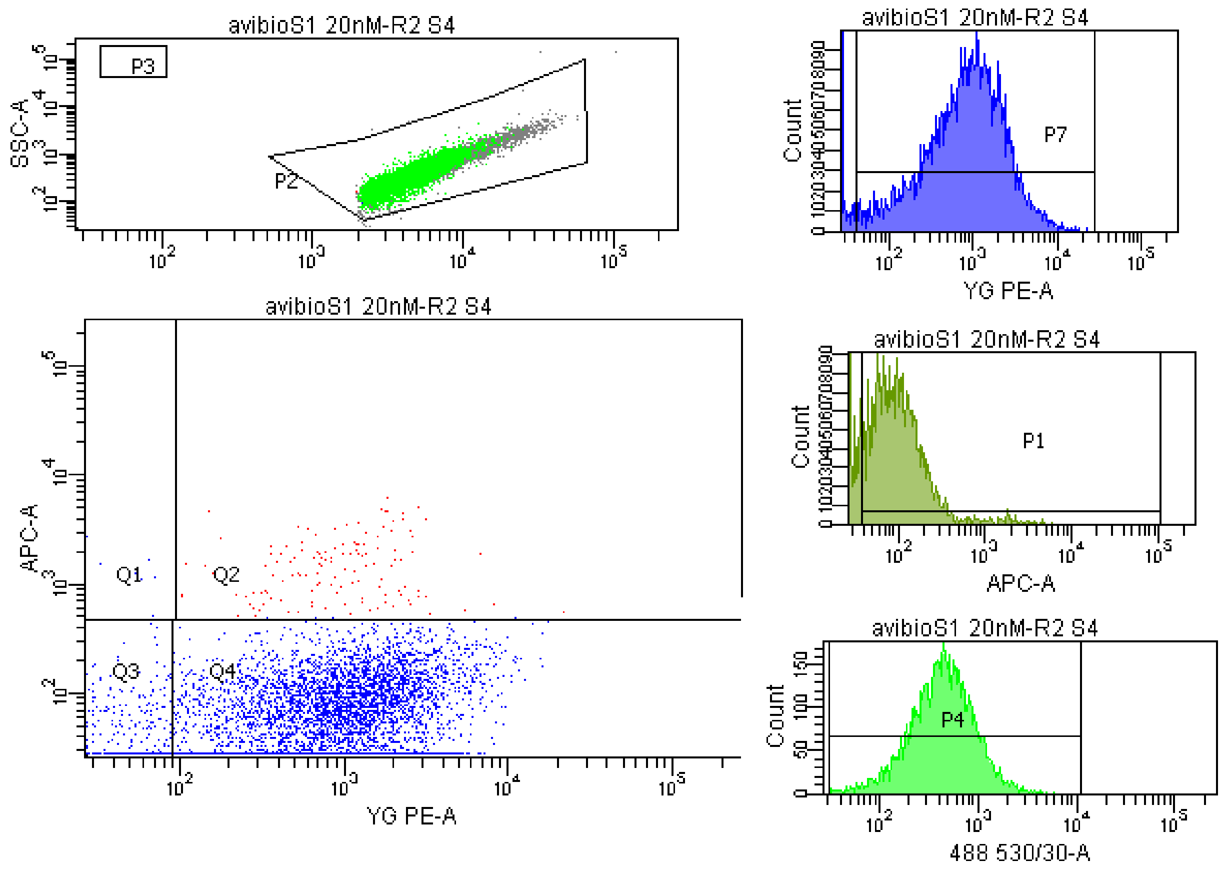

Experiment Name: Bio RBD2 selectons
Specimen Name: avibioS1 20nM
Tube Name: R2 S4
Record Date: Jun 17, 2020 6:00:14 PM
$OP: Nileena
GUID: c9a1eb29-c5d6-4f31-aab8-624abaad4078

Population	Parent...	#Events	%Parent	%Gran...	%Total	488 5... Mean	488 5... Median	PE-A Mean	PE-A Median	APC-A Mean	APC-A Median
All Events	####	####	####	####	####	877	536	629	313	161	54
P2	####	####	####	####	####	855	537	601	314	157	54
P8	P2	7,336	73.4	73.2	73.2	542	429	372	227	85	40
Q1	P8	16	0.2	0.2	0.2	296	241	47	38	2,655	2,288
Q2	P8	131	1.8	1.3	1.3	680	498	539	384	1,629	1,401
Q3	P8	1,884	25.7	18.8	18.8	311	244	33	22	18	10
Q4	P8	5,305	72.3	53.0	52.9	621	500	490	347	62	50
P7	P8	5,652	77.0	56.5	56.4	612	490	474	331	98	51
P1	P8	3,731	50.9	37.3	37.2	657	519	497	337	171	90
P4	P8	7,273	99.1	72.7	72.6	547	434	375	231	85	40

FACSDiva Version 6.1.3

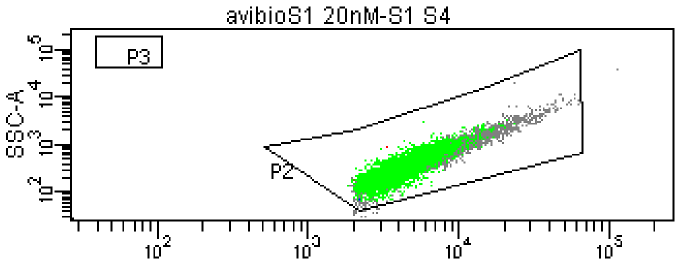

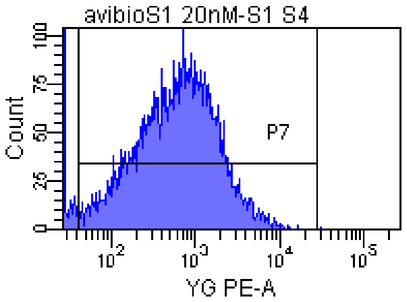

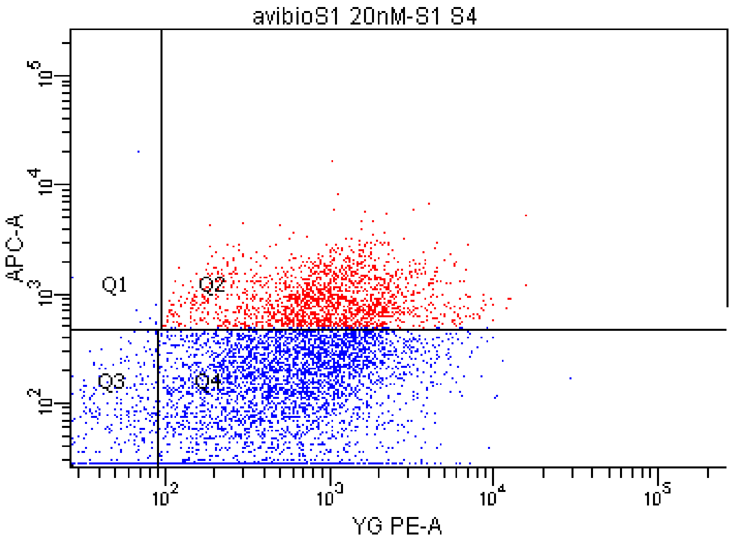

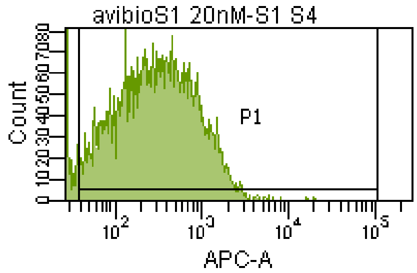

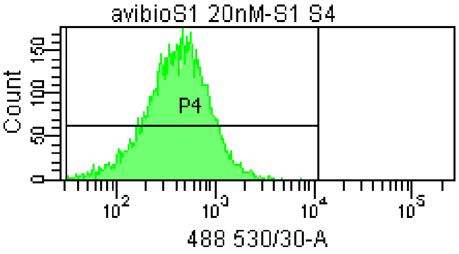

Experiment Name: Bio RBD2 selectons
Specimen Name: avibioS1 20nM
Tube Name: S1 S4
Record Date: Jun 17, 2020 6:01:01 PM
$OP: Nileena
GUID: 9c7e42b3-1848-4ddd-bb95-0109581c8636

Population	Parent...	#Events	%Parent	%Gran...	%Total	488 5... Mean	488 5... Median	PE-A Mean	PE-A Median	APC-A Mean	APC-A Median
All Events	####	####	####	####	####	805	502	496	241	498	205
P2	####	####	####	####	####	799	504	491	241	495	205
P8	P2	7,498	75.0	74.8	74.8	513	416	309	180	345	150
Q1	P8	14	0.2	0.1	0.1	395	235	68	40	4,903	681
Q2	P8	1,766	23.6	17.7	17.6	722	610	531	372	1,027	832
Q3	P8	1,838	24.5	18.4	18.3	310	251	36	23	27	15
Q4	P8	3,880	51.7	38.8	38.7	515	429	338	220	169	146
P7	P8	5,921	79.0	59.2	59.1	566	471	381	251	425	238
P1	P8	5,568	74.3	55.7	55.6	575	480	378	250	467	267
P4	P8	7,418	98.9	74.2	74.0	519	420	312	183	349	152

FACSDiva Version 6.1.3

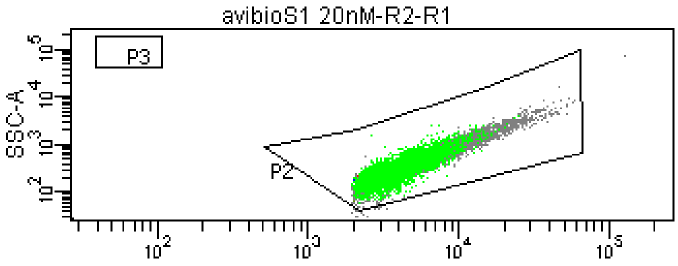

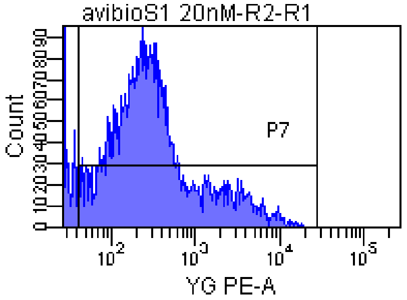

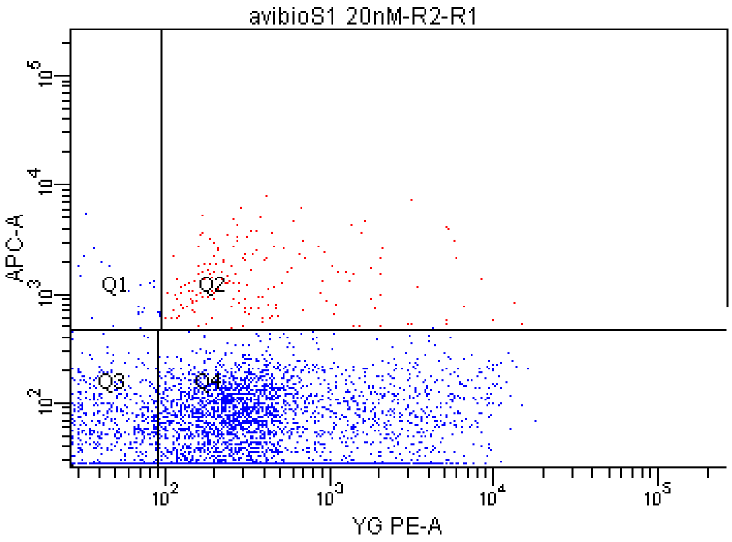

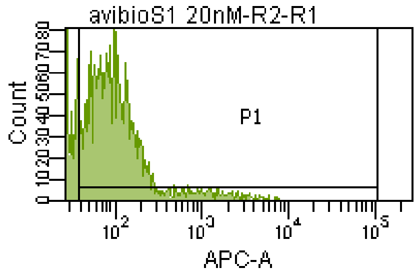

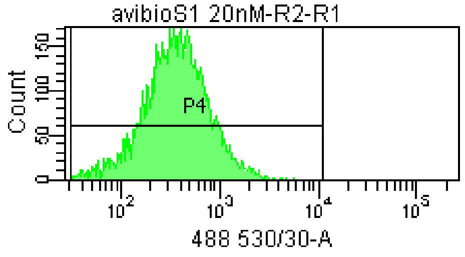

Experiment Name: Bio RBD2 selectons
Specimen Name: avibioS1 20nM
Tube Name: R2-R1
Record Date: Jun 17, 2020 6:01:50 PM
$OP: Nileena
GUID: 64f4ec36-a2fe-48a0-8d8d-0d52a7e1c208

Population	Parent...	#Events	%Parent	%Gran...	%Total	488 5... Mean	488 5... Median	PE-A Mean	PE-A Median	APC-A Mean	APC-A Median
All Events	####	####	####	####	####	709	415	331	99	140	43
P2	####	####	####	####	####	701	416	323	99	139	43
P8	P2	7,782	77.8	77.6	77.6	454	352	205	76	94	32
Q1	P8	62	0.8	0.6	0.6	410	342	49	50	1,931	1,365
Q2	P8	175	2.2	1.8	1.7	632	448	380	154	1,660	1,213
Q3	P8	3,373	43.3	33.7	33.6	337	269	38	24	31	19
Q4	P8	4,172	53.6	41.7	41.6	542	429	335	144	52	40
P7	P8	4,905	63.0	49.0	48.9	526	412	305	128	111	41
P1	P8	3,607	46.4	36.1	36.0	555	431	282	104	208	88
P4	P8	7,687	98.8	76.9	76.7	460	356	207	77	94	33

FACSDiva Version 6.1.3

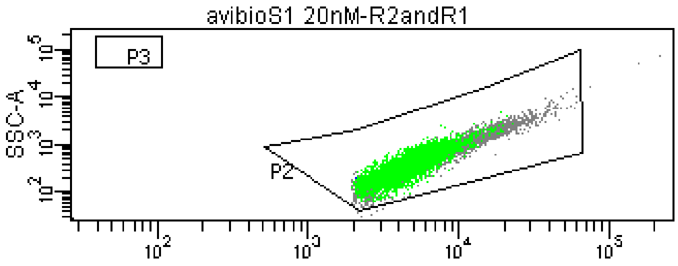

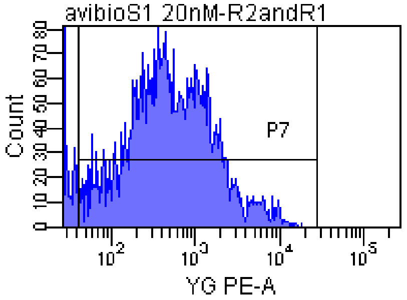

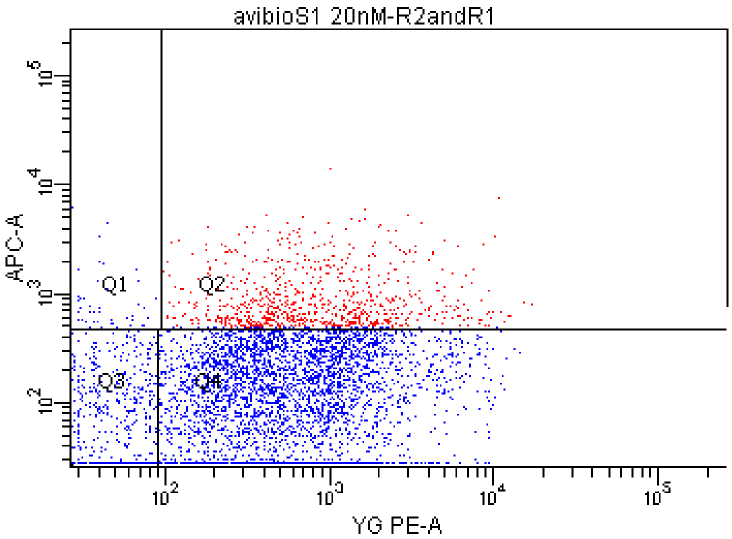

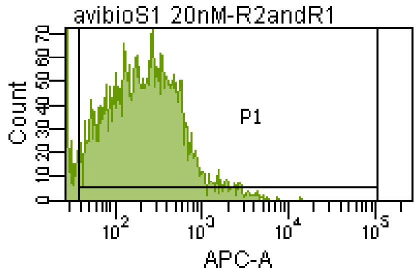

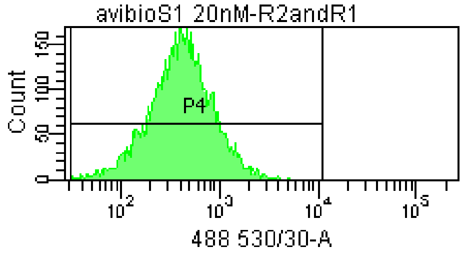

Experiment Name: Bio RBD2 selectons
Specimen Name: avibioS1 20nM
Tube Name: R2andR1
Record Date: Jun 17, 2020 6:02:43 PM
$OP: Nileena
GUID: ca9126b1-1b7d-4f73-88e7-71f126920b4d

Population	Parent...	#Events	%Parent	%Gran...	%Total	488 5... Mean	488 5... Median	PE-A Mean	PE-A Median	APC-A Mean	APC-A Median
All Events	####	####	####	####	####	858	495	503	179	339	128
P2	####	####	####	####	####	838	495	483	179	330	129
P8	P2	7,287	72.9	72.7	72.7	514	404	273	125	230	93
Q1	P8	107	1.5	1.1	1.1	431	373	57	40	1,163	725
Q2	P8	798	11.0	8.0	8.0	817	626	570	318	1,072	710
Q3	P8	2,451	33.6	24.5	24.5	342	286	36	25	51	26
Q4	P8	3,931	53.9	39.3	39.2	561	451	367	211	146	117
P7	P8	5,096	69.9	51.0	50.9	591	467	377	206	290	144
P1	P8	4,908	67.4	49.1	49.0	589	463	346	175	344	182
P4	P8	7,207	98.9	72.1	71.9	519	408	276	126	232	95

FACSDiva Version 6.1.3

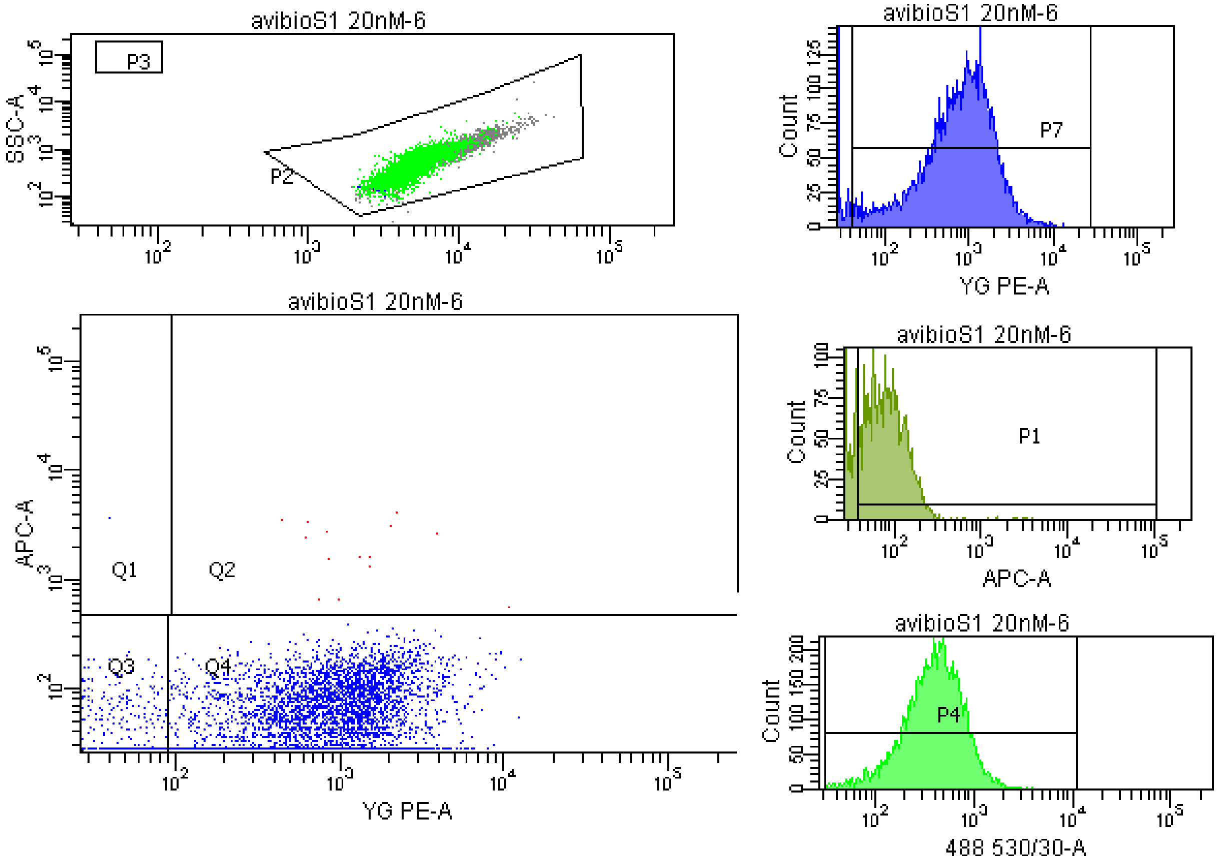

Experiment Name: Bio RBD2 selectons
Specimen Name: avibioS1 20nM
Tube Name: 6
Record Date: Jun 17, 2020 6:09:56 PM
$OP: Nileena
GUID: 4f19e81c-3f59-458e-ac8a-d8b26e6354e3

Population	Parent...	#Events	%Parent	%Gran...	%Total	488 5... Mean	488 5... Median	PE-A Mean	PE-A Median	APC-A Mean	APC-A Median
All Events	####	####	####	####	####	566	435	398	266	93	29
P2	####	####	####	####	####	567	435	398	266	93	29
P8	P2	8,737	87.4	87.3	87.3	456	392	314	228	46	24
Q1	P8	18	0.2	0.2	0.2	335	303	34	41	5,326	4,510
Q2	P8	14	0.2	0.1	0.1	871	768	814	446	2,047	1,948
Q3	P8	2,527	28.9	25.3	25.3	310	262	52	33	15	8
Q4	P8	6,178	70.7	61.8	61.8	515	450	421	337	39	31
P7	P8	6,454	73.9	64.5	64.5	509	445	408	325	42	30
P1	P8	3,553	40.7	35.5	35.5	539	465	407	315	124	79
P4	P8	8,595	98.4	86.0	85.9	464	398	319	233	47	25

FACSDiva Version 6.1.3

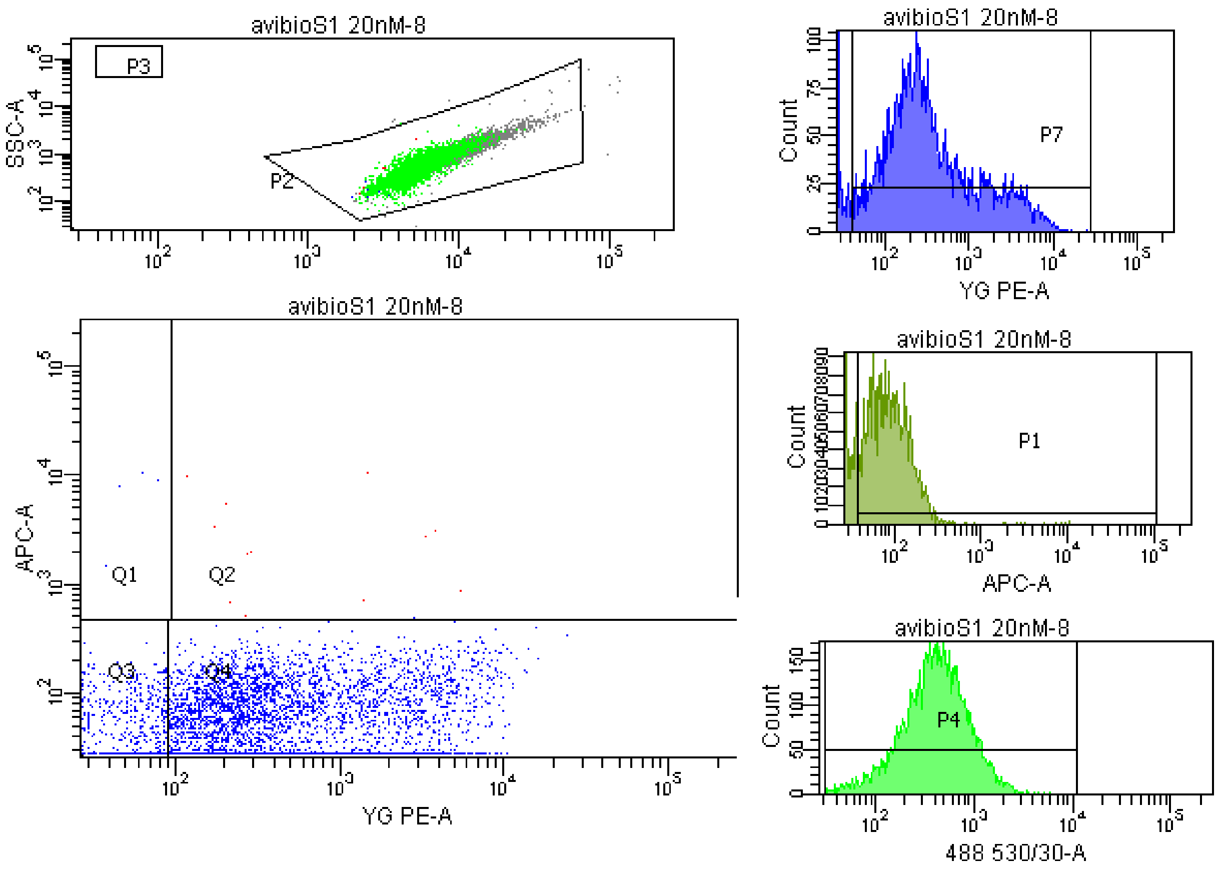

Experiment Name: Bio RBD2 selectons
Specimen Name: avibioS1 20nM
Tube Name: 8
Record Date: Jun 17, 2020 6:11:00 PM
$OP: Nileena
GUID: 441b84ba-82f9-4ab0-a1f8-a3eb5afe92d2

Population	Parent...	#Events	%Parent	%Gran...	%Total	488 5... Mean	488 5... Median	PE-A Mean	PE-A Median	APC-A Mean	APC-A Median
All Events	####	####	####	####	####	863	498	514	153	212	40
P2	####	####	####	####	####	801	497	477	153	161	40
P8	P2	7,798	78.0	77.9	77.9	507	404	275	114	55	29
Q1	P8	19	0.2	0.2	0.2	289	180	100	46	4,729	4,818
Q2	P8	12	0.2	0.1	0.1	907	626	597	332	3,360	2,242
Q3	P8	2,971	38.1	29.7	29.7	335	293	57	49	20	14
Q4	P8	4,796	61.5	48.0	47.9	613	499	410	185	49	40
P7	P8	5,326	68.3	53.3	53.2	592	481	378	168	60	38
P1	P8	3,448	44.2	34.5	34.4	628	494	395	156	132	81
P4	P8	7,673	98.4	76.7	76.6	515	410	279	117	54	29

FACSDiva Version 6.1.3

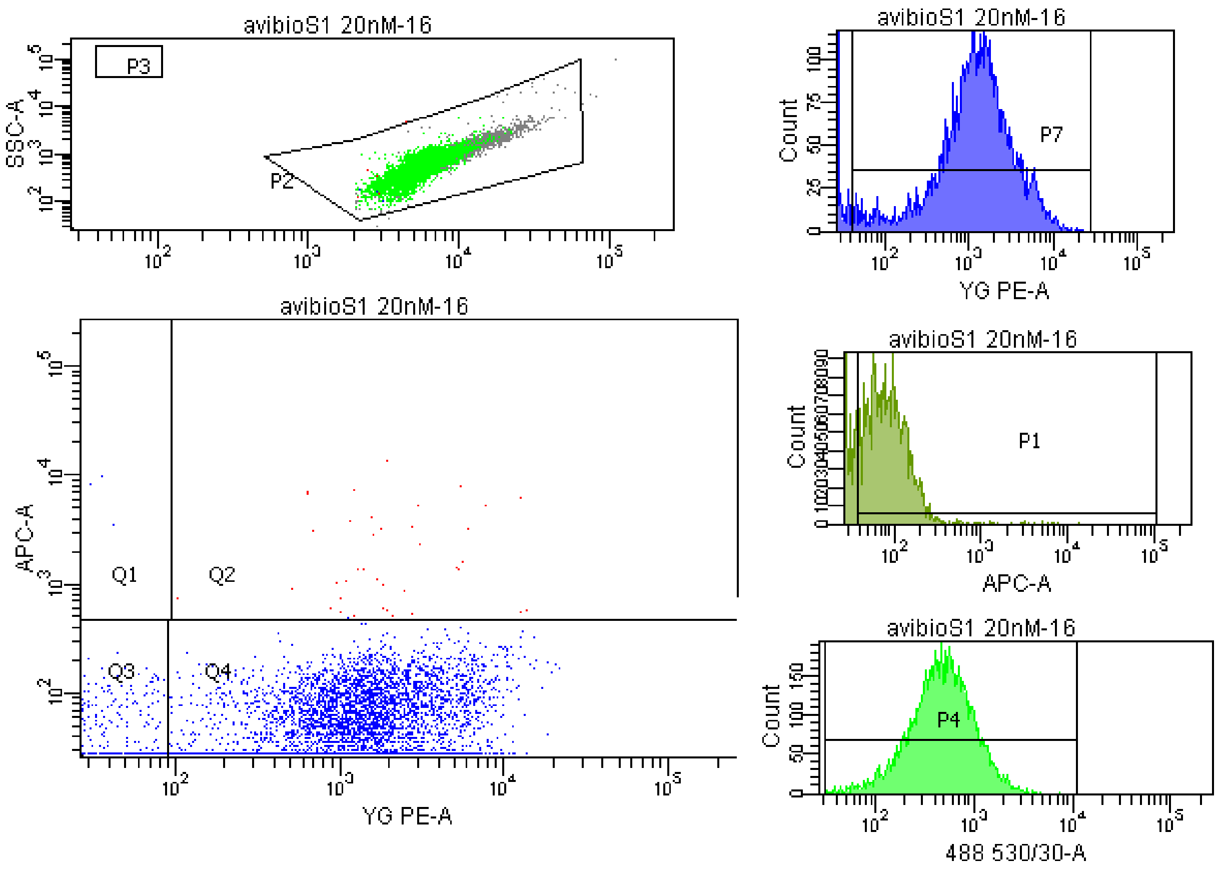

Experiment Name:	Bio RBD2 selectons
Specimen Name:	avibioS1 20nM
Tube Name:	16
Record Date:	Jun 17, 2020 6:12:04 PM
$OP:	Nileena
GUID:	1a3366f2-fb0d-46e2-b016-35593e10ee69

Population	Parent...	#Events	%Parent	%Gran...	%Total	488 5... Mean	488 5... Median	PE-A Mean	PE-A Median	APC-A Mean	APC-A Median
All Events	####	####	####	####	####	828	546	718	393	214	33
P2	####	####	####	####	####	811	546	704	393	183	33
P8	P2	8,214	82.1	82.1	82.1	581	465	511	330	64	25
Q1	P8	31	0.4	0.3	0.3	406	258	95	66	5,214	4,417
Q2	P8	38	0.5	0.4	0.4	1,163	753	1,201	714	2,648	1,301
Q3	P8	2,297	28.0	23.0	22.9	321	270	57	45	18	11
Q4	P8	5,848	71.2	58.5	58.4	680	560	686	487	38	30
P7	P8	6,077	74.0	60.8	60.7	673	553	671	470	55	30
P1	P8	3,397	41.4	34.0	33.9	719	573	674	448	166	79
P4	P8	8,101	98.6	81.0	80.9	589	471	517	336	63	26

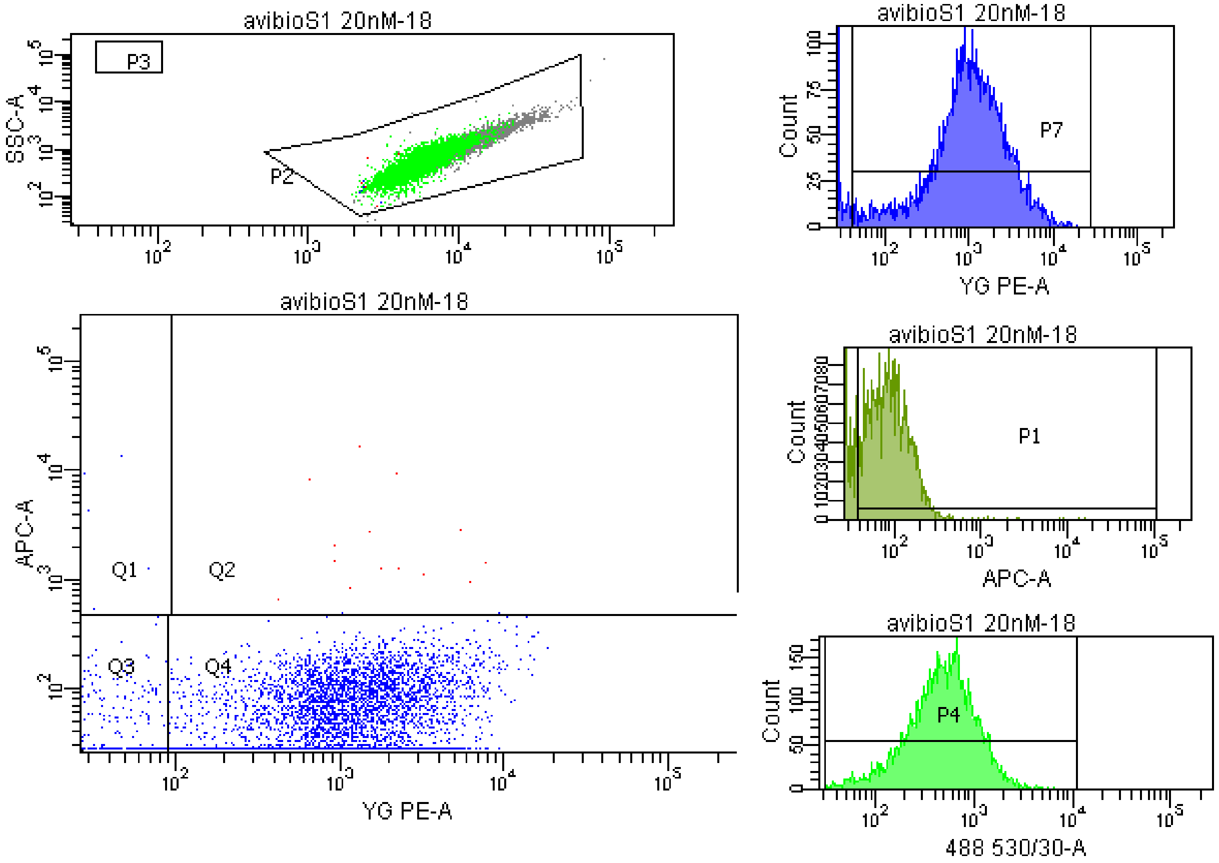

Experiment Name: Bio RBD2 selectons
Specimen Name: avibioS1 20nM
Tube Name: 18
Record Date: Jun 17, 2020 6:13:05 PM
$OP: Nileena
GUID: d7fd3a78-0c01-4c63-88da-25d03fc903ff

Population	Parent...	#Events	%Parent	%Gran...	%Total	488 5... Mean	488 5... Median	PE-A Mean	PE-A Median	APC-A Mean	APC-A Median
All Events	####	####	####	####	####	933	625	725	403	144	43
P2	####	####	####	####	####	919	625	719	403	140	43
P8	P2	7,322	73.2	73.2	73.2	591	477	448	293	64	30
Q1	P8	17	0.2	0.2	0.2	297	303	58	45	8,508	8,328
Q2	P8	14	0.2	0.1	0.1	1,506	946	1,078	743	3,475	1,392
Q3	P8	2,115	28.9	21.2	21.1	303	254	54	43	18	11
Q4	P8	5,176	70.7	51.8	51.7	707	594	608	432	46	38
P7	P8	5,344	73.0	53.4	53.4	699	586	594	421	57	38
P1	P8	3,294	45.0	32.9	32.9	721	584	592	392	154	84
P4	P8	7,208	98.4	72.1	72.0	600	483	455	299	64	30

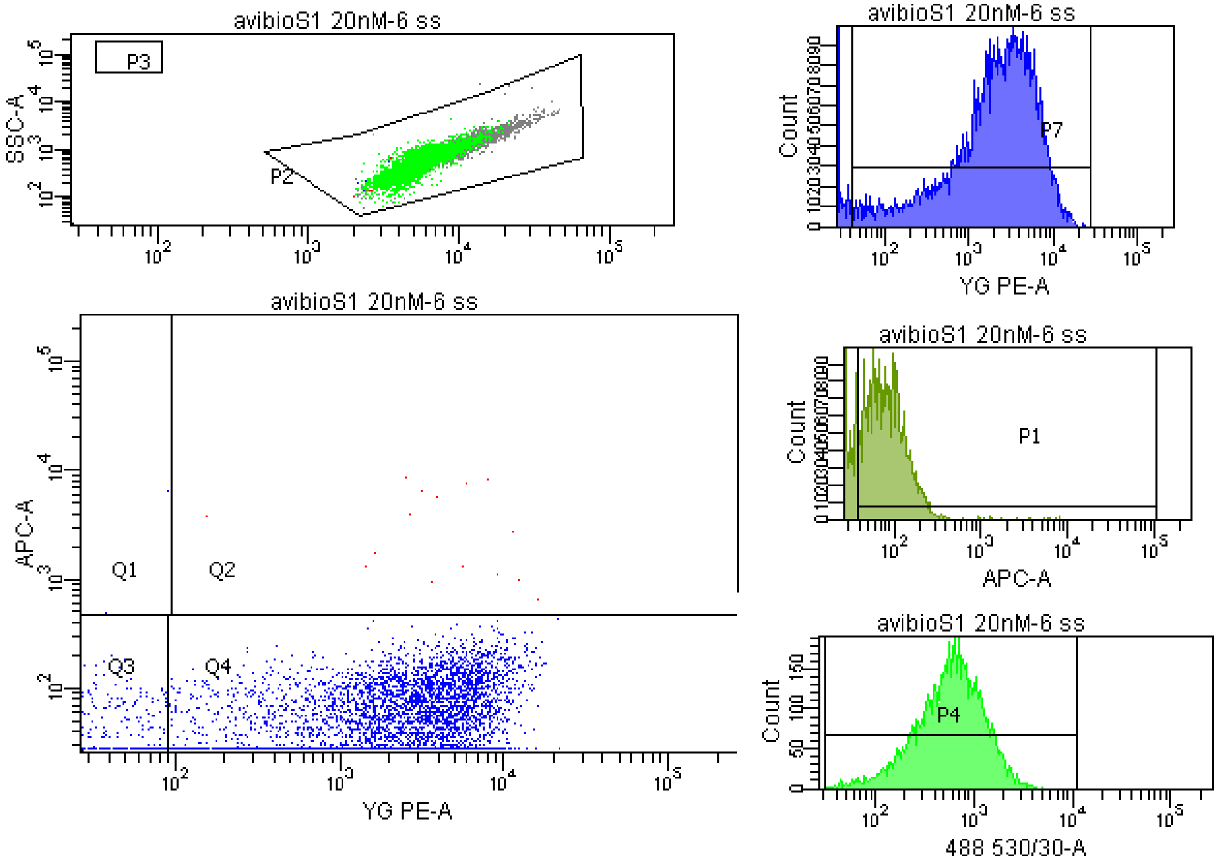

Experiment Name: Bio RBD2 selectons
Specimen Name: avibioS1 20nM
Tube Name: 6 ss
Record Date: Jun 17, 2020 6:14:13 PM
$OP: Nileena
GUID: 7f4b4b0e-137c-4e02-bdbe-e56b78177501

Population	Parent...	#Events	%Parent	%Gran...	%Total	488 5... Mean	488 5... Median	PE-A Mean	PE-A Median	APC-A Mean	APC-A Median
All Events	####	####	####	####	####	960	691	1,157	703	95	34
P2	####	####	####	####	####	960	691	1,157	702	95	34
P8	P2	8,114	81.1	81.1	81.1	709	586	841	549	52	24
Q1	P8	19	0.2	0.2	0.2	233	133	55	47	5,391	6,138
Q2	P8	15	0.2	0.2	0.1	1,561	1,256	2,100	1,445	3,528	2,621
Q3	P8	2,173	26.8	21.7	21.7	332	275	55	44	15	8
Q4	P8	5,907	72.8	59.1	59.1	847	718	1,130	873	40	32
P7	P8	6,108	75.3	61.1	61.1	834	702	1,100	837	49	31
P1	P8	3,328	41.0	33.3	33.3	902	749	1,171	874	136	77
P4	P8	8,025	98.9	80.2	80.2	717	591	850	564	52	24

FACSDiva Version 6.1.3

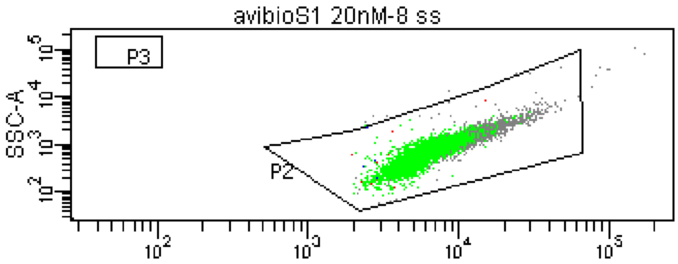

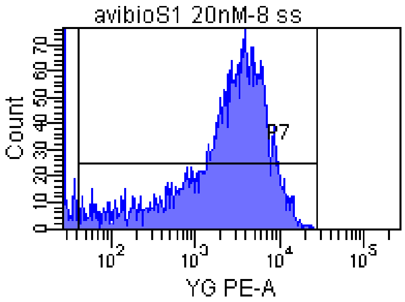

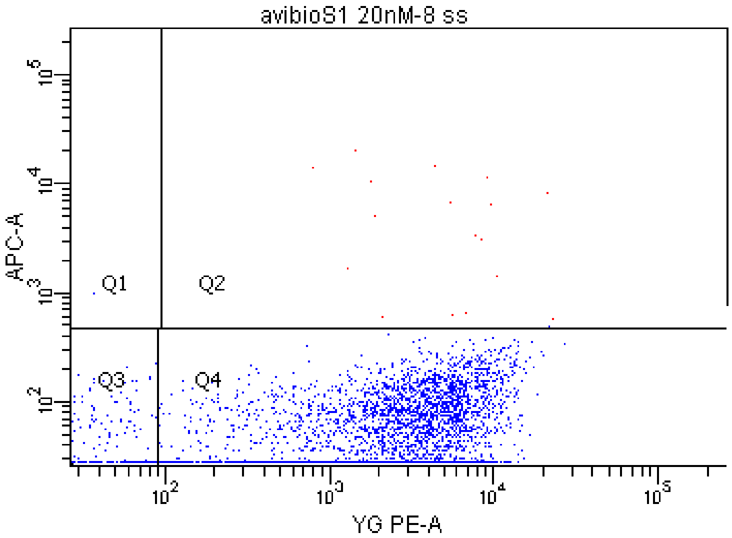

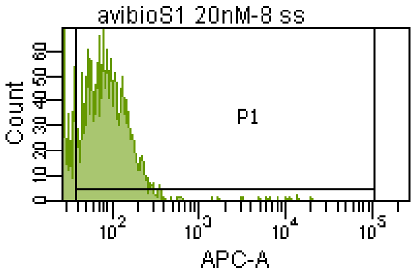

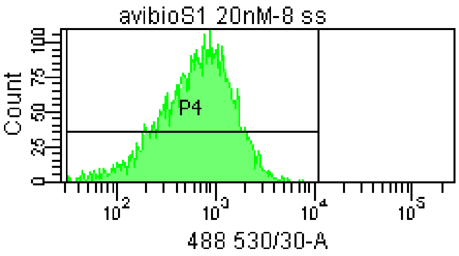

Experiment Name: Bio RBD2 selectons
Specimen Name: avibioS1 20nM
Tube Name: 8 ss
Record Date: Jun 17, 2020 6:15:55 PM
$OP: Nileena
GUID: e307b49d-b614-4789-b210-7b23cec7afb0

Population	Parent...	#Events	%Parent	%Gran...	%Total	488 5... Mean	488 5... Median	PE-A Mean	PE-A Median	APC-A Mean	APC-A Median
All Events	####	####	####	####	####	1,386	897	1,549	950	254	50
P2	####	####	####	####	####	1,325	897	1,522	949	218	50
P8	P2	5,161	71.9	71.8	71.8	831	668	954	665	91	33
Q1	P8	19	0.4	0.3	0.3	355	389	116	72	7,663	4,715
Q2	P8	17	0.3	0.2	0.2	3,092	2,732	2,815	2,524	6,195	4,770
Q3	P8	1,460	28.3	20.3	20.3	331	277	59	50	20	13
Q4	P8	3,665	71.0	51.1	51.0	1,022	876	1,307	1,082	51	42
P7	P8	3,805	73.7	53.0	52.9	1,011	862	1,272	1,043	78	41
P1	P8	2,417	46.8	33.7	33.6	1,051	885	1,284	1,008	200	82
P4	P8	5,085	98.5	70.8	70.7	841	680	967	682	91	34

FACSDiva Version 6.1.3

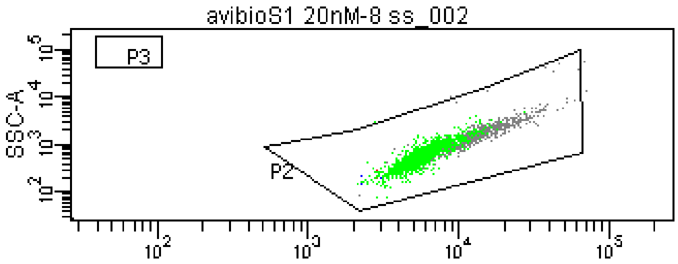

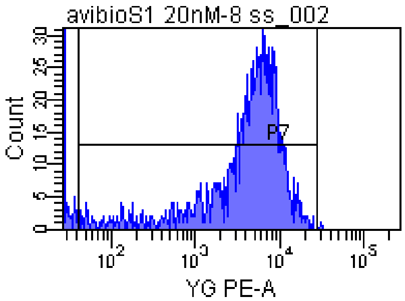

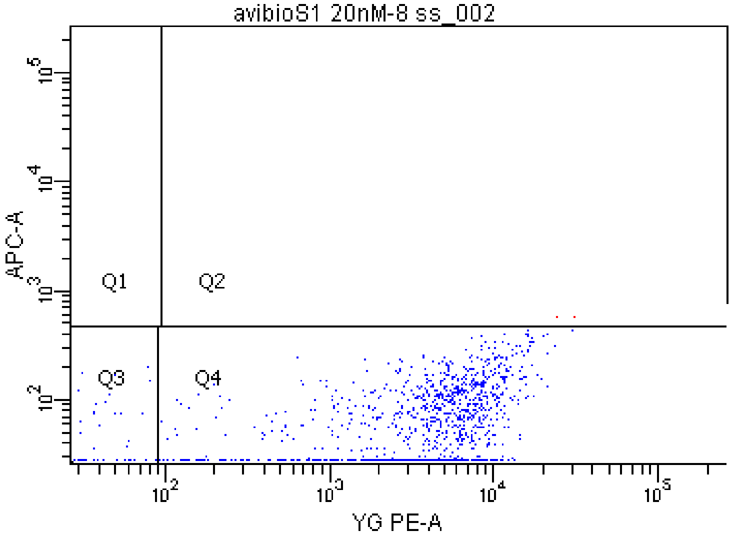

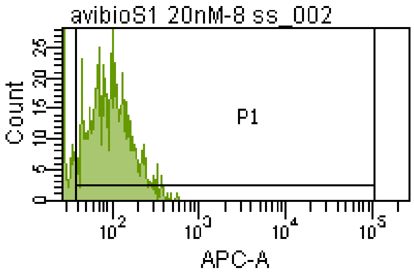

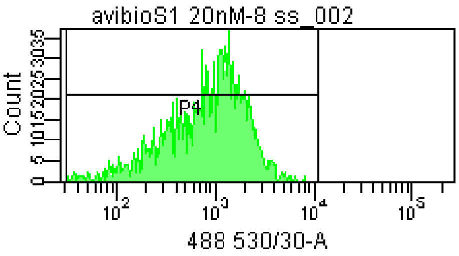

Experiment Name: Bio RBD2 selectons
Specimen Name: avibioS1 20nM
Tube Name: 8 ss_002
Record Date: Jun 17, 2020 6:23:31 PM
$OP: Nileena
GUID: 7401b544-0908-4270-98ef-0047f48ec545

Population	Parent...	#Events	%Parent	%Gran...	%Total	488 5... Mean	488 5... Median	PE-A Mean	PE-A Median	APC-A Mean	APC-A Median
All Events	####	####	####	####	####	1,857	1,256	2,478	1,778	223	68
P2	####	####	####	####	####	1,837	1,256	2,456	1,775	204	68
P8	P2	1,464	68.6	68.5	68.5	1,104	925	1,563	1,390	64	46
Q1	P8	1	0.1	0.0	0.0	1,393	1,393	202	202	9,275	9,275
Q2	P8	2	0.1	0.1	0.1	6,267	6,267	10,193	10,193	543	543
Q3	P8	343	23.4	16.1	16.1	315	270	54	46	19	12
Q4	P8	1,118	76.4	52.4	52.3	1,337	1,170	2,011	1,797	69	59
P7	P8	1,140	77.9	53.4	53.4	1,316	1,158	1,972	1,761	68	59
P1	P8	810	55.3	38.0	37.9	1,385	1,202	2,025	1,812	120	92
P4	P8	1,451	99.1	68.0	67.9	1,114	943	1,577	1,404	65	47

FACSDiva Version 6.1.3

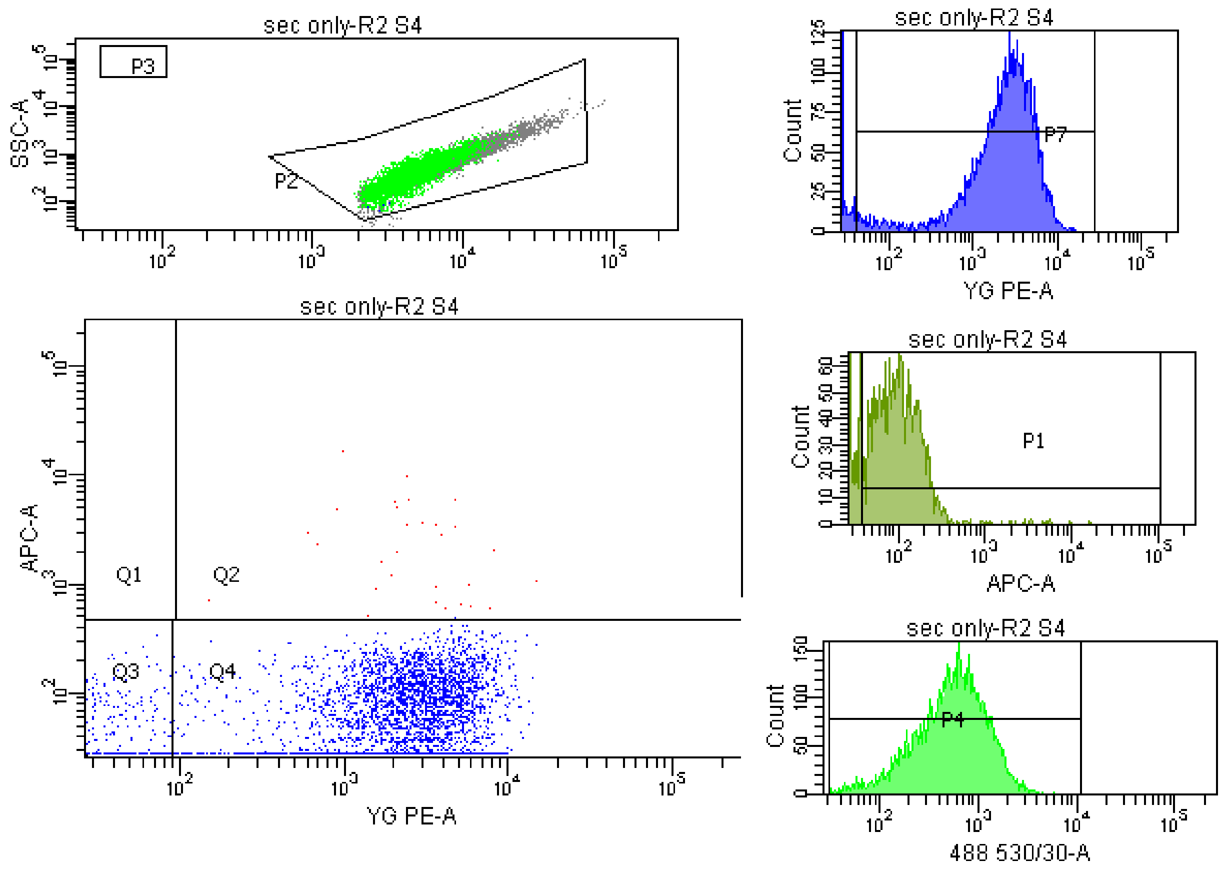

Experiment Name: Bio RBD2 selectons
Specimen Name: sec only
Tube Name: R2 S4
Record Date: Jun 23, 2020 3:50:30 PM
$OP: Nileena
GUID: 53aa2afb-a70a-4a39-b272-437d468ae582

Population	Parent...	#Events	%Parent	%Gran...	%Total	488 5... Mean	488 5... Median	PE-A Mean	PE-A Median	APC-A Mean	APC-A Median
All Events	####	####	####	####	####	1,125	742	1,201	849	97	34
P2	####	####	####	####	####	1,118	743	1,194	851	94	34
P8	P2	6,788	67.9	67.7	67.7	682	556	759	611	42	20
Q1	P8	8	0.1	0.1	0.1	342	288	13	-6	1,326	1,301
Q2	P8	29	0.4	0.3	0.3	940	670	1,111	1,003	3,017	1,933
Q3	P8	1,773	26.1	17.7	17.7	261	217	34	-2	8	1
Q4	P8	4,978	73.3	49.8	49.7	831	699	1,017	874	34	27
P7	P8	5,147	75.8	51.5	51.3	817	683	994	855	51	27
P1	P8	2,826	41.6	28.3	28.2	805	657	925	775	141	93
P4	P8	6,674	98.3	66.7	66.6	694	566	772	627	42	21

FACSDiva Version 6.1.3

sec only-S1 S4

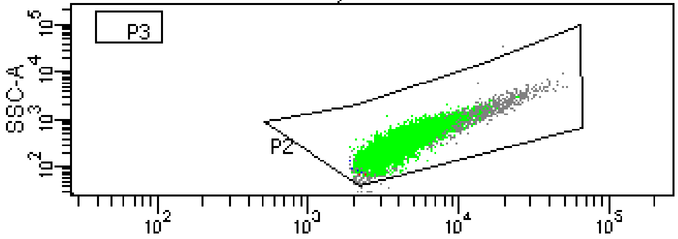

sec only-S1 S4

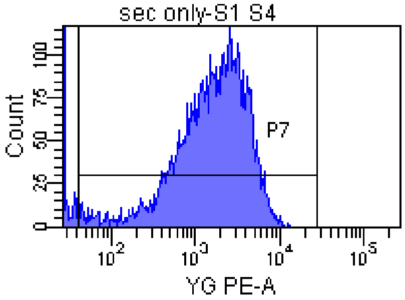

sec only-S1 S4

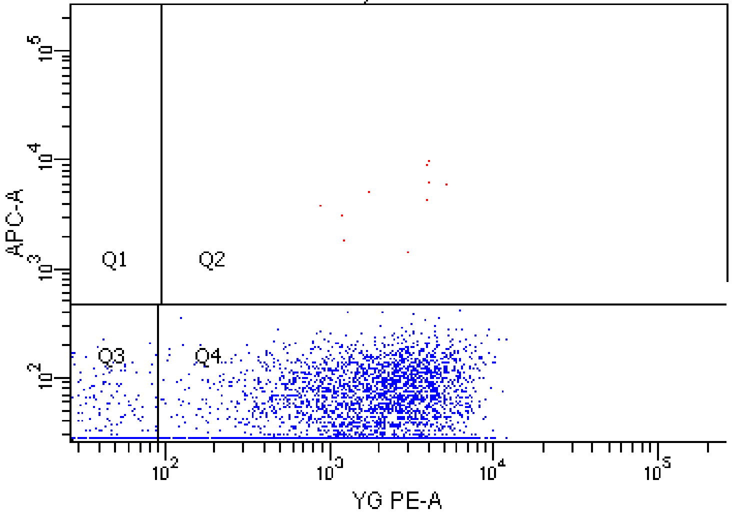

sec only-S1 S4

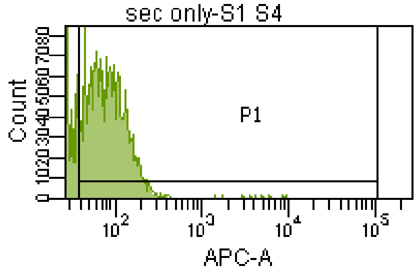

sec only-S1 S4

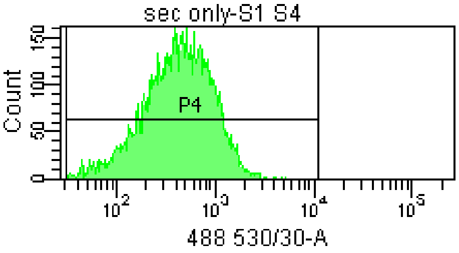

Experiment Name: Bio RBD2 selectons
Specimen Name: sec only
Tube Name: S1 S4
Record Date: Jun 23, 2020 3:51:39 PM
$OP: Nileena
GUID: 91b76602-991b-42dc-96c2-fa4b2c352c0f

Population	Parent...	#Events	%Parent	%Gran...	%Total	488 5... Mean	488 5... Median	PE-A Mean	PE-A Median	APC-A Mean	APC-A Median
All Events	####	####	####	####	####	728	481	721	461	51	17
P2	####	####	####	####	####	727	483	721	463	45	17
P8	P2	7,989	79.9	79.7	79.7	525	421	545	379	26	12
Q1	P8	4	0.1	0.0	0.0	309	311	105	49	2,130	1,574
Q2	P8	10	0.1	0.1	0.1	923	801	932	1,010	4,833	4,429
Q3	P8	2,178	27.3	21.8	21.7	228	190	24	0	7	-1
Q4	P8	5,797	72.6	58.0	57.8	636	537	740	596	23	15
P7	P8	5,945	74.4	59.4	59.3	629	531	725	581	31	16
P1	P8	2,741	34.3	27.4	27.3	636	522	687	538	109	77
P4	P8	7,822	97.9	78.2	78.0	536	431	556	396	26	12

FACSDiva Version 6.1.3

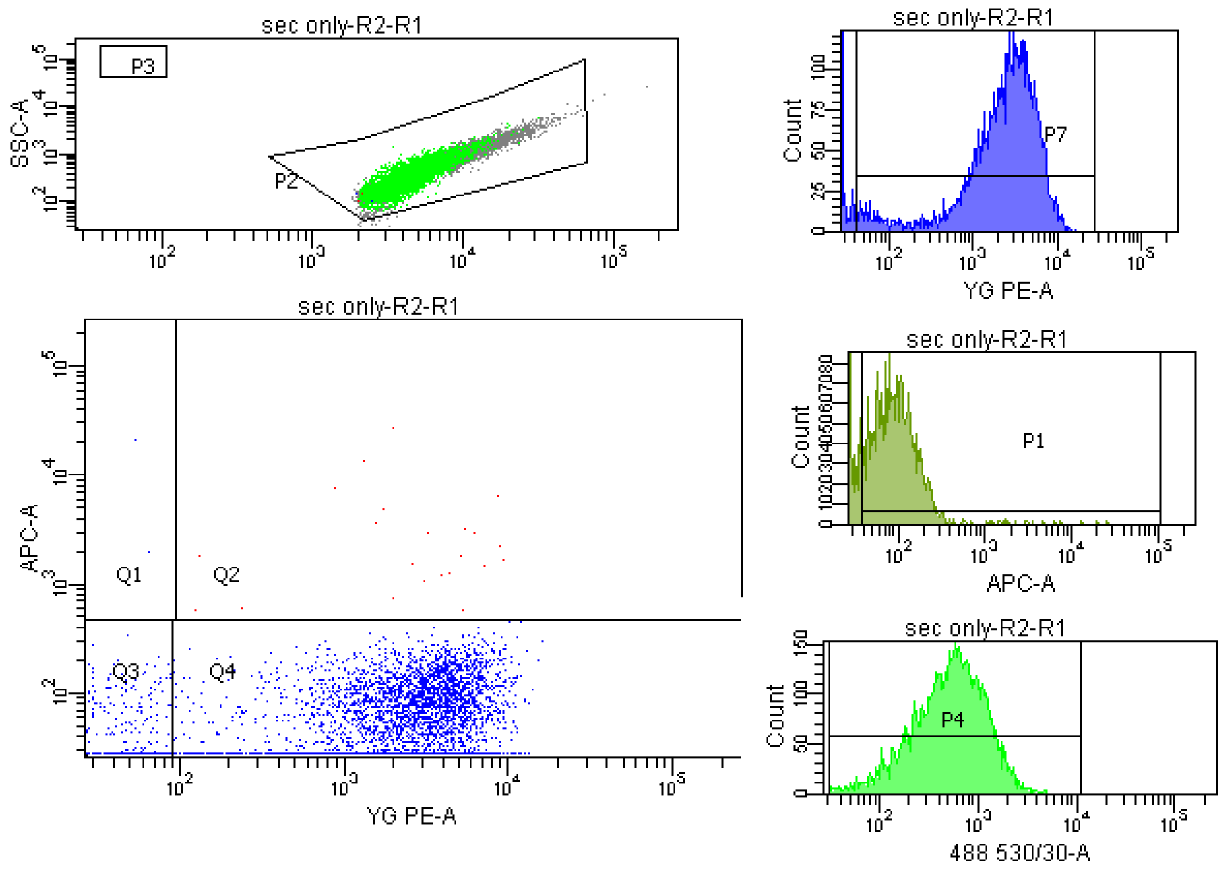

Experiment Name: Bio RBD2 selectons
Specimen Name: sec only
Tube Name: R2-R1
Record Date: Jun 23, 2020 3:52:30 PM
$OP: Nileena
GUID: 4d5acb32-875d-4716-8423-6f0876e4fb86

Population	Parent...	#Events	%Parent	%Gran...	%Total	488 5... Mean	488 5... Median	PE-A Mean	PE-A Median	APC-A Mean	APC-A Median
All Events	####	####	####	####	####	972	632	1,077	754	79	30
P2	####	####	####	####	####	963	633	1,065	756	74	30
P8	P2	7,615	76.1	76.0	76.0	651	523	758	581	44	20
Q1	P8	8	0.1	0.1	0.1	392	322	82	56	5,751	2,902
Q2	P8	22	0.3	0.2	0.2	1,104	838	1,354	1,038	3,864	1,716
Q3	P8	2,205	29.0	22.0	22.0	254	217	31	9	9	2
Q4	P8	5,380	70.7	53.8	53.7	812	681	1,054	900	34	28
P7	P8	5,611	73.7	56.1	56.0	796	663	1,019	869	53	28
P1	P8	3,116	40.9	31.2	31.1	793	649	961	820	141	87
P4	P8	7,493	98.4	74.9	74.8	662	533	770	602	45	21

FACSDiva Version 6.1.3

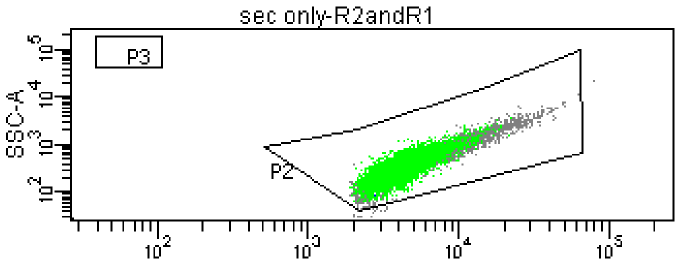

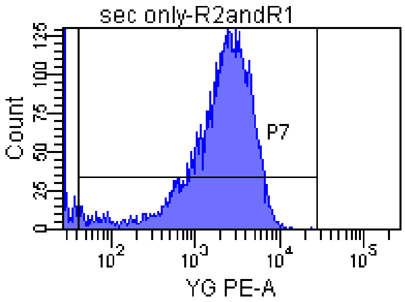

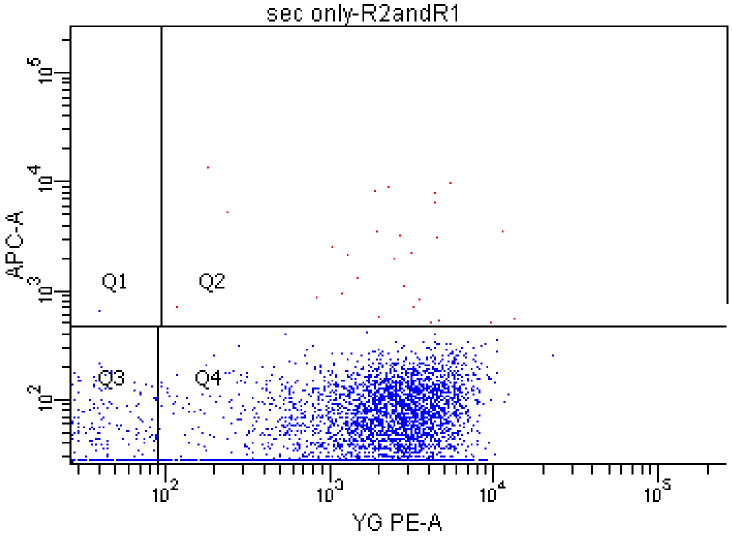

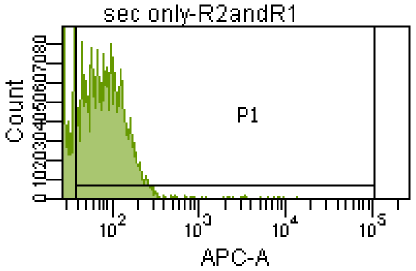

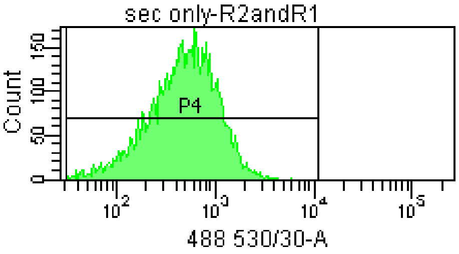

Experiment Name: Bio RBD2 selectons
Specimen Name: sec only
Tube Name: R2andR1
Record Date: Jun 23, 2020 3:53:25 PM
$OP: Nileena
GUID: b11ff262-d7ad-4046-a694-152889da0cb9

Population	Parent...	#Events	%Parent	%Gran...	%Total	488 5... Mean	488 5... Median	PE-A Mean	PE-A Median	APC-A Mean	APC-A Median
All Events	####	####	####	####	####	848	569	889	641	81	26
P2	####	####	####	####	####	847	571	887	642	81	27
P8	P2	7,906	79.1	78.8	78.8	593	484	648	530	39	19
Q1	P8	9	0.1	0.1	0.1	300	280	52	10	1,325	1,192
Q2	P8	27	0.3	0.3	0.3	1,021	669	1,244	777	3,244	2,087
Q3	P8	2,124	26.9	21.2	21.2	244	204	31	5	13	8
Q4	P8	5,746	72.7	57.5	57.2	721	619	874	759	31	24
P7	P8	5,928	75.0	59.3	59.0	712	610	855	741	46	24
P1	P8	3,078	38.9	30.8	30.7	713	586	801	689	125	81
P4	P8	7,775	98.3	77.8	77.4	604	494	659	546	39	19

FACSDiva Version 6.1.3

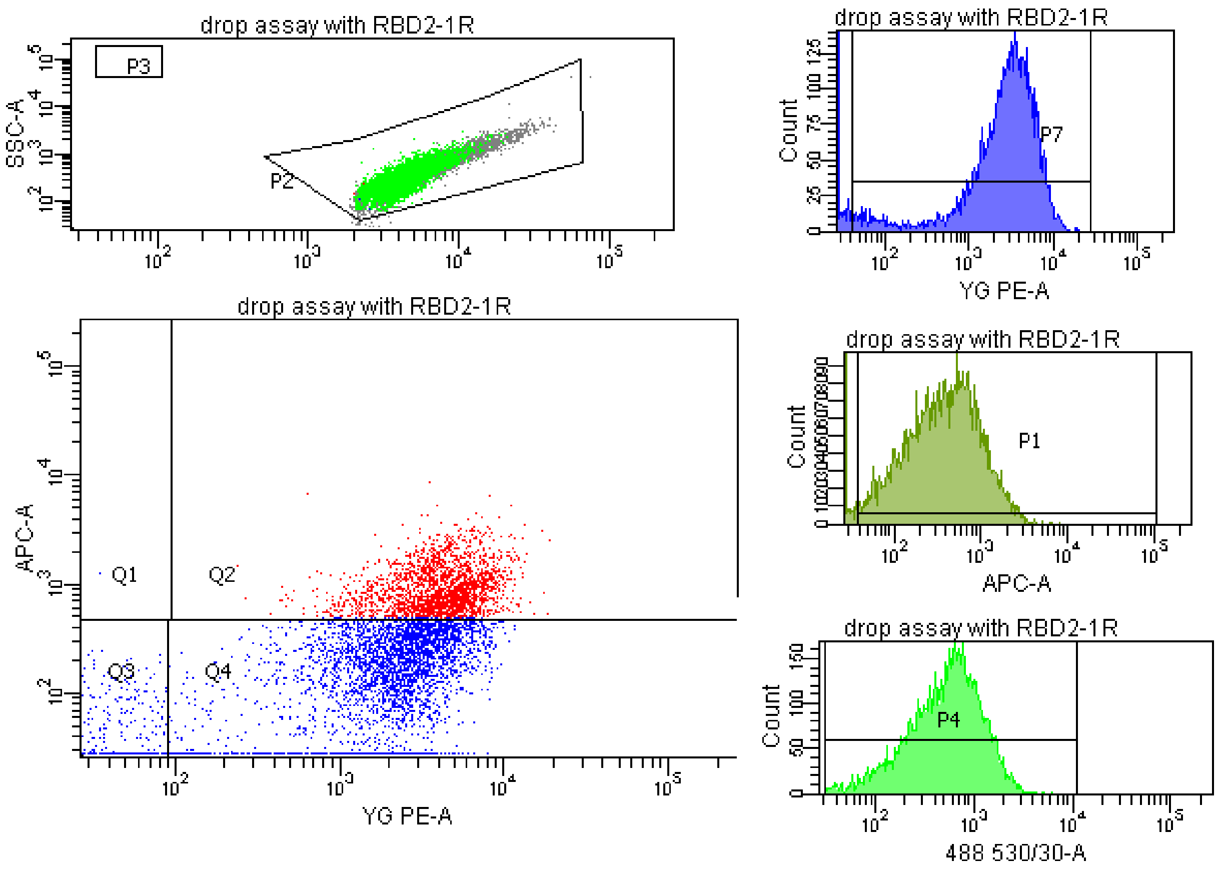

Experiment Name: Bio RBD2 selectons
Specimen Name: drop assay with RBD2
Tube Name: 1R
Record Date: Jun 23, 2020 3:57:04 PM
$OP: Nileena
GUID: 4cd32308-35db-4cd7-90dc-9e84aa1cf53a

Population	Parent...	#Events	%Parent	%Gran...	%Total	488 5... Mean	488 5... Median	PE-A Mean	PE-A Median	APC-A Mean	APC-A Median
All Events	####	####	####	####	####	876	618	1,063	778	492	230
P2	####	####	####	####	####	873	619	1,062	779	490	231
P8	P2	8,068	80.7	80.5	80.5	656	541	828	670	364	182
Q1	P8	8	0.1	0.1	0.1	436	348	131	69	4,463	4,472
Q2	P8	2,241	27.8	22.4	22.4	1,075	965	1,580	1,441	979	791
Q3	P8	2,349	29.1	23.5	23.4	230	201	30	13	10	3
Q4	P8	3,470	43.0	34.7	34.6	674	600	884	775	196	185
P7	P8	5,908	73.2	59.1	58.9	811	691	1,121	972	487	331
P1	P8	5,921	73.4	59.2	59.1	789	676	1,078	949	502	331
P4	P8	7,905	98.0	79.0	78.8	669	552	845	691	370	191

FACSDiva Version 6.1.3

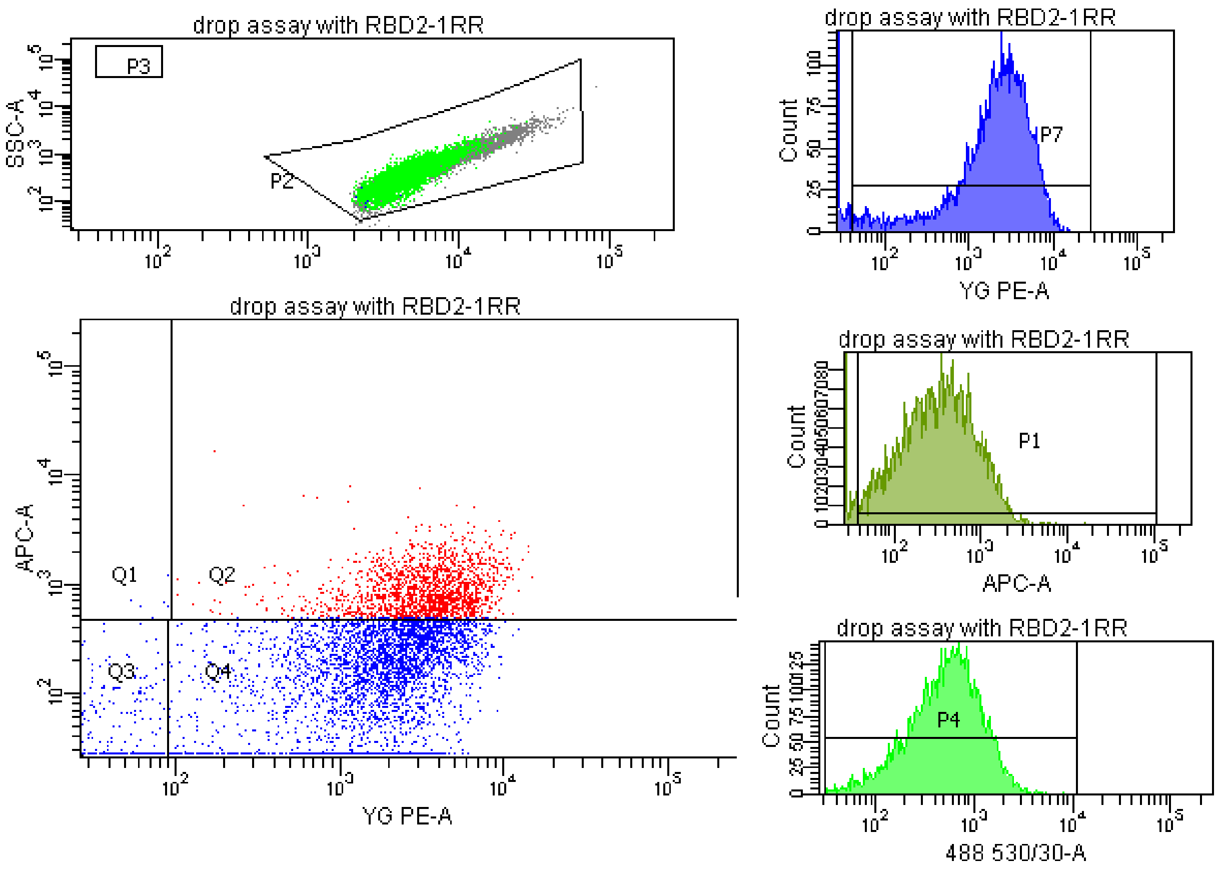

Experiment Name: Bio RBD2 selectons
Specimen Name: drop assay with RBD2
Tube Name: 1RR
Record Date: Jun 23, 2020 3:58:00 PM
$OP: Nileena
GUID: 0a23dd06-f2ee-4334-a3f4-2a278b2082e2

Population	Parent...	#Events	%Parent	%Gran...	%Total	488 5... Mean	488 5... Median	PE-A Mean	PE-A Median	APC-A Mean	APC-A Median
All Events	####	####	####	####	####	1,014	665	1,023	695	530	242
P2	####	####	####	####	####	1,013	665	1,021	697	530	242
P8	P2	7,328	73.3	73.2	73.2	655	526	698	525	327	164
Q1	P8	10	0.1	0.1	0.1	212	209	14	5	771	656
Q2	P8	1,723	23.5	17.2	17.2	1,089	966	1,359	1,230	976	782
Q3	P8	2,088	28.5	20.9	20.8	265	219	32	3	12	3
Q4	P8	3,507	47.9	35.1	35.0	676	591	771	652	194	178
P7	P8	5,393	73.6	53.9	53.8	797	668	939	774	440	283
P1	P8	5,368	73.3	53.7	53.6	778	652	901	749	452	288
P4	P8	7,209	98.4	72.1	72.0	666	535	709	542	332	170

FACSDiva Version 6.1.3

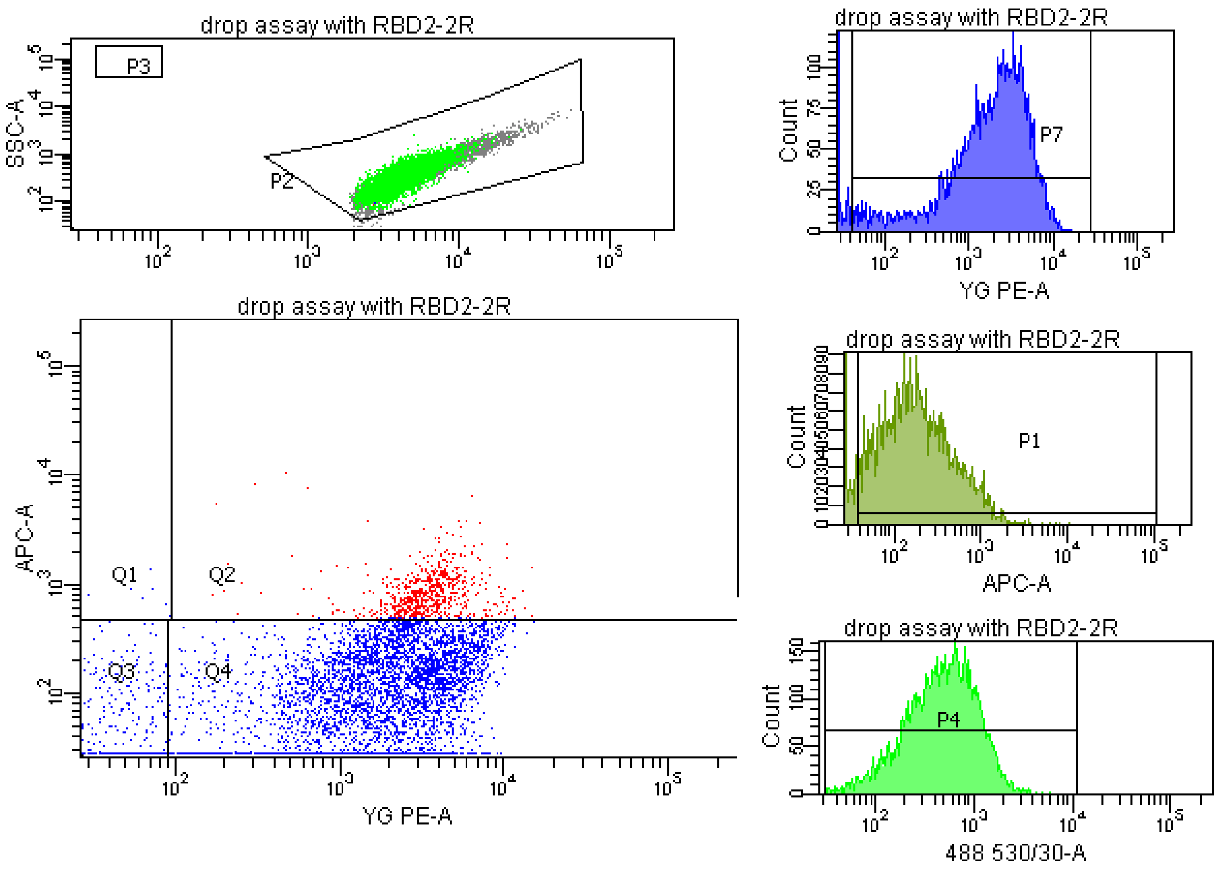

Experiment Name: Bio RBD2 selectons
Specimen Name: drop assay with RBD2
Tube Name: 2R
Record Date: Jun 23, 2020 4:00:55 PM
$OP: Nileena
GUID: 6c0177d4-56b7-418f-a73a-62381bd845a0

Population	Parent...	#Events	%Parent	%Gran...	%Total	488 5... Mean	488 5... Median	PE-A Mean	PE-A Median	APC-A Mean	APC-A Median
All Events	####	####	####	####	####	790	544	856	547	231	87
P2	####	####	####	####	####	792	545	858	549	232	88
P8	P2	7,999	80.0	79.6	79.6	592	473	657	451	161	72
Q1	P8	10	0.1	0.1	0.1	380	307	37	22	778	741
Q2	P8	718	9.0	7.2	7.1	1,015	878	1,320	1,176	913	736
Q3	P8	2,124	26.6	21.2	21.1	242	202	25	3	11	2
Q4	P8	5,147	64.3	51.5	51.2	678	572	826	638	116	91
P7	P8	6,088	76.1	60.9	60.6	704	594	858	686	208	110
P1	P8	4,960	62.0	49.6	49.4	725	619	880	722	270	152
P4	P8	7,846	98.1	78.5	78.1	604	483	669	470	164	74

FACSDiva Version 6.1.3

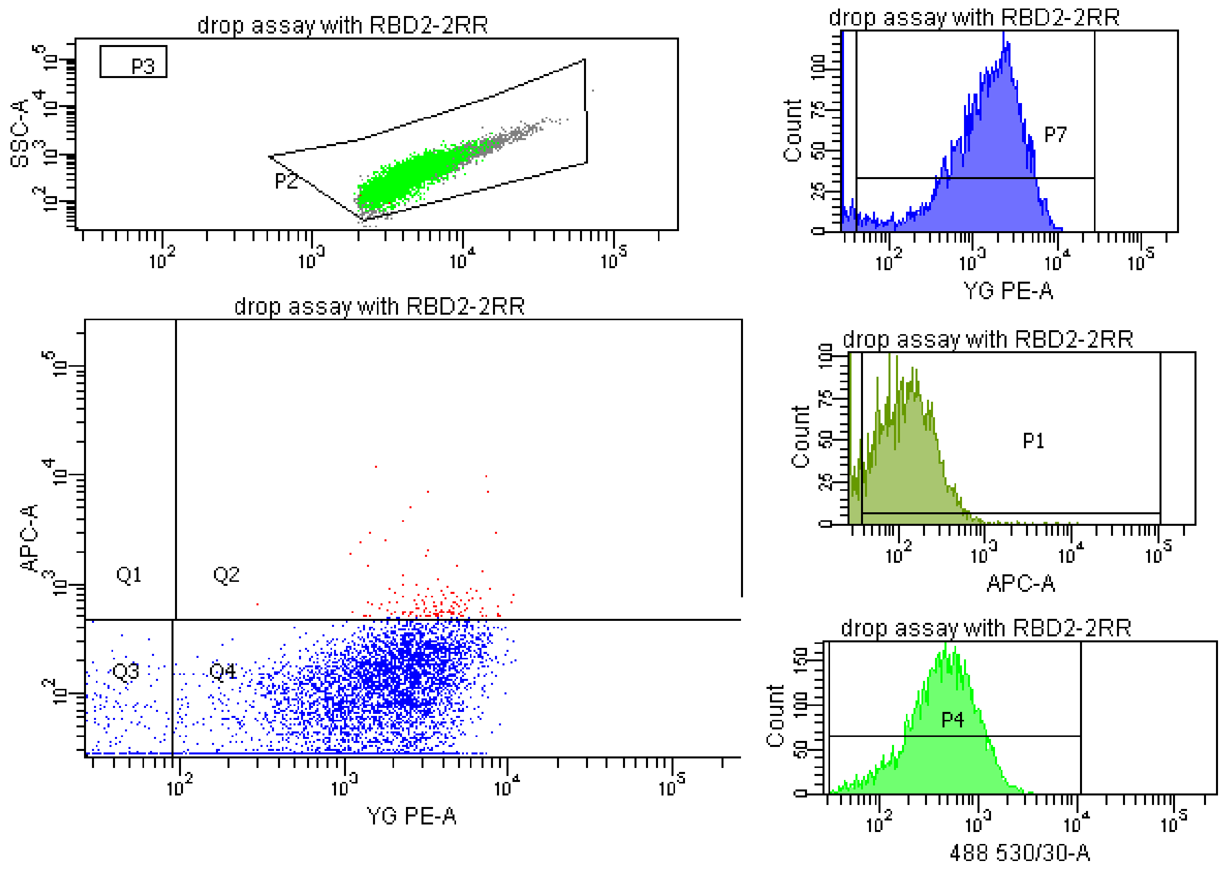

Experiment Name: Bio RBD2 selectons
Specimen Name: drop assay with RBD2
Tube Name: 2RR
Record Date: Jun 23, 2020 4:02:04 PM
$OP: Nileena
GUID: b3065f60-8f7d-4fd0-896f-d410780050dd

Population	Parent...	#Events	%Parent	%Gran...	%Total	488 5... Mean	488 5... Median	PE-A Mean	PE-A Median	APC-A Mean	APC-A Median
All Events	####	####	####	####	####	697	496	653	446	121	64
P2	####	####	####	####	####	695	497	652	446	121	64
P8	P2	8,056	80.6	80.3	80.3	538	439	527	378	89	55
Q1	P8	3	0.0	0.0	0.0	588	484	38	58	2,297	2,055
Q2	P8	134	1.7	1.3	1.3	1,121	997	1,408	1,312	1,043	594
Q3	P8	2,021	25.1	20.2	20.2	239	202	28	5	9	3
Q4	P8	5,898	73.2	59.0	58.8	627	537	679	549	94	75
P7	P8	6,170	76.6	61.7	61.5	631	536	681	547	113	75
P1	P8	4,589	57.0	45.9	45.8	653	559	709	598	169	120
P4	P8	7,907	98.2	79.1	78.8	549	448	537	391	90	57

FACSDiva Version 6.1.3

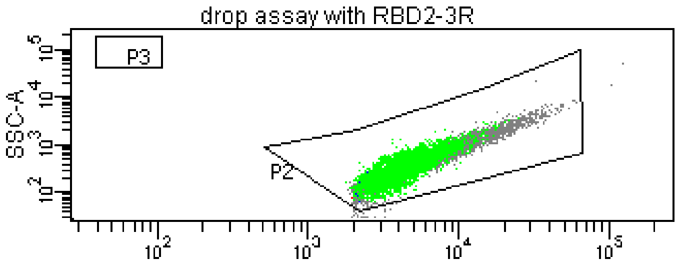

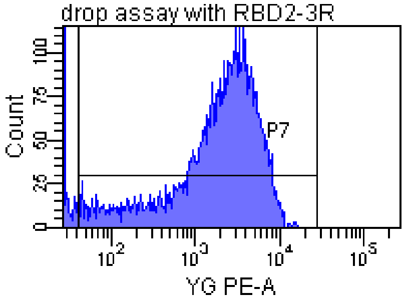

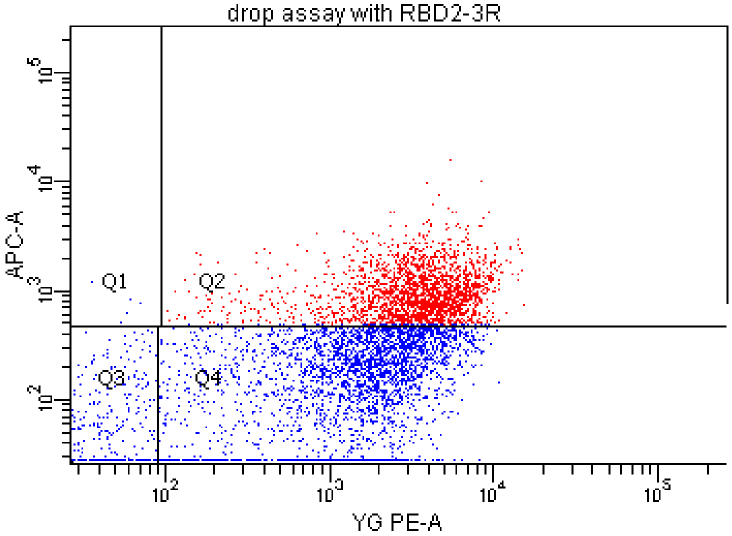

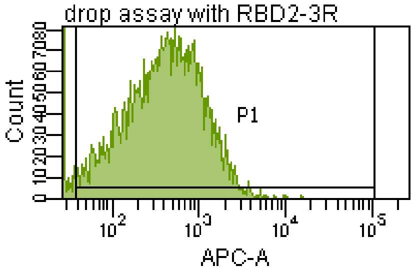

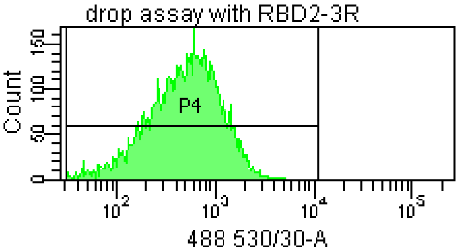

Experiment Name: Bio RBD2 selectons
Specimen Name: drop assay with RBD2
Tube Name: 3R
Record Date: Jun 23, 2020 4:03:10 PM
$OP: Nileena
GUID: 6b60822a-c643-448c-9492-a7ca3da0f05d

Population	Parent...	#Events	%Parent	%Gran...	%Total	488 5... Mean	488 5... Median	PE-A Mean	PE-A Median	APC-A Mean	APC-A Median
All Events	####	####	####	####	####	941	606	997	645	619	287
P2	####	####	####	####	####	930	607	983	647	611	289
P8	P2	7,661	76.6	76.3	76.3	625	502	703	488	410	201
Q1	P8	14	0.2	0.1	0.1	362	339	29	18	1,856	1,005
Q2	P8	2,283	29.8	22.8	22.7	1,022	907	1,371	1,247	1,070	864
Q3	P8	2,223	29.0	22.2	22.2	249	207	30	8	17	7
Q4	P8	3,141	41.0	31.4	31.3	604	536	697	572	202	190
P7	P8	5,655	73.8	56.6	56.3	760	638	943	778	548	366
P1	P8	5,716	74.6	57.2	57.0	742	626	907	743	555	363
P4	P8	7,500	97.9	75.0	74.7	639	512	718	508	418	210

FACSDiva Version 6.1.3

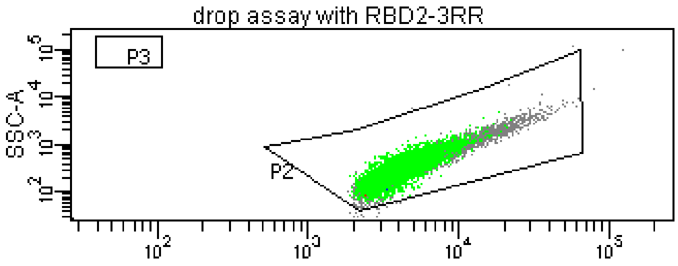

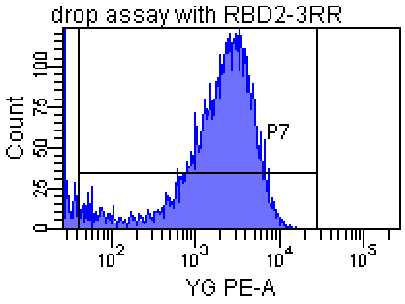

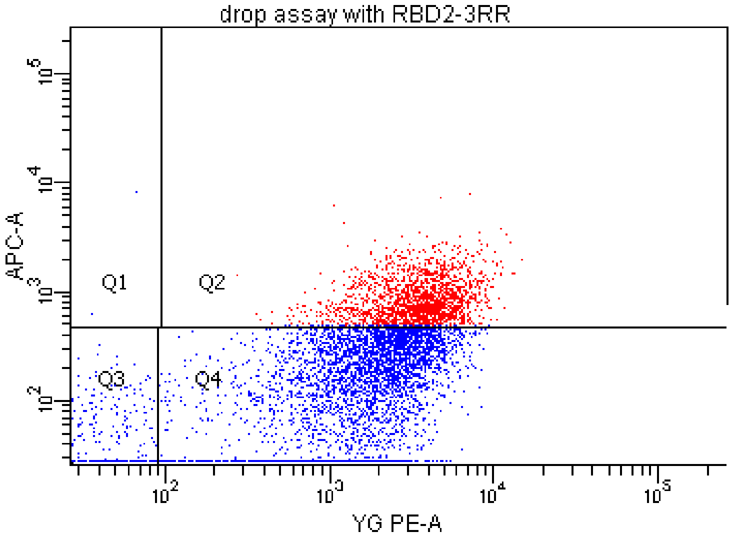

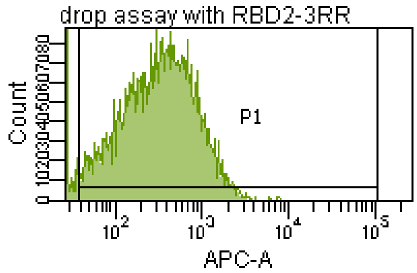

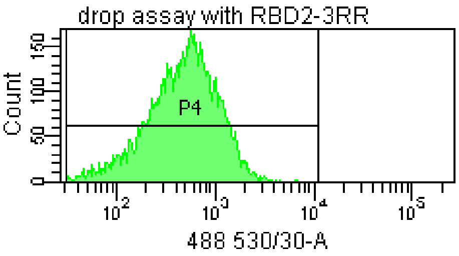

Experiment Name: Bio RBD2 selectons
Specimen Name: drop assay with RBD2
Tube Name: 3RR
Record Date: Jun 23, 2020 4:04:09 PM
$OP: Nileena
GUID: 17f84737-7b29-40fe-859d-e5a4958c4e83

Population	Parent...	#Events	%Parent	%Gran...	%Total	488 5... Mean	488 5... Median	PE-A Mean	PE-A Median	APC-A Mean	APC-A Median
All Events	####	####	####	####	####	863	560	899	600	480	207
P2	####	####	####	####	####	847	561	867	602	466	208
P8	P2	7,915	79.1	78.8	78.8	594	487	654	493	309	155
Q1	P8	14	0.2	0.1	0.1	519	421	63	38	1,752	800
Q2	P8	1,824	23.0	18.2	18.2	1,011	905	1,332	1,214	920	759
Q3	P8	2,285	28.9	22.8	22.8	252	207	29	11	12	3
Q4	P8	3,792	47.9	37.9	37.8	600	530	705	596	189	173
P7	P8	5,824	73.6	58.2	58.0	719	605	880	740	413	281
P1	P8	5,759	72.8	57.6	57.4	703	595	847	725	433	288
P4	P8	7,782	98.3	77.8	77.5	605	495	664	507	314	161

FACSDiva Version 6.1.3

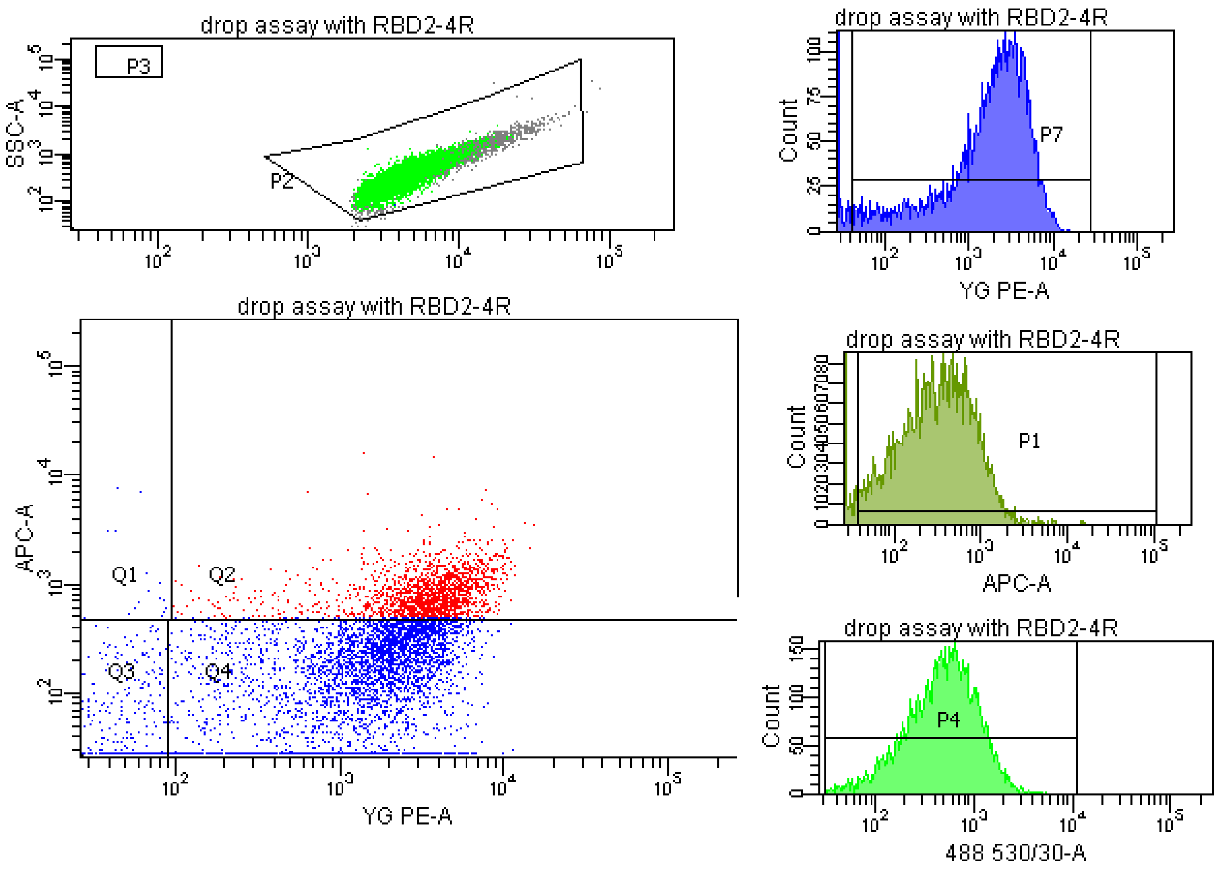

Experiment Name:	Bio RBD2 selectons
Specimen Name:	drop assay with RBD2
Tube Name:	4R
Record Date:	Jun 23, 2020 4:05:12 PM
$OP:	Nileena
GUID:	0fa1e963-796b-4ca3-b78f-1ba74e6aa308

Population	Parent...	#Events	%Parent	%Gran...	%Total	488 5... Mean	488 5... Median	PE-A Mean	PE-A Median	APC-A Mean	APC-A Median
All Events	####	####	####	####	####	905	590	926	610	475	211
P2	####	####	####	####	####	892	591	913	611	464	212
P8	P2	7,705	77.0	76.8	76.8	607	489	652	470	305	154
Q1	P8	21	0.3	0.2	0.2	289	257	44	29	2,006	898
Q2	P8	1,730	22.5	17.3	17.3	1,055	949	1,386	1,275	917	757
Q3	P8	2,112	27.4	21.1	21.1	257	213	28	3	16	7
Q4	P8	3,842	49.9	38.4	38.3	599	524	667	563	179	163
P7	P8	5,800	75.3	58.0	57.8	724	610	858	710	399	262
P1	P8	5,526	71.7	55.3	55.1	716	605	844	711	433	282
P4	P8	7,575	98.3	75.8	75.5	617	497	663	485	310	159

FACSDiva Version 6.1.3

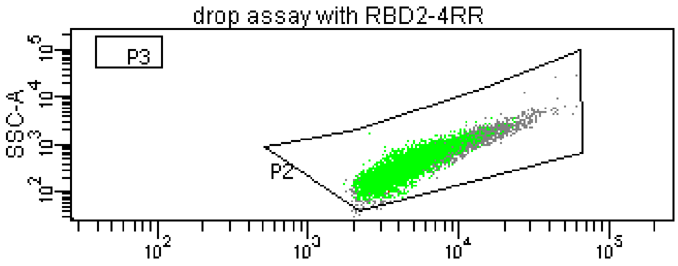

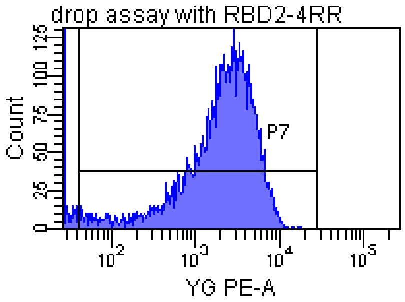

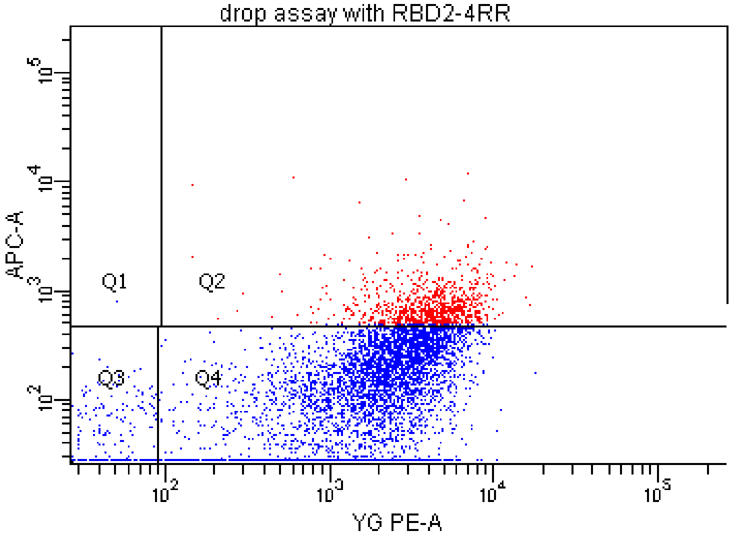

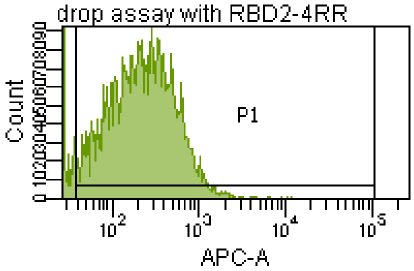

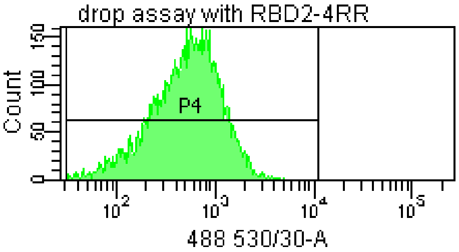

Experiment Name: Bio RBD2 selectons
Specimen Name: drop assay with RBD2
Tube Name: 4RR
Record Date: Jun 23, 2020 4:06:10 PM
$OP: Nileena
GUID: 33a58eed-0953-4dde-afc9-22ca68bb074d

Population	Parent...	#Events	%Parent	%Gran...	%Total	488 5... Mean	488 5... Median	PE-A Mean	PE-A Median	APC-A Mean	APC-A Median
All Events	####	####	####	####	####	914	631	963	691	320	146
P2	####	####	####	####	####	915	633	965	693	320	146
P8	P2	7,674	76.7	76.6	76.6	637	527	703	549	205	109
Q1	P8	8	0.1	0.1	0.1	289	262	34	17	1,572	1,073
Q2	P8	931	12.1	9.3	9.3	1,196	1,058	1,578	1,448	836	642
Q3	P8	1,973	25.7	19.7	19.7	265	216	32	12	10	3
Q4	P8	4,762	62.1	47.6	47.5	682	598	811	701	159	139
P7	P8	5,856	76.3	58.6	58.4	755	642	913	772	263	171
P1	P8	5,257	68.5	52.6	52.4	767	661	931	814	305	201
P4	P8	7,552	98.4	75.5	75.3	647	534	714	567	208	113

FACSDiva Version 6.1.3

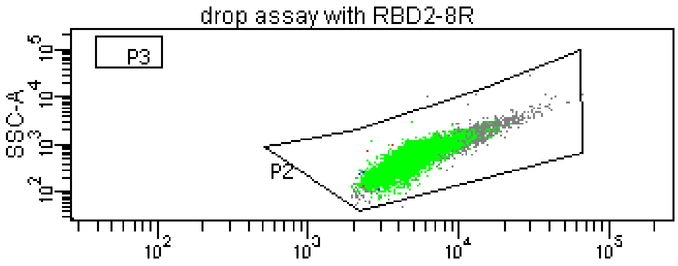

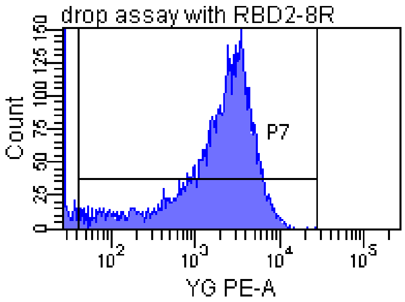

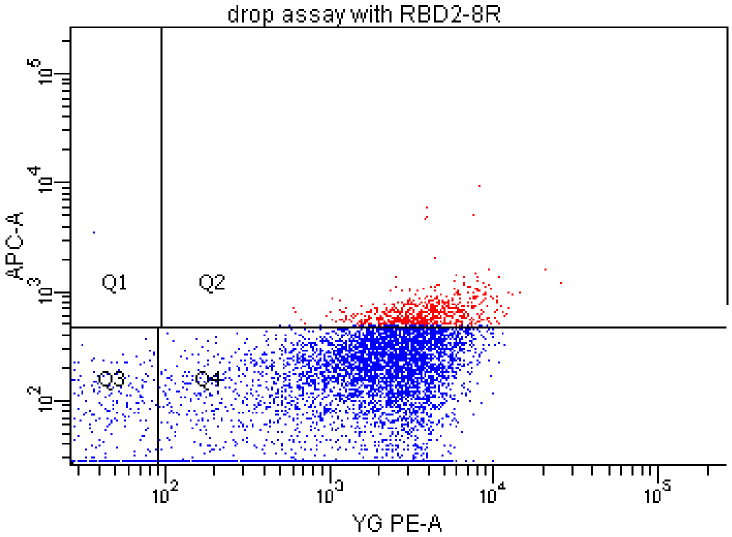

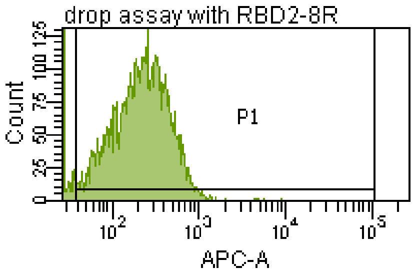

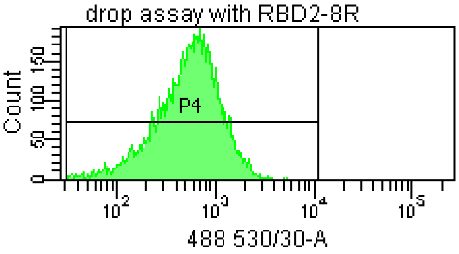

Experiment Name: Bio RBD2 selectons
Specimen Name: drop assay with RBD2
Tube Name: 8R
Record Date: Jun 23, 2020 4:08:09 PM
$OP: Nileena
GUID: 946e59dc-44d4-45c9-9811-f83977c560c0

Population	Parent...	#Events	%Parent	%Gran...	%Total	488 5... Mean	488 5... Median	PE-A Mean	PE-A Median	APC-A Mean	APC-A Median
All Events	####	####	####	####	####	863	644	922	669	283	170
P2	####	####	####	####	####	861	645	920	669	282	169
P8	P2	7,990	79.9	79.9	79.9	641	553	695	552	181	137
Q1	P8	9	0.1	0.1	0.1	198	129	105	58	3,261	2,209
Q2	P8	655	8.2	6.6	6.5	1,272	1,158	1,585	1,367	679	591
Q3	P8	2,083	26.1	20.8	20.8	302	261	47	37	15	6
Q4	P8	5,243	65.6	52.4	52.4	698	631	842	750	179	172
P7	P8	6,120	76.6	61.2	61.2	746	654	894	786	228	188
P1	P8	5,884	73.6	58.8	58.8	732	639	846	733	258	201
P4	P8	7,868	98.5	78.7	78.6	651	560	705	566	183	139

FACSDiva Version 6.1.3

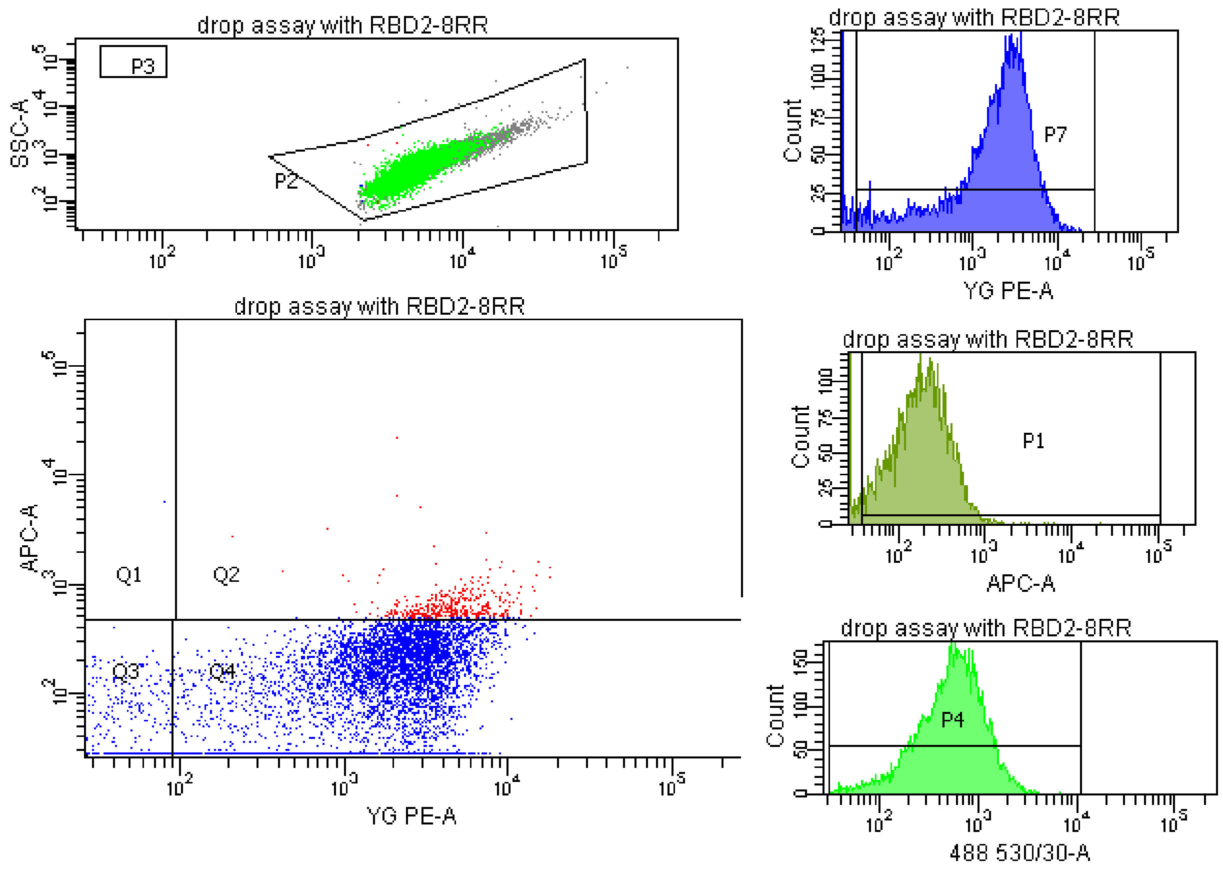

Experiment Name: Bio RBD2 selectons
Specimen Name: drop assay with RBD2
Tube Name: 8RR
Record Date: Jun 23, 2020 4:09:18 PM
$OP: Nileena
GUID: 2749d446-2c53-46c3-8126-c5b49e0dab3b

Population	Parent...	#Events	%Parent	%Gran...	%Total	488 5... Mean	488 5... Median	PE-A Mean	PE-A Median	APC-A Mean	APC-A Median
All Events	####	####	####	####	####	922	660	983	679	314	147
P2	####	####	####	####	####	904	660	970	679	274	147
P8	P2	7,767	77.7	77.6	77.6	649	548	696	543	161	116
Q1	P8	15	0.2	0.2	0.1	207	137	66	29	3,087	2,503
Q2	P8	393	5.1	3.9	3.9	1,522	1,388	1,866	1,662	731	568
Q3	P8	2,002	25.8	20.0	20.0	299	252	50	40	20	12
Q4	P8	5,357	69.0	53.6	53.5	717	643	854	755	163	150
P7	P8	5,980	77.0	59.8	59.7	755	655	891	767	196	156
P1	P8	5,633	72.5	56.3	56.3	752	649	858	727	228	172
P4	P8	7,631	98.2	76.3	76.2	661	557	708	562	163	119

FACSDiva Version 6.1.3

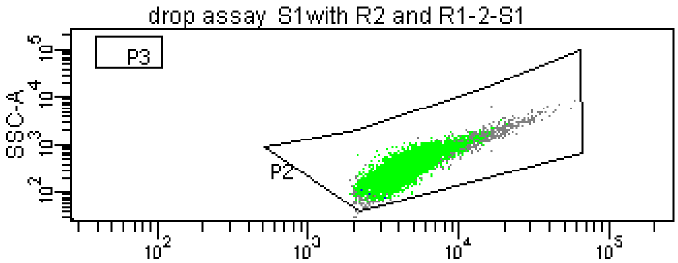

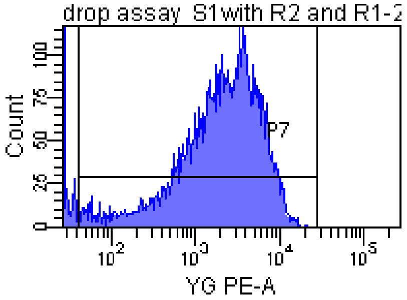

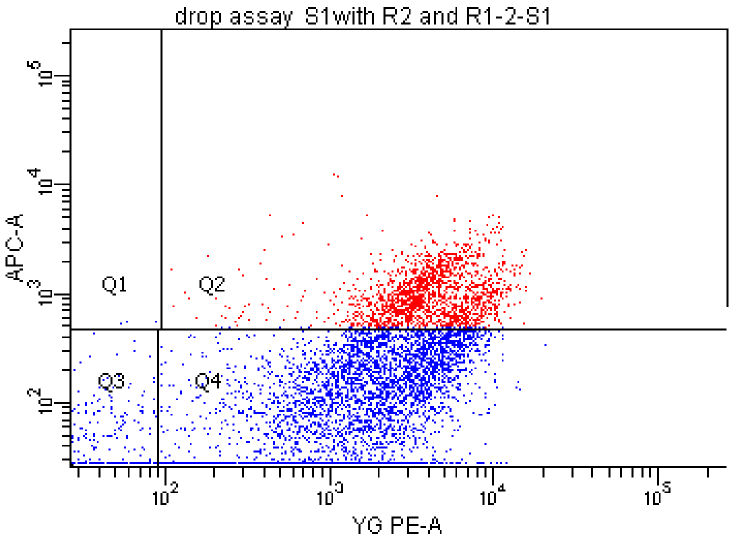

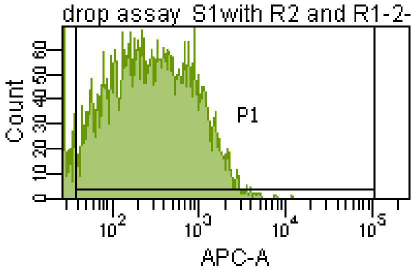

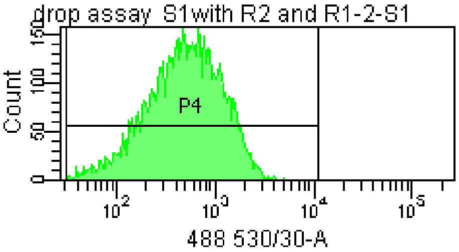

Experiment Name: Bio RBD2 selectons
Specimen Name: drop assay S1with R2 and R1
Tube Name: 2-S1
Record Date: Jun 23, 2020 6:57:56 PM
$OP: Nileena
GUID: 56dfb3fc-f465-46bc-afc4-e4f67cac53eb

Population	Parent...	#Events	%Parent	%Gran...	%Total	488 5... Mean	488 5... Median	PE-A Mean	PE-A Median	APC-A Mean	APC-A Median
All Events	####	####	####	####	####	809	548	971	588	405	120
P2	####	####	####	####	####	810	549	972	589	405	121
P8	P2	8,590	85.9	85.8	85.8	645	489	796	504	305	97
Q1	P8	10	0.1	0.1	0.1	314	185	74	45	1,968	919
Q2	P8	1,781	20.7	17.8	17.8	1,153	1,039	1,735	1,483	1,098	877
Q3	P8	2,341	27.3	23.4	23.4	232	192	28	22	9	3
Q4	P8	4,458	51.9	44.6	44.5	660	542	825	608	141	108
P7	P8	6,402	74.5	64.0	63.9	787	645	1,059	798	404	190
P1	P8	5,608	65.3	56.1	56.0	816	685	1,111	907	473	246
P4	P8	8,492	98.9	84.9	84.8	652	496	805	512	309	100

FACSDiva Version 6.1.3

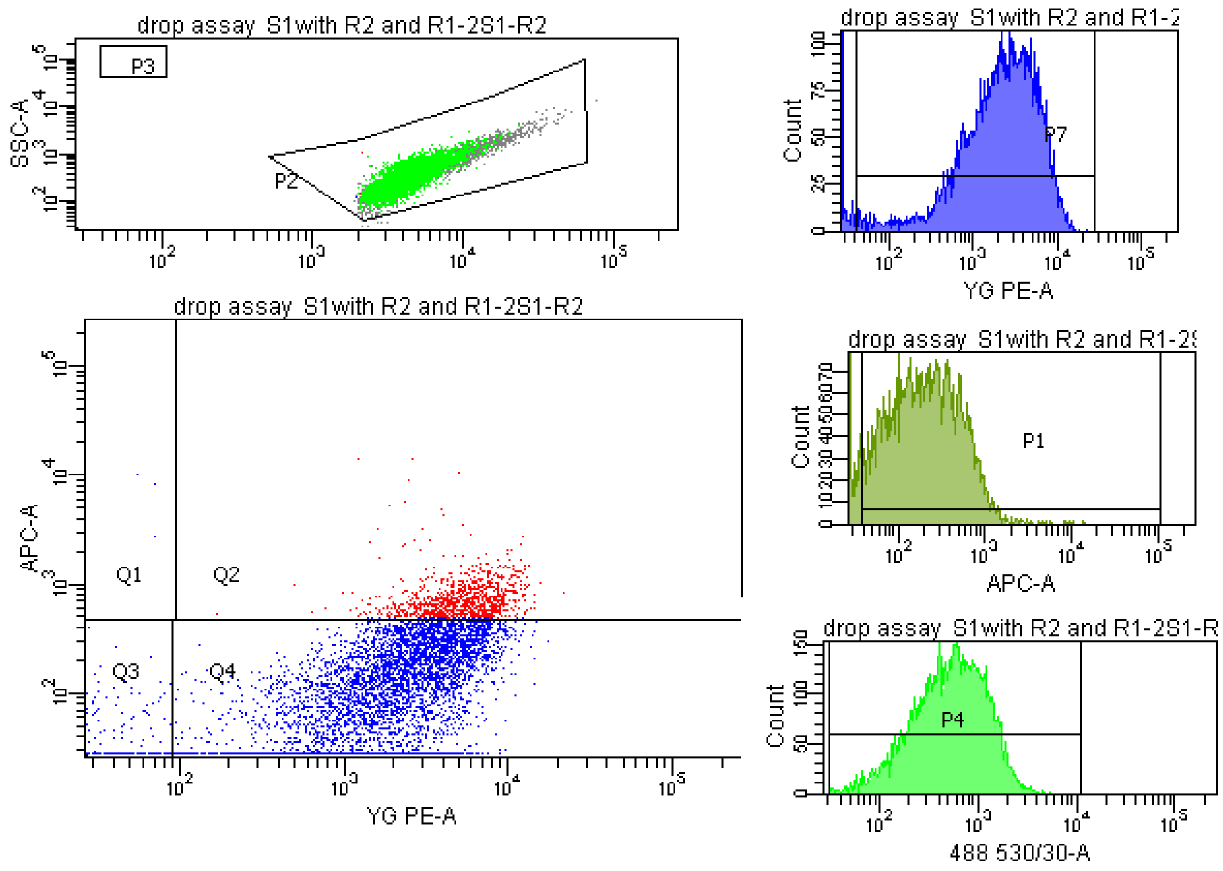

Experiment Name: Bio RBD2 selectons
Specimen Name: drop assay S1with R2 and R1
Tube Name: 2S1-R2
Record Date: Jun 23, 2020 7:00:07 PM
$OP: Nileena
GUID: 0aa0bbb9-b555-4445-9f26-212c2cb7a55a

Population	Parent...	#Events	%Parent	%Gran...	%Total	488 5... Mean	488 5... Median	PE-A Mean	PE-A Median	APC-A Mean	APC-A Median
All Events	####	####	####	####	####	866	609	1,027	657	266	101
P2	####	####	####	####	####	866	610	1,026	661	266	102
P8	P2	8,343	83.4	83.3	83.3	673	533	833	556	208	81
Q1	P8	12	0.1	0.1	0.1	309	256	39	25	17,541	3,896
Q2	P8	970	11.6	9.7	9.7	1,375	1,268	2,101	1,985	810	657
Q3	P8	2,043	24.5	20.4	20.4	238	199	27	21	9	3
Q4	P8	5,318	63.7	53.2	53.1	712	604	913	712	135	104
P7	P8	6,407	76.8	64.1	63.9	805	668	1,077	823	238	135
P1	P8	5,317	63.7	53.2	53.1	859	746	1,171	968	300	184
P4	P8	8,246	98.8	82.5	82.3	681	540	843	567	210	83

FACSDiva Version 6.1.3

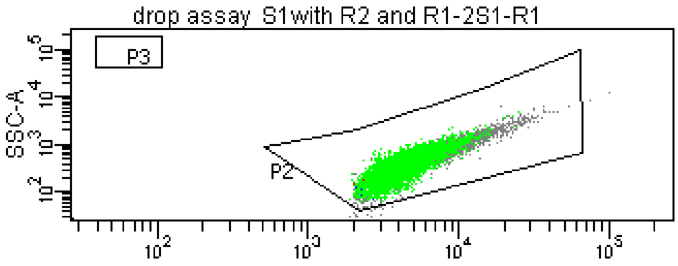

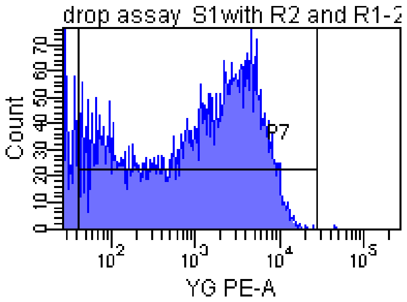

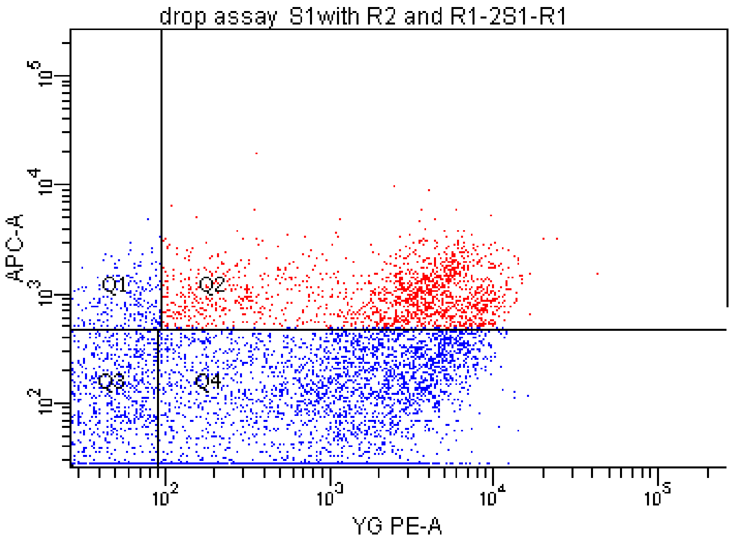

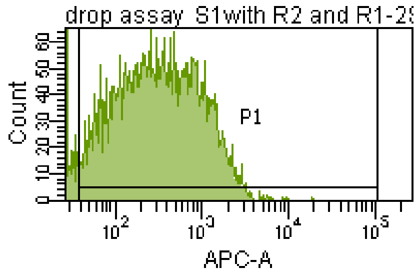

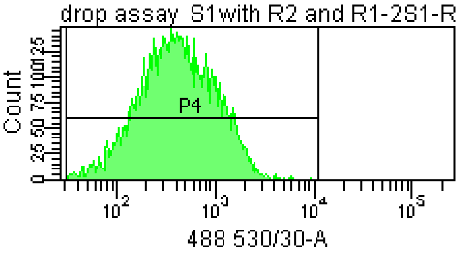

Experiment Name: Bio RBD2 selectons
Specimen Name: drop assay S1with R2 and R1
Tube Name: 2S1-R1
Record Date: Jun 23, 2020 7:01:41 PM
$OP: Nileena
GUID: fcc97e18-e2de-4673-81d5-cc231c0bb83a

Population	Parent...	#Events	%Parent	%Gran...	%Total	488 5... Mean	488 5... Median	PE-A Mean	PE-A Median	APC-A Mean	APC-A Median
All Events	####	####	####	####	####	716	443	702	181	404	121
P2	####	####	####	####	####	712	444	696	182	403	121
P8	P2	8,521	85.2	85.0	85.0	563	395	558	131	315	99
Q1	P8	237	2.8	2.4	2.4	353	302	52	44	998	786
Q2	P8	1,577	18.5	15.8	15.7	1,057	941	1,406	1,237	1,141	921
Q3	P8	3,434	40.3	34.3	34.3	255	215	32	25	44	19
Q4	P8	3,273	38.4	32.7	32.6	663	536	738	493	152	123
P7	P8	5,564	65.3	55.6	55.5	730	566	838	496	454	225
P1	P8	5,628	66.0	56.3	56.1	697	522	774	372	481	248
P4	P8	8,383	98.4	83.8	83.6	572	400	567	140	319	104

FACSDiva Version 6.1.3

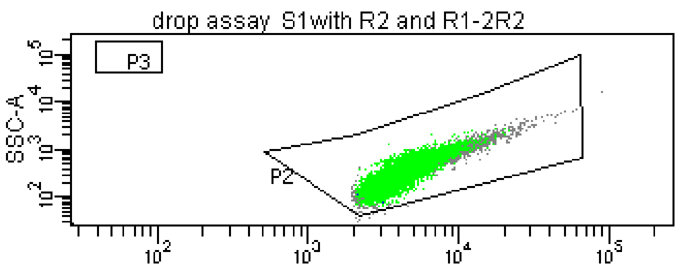

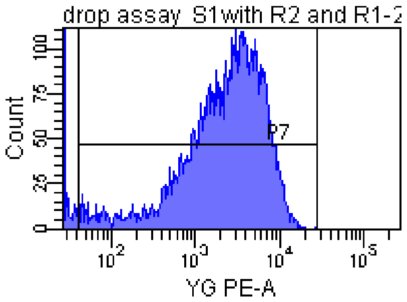

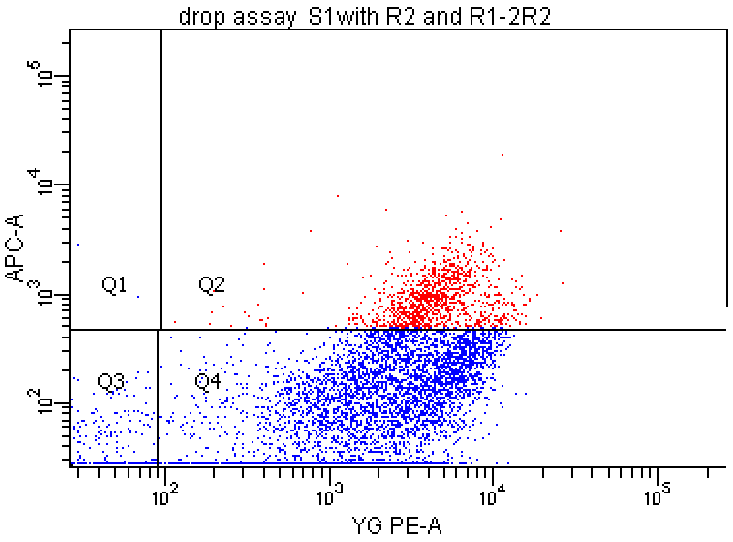

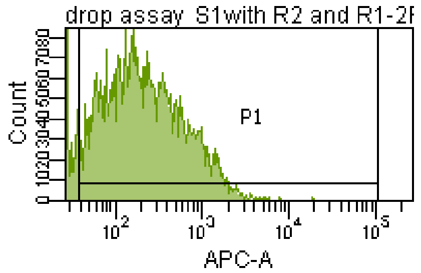

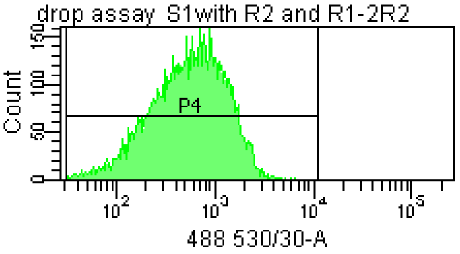

Experiment Name: Bio RBD2 selectons
Specimen Name: drop assay S1with R2 and R1
Tube Name: 2R2
Record Date: Jun 23, 2020 7:02:57 PM
$OP: Nileena
GUID: 31888217-0f08-48c1-9355-71d27fd8e8b3

Population	Parent...	#Events	%Parent	%Gran...	%Total	488 5... Mean	488 5... Median	PE-A Mean	PE-A Median	APC-A Mean	APC-A Median
All Events	####	####	####	####	####	883	631	1,094	707	317	97
P2	####	####	####	####	####	881	632	1,094	708	291	98
P8	P2	8,414	84.1	84.0	84.0	705	553	900	598	223	80
Q1	P8	6	0.1	0.1	0.1	562	378	90	52	2,662	2,717
Q2	P8	1,162	13.8	11.6	11.6	1,265	1,127	1,867	1,597	1,019	824
Q3	P8	2,134	25.4	21.3	21.3	244	201	30	22	12	7
Q4	P8	5,112	60.8	51.1	51.0	771	640	1,044	779	128	98
P7	P8	6,447	76.6	64.5	64.4	848	711	1,165	924	286	131
P1	P8	5,414	64.3	54.1	54.0	893	773	1,240	1,041	351	172
P4	P8	8,320	98.9	83.2	83.1	713	560	910	609	226	82

FACSDiva Version 6.1.3

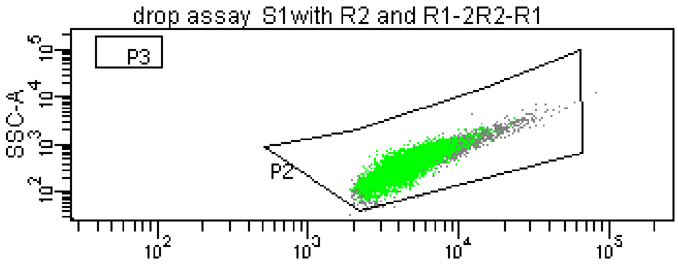

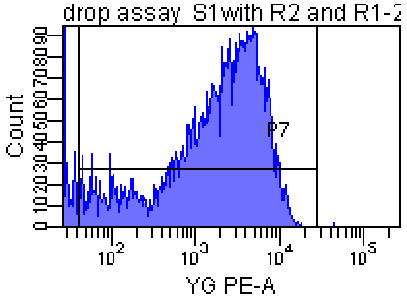

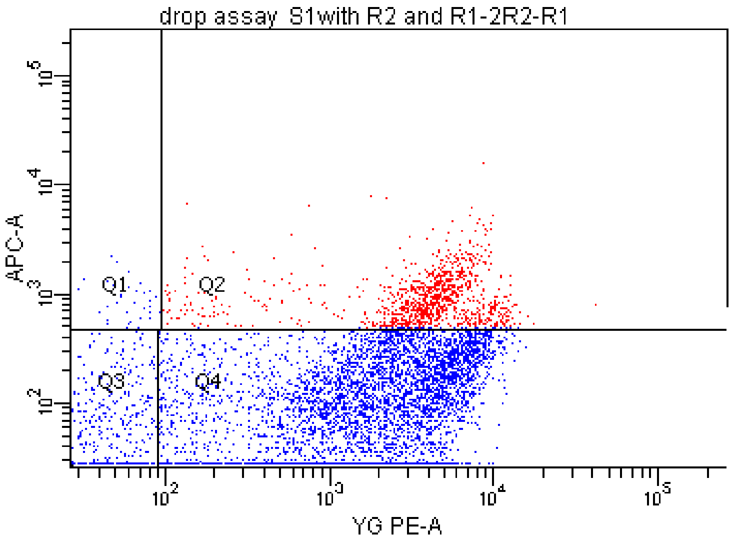

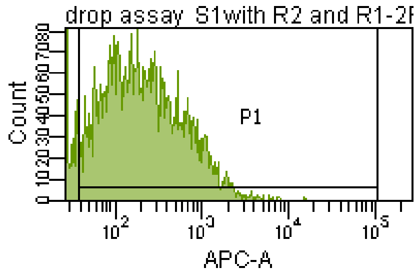

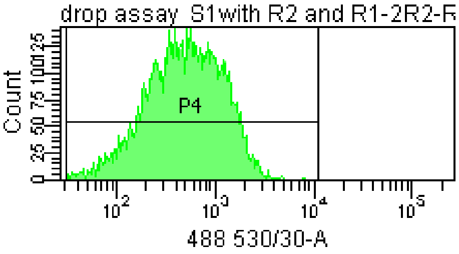

Experiment Name:	Bio RBD2 selectons
Specimen Name:	drop assay S1with R2 and R1
Tube Name:	2R2-R1
Record Date:	Jun 23, 2020 7:04:22 PM
$OP:	Nileena
GUID:	d687217e-02a1-451d-a5ca-be487dedfae5

Population	Parent...	#Events	%Parent	%Gran...	%Total	488 5... Mean	488 5... Median	PE-A Mean	PE-A Median	APC-A Mean	APC-A Median
All Events	####	####	####	####	####	840	561	991	545	289	95
P2	####	####	####	####	####	839	562	989	546	288	96
P8	P2	8,364	83.6	83.5	83.5	656	487	788	432	218	79
Q1	P8	41	0.5	0.4	0.4	412	376	71	49	1,393	884
Q2	P8	1,076	12.9	10.8	10.7	1,208	1,104	1,738	1,545	1,025	794
Q3	P8	2,523	30.2	25.2	25.2	247	210	30	23	21	9
Q4	P8	4,724	56.5	47.2	47.2	751	614	984	715	129	99
P7	P8	6,126	73.2	61.3	61.2	805	657	1,065	794	287	131
P1	P8	5,332	63.7	53.3	53.2	829	692	1,096	860	346	170
P4	P8	8,265	98.8	82.6	82.5	664	495	798	446	221	81

FACSDiva Version 6.1.3

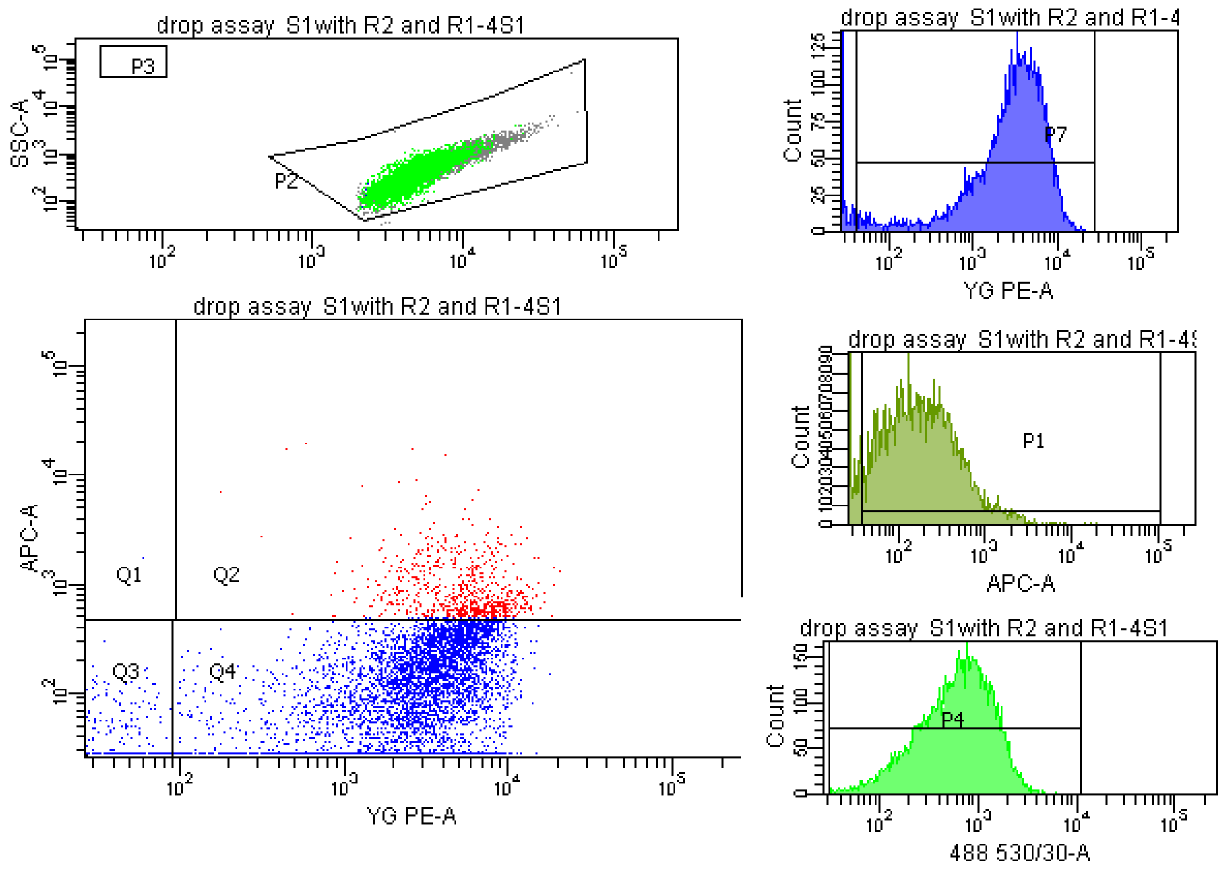

Experiment Name: Bio RBD2 selectons
Specimen Name: drop assay S1with R2 and R1
Tube Name: 4S1
Record Date: Jun 23, 2020 7:06:00 PM
$OP: Nileena
GUID: d0a32aff-e7fb-4ac7-a531-5b2380cadd7a

Population	Parent...	#Events	%Parent	%Gran...	%Total	488 5... Mean	488 5... Median	PE-A Mean	PE-A Median	APC-A Mean	APC-A Median
All Events	####	####	####	####	####	1,000	740	1,282	974	290	90
P2	####	####	####	####	####	1,001	741	1,283	977	290	90
P8	P2	8,171	81.7	81.6	81.6	771	630	1,018	809	190	71
Q1	P8	14	0.2	0.1	0.1	308	317	71	27	2,126	1,013
Q2	P8	742	9.1	7.4	7.4	1,438	1,297	2,215	2,072	1,128	729
Q3	P8	2,125	26.0	21.2	21.2	259	216	29	23	11	4
Q4	P8	5,290	64.7	52.9	52.8	885	770	1,250	1,091	126	94
P7	P8	6,163	75.4	61.6	61.5	939	806	1,341	1,149	244	113
P1	P8	5,058	61.9	50.6	50.5	964	846	1,364	1,214	313	158
P4	P8	8,093	99.0	80.9	80.8	779	635	1,028	821	192	72

FACSDiva Version 6.1.3

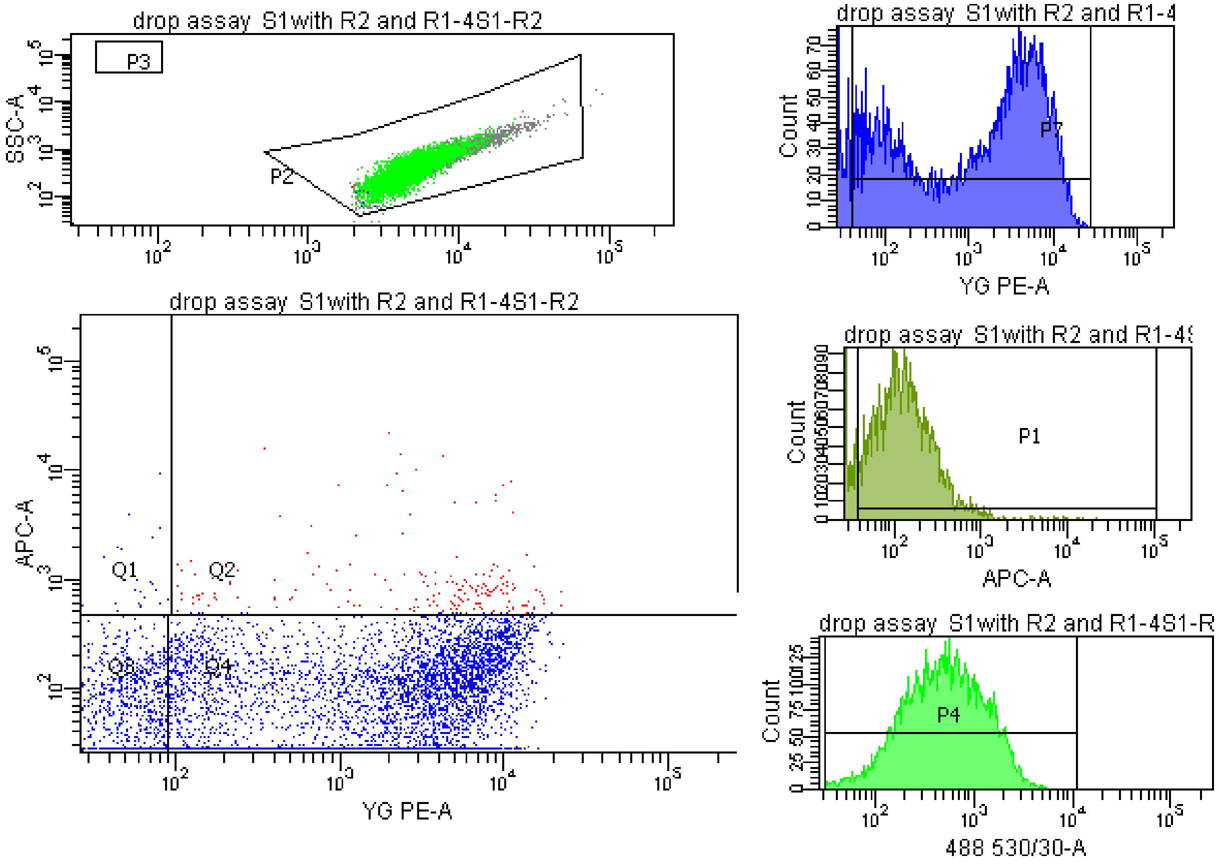

Experiment Name: Bio RBD2 selectons
Specimen Name: drop assay S1with R2 and R1
Tube Name: 4S1-R2
Record Date: Jun 23, 2020 7:09:30 PM
$OP: Nileena
GUID: 5125d435-2ebb-44ba-8b93-988ed6d3ac3c

Population	Parent...	#Events	%Parent	%Gran...	%Total	488 5... Mean	488 5... Median	PE-A Mean	PE-A Median	APC-A Mean	APC-A Median
All Events	####	####	####	####	####	916	573	1,062	289	183	62
P2	####	####	####	####	####	909	573	1,052	290	182	62
P8	P2	8,279	82.8	82.6	82.6	698	484	820	159	112	50
Q1	P8	39	0.5	0.4	0.4	369	358	51	49	2,144	878
Q2	P8	184	2.2	1.8	1.8	1,445	1,239	2,002	1,753	1,535	753
Q3	P8	3,276	39.6	32.8	32.7	280	228	35	28	25	13
Q4	P8	4,780	57.7	47.8	47.7	959	778	1,318	989	101	81
P7	P8	5,725	69.2	57.2	57.1	892	690	1,173	751	147	80
P1	P8	4,562	55.1	45.6	45.5	910	699	1,191	679	211	114
P4	P8	8,171	98.7	81.7	81.5	707	492	830	171	113	51

FACSDiva Version 6.1.3

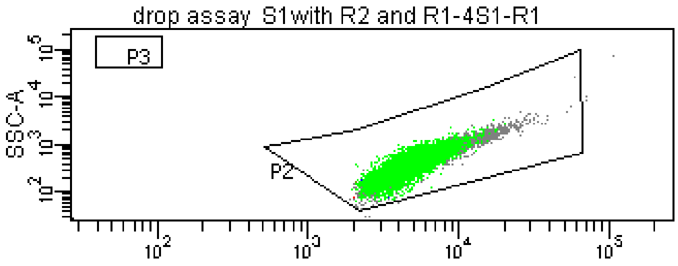

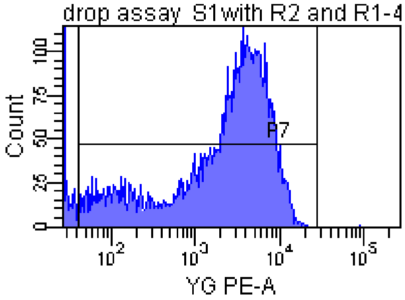

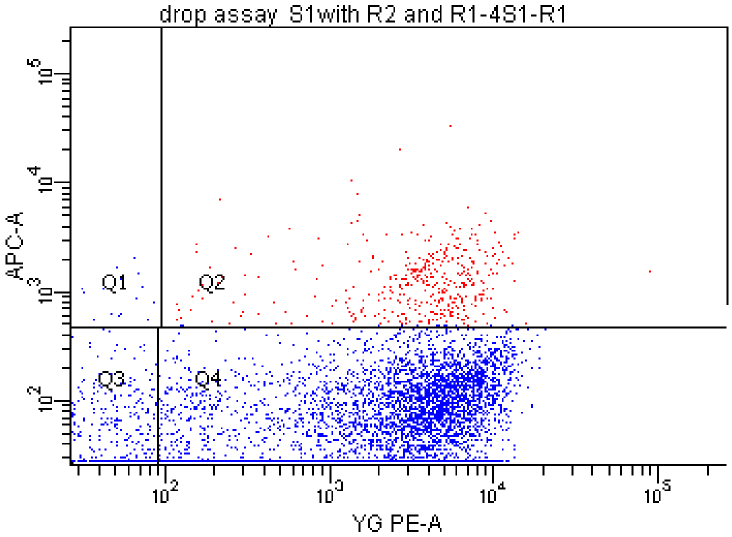

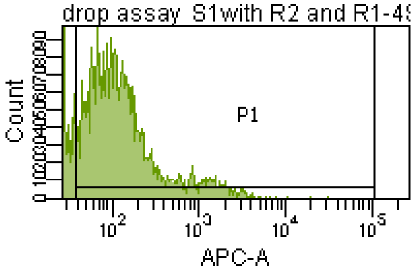

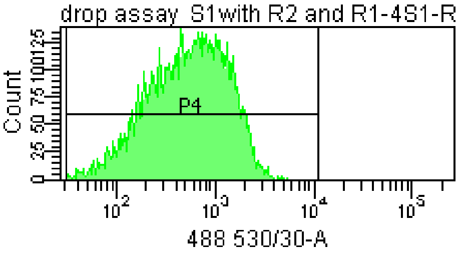

Experiment Name: Bio RBD2 selectons
Specimen Name: drop assay S1with R2 and R1
Tube Name: 4S1-R1
Record Date: Jun 23, 2020 7:10:47 PM
$OP: Nileena
GUID: 8044d529-f99c-4358-93cf-7db7b249e5ed

Population	Parent...	#Events	%Parent	%Gran...	%Total	488 5... Mean	488 5... Median	PE-A Mean	PE-A Median	APC-A Mean	APC-A Median
All Events	####	####	####	####	####	912	628	1,169	715	207	48
P2	####	####	####	####	####	903	629	1,158	716	176	48
P8	P2	8,589	85.9	85.7	85.7	721	543	935	538	128	40
Q1	P8	25	0.3	0.2	0.2	249	243	41	28	1,136	927
Q2	P8	422	4.9	4.2	4.2	1,150	1,037	1,744	1,549	1,539	1,158
Q3	P8	2,715	31.6	27.2	27.1	252	208	29	24	14	6
Q4	P8	5,427	63.2	54.3	54.2	925	774	1,329	1,101	70	55
P7	P8	6,208	72.3	62.1	62.0	901	757	1,279	1,062	169	59
P1	P8	4,364	50.8	43.6	43.6	945	812	1,340	1,135	260	103
P4	P8	8,483	98.8	84.8	84.7	728	553	943	565	129	41

FACSDiva Version 6.1.3

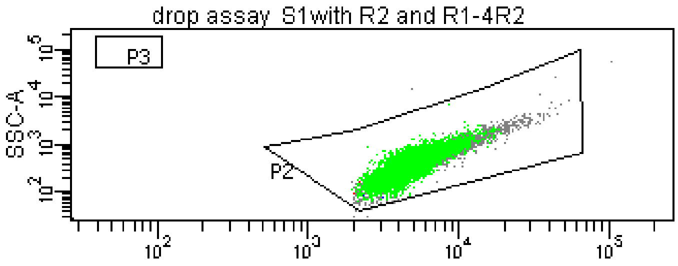

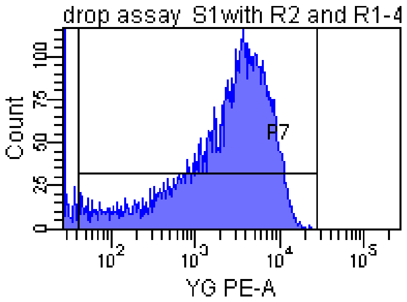

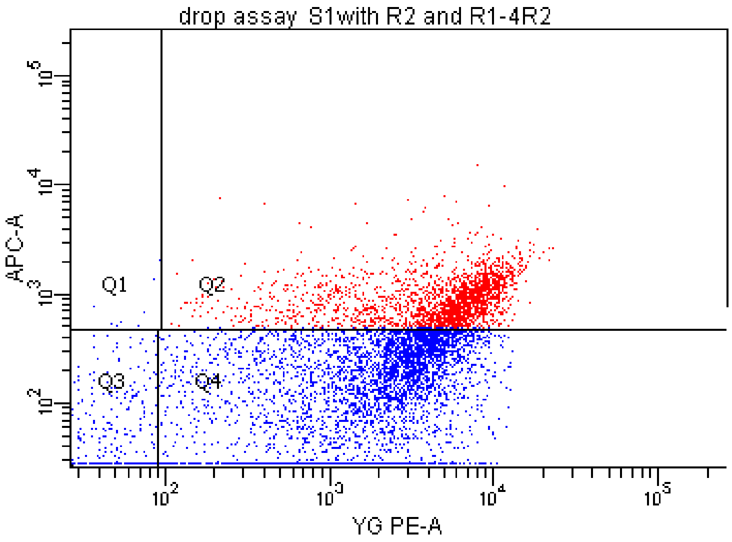

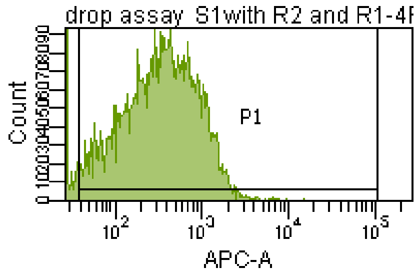

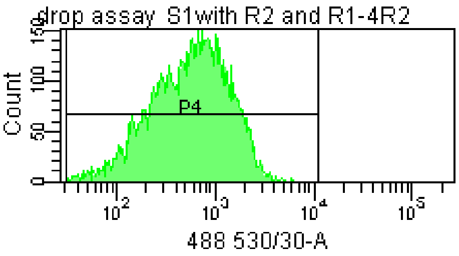

Experiment Name: Bio RBD2 selectons
Specimen Name: drop assay S1with R2 and R1
Tube Name: 4R2
Record Date: Jun 23, 2020 7:12:44 PM
$OP: Nileena
GUID: 09f1cd85-4553-451a-8ac1-83a0f5c3fed8

Population	Parent...	#Events	%Parent	%Gran...	%Total	488 5... Mean	488 5... Median	PE-A Mean	PE-A Median	APC-A Mean	APC-A Median
All Events	####	####	####	####	####	960	663	1,222	768	461	204
P2	####	####	####	####	####	954	663	1,215	768	457	204
P8	P2	8,573	85.7	85.6	85.6	754	582	988	625	340	168
Q1	P8	16	0.2	0.2	0.2	333	250	47	50	1,536	876
Q2	P8	2,126	24.8	21.3	21.2	1,362	1,266	2,105	2,073	981	809
Q3	P8	2,351	27.4	23.5	23.5	253	211	28	25	15	5
Q4	P8	4,080	47.6	40.8	40.7	727	641	962	844	189	174
P7	P8	6,442	75.1	64.4	64.3	922	763	1,306	1,067	446	290
P1	P8	6,223	72.6	62.2	62.1	916	763	1,293	1,074	472	310
P4	P8	8,497	99.1	85.0	84.9	760	588	996	644	343	171

FACSDiva Version 6.1.3

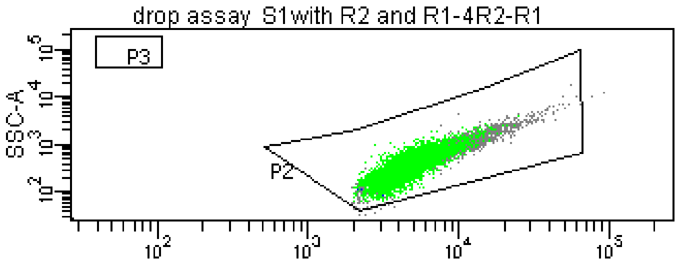

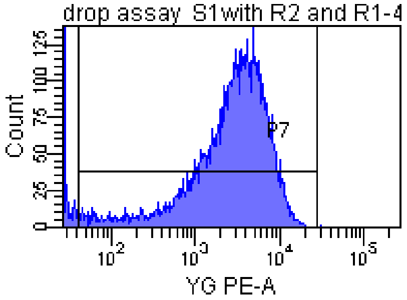

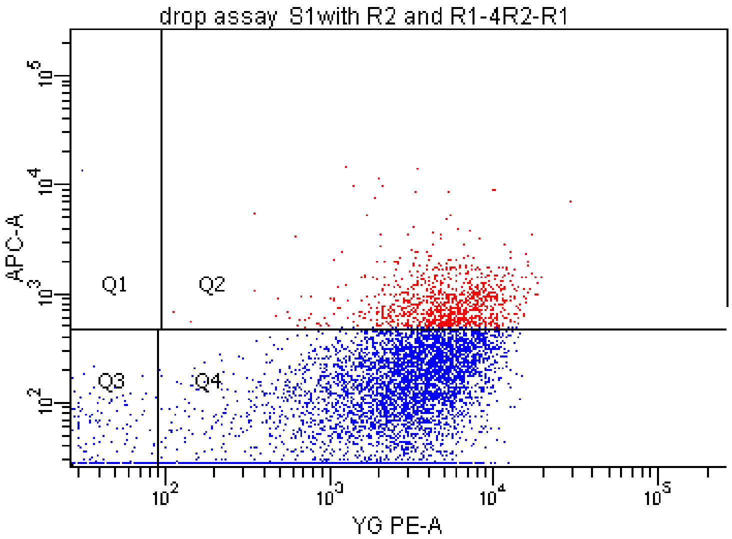

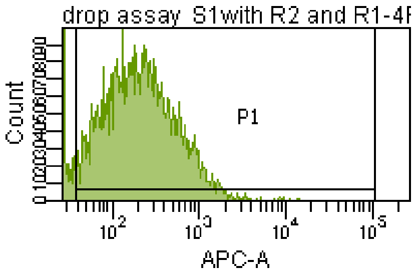

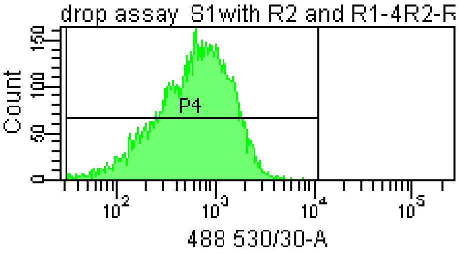

Experiment Name: Bio RBD2 selectons
Specimen Name: drop assay S1with R2 and R1
Tube Name: 4R2-R1
Record Date: Jun 23, 2020 7:13:54 PM
$OP: Nileena
GUID: 6103f755-116b-4edb-94f9-fc726162dd93

Population	Parent...	#Events	%Parent	%Gran...	%Total	488 5... Mean	488 5... Median	PE-A Mean	PE-A Median	APC-A Mean	APC-A Median
All Events	####	####	####	####	####	1,033	743	1,323	957	317	124
P2	####	####	####	####	####	1,031	745	1,320	959	315	124
P8	P2	8,225	82.2	82.1	82.1	784	633	1,035	808	220	98
Q1	P8	9	0.1	0.1	0.1	317	217	35	31	2,319	939
Q2	P8	1,013	12.3	10.1	10.1	1,450	1,306	2,176	1,939	993	741
Q3	P8	2,030	24.7	20.3	20.3	253	214	32	23	13	6
Q4	P8	5,173	62.9	51.7	51.6	863	746	1,207	1,017	146	121
P7	P8	6,315	76.8	63.1	63.0	944	802	1,338	1,123	278	152
P1	P8	5,644	68.6	56.4	56.3	966	835	1,370	1,188	324	180
P4	P8	8,138	98.9	81.4	81.2	792	641	1,046	820	222	100

FACSDiva Version 6.1.3

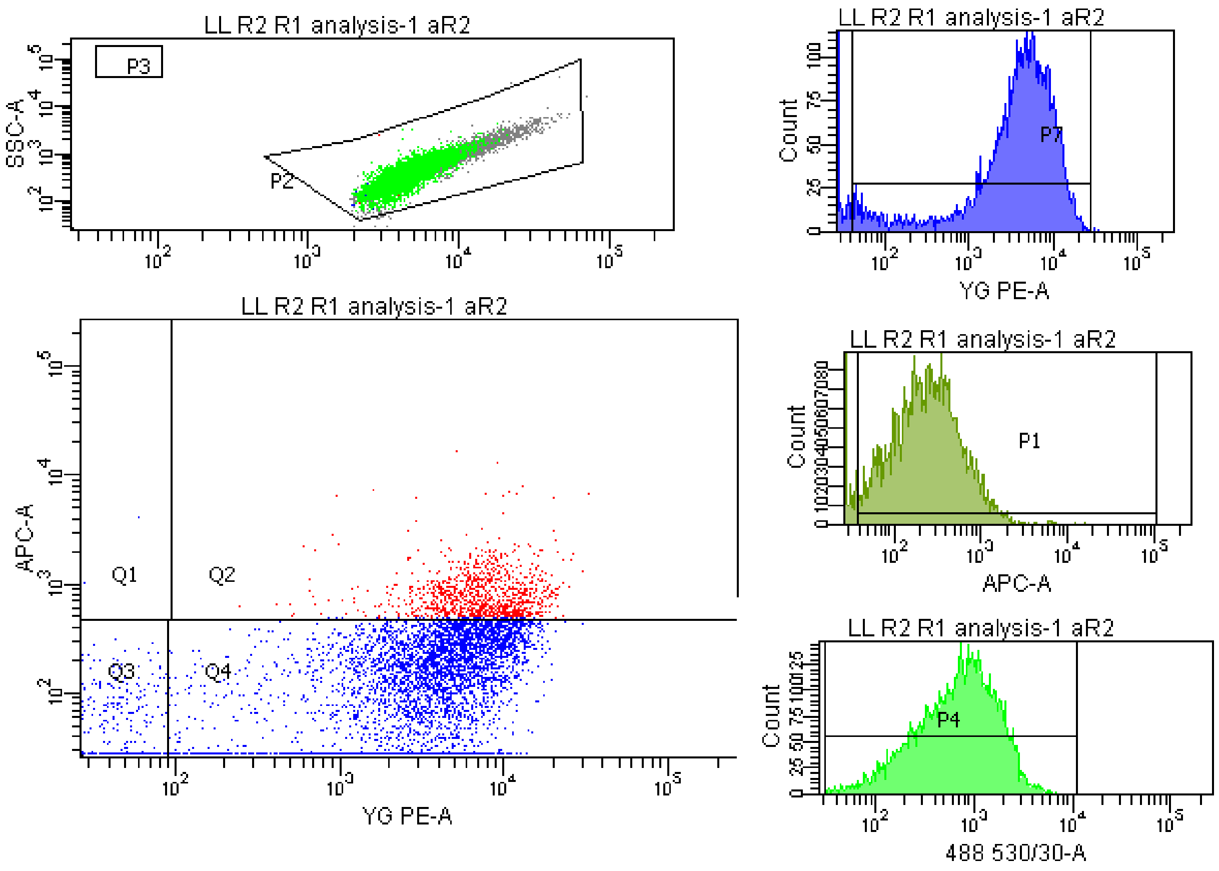

Experiment Name: Bio RBD2 selectons
Specimen Name: LL R2 R1 analysis
Tube Name: 1 aR2
Record Date: Jun 24, 2020 6:01:10 PM
$OP: Nileena
GUID: 3853b3d8-c920-44bb-9e2f-45eb6d8e6951

Population	Parent...	#Events	%Parent	%Gran...	%Total	488 5... Mean	488 5... Median	PE-A Mean	PE-A Median	APC-A Mean	APC-A Median
All Events	####	####	####	####	####	1,333	889	1,873	1,275	405	155
P2	####	####	####	####	####	1,316	891	1,870	1,275	377	155
P8	P2	7,753	77.5	77.4	77.4	911	717	1,338	1,003	222	115
Q1	P8	15	0.2	0.2	0.1	269	194	102	74	2,860	1,541
Q2	P8	1,009	13.0	10.1	10.1	1,735	1,561	2,921	2,563	929	726
Q3	P8	2,165	27.9	21.6	21.6	270	227	33	22	11	3
Q4	P8	4,564	58.9	45.6	45.6	1,035	879	1,610	1,340	157	143
P7	P8	5,809	74.9	58.1	58.0	1,122	935	1,767	1,468	284	177
P1	P8	5,306	68.4	53.1	53.0	1,115	932	1,746	1,479	339	207
P4	P8	7,651	98.7	76.5	76.4	923	726	1,355	1,025	224	118

FACSDiva Version 6.1.3

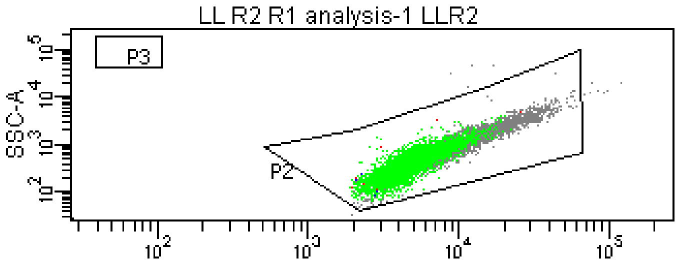

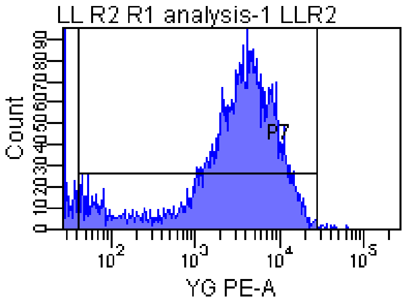

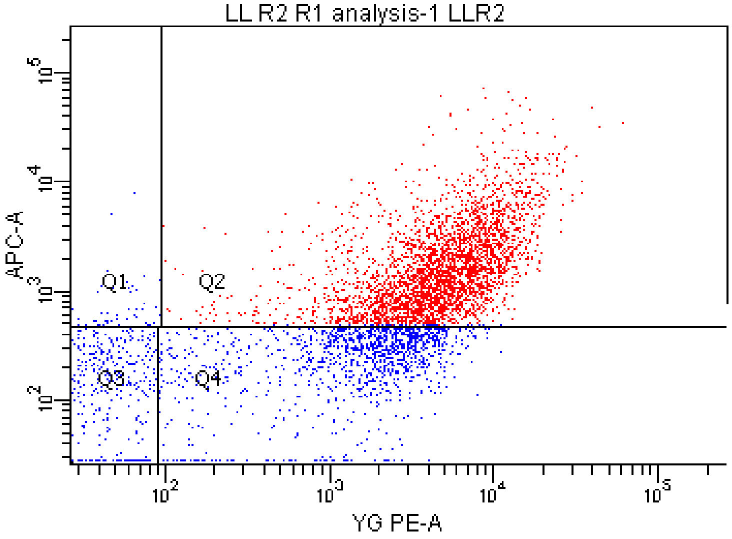

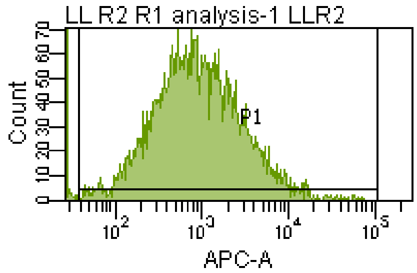

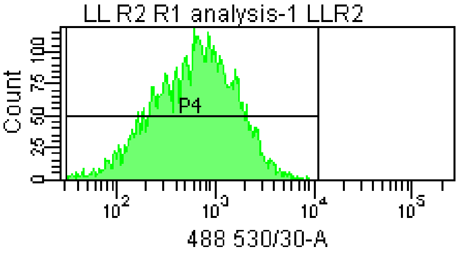

Experiment Name: Bio RBD2 selectons
Specimen Name: LL R2 R1 analysis
Tube Name: 1 LLR2
Record Date: Jun 24, 2020 6:02:38 PM
$OP: Nileena
GUID: fa5f846b-906e-47a9-8660-f5ca1a31795a

Population	Parent...	#Events	%Parent	%Gran...	%Total	488 5... Mean	488 5... Median	PE-A Mean	PE-A Median	APC-A Mean	APC-A Median
All Events	####	####	####	####	####	1,721	946	2,336	1,246	4,048	886
P2	####	####	####	####	####	1,694	946	2,303	1,245	3,931	885
P8	P2	6,748	67.5	67.3	67.3	872	632	1,169	722	1,374	493
Q1	P8	194	2.9	1.9	1.9	376	329	64	41	841	616
Q2	P8	3,250	48.2	32.5	32.4	1,367	1,129	2,110	1,703	2,610	1,364
Q3	P8	2,015	29.9	20.2	20.1	278	232	33	21	140	131
Q4	P8	1,289	19.1	12.9	12.9	627	580	738	629	265	278
P7	P8	4,774	70.7	47.7	47.6	1,097	875	1,605	1,241	1,814	821
P1	P8	6,178	91.6	61.8	61.7	924	686	1,270	857	1,503	565
P4	P8	6,651	98.6	66.5	66.4	883	645	1,182	742	1,386	504

FACSDiva Version 6.1.3

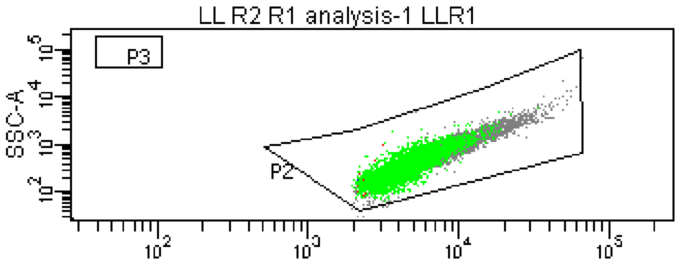

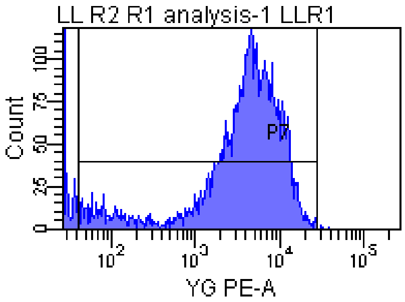

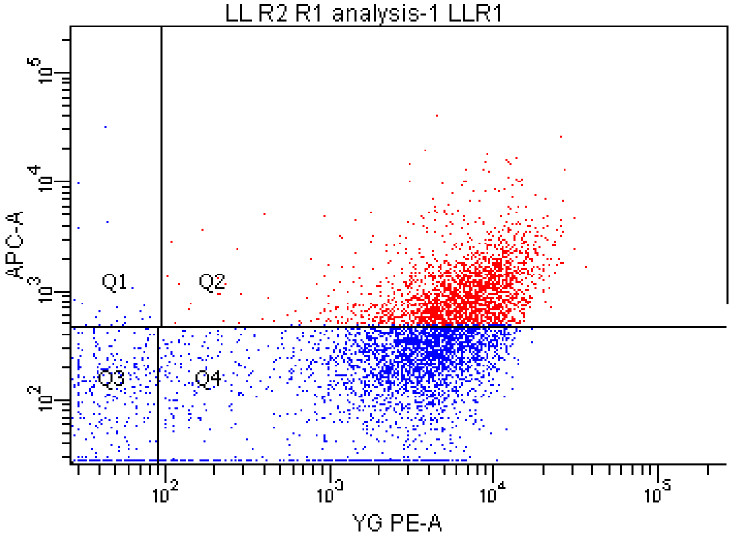

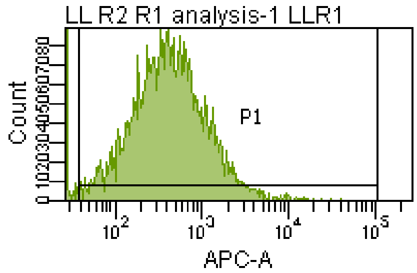

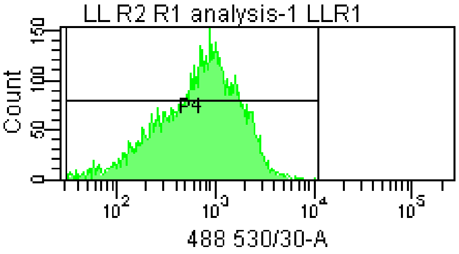

Experiment Name:	Bio RBD2 selectons
Specimen Name:	LL R2 R1 analysis
Tube Name:	1 LLR1
Record Date:	Jun 24, 2020 6:03:48 PM
$OP:	Nileena
GUID:	1814e451-cbec-4dc3-9375-15f55ece4dbd

Population	Parent...	#Events	%Parent	%Gran...	%Total	488 5... Mean	488 5... Median	PE-A Mean	PE-A Median	APC-A Mean	APC-A Median
All Events	####	####	####	####	####	1,541	973	2,197	1,436	1,031	397
P2	####	####	####	####	####	1,543	974	2,195	1,440	1,018	398
P8	P2	7,269	72.7	72.6	72.6	939	737	1,380	1,042	540	275
Q1	P8	101	1.4	1.0	1.0	417	307	66	37	1,335	640
Q2	P8	2,193	30.2	21.9	21.9	1,621	1,455	2,704	2,417	1,327	859
Q3	P8	1,984	27.3	19.8	19.8	275	233	31	20	107	89
Q4	P8	2,991	41.1	29.9	29.9	897	798	1,348	1,180	223	224
P7	P8	5,395	74.2	53.9	53.9	1,161	960	1,838	1,513	669	376
P1	P8	6,310	86.8	63.1	63.0	1,025	829	1,551	1,252	625	333
P4	P8	7,181	98.8	71.8	71.7	951	746	1,397	1,064	540	279

FACSDiva Version 6.1.3

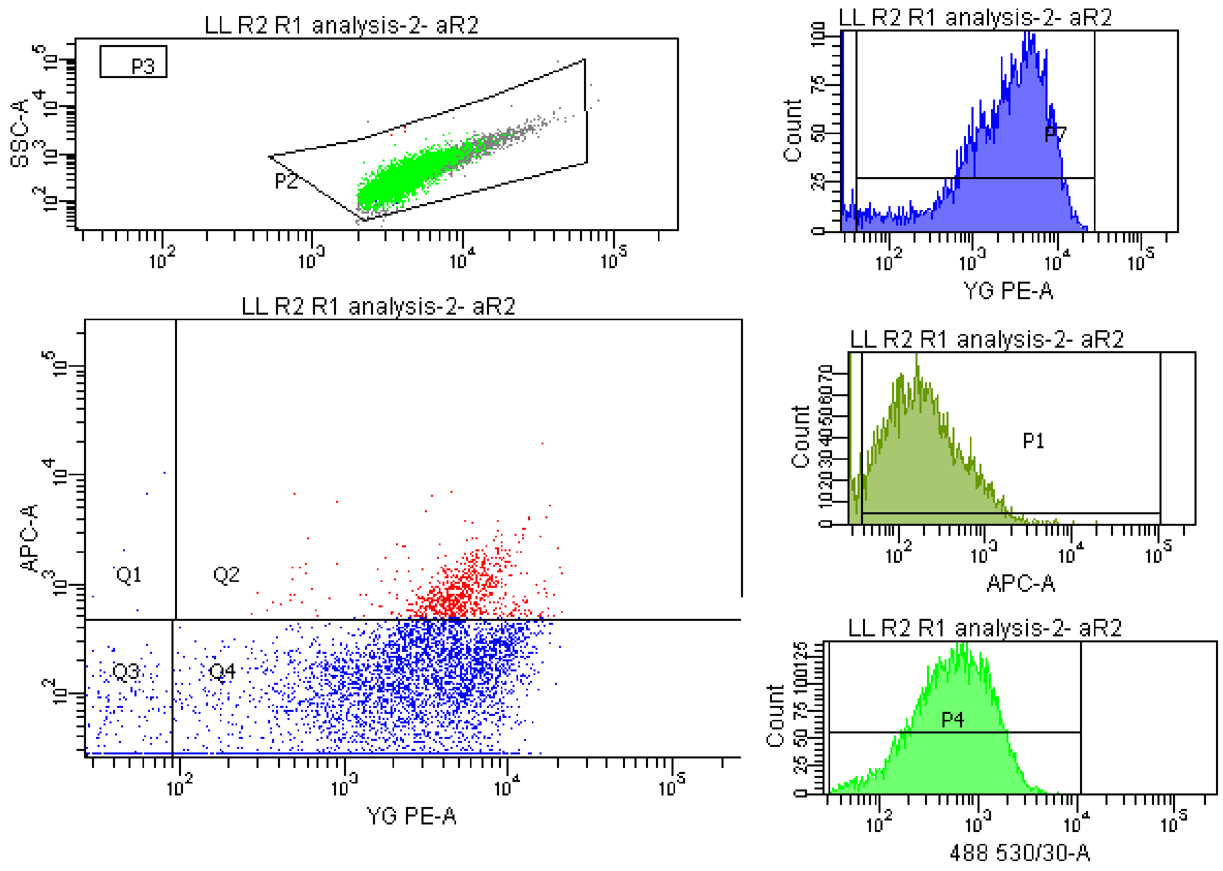

Experiment Name: Bio RBD2 selectons
Specimen Name: LL R2 R1 analysis
Tube Name: 2- aR2
Record Date: Jun 24, 2020 6:05:05 PM
$OP: Nileena
GUID: f8e6f8eb-e6ef-4464-80a9-b64f59d39618

Population	Parent...	#Events	%Parent	%Gran...	%Total	488 5... Mean	488 5... Median	PE-A Mean	PE-A Median	APC-A Mean	APC-A Median
All Events	####	####	####	####	####	1,011	664	1,285	758	279	87
P2	####	####	####	####	####	980	664	1,252	760	249	87
P8	P2	8,098	81.0	80.8	80.8	737	555	975	594	171	71
Q1	P8	16	0.2	0.2	0.2	799	567	145	84	3,037	1,994
Q2	P8	792	9.8	7.9	7.9	1,386	1,209	2,060	1,817	991	763
Q3	P8	2,094	25.9	20.9	20.9	249	214	29	17	8	1
Q4	P8	5,196	64.2	52.0	51.8	835	680	1,194	849	103	82
P7	P8	6,155	76.0	61.6	61.4	893	729	1,275	969	218	104
P1	P8	4,849	59.9	48.5	48.4	921	756	1,311	1,040	308	161
P4	P8	7,981	98.6	79.8	79.6	743	564	989	611	172	73

FACSDiva Version 6.1.3

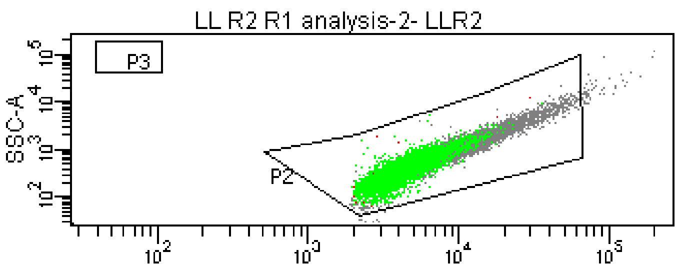

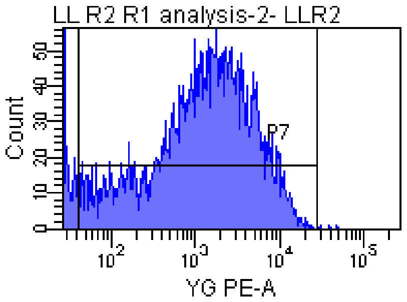

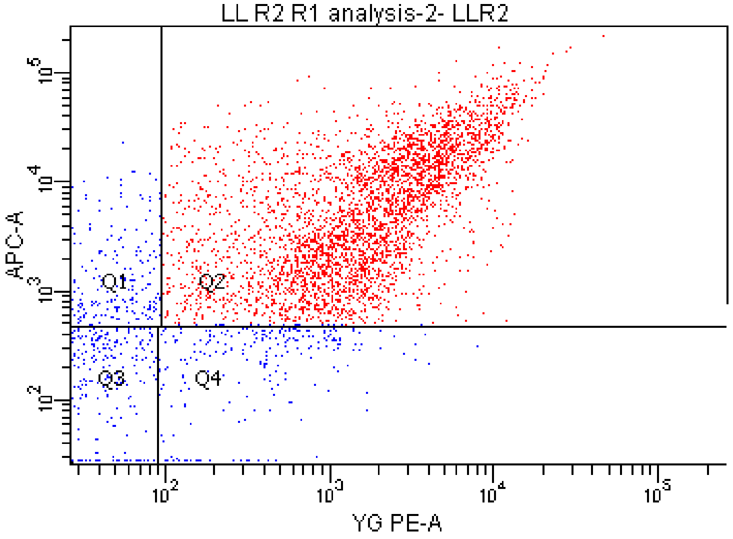

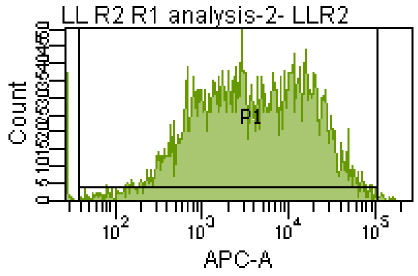

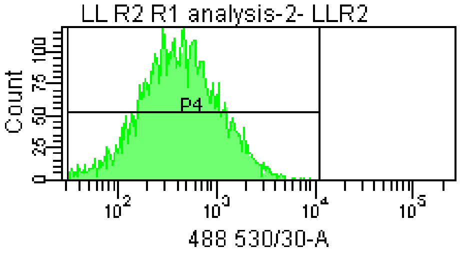

Experiment Name:	Bio RBD2 selectons
Specimen Name:	LL R2 R1 analysis
Tube Name:	2- LLR2
Record Date:	Jun 24, 2020 6:06:18 PM
$OP:	Nileena
GUID:	23285b18-fff1-444b-b656-6fe2771d4698

Population	Parent...	#Events	%Parent	%Gran...	%Total	488 5... Mean	488 5... Median	PE-A Mean	PE-A Median	APC-A Mean	APC-A Median
All Events	####	####	####	####	####	1,764	651	1,979	482	25,526	3,762
P2	####	####	####	####	####	1,580	648	1,770	479	24,455	3,706
P8	P2	6,594	65.9	65.5	65.5	603	398	565	176	6,335	1,068
Q1	P8	661	10.0	6.6	6.6	345	281	44	34	1,571	739
Q2	P8	3,610	54.7	36.1	35.9	869	638	991	616	11,163	5,202
Q3	P8	2,053	31.1	20.5	20.4	244	198	27	17	177	168
Q4	P8	270	4.1	2.7	2.7	408	346	231	156	278	312
P7	P8	4,124	62.5	41.2	41.0	793	586	873	510	9,681	3,758
P1	P8	6,166	93.5	61.7	61.2	608	416	585	219	6,483	1,325
P4	P8	6,427	97.5	64.3	63.8	611	411	573	189	6,417	1,164

FACSDiva Version 6.1.3

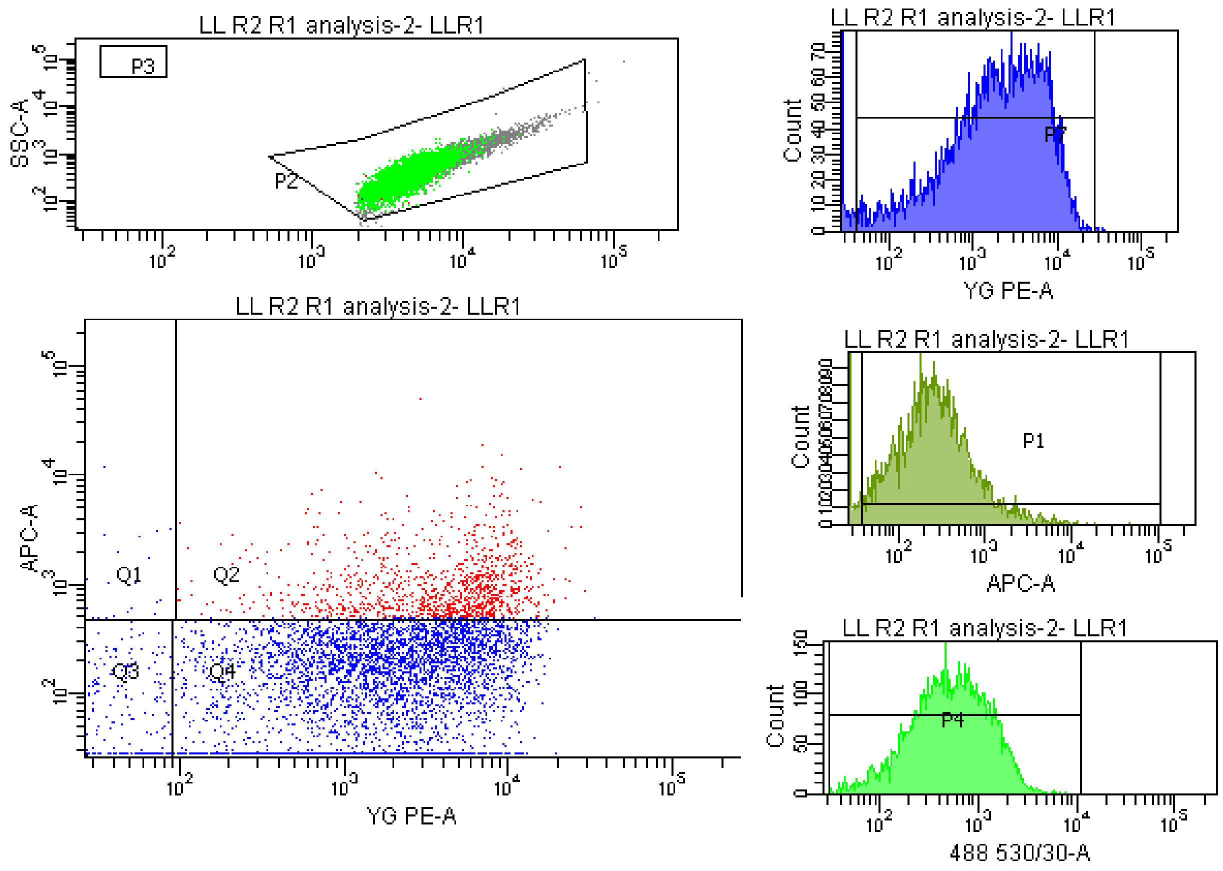

Experiment Name: Bio RBD2 selectons
Specimen Name: LL R2 R1 analysis
Tube Name: 2- LLR1
Record Date: Jun 24, 2020 6:07:26 PM
$OP: Nileena
GUID: 5c1a08ce-0cab-4a50-9169-b536ecb43399

Population	Parent...	#Events	%Parent	%Gran...	%Total	488 5... Mean	488 5... Median	PE-A Mean	PE-A Median	APC-A Mean	APC-A Median
All Events	####	####	####	####	####	1,081	657	1,340	610	656	211
P2	####	####	####	####	####	1,057	658	1,308	610	613	212
P8	P2	7,732	77.3	77.1	77.1	708	512	887	426	329	163
Q1	P8	98	1.3	1.0	1.0	396	302	57	38	1,478	688
Q2	P8	1,155	14.9	11.6	11.5	1,322	1,214	1,897	1,684	1,301	798
Q3	P8	2,002	25.9	20.0	20.0	255	214	29	15	85	69
Q4	P8	4,477	57.9	44.8	44.7	760	616	1,029	656	163	152
P7	P8	5,777	74.7	57.8	57.6	858	685	1,172	746	389	200
P1	P8	6,030	78.0	60.3	60.1	791	606	1,028	569	430	223
P4	P8	7,621	98.6	76.2	76.0	719	521	900	443	331	164

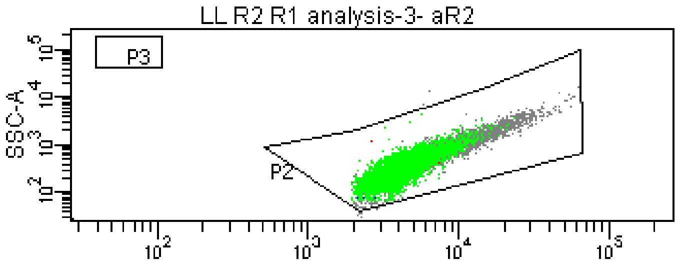

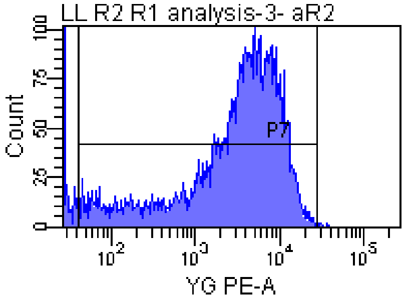

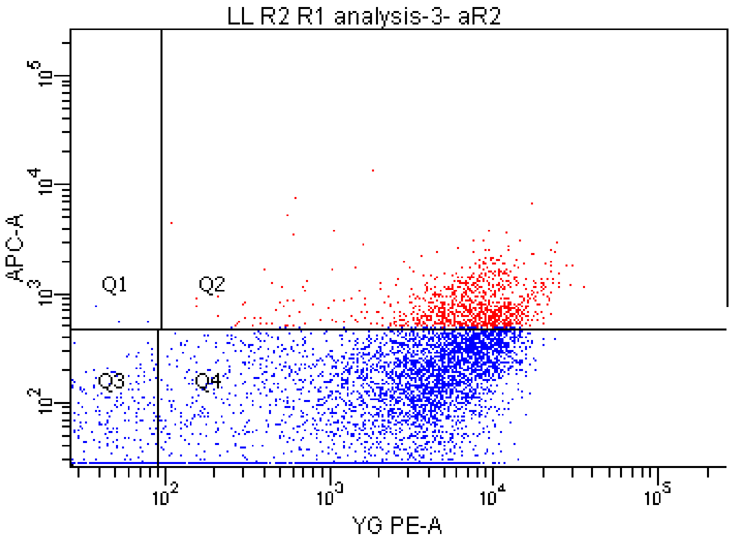

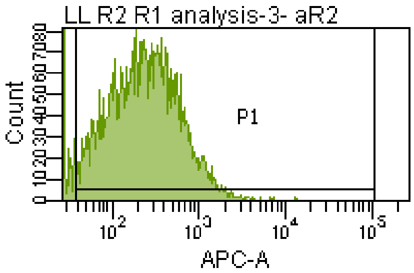

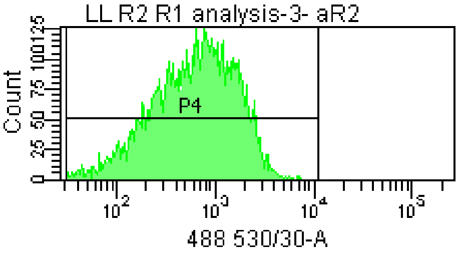

Experiment Name: Bio RBD2 selectons
Specimen Name: LL R2 R1 analysis
Tube Name: 3- aR2
Record Date: Jun 24, 2020 6:08:49 PM
$OP: Nileena
GUID: 44a36f1b-f2d9-4e1d-8a99-29731873db59

Population	Parent...	#Events	%Parent	%Gran...	%Total	488 5... Mean	488 5... Median	PE-A Mean	PE-A Median	APC-A Mean	APC-A Median
All Events	####	####	####	####	####	1,318	829	1,846	1,147	383	145
P2	####	####	####	####	####	1,320	832	1,848	1,149	379	146
P8	P2	7,628	76.3	76.1	76.1	869	645	1,262	812	213	102
Q1	P8	15	0.2	0.2	0.1	503	244	70	44	2,266	1,011
Q2	P8	1,005	13.2	10.0	10.0	1,757	1,597	3,003	2,772	881	698
Q3	P8	2,231	29.2	22.3	22.3	266	221	28	14	11	3
Q4	P8	4,377	57.4	43.8	43.7	973	806	1,496	1,235	155	133
P7	P8	5,623	73.7	56.2	56.1	1,081	877	1,697	1,366	279	173
P1	P8	5,117	67.1	51.2	51.1	1,098	909	1,714	1,416	328	204
P4	P8	7,501	98.3	75.0	74.9	884	656	1,284	851	216	105

FACSDiva Version 6.1.3

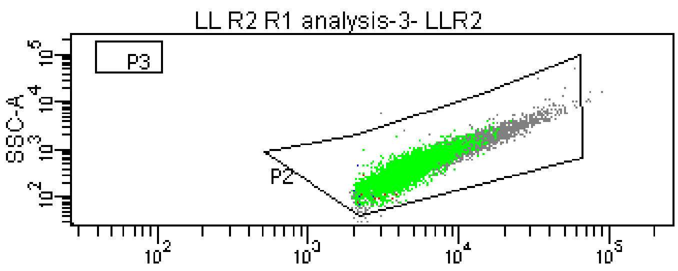

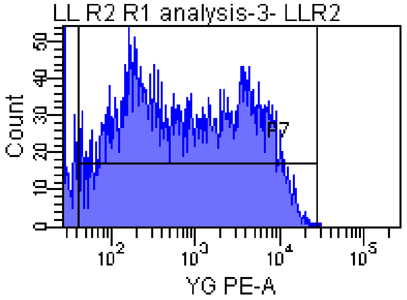

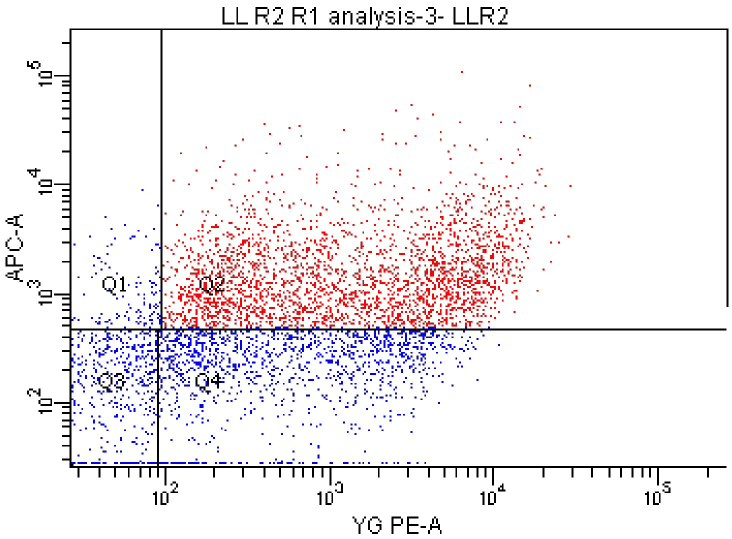

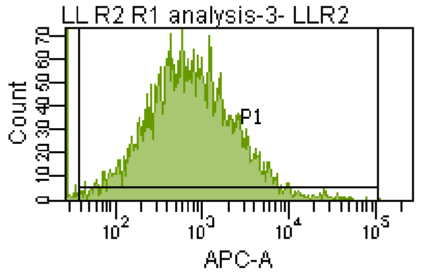

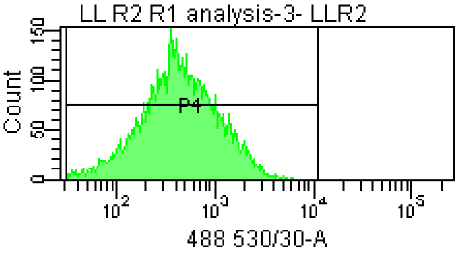

Experiment Name: Bio RBD2 selectons
Specimen Name: LL R2 R1 analysis
Tube Name: 3- LLR2
Record Date: Jun 24, 2020 6:10:09 PM
$OP: Nileena
GUID: f0d2574a-6d89-4788-9ee2-81388cf07c1a

Population	Parent...	#Events	%Parent	%Gran...	%Total	488 5... Mean	488 5... Median	PE-A Mean	PE-A Median	APC-A Mean	APC-A Median
All Events	####	####	####	####	####	1,060	570	971	227	3,101	578
P2	####	####	####	####	####	1,054	570	967	227	3,041	578
P8	P2	7,100	71.0	70.9	70.9	602	412	555	120	1,072	348
Q1	P8	186	2.6	1.9	1.9	410	361	59	49	1,375	693
Q2	P8	2,789	39.3	27.9	27.8	968	767	1,140	564	2,400	1,254
Q3	P8	2,650	37.3	26.5	26.4	283	234	33	19	110	89
Q4	P8	1,475	20.8	14.8	14.7	507	432	450	227	252	259
P7	P8	4,696	66.1	47.0	46.9	765	569	820	323	1,544	673
P1	P8	6,092	85.8	60.9	60.8	654	460	639	166	1,235	455
P4	P8	6,964	98.1	69.6	69.5	614	420	566	125	1,086	361

FACSDiva Version 6.1.3

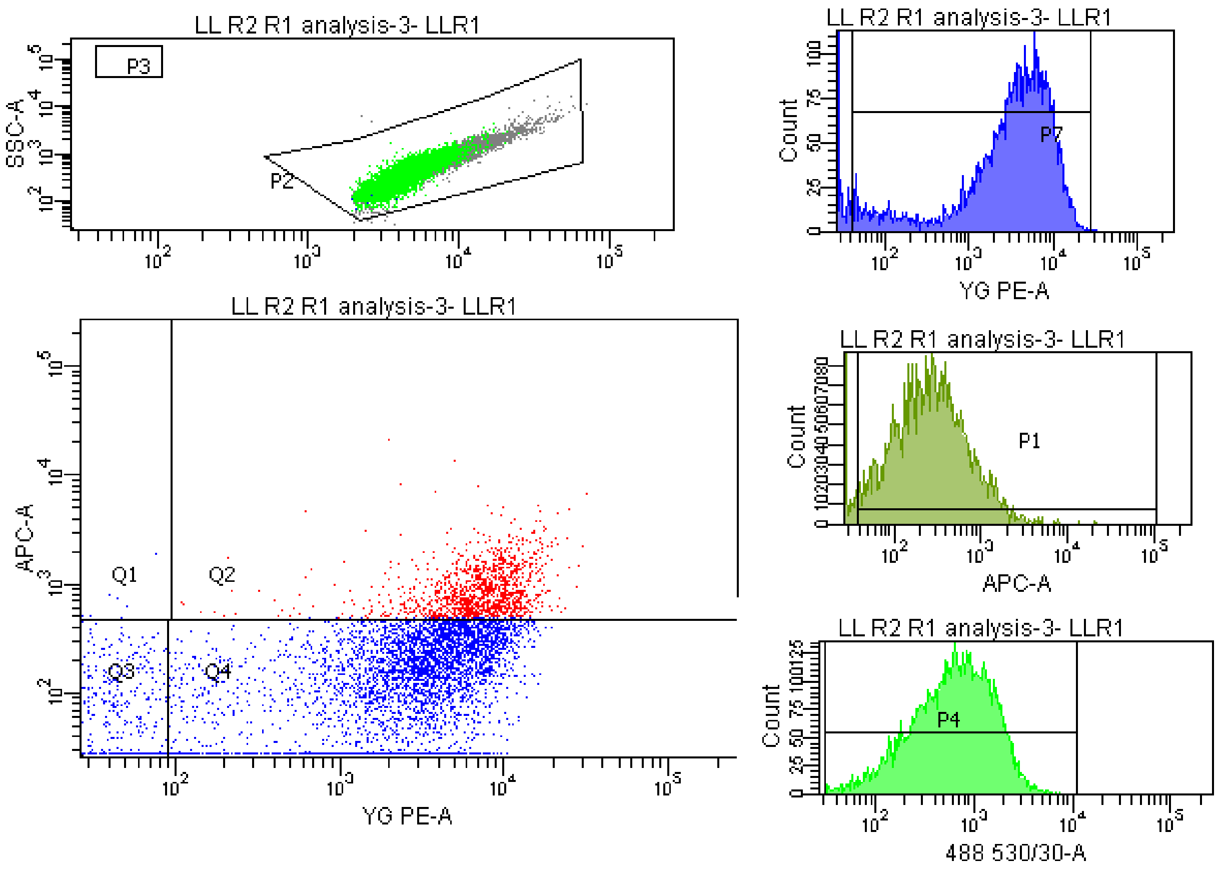

Experiment Name:	Bio RBD2 selectons
Specimen Name:	LL R2 R1 analysis
Tube Name:	3- LLR1
Record Date:	Jun 24, 2020 6:11:20 PM
$OP:	Nileena
GUID:	7853778e-9506-41c5-acdc-f5390c63a987

Population	Parent...	#Events	%Parent	%Gran...	%Total	488 5... Mean	488 5... Median	PE-A Mean	PE-A Median	APC-A Mean	APC-A Median
All Events	####	####	####	####	####	1,307	812	1,803	1,122	542	197
P2	####	####	####	####	####	1,283	814	1,804	1,124	516	198
P8	P2	7,676	76.8	76.6	76.6	834	630	1,194	801	276	144
Q1	P8	28	0.4	0.3	0.3	389	286	64	28	1,722	804
Q2	P8	1,228	16.0	12.3	12.3	1,702	1,568	2,882	2,651	1,014	760
Q3	P8	2,300	30.0	23.0	23.0	275	231	25	16	51	39
Q4	P8	4,120	53.7	41.2	41.1	890	758	1,352	1,124	172	155
P7	P8	5,585	72.8	55.8	55.7	1,041	862	1,626	1,301	351	208
P1	P8	5,808	75.7	58.1	58.0	973	795	1,478	1,172	372	213
P4	P8	7,560	98.5	75.6	75.4	847	642	1,213	823	280	146

FACSDiva Version 6.1.3

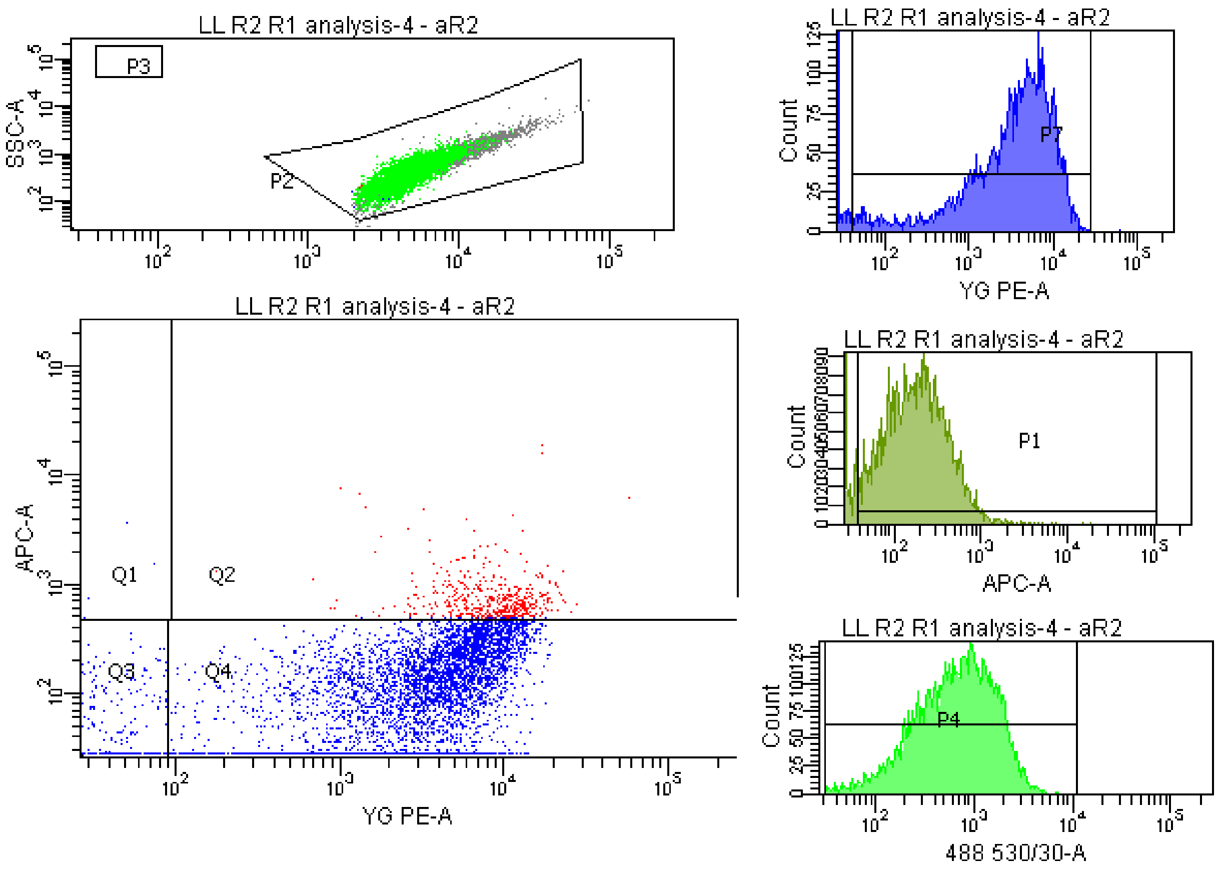

Experiment Name: Bio RBD2 selectons
Specimen Name: LL R2 R1 analysis
Tube Name: 4 - aR2
Record Date: Jun 24, 2020 6:12:42 PM
$OP: Nileena
GUID: 389404f0-f810-431e-af92-6735404fbd25

Population	Parent...	#Events	%Parent	%Gran...	%Total	488 5... Mean	488 5... Median	PE-A Mean	PE-A Median	APC-A Mean	APC-A Median
All Events	####	####	####	####	####	1,245	813	1,725	1,101	275	105
P2	####	####	####	####	####	1,241	814	1,722	1,105	273	105
P8	P2	8,013	80.1	80.0	80.0	878	669	1,266	873	148	84
Q1	P8	11	0.1	0.1	0.1	292	270	51	51	1,346	1,236
Q2	P8	495	6.2	5.0	4.9	2,072	1,867	3,444	3,379	876	626
Q3	P8	2,139	26.7	21.4	21.4	272	232	31	17	12	5
Q4	P8	5,368	67.0	53.7	53.6	1,010	868	1,560	1,307	132	111
P7	P8	6,028	75.2	60.3	60.2	1,075	898	1,671	1,377	190	124
P1	P8	5,144	64.2	51.4	51.3	1,107	944	1,725	1,504	244	161
P4	P8	7,898	98.6	79.0	78.8	888	683	1,282	897	149	86

FACSDiva Version 6.1.3

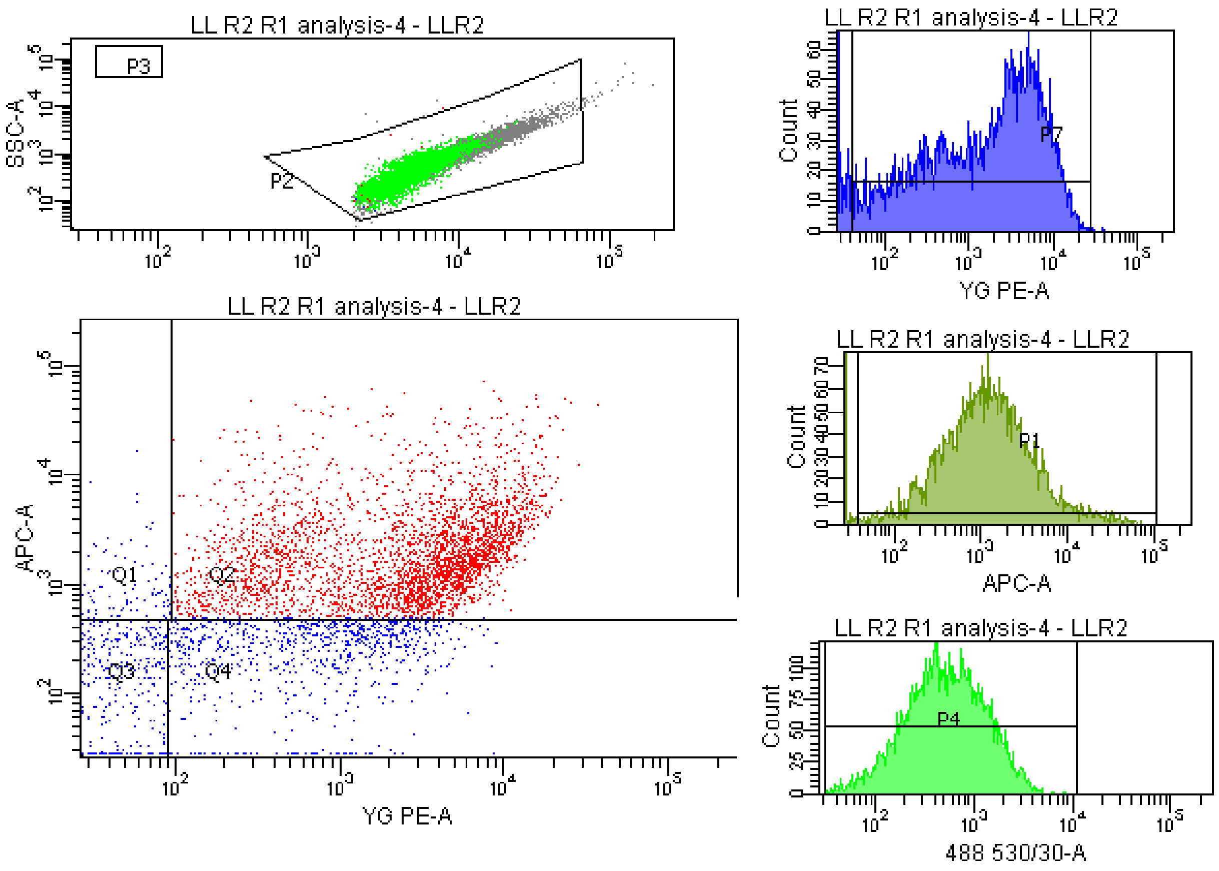

Experiment Name:	Bio RBD2 selectons
Specimen Name:	LL R2 R1 analysis
Tube Name:	4 - LLR2
Record Date:	Jun 24, 2020 6:13:58 PM
$OP:	Nileena
GUID:	becacc50-31dc-4f43-bcd2-76b2ed8f3b05

Population	Parent...	#Events	%Parent	%Gran...	%Total	488 5... Mean	488 5... Median	PE-A Mean	PE-A Median	APC-A Mean	APC-A Median
All Events	####	####	####	####	####	1,518	754	1,749	552	6,518	1,091
P2	####	####	####	####	####	1,437	754	1,668	547	6,057	1,087
P8	P2	6,908	69.1	68.8	68.8	725	495	804	223	1,735	573
Q1	P8	247	3.6	2.5	2.5	426	372	59	45	1,212	687
Q2	P8	3,466	50.2	34.7	34.5	1,099	904	1,466	1,116	3,218	1,593
Q3	P8	2,311	33.5	23.1	23.0	285	241	31	22	127	119
Q4	P8	884	12.8	8.8	8.8	492	427	440	305	267	282
P7	P8	4,607	66.7	46.1	45.9	941	740	1,186	760	2,487	1,121
P1	P8	6,186	89.5	61.9	61.6	773	550	893	317	1,941	713
P4	P8	6,801	98.5	68.0	67.7	737	505	817	233	1,756	589

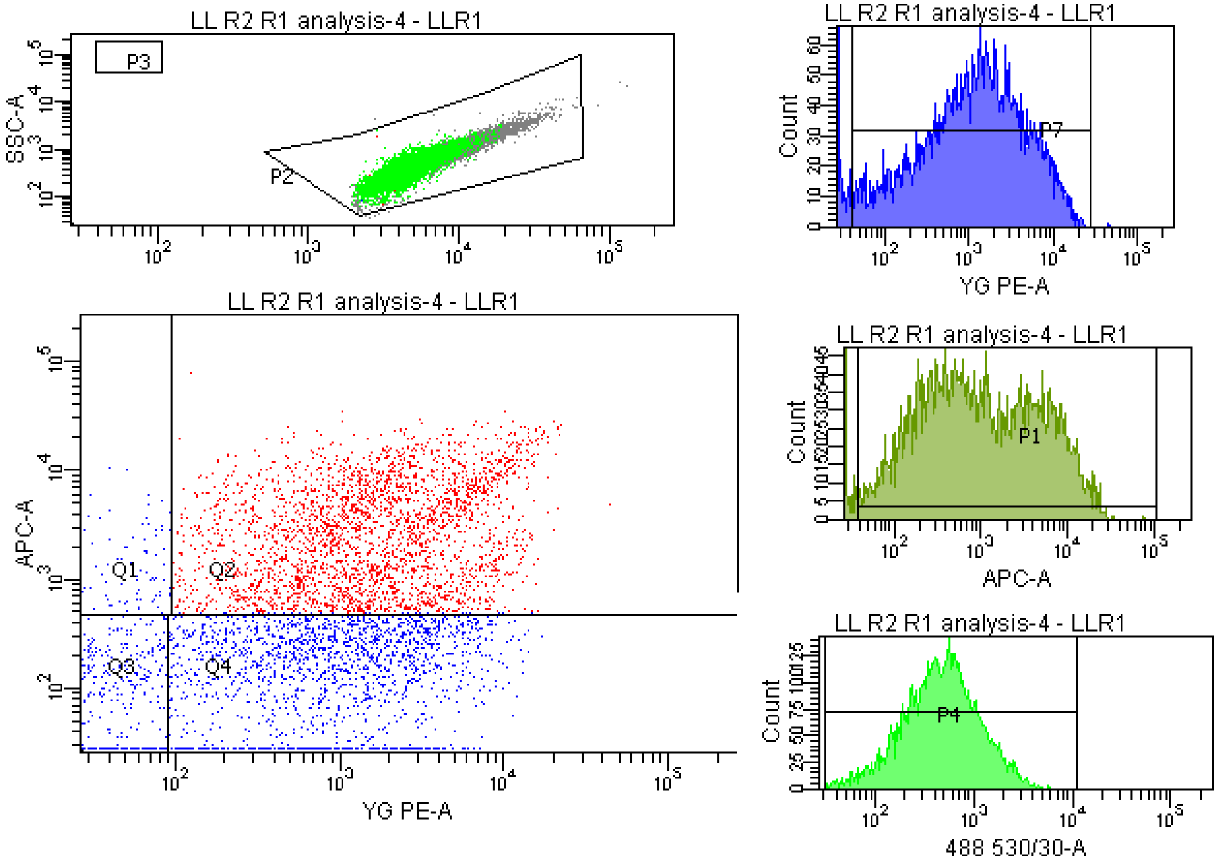

Experiment Name:	Bio RBD2 selectons
Specimen Name:	LL R2 R1 analysis
Tube Name:	4 - LLR1
Record Date:	Jun 24, 2020 6:15:03 PM
$OP:	Nileena
GUID:	13e763c1-1a26-4748-b050-08e9ff4312f9

Population	Parent...	#Events	%Parent	%Gran...	%Total	488 5... Mean	488 5... Median	PE-A Mean	PE-A Median	APC-A Mean	APC-A Median
All Events	####	####	####	####	####	1,143	616	1,183	391	4,115	584
P2	####	####	####	####	####	1,131	616	1,172	392	4,071	584
P8	P2	7,174	71.7	71.6	71.6	639	457	605	219	1,846	306
Q1	P8	137	1.9	1.4	1.4	341	287	65	45	1,717	905
Q2	P8	2,890	40.3	28.9	28.9	945	716	1,080	615	4,314	2,536
Q3	P8	2,364	33.0	23.6	23.6	283	237	33	19	85	68
Q4	P8	1,783	24.9	17.8	17.8	637	533	636	365	190	183
P7	P8	4,925	68.7	49.2	49.2	802	613	866	473	2,624	745
P1	P8	5,958	83.0	59.6	59.5	703	516	704	314	2,226	492
P4	P8	7,043	98.2	70.4	70.3	647	467	616	229	1,877	317

FACSDiva Version 6.1.3

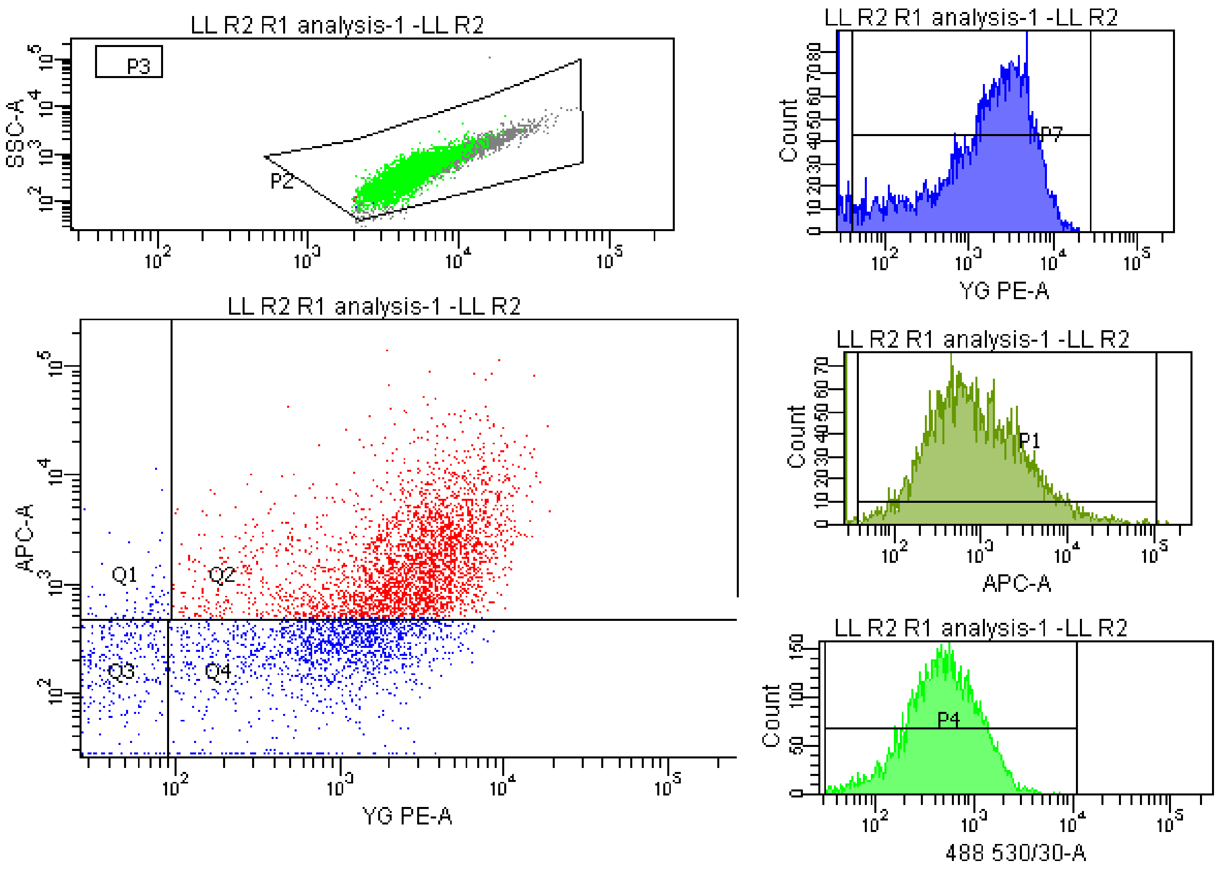

Experiment Name: Bio RBD2 selectons
Specimen Name: LL R2 R1 analysis
Tube Name: 1 -LL R2
Record Date: Jun 25, 2020 1:40:24 PM
$OP: Nileena
GUID: 5dbb0ac4-ac5a-470a-9beb-3fb9901b9567

Population	Parent...	#Events	%Parent	%Gran...	%Total	488 5... Mean	488 5... Median	PE-A Mean	PE-A Median	APC-A Mean	APC-A Median
All Events	####	####	####	####	####	897	584	866	439	2,515	511
P2	####	####	####	####	####	899	585	865	443	2,522	513
P8	P2	7,886	78.9	78.6	78.6	631	473	599	266	1,353	372
Q1	P8	218	2.8	2.2	2.2	415	377	71	46	972	615
Q2	P8	3,194	40.5	31.9	31.8	995	858	1,210	1,033	3,017	1,412
Q3	P8	2,894	36.7	28.9	28.8	286	251	37	21	143	128
Q4	P8	1,580	20.0	15.8	15.7	556	501	465	364	259	260
P7	P8	4,999	63.4	50.0	49.8	828	679	924	691	2,028	740
P1	P8	7,152	90.7	71.5	71.3	667	510	655	356	1,458	439
P4	P8	7,780	98.7	77.8	77.6	639	480	607	276	1,369	379

FACSDiva Version 6.1.3

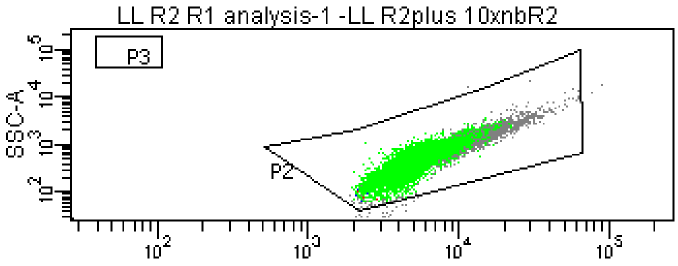

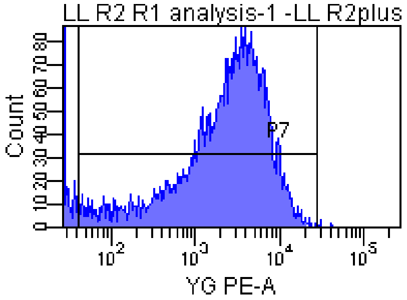

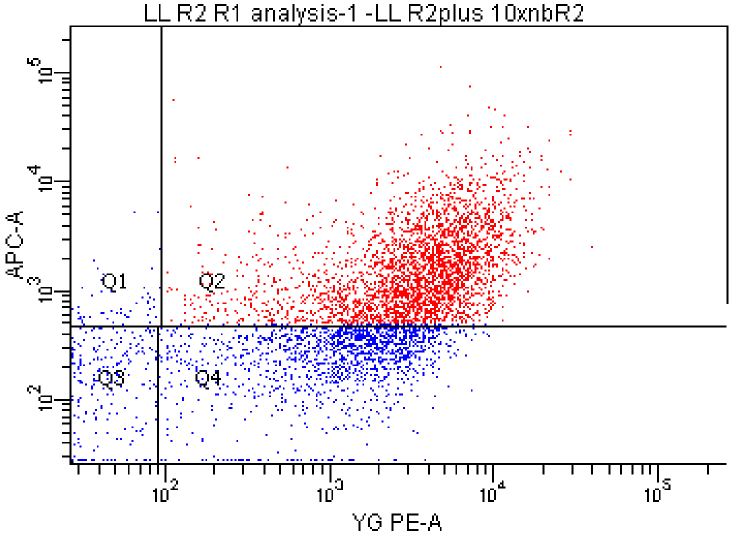

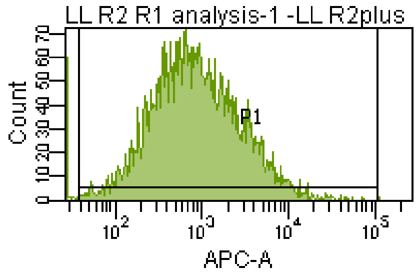

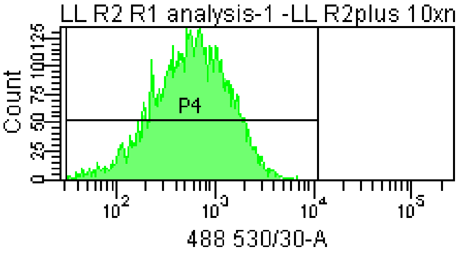

Experiment Name: Bio RBD2 selectons
Specimen Name: LL R2 R1 analysis
Tube Name: 1 -LL R2plus 10xnbR2
Record Date: Jun 25, 2020 1:42:13 PM
$OP: Nileena
GUID: 63afcc58-b836-409c-9aed-29f84daf2230

Population	Parent...	#Events	%Parent	%Gran...	%Total	488 5... Mean	488 5... Median	PE-A Mean	PE-A Median	APC-A Mean	APC-A Median
All Events	####	####	####	####	####	1,211	760	1,311	715	2,416	651
P2	####	####	####	####	####	1,210	764	1,310	722	2,386	653
P8	P2	7,417	74.2	73.8	73.8	770	576	830	436	1,231	431
Q1	P8	287	3.9	2.9	2.9	457	382	73	53	1,286	638
Q2	P8	3,245	43.8	32.4	32.3	1,209	1,041	1,584	1,333	2,473	1,339
Q3	P8	2,373	32.0	23.7	23.6	299	254	39	29	139	119
Q4	P8	1,512	20.4	15.1	15.0	626	574	596	488	267	272
P7	P8	4,914	66.3	49.1	48.9	998	820	1,224	947	1,714	776
P1	P8	6,791	91.6	67.9	67.6	813	625	901	543	1,328	504
P4	P8	7,350	99.1	73.5	73.1	777	580	837	447	1,241	438

FACSDiva Version 6.1.3

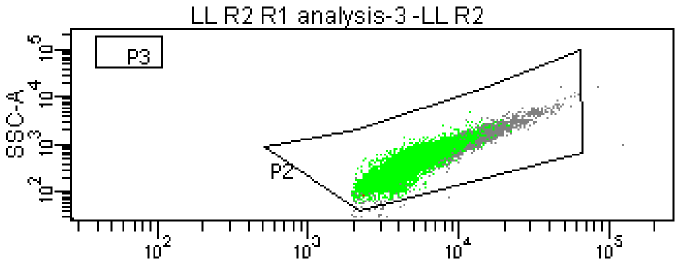

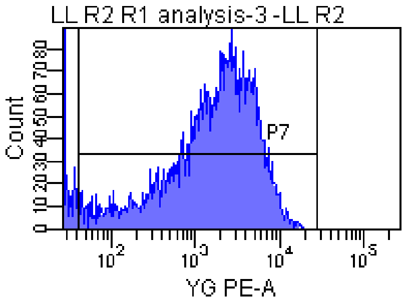

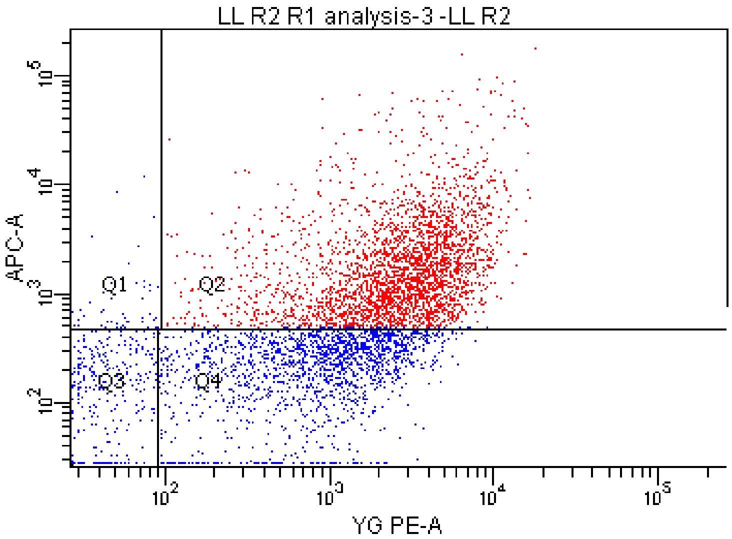

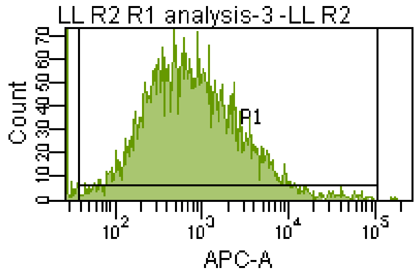

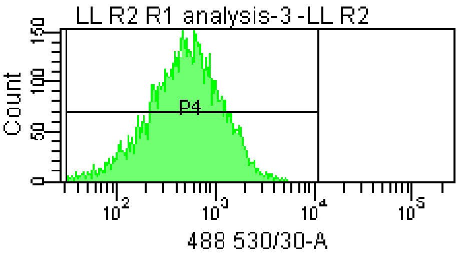

Experiment Name: Bio RBD2 selectons
Specimen Name: LL R2 R1 analysis
Tube Name: 3 -LL R2
Record Date: Jun 25, 2020 1:45:04 PM
$OP: Nileena
GUID: 4cc5d21c-cf18-453b-8fc8-41ea1a4ee784

Population	Parent...	#Events	%Parent	%Gran...	%Total	488 5... Mean	488 5... Median	PE-A Mean	PE-A Median	APC-A Mean	APC-A Median
All Events	####	####	####	####	####	1,030	631	975	490	3,738	524
P2	####	####	####	####	####	1,011	633	949	493	3,699	526
P8	P2	7,646	76.5	76.2	76.2	654	494	613	317	1,464	343
Q1	P8	156	2.0	1.6	1.6	464	366	78	54	972	608
Q2	P8	3,064	40.1	30.6	30.5	1,042	885	1,218	1,030	3,357	1,362
Q3	P8	2,636	34.5	26.4	26.3	283	243	38	27	121	101
Q4	P8	1,790	23.4	17.9	17.8	555	510	472	374	243	245
P7	P8	5,038	65.9	50.4	50.2	845	682	912	689	2,143	684
P1	P8	6,755	88.3	67.6	67.3	704	542	685	415	1,609	437
P4	P8	7,545	98.7	75.4	75.2	663	499	621	326	1,482	350

FACSDiva Version 6.1.3

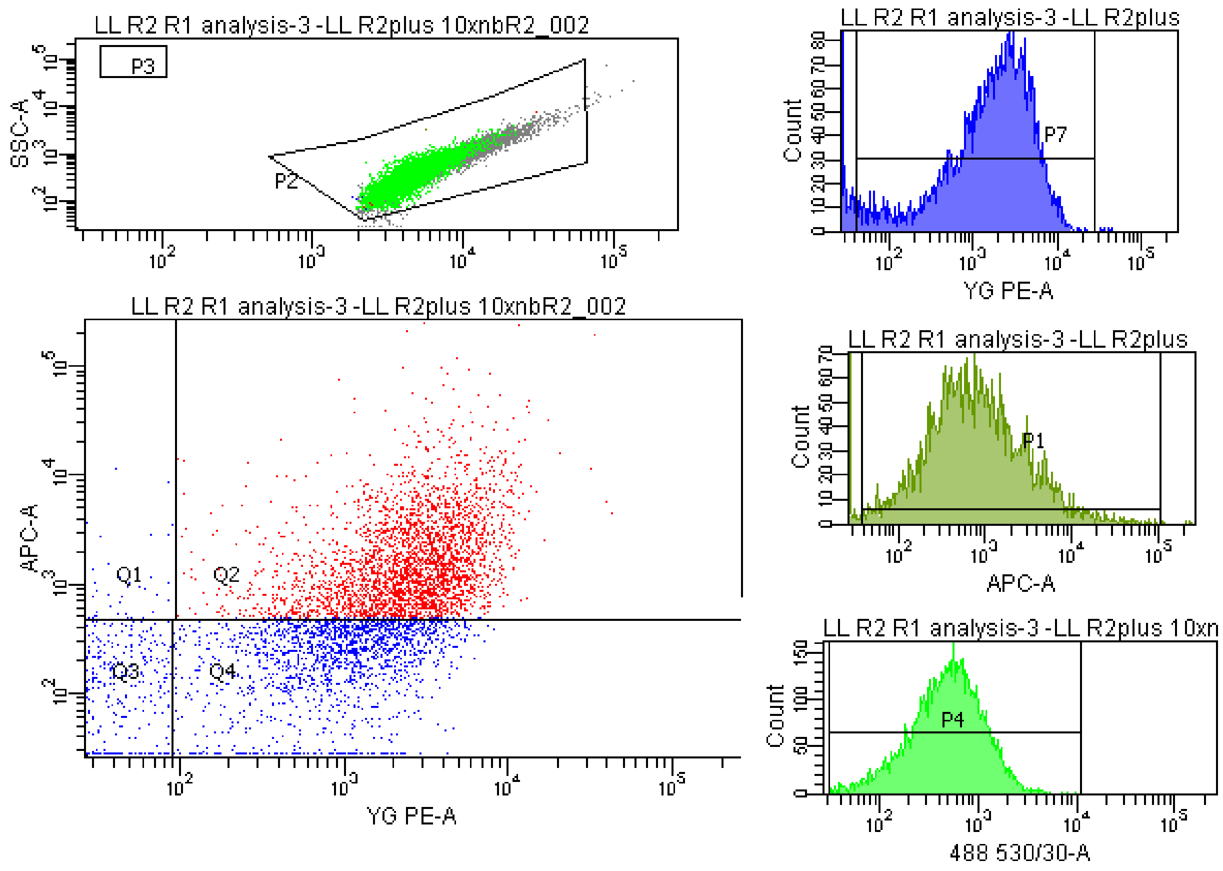

Experiment Name:	Bio RBD2 selectons
Specimen Name:	LL R2 R1 analysis
Tube Name:	3 -LL R2plus 10xnbR2_002
Record Date:	Jun 25, 2020 1:46:06 PM
$OP:	Nileena
GUID:	d48a8c5d-d20a-434e-9c22-9f01ee230076

Population	Parent...	#Events	%Parent	%Gran...	%Total	488 5... Mean	488 5... Median	PE-A Mean	PE-A Median	APC-A Mean	APC-A Median
All Events	####	####	####	####	####	1,045	621	956	487	3,851	559
P2	####	####	####	####	####	1,007	622	921	489	3,488	563
P8	P2	7,612	76.1	75.6	75.6	639	489	594	315	1,456	367
Q1	P8	212	2.8	2.1	2.1	392	322	53	43	1,767	726
Q2	P8	3,071	40.3	30.7	30.5	1,011	849	1,165	962	3,246	1,312
Q3	P8	2,606	34.2	26.1	25.9	282	241	38	21	125	104
Q4	P8	1,723	22.6	17.2	17.1	546	496	483	374	241	246
P7	P8	4,978	65.4	49.8	49.4	822	672	879	674	2,056	691
P1	P8	6,729	88.4	67.3	66.8	678	534	659	408	1,502	451
P4	P8	7,513	98.7	75.1	74.6	642	496	599	327	1,421	375

FACSDiva Version 6.1.3

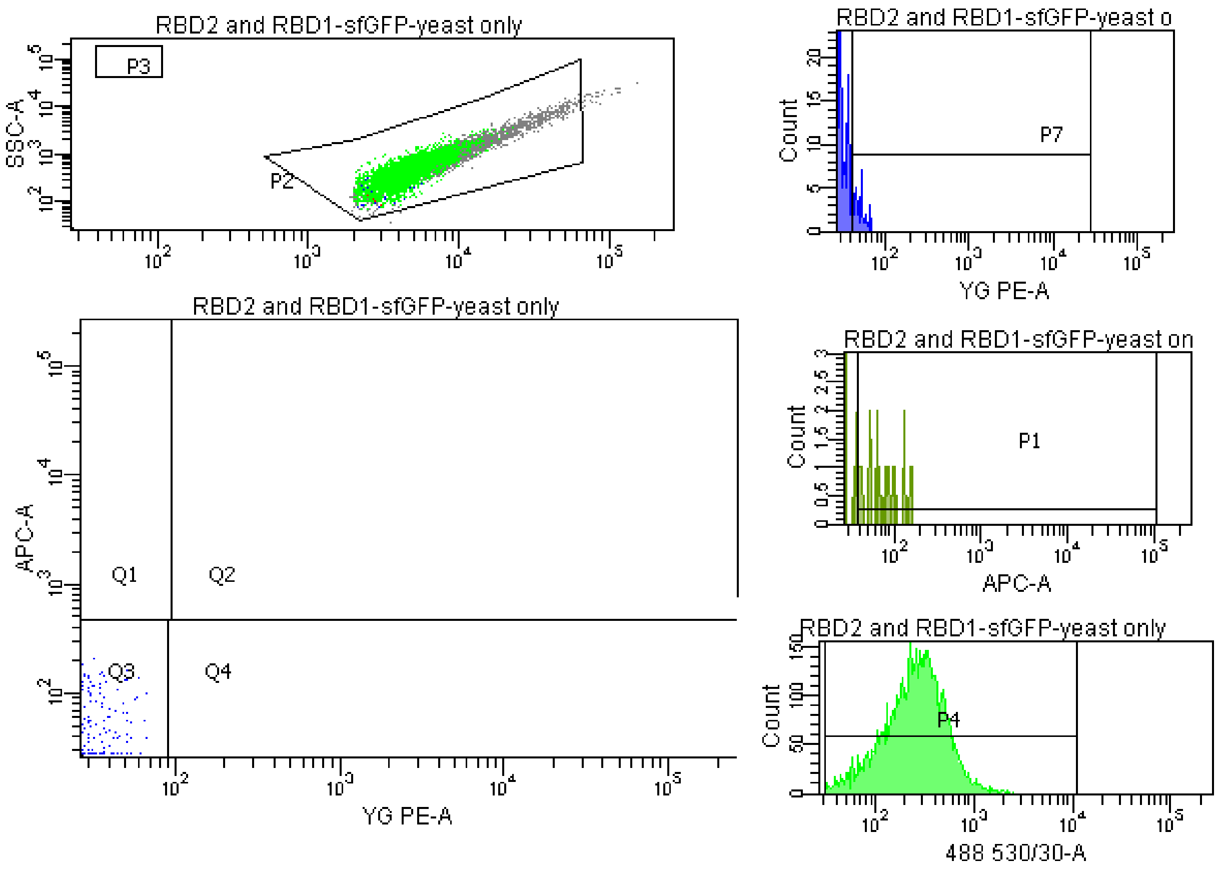

Experiment Name: Bio RBD2 selectons
Specimen Name: RBD2 and RBD1-sfGFP
Tube Name: yeast only
Record Date: Jul 14, 2020 12:07:19 PM
$OP: Nileena
GUID: 856096c9-3673-4aa1-91fa-9ec731115fc4

Population	Parent...	#Events	%Parent	%Gran...	%Total	488 5... Mean	488 5... Median	PE-A Mean	PE-A Median	APC-A Mean	APC-A Median
All Events	####	####	####	####	####	530	316	199	50	30	14
P2	####	####	####	####	####	499	316	171	50	29	14
P8	P2	6,666	66.7	66.4	66.4	282	242	61	29	16	8
Q1	P8	0	0.0	0.0	0.0	####	####	####	####	####	####
Q2	P8	0	0.0	0.0	0.0	####	####	####	####	####	####
Q3	P8	6,666	100.0	66.7	66.4	282	242	61	29	16	8
Q4	P8	0	0.0	0.0	0.0	####	####	####	####	####	####
P7	P8	34	0.5	0.3	0.3	447	430	139	101	55	41
P1	P8	1,847	27.7	18.5	18.4	358	300	76	37	81	69
P4	P8	6,203	93.1	62.0	61.8	304	257	64	32	17	10

FACSDiva Version 6.1.3

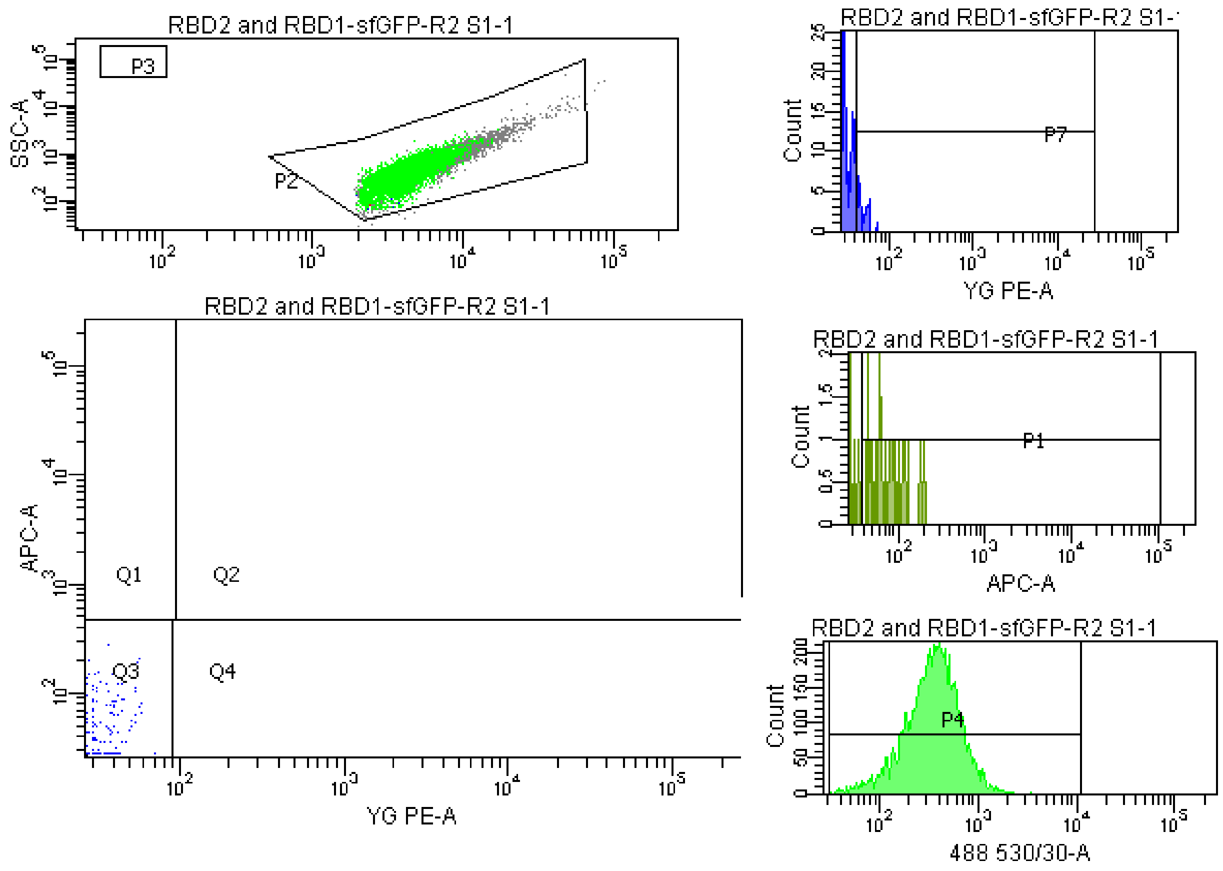

Experiment Name: Bio RBD2 selectons
Specimen Name: RBD2 and RBD1-sfGFP
Tube Name: R2 S1-1
Record Date: Jul 14, 2020 12:08:35 PM
$OP: Nileena
GUID: ce9b5587-2a6d-45ef-b9c5-8a5f4955edd2

Population	Parent...	#Events	%Parent	%Gran...	%Total	488 5... Mean	488 5... Median	PE-A Mean	PE-A Median	APC-A Mean	APC-A Median
All Events	####	####	####	####	####	564	392	103	52	24	12
P2	####	####	####	####	####	555	393	98	52	23	12
P8	P2	8,079	80.8	80.7	80.7	393	348	54	44	16	8
Q1	P8	0	0.0	0.0	0.0	####	####	####	####	####	####
Q2	P8	0	0.0	0.0	0.0	####	####	####	####	####	####
Q3	P8	8,079	100.0	80.8	80.7	393	348	54	44	16	8
Q4	P8	0	0.0	0.0	0.0	####	####	####	####	####	####
P7	P8	31	0.4	0.3	0.3	530	456	126	117	70	62
P1	P8	2,173	26.9	21.7	21.7	448	389	67	52	76	66
P4	P8	7,999	99.0	80.0	79.9	397	350	54	44	16	9

FACSDiva Version 6.1.3

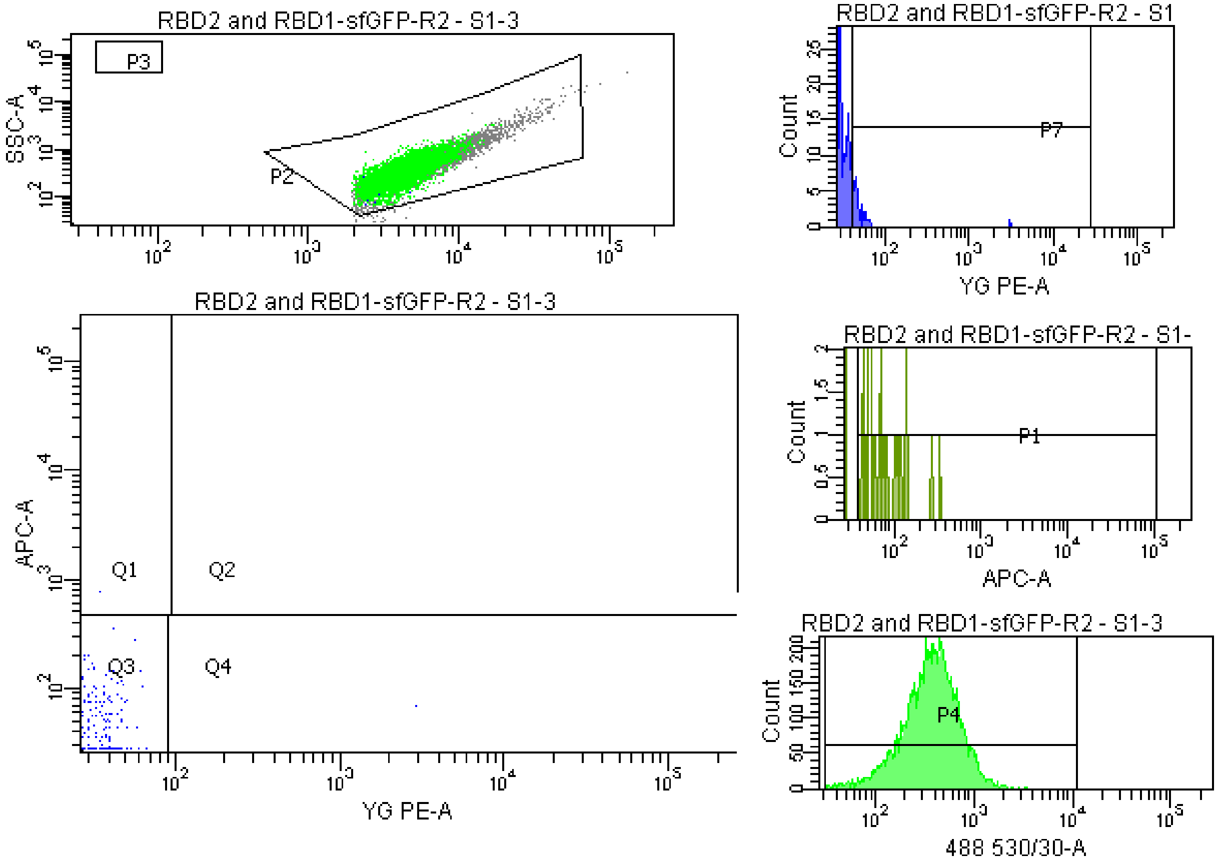

Experiment Name: Bio RBD2 selectons
Specimen Name: RBD2 and RBD1-sfGFP
Tube Name: R2 - S1-3
Record Date: Jul 14, 2020 12:10:03 PM
$OP: Nileena
GUID: 7b4c66ee-b4b7-43a7-ac78-d35d7229c041

Population	Parent...	#Events	%Parent	%Gran...	%Total	488 5... Mean	488 5... Median	PE-A Mean	PE-A Median	APC-A Mean	APC-A Median
All Events	####	####	####	####	####	617	420	95	43	24	13
P2	####	####	####	####	####	605	421	89	44	24	13
P8	P2	7,954	79.5	79.1	79.1	424	370	48	35	16	9
Q1	P8	1	0.0	0.0	0.0	1,914	1,914	213	213	737	737
Q2	P8	0	0.0	0.0	0.0	####	####	####	####	####	####
Q3	P8	7,952	100.0	79.5	79.1	423	370	48	35	16	9
Q4	P8	1	0.0	0.0	0.0	763	763	898	898	67	67
P7	P8	39	0.5	0.4	0.4	677	653	164	123	58	48
P1	P8	2,204	27.7	22.0	21.9	488	416	61	44	78	66
P4	P8	7,856	98.8	78.6	78.1	429	373	48	35	16	9

FACSDiva Version 6.1.3

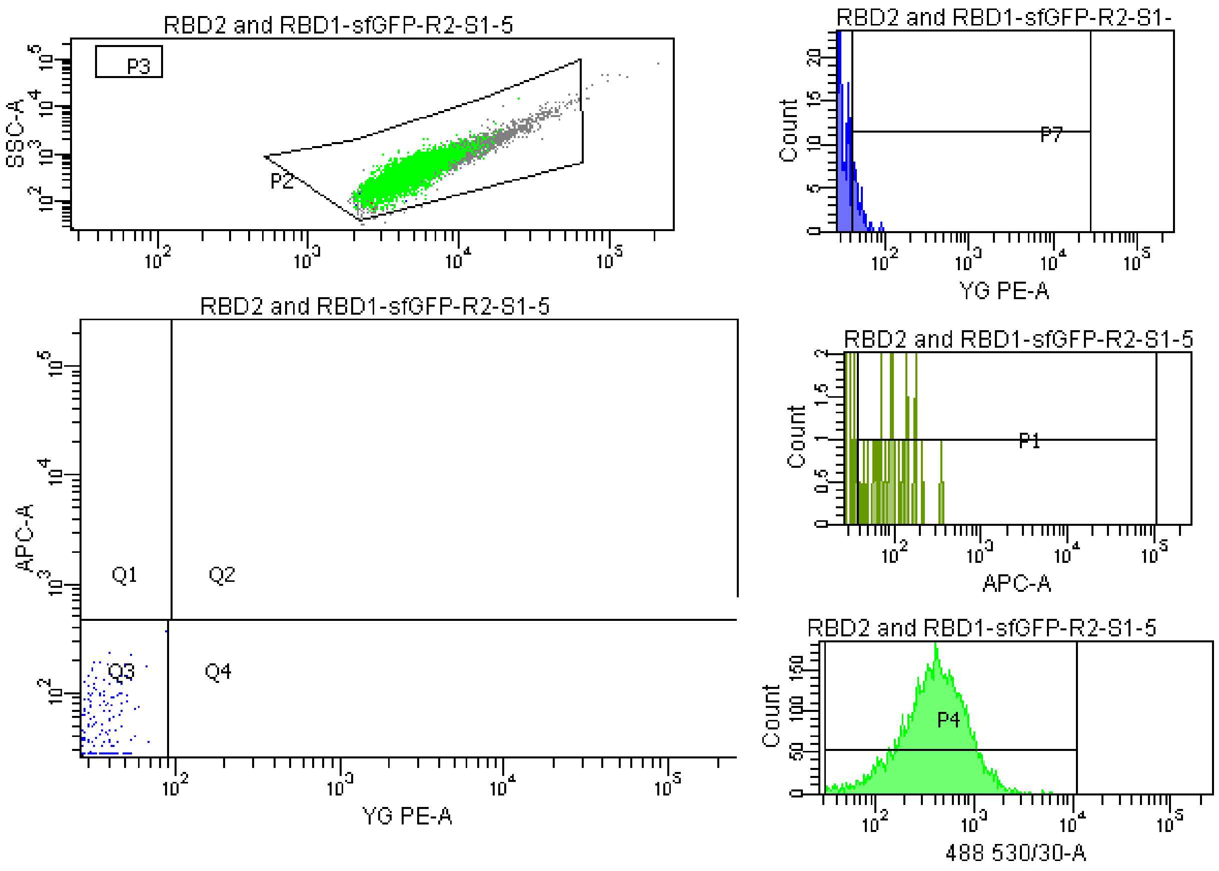

Experiment Name: Bio RBD2 selectons
Specimen Name: RBD2 and RBD1-sfGFP
Tube Name: R2-S1-5
Record Date: Jul 14, 2020 12:11:29 PM
$OP: Nileena
GUID: 71167f66-9948-469e-8680-bdcbc1384bd1

Population	Parent...	#Events	%Parent	%Gran...	%Total	488 5... Mean	488 5... Median	PE-A Mean	PE-A Median	APC-A Mean	APC-A Median
All Events	####	####	####	####	####	824	483	150	50	29	15
P2	####	####	####	####	####	779	483	124	50	27	15
P8	P2	7,570	75.7	75.5	75.5	478	394	57	35	16	9
Q1	P8	1	0.0	0.0	0.0	1,279	1,279	134	134	1,185	1,185
Q2	P8	0	0.0	0.0	0.0	####	####	####	####	####	####
Q3	P8	7,569	100.0	75.7	75.5	478	393	57	35	16	9
Q4	P8	0	0.0	0.0	0.0	####	####	####	####	####	####
P7	P8	47	0.6	0.5	0.5	967	708	229	126	67	61
P1	P8	2,063	27.3	20.6	20.6	592	473	70	48	78	68
P4	P8	7,440	98.3	74.4	74.2	487	400	57	36	16	9

FACSDiva Version 6.1.3

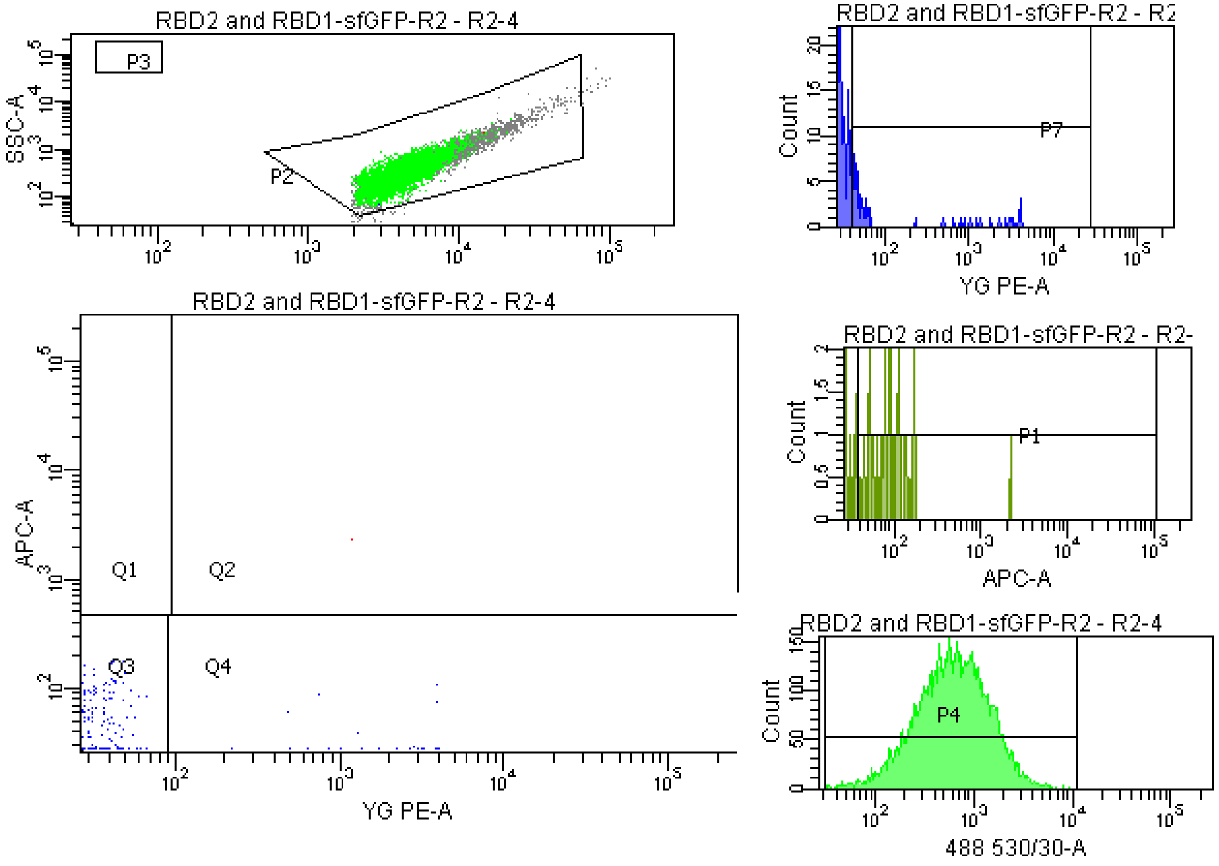

Experiment Name: Bio RBD2 selectons
Specimen Name: RBD2 and RBD1-sfGFP
Tube Name: R2 - R2-4
Record Date: Jul 14, 2020 12:12:22 PM
$OP: Nileena
GUID: 34175053-e124-4cf5-9ce8-b54e721fe74b

Population	Parent...	#Events	%Parent	%Gran...	%Total	488 5... Mean	488 5... Median	PE-A Mean	PE-A Median	APC-A Mean	APC-A Median
All Events	####	####	####	####	####	1,371	726	118	46	28	12
P2	####	####	####	####	####	1,290	728	101	46	26	12
P8	P2	7,943	79.4	78.9	78.9	810	605	50	36	15	8
Q1	P8	1	0.0	0.0	0.0	348	348	97	97	2,467	2,467
Q2	P8	1	0.0	0.0	0.0	252	252	425	425	2,226	2,226
Q3	P8	7,920	99.7	79.2	78.7	810	605	48	36	14	8
Q4	P8	21	0.3	0.2	0.2	817	699	769	828	11	10
P7	P8	65	0.8	0.6	0.6	923	789	327	129	76	33
P1	P8	2,103	26.5	21.0	20.9	903	637	64	45	79	67
P4	P8	7,889	99.3	78.9	78.4	814	609	50	36	15	8

FACSDiva Version 6.1.3

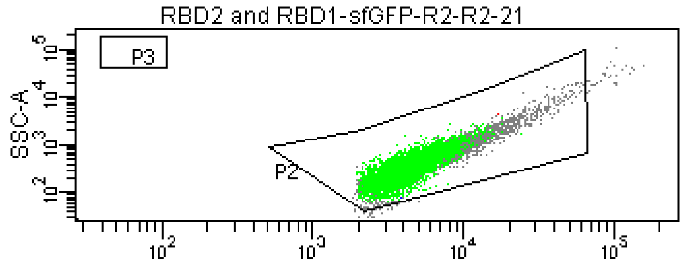

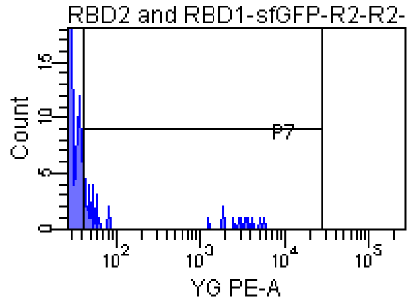

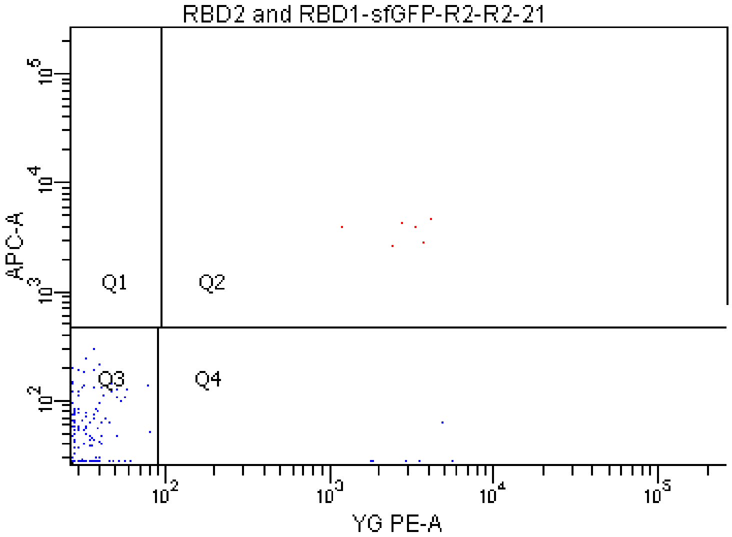

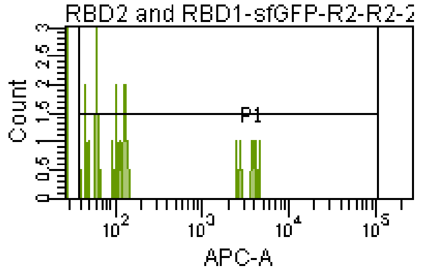

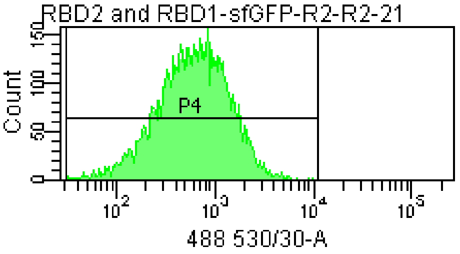

Experiment Name:	Bio RBD2 selectons
Specimen Name:	RBD2 and RBD1-sfGFP
Tube Name:	R2-R2-21
Record Date:	Jul 14, 2020 12:14:15 PM
$OP:	Nileena
GUID:	d4d0921e-834e-4599-989c-d224f1cd0c59

Population	Parent...	#Events	%Parent	%Gran...	%Total	488 5... Mean	488 5... Median	PE-A Mean	PE-A Median	APC-A Mean	APC-A Median
All Events	####	####	####	####	####	1,501	719	171	41	38	14
P2	####	####	####	####	####	1,288	719	114	41	34	14
P8	P2	7,919	79.2	78.6	78.6	791	603	49	32	20	10
Q1	P8	4	0.1	0.0	0.0	323	341	24	6	2,076	972
Q2	P8	6	0.1	0.1	0.1	1,573	1,251	1,026	1,026	3,592	3,814
Q3	P8	7,903	99.8	79.0	78.4	791	602	47	32	16	10
Q4	P8	6	0.1	0.1	0.1	1,044	1,116	1,164	1,141	-1	-12
P7	P8	37	0.5	0.4	0.4	1,150	1,032	428	145	628	61
P1	P8	2,156	27.2	21.6	21.4	886	649	65	43	90	66
P4	P8	7,840	99.0	78.4	77.8	798	608	49	33	20	10

FACSDiva Version 6.1.3

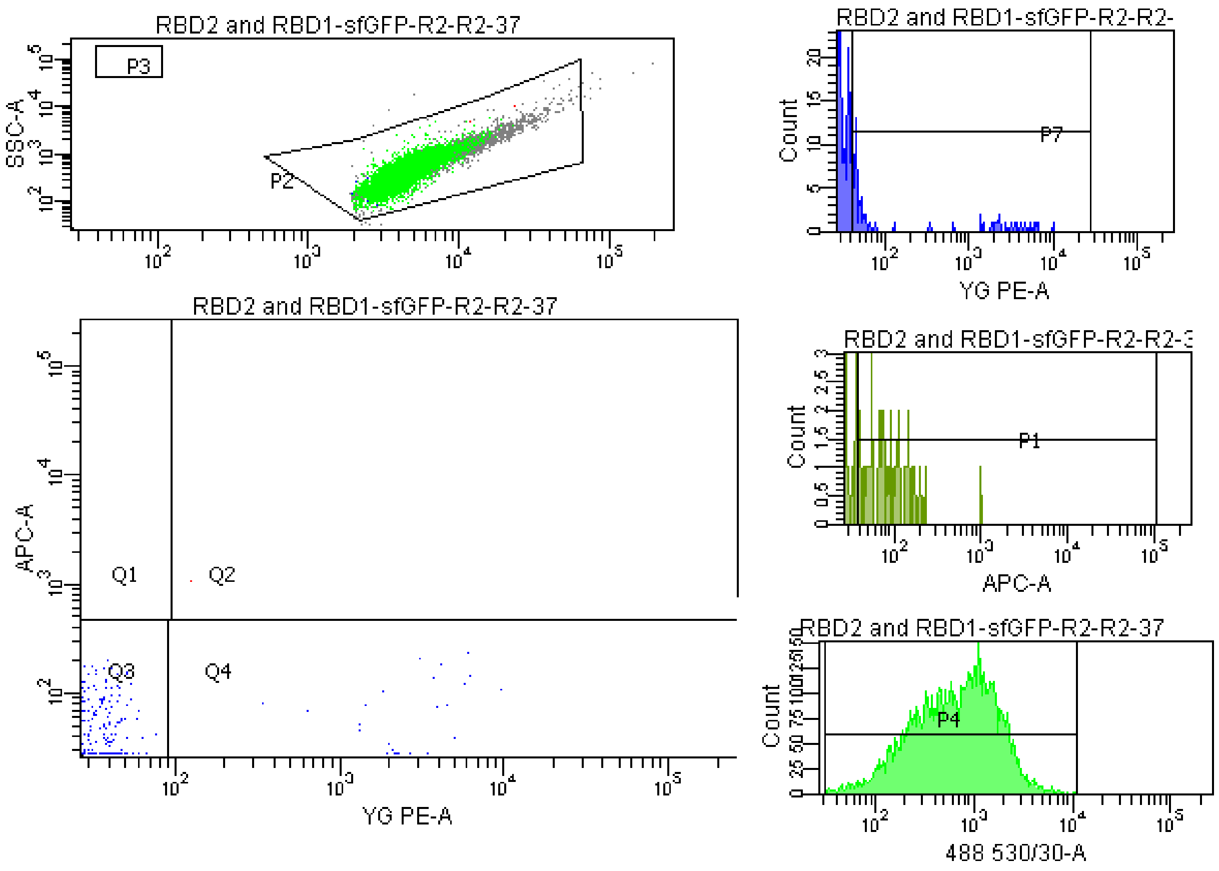

Experiment Name: Bio RBD2 selectons
Specimen Name: RBD2 and RBD1-sfGFP
Tube Name: R2-R2-37
Record Date: Jul 14, 2020 12:15:29 PM
$OP: Nileena
GUID: 2d638a3e-612f-4985-bfd4-c65bed79058f

Population	Parent...	#Events	%Parent	%Gran...	%Total	488 5... Mean	488 5... Median	PE-A Mean	PE-A Median	APC-A Mean	APC-A Median
All Events	####	####	####	####	####	1,581	860	163	54	25	11
P2	####	####	####	####	####	1,509	859	141	54	23	11
P8	P2	7,838	78.4	78.2	78.2	913	662	66	43	13	6
Q1	P8	0	0.0	0.0	0.0	####	####	####	####	####	####
Q2	P8	1	0.0	0.0	0.0	2,014	2,014	4,638	4,638	1,007	1,007
Q3	P8	7,813	99.7	78.1	78.0	911	661	62	43	13	6
Q4	P8	24	0.3	0.2	0.2	1,575	1,289	1,242	989	71	68
P7	P8	72	0.9	0.7	0.7	1,452	1,250	587	165	69	48
P1	P8	1,945	24.8	19.4	19.4	1,132	838	100	56	75	65
P4	P8	7,754	98.9	77.5	77.4	920	672	67	44	13	7

FACSDiva Version 6.1.3

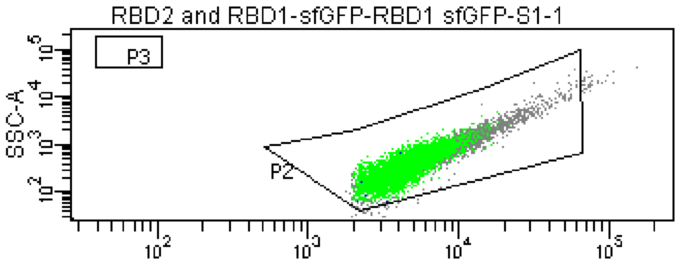

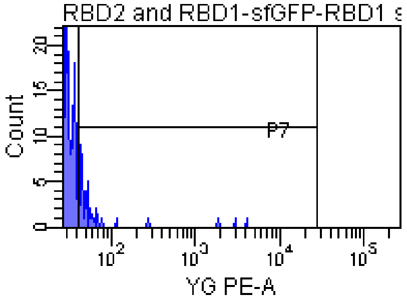

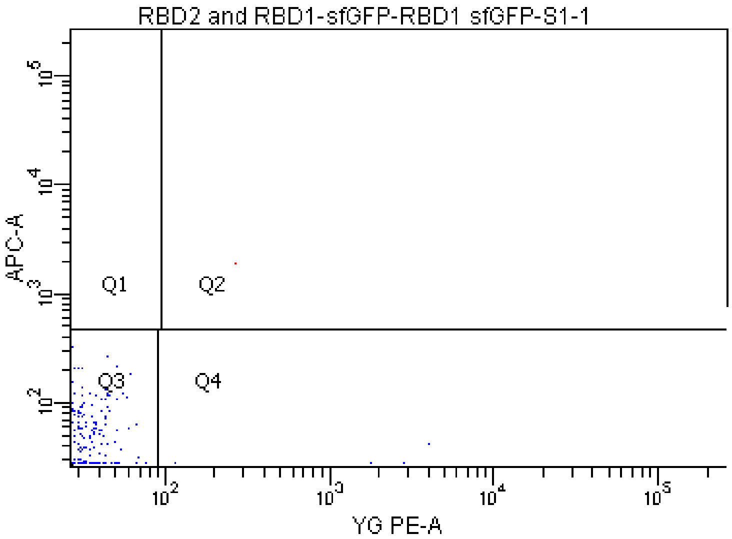

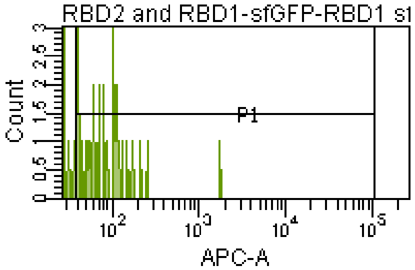

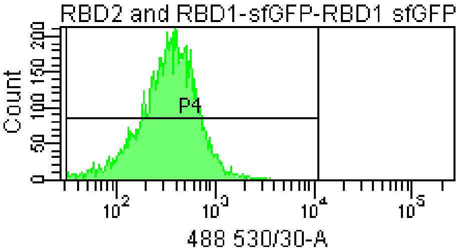

Experiment Name: Bio RBD2 selectons
Specimen Name: RBD2 and RBD1-sfGFP
Tube Name: RBD1 sfGFP-S1-1
Record Date: Jul 14, 2020 12:17:10 PM
$OP: Nileena
GUID: 7efd88e8-c183-4a90-8f05-87766b984a0f

Population	Parent...	#Events	%Parent	%Gran...	%Total	488 5... Mean	488 5... Median	PE-A Mean	PE-A Median	APC-A Mean	APC-A Median
All Events	####	####	####	####	####	718	392	152	43	32	13
P2	####	####	####	####	####	644	392	116	43	29	13
P8	P2	7,898	79.0	78.4	78.4	394	340	49	34	16	8
Q1	P8	1	0.0	0.0	0.0	323	323	29	29	1,598	1,598
Q2	P8	1	0.0	0.0	0.0	443	443	133	133	1,791	1,791
Q3	P8	7,892	99.9	78.9	78.3	393	340	49	34	15	8
Q4	P8	4	0.1	0.0	0.0	903	767	796	751	8	4
P7	P8	44	0.6	0.4	0.4	674	558	288	109	103	55
P1	P8	2,154	27.3	21.5	21.4	461	395	64	43	79	66
P4	P8	7,775	98.4	77.8	77.1	400	344	50	34	16	9

FACSDiva Version 6.1.3

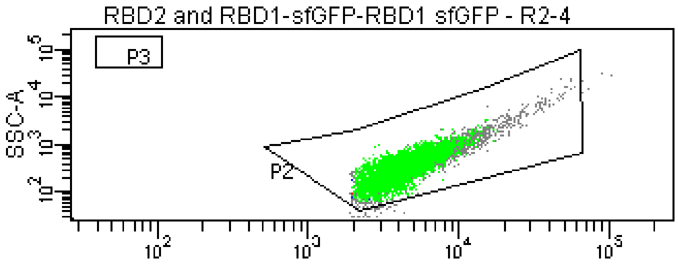

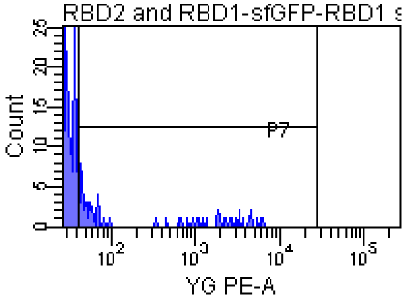

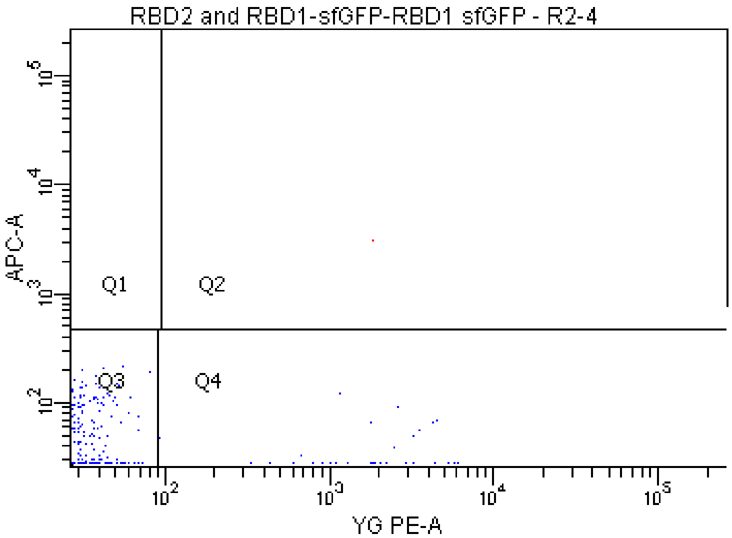

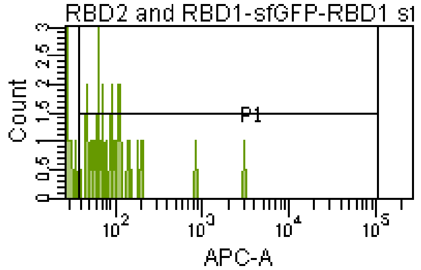

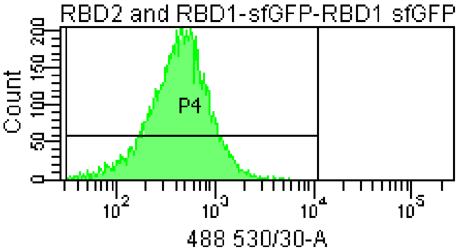

Experiment Name: Bio RBD2 selectons
Specimen Name: RBD2 and RBD1-sfGFP
Tube Name: RBD1 sfGFP - R2-4
Record Date: Jul 14, 2020 12:18:58 PM
$OP: Nileena
GUID: 80b7347d-35a5-4876-ad1d-6741d834d42f

Population	Parent...	#Events	%Parent	%Gran...	%Total	488 5... Mean	488 5... Median	PE-A Mean	PE-A Median	APC-A Mean	APC-A Median
All Events	####	####	####	####	####	759	476	90	36	31	9
P2	####	####	####	####	####	743	477	85	36	30	9
P8	P2	8,403	84.0	83.5	83.5	518	436	46	29	14	7
Q1	P8	1	0.0	0.0	0.0	828	828	114	114	850	850
Q2	P8	2	0.0	0.0	0.0	1,028	1,028	381	381	1,900	1,900
Q3	P8	8,371	99.6	83.7	83.2	517	436	43	29	14	7
Q4	P8	29	0.3	0.3	0.3	777	591	795	628	16	6
P7	P8	74	0.9	0.7	0.7	870	693	401	144	88	29
P1	P8	2,248	26.8	22.5	22.3	591	490	57	38	77	65
P4	P8	8,289	98.6	82.9	82.4	525	440	46	31	14	7

FACSDiva Version 6.1.3

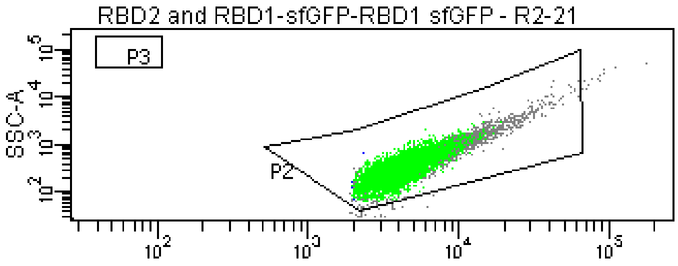

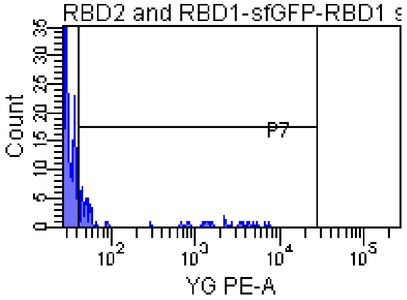

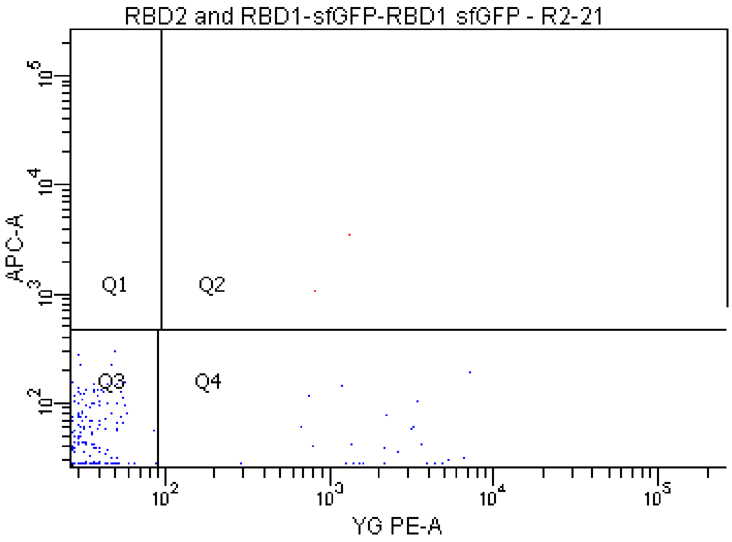

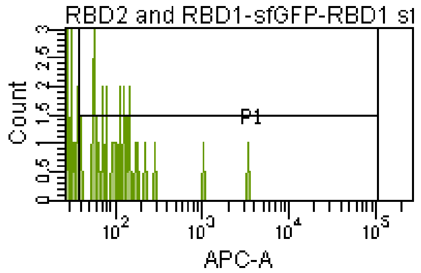

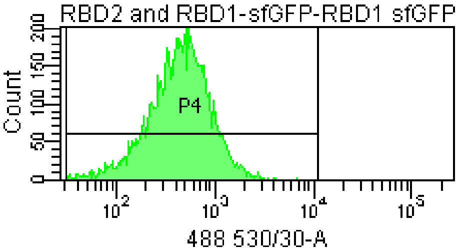

Experiment Name: Bio RBD2 selectons
Specimen Name: RBD2 and RBD1-sfGFP
Tube Name: RBD1 sfGFP - R2-21
Record Date: Jul 14, 2020 12:20:10 PM
$OP: Nileena
GUID: 51a5d0b4-583d-4c97-a6e5-9d9d96c249d1

Population	Parent...	#Events	%Parent	%Gran...	%Total	488 5... Mean	488 5... Median	PE-A Mean	PE-A Median	APC-A Mean	APC-A Median
All Events	####	####	####	####	####	818	494	101	36	25	10
P2	####	####	####	####	####	762	494	84	36	23	10
P8	P2	8,243	82.4	82.0	82.0	523	439	45	28	14	6
Q1	P8	1	0.0	0.0	0.0	985	985	60	60	1,228	1,228
Q2	P8	2	0.0	0.0	0.0	689	689	377	377	2,202	2,202
Q3	P8	8,216	99.7	82.2	81.7	522	438	42	28	13	6
Q4	P8	24	0.3	0.2	0.2	853	695	952	768	38	35
P7	P8	67	0.8	0.7	0.7	858	699	432	156	120	41
P1	P8	2,135	25.9	21.3	21.2	593	488	59	37	78	66
P4	P8	8,137	98.7	81.4	80.9	530	443	45	29	14	7

FACSDiva Version 6.1.3

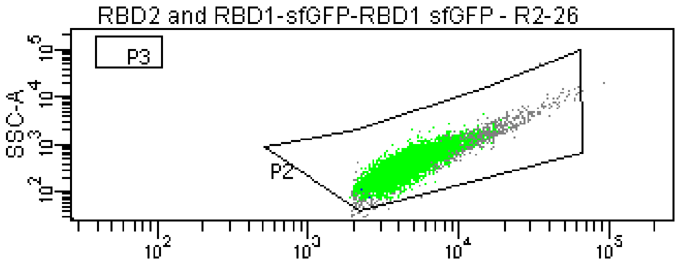

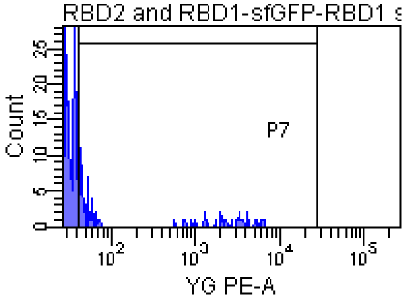

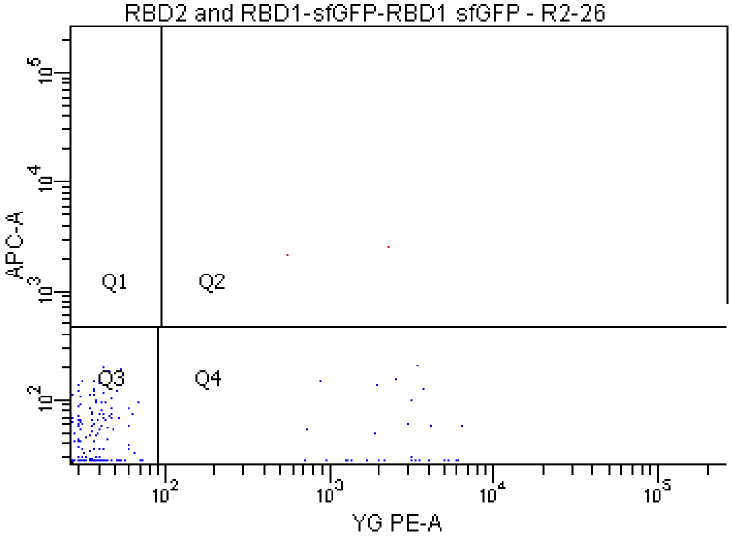

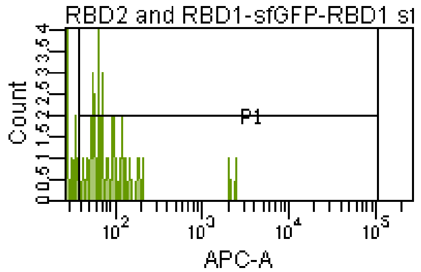

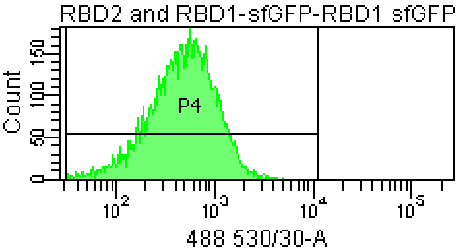

Experiment Name: Bio RBD2 selectons
Specimen Name: RBD2 and RBD1-sfGFP
Tube Name: RBD1 sfGFP - R2-26
Record Date: Jul 14, 2020 12:21:20 PM
$OP: Nileena
GUID: 182de319-1058-45ad-aeeb-423aaaeb8676

Population	Parent...	#Events	%Parent	%Gran...	%Total	488 5... Mean	488 5... Median	PE-A Mean	PE-A Median	APC-A Mean	APC-A Median
All Events	####	####	####	####	####	825	544	92	40	21	9
P2	####	####	####	####	####	816	545	88	40	20	9
P8	P2	8,201	82.0	81.8	81.8	578	474	52	33	13	6
Q1	P8	4	0.0	0.0	0.0	719	431	21	21	1,156	982
Q2	P8	2	0.0	0.0	0.0	1,080	1,080	480	480	2,220	2,220
Q3	P8	8,167	99.6	81.7	81.4	577	473	48	33	12	6
Q4	P8	28	0.3	0.3	0.3	814	725	1,002	883	36	23
P7	P8	77	0.9	0.8	0.8	793	741	452	166	100	37
P1	P8	2,045	24.9	20.4	20.4	679	559	68	46	78	65
P4	P8	8,060	98.3	80.6	80.4	589	484	52	34	13	6

FACSDiva Version 6.1.3

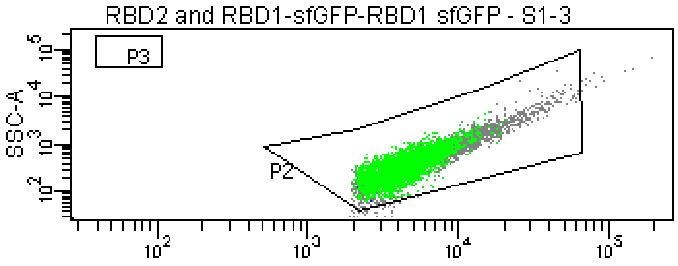

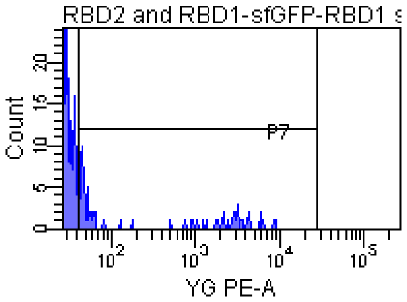

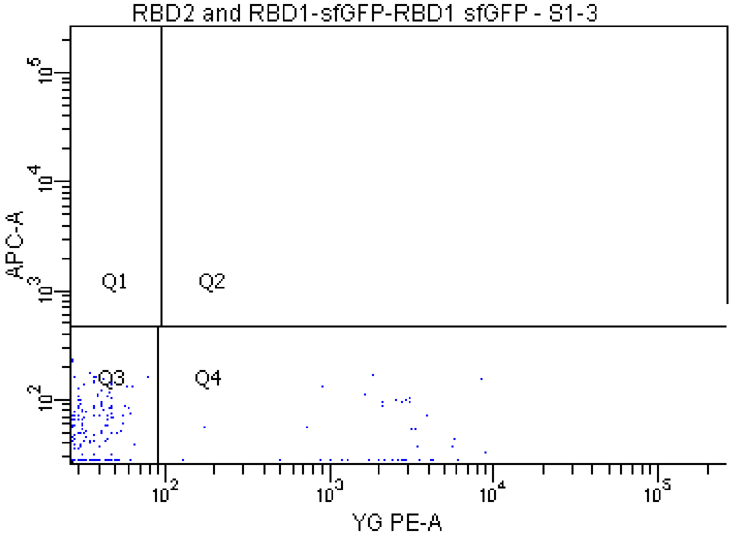

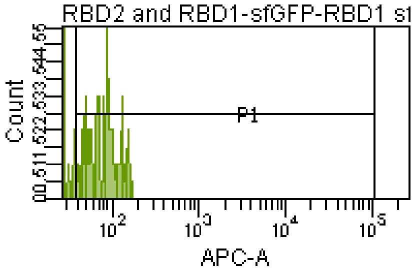

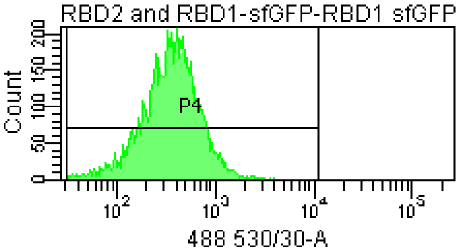

Experiment Name: Bio RBD2 selectons
Specimen Name: RBD2 and RBD1-sfGFP
Tube Name: RBD1 sfGFP - S1-3
Record Date: Jul 14, 2020 12:22:45 PM
$OP: Nileena
GUID: 1b18ff64-bd6a-41c4-a8ba-14fcf57a424c

Population	Parent...	#Events	%Parent	%Gran...	%Total	488 5... Mean	488 5... Median	PE-A Mean	PE-A Median	APC-A Mean	APC-A Median
All Events	####	####	####	####	####	698	408	137	44	29	14
P2	####	####	####	####	####	660	409	117	44	28	14
P8	P2	7,782	77.8	77.4	77.4	414	352	52	35	16	9
Q1	P8	0	0.0	0.0	0.0	####	####	####	####	####	####
Q2	P8	0	0.0	0.0	0.0	####	####	####	####	####	####
Q3	P8	7,743	99.5	77.4	77.0	410	351	47	35	16	9
Q4	P8	39	0.5	0.4	0.4	1,051	831	1,030	930	35	32
P7	P8	93	1.2	0.9	0.9	831	638	522	190	48	48
P1	P8	2,198	28.2	22.0	21.9	486	409	69	44	78	67
P4	P8	7,684	98.7	76.8	76.4	419	356	53	35	17	9

FACSDiva Version 6.1.3

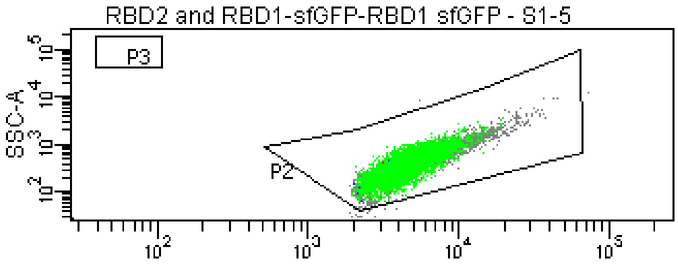

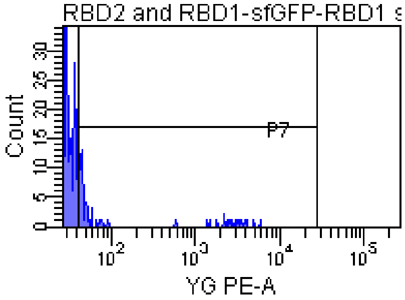

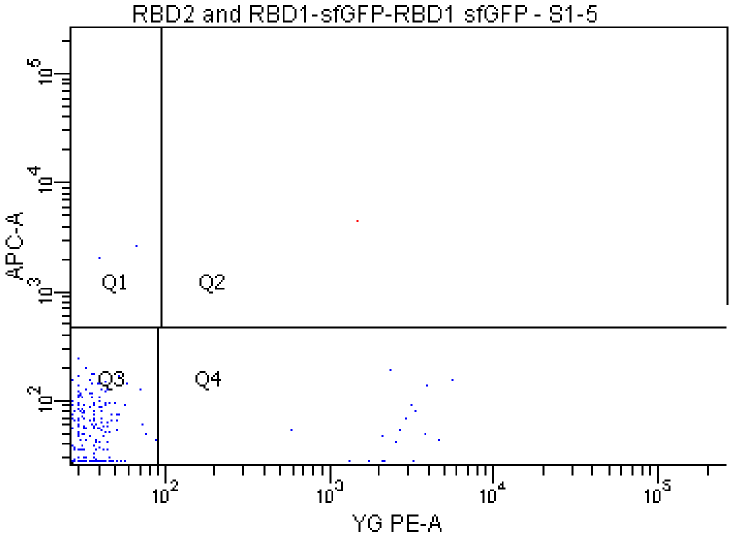

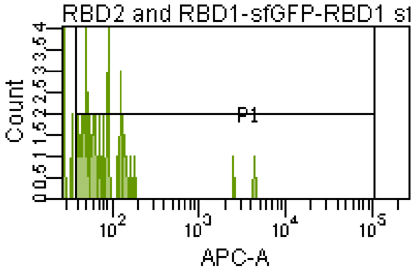

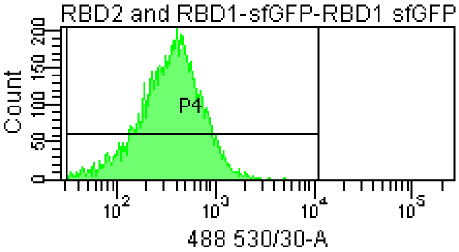

Experiment Name: Bio RBD2 selectons
Specimen Name: RBD2 and RBD1-sfGFP
Tube Name: RBD1 sfGFP - S1-5
Record Date: Jul 14, 2020 12:25:05 PM
$OP: Nileena
GUID: d61a23b0-e388-4c75-9fff-25896d9dbf08

Population	Parent...	#Events	%Parent	%Gran...	%Total	488 5... Mean	488 5... Median	PE-A Mean	PE-A Median	APC-A Mean	APC-A Median
All Events	####	####	####	####	####	517	384	66	35	20	9
P2	####	####	####	####	####	517	384	66	35	20	9
P8	P2	8,431	84.3	84.1	84.1	423	354	48	29	15	8
Q1	P8	6	0.1	0.1	0.1	471	537	53	52	1,801	1,815
Q2	P8	1	0.0	0.0	0.0	1,332	1,332	497	497	4,272	4,272
Q3	P8	8,407	99.7	84.1	83.8	422	353	46	29	13	8
Q4	P8	17	0.2	0.2	0.2	934	826	967	905	54	47
P7	P8	73	0.9	0.7	0.7	765	651	343	143	146	50
P1	P8	2,211	26.2	22.1	22.0	514	420	69	43	81	65
P4	P8	8,260	98.0	82.6	82.4	432	360	48	31	16	8

FACSDiva Version 6.1.3

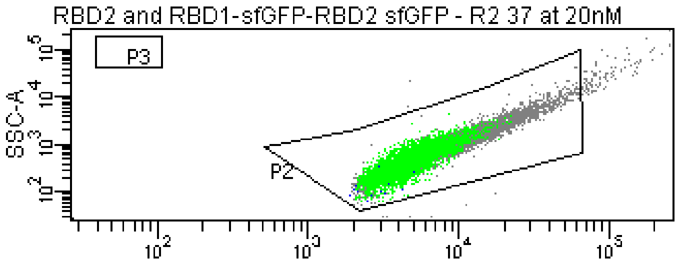

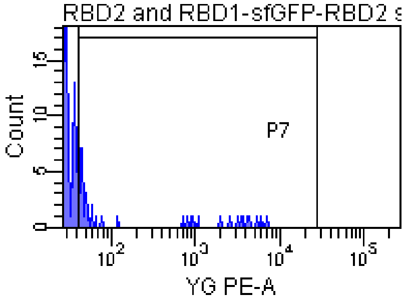

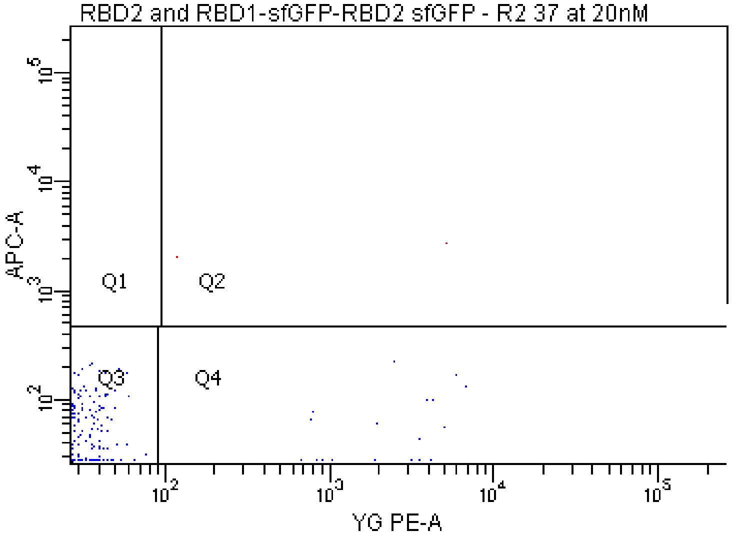

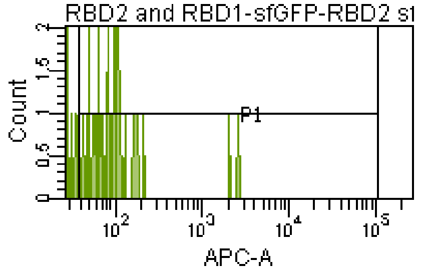

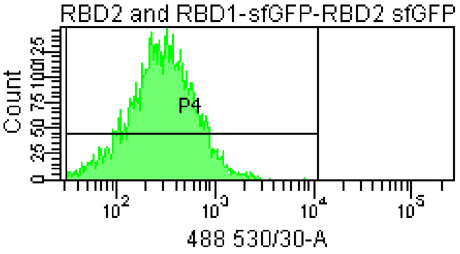

Experiment Name: Bio RBD2 selectons
Specimen Name: RBD2 and RBD1-sfGFP
Tube Name: RBD2 sfGFP - R2 37 at 20nM
Record Date: Jul 14, 2020 4:58:22 PM
$OP: Nileena
GUID: c73a8712-ae5d-4656-9436-40ebe4cce9cd

Population	Parent...	#Events	%Parent	%Gran...	%Total	488 5... Mean	488 5... Median	PE-A Mean	PE-A Median	APC-A Mean	APC-A Median
All Events	####	####	####	####	####	1,044	405	446	64	60	21
P2	####	####	####	####	####	765	401	210	63	42	20
P8	P2	6,660	66.6	65.8	65.8	358	279	69	40	16	10
Q1	P8	2	0.0	0.0	0.0	556	556	53	53	2,484	2,484
Q2	P8	2	0.0	0.0	0.0	4,536	4,536	958	958	2,293	2,293
Q3	P8	6,638	99.7	66.4	65.6	356	278	67	40	15	9
Q4	P8	18	0.3	0.2	0.2	833	841	1,015	929	54	47
P7	P8	51	0.8	0.5	0.5	814	609	475	167	150	56
P1	P8	1,736	26.1	17.4	17.2	470	362	103	54	80	66
P4	P8	6,471	97.2	64.7	64.0	369	287	71	41	17	10

FACSDiva Version 6.1.3

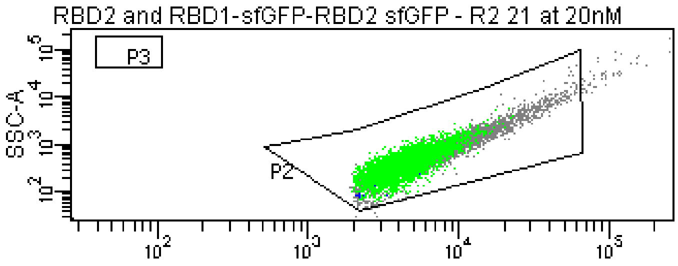

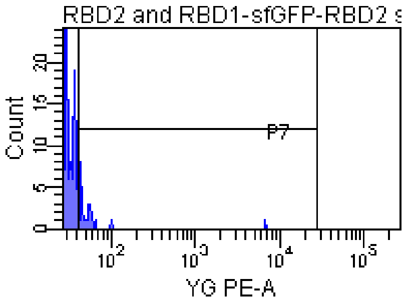

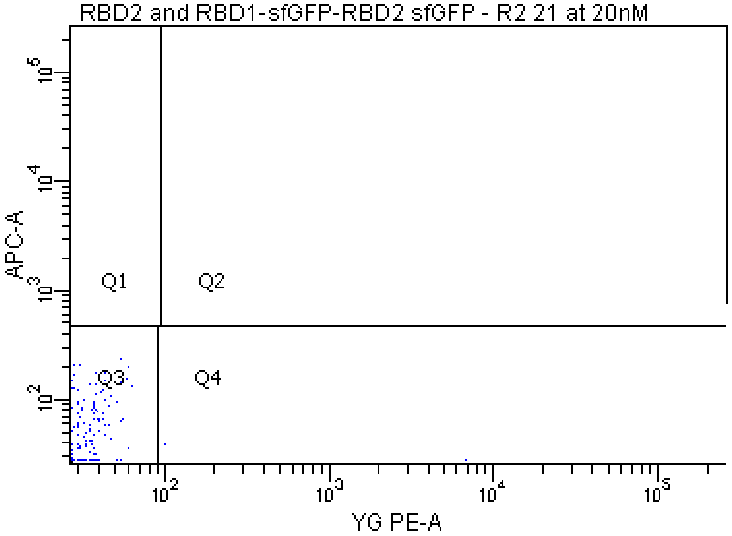

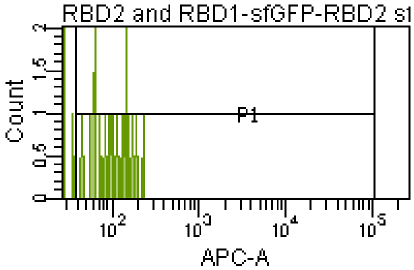

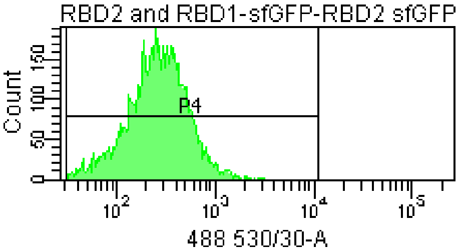

Experiment Name: Bio RBD2 selectons
Specimen Name: RBD2 and RBD1-sfGFP
Tube Name: RBD2 sfGFP - R2 21 at 20nM
Record Date: Jul 14, 2020 5:00:42 PM
$OP: Nileena
GUID: 65d4e1bc-507c-46a0-8dc5-2d0831e14b1d

Population	Parent...	#Events	%Parent	%Gran...	%Total	488 5... Mean	488 5... Median	PE-A Mean	PE-A Median	APC-A Mean	APC-A Median
All Events	####	####	####	####	####	692	308	219	41	44	15
P2	####	####	####	####	####	560	307	128	41	35	15
P8	P2	7,671	76.7	76.2	76.2	306	254	44	31	16	8
Q1	P8	2	0.0	0.0	0.0	1,293	1,293	326	326	1,003	1,003
Q2	P8	0	0.0	0.0	0.0	####	####	####	####	####	####
Q3	P8	7,667	99.9	76.7	76.2	306	254	44	31	16	8
Q4	P8	2	0.0	0.0	0.0	782	782	1,156	1,156	10	10
P7	P8	27	0.4	0.3	0.3	513	446	194	111	86	72
P1	P8	2,121	27.6	21.2	21.1	365	293	61	38	77	66
P4	P8	7,406	96.5	74.1	73.6	317	261	45	32	16	8

FACSDiva Version 6.1.3

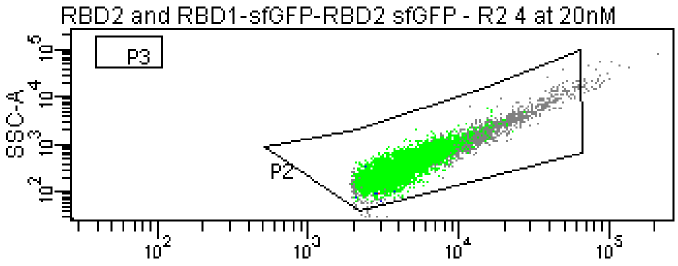

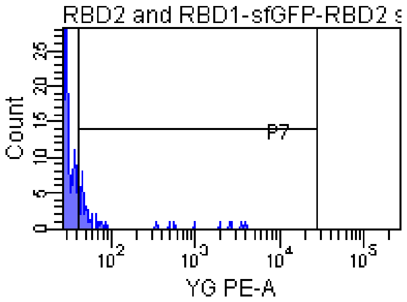

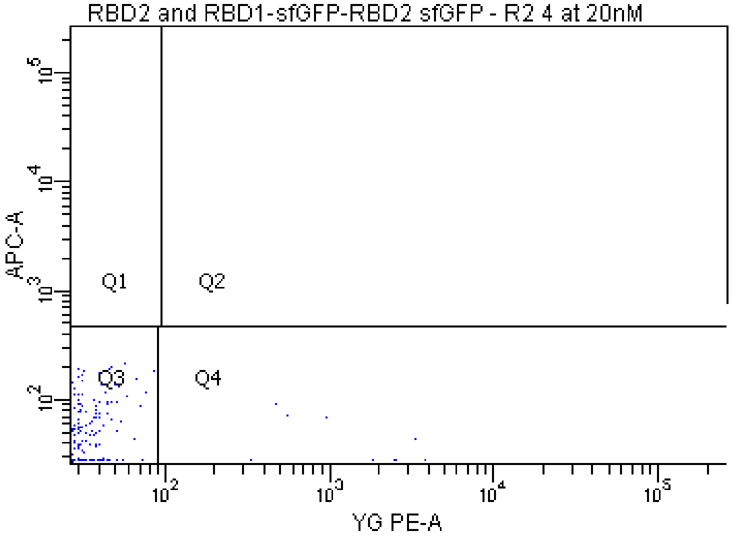

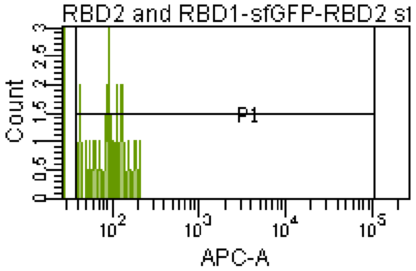

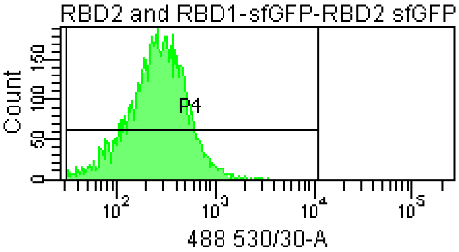

Experiment Name: Bio RBD2 selectons
Specimen Name: RBD2 and RBD1-sfGFP
Tube Name: RBD2 sfGFP - R2 4 at 20nM
Record Date: Jul 14, 2020 5:02:40 PM
$OP: Nileena
GUID: b1b46b13-76ab-4e64-b2cd-6d0903cc8681

Population	Parent...	#Events	%Parent	%Gran...	%Total	488 5... Mean	488 5... Median	PE-A Mean	PE-A Median	APC-A Mean	APC-A Median
All Events	####	####	####	####	####	586	303	151	38	43	14
P2	####	####	####	####	####	528	303	114	38	39	14
P8	P2	7,795	78.0	77.6	77.6	304	254	41	28	15	8
Q1	P8	0	0.0	0.0	0.0	####	####	####	####	####	####
Q2	P8	0	0.0	0.0	0.0	####	####	####	####	####	####
Q3	P8	7,786	99.9	77.9	77.5	304	254	41	28	15	8
Q4	P8	9	0.1	0.1	0.1	760	848	664	734	35	23
P7	P8	42	0.5	0.4	0.4	614	458	256	148	72	67
P1	P8	2,095	26.9	21.0	20.8	369	299	56	37	76	66
P4	P8	7,561	97.0	75.6	75.2	314	262	42	29	16	9
